# Diastereoselective desymmetric 1,2-*cis*-glycosylation of *meso*-diols via chirality transfer from a glycosyl donor

**DOI:** 10.1038/s41467-020-16365-8

**Published:** 2020-05-15

**Authors:** Masamichi Tanaka, Koji Sato, Ryoki Yoshida, Nobuya Nishi, Rikuto Oyamada, Kazuki Inaba, Daisuke Takahashi, Kazunobu Toshima

**Affiliations:** Department of Applied Chemistry, Faculty of Science and Technology, Keio University, 3-14-1 Hiyoshi, Kohoku-ku Yokohama, 223-8522 Japan

**Keywords:** Synthetic chemistry methodology, Carbohydrate chemistry, Natural product synthesis

## Abstract

Chemical desymmetrization reactions of *meso*-diols are highly effective for the precise and efficient synthesis of chiral molecules. However, even though enzyme-catalyzed desymmetric glycosylations are frequently found in nature, there is no method for highly diastereoselective desymmetric chemical glycosylation of *meso*-diols. Herein, we report a highly diastereoselective desymmetric 1,2-*cis*-glycosylation of *meso*-diols found in *myo*-inositol 1,3,5-orthoesters using a boronic acid catalyst based on predictions of regioselectivity by density functional theory (DFT) calculations. The enantiotopic hydroxyl groups of the *meso*-diols are clearly differentiated by the stereochemistry at the C2 position of the glycosyl donor with excellent regioselectivities. In addition, the present method is successfully applied to the synthesis of core structures of phosphatidylinositolmannosides (PIMs) and glycosylphosphatidylinositol (GPI) anchors, and common β-mannoside structures of the LLBM-782 series of antibiotics.

## Abstract (web summary)

Enzyme-catalyzed desymmetric glycosylations are often found in nature, however the corresponding chemical methods are lacking. Here, the authors report a highly diastereoselective desymmetric 1,2-*cis*-glycosylation of *meso*-diols found in *myo*-inositol 1,3,5-orthoesters using a boronic acid catalyst.

## Introduction

**Fig. 1:**
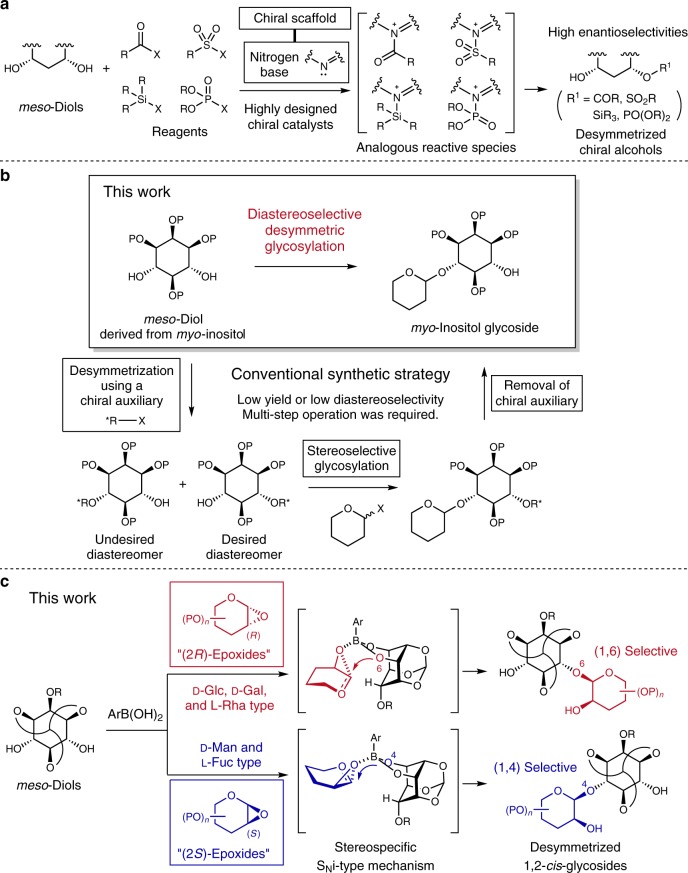
Enantio- or diastereoselective functionalizations of *meso*-diols. **a** Highly enantioselective functionalizations of *meso*-diols using highly designed chiral catalysts. **b** Synthetic strategy for *myo*-inositol glycoside from a *meso*-diol. **c** Boronic-acid-catalyzed desymmetric 1,2-*cis*-glycosylations of *meso*-diols. X leaving group, P protecting group, Ar aryl, Glc glucose, Gal galactose, Rha rhamnose, Man mannose, Fuc fucose.

The desymmetrization of *meso*-diols is an attractive method for preparing chiral molecules bearing multiple stereogenic centers in one operation. Highly designed chiral catalysts possessing a nitrogen base on the chiral scaffold allow for the enantioselective functionalization^1,2^ of *meso*-diols by desymmetrization reactions, such as acylation^3^, silylation^4,5^, and sulfonylation^6^ (Fig. 1a). In addition, other approaches, such as oxidation^7–9^, Heck reaction^10^, and lipase-catalyzed selective acylation^11^ were also reported. However, the repertoire of enantio- or diastereoselective functionalization reactions of *meso*-diols is still limited. Therefore, to efficiently synthesize diversified valuable compounds from *meso*-diols, a new repertoire of desymmetric reactions has been in great demand. In this context, our interest was directed toward biosynthetic reactions that provide many biologically active natural products derived from *meso*-compounds. In nature, *meso*-compounds representing *myo*-inositol and 2-deoxystreptamine derivatives have been modified by enzymatic desymmetrization reactions, such as phosphorylation^12^ and glycosylation^13^. With regard to phosphorylation, Miller et al. focused on the analogy of key intermediates between phosphorylation and acylation, and developed a pioneering highly enantioselective desymmetric phosphorylation of a *myo*-inositol derivative using chiral peptide catalysts discovered by screening a peptide library^14,15^. Recently, Fukase and Fujimoto et al. have reported another method for desymmetric phosphorylation of a *myo*-inositol derivative^16^. On the other hand, for glycosylation, despite the existence of many natural glycosides derived from *meso*-compounds, there is no method for the highly diastereoselective desymmetric glycosylation of *meso*-diols.

Highly diastereoselective desymmetric glycosylations of *meso*-diols can greatly increase the efficiency with which desymmetrized *meso*-compounds by glycosylations are prepared. As an example, the conventional synthetic strategy of *myo*-inositol glycoside is shown in Fig. 1b. In this strategy, *myo*-inositol glycosides were synthesized using a chiral auxiliary^17,18^. A *meso*-diol derived from *myo*-inositol was first functionalized using a chiral auxiliary, followed by stereoselective glycosylation of the desired diastereomer and finally, removal of the chiral auxiliary to give the desired *myo*-inositol glycoside. However, most desymmetrization methods of *myo*-inositol derivatives using a chiral auxiliary proceeded with low regioselectivity, providing a mixture with an undesired diastereomer. In addition, at least three steps were required to introduce the sugar moiety into the desired position in the *myo*-inositol derivatives. In another approach, optically pure *myo*-inositols were synthesized from d-glucose through Ferrier rearrangement^19^, however, a tedious multi-step operation was required. Thus, a highly diastereoselective desymmetric glycosylation of *meso*-diols would provide the desired *myo*-inositol glycosides in only one step.

In the development of a desymmetric glycosylation of *meso*-diols, it is challenging to completely and simultaneously control both the α/β stereoselectivity of the anomeric center and the regioselectivity of the glycosylation site. Efficient stereoselective glycosylation methods have been developed for the α/β stereoselectivity^20^. However, there is no efficient desymmetric glycosylation method that can predict and concisely control the regioselectivity in the reaction with *meso*-diols. Although several cases of desymmetric α-mannosylations of *meso*-diols have been reported so far, the regioselectivities were low to moderate^21,22^. In addition, the regioselectivities of these glycosylations using other substrates are unpredictable because detailed reaction mechanisms and transition states during the glycosylations are still unclear.

In this context, we focus on our organoboron-catalyzed 1,2-*cis*-glycosylations of 1,2-anhydro donors and mono-, di-, and poly-ol sugar acceptors^23–29^. The combination of a 1,2-anhydro donor and a boronic or borinic acid catalyst in the glycosylation gives excellent 1,2-*cis*-stereoselectivities. In addition, the S_N_i-type mechanism and the transition states are supported by mechanistic studies. Thus, we expect that the glycosylation of a 1,2-anhydro donor and a *meso*-diol using a boronic acid catalyst will give a 1,2-*cis*-glycoside with high stereoselectivity via the S_N_i-type mechanism, and the regioselectivity will be predictable by analyzing transition states with density functional theory (DFT) calculations. Herein, we report a highly diastereoselective desymmetric 1,2-*cis*-glycosylation of *meso*-diols found in *myo*-inositol 1,3,5-orthoesters using a boronic acid catalyst based on predictions of regioselectivity by DFT calculations (Fig. 1c). The enantiotopic hydroxyl groups of the *meso*-diols are clearly differentiated by the stereochemistry at the C2 position of the 1,2-anhydro glycosyl donor with excellent regioselectivity. In addition, the present method is successfully applied to the synthesis of core structures of phosphatidylinositolmannosides (PIMs) and glycosylphosphatidylinositol (GPI) anchors, and common β-mannoside structure of the LLBM-782 series of antibiotics.

## Results

### Desymmetric glycosylations of *myo*-inositol derivatives

**Fig. 2:**
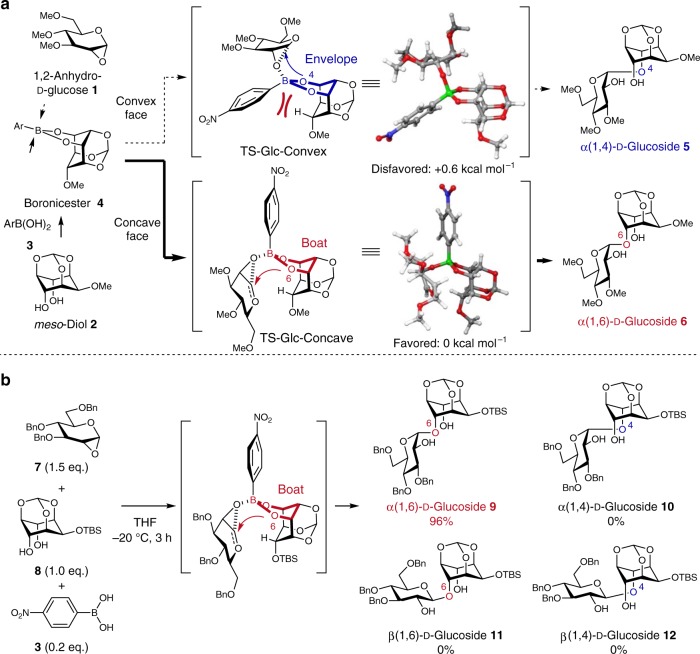
Desymmetric glycosylation using 1,2-anhydro-d-glucose. **a** Transition states calculated using B3LYP/6-31G* (single point energy: B3LYP/6-31+G**). **b** Desymmetric glycosylation of **7** and **8**. TS transition state, Me methyl, Bn benzyl, THF tetrahydrofuran, TBS *tert*-butyldimethylsilyl.

To investigate our hypothesis, we first selected 1,2-anhydro-d-glucose **1**, 2-*O*-methyl-*myo*-inositol 1,3,5-orthoformate (**2**), and 4-nitrophenylboronic acid (**3**) as the glycosyl donor, *meso*-diol acceptor, and arylboronic acid, respectively, and investigated the transition states by DFT calculations (Fig. 2a, Supplementary Fig. 41). It was found that donor **1** could approach the boron atom from either the convex or concave face of boronic ester **4**, forming reasonable transition states in which the anomeric center of the donor is near the oxygen atom of the 4 position (TS-Glc-Convex) or 6 position (TS-Glc-Concave), respectively. The difference in activation energy was found to be 0.6 kcal mol^−1^. These results indicated that the glycosylation at the 6 position via TS-Glc-Concave was favored over glycosylation at the 4 position via TS-Glc-Convex. The activation energy difference was caused by the ring strain in the envelope conformation of the dioxaborinane ring in TS-Glc-Convex, which was induced by steric hindrance between the aryl group of the boronic acid and the hydrogen atom at the 2 position of the *meso*-diol **2**. Therefore, we expected that the desymmetric glycosylation of 1,2-anhydro-d-glucose **7** and *meso*-diol **8** would proceed regioselectively to give the corresponding α(1,6)-d-glucoside **9** via the favored boat-type transition state similar to TS-Glc-Concave.

To investigate our prediction, the desymmetric glycosylation of **7** and **8** using a catalytic amount of **3** was examined (Fig. 2b, Supplementary Table 1). It was found that the glycosylation proceeded smoothly to provide α(1,6)-d-glucoside **9** in 96% yield with excellent regio- and stereoselectivities without forming any other regio- and stereoisomers **10**–**12** (Supplementary Figs. 2, 3, and 6–8, Supplementary Tables 2 and 3). This observed regioselectivity was good agreement with the calculation result using the model compounds (Fig. 2a). Since 1,2-anhydro donor **7** was the only chiral source in this glycosylation, the absolute configuration of stereogenic center(s) in the glycosyl donor was an origin of the excellent regioselectivity of this glycosylation. This result indicated that the chirality of the glycosyl donor was completely transferred to the *meso*-diol, leading to the clear differentiation of enantiotopic hydroxyl groups in *meso*-diols.

**Fig. 3:**
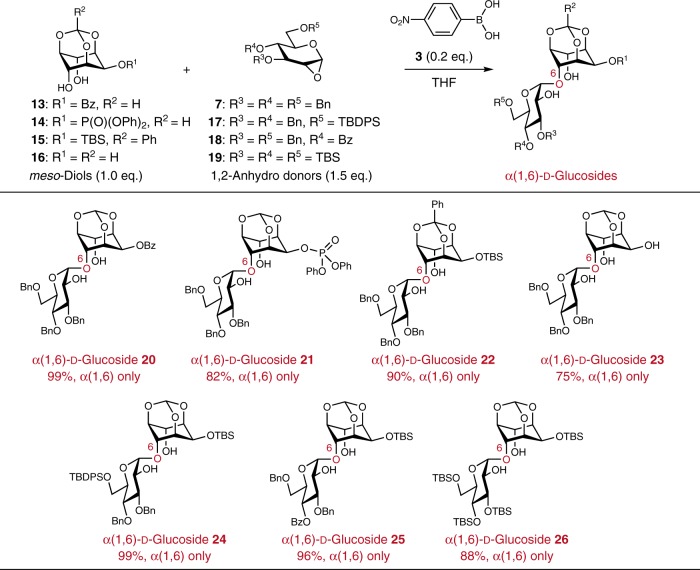
Substrate scope of the protecting groups in the desymmetric glycosylation using 1,2-anhydro-d-glucose. Bz benzoyl, Ph phenyl, TBDPS tert-butyldiphenylsilyl.

The effect of protecting groups of the *meso*-diol using **13**–**15** was also examined (Fig. 3). The corresponding α(1,6)-d-glucosides **20**–**22** were obtained as single isomers in high yields (Supplementary Figs. 2, 4, and 9–12, Supplementary Tables 4–9). In addition, even when triol **16** possessing a more reactive hydroxyl group at 2 position was used, α(1,6)-d-glucoside **23** was obtained with excellent diastereoselectivity (Supplementary Tables 10 and 11). This result indicated that the activation of 4,6-diol by the boronate ester formation was more important for the glycosylation than the innate reactivity of hydroxyl group. Next, the effect of protecting groups of the glycosyl donor using **17**–**19** (synthesis of **18** was described in Supplementary Fig. 1) was examined. In these cases, also, the desymmetric glycosylations proceeded effectively to give the corresponding α(1,6)-d-glucosides **24**–**26** with high regio- and 1,2-*cis*-stereoselectivities in high yields (Supplementary Figs. 2, 5, and 13–15, Supplementary Tables 12–17). These results clearly indicated that the regioselectivity of the present glycosylations was not affected by the protecting groups.

### Construction of a prediction model

**Fig. 4:**
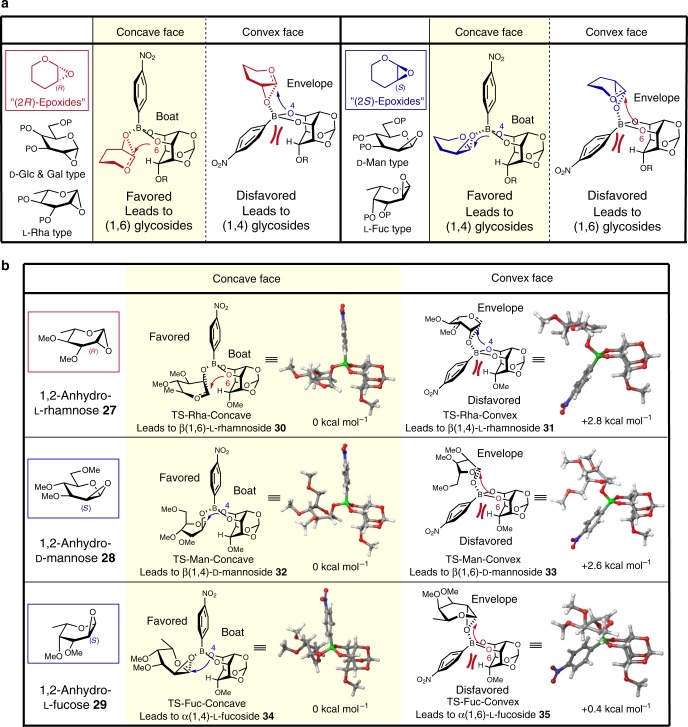
Desymmetric glycosylation using several 1,2-anhydro donors. **a** Prediction model for regioselectivity. **b** Transition states calculated using B3LYP/6-31G* (single point energy: B3LYP/6-31+G**). The atoms colored gray, white, blue, red, and green in transition states represent C, H, N, O, and B, respectively.

We hypothesized that the regioselectivity was mainly affected by the C2 configuration of the glycosyl donor, and constructed a prediction model for the regioselectivities using (2*R*)-epoxide donors, such as 1,2-anhydro-d-glucose, d-galactose, and l-rhamnose, and (2*S*)-epoxide donors, such as 1,2-anhydro-d-mannose and l-fucose, as shown in Fig. 4a. According to this model, it was expected that the glycosylations of (2*R*)-epoxide donors would occur at the 6 position via boat-type transition states. On the other hand, in the cases of 1,2-anhydro donors possessing S configuration at the C2 position, the regioselectivity would be reversed and the glycosylations would occur at the 4 position.

To validate this prediction model, we analyzed the boronic acid **3** catalyzed glycosylations of *meso*-diol **2** with several 1,2-anhydro glycosyl donors **27**–**29** by DFT calculations (Fig. 4b). Using 1,2-anhydro-l-rhamnose **27**, two transition states were found in which the donor approached from the concave and convex faces of boronic ester **4**, i.e., TS-Rha-Concave which leads to β(1,6)-l-rhamnoside **30** and TS-Rha-Convex which leads to β(1,4)-l-rhamnoside **31**. As with 1,2-anhydro-d-glucose **1**, TS-Rha-Concave was favored over TS-Rha-Convex by 2.8 kcal mol^−1^ due to a similar ring strain of the envelope conformation, indicating that β-rhamnosylation using 1,2-anhydro-l-rhamnose would occur at the 6 position. Also, when 1,2-anhydro-d-mannose **28** and l-fucose **29** possessing the *S* configuration at the C2 position were used, the approach of these donors from the concave face to the boron atom was favored, similar to the (2*R*)-epoxide donor, and the anomeric center of the glycosyl donor was positioned near the 4 position in the favored TSs (TS-Man-Concave and TS-Fuc-Concave) as expected, indicating that β-mannosylation and α-fucosylation would proceed regioselectively at the 4 position. These predictions of regioselectivities by DFT calculations were in good agreement with the prediction model, suggesting that the regioselectivity of the present glycosylation reaction was attributed to the stereochemistry at C2 of the glycosyl donor.

**Fig. 5:**
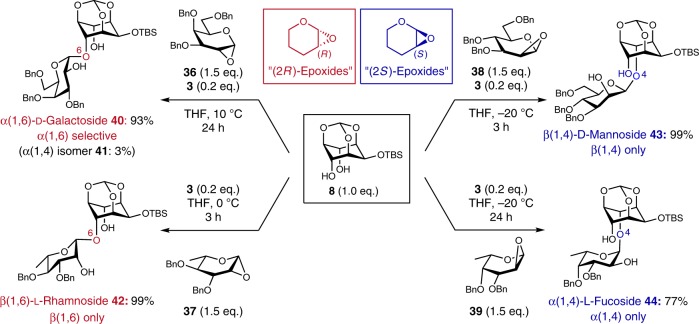
Substrate scope of the glycosyl donors in the desymmetric glycosylation of **8**. In all cases, the formation of 1.2-*trans* glycoside was not observed.

In fact, we investigated the glycosylations of several 1,2-anhydro donors and *meso*-diol **8** (Fig. 5). Using 1,2-anhydro-d-galactose **36**, similarly to 1,2-anhydro-d-glucose **7**, α(1,6)-d-galactoside **40** was obtained in high yield with high regio- and stereoselectivities (Supplementary Figs. 16–18, Supplementary Tables 18 and 19). The use of 1,2-anhydro-l-rhamnose **37** gave β(1,6)-l-rhamnoside **42** as a single isomer as expected (Supplementary Figs. 19–21, Supplementary Tables 20 and 21). Also, when 1,2-anhydro-d-mannose **38** was used, β-mannosylation occurred at the 4 position in **8** according to our prediction model, and β(1,4)-d-mannoside **43** was obtained with complete regio- and stereoselectivities (Supplementary Figs. 22–24, Supplementary Tables 22 and 23). Similarly, the glycosylation using 1,2-anhydro-l-fucose **39** proceeded regioselectively to give α(1,4)-l-fucoside **44** in good yield (Supplementary Figs. 25–27, Supplementary Tables 24, 25). All of these obtained experimental regioselectivities were in good agreement with our prediction model, clearly demonstrating that the regioselectivity of the present desymmetric glycosylation can be easily predicted.

### Application to acyclic *meso*-diols

**Fig. 6:**
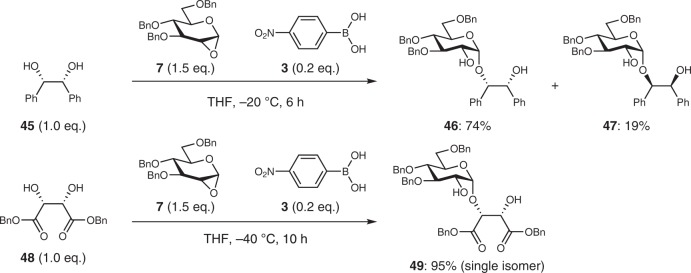
Desymmetric glycosylation using acyclic *meso*-diols. In both cases, the formation of 1.2-*trans* glycoside was not observed.

Next, as a preliminary attempt, we examined the desymmetric glycosylations of acyclic *meso*-diols, which possess high conformational flexibility, to show the possibility of the present glycosylation method (Fig. 6). The results showed that when acyclic *meso*-diols **45** and **48** were used, the glycosylations proceeded smoothly to provide the corresponding 1,2-*cis*-glycosides in high yields with moderate and excellent regioselectivities, respectively, and excellent 1,2-*cis*-α-stereoselectivities (Supplementary Figs. 30–37, Supplementary Tables 28–31). Although the rationale of regioselectivities was still unclear, these results showed the possibility that this glycosylation method could be applicable to acyclic *meso*-diols. Further investigations using several acyclic *meso*-diols is now in progress in our laboratory.

### Synthesis of core structures of PIMs and GPI anchors

**Fig. 7:**
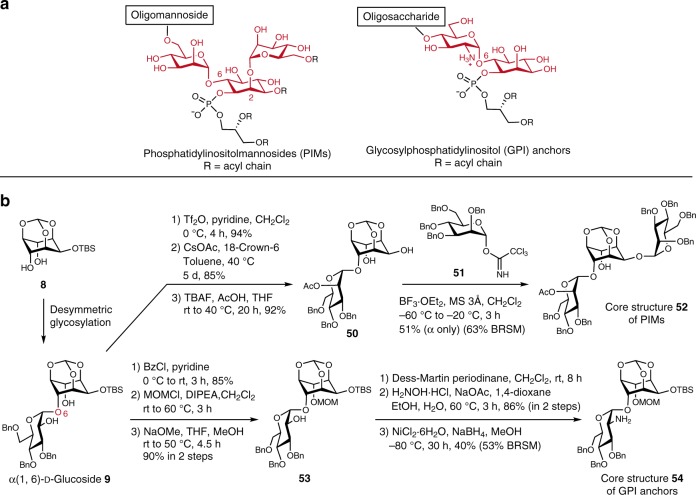
Synthetic scheme of core structures of PIMs and GPI anchors. **a** Chemical structures of PIMs and GPI anchors. **b** Synthesis of core structures **52** and **54** using the present glycosylation method. Tf trifluoromethanesulfonyl, Ac acetyl, TBAF tetra-*n*-butylammonium fluoride, Et ethyl, MS molecular sieve, MOM methoxymethyl, DIPEA *N*,*N*-diisopropylethylamine, BRSM based on recovered starting material.

Overall, we developed the highly diastereoselective desymmetric 1,2-*cis*-glycosylation, which can be able to synthesize various 1,2-*cis*-glycosides. Next, we focused on the representative *myo*-inositol glycosides, PIMs and GPI anchors (Fig. 7a). Although several synthetic strategies have been developed for PIMs^19,21,30–39^ and GPI anchors^17,18,40–46^, which possess α-mannose and α-glucosamine at 6 position of *myo*-inositol, respectively, there are few efficient methods for highly regio- and stereoselective introduction of these sugars. Therefore, the efficient synthesis of core structures of PIMs and GPI anchors using the present glycosylation as a key step was examined (Fig. 7b, Supplementary Figs. 38 and 39). Initially, selective triflation of **9** and displacement using CsOAc, followed by deprotection of TBS group, afforded α(1,6)-d-mannoside **50**. Mannosylation at the 2 position in inositol moiety provided core structure **52** of PIMs. In addition, protection of **9** using benzoyl and methoxymethyl (MOM) groups, followed by deprotection of benzoyl group, gave **53**. Finally, oxidation and oximation, followed by reduction, afforded core structure **54** of GPI anchors. These results indicated that the present glycosylation method could lead to synthesize PIMs and GPI anchors effectively.

### Synthesis of common structure of the LLBM-782 series

**Fig. 8:**
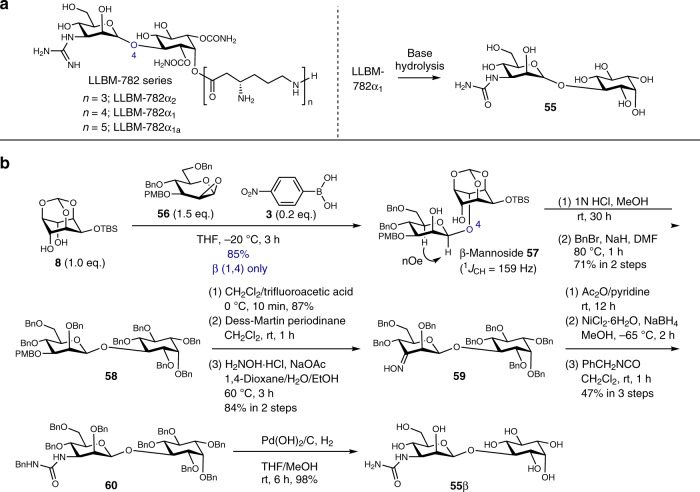
Synthetic scheme of mannoside **55**β. **a** Chemical structures of LLBM-782 series and mannoside **55** produced by base hydrolysis of LLBM-782α_1_. **b** Synthesis of mannoside **55**β using the present glycosylation method. PMB *p*-methoxybenzyl, DMF dimethylformamide.

Finally, we applied the glycosylation method to the synthesis and structural determination of a common mannosyl inositol structure of the LLBM-782 series of antibiotics^47–49^. The β configuration of the anomeric center was supported by the ^1^*J*_CH_ value of LLBM-782α_2_ (163.9 Hz)^48,49^. However, in general, it is difficult to judge the anomeric configuration to be α or β using this ambiguous ^1^*J*_CH_ value^50,51^. Therefore, in order to determine the anomeric configuration of the LLBM-782 series, we planned to synthesize common β-mannoside structure **55**β of the LLBM-782 series using the present glycosylation method, and compare it to mannoside **55**, which was provided by base hydrolysis of LLBM-782α_1_^47^ as shown in Fig. 8a.

The synthetic scheme of **55**β is shown in Fig. 8b (Supplementary Fig. 40). Desymmetric glycosylation of 1,2-anhydro-d-mannose **56** and *meso*-diol **8** proceeded efficiently to provide β(1,4)-d-mannoside **57** in high yield as a single isomer (Supplementary Figs. 28 and 29, Supplementary Tables 26 and 27). The anomeric configuration of **57** was confirmed by ^1^*J*_CH_ of the anomeric center (159 Hz) and nOe experiments. Deprotection of the orthoformate and TBS groups, followed by benzylation, gave **58** in good yield. Deprotection of the PMB group and oxidation, followed by oximation, afforded oxime **59**. Acetylation, reduction, and carbamoylation gave protected urea **60**. Deprotection of the benzyl groups in **60** furnished structurally defined β-mannoside **55**β. The ^13^C-NMR spectrum of the analytical sample of **55**β was identical to the reported data for mannoside **55**^47^ (Supplementary Table 32), indicating that the anomeric configuration of LLBM-782 series is β.

## Discussion

In conclusion, we developed a method for the diastereoselective desymmetric 1,2-*cis*-glycosylation of *meso*-diols by using a combination of 1,2-anhydro donors and boronic acid catalyst. High regioselectivities of the glycosylations were predicted by DFT calculations, and those were in good agreement with our experimental regioselectivities. The difference in regioselectivities using several 1,2-anhydro donors was attributed to the stereochemistry at the C2 position of the glycosyl donor. The present glycosylation method was applied to the synthesis of core structures of PIMs and GPI anchors, and common β-mannoside structure **55**β of the LL-BM782 series of antibiotics. Further applications of this glycosylation method to the synthesis of various glycosides possessing *meso*-structures are now in progress in our laboratory.

## Methods

### Materials

For ^1^H- and ^13^C-NMR spectra of compounds in this study, see Supplementary Information (Supplementary Figs. 42–125). For the detailed synthetic procedures and methods of DFT calculations, see Supplementary Information. For the data of DFT calculations, see Supplementary Dataset 1.

### General procedure for desymmetric glycosylation

To a solution of *meso*-diol (0.02–0.05 mmol, 1.0 equiv.) and *p*-nitrophenylboronic acid (**3**) (4–10 μmol, 0.2 equiv.) in dry THF (0.2 M to *meso*-diol) was added a solution of 1,2-anhydro donor (0.03–0.15 mmol, 1.5–3.0 equiv.) in dry THF (0.2 M to 1,2-anhydro donor) at the temperature indicated under Ar atmosphere. After the reaction mixture was stirred for 3 h, the reaction was quenched by addition of 0.05 M NaBO_3_ aq. (8.8–22 μmol, 0.44 equiv.). To the resultant mixture was added sat. NH_4_Cl aq. (2 mL). The aqueous layer was extracted with EtOAc (3 mL × 3), and then the combined extracts were washed with brine (5 mL), dried over anhydrous Na_2_SO_4_, and concentrated in *vacuo*. The crude material was purified by column chromatography on SiO_2_.

## Supplementary information

Supplementary Information

Description of Additional Supplementary Files

Supplementary Data 1

## Data Availability

The data supporting the findings of this study are available within the paper and its Supplementary Information and Supplementary Data 1 files.
