# Supplementary material for: Diastereoselective desymmetric 1,2-cis-glycosylation of meso-diols via chirality transfer from a glycosyl donor
Source: Nat Commun. 2020 May 15;11:2431. doi: 10.1038/s41467-020-16365-8 (PMC7229163; doi:10.1038/s41467-020-16365-8)
Supplement: Supplementary file 1 — Supplementary Information [file 41467_2020_16365_MOESM1_ESM.pdf]

Supplementary Information

Diastereoselective desymmetric 1,2-*cis*-glycosylation of *meso*-diols via chirality transfer from a glycosyl donor

Tanaka *et al.*

(Correspondence to: dtak@applc.keio.ac.jp, toshima@applc.keio.ac.jp)

Contents

Supplementary Methods

General Information P. 2

Synthesis of *meso*-Diol **14**, 1,2-Anhydro Donor **18**, and 1,2-Anhydro Donor **39** P. 3

General Procedure for Optimization of Desymmetric 1,2-*cis*-Glycosylation of **7** and **8** P. 6

Desymmetric 1,2-*cis*-Glycosylation Reactions of Several 1,2-Anhydro Donors and *meso*-Diols P. 8

Determination of Glycosylation Site P. 19

Synthesis of Core Structures of PIMs and GPI Anchors P. 94

Synthesis of Common Mannosyl Structure **55β** P. 101

DFT Calculations P. 107

NMR Spectral Charts P. 108

Supplementary References P. 191

Supplementary Methods

General Information

NMR spectra were recorded on a JEOL ECA-500 (500 MHz for ^{1}H, 125 MHz for ^{13}C) or JEOL ECZ-400 (400 MHz for ^{1}H, 100 MHz for ^{13}C) spectrometer. ^{1}H-NMR data are reported as follows; chemical shift in parts per million (ppm) downfield or upfield from $CDCl_3$ (δ 7.26), D_2O (δ 4.79) or tetramethylsilane (δ 0.00) integration, multiplicity (br = broad, s = singlet, d = doublet, t = triplet, q = quartet, and m = multiplet) and coupling constants (Hz). ^{13}C chemical shifts are reported in ppm downfield or upfield from $CDCl_3$ (δ 77.0). Using D_2O as an NMR solvent, ^{13}C chemical shifts are reported in ppm downfield or upfield from 1,4-dioxane (δ 67.4) as an external reference. ^{31}P chemical shifts are reported in ppm downfield or upfield from 85% H_3PO_4 (δ 0.00) as an external reference. ESI-TOF Mass spectra were measured on a Waters LCT premier XE. Melting points were determined on a micro hot-stage (Yanako MP-S3) and were uncorrected. Optical rotations were measured on a JASCO P-2200 polarimeter. Silica gel TLC, column chromatography, and reverse-phase column chromatography were performed using Merck TLC 60F-254, Silica Gel 60 N (spherical, neutral, 63-210 μm or 40-50 μm) (Kanto Chemical Co., Inc.). Air- and/or moisture-sensitive reactions were carried out under an argon atmosphere using oven-dried glassware.

1,2-Anhydro donor **7** and **19** were prepared as described by Danishefsky *et al*[1].
meso-Diol **8** and triol **16** were prepared as descrived by Kishi *et al*[2].
meso-Diol **13** was prepared as descrived by O'Hagan *et al*[3].
meso-Diol **15** was prepared as descrived by Jessen *et al*[4].
1,2-Anhydro donor **17** was prepared as described by van Boom *et al*[5].
1,2-Anhydro donor **36** was prepared as described by Wei *et al*[6].
1,2-Anhydro donor **37** was prepared as described by Cao *et al*[7].
1,2-Anhydro donor **38** was prepared as described by Ito *et al*[8].
1,2-Anhydro donor **56** was prepared as described by our laboratory[9].

Synthesis of *meso*-Diol 14, 1,2-Anhydro Donor 18, and 1,2-Anhydro Donor 39

2-*O*-Diphenylphosphoryl-D-*myo*-inositol-1,3,5-orthoformate (14)

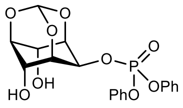

14

To a solution of *myo*-inositol 1,3,5-orthoformate (**16**) (179 mg, 0.944 mmol) in pyridine (3.1 mL) was added diphenyl phosphoryl chloride (235 μL, 1.13 mmol) at 0 °C under Ar atmosphere. After the reaction mixture was stirred for 24 h at room temperature, the reaction was quenched by addition of H_2O (3 mL). The aqueous layer was extracted with EtOAc (6 mL×3), and then the combined extracts were washed with brine (5 mL), dried over anhydrous Na_2SO_4, and concentrated in *vacuo*. Purification of the residue by silica gel column chromatography (1/1 PhMe/EtOAc) gave **14** (277 mg, 0.657 mmol, 70% yield).

Data for **14**: Colorless syrup; ^{1}H-NMR (500 MHz, $CDCl_3$) δ 7.37-7.33 (4H, m), 7.23-7.20 (6H, m), 5.49 (1H, d, *J*=1.0 Hz), 5.06-5.03 (3H, m), 4.42 (2H, m), 4.26 (2H, m), 4.16 (1H, m); ^{13}C-NMR (125 MHz, $CDCl_3$) δ 149.9 (d, $^{31P\text{-}13C}J$=7.8 Hz), 129.9, 126.0, 120.0 (d, $^{31P\text{-}13C}J$=4.8 Hz), 102.2, 72.5 (d, $^{31P\text{-}13C}J$=4.3 Hz), 69.3 (d, $^{31P\text{-}13C}J$=4.8 Hz), 68.1, 67.6; ^{31}P-NMR (202 MHz, $CDCl_3$) δ −11.3; HRMS (ESI-TOF) *m/z* 423.0862 (423.0845 calcd for $C_{19}H_{20}O_9P$ $[M+H]^+$).

Synthesis of 1,2-Anhydro-4-*O*-benzoyl-3,6-di-*O*-benzyl-D-glucose (**18**)

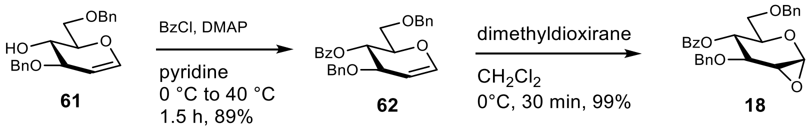

Supplementary Figure 1. Synthesis of 1,2-anhydro donor 18.

4-*O*-Benzoyl-3,6-di-*O*-benzyl-D-glucal (62)

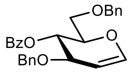

62

To a solution of 3,6-di-*O*-benzyl-D-glucal[10] (**61**) (2.38 g, 7.28 mmol) in pyridine (24.3 mL) was added benzoyl chloride (1.01 mL, 8.74 mmol) and 4-dimethylaminopyridine (178 mg, 1.46 mmol) at 0 °C under Ar atmosphere, and the reaction mixture was stirred for 30 min. After the reaction mixture was gradually warmed to 40 °C over a period of 1 h, the reaction was quenched by addition of H_2O

(20 mL). The resultant mixture was extracted with EtOAc (50 mL×3), and then the extracts were washed with brine (30 mL), dried over anhydrous Na_2SO_4, and concentrated in *vacuo*. The residue was subjected to silica gel column chromatography (4/1 *n*-hexane/EtOAc) to give **62** (2.77 g, 6.44 mmol, 89% yield).

Data for **62**: White solid; $[\alpha]^{22}_D$ −14.1° (*c* 1.83, $CHCl_3$); mp 68-69 °C; ^{1}H-NMR (500 MHz, $CDCl_3$) δ 8.01 (2H, d, *J*=8.0 Hz), 7.53 (1H, t, *J*=6.0 Hz), 7.40 (2H, d, *J*=8.0 Hz), 7.29-7.17 (10H, m), 6.51 (1H, dd, *J*=1.0 Hz, *J*=6.0 Hz), 5.56 (1H, dd, *J*=5.0 Hz, *J*=5.0 Hz), 4.92 (1H, dd, *J*=3.5 Hz, *J*=6.0 Hz), 4.65 and 4.61 (2H, ABq, *J*=12.0 Hz), 4.51 and 4.48 (2H, ABq, *J*=12.0 Hz), 4.43 (1H, m), 4.09 (1H, m), 3.80 (1H, dd, *J*=6.5 Hz, *J*=10.5 Hz), 3.72 (1H, dd, *J*=4.5 Hz, *J*=10.5 Hz); ^{13}C-NMR (125 MHz, $CDCl_3$) δ 165.2, 144.6, 137.9, 137.6, 133.1, 129.6, 129.5, 128.3, 128.2×2, 127.6×2, 127.5, 127.4, 99.2, 75.1, 73.2, 70.3, 69.8, 68.3, 67.9; HRMS (ESI-TOF) *m/z* 453.1656 (453.1678 calcd for $C_{27}H_{26}O_5Na$ $[M+Na]^+$).

1,2-Anhydro-4-*O*-benzoyl-3,6-di-*O*-benzyl-D-glucose (18)

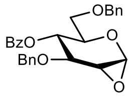

18

To a solution of **62** (44.0 mg, 0.102 mmol) in CH_2Cl_2 (1.0 mL) was added a solution of dimethyldioxirane (2.0 mL, 0.123 mmol, 60 mM in acetone) at 0 ºC under Ar atmosphere, and the reaction mixture was stirred for 30 min. Concentration of the reaction mixture in *vacuo* gave **18** (45.3 mg, 0.101 mmol, 99% yield).

Data for **18**: White solid; $[\alpha]^{22}_D$ +12.4° (*c* 1.87, $CHCl_3$); mp 55-57 °C; ^{1}H-NMR (500 MHz, $CDCl_3$) δ7.93-7.91 (2H, m), 7.59 (1H, t, *J*=7.5 Hz), 7.43 (2H, t, *J*=7.5 Hz), 7.22-7.12 (10H, m), 5.34 (1H, dd, *J*=8.5 Hz, *J*=10.5 Hz), 5.06 (1H, d, *J*=2.0 Hz), 4.75 and 4.58 (2H, ABq, *J*=12.0 Hz), 4.48 and 4.42 (2H, ABq, *J*=12.0 Hz), 4.01 (1H, dd, *J*=1.0 Hz, *J*=8.5 Hz), 3.97 (1H, dt, *J*=10.5 Hz, *J*=4.0 Hz), 3.55 (2H, d, *J*=4.0 Hz), 3.17 (1H, dd, *J*=1.0 Hz, *J*=2.0 Hz); ^{13}C-NMR (125 MHz, $CDCl_3$) δ165.1, 137.4, 136.9, 133.2, 129.8, 129.5, 128.3, 128.2, 127.9, 127.8, 127.7, 127.5, 77.3, 75.0, 73.5, 71.7, 68.6, 68.3×2, 52.4; HRMS (ESI-TOF) *m/z* 447.1805 (447.1808 calcd for $C_{27}H_{27}O_6$ $[M+H]^+$).

1,2-Anhydro-3,4-di-*O*-benzyl-L-fucose (39)

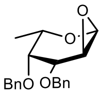

39

To a solution of 3,4-di-*O*-benzyl-L-fucal[11] (45.7 mg, 0.147 mmol) in CH_2Cl_2 (1.5 mL) was added a solution of dimethyldioxirane (2.7 mL, 0.177 mmol, 65 mM in acetone) at 0 ºC under Ar atmosphere, and the reaction mixture was stirred for 30 min. Concentration of the reaction mixture in *vacuo* gave **39** (48.0 mg, 0.147 mmol, quantitative yield).

Data for **39**: Colorless syrup; $[\alpha]^{25}_{D}$ +6.1° (*c* 1.0, $CHCl_3$); ^{1}H-NMR (500 MHz, $CDCl_3$) δ 7.41-7.25 (10H, m), 5.00 (1H, d, *J*=2.5 Hz), 4.98 and 4.70 (2H, ABq, *J*=11.5 Hz), 4.85 and 4.70 (2H, ABq, *J*=12.0 Hz), 3.77 (1H, d, *J*=3.5 Hz), 3.66 (1H, q, *J*=6.5 Hz), 3.45 (1H, m), 3.13 (1H, m), 1.17 (1H, d, *J*=6.5 Hz); ^{13}C-NMR (125 MHz, $CDCl_3$) δ 138.3, 137.6, 128.5, 128.3, 128.2, 127.9, 127.7, 127.5, 77.4, 76.8, 74.6, 71.8, 71.4, 67.4, 51.3, 17.0; HRMS (ESI-TOF) *m/z* 327.1608 (327.1596 calcd for $C_{20}H_{23}O_4$ $[M+H]^+$).

General Procedure for Optimization of Desymmetric 1,2-*cis*-Glycosylation of 7 and 8

To a solution of *meso*-diol **8** (0.025-0.05 mmol, 1.0 equiv.) and *p*-nitrophenylboronic acid (**3**) (5-10 μmol, 0.2 equiv.) in dry THF (0.2 M to **8**) was added a solution of 1,2-anhydro donor **7** (0.038-0.15 mmol, 1.5-3.0 equiv.) in dry THF (0.2 M to **7**) at the temperature indicated under Ar atmosphere. After the reaction mixture was stirred for 3 h, the reaction was quenched by addition of 0.05 M $NaBO_3$ aq. (11-22 μmol, 0.44 equiv.). To the resultant mixture was added sat. NH_4Cl aq. (2 mL). The aqueous layer was extracted with EtOAc (3 mL×3), and then the combined extracts were washed with brine (5 mL), dried over anhydrous Na_2SO_4, and concentrated in *vacuo*. Purification of the residue by silica gel column chromatography (2/1 *n*-hexane/EtOAc) gave **6-*O*-(3,4,6-tri-*O*-benzyl-α-D-glucopyranosyl)-2-*O*-*tert*-butyldimethylsilyl-D-*myo*-inositol-1,3,5-orthoformate (9)** and/or **4-*O*-(3,4,6-tri-*O*-benzyl-α-D-glucopyranosyl)-2-*O*-*tert*-butyldimethylsilyl-D-*myo*-inositol-1,3,5-orthoformate (10)**.

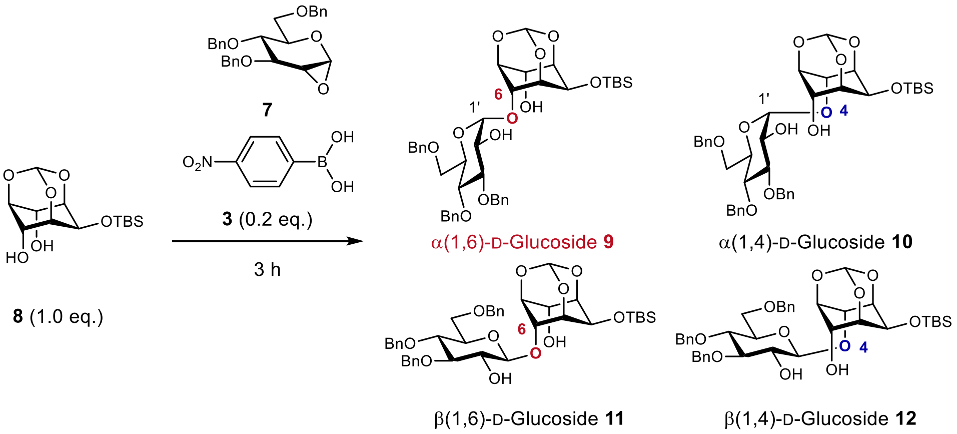

Data for **9**: Colorless foam; R_f 0.46 (2/1 *n*-hexane/EtOAc); $[\alpha]^{24}_{D}$ +57.5° (*c* 1.0, $CHCl_3$); ^{1}H-NMR (500 MHz, $CDCl_3$) δ 7.37-7.26 (13H, m), 7.18-7.16 (2H, m), 5.52 (1H, d, *J*=1.5 Hz), 5.01 (1H, d, *J*=4.0 Hz, H-1'), 4.93 and 4.66 (2H, ABq, *J*=11.5 Hz), 4.78 and 4.53 (2H, ABq, *J*=10.5 Hz), 4.66 (1H, m), 4.64 and 4.52 (2H, ABq, *J*=12.5 Hz), 4.40 (1H, m), 4.27 (1H, m), 4.23 (2H, m), 4.11 (1H, m), 3.82 (1H, m), 3.75 (1H, dd, *J*=3.5 Hz, *J*=11.0 Hz), 3.71-3.63 (4H, m), 3.57 (1H, d, *J*=10.0 Hz), 2.12 (1H, d, *J*=3.0 Hz), 0.92 (9H, s), 0.11 (6H, s); ^{13}C-NMR (125 MHz, $CDCl_3$) δ 138.1, 137.6, 137.4, 128.7, 128.5, 128.1, 128.0×2, 127.9×3, 102.7, 94.9, 82.1, 77.4, 75.5, 75.2, 75.0, 73.7, 71.7, 71.5, 71.0, 70.8, 68.8, 68.3, 68.0, 60.3, 25.9, 18.3, −4.7, −4.8; HRMS (ESI-TOF) 737.3344 (737.3357 calcd for $C_{40}H_{53}O_{11}Si$ $[M+H]^+$).

Data for **10**: White solid; R_f 0.34 (1/1 *n*-hexane/EtOAc); $[\alpha]^{23}_D$ +88.4° (*c* 1.0, $CHCl_3$); mp 121-123 °C; ^{1}H-NMR (500 MHz, $CDCl_3$) δ 7.35-7.26 (13H, m), 7.19-7.18 (2H, m), 5.52 (1H, d, *J*=1.0 Hz), 5.08 (1H, d, *J*=4.0 Hz, H-1'), 4.87 and 4.67 (2H, ABq, *J*=11.5 Hz), 4.81 and 4.55 (2H, ABq, *J*=11.5 Hz), 4.63 (1H, m), 4.61 and 4.50 (2H, ABq, *J*=12.0 Hz), 4.50 (1H, m), 4.31 (1H, m), 4.26 (1H, m), 4.11 (1H, m), 4.09 (1H, m), 3.92 (1H, d, *J*=8.5 Hz), 3.75-3.60 (6H, m), 2.75 (1H, d, *J*=4.0 Hz), 0.96 (9H, s), 0.17 (6H, s); ^{13}C-NMR (125 MHz, $CDCl_3$) δ 138.1, 138.0, 137.6, 128.7, 128.5, 128.4, 128.1, 127.9, 127.8, 127.7, 127.5, 102.7, 96.7, 82.2, 75.4, 75.0, 74.6, 73.6, 73.3, 71.9, 71.4, 71.2, 68.2, 67.9, 67.0, 61.0, 26.1, 18.6, −4.4, −4.6; HRMS (ESI-TOF) 737.3391 (737.3357 calcd for $C_{40}H_{53}O_{11}Si$ $[M+H]^+$).

Supplementary Table 1. Optimization of reaction conditions.

Entry	Solv.	Temp.	Donor **7**	Yields (%)				Recovery of **8** (%)
				α(1,6): **9**	α(1,4): **10**	β(1,6): **11**	β(1,4): **12**	
1	DMF	-40 °C	3.0 eq.	71	20	0	0	5
2	CH_2Cl_2	-40 °C	3.0 eq.	58	7	0	0	20
3	MeCN	-40 °C	3.0 eq.	45	0	0	0	47
4	THF	-40 °C	3.0 eq.	90	0	0	0	trace
5	THF	-40 °C	3.0 eq.	90	0	0	0	trace
6	THF	-20 °C	3.0 eq.	95	0	0	0	0
7	THF	-20 °C	2.0 eq.	94	0	0	0	0
8	THF	-20 °C	1.5 eq.	96	0	0	0	0

Desymmetric 1,2-*cis*-Glycosylation Reactions of Several 1,2-Anhydro Donors and *meso*-Diols

2-*O*-Benzoyl-6-*O*-(3,4,6-tri-*O*-benzyl-α-D-glucopyranosyl)-D-*myo*-inositol-1,3,5-orthoformate (20)

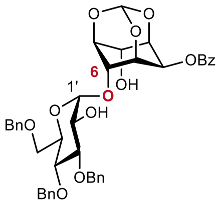

α(1,6)-D-Glucoside **20**

To a solution of **13** (8.1 mg, 27.5 μmol) and *p*-nitrophenylboronic acid (**3**) (0.92 mg, 5.51 μmol) in dry THF (206 μL) was added a solution of **7** (17.9 mg, 41.3 μmol) in dry THF (206 μL) at −40 ºC under Ar atmosphere. After the reaction mixture was stirred for 12 h, the reaction was quenched by addition of 0.05 M $NaBO_3$ aq. (0.242 mL, 12.1 μmol). To the resultant mixture was added sat. NH_4Cl aq. (2 mL). The aqueous layer was extracted with EtOAc (3 mL×3), and then the combined extracts were washed with brine (5 mL), dried over anhydrous Na_2SO_4, and concentrated in *vacuo*. Purification of the residue by silica gel column chromatography (2/1 *n*-hexane/EtOAc) gave **20** (19.9 mg, 27.4 μmol, 99% yield).

Data for **20**: Colorless syrup; R_f 0.53 (3/1 PhMe/EtOAc); $[\alpha]^{22}_D$ +70.4° (*c* 1.0, $CHCl_3$); ^{1}H-NMR (500 MHz, $CDCl_3$) δ 8.13 (2H, d, *J*=8.0 Hz), 7.59 (1H, t, *J*=8.0 Hz), 7.46 (2H, t, *J*=8.0 Hz), 7.34-7.26 (13H, m), 7.17-7.16 (2H, m), 5.58 (1H, s), 5.51 (1H, br-s), 5.27 (1H, d, *J*=3.5 Hz, H-1'), 4.89 and 4.79 (2H, ABq, *J*=11.0 Hz), 4.81 and 4.53 (2H, ABq, *J*=11.0 Hz), 4.73 (1H, m), 4.69 (1H, m), 4.63 and 4.52 (2H, ABq, *J*=12.0 Hz), 4.51 (1H, m), 4.43 (1H, m), 4.35 (1H, m), 3.93 (1H, *J*=10.5 Hz), 3.87-3.84 (2H, m), 3.77-3.66 (4H, m), 2.59 (1H, d, *J*=3.5 Hz); ^{13}C-NMR (125 MHz, $CDCl_3$) δ 166.6, 138.2, 137.8, 137.6, 133.5, 130.0, 129.3, 128.6, 128.5, 127.9×3, 102.7, 95.2, 82.2, 77.2, 75.6, 75.1, 73.7, 72.6, 71.8, 71.6, 70.3, 68.9, 68.5, 68.2, 68.1, 63.2; HRMS (ESI-TOF) 749.2543 (749.2574 calcd for $C_{41}H_{42}O_{12}Na$ $[M+Na]^+$).

6-*O*-(3,4,6-Tri-*O*-benzyl-α-D-glucopyranosyl)-2-*O*-diphenylphosphoryl-D-*myo*-inositol-1,3,5-orthoformate (21)

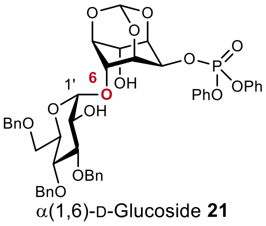

α(1,6)-D-Glucoside **21**

To a solution of **14** (12.1 mg, 28.7 μmol) and *p*-nitrophenylboronic acid (**3**) (0.96 mg, 5.73 μmol) in dry THF (215 μL) was added a solution of **7** (18.6 mg, 43.0 μmol) in dry THF (215 μL) at −20 ºC under Ar atmosphere. After the reaction mixture was stirred for 6 h at −20 ºC, the reaction was quenched by addition of 0.05 M $NaBO_3$ aq. (0.252 mL, 12.6 μmol). To the resultant mixture was added sat. NH_4Cl aq. (2 mL). The aqueous layer was extracted with EtOAc (3 mL×3), and then the combined extracts were washed with brine (5 mL), dried over anhydrous Na_2SO_4, and concentrated in *vacuo*. Purification of the residue by preparative TLC (2/1 PhMe/acetone) gave **21** (20.2 mg, 23.6 μmol, 82% yield).

Data for **21**: Colorless syrup; R_f 0.72 (1/1 PhMe/EtOAc); $[\alpha]^{21}_D$ +63.0° (*c* 1.25, $CHCl_3$); ^{1}H-NMR (500 MHz, $CDCl_3$) δ 7.37-7.19 (23H, m), 7.15-7.13 (2H, m), 5.54 (1H, s), 5.19 (1H, br-d, *J*=8.0 Hz), 4.99 (1H, d, *J*=3.5 Hz, H-1'), 4.83 and 4.81 (2H, ABq, *J*=11.5 Hz), 4.80 and 4.48 (2H, ABq, *J*=10.5 Hz), 4.68 (1H, m), 4.60 and 4.50 (2H, ABq, *J*=12.0 Hz), 4.54 (1H, m), 4.44 (1H, m), 4.28 (2H, m), 3.82-3.59 (7H, m), 3.05 (1H, d, *J*=3.5 Hz); ^{13}C-NMR (125 MHz, $CDCl_3$) δ 150.1×2 (d, $^{31P\text{-}13C}J$=7.8 Hz), 138.3, 137.8, 137.5, 129.9×2, 128.5, 128.4×2, 127.9×2, 127.8×2, 125.8, 125.7, 120.3 (d, $^{31P\text{-}13C}J$=4.8 Hz), 120.1 (d, $^{31P\text{-}13C}J$=4.8 Hz), 102.6, 95.9, 81.9, 76.9, 75.4, 75.1, 73.6, 72.9 (d, $^{31P\text{-}13C}J$=6.6 Hz), 71.6, 70.7, 69.4, 68.5, 68.2, 68.0, 67.8 (d, $^{31P\text{-}13C}J$=4.8 Hz); ^{31}P-NMR (202 MHz, $CDCl_3$) δ −10.1; HRMS (ESI-TOF) 877.2559 (877.2601 calcd for $C_{46}H_{47}O_{14}NaP$ $[M+Na]^+$).

6-*O*-(3,4,6-Tri-*O*-benzyl-α-D-glucopyranosyl)-2-*O*-*tert*-butyldimethylsilyl-D-*myo*-inositol-1,3,5-orthobenzoate (22)

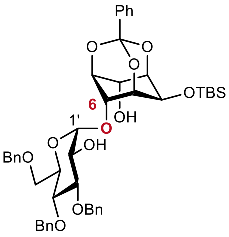

α(1,6)-D-Glucoside **22**

To a solution of **15** (11.5 mg, 30.2 μmol) and *p*-nitrophenylboronic acid (**3**) (1.01 mg, 6.04 μmol) in dry THF (227 μL) was added a solution of **7** (19.6 mg, 45.3 μmol) in dry THF (227 μL) at −15 °C under Ar atmosphere. After the reaction mixture was stirred for 3 h, the reaction was quenched by addition of 0.05 M $NaBO_3$ aq. (0.266 mL, 13.3 μmol). To the resultant mixture was added sat. NH_4Cl aq. (2 mL). The aqueous layer was extracted with EtOAc (3 mL×3), and then the combined extracts were washed with brine (5 mL), dried over anhydrous Na_2SO_4, and concentrated in *vacuo*. Purification of the residue by silica gel column chromatography (3/1 *n*-hexane/EtOAc) gave **22** (22.0 mg, 27.1 μmol, 90% yield).

Data for **22**: Colorless syrup; R_f 0.58 (2/1 *n*-hexane/EtOAc); $[\alpha]^{26}_D$ +96.3° (*c* 1.0, $CHCl_3$); ^{1}H-NMR (500 MHz, $CDCl_3$) δ 7.65-7.63 (2H, m), 7.38-7.27 (16H, m), 7.18-7.16 (2H, m), 5.06 (1H, d, *J*=2.5 Hz, H-1'), 4.95 and 4.68 (2H, ABq, *J*=11.5 Hz), 4.79 and 4.54 (2H, ABq, *J*=10.5 Hz), 4.78 (1H, m), 4.64 and 4.52 (2H, ABq, *J*=11.5 Hz), 4.52 (1H, m), 4.44 (1H, m), 4.38 (1H, m), 4.28 (1H, m), 4.26 (1H, m), 3.85 (1H, m), 3.77 (1H, dd, *J*=3.0 Hz, *J*=10.5 Hz), 3.71-3.67 (4H, m), 3.59 (1H, d, *J*=10.0 Hz), 2.16 (1H, br-s), 0.94 (9H, s), 0.11 (6H, s); ^{13}C-NMR (125 MHz, $CDCl_3$) δ 138.1, 137.6, 137.5, 137.2, 129.4, 128.7, 128.5×2, 128.1, 128.0×2, 127.9, 125.4, 107.4, 95.0, 82.1, 77.4, 76.5, 75.5, 75.0, 73.7, 72.8, 71.7, 71.0, 70.7, 69.7, 68.3, 68.0, 59.3, 25.8, 18.2, −4.7; HRMS (ESI-TOF) *m/z* 813.3701 (813.3670 calcd for $C_{46}H_{57}O_{11}Si$ $[M+H]^+$).

6-*O*-(3,4,6-Tri-*O*-benzyl-α-D-glucopyranosyl)-D-*myo*-inositol-1,3,5-orthoformate (23)

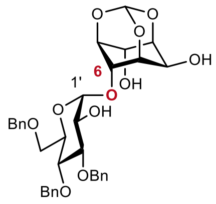

α(1,6)-D-Glucoside **23**

To a solution of **16** (4.4 mg, 23.1 μmol) and *p*-nitrophenylboronic acid (**3**) (0.77 mg, 4.61 μmol) in dry THF (174 μL) was added a solution of **7** (15.0 mg, 34.7 μmol) in dry THF (174 μL) at room temperature under Ar atmosphere. After the reaction mixture was stirred for 12 h, the reaction was quenched by addition of 0.05 M $NaBO_3$ aq. (0.204 mL, 10.2 μmol). To the resultant mixture was added sat. NH_4Cl aq. (2 mL). The aqueous layer was extracted with EtOAc (3 mL×3), and then the combined extracts were washed with brine (5 mL), dried over anhydrous Na_2SO_4, and concentrated in *vacuo*. Purification of the residue by silica gel column chromatography (1/2 PhMe/EtOAc) gave **23** (10.8 mg, 17.3 μmol, 75% yield).

Data for **23**: Colorless syrup; R_f 0.43 (1/2 PhMe/EtOAc); $[\alpha]^{26}_{D}$ +92.3° (*c* 1.08, $CHCl_3$); ^{1}H-NMR (500 MHz, $CDCl_3$) δ 7.37-7.26 (13H, m), 7.17-7.15 (2H, m), 5.47 (1H, d, *J*=1.0 Hz), 5.03 (1H, d, *J*=3.5 Hz, H-1'), 4.90 and 4.69 (2H, ABq, *J*=11.5 Hz), 4.78 and 4.52 (2H, ABq, *J*=10.5 Hz), 4.68 (1H, m), 4.62 and 4.51 (2H, ABq, *J*=12.0 Hz), 4.43 (1H, m), 4.36 (1H, m), 4.27 (1H, m), 4.18 (1H, m), 4.11 (1H, m), 3.83 (1H, m), 3.75-3.62 (6H, m), 3.17 (1H, d, *J*=11.5 Hz), 2.36 (1H, d, *J*=3.0 Hz); ^{13}C-NMR (125 MHz, $CDCl_3$) δ 138.0, 137.6, 137.5, 128.7, 128.5, 128.1, 128.0, 127.9×3, 102.8, 95.4, 82.1, 75.5, 75.0, 74.7, 73.7, 71.8, 71.3, 71.0, 70.6, 68.5, 68.1, 67.7, 60.2; HRMS (ESI-TOF) *m/z* 645.2328 (645.2312 calcd for $C_{34}H_{38}O_{11}Na$ $[M+Na]^+$).

6-*O*-(3,4-Di-*O*-benzyl-6-*O*-*tert*-butyldiphenylsilyl-α-D-glucopyranosyl)-2-*O*-*tert*-butyldimethylsilyl-D-*myo*-inositol-1,3,5-orthoformate (24)

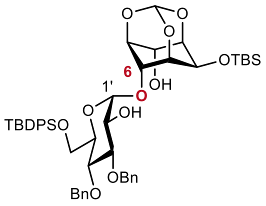

α(1,6)-D-Glucoside **24**

To a solution of **8** (10.1 mg, 33.2 μmol) and *p*-nitrophenylboronic acid (**3**) (1.11 mg, 6.64 μmol) in dry THF (249 μL) was added a solution of **17** (28.9 mg, 49.8 μmol) in dry THF (249 μL) at −20 ºC under Ar atmosphere. After the reaction mixture was stirred for 12 h at −20 ºC, the reaction was quenched by addition of 0.05 M $NaBO_3$ aq. (0.292 mL, 14.6 μmol). To the resultant mixture was added sat. NH_4Cl aq. (2 mL). The aqueous layer was extracted with EtOAc (3 mL×3), and then the combined extracts were washed with brine (5 mL), dried over anhydrous Na_2SO_4, and concentrated in *vacuo*. Purification of the residue by silica gel column chromatography (10/1 PhMe/EtOAc) gave **24** (29.1 mg, 32.9 μmol, 99% yield).

Data for **24**: Colorless syrup; R_f 0.37 (3/1 *n*-hexane/EtOAc); $[\alpha]^{20}_{D}$ +65.5° (*c* 2.81, $CHCl_3$); ^{1}H-NMR (500 MHz, $CDCl_3$) δ 7.69-7.66 (4H, m), 7.46-7.41 (2H, m), 7.39-7.27 (12H, m), 7.18-7.15 (2H, m), 5.52 (1H, d, *J*=1.0 Hz), 4.96 and 4.67 (2H, ABq, *J*=12.0 Hz), 4.96 (1H, br-s, H-1'), 4.84 and 4.67 (2H, ABq, *J*=10.5 Hz), 4.63 (1H, m), 4.41 (1H, m), 4.25 (1H, m), 4.23 (1H, m), 4.18 (1H, m), 4.12 (1H, m), 3.92 (2H, s), 3.72-3.65 (4H, m), 3.50 (1H, d, *J*=10.5 Hz), 2.10 (1H, br-s) 1.08 (9H, s), 0.94 (9H, s), 0.14 (6H, s); ^{13}C-NMR (125 MHz, $CDCl_3$) δ 138.0, 137.6, 135.8, 135.6, 133.2, 132.9, 129.8, 128.8, 128.5, 128.2, 128.0, 127.9×2, 127.7, 127.6, 102.7, 94.8, 82.0, 77.4, 75.6, 75.2, 75.1, 73.1, 71.5, 71.0, 70.3, 68.5, 68.4, 62.3, 60.3, 26.8, 25.9, 19.3, 18.3, −4.7×2; HRMS (ESI-TOF) *m/z* 885.4066 (885.4065 calcd for $C_{49}H_{65}O_{11}Si_2$ $[M+H]^+$).

6-*O*-(4-*O*-Benzoyl-3,6-di-*O*-benzyl-α-D-glucopyranosyl)-2-*O*-*tert*-butyldimethylsilyl-D-*myo*-inositol-1,3,5-orthoformate (25)

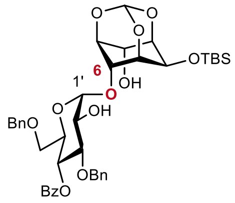

α(1,6)-D-Glucoside **25**

To a solution of **8** (11.1 mg, 36.5 μmol) and *p*-nitrophenylboronic acid (**3**) (1.22 mg, 7.29 μmol) in dry THF (274 μL) was added a solution of **18** (24.4 mg, 54.7 μmol) in dry THF (274 μL) at −20 ºC under Ar atmosphere. After the reaction mixture was stirred for 6 h at −20 ºC, the reaction was quenched by addition of 0.05 M $NaBO_3$ aq. (0.320 mL, 16.0 μmol). To the resultant mixture was added sat. NH_4Cl aq. (2 mL). The aqueous layer was extracted with EtOAc (3 mL×3), and then the combined extracts were washed with brine (5 mL), dried over anhydrous Na_2SO_4, and concentrated in *vacuo*. Purification of the residue by preparative TLC (6/1 PhMe/acetone) gave **25** (26.3 mg, 35.0 μmol, 96% yield).

Data for **25**: Colorless syrup; R_f 0.46 (6/1 PhMe/acetone); $[\alpha]^{21}_D$ +53.1° (*c* 2.63, $CHCl_3$); ^{1}H-NMR (500 MHz, $CDCl_3$) δ 8.01 (2H, d, *J*=8.5 Hz), 7.61 (1H, t, *J*=7.5 Hz), 7.46 (2H, t, *J*=8.5 Hz), 7.26-7.15 (10H, m), 5.55 (1H, d, *J*=1.5 Hz), 5.35 (1H, dd, *J*=9.5 Hz, *J*=10.5 Hz), 5.08 (1H, d, *J*=4.0 Hz, H-1'), 4.71 (1H, m), 4.69 and 4.55 (2H, ABq, *J*=11.5 Hz), 4.50 and 4.46 (2H, ABq, *J*=12.0 Hz), 4.40 (1H, m), 4.34 (1H, m), 4.29 (1H, m), 4.28 (1H, m), 4.14 (1H, m), 4.10 (1H, m), 3.84 (1H, m), 3.79 (1H, dd, *J*=9.0 Hz, *J*=9.5 Hz), 3.71 (1H, d, *J*=9.5 Hz), 3.60 (1H, dd, *J*=3.0 Hz, *J*=11.0 Hz), 3.55 (1H, dd, *J*=5.0 Hz, *J*=11.0 Hz), 2.31 (1H, br-s), 0.94 (9H, s), 0.14 (6H, s); ^{13}C-NMR (125 MHz, $CDCl_3$) δ 165.2, 137.4, 137.1, 133.5, 129.8, 129.2, 128.6, 128.4, 128.1, 128.0, 127.9, 127.8, 102.7, 95.5, 79.8, 75.0×2, 73.8, 71.9, 71.6, 70.9, 70.6, 70.4, 68.9, 68.6, 68.3, 60.3, 25.9, 18.4, −4.7×2; HRMS (ESI-TOF) *m/z* 751.3120 (751.3150 calcd for $C_{40}H_{51}O_{12}Si$ $[M+H]^+$).

2-*O*-*tert*-Butyldimethylsilyl-6-*O*-(3,4,6-tri-*O*-*tert*-butyldimethylsilyl-α-D-glucopyranosyl)-D-*myo*-inositol-1,3,5-orthoformate (26)

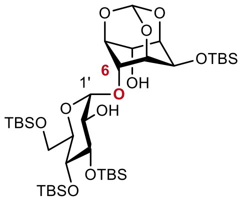

α(1,6)-D-Glucoside **26**

To a solution of **8** (8.1 mg, 26.6 μmol) and *p*-nitrophenylboronic acid (**3**) (0.89 mg, 5.32 μmol) in dry THF (200 μL) was added a solution of **19** (20.2 mg, 39.9 μmol) in dry THF (200 μL) at room temperature under Ar atmosphere. After the reaction mixture was stirred for 20 h, the reaction was quenched by addition of 0.05 M $NaBO_3$ aq. (0.234 mL, 11.7 μmol). To the resultant mixture was added sat. NH_4Cl aq. (2 mL). The aqueous layer was extracted with EtOAc (3 mL×3), and then the combined extracts were washed with brine (5 mL), dried over anhydrous Na_2SO_4, and concentrated in *vacuo*. Purification of the residue by silica gel column chromatography (20/1 PhMe/EtOAc) gave **26** (19.0 mg, 23.5 μmol, 88% yield).

Data for **26**: White solid; R_f 0.20 (20/1 PhMe/EtOAc); $[\alpha]^{21}_D$ +19.5° (*c* 1.85, $CHCl_3$); mp 166-167 °C; ^{1}H-NMR (500 MHz, $CDCl_3$) δ 5.52 (1H, d, *J*=1.5 Hz), 4.94 (1H, d, *J*=1.0 Hz, H-1'), 4.58 (1H, m), 4.53 (1H, m), 4.48 (1H, m), 4.29 (1H, m), 4.14 (2H, m), 4.05 (1H, d, *J*=8.5 Hz), 3.98 (1H, m), 3.94 (1H, dd, *J*=3.0 Hz, *J*=4.5 Hz), 3.90 (1H, dd, *J*=7.0 Hz, *J*=10.0 Hz), 3.84 (1H, dd, *J*=7.0 Hz, *J*=10.0 Hz), 3.69 (1H, m), 3.46 (1H, dd, *J*=4.5 Hz, *J*=10.0 Hz), 3.38 (1H, d, *J*=10.0 Hz), 0.94 (9H, s), 0.91 (18H, s), 0.90 (9H, s), 0.15 (6H, s), 0.12 (3H, s), 0.11 (9H, s), 0.09 (3H, s), 0.07 (3H, s); ^{13}C-NMR (125 MHz, $CDCl_3$) δ 102.6, 95.0, 80.3, 75.1, 74.1, 72.8, 72.2, 70.6, 69.1, 69.0, 68.2, 61.1, 61.0, 26.0, 25.8×2, 25.7, 18.4, 18.2, 18.0, 17.9, −4.5, −4.6×2, −4.7, −4.8, −5.0, −5.4; HRMS (ESI-TOF) *m/z* 831.4348 (831.4362 calcd for $C_{37}H_{76}O_{11}NaSi_4$ $[M+Na]^+$).

6-*O*-(3,4,6-Tri-*O*-benzyl-α-D-galactopyranosyl)-2-*O*-*tert*-butyldimethylsilyl-D-*myo*-inositol-1,3,5-orthoformate (40) and 4-*O*-(3,4,6-tri-*O*-benzyl-α-D-galactopyranosyl)-2-*O*-*tert*-butyldimethylsilyl-D-*myo*-inositol-1,3,5-orthoformate (41)

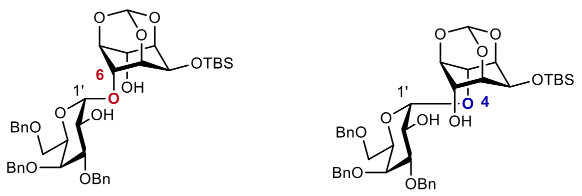

α(1,6)-D-Galactoside **40** α(1,4)-D-Galactoside **41**

To a solution of **8** (7.0 mg, 23.0 μmol) and *p*-nitrophenylboronic acid (**3**) (0.77 mg, 4.61 μmol) in dry THF (173 μL) was added a solution of **36** (14.9 mg, 34.5 μmol) in dry THF (173 μL) at 10 ºC under Ar atmosphere. After the reaction mixture was stirred for 24 h, the reaction was quenched by addition of 0.05 M $NaBO_3$ aq. (0.203 mL, 10.1 μmol). To the resultant mixture was added sat. NH_4Cl aq. (2 mL). The aqueous layer was extracted with EtOAc (3 mL×3), and then the combined extracts were washed with brine (5 mL), dried over anhydrous Na_2SO_4, and concentrated in *vacuo*. Purification of the residue by silica gel column chromatography (5/1 PhMe/acetone) gave **40** (15.8 mg, 21.4 μmol, 93% yield) and **41** (0.59 mg, 0.795 μmol, 3% yield).

Data for **40**: Colorless syrup; R_f 0.57 (3/1 PhMe/EtOAc); $[\alpha]^{24}_D$ +78.4° (*c* 1.0, $CHCl_3$); ^{1}H-NMR (500 MHz, $CDCl_3$) δ 7.38-7.26 (15H, m), 5.52 (1H, s), 5.06 (1H, d, *J*=4.5 Hz, H-1'), 4.84 and 4.55 (2H, ABq, *J*=11.0 Hz), 4.73 and 4.53 (2H, ABq, *J*=11.5 Hz), 4.64 (1H, m), 4.51 and 4.45 (2H, ABq, *J*=11.5 Hz), 4.36 (1H, m), 4.27-4.25 (3H, m), 4.21 (1H, m), 4.10 (1H, m), 4.00-3.98 (2H, m), 3.79 (1H, d, *J*=9.5 Hz), 3.60-3.57 (3H, m), 2.30 (1H, d, *J*=2.5 Hz), 0.92 (9H, s), 0.12 (6H, s); ^{13}C-NMR (125 MHz, $CDCl_3$) δ 138.1, 137.4×2, 128.7, 128.6, 128.4, 128.1×3, 128.0, 127.8, 127.7, 102.7, 95.6, 79.2, 75.2, 74.7, 73.8, 72.9, 72.1, 71.7, 71.4, 70.7, 69.0, 68.8, 68.3, 67.6, 60.3, 25.9, 18.4, −4.7; HRMS (ESI-TOF) *m/z* 737.3384 (737.3357 calcd for $C_{40}H_{53}O_{11}Si$ $[M+H]^+$).

Data for **41**: Colorless syrup; R_f 0.15 (3/1 PhMe/EtOAc); $[\alpha]^{24}_D$ +69.2° (*c* 0.24, $CHCl_3$); ^{1}H-NMR (500 MHz, $CDCl_3$) δ 7.37-7.26 (15H, m), 5.53 (1H, s), 5.13 (1H, d, *J*=4.0 Hz, H-1'), 4.84 and 4.56 (2H, ABq, *J*=11.0 Hz), 4.70 and 4.49 (2H, ABq, *J*=12.0 Hz), 4.63 (1H, m), 4.52 and 4.45 (2H, ABq, *J*=11.5 Hz), 4.33 (1H, m), 4.22 (1H, m), 4.21 (1H, m), 4.12-4.09 (3H, m), 4.03 (1H, br-s), 3.88 (1H, m), 3.63 (1H, dd, *J*=8.0 Hz, *J*=9.0 Hz), 3.59-3.54 (2H, m), 2.73 (1H, br-s), 0.94 (9H, s), 0.13 (6H, s); ^{13}C-NMR (125 MHz, $CDCl_3$) δ 138.1, 137.6, 137.3, 128.7, 128.5, 128.4, 128.2, 128.0, 127.9×2, 127.8, 127.7, 102.7, 96.8, 78.8, 75.0, 74.8, 73.6, 73.3, 72.9, 72.0, 71.1, 70.7, 68.2×2, 67.8, 66.9, 61.1, 26.0, 18.6, −4.5, −4.7; HRMS (ESI-TOF) *m/z* 775.2893 (775.2916 calcd for $C_{40}H_{52}O_{11}SiK$ $[M+K]^+$).

6-*O*-(3,4-Di-*O*-benzyl-β-L-rhamnopyranosyl)-2-*O*-*tert*-butyldimethylsilyl-D-*myo*-inositol-1,3,5-orthoformate (42)

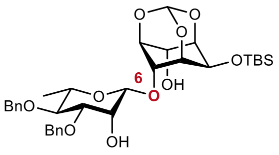

β(1,6)-L-Rhamnoside **42**

To a solution of **8** (8.7 mg, 28.6 μmol) and *p*-nitrophenylboronic acid (**3**) (0.95 mg, 5.72 μmol) in dry THF (215 μL) was added a solution of **37** (14.0 mg, 42.9 μmol) in dry THF (215 μL) at 0 °C under Ar atmosphere. After the reaction mixture was stirred for 3 h, the reaction was quenched by addition of 0.05 M $NaBO_3$ aq. (0.252 mL, 12.6 μmol). To the resultant mixture was added sat. NH_4Cl aq. (2 mL). The aqueous layer was extracted with EtOAc (3 mL×3), and then the combined extracts were washed with brine (5 mL), dried over anhydrous Na_2SO_4, and concentrated in *vacuo*. Purification of the residue by silica gel column chromatography (3/1 PhMe/EtOAc) gave **42** (17.9 mg, 28.4 μmol, 99% yield).

Data for **42**: Colorless syrup; R_f 0.34 (3/1 PhMe/EtOAc); $[\alpha]^{23}_{D}$ +18.1° (*c* 1.0, $CHCl_3$); ^{1}H-NMR (500 MHz, $CDCl_3$) δ 7.36-7.28 (15H, m), 5.49 (1H, d, *J*=1.5 Hz), 4.87 and 4.63 (2H, ABq, *J*=11.0 Hz), 4.71 and 4.68 (2H, ABq, *J*=11.5 Hz), 4.60 (1H, d, *J*=0.5 Hz), 4.56 (1H, m), 4.48 (2H, m), 4.25 (1H, m), 4.13-4.09 (2H, m), 4.00 (1H, br-s), 3.89 (1H, m), 3.54 (1H, dd, *J*=3.5 Hz, *J*=9.0 Hz), 3.47 (1H, dd, *J*=9.0 Hz, *J*=9.0 Hz), 3.37 (1H, m), 2.43 (1H, d, *J*=1.5 Hz), 1.36 (1H, d, *J*=6.0 Hz), 0.95 (9H, s), 0.15 (6H, s); ^{13}C-NMR (125 MHz, $CDCl_3$) δ 137.9, 137.4, 128.6, 128.5, 128.2, 128.1, 127.9×2, 102.5, 99.5 ($^1J_{CH}$=157 Hz), 81.1, 79.1, 75.5, 74.9, 74.2, 72.9, 71.9×2, 69.2, 68.2, 68.1, 61.0, 25.9, 18.4, 17.8, −4.5, −4.7; HRMS (ESI-TOF) *m/z* 631.2917 (631.2939 calcd for $C_{33}H_{47}O_{10}Si$ $[M+H]^+$).

4-*O*-(3,4,6-Tri-*O*-benzyl-β-D-mannopyranosyl)-2-*O*-*tert*-butyldimethylsilyl-D-*myo*-inositol-1,3,5-orthoformate (43)

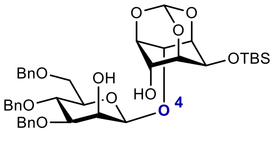

β(1,4)-D-Mannoside **43**

To a solution of **8** (8.8 mg, 28.9 μmol) and *p*-nitrophenylboronic acid (**3**) (0.97 mg, 5.78 μmol) in dry THF (217 μL) was added a solution of **38** (18.8 mg, 43.4 μmol) in dry THF (217 μL) at −20

°C under Ar atmosphere. After the reaction mixture was stirred for 3 h, the reaction was quenched by addition of 0.05 M $NaBO_3$ aq. (0.254 mL, 12.7 μmol). To the resultant mixture was added sat. NH_4Cl aq. (2 mL). The aqueous layer was extracted with EtOAc (3 mL×3), and then the combined extracts were washed with brine (5 mL), dried over anhydrous Na_2SO_4, and concentrated in *vacuo*. Purification of the residue by silica gel column chromatography (2/1 *n*-hexane/EtOAc) gave **43** (21.0 mg, 28.5 μmol, 99% yield).

Data for **43**: White solid; R_f 0.34 (2/1 *n*-hexane/EtOAc); $[\alpha]^{23}_{D}$ −8.0° (*c* 0.78, $CHCl_3$); mp 170-171 °C; ^{1}H-NMR (500 MHz, $CDCl_3$) δ 7.37-7.26 (13H, m), 7.22-7.20 (2H, m), 5.51 (1H, d, *J*=1.0 Hz), 4.82 and 4.54 (2H, ABq, *J*=11.0 Hz), 4.71 and 4.67 (2H, ABq, *J*=11.5 Hz), 4.64 (1H, d, *J*=0.5 Hz), 4.59 and 4.54 (2H, ABq, *J*=12.5 Hz), 4.59 (1H, m), 4.52 (1H, m), 4.48 (1H, m), 4.26 (1H, m), 4.13 (1H, m), 4.10 (1H, m), 4.02 (1H, m), 3.86 (1H, br-s), 3.81 (1H, dd, *J*=9.0 Hz, *J*=9.0 Hz), 3.71 (1H, dd, *J*=2.5 Hz, *J*=10.5 Hz), 3.67 (1H, dd, *J*=5.5 Hz, *J*=10.5 Hz), 3.58 (1H, dd, *J*=3.5 Hz, *J*=9.0 Hz), 3.47 (1H, m), 2.43 (1H, br-s), 0.94 (9H, s), 0.15 (6H, s); ^{13}C-NMR (125 MHz, $CDCl_3$) δ 137.9, 137.8, 137.3, 128.6, 128.5, 128.4, 128.1×2, 127.9, 127.8, 102.5, 99.6 ($^1J_{CH}$=158 Hz), 81.0, 75.2, 75.1, 74.8, 74.4, 73.8, 73.4, 72.8, 71.9, 69.3, 68.7, 68.2, 67.8, 61.0, 26.0, 18.4, −4.6, −4.7; HRMS (ESI-TOF) *m/z* 737.3342 (737.3357 calcd for $C_{40}H_{53}O_{11}Si$ $[M+H]^+$).

4-*O*-(3,4-Di-*O*-benzyl-α-L-fucopyranosyl)-2-*O*-*tert*-butyldimethylsilyl-D-*myo*-inositol-1,3,5-orthoformate (44)

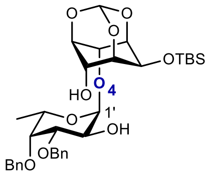

α(1,4)-L-Fucoside **44**

To a solution of **8** (9.8 mg, 32.2 μmol) and *p*-nitrophenylboronic acid (**3**) (1.07 mg, 6.44 μmol) in dry THF (242 μL) was added a solution of **39** (15.8 mg, 48.3 μmol) in dry THF (242 μL) at −20 °C under Ar atmosphere. After the reaction mixture was stirred for 24 h, the reaction was quenched by addition of 0.05 M $NaBO_3$ aq. (0.283 mL, 14.2 μmol). To the resultant mixture was added sat. NH_4Cl aq. (2 mL). The aqueous layer was extracted with EtOAc (3 mL×3), and then the combined extracts were washed with brine (5 mL), dried over anhydrous Na_2SO_4, and concentrated in *vacuo*. Purification of the residue by silica gel column chromatography (5/1 PhMe/acetone) gave **44** (15.7 mg, 24.9 μmol, 77% yield).

Data for **44**: Colorless syrup; R_f 0.58 (3/1 PhMe/EtOAc); $[\alpha]^{24}_{D}$ −95.5° (*c* 1.0, $CHCl_3$); ^{1}H-NMR (500 MHz, $CDCl_3$) δ 7.39-7.26 (10H, m), 5.54 (1H, s), 5.04 (1H, d, *J*=4.0 Hz, H-1'), 4.89 and 4.63 (2H, ABq, *J*=11.0 Hz), 4.76 and 4.54 (2H, ABq, *J*=11.5 Hz), 4.68 (1H, m), 4.43 (1H, m), 4.31 (1H, m), 4.29 (1H, m), 4.22 (1H, m), 4.21 (1H, dd, *J*=4.0 Hz, *J*=10.0 Hz), 4.14 (1H, m), 3.92 (1H, q, *J*=6.5 Hz), 3.84 (1H, br-s), 3.73 (1H, br-s), 3.63 (1H, dd, *J*=2.5 Hz, *J*=10.0 Hz), 2.44 (1H, br-s), 1.23 (1H, d, *J*=6.5 Hz), 0.93 (9H, s), 0.13 (6H, s); ^{13}C-NMR (125 MHz, $CDCl_3$) δ 138.0, 137.4, 128.7, 128.4, 128.2, 127.9, 127.7, 102.8, 94.4, 79.7, 75.6, 75.4, 75.0, 72.2, 71.4, 70.0, 68.6, 68.5, 67.9, 67.1, 60.2, 25.9, 18.3, 16.8, −4.7×2; HRMS (ESI-TOF) 631.2958 (631.2939 calcd for $C_{33}H_{47}O_{10}Si$ $[M+H]^+$).

2-*O*-(3,4,6-Tri-*O*-benzyl-α-D-glucopyranosyl)-1,2-diphenylethan-1-ol (46 and 47)

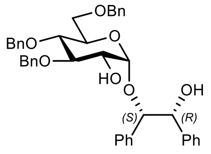

46

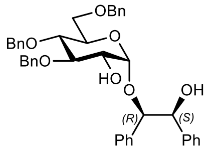

47

To a solution of **45** (5.5 mg, 25.7 μmol) and *p*-nitrophenylboronic acid (**3**) (0.86 mg, 5.15 μmol) in dry THF (194 μL) was added a solution of **7** (16.6 mg, 38.4 μmol) in dry THF (194 μL) at −20 ºC under Ar atmosphere. After the reaction mixture was stirred for 6 h, the reaction was quenched by addition of 0.05 M $NaBO_3$ aq. (0.124 mL, 6.18 μmol). To the resultant mixture was added sat. NH_4Cl aq. (2 mL). The aqueous layer was extracted with EtOAc (3 mL×3), and then the combined extracts were washed with brine (5 mL), dried over anhydrous Na_2SO_4, and concentrated in *vacuo*. Purification of the residue by preparative TLC (2/1 PhMe/EtOAc) gave major glycoside **46** (12.3 mg, 19.0 μmol, 74% yield) and minor glycoside **47** (3.2 mg, 4.95 μmol, 19% yield).

Data for major glycoside **46**: Colorless syrup; R_f 0.69 (2/1 PhMe/EtOAc); $[\alpha]^{25}_{D}$ +100.5° (*c* 0.97, $CHCl_3$); ^{1}H-NMR (500 MHz, $CDCl_3$) δ 7.37-7.21 (18H, m), 7.19-7.16 (2H, m), 4.92 (1H, dd, *J*=1.5 Hz, *J*=6.5 Hz), 4.84 and 4.80 (2H, ABq, *J*=11.0 Hz), 4.75 and 4.43 (2H, ABq, *J*=11.0 Hz), 4.72 (1H, d, *J*=4.0 Hz, H-1), 4.70 (1H, d, *J*=6.5 Hz), 4.56 and 4.42 (2H, ABq, *J*=12.0 Hz), 3.64 (1H, dd, *J*=9.0 Hz, *J*=9.0 Hz), 3.56 (1H, ddd, *J*=4.0 Hz, *J*=8.0 Hz, *J*=9.0 Hz), 3.47 (1H, dd, *J*=9.0 Hz, *J*=10.0 Hz), 3.44 (2H, br-d, *J*=3.0 Hz), 2.92 (1H, m), 2.44 (1H, br-s), 2.17 (1H, d, *J*=8.0 Hz); ^{13}C-NMR (125 MHz, $CDCl_3$) δ 140.7, 138.6, 138.5, 137.9, 136.0, 128.7, 128.5, 128.3×2, 128.2, 128.1, 128.0, 127.8, 127.7, 127.6, 127.4, 127.3, 126.9, 95.0, 82.9, 81.5, 77.3, 76.8, 75.2, 74.3, 73.4, 72.7, 70.6, 68.4; HRMS (ESI-TOF) *m/z* 685.2540 (685.2568 calcd for $C_{41}H_{42}O_7K$ $[M+K]^+$).

Data for minor glycoside **47**: Colorless syrup; R_f 0.48 (2/1 PhMe/EtOAc); $[\alpha]^{25}_D$ +92.0° (*c* 1.0, $CHCl_3$); ^{1}H-NMR (400 MHz, $CDCl_3$) δ 7.42-7.16 (18H, m), 7.13-7.08 (2H, m), 4.95 (1H, d, *J*=4.0 Hz, H-1), 4.91-4.82 (3H, m), 4.75 and 4.42 (2H, ABq, *J*=10.4 Hz), 4.70 (1H, d, *J*=6.0 Hz), 4.46 and 4.26 (2H, ABq, *J*=12.4 Hz), 3.72 (1H, dd, *J*=9.0 Hz, *J*=9.2 Hz), 3.65-3.47 (4H, m), 3.05 (1H, br-d, *J*=9.2 Hz), 2.86 (1H, br-s), 1.93 (1H, d, *J*=7.2 Hz); ^{13}C-NMR (100 MHz, $CDCl_3$) δ 140.0, 138.7, 138.6, 138.0, 137.8, 128.5, 128.4, 128.3, 128.2, 128.1, 128.0, 127.8, 127.6, 127.4, 127.2, 127.1, 100.9, 86.9, 82.9, 77.2, 75.2, 75.1, 73.2×2, 71.1, 67.7; HRMS (ESI-TOF) *m/z* 685.2574 (685.2568 calcd for $C_{41}H_{42}O_7K$ $[M+K]^+$).

(1*S*,2*R*)-2-*O*-(3,4,6-Tri-*O*-benzyl-α-D-glucopyranosyl)-dibenzyl-tartrate (49)

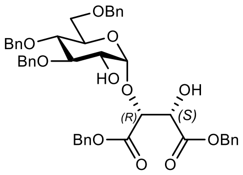

49

To a solution of **48**[12] (11.3 mg, 34.2 μmol) and *p*-nitrophenylboronic acid (**3**) (1.14 mg, 6.83 μmol) in dry THF (256 μL) was added a solution of **7** (22.2 mg, 51.3 μmol) in dry THF (256 μL) at −40 °C under Ar atmosphere. After the reaction mixture was stirred for 10 h, the reaction was quenched by addition of 0.05 M $NaBO_3$ aq. (0.164 mL, 8.20 μmol). To the resultant mixture was added sat. NH_4Cl aq. (2 mL). The aqueous layer was extracted with EtOAc (3 mL×3), and then the combined extracts were washed with brine (5 mL), dried over anhydrous Na_2SO_4, and concentrated in *vacuo*. Purification of the residue by preparative TLC (3/2 *n*-hexane/EtOAc and 2/1 *n*-hexane/acetone) gave **49** (24.8 mg, 32.5 μmol, 95% yield).

Data for **49**: Colorless syrup; R_f 0.59 (1/1 *n*-hexane/EtOAc); $[\alpha]^{26}_D$ +73.6° (*c* 0.98, $CHCl_3$); ^{1}H-NMR (500 MHz, $CDCl_3$) δ 7.42-7.38 (2H, m), 7.36-7.24 (16H, m), 7.15-7.11 (2H, m), 5.14 and 4.98 (2H, ABq, *J*=11.5 Hz), 5.09 and 5.04 (2H, ABq, *J*=12.5 Hz), 4.97 and 4.73 (2H, ABq, *J*=11.0 Hz), 4.92 (1H, d, *J*=4.0 Hz, H-1), 4.80 and 4.44 (2H, ABq, *J*=11.0 Hz), 4.75 (1H, br-s), 4.58 and 4.47 (2H, ABq, *J*=12.5 Hz), 4.52 (1H, d, *J*=2.0 Hz), 3.91 (1H, m), 3.72 (1H, m), 3.67-3.60 (2H, m), 3.67 (1H, br-s), 3.56 (1H, dd, *J*=9.0 Hz, *J*=9.0 Hz), 3.49 (1H, dd, *J*=9.0 Hz, *J*=9.0 Hz), 2.95 (1H, d, *J*=10.0 Hz); ^{13}C-NMR (125 MHz, $CDCl_3$) δ 170.4, 168.7, 138.8, 138.1, 137.7, 134.6, 134.5, 128.8×2, 128.7, 128.6, 128.3×2, 128.0, 127.9×2, 127.7, 127.5, 100.9, 83.5, 80.3, 76.7, 75.3, 75.0, 73.4, 73.3, 71.9, 71.5, 68.4, 68.1, 67.8; HRMS (ESI-TOF) *m/z* 785.2968 (785.2938 calcd for $C_{45}H_{46}O_{11}Na$ $[M+Na]^+$).

Determination of Glycosylation Site

Determination of glycosylation sites of α(1,6)-D-glucosides.

The glycosylation sites of α(1,6)-D-glucosides were determined as described below.

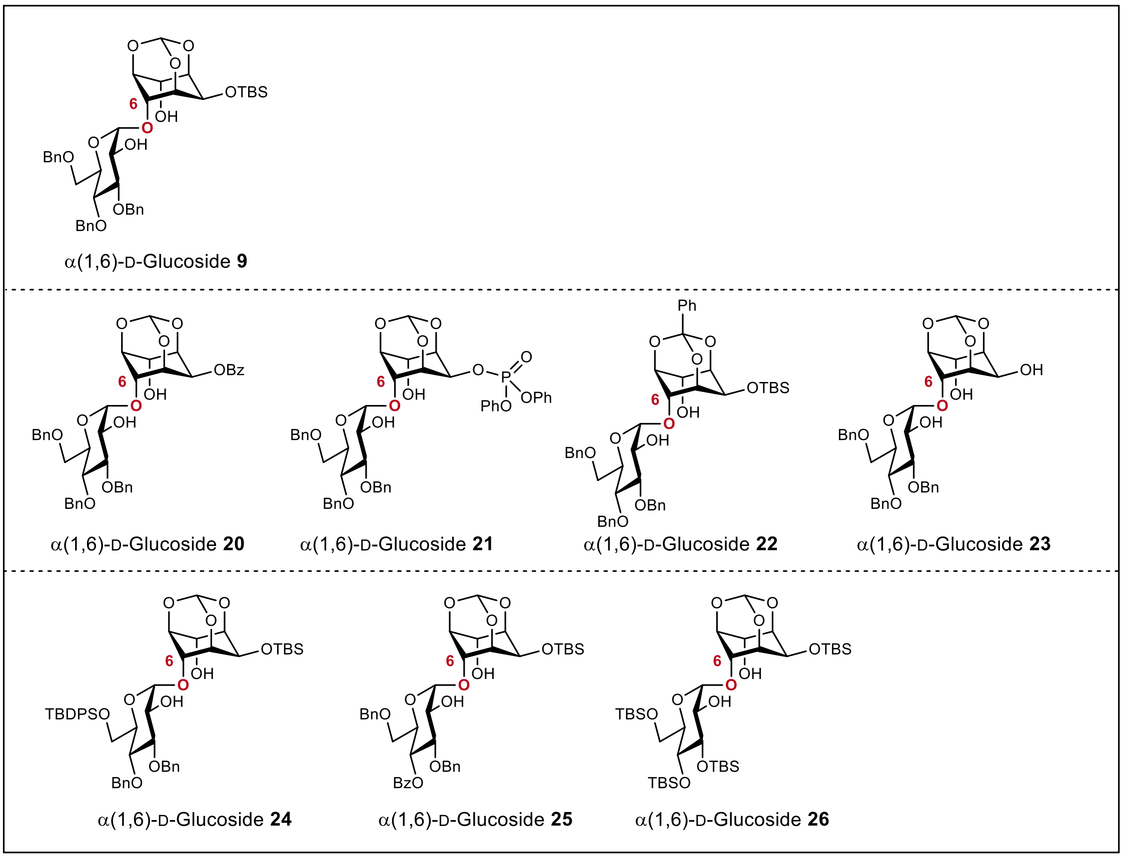

Supplementary Figure 2. Chemical structures of α(1,6)-D-glucosides synthesized by the present desymmetric glycosylation method.

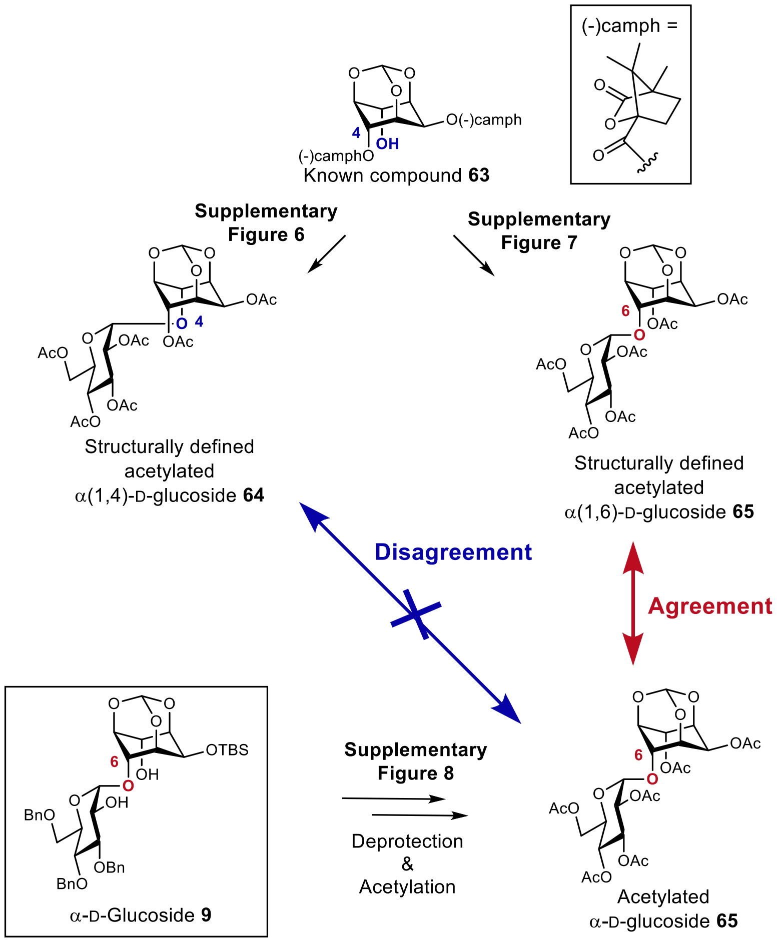

Supplementary Figure 3. Determination of glycosylation site of α-D-glucoside 9.

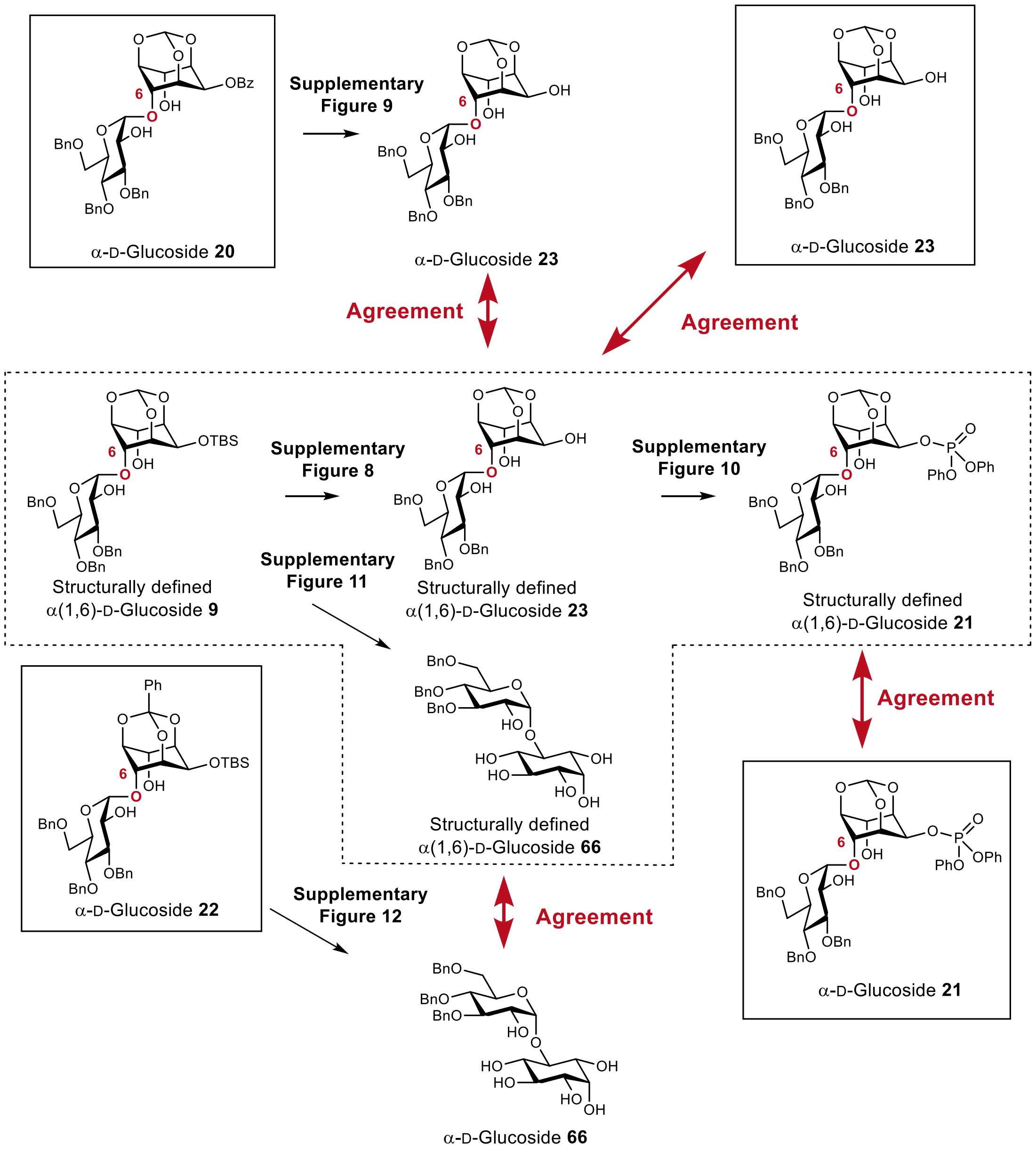

Supplementary Figure 4. Determination of glycosylation sites of α-D-glucosides 20-23.

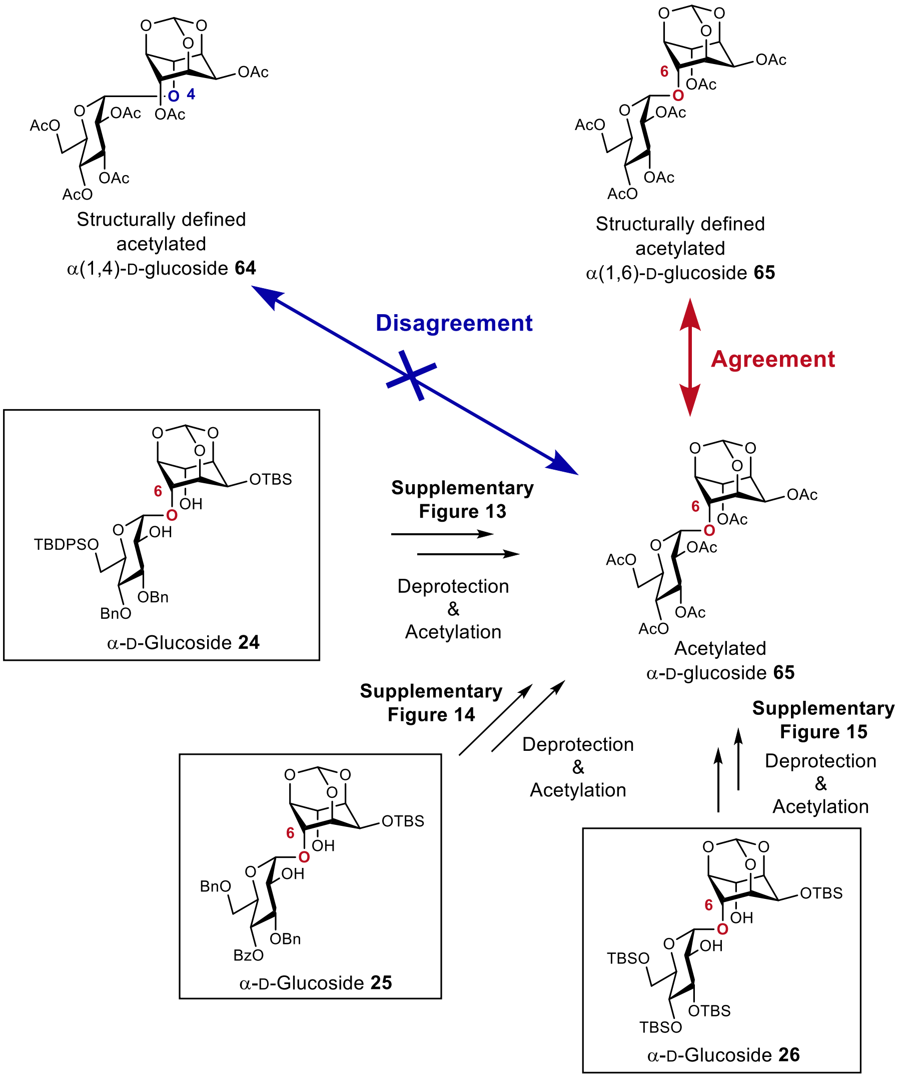

Supplementary Figure 5. Determination of glycosylation sites of α-D-glucosides 24-26.

<u>Synthesis of acetylated α(1,4)-D-glucoside **64** from optically pure inositol **63**.</u>

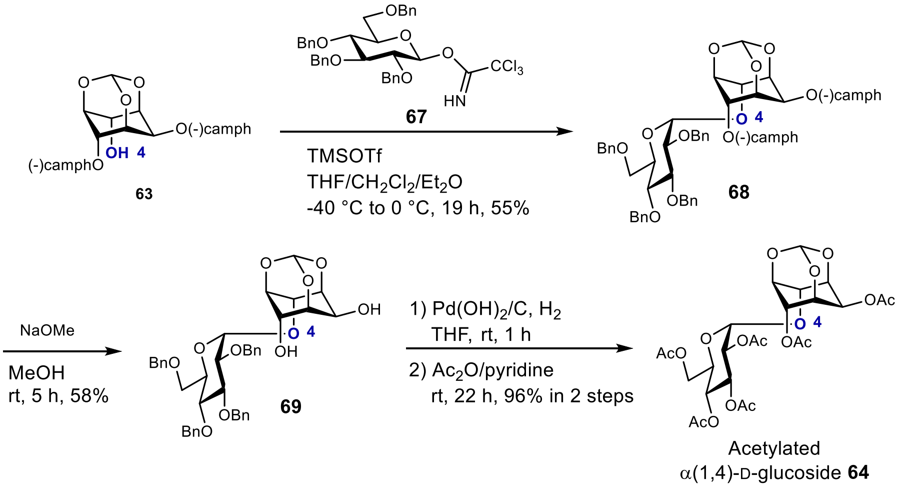

Supplementary Figure 6. Synthesis of acetylated α(1,4)-D-glucoside 64 from optically pure inositol 63.

4-*O*-(2,3,4,6-Tetra-*O*-benzyl-α-D-glucopyranosyl)-2,6-di-*O*-[(-)-ω-camphanoyl]-D-*myo*-inositol-1,3,5-orthoformate (68)

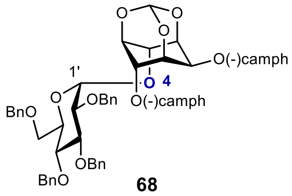

68

To a solution of **67**[14] (54.9 mg, 80.3 μmol) and **63**[13] (29.5 mg, 53.6 μmol) in dry THF-CH_2Cl_2-Et_2O (2/1/1, v/v/v, 803 μL) was added TMSOTf (1.45 μL, 8.03 μmol) at −40 °C. After the reaction mixture was gradually warmed to 0 °C over a period of 4 h and stirred for 15 h, the reaction was quenched by addition of sat. $NaHCO_3$ aq. (1 mL). The resultant mixture was extracted with $CHCl_3$ (5 mL×3), and then the extracts were washed with brine (5 mL), dried over anhydrous Na_2SO_4, and concentrated in *vacuo*. The residue was subjected to silica gel column chromatography (10/1 $CHCl_3$/EtOAc) to give **68** (31.8 mg, 29.7 μmol, 55% yield).

Data for **68**: Colorless syrup; R_f 0.70 (6/1 $CHCl_3$/EtOAc); $[\alpha]^{24}_D$ +19.3° (*c* 1.0, $CHCl_3$); ^{1}H-NMR (500 MHz, $CDCl_3$) δ 7.36-7.21 (18H, m), 7.19-7.17 (2H, m), 5.54-5.53 (2H, m), 5.26 (1H, d, *J*=1.5 Hz), 4.84 and 4.76 (2H, ABq, *J*=11.0 Hz), 4.77 (1H, d, *J*= 4.0 Hz, H-1'), 4.76 and 4.54 (2H, ABq, *J*=12.0 Hz), 4.73 and 4.51 (2H, ABq, *J*=12.5 Hz), 4.64 (1H, m), 4.55 and 4.45 (2H, ABq, *J*=12.0 Hz), 4.51 (1H, m), 4.35-4.33 (2H, m), 4.04 (1H, m), 3.93 (1H, dd, *J*=9.5 Hz, *J*=9.5 Hz), 3.83 (1H, dd, *J*=3.0 Hz, *J*=11.0 Hz), 3.68 (1H, dd, *J*=9.5 Hz, *J*=9.5 Hz), 3.63 (1H, dd, *J*=1.5 Hz, *J*=11.0 Hz), 3.54 (1H, dd, *J*=4.0 Hz, *J*=9.5 Hz), 2.45 (1H, m), 2.22-2.07 (3H, m), 1.93 (1H, m), 1.71 (1H, m), 1.61-1.55 (2H, m), 1.12 (3H, s), 1.09 (3H, s), 0.97 (3H, s), 0.96 (3H, s), 0.93 (6H, s); ^{13}C-NMR (125 MHz, $CDCl_3$) δ 178.0, 177.6, 167.1, 166.3, 139.0, 138.3, 138.0, 137.9, 128.6, 128.3×3, 128.2×2, 127.9, 127.8, 127.5, 127.4×2, 127.3, 103.0, 101.1, 90.9, 90.5, 81.2, 80.0, 77.5, 76.0, 75.1, 74.5, 73.7, 73.5, 71.1, 70.1, 69.3, 68.9, 68.4, 67.7, 65.1, 54.7×2, 54.6, 54.4, 30.6, 30.1, 29.0, 28.4, 16.7×2, 16.5×2, 9.7, 9.6; HRMS (ESI-TOF) 1095.4406 (1095.4354 calcd for $C_{61}H_{68}O_{17}Na$ $[M+Na]^+$).

4-*O*-(2,3,4,6-Tetra-*O*-benzyl-α-D-glucopyranosyl)-D-*myo*-inositol-1,3,5-orthoformate (69)

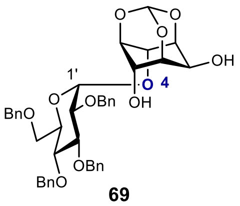

69

To a solution of **68** (31.8 mg, 29.7 μmol) in MeOH (594 μL) was added NaOMe (5.75 μL, 29.7 μmol, 28% in MeOH) at room temperature. After the reaction mixture was stirred for 5 h at room temperature, the reaction was quenched by addition of sat. NH_4Cl aq. (2 mL). The resultant mixture was extracted with $CHCl_3$ (5 mL×3), and then the extracts were washed with brine (5 mL), dried over anhydrous Na_2SO_4, and concentrated in *vacuo*. The residue was subjected to silica gel column chromatography (10/1 $CHCl_3$/EtOAc) to give **69** (12.2 mg, 17.1 μmol, 58% yield).

Data for **69**: Colorless syrup; R_f 0.35 (6/1 $CHCl_3$/EtOAc); $[\alpha]^{23}_D$ +34.5° (*c* 0.73, $CHCl_3$); ^{1}H-NMR (500 MHz, $CDCl_3$) δ 7.37-7.26 (18H, m), 7.14-7.12 (2H, m), 5.46 (1H, d, *J*=1.0 Hz), 4.86 and 4.83 (2H, ABq, *J*=11.0 Hz), 4.81 and 4.47 (2H, ABq, *J*=10.5 Hz), 4.79 and 4.63 (2H, ABq, *J*=12.0 Hz), 4.75 (1H, d, *J*=4.0 Hz, H-1'), 4.57 and 4.46 (2H, ABq, *J*=12.0 Hz), 4.58-4.52 (2H, m), 4.36 (1H, d, *J*=9.0 Hz), 4.22 (1H, m), 4.20 (1H, m), 4.13-4.09 (2H, m), 3.81 (1H, dd, *J*=9.5 Hz, *J*=9.5 Hz), 3.72-3.59 (4H, m), 3.55 (1H, dd, *J*=4.0 Hz, *J*=9.5 Hz), 3.06 (1H, d, *J*=12.0 Hz); ^{13}C-NMR (125 MHz, $CDCl_3$) δ 138.2, 137.8, 137.5, 137.1, 128.7×2, 128.5, 128.4, 128.0, 127.9×2, 127.8×2, 102.8, 96.7, 81.8, 78.2, 75.8, 75.2, 74.8, 74.4, 73.6, 72.8, 71.5, 70.9, 67.8×2, 66.6, 60.7; HRMS (ESI-TOF) 713.2985 (713.2962 calcd for $C_{41}H_{45}O_{11}$ $[M+H]^+$).

2,6-Di-*O*-acetyl-4-*O*-(2,3,4,6-tetra-*O*-acetyl-α-D-glucopyranosyl)-D-*myo*-inositol-1,3,5-orthoformate (64)

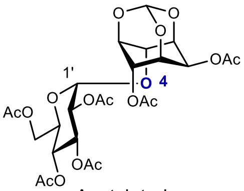

Acetylated
α(1,4)-D-glucoside **64**

To a solution of compound **69** (12.2 mg, 17.1 μmol) in THF (1.71 mL) was added 20% $Pd(OH)_2$/C (12.2 mg, wetted with 50% water) at room temperature under Ar atmosphere. After changing the atmosphere to H_2 (balloon), the reaction mixture was stirred for 1 h. After changing the

atmosphere to Ar, the reaction was filtered through celite pad, and the filtrate was concentrated in *vacuo*. To a solution of the residue in pyridine (428 μL) was added Ac_2O (428 μL) at room temperature. After the reaction mixture was stirred for 22 h at room temperature, the reaction was quenched by addition of H_2O (5 mL). The resultant mixture was extracted with EtOAc (5 mL×3), and then the extracts were washed with brine (5 mL), dried over anhydrous Na_2SO_4, and concentrated in *vacuo*. The residue was subjected to silica gel column chromatography (1/1 *n*-hexane/EtOAc) to give **64** (9.9 mg, 16.4 μmol, 96% yield in 2 steps).

Data for **64**: Colorless syrup; R_f 0.23 (6/1 $CHCl_3$/EtOAc); $[\alpha]^{24}_D$ +23.6° (*c* 0.66, $CHCl_3$); ^{1}H-NMR (500 MHz, $CDCl_3$) δ 5.55 (1H, d, *J*=1.5 Hz), 5.38 (1H, m), 5.36 (1H, dd, *J*=10.0 Hz, *J*=10.5 Hz), 5.26 (1H, d, *J*=4.0 Hz, H-1'), 5.23 (1H, m), 5.10 (1H, dd, *J*=10.0 Hz, *J*=10.0 Hz), 4.82 (1H, dd, *J*=4.0 Hz, *J*=10.5 Hz), 4.55 (1H, m), 4.42-4.30 (5H, m), 4.06 (1H, m), 2.22 (3H, s), 2.21 (3H, s), 2.09 (3H, s), 2.06 (6H, s), 2.02 (3H, s); ^{13}C-NMR (125 MHz, $CDCl_3$) δ 170.5×2, 170.4, 170.0, 169.9, 169.5, 102.9, 99.6, 77.1, 70.7, 70.6, 69.6, 69.4, 68.0×2, 67.8, 66.9, 63.6, 61.7, 21.0, 20.9, 20.7, 20.6×2, 20.4; HRMS (ESI-TOF) 605.1744 (605.1718 calcd for $C_{25}H_{33}O_{17}$ $[M+H]^+$).

Synthesis of acetylated α(1,6)-D-glucoside **65** from optically pure inositol **63**.

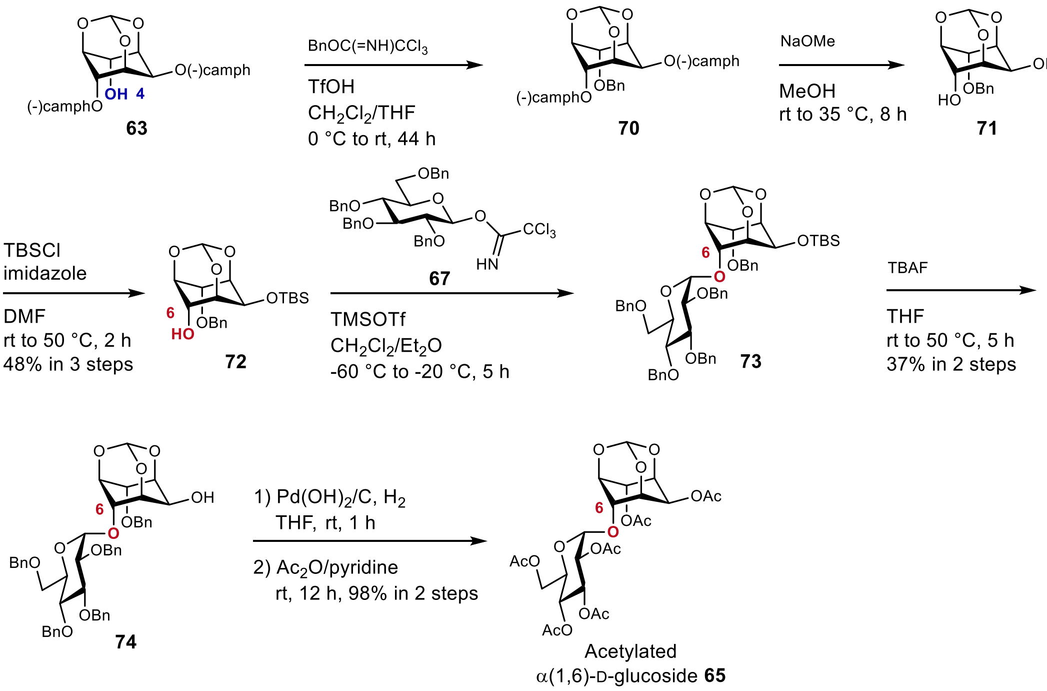

Supplementary Figure 7. Synthesis of acetylated α(1,6)-D-glucoside 65 from optically pure inositol 63.

4-*O*-Benzyl-2-*O*-*tert*-butyldimethylsilyl-D-*myo*-inositol-1,3,5-orthoformate (72)

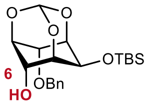

72

To a solution of **63**[13] (71.4 mg, 0.130 mmol) and benzyl 2,2,2-trichloroacetimidate (72.2 mL, 0.389 mmol) in CH_2Cl_2-THF (1/1, v/v, 1.30 mL) was added TfOH (2.29 μL, 25.9 μmol) at 0 °C. After the reaction mixture was stirred for 44 h at room temperature, the reaction was quenched by addition of sat. $NaHCO_3$ aq. (5 mL). The resultant mixture was extracted with $CHCl_3$ (5 mL×3), and then the extracts were washed with brine (5 mL), dried over anhydrous Na_2SO_4, and concentrated in *vacuo*. The residue was subjected to silica gel column chromatography (10/1 *n*-hexane/EtOAc) to give crude **70** (59.0 mg).

To a solution of crude **70** (59.0 mg) in MeOH (1.80 mL) was added NaOMe (178 μL, 0.920 mmol, 28% in MeOH) at room temperature. After the reaction mixture was stirred for 8 h at 35 °C, the reaction mixture was concentrated in *vacuo*. The residue was added to sat. NH_4Cl aq. (2 mL) and extracted with $CHCl_3$ (5 mL×3), and then the extracts were washed with brine (5 mL), dried over anhydrous Na_2SO_4, and concentrated in *vacuo*. The residue was subjected to silica gel column chromatography (10/1 $CHCl_3$/MeOH) to give crude **71** (25.4 mg).

To a solution of crude **71** (25.4 mg) and imidazole (51.8 mg, 0.761 mmol) in DMF (0.906 mL) was added TBSCl (57.4 mg, 0.381 mmol) at room temperature. After the reaction mixture was gradually warmed to 50 °C over a period of 1 h and was stirred for 1 h, the reaction was quenched by addition of H_2O (2 mL). The resultant mixture was extracted with EtOAc (5 mL×3), and then the extracts were washed with brine (5 mL), dried over anhydrous Na_2SO_4, and concentrated in *vacuo*. The residue was subjected to silica gel column chromatography (4/1 PhMe/EtOAc) to give **72** (24.6 mg, 62.2 μmol, 48% in 3 steps).

Data for **72**: Colorless syrup; R_f 0.67 (3/1 PhMe/EtOAc); $[\alpha]^{25}_D$ +11.4° (*c* 1.0, $CHCl_3$); ^{1}H-NMR (500 MHz, $CDCl_3$) δ 7.41-7.34 (3H, m), 7.31-7.30 (2H, m), 5.49 (1H, d, *J*=1.5 Hz), 4.67 (2H, s), 4.44 (1H, m), 4.39 (1H, m), 4.26 (1H, m), 4.22 (1H, m), 4.14 (1H, m), 3.65 (1H, d, *J*=10.5 Hz), 0.95 (9H, s), 0.15 (6H, s); ^{13}C-NMR (125 MHz, $CDCl_3$) δ 135.9, 128.8, 128.7, 128.0, 102.5, 74.9, 74.8, 73.1, 72.5, 68.3, 67.5, 60.8, 25.9, 18.4, −4.6, −4.7; HRMS (ESI-TOF) 395.1899 (395.1890 calcd for $C_{20}H_{31}O_6Si$ $[M+H]^+$).

4-*O*-Benzyl-6-*O*-(2,3,4,6-tetra-*O*-benzyl-α-D-glucopyranosyl)-D-*myo*-inositol-1,3,5-orthoformate (74)

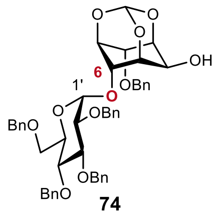

74

To a solution of **67**[14] (40.6 mg, 59.3 μmol) and **72** (11.7 mg, 29.7 μmol) in dry CH_2Cl_2-Et_2O (1/1, v/v, 593 μL) was added TMSOTf (0.80 μL, 4.45 μmol) at −60 °C. After the reaction mixture was gradually warmed to −20 °C over a period of 2 h and was stirred for 3 h, the reaction was quenched by addition of Et_3N (10 μL) and H_2O (1 mL). The resultant mixture was extracted with EtOAc (5 mL×3), and then the extracts were washed with brine (5 mL), dried over anhydrous Na_2SO_4, and concentrated in *vacuo*. The residue was subjected to silica gel column chromatography (2/1 *n*-hexane/Et_2O) to give crude **73** (18.1 mg).

To a solution of crude **73** (18.1 mg) in THF (394 μL) was added TBAF (39.5 μL, 39.5 μmol, 1.0 M in THF) at room temperature. After the reaction mixture was gradually warmed to 50 °C over a period of 1 h and was stirred for 4 h, the reaction mixture was concentrated in *vacuo*. The residue was subjected to silica gel column chromatography (3/1 *n*-hexane/EtOAc) to give **74** (8.8 mg, 11.0 μmol, 37% yield in 2 steps).

Data for **74**: Colorless syrup; R_f 0.40 (6/1 PhMe/acetone); $[\alpha]^{25}_D$ +40.2° (*c* 0.88, $CHCl_3$); ^{1}H-NMR (500 MHz, $CDCl_3$) δ 7.36-7.23 (20H, m), 7.15-7.09 (5H, m), 5.47 (1H, d, *J*=1.0 Hz), 4.79 (1H, d, *J*=3.5 Hz, H-1'), 4.77 and 4.44 (2H, ABq, *J*=11.5 Hz), 4.74 and 4.56 (2H, ABq, *J*=12.0 Hz), 4.67 and 4.53 (2H, ABq, *J*=11.5 Hz), 4.62 and 4.41 (2H, ABq, *J*=11.0 Hz), 4.57 and 4.46 (2H, ABq, *J*=12.0 Hz), 4.46 (1H, m), 4.38-4.34 (2H, m), 4.31 (1H, m), 4.25 (1H, m), 4.13 (1H, m), 3.93 (1H, m), 3.72 (1H, dd, *J*=9.5 Hz, *J*=9.5 Hz), 3.65 (1H, dd, *J*=3.5 Hz, *J*=10.5 Hz), 3.59 (1H, dd, *J*=2.5 Hz, *J*=10.5 Hz), 3.57 (1H, dd, *J*=9.5 Hz, *J*=9.5 Hz), 3.49 (1H, dd, *J*=3.5 Hz, *J*=9.5 Hz), 2.90 (1H, d, *J*=11.5 Hz); ^{13}C-NMR (125 MHz, $CDCl_3$) δ 138.7, 138.4, 138.2, 137.8, 137.4, 128.6, 128.4×2, 128.3, 128.2, 128.0×2, 127.9, 127.8×2, 127.7, 127.6, 127.5×2, 103.2, 97.7, 81.6, 79.7, 77.5, 75.3, 74.9, 73.9, 73.5, 72.8, 72.6, 72.5, 71.8, 71.1, 69.0, 68.4, 61.1; HRMS (ESI-TOF) 825.3275 (825.3251 calcd for $C_{48}H_{50}O_{11}Na$ $[M+Na]^+$).

2,4-Di-*O*-acetyl-6-*O*-(2,3,4,6-tetra-*O*-acetyl-α-D-glucopyranosyl)-D-*myo*-inositol-1,3,5-orthoformate (65)

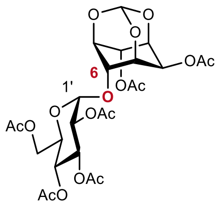

Acetylated
α(1,6)-D-glucoside **65**

To a solution of **74** (8.8 mg, 11.0 μmol) in THF (1.1 mL) was added 20% $Pd(OH)_2/C$ (8.8 mg, wetted with 50% water) at room temperature under Ar atmosphere. After changing the atmosphere to H_2 (balloon), the reaction mixture was stirred for 1 h. After changing the atmosphere to Ar, the reaction was filtered through celite pad, and the filtrate was concentrated in *vacuo*. To a solution of the residue in pyridine (550 μL) was added Ac_2O (550 μL) at room temperature. After the reaction mixture was stirred for 12 h at room temperature, the reaction was quenched by addition of H_2O (5 mL). The resultant mixture was extracted with EtOAc (5 mL×3), and then the extracts were washed with brine (5 mL), dried over anhydrous Na_2SO_4, and concentrated in *vacuo*. The residue was subjected to silica gel column chromatography (1/1 *n*-hexane/EtOAc) to give **65** (6.5 mg, 10.8 μmol, 98% yield in 2 steps).

Data for **65**: Colorless syrup; R_f 0.34 (1/1 *n*-hexane/EtOAc); $[\alpha]^{26}_{D}$ +102.4° (*c* 0.65, $CHCl_3$); ^{1}H-NMR (500 MHz, $CDCl_3$) δ 5.58 (1H, d, *J*=1.0 Hz), 5.50 (1H, m), 5.36 (1H, dd, *J*=10.0 Hz, *J*=10.5 Hz), 5.24 (1H, d, *J*=4.0 Hz, H-1'), 5.17 (1H, m), 5.05 (1H, dd, *J*=9.5 Hz, *J*=10.0 Hz), 4.90 (1H, dd, *J*=4.0 Hz, *J*=10.5 Hz), 4.60 (1H, m), 4.52 (1H, m), 4.33 (1H, m), 4.30 (1H, m), 4.24 (1H, dd, *J*=5.0 Hz, *J*=12.5 Hz), 4.10 (1H, dd, *J*=2.0 Hz, *J*=12.5 Hz), 3.97 (1H, m), 2.27 (3H, s), 2.20 (3H, s), 2.11 (6H, s), 2.03 (3H, s), 2.01 (3H, s); ^{13}C-NMR (125 MHz, $CDCl_3$) δ 170.5, 170.3, 170.2×2, 170.0, 169.4, 103.0, 94.5, 70.6, 70.1, 69.7, 69.4, 68.6, 68.3, 68.0, 67.9, 67.8, 62.9, 61.8, 21.0, 20.8, 20.7, 20.6, 20.5×2; HRMS (ESI-TOF) 605.1696 (605.1718 calcd for $C_{25}H_{33}O_{17}$ $[M+H]^+$).

Synthesis of acetylated α-D-glucoside **65** from **9** synthesized by our desymmetric glycosylation of *meso*-diol **8**.

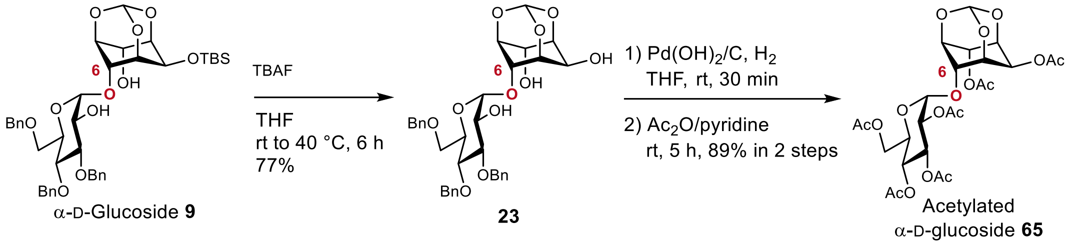

Supplementary Figure 8. Synthesis of acetylated α-D-glucoside 65 from 9.

6-*O*-(3,4,6-Tri-*O*-benzyl-α-D-glucopyranosyl)-D-*myo*-inositol-1,3,5-orthoformate (23)

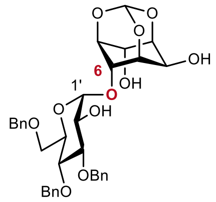

23

To a solution of **9** (13.4 mg, 18.2 μmol) in THF (363 μL) was added TBAF (36.3 μL, 36.3 μmol, 1.0 M in THF) at room temperature. After the reaction mixture was gradually warmed to 40 °C over a period of 1 h and was stirred for 5 h, the reaction mixture was concentrated in *vacuo*. The residue was subjected to silica gel column chromatography (1/1 PhMe/EtOAc) to give **23** (8.7 mg, 14.0 μmol, 77% yield).

Data for **23**: Colorless syrup; R_f 0.43 (1/2 PhMe/EtOAc); $[\alpha]^{26}_{D}$ +95.8° (*c* 1.12, $CHCl_3$); ^{1}H-NMR (500 MHz, $CDCl_3$) δ 7.36-7.28 (13H, m), 7.17-7.15 (2H, m), 5.47 (1H, d, *J*=1.0 Hz), 5.02 (1H, d, *J*=3.5 Hz, H-1'), 4.90 and 4.69 (2H, ABq, *J*=11.0 Hz), 4.78 and 4.51 (2H, ABq, *J*=11.0 Hz), 4.68 (1H, m), 4.62 and 4.51 (2H, ABq, *J*=12.0 Hz), 4.42 (1H, m), 4.36 (1H, m), 4.27 (1H, m), 4.18 (1H, m), 4.11 (1H, m), 3.83 (1H, m), 3.75-3.62 (6H, m), 3.21 (1H, d, *J*=11.0 Hz), 2.40 (1H, br-s); ^{13}C-NMR (125 MHz, $CDCl_3$) δ 138.1, 137.6, 137.5, 128.7, 128.5, 128.1, 128.0, 127.9×3, 102.8, 95.4, 82.1, 75.5, 75.0, 74.7, 73.7, 71.8, 71.2, 71.0, 70.6, 68.5, 68.1, 67.7, 60.2; HRMS (ESI-TOF) *m/z* 645.2282 (645.2312 calcd for $C_{34}H_{38}O_{11}Na$ $[M+Na]^+$).

2,4-Di-*O*-acetyl-6-*O*-(2,3,4,6-tetra-*O*-acetyl-α-D-glucopyranosyl)-D-*myo*-inositol-1,3,5-orthoformate (65)

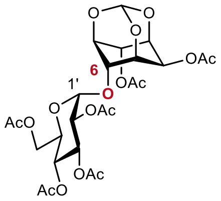

Acetylated
α-D-glucoside **65**

To a solution of **23** (8.7 mg, 14.0 μmol) in THF (1.4 mL) was added 20% $Pd(OH)_2/C$ (8.7 mg, wetted with 50% water) at room temperature under Ar atmosphere. After changing the atmosphere to H_2 (balloon), the reaction mixture was stirred for 30 min. After changing the atmosphere to Ar, the reaction was filtered through celite pad, and the filtrate was concentrated in *vacuo*. To a solution of the residue in pyridine (350 μL) was added Ac_2O (350 μL) at room temperature. After the reaction mixture was stirred for 5 h at room temperature, the reaction was quenched by addition of H_2O (5 mL). The resultant mixture was extracted with EtOAc (5 mL×3), and then the extracts were washed with brine (5 mL), dried over anhydrous Na_2SO_4, and concentrated in *vacuo*. The residue was subjected to silica gel column chromatography (1/1 *n*-hexane/EtOAc) to give **65** (7.5 mg, 12.5 μmol, 89% yield in 2 steps).

Data for **65**: Colorless syrup; R_f 0.34 (1/1 *n*-hexane/EtOAc); $[\alpha]^{23}_D$ +106.1° (*c* 0.76, $CHCl_3$); ^{1}H-NMR (500 MHz, $CDCl_3$) δ 5.57 (1H, d, *J*=1.5 Hz), 5.50 (1H, m), 5.36 (1H, dd, *J*=10.0 Hz, *J*=10.0 Hz), 5.24 (1H, d, *J*=3.5 Hz, H-1'), 5.17 (1H, m), 5.05 (1H, dd, *J*=9.5 Hz, *J*=10.0 Hz), 4.90 (1H, dd, *J*=3.5 Hz, *J*=10.0 Hz), 4.60 (1H, m), 4.52 (1H, m), 4.33 (1H, m), 4.30 (1H, m), 4.24 (1H, dd, *J*=5.0 Hz, *J*=12.5 Hz), 4.10 (1H, dd, *J*=2.0 Hz, *J*=12.5 Hz), 3.97 (1H, m), 2.27 (3H, s), 2.20 (3H, s), 2.11 (3H, s), 2.10 (3H, s), 2.03 (3H, s), 2.01 (3H, s); ^{13}C-NMR (125 MHz, $CDCl_3$) δ 170.5, 170.3, 170.2×2, 170.0, 169.4, 103.0, 94.5, 70.6, 70.1, 69.7, 69.4, 68.6, 68.3, 68.0, 67.9, 67.8, 62.9, 61.8, 21.0, 20.8, 20.7, 20.6, 20.5×2; HRMS (ESI-TOF) 605.1690 (605.1718 calcd for $C_{25}H_{33}O_{17}$ $[M+H]^+$).

Determination of glycosylation site of 9

Comparison of NMR data of acetylated α-D-glucosides **64** and **65**

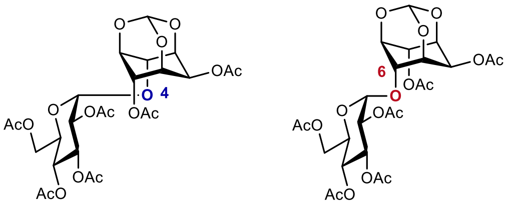

Acetylated α(1,4)-D-glucoside **64**

Acetylated α(1,6)-D-glucoside **65**

Supplementary Table 2. Comparison of ^{1}H-NMR data of acetylated α-D-glucosides 64 and 65.

δ(Structurally defined acetylated α(1,4)-D-glucoside **64**) (500 MHz, $CDCl_3$)	δ(Structurally defined acetylated α(1,6)-D-glucoside **65**) (500 MHz, $CDCl_3$)	δ(Acetylated α-D-glucoside **65** derived from **9**) (500 MHz, $CDCl_3$)
5.55 (d, *J*=1.5 Hz)	5.58 (d, *J*=1.0 Hz)	5.57 (d, *J*=1.5 Hz)
5.38 (m)	5.50 (m)	5.51 (m)
5.36 (dd, *J*=10.0, 10.5 Hz)	5.36 (dd, *J*=10.0, 10.5 Hz)	5.36 (dd, *J*=10.0, 10.0 Hz)
5.26 (d, *J*=4.0 Hz)	5.24 (d, *J*=4.0 Hz)	5.24 (d, *J*=3.5 Hz)
5.23 (m)	5.17 (m)	5.17 (m)
5.10 (dd, *J*=10.0, 10.0 Hz)	5.05 (dd, *J*=9.5, 10.0 Hz)	5.05 (dd, *J*=9.0, 10.0 Hz)
4.82 (dd, *J*=4.0, 10.5 Hz)	4.90 (dd, *J*=4.0, 10.5 Hz)	4.90 (dd, *J*=3.5, 10.5 Hz)
4.55 (m)	4.60 (m)	4.59 (m)
4.42-4.30 (m)	4.52 (m)	4.52 (m)
	4.33 (m)	4.33 (m)
	4.30 (m)	4.30 (m)
	4.24 (dd, *J*=5.0, 12.5 Hz)	4.24 (dd, *J*=5.0, 12.5 Hz)
	4.10 (dd, *J*=2.0, 12.5 Hz)	4.10 (dd, *J*=2.0, 12.0 Hz)
4.06 (m)	3.97 (m)	3.97 (m)
2.22 (s)	2.27 (s)	2.27 (s)
2.21 (s)	2.20 (s)	2.20 (s)
2.09 (s)	2.11×2 (s)	2.11 (s)
2.06×2 (s)		2.10 (s)
	2.03 (s)	2.03 (s)
2.02 (s)	2.01 (s)	2.01 (s)

Supplementary Table 3. Comparison of ^{13}C-NMR data of acetylated α-D-glucosides 64 and 65.

δ(Structurally defined acetylated α(1,4)-D-glucoside **64**) (125 MHz, $CDCl_3$)	δ(Structurally defined acetylated α(1,6)-D-glucoside **65**) (125 MHz, $CDCl_3$)	δ(Acetylated α-D-glucoside **65** derived from **9**) (125 MHz, $CDCl_3$)
170.5×2	170.5	170.5
170.4	170.3	170.3
170.0	170.2×2	170.2×2
169.9	170.0	170.0
169.5	169.4	169.4
102.9	103.0	103.0
99.6	94.5	94.5

77.1	70.6	70.6
70.7	70.1	70.1
70.6	69.7	69.7
69.6	69.4	69.4
69.4	68.6	68.6
68.0×2	68.3	68.3
	68.0	68.0
67.8	67.9	67.9
66.9	67.8	67.8
63.6	62.9	62.9
61.7	61.8	61.8
21.0	21.0	21.0
20.9	20.8	20.8
20.7	20.7	20.7
20.6×2	20.6	20.6
20.4	20.5×2	20.5×2

Synthesis of α-D-glucoside **23** from **20** synthesized by our desymmetric glycosylation of *meso*-diol **13**.

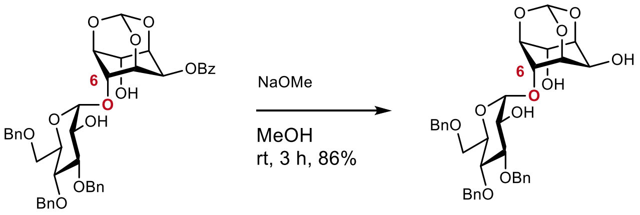

α-D-Glucoside **20** α-D-Glucoside **23**

Supplementary Figure 9. Synthesis of α-D-glucoside 23 from 20.

6-*O*-(3,4,6-Tri-*O*-benzyl-α-D-glucopyranosyl)-D-*myo*-inositol-1,3,5-orthoformate (23)

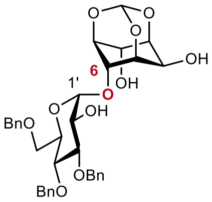

α-D-Glucoside **23**

To a solution of **20** (13.0 mg, 17.9 μmol) in MeOH (1.8 mL) was added NaOMe (0.69 μL, 3.58 μmol, 28% in MeOH) at room temperature. After the reaction mixture was stirred for 3 h at room temperature, the reaction was quenched by addition of sat. NH_4Cl aq. (2 mL). The resultant mixture was extracted with EtOAc (5 mL×3), and then the extracts were washed with brine (5 mL), dried over anhydrous Na_2SO_4, and concentrated in *vacuo*. The residue was subjected to silica gel column chromatography (2/3 *n*-hexane/EtOAc) to give **23** (9.6 mg, 15.4 μmol, 86% yield).

Data for **23**: Colorless syrup; R_f 0.43 (1/2 PhMe/EtOAc); $[\alpha]^{26}_{D}$ +95.5° (*c* 0.86, $CHCl_3$); ^{1}H-NMR (500 MHz, $CDCl_3$) δ 7.37-7.26 (13H, m), 7.17-7.15 (2H, m), 5.47 (1H, d, *J*=1.5 Hz), 5.03 (1H, d, *J*=3.5 Hz, H-1'), 4.91 and 4.69 (2H, ABq, *J*=11.5 Hz), 4.78 and 4.52 (2H, ABq, *J*=10.5 Hz), 4.69 (1H, m), 4.62 and 4.52 (2H, ABq, *J*=12.0 Hz), 4.42 (1H, m), 4.36 (1H, m), 4.27 (1H, m), 4.19 (1H, m), 4.11 (1H, m), 3.83 (1H, m), 3.75-3.62 (6H, m), 3.12 (1H, d, *J*=11.5 Hz), 2.25 (1H, br-s); ^{13}C-NMR (125 MHz, $CDCl_3$) δ 138.0, 137.6, 137.4, 128.7, 128.5, 128.1, 128.0×2, 127.9×2, 102.8, 95.3, 82.1, 75.5, 75.0, 74.8, 73.7, 71.8, 71.3, 71.0, 70.6, 68.5, 68.1, 67.7, 60.2; HRMS (ESI-TOF) *m/z* 645.2313 (645.2312 calcd for $C_{34}H_{38}O_{11}Na$ $[M+Na]^+$).

Determination of glycosylation site of 20

Comparison of NMR data of α-D-glucosides **23**

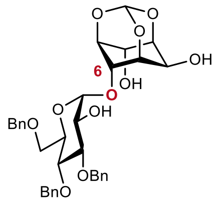

α-D-Glucoside **23**

Supplementary Table 4. Comparison of ^{1}H-NMR data of α-D-glucosides 23.

δ (Structurally defined α(1,6)-D-glucoside **23**) (500 MHz, $CDCl_3$)	δ (α-D-Glucoside **23** derived from **20**) (500 MHz, $CDCl_3$)
7.36-7.28 (m)	7.37-7.26 (m)
7.17-7.15 (m)	7.17-7.15 (m)
5.47 (d, *J*=1.0 Hz)	5.47 (d, *J*=1.5 Hz)
5.02 (d, *J*=3.5 Hz)	5.03 (d, *J*=3.5 Hz)
4.90 and 4.69 (ABq, *J*=11.0 Hz)	4.91 and 4.69 (ABq, *J*=11.5 Hz)
4.78 and 4.51 (ABq, *J*=11.0 Hz)	4.78 and 4.52 (ABq, *J*=10.5 Hz)
4.68 (m)	4.69 (m)
4.62 and 4.51 (ABq, *J*=12.0 Hz)	4.62 and 4.52 (ABq, *J*=12.0 Hz)
4.42 (m)	4.42 (m)
4.36 (m)	4.36 (m)
4.27 (m)	4.27 (m)
4.18 (m)	4.19 (m)
4.11 (m)	4.11 (m)
3.83 (m)	3.83 (m)
3.75-3.62 (m)	3.75-3.62 (m)
3.21 (d, *J*=11.0 Hz)	3.12 (d, *J*=11.5 Hz)
2.40 (br-s)	2.25 (br-s)

Supplementary Table 5. Comparison of ^{13}C-NMR data of α-D-glucosides 23.

δ (Structurally defined α(1,6)-D-glucoside **23**) (125 MHz, $CDCl_3$)	δ (α-D-Glucoside **23** derived from **20**) (125 MHz, $CDCl_3$)
138.1	138.0

137.6	137.6
137.5	137.4
128.7	128.7
128.5	128.5
128.1	128.1
128.0	128.0×2
127.9×3	127.9×2
102.8	102.8
95.4	95.3
82.1	82.1
75.5	75.5
75.0	75.0
74.7	74.8
73.7	73.7
71.8	71.8
71.2	71.3
71.0	71.0
70.6	70.6
68.5	68.5
68.1	68.1
67.7	67.7
60.2	60.2

Synthesis of α(1,6)-D-glucoside **21** from **23**.

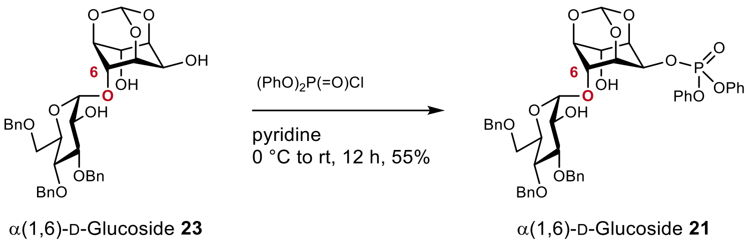

Supplementary Figure 10. Synthesis of α(1,6)-D-glucoside 21 from 23.

6-*O*-(3,4,6-Tri-*O*-benzyl-α-D-glucopyranosyl)-2-*O*-diphenylphosphoryl-D-*myo*-inositol-1,3,5-orthoformate (21)

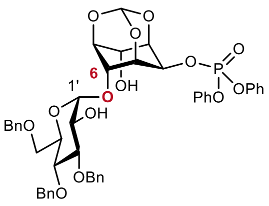

α(1,6)-D-Glucoside **21**

To a solution of **23** (22.4 mg, 36.0 μmol) in pyridine (720 μL) was added diphenyl phosphoryl chloride (8.9 μL, 43.2 μmol) at 0 ºC under Ar atmosphere. After the reaction mixture was stirred for 12 h at room temperature, the reaction was quenched by addition of H_2O (3 mL). The aqueous layer

was extracted with EtOAc (4 mL×3), and then the combined extracts were washed with brine (4 mL), dried over anhydrous Na_2SO_4, and concentrated in *vacuo*. Purification of the residue by silica gel column chromatography (3/1 PhMe/acetone) gave **21** (16.9 mg, 20.0 μmol, 55% yield).

Data for **21**: Colorless syrup; R_f 0.72 (1/1 PhMe/EtOAc); $[\alpha]^{24}_D$ +61.6° (*c* 1.59, $CHCl_3$); ^{1}H-NMR (500 MHz, $CDCl_3$) δ 7.37-7.19 (23H, m), 7.15-7.13 (2H, m), 5.54 (1H, d, *J*=1.0 Hz), 5.19 (1H, dd, *J*=1.0 Hz, *J*=8.0 Hz), 4.99 (1H, d, *J*=4.0 Hz, H-1'), 4.83 and 4.81 (2H, ABq, *J*=11.5 Hz), 4.80 and 4.48 (2H, ABq, *J*=11.0 Hz), 4.68 (1H, m), 4.60 and 4.50 (2H, ABq, *J*=12.0 Hz), 4.54 (1H, m), 4.44 (1H, m), 4.28 (2H, m), 3.82-3.59 (7H, m), 3.08 (1H, d, *J*=4.0 Hz); ^{13}C-NMR (125 MHz, $CDCl_3$) δ 150.1×2 (d, $^{31P\text{-}13C}J$=7.8 Hz), 138.3, 137.8, 137.6, 129.9×2, 128.5×2, 128.4, 127.9×3, 127.8, 125.8, 125.7, 120.3 (d, $^{31P\text{-}13C}J$=4.1 Hz), 120.1 (d, $^{31P\text{-}13C}J$=4.1 Hz), 102.6, 95.8, 81.9, 76.9, 75.5, 75.1, 73.6, 72.9 (d, $^{31P\text{-}13C}J$=6.5 Hz), 71.6, 70.7, 69.4, 68.5, 68.2, 68.1, 67.7 (d, $^{31P\text{-}13C}J$=4.8 Hz); ^{31}P-NMR (202 MHz, $CDCl_3$) δ −10.1; HRMS (ESI-TOF) 877.2571 (877.2601 calcd for $C_{46}H_{47}O_{14}NaP$ $[M+Na]^+$).

Determination of glycosylation site of 21

Comparison of NMR data of α-D-glucosides **21**

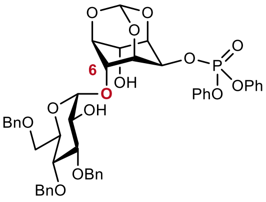

α(1,6)-D-Glucoside **21**

Supplementary Table 6. Comparison of ^{1}H-NMR data of α-D-glucosides 21.

δ (Structurally defined α(1,6)-D-glucoside **21**) (500 MHz, $CDCl_3$)	δ (α-D-Glucoside **21** derived from **14**) (500 MHz, $CDCl_3$)
7.37-7.19 (m)	7.37-7.19 (m)
7.15-7.13 (m)	7.15-7.13 (m)
5.54 (d, *J*=1.0 Hz)	5.54 (s)
5.19 (dd, *J*=1.0, 8.0 Hz)	5.19 (br-d, *J*=8.0 Hz)
4.99 (d, *J*=4.0 Hz)	4.99 (d, *J*=3.5 Hz)
4.83 and 4.81 (ABq, *J*=11.5 Hz)	4.83 and 4.81 (ABq, *J*=11.5 Hz)
4.80 and 4.48 (ABq, *J*=11.0 Hz)	4.80 and 4.48 (ABq, *J*=10.5 Hz)
4.68 (m)	4.68 (m)
4.60 and 4.50 (ABq, *J*=12.0 Hz)	4.60 and 4.50 (ABq, *J*=12.0 Hz)
4.54 (m)	4.54 (m)
4.44 (m)	4.44 (m)
4.28 (m)	4.28 (m)
3.82-3.59 (m)	3.82-3.59 (m)
3.08 (d, *J*=4.0 Hz)	3.05 (d, *J*=3.5 Hz)

Supplementary Table 7. Comparison of ^{13}C-NMR data of α-D-glucosides 21.

δ(Structurally defined α(1,6)-D-glucoside **21**) (125 MHz, $CDCl_3$)	δ(α-D-Glucoside **21** derived from **14**) (125 MHz, $CDCl_3$)
150.1×2 (d, ${}^{31P\text{-}13C}J$=7.8 Hz)	150.1×2 (d, ${}^{31P\text{-}13C}J$=7.8 Hz)
138.3	138.3
137.8	137.8
137.6	137.5
129.9×2	129.9×2
128.5×2	128.5
128.4	128.4×2
127.9×3	127.9×2
127.8	127.8×2
125.8	125.8
125.7	125.7
120.3 (d, ${}^{31P\text{-}13C}J$=4.1 Hz)	120.3 (d, ${}^{31P\text{-}13C}J$=4.8 Hz)
120.1 (d, ${}^{31P\text{-}13C}J$=4.1 Hz)	120.1 (d, ${}^{31P\text{-}13C}J$=4.8 Hz)
102.6	102.6
95.8	95.9
81.9	81.9
76.9	76.9
75.5	75.4
75.1	75.1
73.6	73.6
72.9 (d, ${}^{31P\text{-}13C}J$=6.5 Hz)	72.9 (d, ${}^{31P\text{-}13C}J$=6.6 Hz)
71.6	71.6
70.7	70.7
69.4	69.4
68.5	68.5
68.2	68.2
68.1	68.0
67.7 (d, ${}^{31P\text{-}13C}J$=4.8 Hz)	67.8 (d, ${}^{31P\text{-}13C}J$=4.8 Hz)

Synthesis of α(1,6)-D-glucoside **66** from **9**.

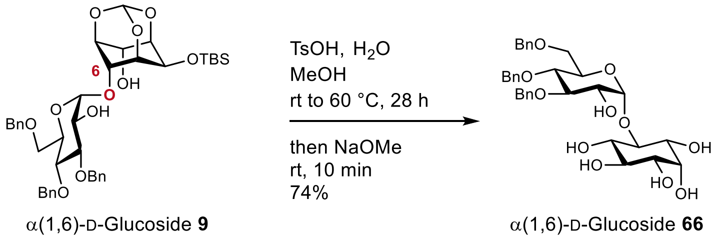

Supplementary Figure 11. Synthesis of α(1,6)-D-glucoside 66 from 9.

6-*O*-(3,4,6-Tri-*O*-benzyl-α-D-glucopyranosyl)-D-*myo*-inositol (66)

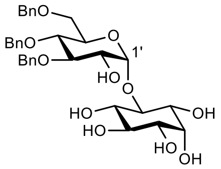

α(1,6)-D-Glucoside **66**

To a solution of **9** (16.2 mg, 22.0 μmol) and H_2O (198 μL, 11.0 mmol) in MeOH (880 μL) was added TsOH· H_2O (3.8 mg, 22.0 μmol) at room temperature under Ar atmosphere. After the reaction mixture was stirred for 28 h at 60 °C, the reaction mixture was cooled to room temperature. To the resultant mixture was added NaOMe (42.4 μL, 0.220 mmol, 28% in MeOH) at room temperature. After the reaction mixture was stirred for 10 min, the reaction mixture was dried over anhydrous Na_2SO_4, and concentrated in *vacuo*. Purification of the residue by silica gel column chromatography (6/1 $CHCl_3$/MeOH) gave **66** (10.0 mg, 16.3 μmol, 74% yield).

Data for **66**: Colorless syrup; R_f 0.44 (4/1 $CHCl_3$/MeOH); $[\alpha]^{24}_D$ +74.0° (*c* 1.0, MeOH); ^{1}H-NMR (500 MHz, $CDCl_3$) δ 7.38-7.22 (13H, m), 7.14-7.12 (2H, m), 5.13 (1H, d, *J*=4.0 Hz, H-1'), 4.95 and 4.78 (2H, ABq, *J*=11.5 Hz), 4.74 and 4.50 (2H, ABq, *J*=11.5 Hz), 4.58 and 4.49 (2H, ABq, *J*=12.5 Hz), 4.16 (1H, m), 3.98 (1H, dd, *J*=2.5 Hz, *J*=2.5 Hz), 3.81 (1H, dd, *J*=9.5 Hz, *J*=9.5 Hz), 3.73 (1H, dd, *J*=9.5 Hz, *J*=9.5 Hz), 3.68-3.61 (5H, m), 3.52 (1H, dd, *J*=9.5 Hz, *J*=10.0 Hz), 3.37 (1H, dd, *J*=2.5 Hz, *J*=9.5 Hz), 3.29 (1H, dd, *J*=9.5 Hz, *J*=9.5 Hz); ^{13}C-NMR (125 MHz, $CDCl_3$) δ 140.3, 139.7, 139.3, 129.4, 129.3×3, 128.9, 128.8, 128.7, 128.5, 102.1, 84.7, 84.1, 78.9, 76.4, 75.9, 75.0, 74.5×2, 74.4, 73.7, 73.4, 73.3, 72.1, 69.8; HRMS (ESI-TOF) *m/z* 635.2438 (635.2468 calcd for $C_{33}H_{40}O_{11}Na$ $[M+Na]^+$).

Synthesis of α-D-glucoside **66** from **22**.

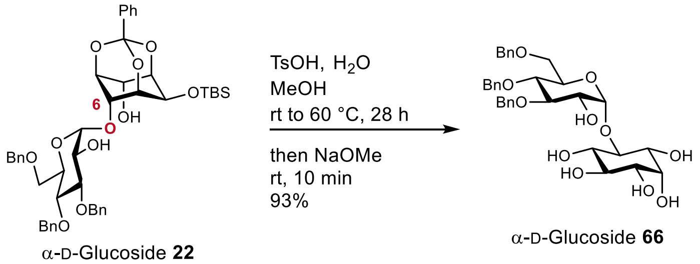

Supplementary Figure 12. Synthesis of α-D-glucoside 66 from 22.

6-*O*-(3,4,6-Tri-*O*-benzyl-α-D-glucopyranosyl)-D-*myo*-inositol (66)

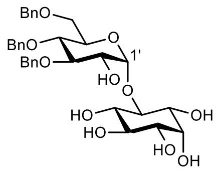

α-D-Glucoside **66**

To a solution of **22** (22.0 mg, 27.1 μmol) and H_2O (244 μL, 13.5 mmol) in MeOH (1.08 mL) was added TsOH· H_2O (4.7 mg, 27.1 μmol) at room temperature under Ar atmosphere. After the reaction mixture was stirred for 28 h at 60 °C, the reaction mixture was cooled to room temperature. To the resultant mixture was added NaOMe (52.2 μL, 0.271 mmol, 28% in MeOH) at room temperature. After the reaction mixture was stirred for 10 min, the reaction mixture was dried over anhydrous Na_2SO_4, and concentrated in *vacuo*. Purification of the residue by silica gel column chromatography (6/1 $CHCl_3$/MeOH) gave **66** (15.5 mg, 25.3 μmol, 93% yield).

Data for **66**: Colorless syrup; R_f 0.44 (4/1 $CHCl_3$/MeOH); $[\alpha]^{24}_D$ +71.5° (*c* 1.0, MeOH); ^{1}H-NMR (500 MHz, $CDCl_3$) δ 7.38-7.22 (13H, m), 7.14-7.12 (2H, m), 5.13 (1H, d, *J*=4.0 Hz, H-1'), 4.95 and 4.78 (2H, ABq, *J*=11.5 Hz), 4.74 and 4.50 (2H, ABq, *J*=11.5 Hz), 4.58 and 4.49 (2H, ABq, *J*=12.5 Hz), 4.16 (1H, m), 3.99 (1H, dd, *J*=2.5 Hz, *J*=2.5 Hz), 3.81 (1H, dd, *J*=9.5 Hz, *J*=9.5 Hz), 3.73 (1H, dd, *J*=9.5 Hz, *J*=9.5 Hz), 3.68-3.61 (5H, m), 3.52 (1H, dd, *J*=9.5 Hz, *J*=10.0 Hz), 3.37 (1H, dd, *J*=2.5 Hz, *J*=9.5 Hz), 3.29 (1H, dd, *J*=9.5 Hz, *J*=9.5 Hz); ^{13}C-NMR (125 MHz, $CDCl_3$) δ 140.3, 139.7, 139.3, 129.4, 129.3×3, 128.9, 128.8, 128.7, 128.5, 102.1, 84.7, 84.1, 78.9, 76.4, 75.9, 75.0, 74.5×2, 74.4, 73.7, 73.4, 73.2, 72.0, 69.8; HRMS (ESI-TOF) *m/z* 635.2452 (635.2468 calcd for $C_{33}H_{40}O_{11}Na$ $[M+Na]^+$).

Determination of glycosylation site of 22

Comparison of NMR data of α-D-glucosides **66**

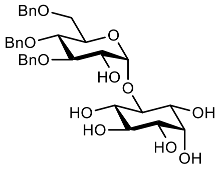

α(1,6)-D-Glucoside **66**

Supplementary Table 8. Comparison of ^{1}H-NMR data of α-D-glucosides 66.

δ (Structurally defined α(1,6)-D-glucoside **66**) (500 MHz, $CDCl_3$)	δ (α-D-Glucoside **66** derived from **22**) (500 MHz, $CDCl_3$)
7.38-7.22 (m)	7.38-7.22 (m)
7.14-7.12 (m)	7.14-7.12 (m)

5.13 (d, *J*=4.0 Hz)	5.13 (d, *J*=4.0 Hz)
4.95 and 4.78 (ABq, *J*=11.5 Hz)	4.95 and 4.78 (ABq, *J*=11.5 Hz)
4.74 and 4.50 (ABq, *J*=11.5 Hz)	4.74 and 4.50 (ABq, *J*=11.5 Hz)
4.58 and 4.49 (ABq, *J*=12.5 Hz)	4.58 and 4.49 (ABq, *J*=12.5 Hz)
4.16 (m)	4.16 (m)
3.98 (dd, *J*=2.5, 2.5 Hz)	3.99 (dd, *J*=2.5, 2.5 Hz)
3.81 (dd, *J*=9.5, 9.5 Hz)	3.81 (dd, *J*=9.5, 9.5 Hz)
3.73 (dd, *J*=9.5, 9.5 Hz)	3.73 (dd, *J*=9.5, 9.5 Hz)
3.68-3.61 (m)	3.68-3.61 (m)
3.52 (dd, *J*=9.5, 10.0 Hz)	3.52 (dd, *J*=9.5, 10.0 Hz)
3.37 (dd, *J*=2.5, 9.5 Hz)	3.37 (dd, *J*=2.5, 9.5 Hz)
3.29 (dd, *J*=9.5, 9.5 Hz)	3.29 (dd, *J*=9.5, 9.5 Hz)

Supplementary Table 9. Comparison of ^{13}C-NMR data of α-D-glucosides 66.

δ (Structurally defined α(1,6)-D-glucoside **66**) (125 MHz, $CDCl_3$)	δ (α-D-Glucoside **66** derived from **22**) (125 MHz, $CDCl_3$)
140.3	140.3
139.7	139.7
139.3	139.3
129.4	129.4
129.3×3	129.3×3
128.9	128.9
128.8	128.8
128.7	128.7
128.5	128.5
102.1	102.1
84.7	84.7
84.1	84.1
78.9	78.9
76.4	76.4
75.9	75.9
75.0	75.0
74.5×2	74.5×2
74.4	74.4
73.7	73.7
73.4	73.4
73.2	73.2
72.1	72.0
69.8	69.8

Determination of glycosylation site of 23 from 16

Comparison of NMR data of α-D-glucosides **23**

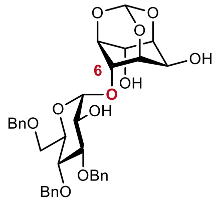

α-D-Glucoside **23**

Supplementary Table 10. Comparison of ^{1}H-NMR data of α-D-glucosides 23.

δ(Structurally defined α(1,6)-D-glucoside **23**) (500 MHz, $CDCl_3$)	δ(α-D-Glucoside **23** derived from **16**) (500 MHz, $CDCl_3$)
7.36-7.28 (m)	7.37-7.26 (m)
7.17-7.15 (m)	7.17-7.15 (m)
5.47 (d, *J*=1.0 Hz)	5.47 (d, *J*=1.0 Hz)
5.02 (d, *J*=3.5 Hz)	5.03 (d, *J*=3.5 Hz)
4.90 and 4.69 (ABq, *J*=11.0 Hz)	4.90 and 4.69 (ABq, *J*=11.5 Hz)
4.78 and 4.51 (ABq, *J*=11.0 Hz)	4.78 and 4.52 (ABq, *J*=10.5 Hz)
4.68 (m)	4.68 (m)
4.62 and 4.51 (ABq, *J*=12.0 Hz)	4.62 and 4.51 (ABq, *J*=12.0 Hz)
4.42 (m)	4.43 (m)
4.36 (m)	4.36 (m)
4.27 (m)	4.27 (m)
4.18 (m)	4.18 (m)
4.11 (m)	4.11 (m)
3.83 (m)	3.83 (m)
3.75-3.62 (m)	3.75-3.62 (m)
3.21 (d, *J*=11.0 Hz)	3.17 (d, *J*=11.5 Hz)
2.40 (br-s)	2.36 (br-s)

Supplementary Table 11. Comparison of ^{13}C-NMR data of α-D-glucosides 23.

δ(Structurally defined α(1,6)-D-glucoside **23**) (125 MHz, $CDCl_3$)	δ(α-D-Glucoside **23** derived from **16**) (125 MHz, $CDCl_3$)
138.1	138.0
137.6	137.6
137.5	137.5
128.7	128.7
128.5	128.5
128.1	128.1
128.0	128.0
127.9×3	127.9×3
102.8	102.8
95.4	95.4
82.1	82.1
75.5	75.5
75.0	75.0
74.7	74.7

73.7	73.7
71.8	71.8
71.2	71.3
71.0	71.0
70.6	70.6
68.5	68.5
68.1	68.1
67.7	67.7
60.2	60.2

Synthesis of acetylated α-D-glucoside **65** from **24** synthesized by our desymmetric glycosylation of *meso*-diol **8**.

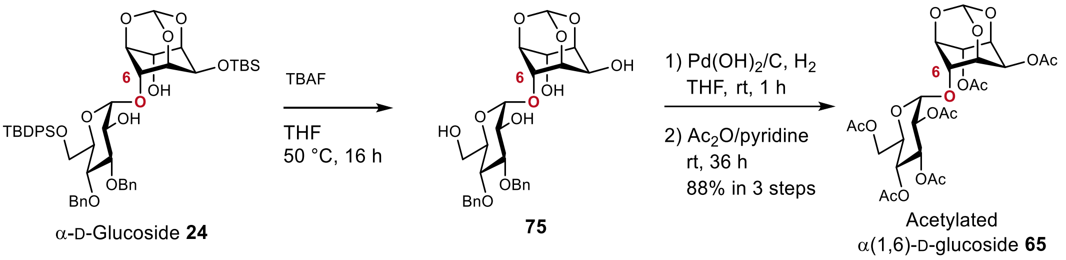

Supplementary Figure 13. Synthesis of acetylated α-D-glucoside 65 from 24.

2,4-Di-*O*-acetyl-6-*O*-(2,3,4,6-tetra-*O*-acetyl-α-D-glucopyranosyl)-D-*myo*-inositol-1,3,5-orthoformate (65)

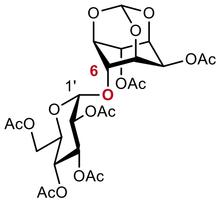

To a solution of **24** (28.1 mg, 31.7 μmol) in THF (634 μL) was added TBAF (127 μL, 127 μmol, 1.0 M in THF) at room temperature. After the reaction mixture was stirred for 16 h at 50 °C, the reaction mixture was concentrated in *vacuo*. The residue was subjected to silica gel column chromatography (1/1 PhMe/acetone) to give crude **75** (16.2 mg).

To a solution of crude **75** (16.2 mg) in THF (3.2 mL) was added 20% $Pd(OH)_2/C$ (16.2 mg, wetted with 50% water) at room temperature under Ar atmosphere. After changing the atmosphere to H_2 (balloon), the reaction mixture was stirred for 1 h. After changing the atmosphere to Ar, the reaction was filtered through celite pad, and the filtrate was concentrated in *vacuo*. To a solution of the residue in pyridine (800 μL) was added Ac_2O (800 μL) at room temperature. After the reaction mixture was stirred for 36 h at room temperature, the reaction was quenched by addition of H_2O (5 mL). The resultant mixture was extracted with EtOAc (5 mL×3), and then the extracts were washed

with brine (5 mL), dried over anhydrous Na_2SO_4, and concentrated in *vacuo*. The residue was subjected to silica gel column chromatography (2/1 *n*-hexane/EtOAc) to give **65** (16.8 mg, 27.8 μmol, 88% yield in 3 steps).

Data for **65**: Colorless syrup; R_f 0.34 (1/1 *n*-hexane/EtOAc); $[\alpha]^{23}_D$ +100.9° (*c* 1.68, $CHCl_3$); ^{1}H-NMR (500 MHz, $CDCl_3$) δ 5.58 (1H, d, *J*=1.0 Hz), 5.51 (1H, m), 5.36 (1H, dd, *J*=10.0 Hz, *J*=10.5 Hz), 5.24 (1H, d, *J*=4.0 Hz, H-1'), 5.17 (1H, m), 5.05 (1H, dd, *J*=9.5 Hz, *J*=10.0 Hz), 4.90 (1H, dd, *J*=4.0 Hz, *J*=10.5 Hz), 4.60 (1H, m), 4.52 (1H, m), 4.33 (1H, m), 4.30 (1H, m), 4.25 (1H, dd, *J*=5.0 Hz, *J*=12.5 Hz), 4.10 (1H, dd, *J*=2.0 Hz, *J*=12.5 Hz), 3.97 (1H, m), 2.27 (3H, s), 2.20 (3H, s), 2.11 (6H, s), 2.03 (3H, s), 2.01 (3H, s); ^{13}C-NMR (125 MHz, $CDCl_3$) δ 170.5, 170.3, 170.2×2, 170.0, 169.4, 103.0, 94.5, 70.6, 70.1, 69.6, 69.3, 68.5, 68.2, 67.9×2, 67.8, 62.9, 61.8, 21.0, 20.8, 20.7, 20.6, 20.5×2; HRMS (ESI-TOF) 627.1556 (627.1537 calcd for $C_{25}H_{32}O_{17}Na$ $[M+Na]^+$).

Determination of glycosylation site of 24

Comparison of NMR data of acetylated α-D-glucosides **64** and **65**

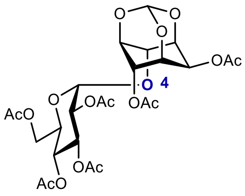

Acetylated
α(1,4)-D-glucoside **64**

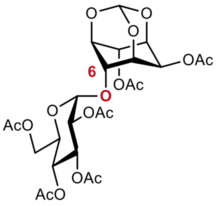

Acetylated
α(1,6)-D-glucoside **65**

Supplementary Table 12. Comparison of ^{1}H-NMR data of acetylated α-D-glucosides 64 and 65.

δ (Structurally defined acetylated α(1,4)-D-glucoside **64**) (500 MHz, $CDCl_3$)	δ (Structurally defined acetylated α (1,6)-D-glucoside **65**) (500 MHz, $CDCl_3$)	δ (Acetylated α-D-glucoside **65** derived from **24**) (500 MHz, $CDCl_3$)
5.55 (d, *J*=1.5 Hz)	5.58 (d, *J*=1.0 Hz)	5.58 (d, *J*=1.0 Hz)
5.38 (m)	5.50 (m)	5.51 (m)
5.36 (dd, *J*=10.0, 10.5 Hz)	5.36 (dd, *J*=10.0, 10.5 Hz)	5.36 (dd, *J*=10.0, 10.5 Hz)
5.26 (d, *J*=4.0 Hz)	5.24 (d, *J*=4.0 Hz)	5.24 (d, *J*=4.0 Hz)
5.23 (m)	5.17 (m)	5.17 (m)
5.10 (dd, *J*=10.0, 10.0 Hz)	5.05 (dd, *J*=9.5, 10.0 Hz)	5.05 (dd, *J*=9.5, 10.0 Hz)
4.82 (dd, *J*=4.0, 10.5 Hz)	4.90 (dd, *J*=4.0, 10.5 Hz)	4.90 (dd, *J*=4.0, 10.5 Hz)
4.55 (m)	4.60 (m)	4.60 (m)
4.42-4.30 (m)	4.52 (m)	4.52 (m)
	4.33 (m)	4.33 (m)
	4.30 (m)	4.30 (m)
	4.24 (dd, *J*=5.0, 12.5 Hz)	4.25 (dd, *J*=5.0, 12.5 Hz)

	4.10 (dd, *J*=2.0, 12.5 Hz)	4.10 (dd, *J*=2.0, 12.5 Hz)
4.06 (m)	3.97 (m)	3.97 (m)
2.22 (s)	2.27 (s)	2.27 (s)
2.21 (s)	2.20 (s)	2.20 (s)
2.09 (s)	2.11×2 (s)	2.11×2 (s)
2.06×2 (s)		
	2.03 (s)	2.03 (s)
2.02 (s)	2.01 (s)	2.01 (s)

Supplementary Table 13. Comparison of ^{13}C-NMR data of acetylated α-D-glucosides 64 and 65.

δ(Structurally defined acetylated α(1,4)-D-glucoside **64**) (125 MHz, $CDCl_3$)	δ(Structurally defined acetylated α(1,6)-D-glucoside **65**) (125 MHz, $CDCl_3$)	δ(Acetylated α-D-glucoside **65** derived from **24**) (125 MHz, $CDCl_3$)
170.5×2	170.5	170.5
170.4	170.3	170.3
170.0	170.2×2	170.2×2
169.9	170.0	170.0
169.5	169.4	169.4
102.9	103.0	103.0
99.6	94.5	94.5
77.1	70.6	70.6
70.7	70.1	70.1
70.6	69.7	69.6
69.6	69.4	69.3
69.4	68.6	68.5
68.0×2	68.3	68.2
	68.0	67.9×2
67.8	67.9	
66.9	67.8	67.8
63.6	62.9	62.9
61.7	61.8	61.8
21.0	21.0	21.0
20.9	20.8	20.8
20.7	20.7	20.7
20.6×2	20.6	20.6
20.4	20.5×2	20.5×2

Synthesis of acetylated α-D-glucoside **65** from **25** synthesized by our desymmetric glycosylation of *meso*-diol **8**.

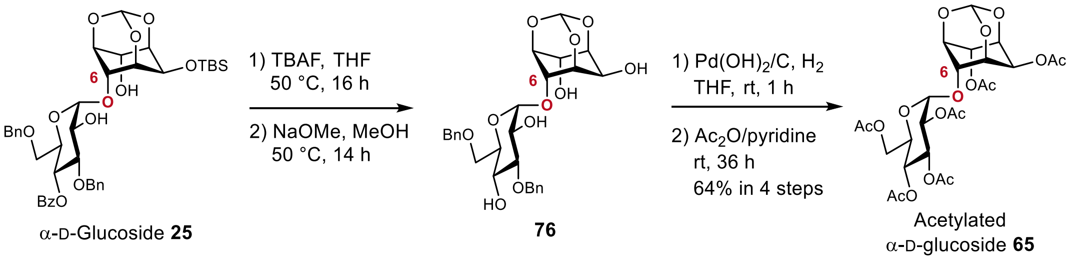

Supplementary Figure 14. Synthesis of acetylated α-D-glucoside 65 from 25.

2,4-Di-*O*-acetyl-6-*O*-(2,3,4,6-tetra-*O*-acetyl-α-D-glucopyranosyl)-D-*myo*-inositol-1,3,5-orthoformate (65)

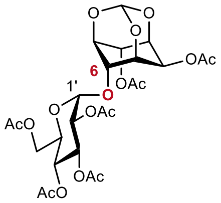

Acetylated
α-D-glucoside **65**

To a solution of **25** (26.3 mg, 35.0 μmol) in THF (700 μL) was added TBAF (70 μL, 70 μmol, 1.0 M in THF) at room temperature. After the reaction mixture was stirred for 16 h at 50 °C, the reaction mixture was concentrated in *vacuo*. To a solution of the residue in MeOH (700 μL) was added NaOMe (67.5 μL, 350 μmol, 28% in MeOH) at room temperature. After the reaction mixture was stirred for 14 h at 50 °C, the reaction was quenched by addition of sat. NH_4Cl aq. (5 mL). The resultant mixture was extracted with EtOAc (5 mL×6), and then the extracts were washed with brine (5 mL), dried over anhydrous Na_2SO_4, and concentrated in *vacuo*. The residue was subjected to silica gel column chromatography (2/1 PhMe/acetone) to give crude **76** (17.1 mg).

To a solution of crude **76** (17.1 mg) in THF (3.5 mL) was added 20% $Pd(OH)_2/C$ (17.1 mg, wetted with 50% water) at room temperature under Ar atmosphere. After changing the atmosphere to H_2 (balloon), the reaction mixture was stirred for 1 h. After changing the atmosphere to Ar, the reaction was filtered through celite pad, and the filtrate was concentrated in *vacuo*. To a solution of the residue in pyridine (1.75 mL) was added Ac_2O (1.75 mL) at room temperature. After the reaction mixture was stirred for 36 h at room temperature, the reaction was quenched by addition of H_2O (5 mL). The resultant mixture was extracted with EtOAc (5 mL×3), and then the extracts were washed with brine (5 mL), dried over anhydrous Na_2SO_4, and concentrated in *vacuo*. The residue was subjected to silica gel column chromatography (2/1 *n*-hexane/EtOAc) to give **65** (13.5 mg, 22.3 μmol, 64% yield in 4 steps).

Data for **65**: Colorless syrup; R_f 0.34 (1/1 *n*-hexane/EtOAc); $[\alpha]^{23}_D$ +102.8° (*c* 1.35, $CHCl_3$); ^{1}H-NMR (500 MHz, $CDCl_3$) δ 5.58 (1H, d, *J*=1.0 Hz), 5.51 (1H, m), 5.36 (1H, dd, *J*=10.0 Hz, *J*=10.5 Hz), 5.24 (1H, d, *J*=4.0 Hz, H-1'), 5.17 (1H, m), 5.05 (1H, dd, *J*=9.5 Hz, *J*=10.0 Hz), 4.90 (1H, dd, *J*=4.0 Hz, *J*=10.5 Hz), 4.60 (1H, m), 4.52 (1H, m), 4.33 (1H, m), 4.30 (1H, m), 4.25 (1H, dd, *J*=5.0 Hz, *J*=12.5 Hz), 4.10 (1H, dd, *J*=2.0 Hz, *J*=12.5 Hz), 3.97 (1H, m), 2.27 (3H, s), 2.20 (3H, s), 2.11 (6H, s), 2.03 (3H, s), 2.01 (3H, s); ^{13}C-NMR (125 MHz, $CDCl_3$) δ 170.5, 170.3, 170.2×2, 170.0, 169.4, 103.0, 94.5, 70.6, 70.1, 69.6, 69.3, 68.5, 68.2, 67.9×2, 67.8, 62.9, 61.8, 21.0, 20.8, 20.7, 20.6, 20.5×2; HRMS (ESI-TOF) 627.1545 (627.1537 calcd for $C_{25}H_{32}O_{17}Na$ $[M+Na]^+$).

Determination of glycosylation site of 25

Comparison of NMR data of acetylated α-D-glucosides **64** and **65**

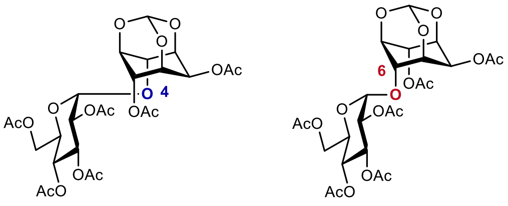

Acetylated α(1,4)-D-glucoside **64**   Acetylated α(1,6)-D-glucoside **65**

Supplementary Table 14. Comparison of ¹H-NMR data of acetylated α-D-glucosides 64 and 65.

<table>
<tr><th>δ (Structurally defined acetylated α(1,4)-D-glucoside 64)
(500 MHz, CDCl₃)</th><th>δ (Structurally defined acetylated α(1,6)-D-glucoside 65)
(500 MHz, CDCl₃)</th><th>δ (Acetylated α-D-glucoside 65 derived from 25)
(500 MHz, CDCl₃)</th></tr>
<tr><td>5.55 (d, J=1.5 Hz)</td><td>5.58 (d, J=1.0 Hz)</td><td>5.58 (d, J=1.0 Hz)</td></tr>
<tr><td>5.38 (m)</td><td>5.50 (m)</td><td>5.51 (m)</td></tr>
<tr><td>5.36 (dd, J=10.0, 10.5 Hz)</td><td>5.36 (dd, J=10.0, 10.5 Hz)</td><td>5.36 (dd, J=10.0, 10.5 Hz)</td></tr>
<tr><td>5.26 (d, J=4.0 Hz)</td><td>5.24 (d, J=4.0 Hz)</td><td>5.24 (d, J=4.0 Hz)</td></tr>
<tr><td>5.23 (m)</td><td>5.17 (m)</td><td>5.17 (m)</td></tr>
<tr><td>5.10 (dd, J=10.0, 10.0 Hz)</td><td>5.05 (dd, J=9.5, 10.0 Hz)</td><td>5.05 (dd, J=9.5, 10.0 Hz)</td></tr>
<tr><td>4.82 (dd, J=4.0, 10.5 Hz)</td><td>4.90 (dd, J=4.0, 10.5 Hz)</td><td>4.90 (dd, J=4.0, 10.5 Hz)</td></tr>
<tr><td>4.55 (m)</td><td>4.60 (m)</td><td>4.60 (m)</td></tr>
<tr><td rowspan="5">4.42-4.30 (m)</td><td>4.52 (m)</td><td>4.52 (m)</td></tr>
<tr><td>4.33 (m)</td><td>4.33 (m)</td></tr>
<tr><td>4.30 (m)</td><td>4.30 (m)</td></tr>
<tr><td>4.24 (dd, J=5.0, 12.5 Hz)</td><td>4.25 (dd, J=5.0, 12.5 Hz)</td></tr>
<tr><td>4.10 (dd, J=2.0, 12.5 Hz)</td><td>4.10 (dd, J=2.0, 12.5 Hz)</td></tr>
<tr><td>4.06 (m)</td><td>3.97 (m)</td><td>3.97 (m)</td></tr>
<tr><td>2.22 (s)</td><td>2.27 (s)</td><td>2.27 (s)</td></tr>
<tr><td>2.21 (s)</td><td>2.20 (s)</td><td>2.20 (s)</td></tr>
<tr><td>2.09 (s)</td><td rowspan="2">2.11×2 (s)</td><td rowspan="2">2.11×2 (s)</td></tr>
<tr><td rowspan="2">2.06×2 (s)</td></tr>
<tr><td>2.03 (s)</td><td>2.03 (s)</td></tr>
<tr><td>2.02 (s)</td><td>2.01 (s)</td><td>2.01 (s)</td></tr>
</table>

Supplementary Table 15. Comparison of ¹³C-NMR data of acetylated α-D-glucosides 64 and 65.

δ (Structurally defined acetylated α(1,4)-D-glucoside **64**) (125 MHz, CDCl₃)	δ (Structurally defined acetylated α(1,6)-D-glucoside **65**) (125 MHz, CDCl₃)	δ (Acetylated α-D-glucoside **65** derived from **25**) (125 MHz, CDCl₃)
170.5×2	170.5	170.5
170.4	170.3	170.3
170.0	170.2×2	170.2×2
169.9	170.0	170.0
169.5	169.4	169.4
102.9	103.0	103.0

99.6	94.5	94.5
77.1	70.6	70.6
70.7	70.1	70.1
70.6	69.7	69.6
69.6	69.4	69.3
69.4	68.6	68.5
68.0×2	68.3	68.2
	68.0	67.9×2
67.8	67.9	
66.9	67.8	67.8
63.6	62.9	62.9
61.7	61.8	61.8
21.0	21.0	21.0
20.9	20.8	20.8
20.7	20.7	20.7
20.6×2	20.6	20.6
20.4	20.5×2	20.5×2

Synthesis of acetylated α-D-glucoside **65** from **26** synthesized by our desymmetric glycosylation of *meso*-diol **8**.

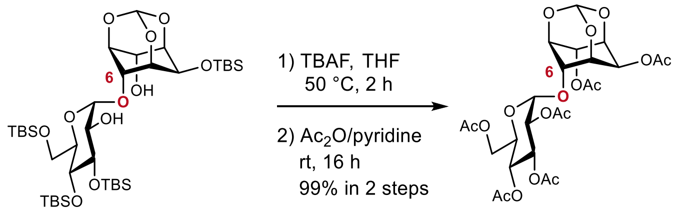

α-D-Glucoside **26**

Acetylated α(1,6)-D-glucoside **65**

Supplementary Figure 15. Synthesis of acetylated α-D-glucoside 65 from 26.

2,4-Di-*O*-acetyl-6-*O*-(2,3,4,6-tetra-*O*-acetyl-α-D-glucopyranosyl)-D-*myo*-inositol-1,3,5-orthoformate (65)

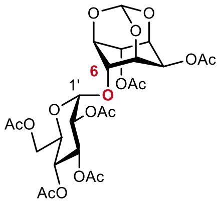

Acetylated α-D-glucoside **65**

To a solution of **26** (18.5 mg, 22.9 μmol) in THF (2.3 mL) was added TBAF (274 μL, 274 μmol, 1.0 M in THF) at room temperature. After the reaction mixture was stirred for 2 h at 50 °C, the reaction mixture was concentrated in *vacuo*. To a solution of the residue in pyridine (600 μL) was added Ac_2O (600 μL) at room temperature. After the reaction mixture was stirred for 16 h at room temperature, the reaction was quenched by addition of H_2O (5 mL). The resultant mixture was

extracted with EtOAc (5 mL×3), and then the extracts were washed with brine (5 mL), dried over anhydrous Na_2SO_4, and concentrated in *vacuo*. The residue was subjected to silica gel column chromatography (4/1 *n*-hexane/acetone) to give **65** (13.8 mg, 22.8 μmol, 99% yield in 2 steps).

Data for **65**: Colorless syrup; R_f 0.34 (1/1 *n*-hexane/EtOAc); $[\alpha]^{24}_{D}$ +107.2° (*c* 1.38, $CHCl_3$); ^{1}H-NMR (500 MHz, $CDCl_3$) δ 5.58 (1H, d, *J*=1.0 Hz), 5.51 (1H, m), 5.36 (1H, dd, *J*=10.0 Hz, *J*=10.5 Hz), 5.24 (1H, d, *J*=4.0 Hz, H-1'), 5.17 (1H, m), 5.05 (1H, dd, *J*=9.5 Hz, *J*=10.0 Hz), 4.90 (1H, dd, *J*=4.0 Hz, *J*=10.5 Hz), 4.60 (1H, m), 4.52 (1H, m), 4.33 (1H, m), 4.30 (1H, m), 4.25 (1H, dd, *J*=5.0 Hz, *J*=12.5 Hz), 4.10 (1H, dd, *J*=2.0 Hz, *J*=12.5 Hz), 3.97 (1H, m), 2.27 (3H, s), 2.20 (3H, s), 2.11 (6H, s), 2.03 (3H, s), 2.01 (3H, s); ^{13}C-NMR (125 MHz, $CDCl_3$) δ 170.5, 170.3, 170.2×2, 170.0, 169.4, 103.0, 94.5, 70.6, 70.1, 69.6, 69.4, 68.5, 68.2, 67.9×2, 67.8, 62.9, 61.8, 21.0, 20.8, 20.7, 20.6, 20.5×2; HRMS (ESI-TOF) 627.1533 (627.1537 calcd for $C_{25}H_{32}O_{17}Na$ $[M+Na]^+$).

Determination of glycosylation site of 26

Comparison of ^{1}H-NMR data of acetylated α-D-glucosides **64** and **65**

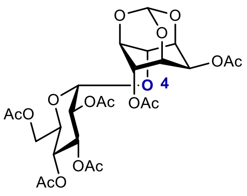

Acetylated
α(1,4)-D-glucoside **64**

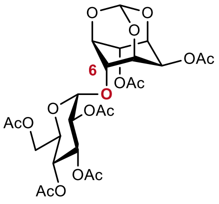

Acetylated
α(1,6)-D-glucoside **65**

Supplementary Table 16. Comparison of ^{1}H-NMR data of acetylated α-D-glucosides 64 and 65.

δ (Structurally defined acetylated α(1,4)-D-glucoside **64**) (500 MHz, $CDCl_3$)	δ (Structurally defined acetylated α(1,6)-D-glucoside **65**) (500 MHz, $CDCl_3$)	δ (Acetylated α-D-glucoside **65** derived from **26**) (500 MHz, $CDCl_3$)
5.55 (d, *J*=1.5 Hz)	5.58 (d, *J*=1.0 Hz)	5.58 (d, *J*=1.0 Hz)
5.38 (m)	5.50 (m)	5.51 (m)
5.36 (dd, *J*=10.0, 10.5 Hz)	5.36 (dd, *J*=10.0, 10.5 Hz)	5.36 (dd, *J*=10.0, 10.5 Hz)
5.26 (d, *J*=4.0 Hz)	5.24 (d, *J*=4.0 Hz)	5.24 (d, *J*=4.0 Hz)
5.23 (m)	5.17 (m)	5.17 (m)
5.10 (dd, *J*=10.0, 10.0 Hz)	5.05 (dd, *J*=9.5, 10.0 Hz)	5.05 (dd, *J*=9.5, 10.0 Hz)
4.82 (dd, *J*=4.0, 10.5 Hz)	4.90 (dd, *J*=4.0, 10.5 Hz)	4.90 (dd, *J*=4.0, 10.5 Hz)
4.55 (m)	4.60 (m)	4.60 (m)
4.42-4.30 (m)	4.52 (m)	4.52 (m)
	4.33 (m)	4.33 (m)
	4.30 (m)	4.30 (m)
	4.24 (dd, *J*=5.0, 12.5 Hz)	4.25 (dd, *J*=5.0, 12.5 Hz)

<table>
<tr><td></td><td>4.10 (dd, J=2.0, 12.5 Hz)</td><td>4.10 (dd, J=2.0, 12.5 Hz)</td></tr>
<tr><td>4.06 (m)</td><td>3.97 (m)</td><td>3.97 (m)</td></tr>
<tr><td>2.22 (s)</td><td>2.27 (s)</td><td>2.27 (s)</td></tr>
<tr><td>2.21 (s)</td><td>2.20 (s)</td><td>2.20 (s)</td></tr>
<tr><td>2.09 (s)</td><td rowspan="2">2.11×2 (s)</td><td rowspan="2">2.11×2 (s)</td></tr>
<tr><td rowspan="2">2.06×2 (s)</td></tr>
<tr><td>2.03 (s)</td><td>2.03 (s)</td></tr>
<tr><td>2.02 (s)</td><td>2.01 (s)</td><td>2.01 (s)</td></tr>
</table>

Supplementary Table 17. Comparison of ^{13}C-NMR data of acetylated α-D-glucosides 64 and 65.

<table>
<tr><th>δ (Structurally defined acetylated α(1,4)-D-glucoside 64) (125 MHz, $CDCl_3$)</th><th>δ (Structurally defined acetylated α(1,6)-D-glucoside 65) (125 MHz, $CDCl_3$)</th><th>δ (Acetylated α-D-glucoside 65 derived from 26) (125 MHz, $CDCl_3$)</th></tr>
<tr><td>170.5×2</td><td>170.5</td><td>170.5</td></tr>
<tr><td>170.4</td><td>170.3</td><td>170.3</td></tr>
<tr><td>170.0</td><td>170.2×2</td><td>170.2×2</td></tr>
<tr><td>169.9</td><td>170.0</td><td>170.0</td></tr>
<tr><td>169.5</td><td>169.4</td><td>169.4</td></tr>
<tr><td>102.9</td><td>103.0</td><td>103.0</td></tr>
<tr><td>99.6</td><td>94.5</td><td>94.5</td></tr>
<tr><td>77.1</td><td>70.6</td><td>70.6</td></tr>
<tr><td>70.7</td><td>70.1</td><td>70.1</td></tr>
<tr><td>70.6</td><td>69.7</td><td>69.6</td></tr>
<tr><td>69.6</td><td>69.4</td><td>69.4</td></tr>
<tr><td>69.4</td><td>68.6</td><td>68.5</td></tr>
<tr><td rowspan="2">68.0×2</td><td>68.3</td><td>68.2</td></tr>
<tr><td>68.0</td><td rowspan="2">67.9×2</td></tr>
<tr><td>67.8</td><td>67.9</td></tr>
<tr><td>66.9</td><td>67.8</td><td>67.8</td></tr>
<tr><td>63.6</td><td>62.9</td><td>62.9</td></tr>
<tr><td>61.7</td><td>61.8</td><td>61.8</td></tr>
<tr><td>21.0</td><td>21.0</td><td>21.0</td></tr>
<tr><td>20.9</td><td>20.8</td><td>20.8</td></tr>
<tr><td>20.7</td><td>20.7</td><td>20.7</td></tr>
<tr><td>20.6×2</td><td>20.6</td><td>20.6</td></tr>
<tr><td>20.4</td><td>20.5×2</td><td>20.5×2</td></tr>
</table>

Determination of glycosylation site of α(1,6)-D-galactoside 40.

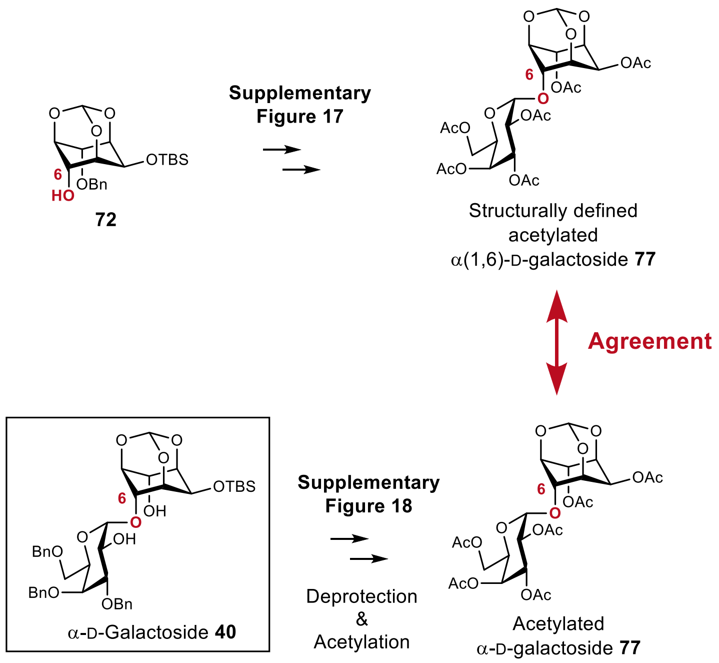

Supplementary Figure 16. Determination of glycosylation site of α-D-galactoside 40.

<u>Synthesis of acetylated α(1,6)-D-galactoside **77** from optically pure inositol **72**.</u>

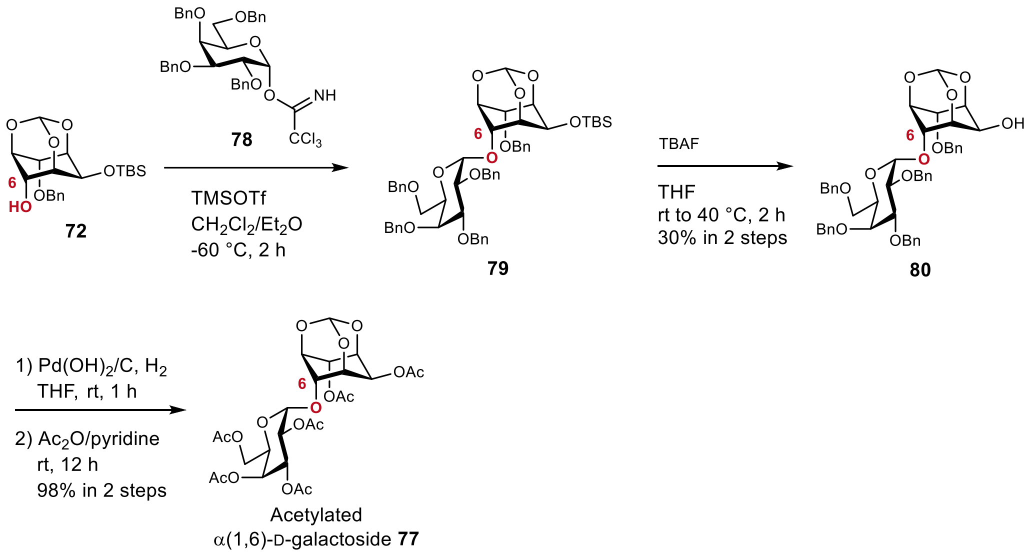

Supplementary Figure 17. Synthesis of acetylated α(1,6)-D-galactoside 77 from optically pure inositol 72.

4-*O*-Benzyl-6-*O*-(2,3,4,6-tetra-*O*-benzyl-α-D-galactopyranosyl)-D-*myo*-inositol-1,3,5-orthoformate (80)

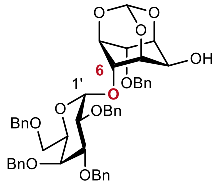

80

To a solution of **78**[15] (43.1 mg, 62.9 μmol) and **72** (12.4 mg, 31.4 μmol) in dry CH_2Cl_2-Et_2O (1/1, v/v, 628 μL) was added TMSOTf (0.85 μL, 4.72 μmol) at −60 °C. After the reaction mixture was stirred for 2 h at −60 °C, the reaction was quenched by addition of Et_3N (10 μL) and H_2O (1 mL). The resultant mixture was extracted with EtOAc (5 mL×3), and then the extracts were washed with brine (5 mL), dried over anhydrous Na_2SO_4, and concentrated in *vacuo*. The residue was subjected to silica gel column chromatography (20/1 PhMe/EtOAc) to give crude **79** (16.6 mg).

To a solution of crude **79** (16.6 mg) in THF (362 μL) was added TBAF (36.2 μL, 36.2 μmol, 1.0 M in THF) at room temperature. After the reaction mixture was gradually warmed to 40 °C over a period of 1 h and was stirred for 1 h, the reaction mixture was concentrated in *vacuo*. The residue was subjected to silica gel column chromatography (2/1 $CHCl_3$/Et_2O) to give **80** (7.6 mg, 9.47 μmol, 30% yield in 2 steps).

Data for **80**: Colorless syrup; R_f 0.42 (6/1 PhMe/acetone); $[\alpha]^{24}_D$ +31.2° (*c* 0.76, $CHCl_3$); ^{1}H-NMR (500 MHz, $CDCl_3$) δ 7.38-7.26 (18H, m), 7.22-7.13 (7H, m), 5.47 (1H, d, *J*=1.0 Hz), 4.83 (1H, d, *J*=3.5 Hz H-1'), 4.80 and 4.44 (2H, ABq, *J*=12.0 Hz), 4.75 and 4.55 (2H, ABq, *J*=12.0 Hz), 4.54 and 4.46 (2H, ABq, *J*=12.0 Hz), 4.54 and 4.35 (2H, ABq, *J*=12.0 Hz), 4.52 (1H, m), 4.37 and 4.24 (2H, ABq, *J*=11.0 Hz), 4.36-4.34 (2H, m), 4.31 (1H, m), 4.25 (1H, m), 4.12 (1H, m), 4.10 (1H, m), 3.90 (1H, dd, *J*=3.5 Hz, *J*=10.0 Hz), 3.46 (1H, dd, *J*=7.0 Hz, *J*=9.5 Hz), 3.35 (1H, dd, *J*=3.0 Hz, 10.0 Hz), 3.32 (1H, br-s), 3.25 (1H, dd, *J*=5.0 Hz, 10.0 Hz), 2.92 (1H, d, *J*=11.5 Hz); ^{13}C-NMR (125 MHz, $CDCl_3$) δ 138.8, 138.6, 138.5, 138.1, 137.3, 128.4, 128.3, 128.2, 128.0×2, 127.8×2, 127.7×2, 127.6, 127.3, 127.2, 103.2, 100.1, 78.8, 76.3, 74.5, 74.4×2, 74.3, 73.5, 73.4, 73.1, 72.8, 72.5, 72.4, 70.1, 69.7, 69.2, 61.2; HRMS (ESI-TOF) 825.3220 (825.3251 calcd for $C_{48}H_{50}O_{11}Na$ $[M+Na]^+$).

2,4-Di-*O*-acetyl-6-*O*-(2,3,4,6-tetra-*O*-acetyl-α-D-galactopyranosyl)-D-*myo*-inositol-1,3,5-orthoformate (77)

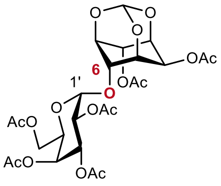

Acetylated
α(1,6)-D-galactoside **77**

To a solution of **80** (7.6 mg, 9.47 μmol) in THF (947 μL) was added 20% $Pd(OH)_2/C$ (7.6 mg, wetted with 50% water) at room temperature under Ar atmosphere. After changing the atmosphere to H_2 (balloon), the reaction mixture was stirred for 1 h. After changing the atmosphere to Ar, the reaction was filtered through celite pad, and the filtrate was concentrated in *vacuo*. To a solution of the residue in pyridine (473 μL) was added Ac_2O (473 μL) at room temperature. After the reaction mixture was stirred for 12 h at room temperature, the reaction was quenched by addition of H_2O (5 mL). The resultant mixture was extracted with EtOAc (5 mL×3), and then the extracts were washed with brine (5 mL), dried over anhydrous Na_2SO_4, and concentrated in *vacuo*. The residue was subjected to silica gel column chromatography (1/1 *n*-hexane/EtOAc) to give **77** (5.6 mg, 9.26 μmol, 98% yield in 2 steps).

Data for **77**: White solid; R_f 0.37 (1/1 *n*-hexane/EtOAc); $[\alpha]^{26}_D$ +99.8° (*c* 0.56, $CHCl_3$); mp 217-218 °C; ^{1}H-NMR (500 MHz, $CDCl_3$) δ 5.57 (1H, d, *J*=1.0 Hz), 5.50 (1H, m), 5.42 (1H, dd, *J*=2.0 Hz, *J*=3.5 Hz), 5.29 (1H, d, *J*=3.5 Hz, H-1'), 5.22 (1H, dd, *J*=3.5 Hz, *J*=11.0 Hz), 5.15 (1H, m), 5.14 (1H, dd, *J*=3.5 Hz, *J*=11.0 Hz), 4.60 (1H, m), 4.51 (1H, m), 4.33 (1H, m), 4.29 (1H, m), 4.21 (1H, m), 4.14-4.07 (2H, m), 2.20 (6H, s), 2.16 (3H, s), 2.12 (3H, s), 2.07 (3H, s), 1.99 (3H, s); ^{13}C-NMR (125 MHz, $CDCl_3$) δ 170.6, 170.4, 170.3, 170.1, 169.9×2, 103.0, 95.2, 70.7, 69.3, 68.7, 68.0, 67.7, 67.4, 67.2, 67.0, 63.0, 61.8, 21.0, 20.7, 20.6×3; HRMS (ESI-TOF) 605.1713 (605.1718 calcd for $C_{25}H_{33}O_{17}$ $[M+H]^+$).

<u>Synthesis of acetylated α-D-galactoside **77** from **40** synthesized by our desymmetric glycosylation of *meso*-diol **8**.</u>

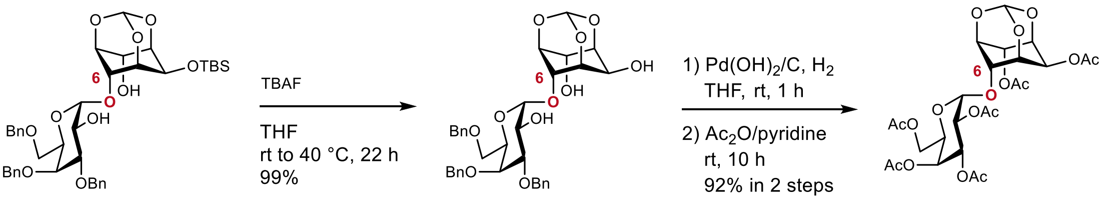

Supplementary Figure 18. Synthesis of acetylated α-D-galactoside 77 from 40.

6-*O*-(3,4,6-Tri-*O*-benzyl-α-D-galactopyranosyl)-D-*myo*-inositol-1,3,5-orthoformate (81)

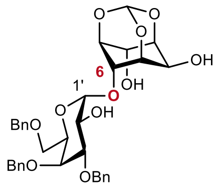

81

To a solution of **40** (13.1 mg, 17.8 μmol) in THF (356 μL) was added TBAF (35.6 μL, 35.6 μmol, 1.0 M in THF) at room temperature. The reaction mixture was stirred for 7 h and then was warmed to 40 °C. After being stirred for 15 h at 40 °C, the reaction mixture was concentrated in *vacuo*. The residue was subjected to silica gel column chromatography (1/1 PhMe/EtOAc) to give **81** (11.0 mg, 17.7 μmol, 99% yield).

Data for **81**: White solid; R_f 0.20 (6/1 PhMe/EtOAc); $[\alpha]^{24}_D$ +80.1° (*c* 1.0, $CHCl_3$); mp 176-177 °C; ^{1}H-NMR (500 MHz, $CDCl_3$) δ 7.38-7.26 (15H, m), 5.47 (1H, d, *J*=1.0 Hz), 5.09 (1H, d, *J*=4.0 Hz H-1'), 4.84 and 4.54 (2H, ABq, *J*=11.5 Hz), 4.71 and 4.55 (2H, ABq, *J*=11.5 Hz), 4.66 (1H, m), 4.51 and 4.44 (2H, ABq, *J*=11.5 Hz), 4.38-4.34 (2H, m), 4.28 (1H, m), 4.18 (1H, m), 4.17 (1H, m), 4.12 (1H, m), 4.00-3.98 (2H, m), 3.85 (1H, d, *J*=10.0 Hz), 3.60-3.53 (3H, m), 3.14 (1H, d, *J*=12.0 Hz), 2.36 (1H, d, *J*=4.0 Hz); ^{13}C-NMR (125 MHz, $CDCl_3$) δ 138.0, 137.4×2, 128.7, 128.6, 128.4, 128.1×3, 128.0, 127.8, 127.7, 102.9, 96.3, 79.2, 74.7, 74.6, 73.8, 73.1, 72.3, 71.5, 71.3, 70.8, 68.9, 68.7, 67.7, 67.6, 60.3; HRMS (ESI-TOF) 645.2318 (645.2312 calcd for $C_{34}H_{38}O_{11}Na$ $[M+Na]^+$).

2,4-Di-*O*-acetyl-6-*O*-(2,3,4,6-tetra-*O*-acetyl-α-D-galactopyranosyl)-D-*myo*-inositol-1,3,5-orthoformate (77)

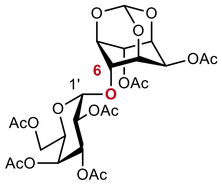

Acetylated
α-D-galactoside **77**

To a solution of **81** (11.0 mg, 17.7 μmol) in THF (1.8 mL) was added 20% $Pd(OH)_2$/C (11.0 mg, wetted with 50% water) at room temperature under Ar atmosphere. After changing the atmosphere to H_2 (balloon), the reaction mixture was stirred for 1 h. After changing the atmosphere to Ar, the reaction was filtered through celite pad, and the filtrate was concentrated in *vacuo*. To a solution of the residue in pyridine (450 μL) was added Ac_2O (450 μL) at room temperature. After the reaction mixture was stirred for 10 h at room temperature, the reaction was quenched by addition of H_2O (5 mL). The resultant mixture was extracted with EtOAc (5 mL×3), and then the extracts were washed

with brine (5 mL), dried over anhydrous Na_2SO_4, and concentrated in *vacuo*. The residue was subjected to silica gel column chromatography (1/1 *n*-hexane/EtOAc) to give **77** (9.8 mg, 16.2 μmol, 92% yield in 2 steps).

Data for **77**: White solid; R_f 0.37 (1/1 *n*-hexane/EtOAc); $[\alpha]^{26}_D$ +100.5° (*c* 0.98, $CHCl_3$); mp 217-218 °C; ^{1}H-NMR (500 MHz, $CDCl_3$) δ 5.57 (1H, d, *J*=1.5 Hz), 5.50 (1H, m), 5.42 (1H, br-d, *J*=3.5 Hz), 5.29 (1H, d, *J*=3.5 Hz, H-1'), 5.22 (1H, dd, *J*=3.5 Hz, *J*=11.0 Hz), 5.15 (1H, m), 5.14 (1H, dd, *J*=3.5 Hz, *J*=11.0 Hz), 4.60 (1H, m), 4.51 (1H, m), 4.33 (1H, m), 4.29 (1H, m), 4.21 (1H, m), 4.14-4.07 (2H, m), 2.20 (6H, s), 2.16 (3H, s), 2.12 (3H, s), 2.07 (3H, s), 1.99 (3H, s); ^{13}C-NMR (125 MHz, $CDCl_3$) δ 170.6, 170.4, 170.3, 170.1, 169.9×2, 103.0, 95.2, 70.7, 69.3, 68.7, 68.0, 67.7, 67.4, 67.2, 67.0, 62.9, 61.8, 21.0, 20.7, 20.6×3; HRMS (ESI-TOF) 605.1716 (605.1718 calcd for $C_{25}H_{33}O_{17}$ $[M+H]^+$).

Determination of glycosylation site of 40

Comparison of NMR data of acetylated α-D-galactosides **77**

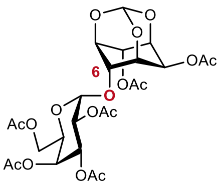

Acetylated
α(1,6)-D-galactoside **77**

Supplementary Table 18. Comparison of ^{1}H-NMR data of acetylated α-D-galactosides 77.

δ (Structurally defined acetylated α(1,6)-D-galactoside **77**) (500 MHz, $CDCl_3$)	δ (Acetylated α-D-galactoside **77** derived from **40**) (500 MHz, $CDCl_3$)
5.57 (d, *J*=1.0 Hz)	5.57 (d, *J*=1.5 Hz)
5.50 (m)	5.50 (m)
5.42 (d, *J*=2.0, 3.5 Hz)	5.42 (br-d, *J*=3.5 Hz)
5.29 (d, *J*=3.5 Hz)	5.29 (d, *J*=3.5 Hz)
5.22 (dd, *J*=3.5, 11.0 Hz)	5.22 (dd, *J*=3.5, 11.0 Hz)
5.15 (m)	5.15 (m)
5.14 (dd, *J*=3.5, 11.0 Hz)	5.14 (dd, *J*=3.5, 11.0 Hz)
4.60 (m)	4.60 (m)
4.51 (m)	4.51 (m)
4.33 (m)	4.33 (m)
4.29 (m)	4.29 (m)
4.21 (m)	4.21 (m)
4.14-4.07 (m)	4.14-4.07 (m)
2.20 (s)	2.20 (s)
2.16 (s)	2.16 (s)
2.12 (s)	2.12 (s)

2.07 (s)	2.07 (s)
1.99 (s)	1.99 (s)

Supplementary Table 19. Comparison of ^{13}C-NMR data of acetylated α-D-galactosides 77.

δ (Structurally defined acetylated α(1,6)-D-galactoside **77**) (125 MHz, $CDCl_3$)	δ (Acetylated α-D-galactoside **77** derived from **40**) (125 MHz, $CDCl_3$)
170.6	170.6
170.4	170.4
170.3	170.3
170.1	170.1
169.9×2	169.9×2
103.0	103.0
95.2	95.2
70.7	70.7
69.3	69.3
68.7	68.7
68.0	68.0
67.7	67.7
67.4	67.4
67.2	67.2
67.0	67.0
63.0	62.9
61.8	61.8
21.0	21.0
20.7	20.7
20.6×3	20.6×3

Determination of glycosylation site of β(1,6)-L-rhamnoside 42.

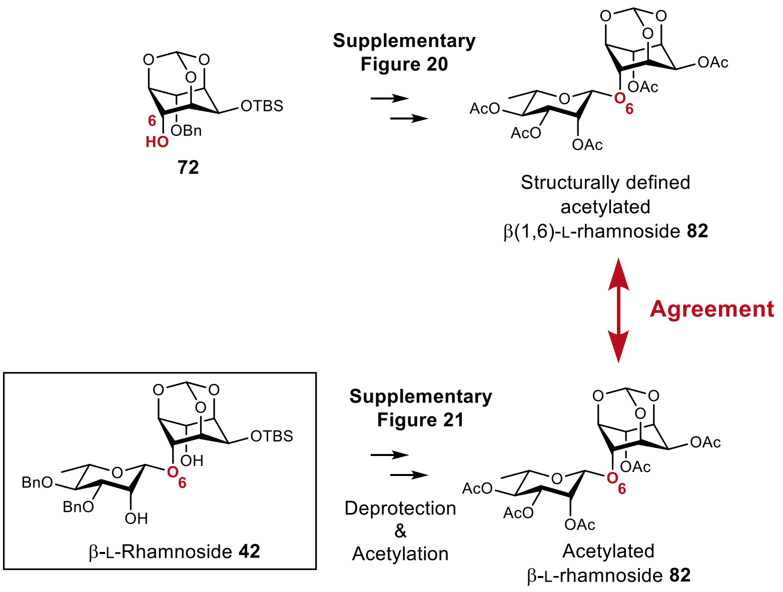

Supplementary Figure 19. Determination of glycosylation site of β-L-rhamnoside 42 synthesized by our desymmetric glycosylation of *meso*-diol 8.

Synthesis of acetylated β(1,6)-L-rhamnoside **82** from optically pure inositol **72**.

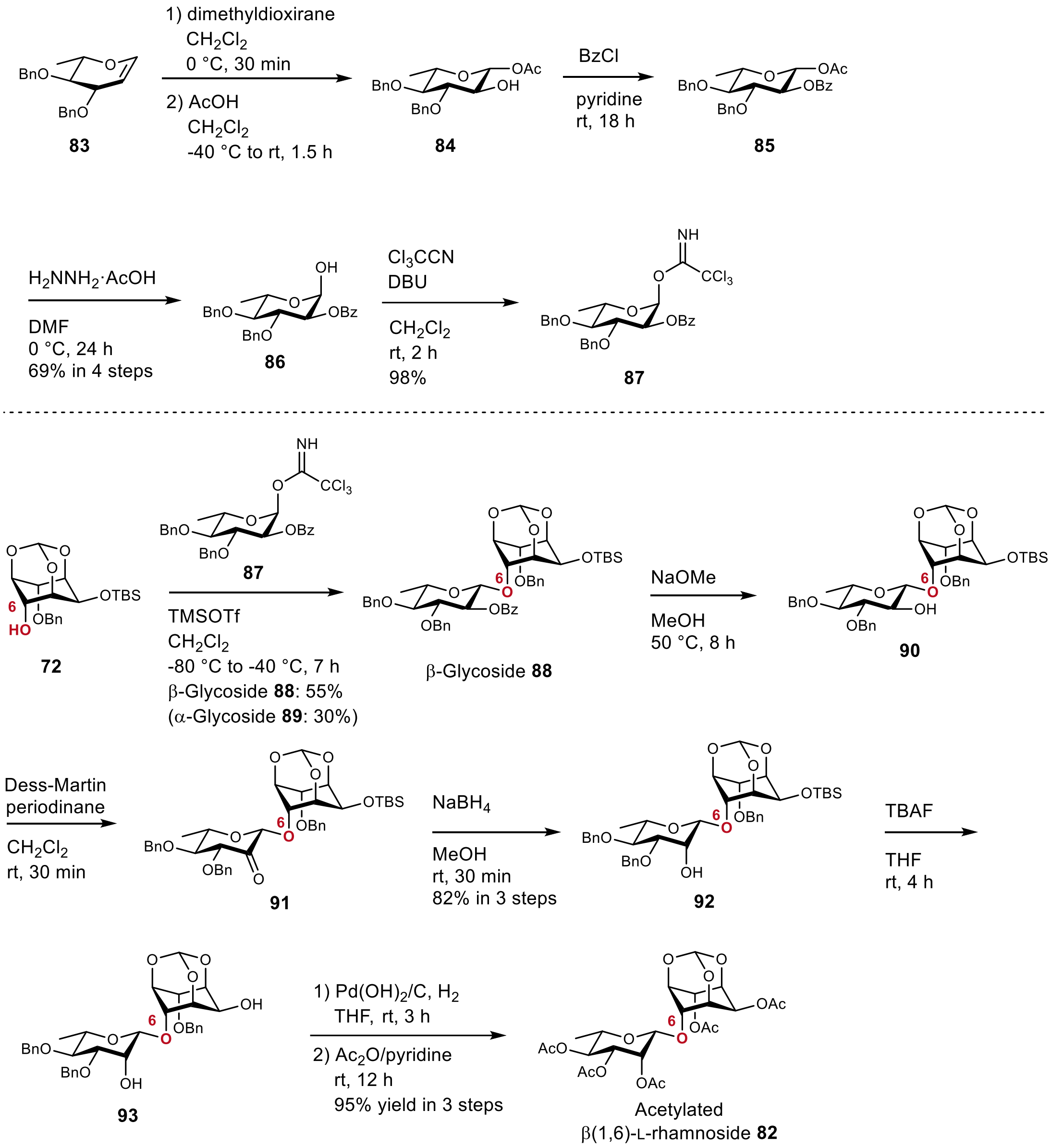

Supplementary Figure 20. Synthesis of acetylated β(1,6)-L-rhamnoside 82 from optically pure inositol 72.

2-*O*-Benzoyl-3,4-di-*O*-benzyl-6-deoxy-α-L-glucopyranose (86)

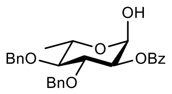

86

To a solution of 3,4-di-*O*-benzyl-L-rhamnal[16] (**83**) (1.06 g, 3.41 mmol) in CH_2Cl_2 (34.1 mL) was added dimethyldioxirane (59.3 mL, 4.09 mmol, 69 mM in acetone) at 0 °C. After the reaction mixture was stirred for 30 min at 0 °C, the reaction mixture was concentrated in *vacuo*.

To a solution of the residue in CH_2Cl_2 (34.1 mL) was added AcOH (234 μL, 4.09 mmol) at −40 °C. After the reaction mixture was stirred for 1.5 h at room temperature, the reaction mixture was concentrated in *vacuo*.

To a solution of the residue in pyridine (17.0 mL) was added BzCl (475 μL, 4.09 mmol) at 0 °C. After the reaction mixture was stirred for 18 h at room temperature, the reaction was quenched by addition of H_2O (20 mL). The resultant mixture was extracted with EtOAc (40 mL×3), and then the extracts were washed with brine (40 mL), dried over anhydrous Na_2SO_4, and concentrated in *vacuo*. The residue was subjected to silica gel column chromatography (4/1 *n*-hexane/acetone) to give crude **85** (1.56 g).

To a solution of crude **85** (1.56 g) in DMF (16.0 mL) was added H_2NNH_2· AcOH (351 mg, 3.81 mmol) at -40 °C. After the reaction mixture was stirred for 24 h at 0 °C, the reaction was quenched by addition of sat. $NaHCO_3$ aq. (10 mL). The resultant mixture was extracted with EtOAc (40 mL×3), and then the extracts were washed with brine (40 mL), dried over anhydrous Na_2SO_4, and concentrated in *vacuo*. The residue was subjected to silica gel column chromatography (2/1 $CHCl_3/Et_2O$) to give crude **86** (1.39 g). Recrystalization from *n*-hexane-EtOAc gave **86** (1.06 g, 2.36 mmol, 69% yield in 4 steps).

Data for **86**: White solid; R_f 0.36 (2/1 *n*-hexane/EtOAc); $[\alpha]^{21}_D$ −143.4° (*c* 1.16, $CHCl_3$); mp 164-165 °C; ^{1}H-NMR (500 MHz, $CDCl_3$) δ 8.06-8.04 (2H, m), 7.57 (1H, m), 7.45-7.42 (2H, m), 7.36-7.28 (5H, m), 7.21-7.16 (5H, m), 5.46 (1H, dd, *J*=4.0 Hz, *J*=3.5 Hz), 5.11 (1H, ddd, *J*=1.0 Hz, *J*=4.0 Hz, *J*=10.0 Hz), 4.90 and 4.67 (2H, ABq, *J*=11.5 Hz), 4.83 and 4.81 (2H, ABq, *J*=11.0 Hz), 4.19 (1H, dd, *J*=9.5 Hz, *J*=10.0 Hz), 4.10 (1H, m), 3.29 (1H, dd, *J*=9.5 Hz, *J*=9.5 Hz), 2.83 (1H, dd, *J*=1.0 Hz, *J*=4.0 Hz), 1.30 (3H, d, *J*=6.0 Hz); ^{13}C-NMR (125 MHz, $CDCl_3$) δ 165.9, 138.1×2, 133.3, 129.8, 129.6, 128.5, 128.4, 128.3, 128.0, 127.9, 127.8, 127.6, 90.4, 83.8, 79.4, 75.5, 75.4, 74.5, 67.0, 17.8; HRMS (ESI-TOF) 487.1503 (487.1523 calcd for $C_{27}H_{28}O_6K$ $[M+K]^+$).

2-*O*-Benzoyl-3,4-di-*O*-benzyl-6-deoxy-α-L-glucopyranosyl trichloroacetimidate (87)

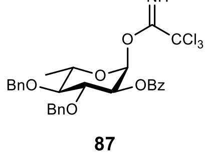

87

To a solution of **86** (123 mg, 0.275 mmol) and Cl_3CCN (167 μL, 1.65 mmol) in CH_2Cl_2 (2.7 mL) was added DBU (24.7 μL, 0.165 mmol) at 0 °C. After the reaction mixture was stirred for 2 h at room

temperature, the reaction mixture was concentrated in *vacuo*. The residue was subjected to silica gel column chromatography (3/1 *n*-hexane/Et_2O) to give **87** (160 mg, 0.269 mmol, 98% yield).

Data for **87**: Colorless syrup; R_f 0.70 (66/33/2 *n*-hexane/EtOAc/Et_3N); $[\alpha]^{21}_D$ −123.7° (*c* 0.93, $CHCl_3$); ^{1}H-NMR (500 MHz, $CDCl_3$) δ 8.48 (1H, br-s), 7.98-7.96 (2H, m), 7.55 (1H, m), 7.42-7.38 (2H, m), 7.37-7.28 (5H, m), 7.21-7.15 (5H, m), 6.53 (1H, d, *J*=3.5 Hz), 5.35 (1H, dd, *J*=3.5 Hz, *J*=10.0 Hz), 4.93 and 4.70 (2H, ABq, *J*=10.5 Hz), 4.84 and 4.80 (2H, ABq, *J*=11.0 Hz), 4.24 (1H, dd, *J*=9.5 Hz, *J*=10.0 Hz), 4.07 (1H, m), 3.40 (1H, dd, *J*=9.5 Hz, *J*=9.5 Hz), 1.35 (3H, d, *J*=6.0 Hz); ^{13}C-NMR (125 MHz, $CDCl_3$) δ 165.5, 160.7, 137.8, 137.7, 133.3, 129.7, 129.2, 128.5, 128.4, 128.3, 128.2, 128.0, 127.7, 93.8, 91.0, 83.0, 79.3, 75.7, 75.5, 72.8, 70.0, 17.8; HRMS (ESI-TOF) 592.1056 (592.1060 calcd for $C_{29}H_{29}NO_6Cl_3$ $[M+H]^+$).

4-*O*-Benzyl-2-*O*-*tert*-butyldimethylsilyl-6-*O*-(2-*O*-benzoyl-3,4-di-*O*-benzyl-6-deoxy-β-L-glucopyranosyl)-D-*myo*-inositol-1,3,5-orthoformate (88) and 4-*O*-benzyl-2-*O*-*tert*-butyldimethylsilyl-6-*O*-(2-*O*-benzoyl-3,4-di-*O*-benzyl-6-deoxy-α-L-glucopyranosyl)-D-*myo*-inositol-1,3,5-orthoformate (89)

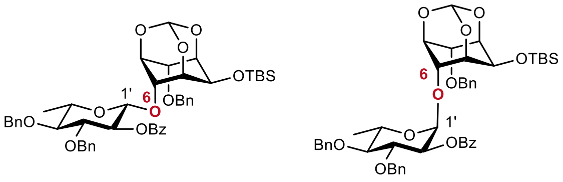

88 **89**

To a solution of **87** (485 mg, 0.818 mmol) and **72** (26.9 mg, 68.2 μmol) in dry CH_2Cl_2 (628 μL) was added TMSOTf (2.5 μL, 13.6 μmol) at −60 °C. The reaction mixture was stirred for 2 h at −60 °C and then was warmed to −40 °C. After being stirred for 5 h at −40 °C, the reaction was quenched by addition of sat. $NaHCO_3$ aq. (1 mL). The resultant mixture was extracted with $CHCl_3$ (5 mL×3), and then the extracts were washed with brine (5 mL), dried over anhydrous Na_2SO_4, and concentrated in *vacuo*. The residue was subjected to silica gel column chromatography (1/1 *n*-hexane/Et_2O) to give β-glycoside **88** (30.8 mg, 37.3 μmol, 55% yield) and α-glycoside **89** (17.1 mg, 20.7 μmol, 30% yield).

Data for **88**: Colorless syrup; R_f 0.48 (1/1 *n*-hexane/Et_2O); $[\alpha]^{23}_D$ +15.2° (*c* 1.0, $CHCl_3$); ^{1}H-NMR (500 MHz, $CDCl_3$) δ 7.99-7.97 (2H, m), 7.56 (1H, m), 7.43-7.37 (4H, m), 7.35-7.25 (8H, m), 7.13-7.10 (5H, m), 5.48 (1H, d, *J*=1.5 Hz), 5.25 (1H, dd, *J*=8.0 Hz, *J*=9.5 Hz), 4.79 and 4.56 (2H, ABq, *J*=11.0 Hz), 4.77 and 4.45 (2H, ABq, *J*=12.0 Hz), 4.69 and 4.60 (2H, ABq, *J*=11.0 Hz), 4.64 (1H, d, *J*=8.0 Hz, H-1'), 4.59 (1H, m), 4.46 (1H, m), 4.24 (1H, m), 4.21 (1H, m), 4.02 (1H, m), 3.92 (1H, m),

3.71 (1H, dd, J=9.5 Hz, J=9.5 Hz), 3.48 (1H, m), 3.03 (1H, dd, J=9.5 Hz, J=9.5 Hz), 1.29 (3H, d, J=6.5 Hz), 0.79 (9H, s), −0.10 (3H, s), −0.11 (3H, s); ^{13}C-NMR (125 MHz, $CDCl_3$) δ 164.8, 138.2, 137.8×2, 133.1, 129.8, 128.5, 128.3, 128.2, 128.0, 127.9×2, 127.6, 127.3, 126.9, 103.0, 101.8, 82.9, 82.4, 75.4, 75.3, 74.8, 73.5×2, 73.2, 73.1, 71.6, 70.1, 68.7, 61.4, 25.8, 18.2, 17.8, −4.9, −5.1; HRMS (ESI-TOF) 825.3638 (825.3670 calcd for $C_{47}H_{57}O_{11}Si$ $[M+H]^+$).

Data for **89**: Colorless syrup; R_f 0.54 (1/1 *n*-hexane/Et_2O); $[\alpha]^{23}_{D}$ −102.2° (*c* 1.0, $CHCl_3$); ^{1}H-NMR (500 MHz, $CDCl_3$) δ 7.99-7.97 (2H, m), 7.57 (1H, m), 7.41-7.31 (7H, m), 7.26-7.12 (8H, m), 7.07-7.06 (2H, m), 5.45 (1H, d, J=1.0 Hz), 5.38 (1H, d, J=3.5 Hz, H-1'), 5.09 (1H, dd, J=3.5 Hz, J=10.0 Hz), 4.89 and 4.68 (2H, ABq, J=11.0 Hz), 4.65 and 4.61 (2H, ABq, J=11.0 Hz), 4.43 (1H, m), 4.38 and 4.13 (2H, ABq, J=12.5 Hz), 4.33 (1H, m), 4.28 (1H, m), 4.11 (1H, m), 4.03 (1H, dd, J=9.0 Hz, J=10.0 Hz), 3.87 (1H, m), 3.79 (1H, m), 3.30 (1H, dd, J=9.0 Hz, J=9.0 Hz), 1.33 (3H, d, J=6.0 Hz), 0.96 (9H, s), 0.15 (6H, s); ^{13}C-NMR (125 MHz, $CDCl_3$) δ 165.7, 138.0, 137.4, 133.3, 129.7, 129.6, 128.6, 128.5, 128.4, 128.3, 128.0, 127.9×2, 127.8, 127.7, 127.6, 103.1, 94.8, 83.5, 79.6, 75.5, 75.4, 74.1, 73.9, 73.1, 71.9, 71.4, 67.9, 67.4, 61.8, 26.0, 18.4, 17.9, −4.5, −4.7; HRMS (ESI-TOF) 825.3693 (825.3670 calcd for $C_{47}H_{57}O_{11}Si$ $[M+H]^+$).

4-*O*-Benzyl-2-*O*-*tert*-butyldimethylsilyl-6-*O*-(3,4-di-*O*-benzyl-β-L-rhamnopyranosyl)-D-*myo*-inositol-1,3,5-orthoformate (92)

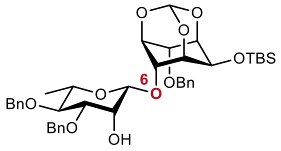

92

To a solution of **88** (13.0 mg, 15.8 μmol) in MeOH (630 μL) was added NaOMe (3.0 μL, 0.158 mmol, 28% in MeOH) at room temperature. After the reaction mixture was stirred for 8 h at 50 °C, the reaction was quenched by addition of sat. NH_4Cl aq. (1 mL). The resultant mixture was extracted with EtOAc (5 mL×3), and then the extracts were washed with brine (5 mL), dried over anhydrous Na_2SO_4, and concentrated in *vacuo*. The residue was subjected to silica gel column chromatography (2/1 *n*-hexane/Et_2O) to give crude **90** (10.1 mg).

To a solution of crude **90** (10.1 mg) in CH_2Cl_2 (1.4 mL) was added Dess-Martin periodinane (17.8 mg, 42.0 μmol) at room temperature. After the reaction mixture was stirred for 30 min at room temperature, the reaction mixture was filtered through celite pad, and to the filtrate was added sat. $NaHCO_3$ aq. (1 mL). The resultant mixture was extracted with $CHCl_3$ (5 mL×3), and then the extracts were washed with brine (5 mL), dried over anhydrous Na_2SO_4, and concentrated in *vacuo*. The

residue was subjected to silica gel column chromatography (3/1 *n*-hexane/EtOAc) to give crude **91** (9.7 mg).

To a solution of crude **91** (9.7 mg) in MeOH (1.4 mL) was added $NaBH_4$ (5.1 mg, 0.135 mmol) at room temperature. After the reaction mixture was stirred for 30 min at room temperature, the reaction was quenched by addition of H_2O (1 mL). The resultant mixture was extracted with EtOAc (5 mL×3), and then the extracts were washed with brine (5 mL), dried over anhydrous Na_2SO_4, and concentrated in *vacuo*. The residue was subjected to silica gel column chromatography (1/1 *n*-hexane/Et_2O) to give **92** (9.3 mg, 12.9 mmol, 82% yield in 3 steps).

Data for **92**: Colorless syrup; R_f 0.43 (1/1 *n*-hexane/Et_2O); $[\alpha]^{24}_D$ +7.2° (*c* 0.71, $CHCl_3$); ^{1}H-NMR (500 MHz, $CDCl_3$) δ 7.40-7.26 (15H, m), 5.46 (1H, s), 4.98 and 4.66 (2H, ABq, *J*=10.5 Hz), 4.81 and 4.68 (2H, ABq, *J*=12.0 Hz), 4.69 and 4.62 (2H, ABq, *J*=12.5 Hz), 4.62 (1H, s), 4.54 (1H, m), 4.49 (1H, m), 4.26 (1H, m), 4.23 (1H, m), 4.06-4.02 (3H, m), 3.61 (1H, dd, *J*=9.0 Hz, *J*=9.0 Hz), 3.49 (1H, dd, *J*=2.5 Hz, *J*=9.0 Hz), 3.36 (1H, m), 3.03 (1H, m), 1.37 (3H, d, *J*=6.0 Hz), 0.94 (9H, s), 0.14 (3H, s), 0.13 (3H, s); ^{13}C-NMR (125 MHz, $CDCl_3$) δ 138.3, 138.0, 137.3, 128.6, 128.4, 128.1, 127.9, 127.8×2, 102.8, 99.2 ($^1J_{CH}$=159 Hz), 80.9, 79.4, 75.5, 73.6, 73.1×2, 72.8, 71.9, 71.8, 71.3, 69.6, 68.4, 61.5, 26.0, 18.5, 18.0, −4.6×2; HRMS (ESI-TOF) 743.3224 (743.3227 calcd for $C_{40}H_{52}O_{10}SiNa$ $[M+Na]^+$).

2,4-*O*-Acetyl-6-*O*-(2,3,4-tri-*O*-acetyl-β-L-rhamnopyranosyl)-D-*myo*-inositol-1,3,5-orthoformate (82)

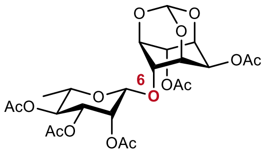

Acetylated
β(1,6)-L-rhamnoside **82**

To a solution of **92** (7.1 mg, 9.85 μmol) in THF (394 μL) was added TBAF (19.7 μL, 19.7 μmol, 1.0 M in THF) at room temperature. After the reaction mixture was stirred for 4 h at room temperature, the reaction mixture was concentrated in *vacuo*. The residue was subjected to silica gel column chromatography (1/1 *n*-hexane/EtOAc) to give crude **93** (6.0 mg).

To a solution of crude **93** (6.0 mg) in THF (985 μL) was added 20% $Pd(OH)_2/C$ (6.0 mg, wetted with 50% water) at room temperature under Ar atmosphere. After changing the atmosphere to H_2 (balloon), the reaction mixture was stirred for 3 h. After changing the atmosphere to Ar, the reaction was filtered through celite pad, and the filtrate was concentrated in *vacuo*. To a solution of the residue

in pyridine (493 μL) was added Ac_2O (493 μL) at room temperature. After the reaction mixture was stirred for 12 h at room temperature, the reaction was quenched by addition of H_2O (5 mL). The resultant mixture was extracted with EtOAc (5 mL×3), and then the extracts were washed with brine (5 mL), dried over anhydrous Na_2SO_4, and concentrated in *vacuo*. The residue was subjected to silica gel column chromatography (3/1 *n*-hexane/EtOAc) to give **82** (5.1 mg, 9.33 μmol, 95% yield in 3 steps).

Data for **82**: Colorless syrup; R_f 0.33 (1/1 *n*-hexane/EtOAc); $[\alpha]^{25}_D$ +42.0° (*c* 0.51, $CHCl_3$); ^{1}H-NMR (500 MHz, $CDCl_3$) δ 5.55 (1H, d, *J*=1.5 Hz), 5.50 (1H, m), 5.48 (1H, m), 5.14 (1H, m), 5.01 (1H, dd, *J*=3.5 Hz, *J*=10.0 Hz), 4.97 (1H, dd, *J*=10.0 Hz, *J*=10.0 Hz), 4.77 (1H, d, *J*=1.5 Hz), 4.67 (1H, m), 4.52 (1H, m), 4.26-4.25 (2H, m), 3.52 (1H, m), 2.21 (3H, s), 2.18 (3H, s), 2.16 (3H, s), 2.06 (3H, s), 1.99 (3H, s), 1.24 (3H, d, *J*=6.0 Hz); ^{13}C-NMR (125 MHz, $CDCl_3$) δ 170.3, 170.0×2, 169.8, 169.6, 102.9, 99.0, 75.0, 70.8, 70.6, 70.2, 69.7, 69.5, 69.0, 67.8, 67.3, 63.1, 21.1, 21.0, 20.8, 20.7, 20.6, 17.3; HRMS (ESI-TOF) 569.1462 (569.1482 calcd for $C_{23}H_{30}O_{15}Na$ $[M+Na]^+$).

Synthesis of acetylated β-L-rhamnoside **82** from **42**.

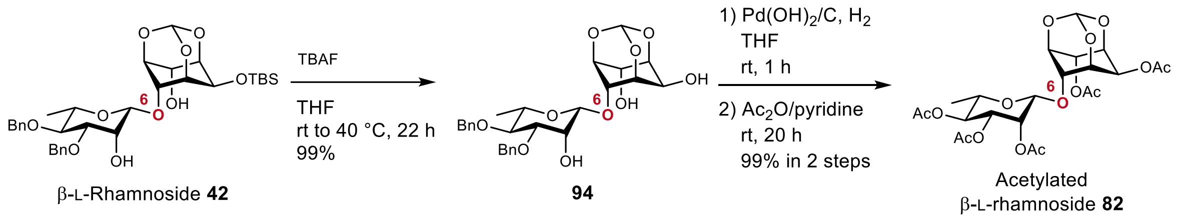

Supplementary Figure 21. Synthesis of acetylated β-L-rhamnoside 82 from 42.

6-*O*-(3,4-Di-*O*-benzyl-β-L-rhamnopyranosyl)-D-*myo*-inositol-1,3,5-orthoformate (94)

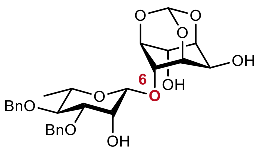

94

To a solution of **42** (13.3 mg, 21.1 μmol) in THF (422 μL) was added TBAF (42.2 μL, 42.2 μmol, 1.0 M in THF) at room temperature. The reaction mixture was stirred for 7 h and then was warmed to 40 °C. After being stirred for 15 h at 40 °C, the reaction mixture was concentrated in *vacuo*. The residue was subjected to silica gel column chromatography (1/1 PhMe/EtOAc) to give **94** (10.8 mg, 20.9 μmol, 99% yield).

Data for **94**: Colorless syrup; R_f 0.14 (1/1 PhMe/EtOAc); $[\alpha]^{25}_{D}$ +9.1° (*c* 1.0, $CHCl_3$); ^{1}H-NMR (500 MHz, $CDCl_3$) δ 7.36-7.29 (10H, m), 5.45 (1H, s), 4.88 and 4.63 (2H, ABq, *J*=11.0 Hz), 4.70 and 4.67 (2H, ABq, *J*=11.5 Hz), 4.61 (1H, s), 4.60 (1H, m), 4.52-4.50 (2H, m), 4.21-4.18 (2H, m), 4.12 (1H, m), 4.03 (1H, br-s), 4.01 (1H, d, *J*=8.0 Hz), 3.54 (1H, dd, *J*=3.5 Hz, *J*=9.0 Hz), 3.46 (1H, dd, *J*=9.0 Hz, *J*=9.0 Hz) 3.37 (1H, m), 3.09 (1H, d, *J*=12.0 Hz), 2.48 (1H, br-s), 1.36 (3H, d, *J*=6.0 Hz); ^{13}C-NMR (125 MHz, $CDCl_3$) δ 137.9, 137.2, 128.7, 128.5, 128.2, 128.1, 127.9×2, 102.6, 99.5, 80.9, 79.0, 75.5, 74.7, 73.6, 72.4, 71.9, 68.9, 67.9, 67.7, 60.8, 17.8; HRMS (ESI-TOF) 539.1913 (539.1893 calcd for $C_{27}H_{32}O_{10}Na$ $[M+Na]^+$).

2,4-Di-*O*-acetyl-6-*O*-(2,3,4-tri-*O*-acetyl-β-L-rhamnopyranosyl)-D-*myo*-inositol-1,3,5-orthoformate (82)

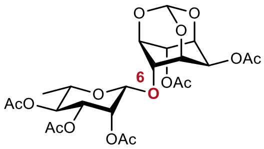

Acetylated
β-L-rhamnoside **82**

To a solution of **94** (12.9 mg, 25.0 μmol) in THF (2.5 mL) was added 20% $Pd(OH)_2/C$ (12.9 mg, wetted with 50% water) at room temperature under Ar atmosphere. After changing the atmosphere to H_2 (balloon), the reaction mixture was stirred for 1 h. After changing the atmosphere to Ar, the reaction was filtered through celite pad, and the filtrate was concentrated in *vacuo*. To a solution of the residue in pyridine (625 μL) was added Ac_2O (625 μL) at room temperature. After the reaction mixture was stirred for 20 h at room temperature, the reaction was quenched by addition of H_2O (5 mL). The resultant mixture was extracted with EtOAc (5 mL×3), and then the extracts were washed with brine (5 mL), dried over anhydrous Na_2SO_4, and concentrated in *vacuo*. The residue was subjected to silica gel column chromatography (2/1 *n*-hexane/EtOAc) to give **82** (13.6 mg, 24.9 μmol, 99% yield in 2 steps).

Data for **82**: Colorless syrup; R_f 0.33 (1/1 *n*-hexane/EtOAc); $[\alpha]^{27}_{D}$ +42.5° (*c* 0.41, $CHCl_3$); ^{1}H-NMR (500 MHz, $CDCl_3$) δ 5.56 (1H, d, *J*=1.0 Hz), 5.50 (1H, m), 5.48 (1H, m), 5.14 (1H, m), 5.01 (1H, dd, *J*=3.5 Hz, *J*=10.0 Hz), 4.97 (1H, dd, *J*=10.0 Hz, *J*=10.0 Hz), 4.77 (1H, d, *J*=1.5 Hz), 4.67 (1H, m), 4.52 (1H, m), 4.26-4.25 (2H, m), 3.52 (1H, m), 2.21 (3H, s), 2.18 (3H, s), 2.16 (3H, s), 2.06 (3H, s), 1.99 (3H, s), 1.24 (3H, d, *J*=6.5 Hz); ^{13}C-NMR (125 MHz, $CDCl_3$) δ 170.3, 170.0×2, 169.8, 169.6, 102.9, 99.0, 75.0, 70.8, 70.6, 70.2, 69.7, 69.5, 69.0, 67.8, 67.3, 63.1, 21.1, 21.0, 20.8, 20.7, 20.6, 17.3; HRMS (ESI-TOF) 569.1498 (569.1482 calcd for $C_{23}H_{30}O_{15}Na$ $[M+Na]^+$).

Determination of glycosylation site of 42

Comparison of NMR data of acetylated β-L-rhamnosides **82**

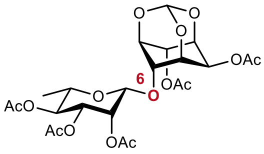

Acetylated
β(1,6)-L-rhamnoside **82**

Supplementary Table 20. Comparison of ^{1}H-NMR data of acetylated β-L-rhamnosides 82.

δ(Structurally defined acetylated β(1,6)-L-rhamnoside **82**) (500 MHz, $CDCl_3$)	δ(Acetylated β-L-rhamnoside **82** derived from **42**) (500 MHz, $CDCl_3$)
5.55 (d, *J*=1.5 Hz)	5.56 (d, *J*=1.0 Hz)
5.50 (m)	5.50 (m)
5.48 (m)	5.48 (m)
5.14 (m)	5.14 (m)
5.01 (dd, *J*=3.5, 10.0 Hz)	5.01 (dd, *J*=3.5, 10.0 Hz)
4.97 (dd, *J*=10.0, 10.0 Hz)	4.97 (dd, *J*=10.0, 10.0 Hz)
4.77 (d, *J*=1.5 Hz)	4.77 (d, *J*=1.5 Hz)
4.67 (m),	4.67 (m),
4.52 (m)	4.52 (m)
4.26-4.25 (m)	4.26-4.25 (m)
3.52 (m)	3.52 (m)
2.21 (s)	2.21 (s)
2.18 (s)	2.18 (s)
2.16 (s)	2.16 (s)
2.06 (s)	2.06 (s)
1.99 (s)	1.99 (s)
1.24 (d, *J*=6.0 Hz)	1.24 (d, *J*=6.5 Hz)

Supplementary Table 21. Comparison of ^{13}C-NMR data of acetylated β-L-rhamnosides 82.

δ(Structurally defined acetylated β(1,6)-L-rhamnoside **82**) (125 MHz, $CDCl_3$)	δ(Acetylated β-L-rhamnoside **82** derived from **42**) (125 MHz, $CDCl_3$)
170.3	170.3
170.0×2	170.0×2
169.8	169.8
169.6	169.6
102.9	102.9
99.0	99.0
75.0	75.0
70.8	70.8
70.6	70.6
70.2	70.2
69.7	69.7
69.5	69.5
69.0	69.0
67.8	67.8
67.3	67.3

63.1	63.1
21.1	21.1
21.0	21.0
20.8	20.8
20.7	20.7
20.6	20.6
17.3	17.3

Determination of glycosylation site of β(1,4)-D-mannoside 43.

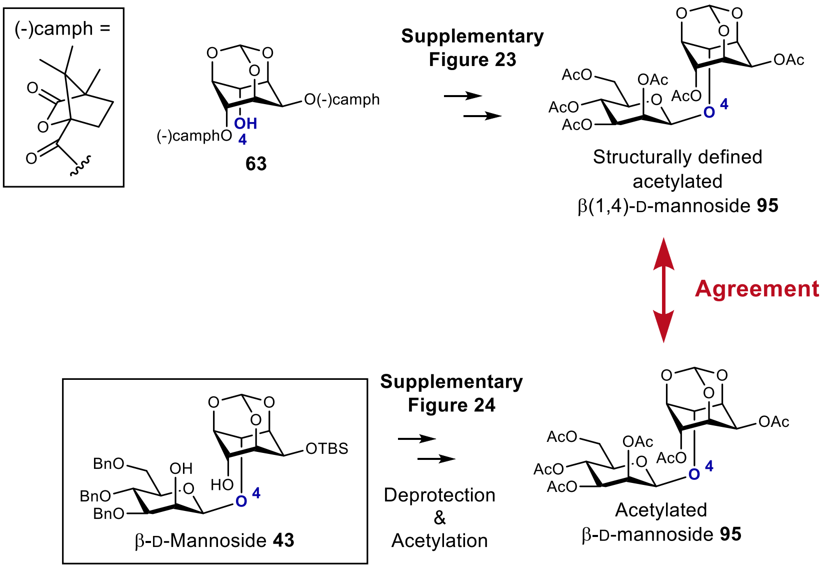

Supplementary Figure 22. Determination of glycosylation site of β-D-mannoside 43.

Synthesis of acetylated β(1,4)-D-mannoside **95** from optically pure inositol **63**.

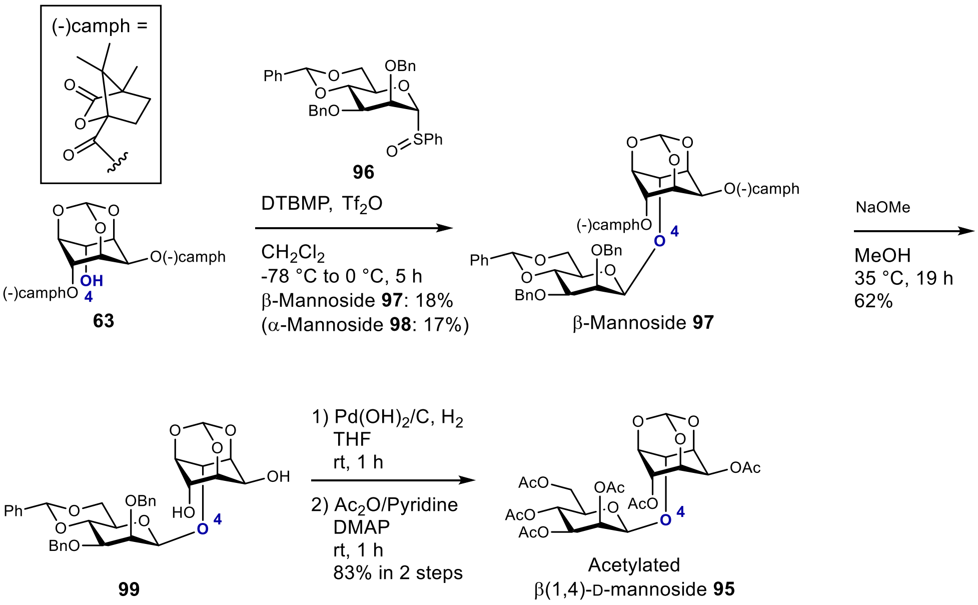

Supplementary Figure 23. Synthesis of acetylated β(1,4)-D-mannoside 95 from optically pure inositol 63.

4-*O*-(2,3-Di-*O*-benzyl-4,6-*O*-benzylidene-β-D-mannopyranosyl)-2,6-di-*O*-[(-)-ω-camphanoyl]-D-*myo*-inositol-1,3,5-orthoformate (97) and 4-*O*-(2,3-di-*O*-benzyl-4,6-*O*-benzylidene-α-D-mannopyranosyl)-2,6-di-*O*-[(-)-ω-camphanoyl]-D-*myo*-inositol-1,3,5-orthoformate (98)

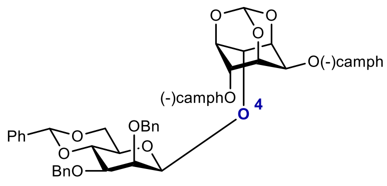

97

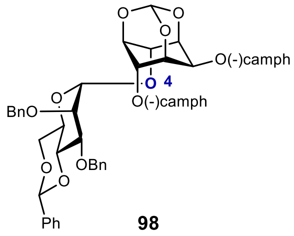

98

To a solution of **96**[17] (10.0 mg, 18.0 μmol) and 2,6-di-*tert*-butyl-4-methylpyridine (7.4 mg, 35.9 μmol) in CH_2Cl_2 (720 μL) was added Tf_2O (3.33 μL, 19.8 μmol) at −78 °C and was dropwised a solution of **63**[13] (11.2 mg, 21.5 μmol) in CH_2Cl_2 (180 μL). After the reaction mixture was gradually warmed to 0 °C over a period of 5 h, the reaction was quenched by addition of sat. $NaHCO_3$ aq. (1 mL). The resultant mixture was extracted with EtOAc (5 mL×3), and then the extracts were washed with brine (5 mL), dried over anhydrous Na_2SO_4, and concentrated in *vacuo*. The residue was subjected to silica gel column chromatography (2/1 *n*-hexane/EtOAc) to give β-mannoside **97** (3.0 mg, 3.15 μmol, 18% yield) and α-mannoside **98** (2.9 mg, 3.05 μmol, 17% yield).

Data for **97**: White solid; R_f 0.18 (2/1 *n*-hexane/EtOAc); $[\alpha]^{25}_D$ −43.3° (*c* 1.0, $CHCl_3$); mp 118-119 °C; ^{1}H-NMR (500 MHz, $CDCl_3$) δ 7.51-7.46 (4H, m), 7.41-7.23 (11H, m), 5.57 (1H, s), 5.55 (1H, d, *J*=1.0 Hz), 5.52 (1H, m), 5.34 (1H, m), 5.01 and 4.81 (2H, ABq, *J*=12.5 Hz), 4.80 (1H, m), 4.60 and 4.57 (2H, ABq, *J*=12.0 Hz), 4.60 (1H, br-s), 4.54 (1H, m), 4.50 (1H, m), 4.36 (1H, m), 4.28 (1H, dd, *J*=5.0 Hz, *J*=11.0 Hz), 4.05 (1H, d, *J*=3.5 Hz), 4.04 (1H, dd, *J*=10.0 Hz, *J*=10.0 Hz), 3.74 (1H, dd, *J*=10.5 Hz, *J*=11.0 Hz), 3.57 (1H, dd, *J*=3.5 Hz, *J*=10.0 Hz), 3.33 (1H, m), 2.55 (1H, m), 2.33 (1H, m), 2.26-2.18 (2H, m), 1.99 (1H, m), 1.79-1.71 (2H, m), 1.35 (1H, m), 1.17 (3H, s), 1.15 (3H, s), 1.08 (3H, s), 1.06 (3H, s), 1.04 (3H, s), 0.94 (3H, s); ^{13}C-NMR (125 MHz, $CDCl_3$) δ 177.9, 177.8, 166.9, 166.8, 138.1, 137.9, 137.3, 129.0, 128.7, 128.3, 128.2×2, 127.6×2, 126.0, 104.4 ($^1J_{CH}$=154 Hz), 103.0, 101.5, 91.0, 90.6, 78.0, 77.1, 76.4, 75.8, 75.3, 72.3, 69.2, 69.1, 69.0, 68.7, 68.4, 67.9, 64.8, 54.8, 54.7, 54.5×2, 30.7, 30.6, 29.1, 28.8, 16.9, 16.8, 16.6×2, 9.7, 9.6; HRMS (ESI-TOF) 981.3920 (981.3909 calcd for $C_{54}H_{61}O_{17}$ $[M+H]^+$).

Data for **98**: Colorless syrup; R_f 0.20 (2/1 *n*-hexane/EtOAc); $[\alpha]^{23}_D$ +11.0° (*c* 0.74, $CHCl_3$); ^{1}H-NMR (500 MHz, $CDCl_3$) δ 7.51-7.42 (2H, m), 7.44-7.42 (2H, m), 7.39-7.22 (11H, m), 5.70 (1H, m), 5.64 (1H, s), 5.55 (1H, d, *J*=1.5 Hz), 5.16 (1H, m), 5.07 and 4.80 (2H, ABq, *J*=12.0 Hz), 5.00 (1H, d, *J*=1.5 Hz), 4.88 and 4.82 (2H, ABq, *J*=12.0 Hz), 4.64 (1H, m), 4.48 (1H, m), 4.42 (1H, m), 4.33 (1H, dd,

J=1.5 Hz, *J*=3.5 Hz), 4.31-4.26 (3H, m), 3.98 (1H, dd, *J*=3.0 Hz, *J*=9.5 Hz), 3.88 (1H, dd, *J*=10.0 Hz, *J*=10.0 Hz), 3.74 (1H, m), 2.50 (1H, m), 2.39 (1H, m), 2.11 (1H, m), 1.94 (1H, m), 1.86-1.80 (2H, m), 1.72 (1H, m), 1.44 (1H, m), 1.13 (3H, s), 1.10 (3H, s), 1.06 (3H, s), 1.02 (9H, s); ^{13}C-NMR (125 MHz, $CDCl_3$) δ 177.8, 177.5, 167.0, 166.7, 138.9, 138.7, 137.4, 128.8, 128.2×2, 128.1, 127.5×2, 127.3, 126.0, 103.1 ($^1J_{CH}$=169 Hz), 101.4, 98.3, 90.9, 90.4, 78.6, 76.4, 76.2, 74.1, 72.7, 70.1, 68.8×2, 68.5, 68.2, 66.3, 65.2, 64.4, 54.9, 54.8, 54.7, 54.4, 30.9, 30.7, 28.9, 28.6, 16.8×2, 16.7, 16.4, 9.7×2; HRMS (ESI-TOF) 981.3940 (981.3909 calcd for $C_{54}H_{61}O_{17}$ [M+H]$^+$).

4-*O*-(2,3-Di-*O*-benzyl-4,6-*O*-benzylidene-β-D-mannopyranosyl)-D-*myo*-inositol-1,3,5-orthoformate (99)

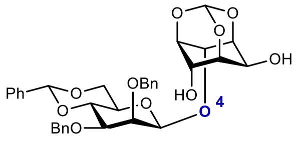

99

To a solution of **97** (11.4 mg, 12.0 μmol) in MeOH-CH_2Cl_2 (2/1, v/v, 360 μL) was added NaOMe (23.2 μL, 0.120 mmol, 28% in MeOH) at room temperature. After the reaction mixture was stirred for 19 h at 35 °C, the reaction was quenched by addition of Amberlite® IR 120 H$^+$ form. The resultant mixture was filtered, and the filtrate was concentrated in *vacuo*. The residue was subjected to silica gel column chromatography (1/1 *n*-hexane/EtOAc) to give **99** (4.6 mg, 7.41 μmol, 62% yield).

Data for **99**: White solid; R$_f$ 0.33 (1/1 *n*-hexane/EtOAc); $[\alpha]^{20}_D$ −25.7° (*c* 0.35, $CHCl_3$); mp 107-108 °C; ^{1}H-NMR (500 MHz, $CDCl_3$) δ 7.50-7.48 (2H, m), 7.41-7.28 (13H, m), 5.62 (1H, s), 5.43 (1H, d, *J*=1.0 Hz), 4.86 and 4.75 (2H, ABq, *J*=11.0 Hz), 4.86 and 4.69 (2H, ABq, *J*=12.5 Hz), 4.65 (1H, d, *J*=1.0 Hz), 4.53 (1H, m), 4.47 (1H, m), 4.44 (1H, m), 4.31 (1H, dd, *J*=5.0 Hz, *J*=10.5 Hz), 4.21 (1H, dd, *J*=10.0 Hz, *J*=10.0 Hz), 4.18 (1H, m), 4.12 (1H, m), 3.93-3.89 (3H, m), 3.66 (1H, dd, *J*=3.0 Hz, *J*=10.0 Hz), 3.38 (1H, m), 3.06 (1H, d, *J*=12.5 Hz); ^{13}C-NMR (125 MHz, $CDCl_3$) δ 138.0, 137.2×2, 129.0, 128.9, 128.5, 128.4, 128.2, 128.1, 127.8, 127.7, 126.0, 102.6, 101.5, 101.4, 78.5, 78.0, 75.9, 75.3, 74.9, 74.6, 73.2, 72.3, 68.8, 68.2, 67.8, 67.5, 60.7; HRMS (ESI-TOF) 621.2336 (621.2336 calcd for $C_{34}H_{37}O_{11}$ [M+H]$^+$).

2,6-Di-*O*-acetyl-4-*O*-(2,3,4,6-tetra-*O*-acetyl-β-D-mannopyranosyl)-D-*myo*-inositol-1,3,5-orthoformate (95)

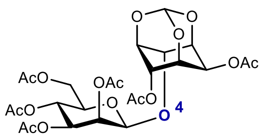

Acetylated
β(1,4)-D-mannoside **95**

To a solution of **99** (4.6 mg) in THF (741 μL) was added 20% $Pd(OH)_2/C$ (4.6 mg, wetted with 50% water) at room temperature under Ar atmosphere. After changing the atmosphere to H_2 (balloon), the reaction mixture was stirred for 1 h. After changing the atmosphere to Ar, the reaction was filtered through celite pad, and the filtrate was concentrated in *vacuo*. To a solution of the residue in pyridine (185 μL) were added Ac_2O (185 μL) and 4-dimethylaminopyridine (0.18 mg, 1.48 μmol) at room temperature. After the reaction mixture was stirred for 1 h at room temperature, the reaction was quenched by addition of H_2O (5 mL). The resultant mixture was extracted with EtOAc (5 mL×3), and then the extracts were washed with brine (5 mL), dried over anhydrous Na_2SO_4, and concentrated in *vacuo*. The residue was subjected to silica gel column chromatography (2/1 *n*-hexane/EtOAc) to give **95** (3.7 mg, 6.12 μmol, 83% yield in 2 steps).

Data for **95**: Colorless syrup; R_f 0.26 (1/1 *n*-hexane/EtOAc); $[\alpha]^{25}_{D}$ −33.0° (*c* 0.37, $CHCl_3$); ^{1}H-NMR (500 MHz, $CDCl_3$) δ 5.55 (1H, d, *J*=1.5 Hz), 5.53 (1H, dd, *J*=1.0 Hz, *J*=3.0 Hz), 5.48 (1H, m), 5.16 (1H, dd, *J*=10.0 Hz, *J*=10.0 Hz), 5.14 (1H, m), 5.06 (1H, dd, *J*=3.0 Hz, *J*=10.0 Hz), 4.81 (1H, d, *J*=1.0 Hz), 4.69 (1H, m), 4.53 (1H, m), 4.27-4.22 (3H, m), 4.05 (1H, dd, *J*=2.0 Hz, *J*=12.0 Hz), 3.68 (1H, m), 2.21 (3H, s), 2.18 (3H, s), 2.16 (3H, s), 2.12 (3H, s), 2.05 (3H, s), 2.00 (3H, s); ^{13}C-NMR (125 MHz, $CDCl_3$) δ 170.6, 170.3, 169.9×2, 169.5, 169.4, 102.9, 99.5, 75.5, 72.7, 70.6, 69.7, 69.4, 68.7, 67.8, 67.2, 65.5, 63.0, 62.4, 21.1, 20.9, 20.8, 20.7, 20.6, 20.5; HRMS (ESI-TOF) 605.1693 (605.1718 calcd for $C_{25}H_{33}O_{17}$ $[M+H]^+$).

<u>Synthesis of acetylated β-D-mannoside **95** from **43**.</u>

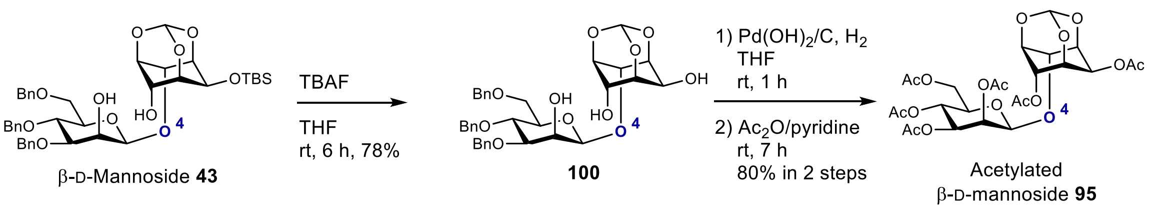

Supplementary Figure 24. Synthesis of acetylated β-D-mannoside 95.

4-*O*-(3,4,6-Tri-*O*-benzyl-β-D-mannopyranosyl)-D-*myo*-inositol-1,3,5-orthoformate (100)

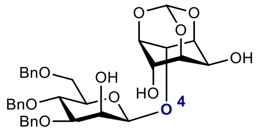

100

To a solution of **43** (15.3 mg, 20.8 μmol) in THF (416 μL) was added TBAF (41.6 μL, 41.6 μmol, 1.0 M in THF) at room temperature. After the reaction mixture was stirred for 6 h at room temperature, the reaction was quenched by addition of sat. NH_4Cl aq. (2 mL). The resultant mixture was extracted with EtOAc (5 mL×3), and then the extracts were washed with brine (5 mL), dried over anhydrous Na_2SO_4, and concentrated in *vacuo*. The residue was subjected to silica gel column chromatography (10/1 $CHCl_3$/MeOH) to give **100** (10.1 mg, 16.2 μmol, 78% yield).

Data for **100**: Colorless syrup; R_f 0.40 (6/1 PhMe/acetone); $[\alpha]^{25}_{D}$ +40.2° (*c* 0.88, $CHCl_3$); ^{1}H-NMR (500 MHz, $CDCl_3$) δ 7.37-7.28 (13H, m), 7.22-7.21 (2H, m), 5.46 (1H, s), 4.83 and 4.54 (2H, ABq, *J*=10.5 Hz), 4.70 and 4.66 (2H, ABq, *J*=11.5 Hz), 4.63 (1H, s), 4.62 (1H, m), 4.59 and 4.54 (2H, ABq, *J*=12.0 Hz), 4.55 (1H, m), 4.52 (1H, m), 4.21 (1H, m), 4.19 (1H, m), 4.13 (1H, m), 4.04 (1H, d, *J*=3.0 Hz), 3.79 (1H, dd, *J*=9.5 Hz, *J*=9.5 Hz), 3.71 (1H, dd, *J*=2.5 Hz, *J*=11.0 Hz), 3.67 (1H, dd, *J*=5.5 Hz, *J*=11.0 Hz), 3.58 (1H, dd, *J*=3.0 Hz, *J*=9.5 Hz), 3.47 (1H, m), 3,13 (1H, br-s), 2.57 (1H, br-s); ^{13}C-NMR (125 MHz, $CDCl_3$) δ 137.9, 137.8, 137.2, 128.7, 128.5, 128.4, 128.2, 128.1, 127.9, 127.8, 127.7, 102.7, 99.7, 81.0, 75.2×2, 74.6, 73.8×2, 73.4, 72.4, 71.9, 69.0, 68.8, 67.7×2, 60.8; HRMS (ESI-TOF) 645.2316 (645.2312 calcd for $C_{34}H_{38}O_{11}Na$ $[M+Na]^+$).

2,6-Di-*O*-acetyl-4-*O*-(2,3,4,6-tetra-*O*-acetyl-β-D-mannopyranosyl)-D-*myo*-inositol-1,3,5-orthoformate (95)

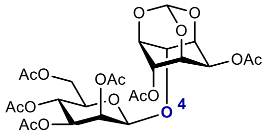

Acetylated
β-D-mannoside **95**

To a solution of **100** (10.1 mg, 16.2 μmol) in THF (1.62 mL) was added 20% $Pd(OH)_2$/C (10.1 mg, wetted with 50% water) at room temperature under Ar atmosphere. After changing the atmosphere to H_2 (balloon), the reaction mixture was stirred for 1 h. After changing the atmosphere to Ar, the reaction was filtered through celite pad, and the filtrate was concentrated in *vacuo*. To a solution of the residue in pyridine (405 μL) was added Ac_2O (405 μL) at room temperature. After the reaction mixture was stirred for 7 h at room temperature, the reaction was quenched by addition of H_2O (2 mL). The resultant mixture was extracted with EtOAc (5 mL×3), and then the extracts were

washed with brine (5 mL), dried over anhydrous Na_2SO_4, and concentrated in *vacuo*. The residue was subjected to silica gel column chromatography (2/1 *n*-hexane/EtOAc) to give **95** (7.8 mg, 12.9 μmol, 80% yield in 2 steps).

Data for **95**: Colorless syrup; R_f 0.26 (1/1 *n*-hexane/EtOAc); $[\alpha]^{27}_{D}$ −33.9° (*c* 0.76, $CHCl_3$); ^{1}H-NMR (500 MHz, $CDCl_3$) δ 5.55 (1H, d, *J*=1.5 Hz), 5.53 (1H, dd, *J*=1.0 Hz, *J*=3.0 Hz), 5.48 (1H, m), 5.16 (1H, dd, *J*=10.0 Hz, *J*=10.0 Hz), 5.14 (1H, m), 5.06 (1H, dd, *J*=3.0 Hz, *J*=10.0 Hz), 4.81 (1H, d, *J*=1.0 Hz), 4.69 (1H, m), 4.53 (1H, m), 4.27-4.22 (3H, m), 4.05 (1H, dd, *J*=2.0 Hz, *J*=12.0 Hz), 3.68 (1H, m), 2.21 (3H, s), 2.18 (3H, s), 2.16 (3H, s), 2.12 (3H, s), 2.05 (3H, s), 2.00 (3H, s); ^{13}C-NMR (125 MHz, $CDCl_3$) δ 170.6, 170.3, 169.9×2, 169.5, 169.4, 102.9, 99.5, 75.5, 72.7, 70.6, 69.7, 69.4, 68.7, 67.8, 67.2, 65.5, 63.0, 62.4, 21.1, 20.9, 20.7×2, 20.6, 20.5; HRMS (ESI-TOF) 605.1714 (605.1718 calcd for $C_{25}H_{33}O_{17}$ $[M+H]^+$).

Determination of glycosylation site of 43

Comparison of NMR data of acetylated β-D-mannosides **95**

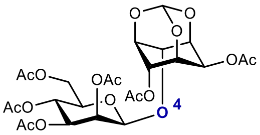

Acetylated
β(1,4)-D-mannoside **95**

Supplementary Table 22. Comparison of ^{1}H-NMR data of acetylated β-D-mannosides 95.

δ (Structurally defined acetylated β(1,4)-D-mannoside **95**)<br>(500 MHz, $CDCl_3$)	δ (Acetylated β-D-mannoside **95** derived from **43**)<br>(500 MHz, $CDCl_3$)
5.55 (d, *J*=1.5 Hz)	5.55 (d, *J*=1.5 Hz)
5.53 (dd, *J*=1.0, 3.0 Hz)	5.53 (dd, *J*=1.0, 3.0 Hz)
5.48 (m)	5.48 (m)
5.16 (dd, *J*=10.0, 10.0 Hz)	5.16 (dd, *J*=10.0, 10.0 Hz)
5.14 (m)	5.14 (m)
5.06 (dd, *J*=3.0, 10.0 Hz)	5.06 (dd, *J*=3.0, 10.0 Hz)
4.81 (d, *J*=1.0 Hz)	4.81 (d, *J*=1.0 Hz)
4.69 (m)	4.69 (m)
4.53 (m)	4.53 (m)
4.27-4.22 (m)	4.27-4.22 (m)
4.05 (dd, *J*=2.0, 12.0 Hz)	4.05 (dd, *J*=2.0, 12.0 Hz)
3.68 (m)	3.68 (m)
2.21 (s)	2.21 (s)
2.18 (s)	2.18 (s)
2.16 (s)	2.16 (s)
2.12 (s)	2.12 (s)

2.05 (s)	2.06 (s)
2.00 (s)	2.00 (s)

Supplementary Table 23. Comparison of ^{13}C-NMR data of acetylated β-D-mannosides 95.

<table>
<tr><th>δ (Structurally defined acetylated β(1,4)-D-mannoside 95)
(125 MHz, $CDCl_3$)</th><th>δ (Acetylated β-D-mannoside 95 derived from 43)
(125 MHz, $CDCl_3$)</th></tr>
<tr><td>170.6</td><td>170.6</td></tr>
<tr><td>170.3</td><td>170.3</td></tr>
<tr><td>169.9×2</td><td>169.9×2</td></tr>
<tr><td>169.5</td><td>169.5</td></tr>
<tr><td>169.4</td><td>169.4</td></tr>
<tr><td>102.9</td><td>102.9</td></tr>
<tr><td>99.5</td><td>99.5</td></tr>
<tr><td>75.5</td><td>75.5</td></tr>
<tr><td>72.7</td><td>72.7</td></tr>
<tr><td>70.6</td><td>70.6</td></tr>
<tr><td>69.7</td><td>69.7</td></tr>
<tr><td>69.4</td><td>69.4</td></tr>
<tr><td>68.7</td><td>68.7</td></tr>
<tr><td>67.8</td><td>67.8</td></tr>
<tr><td>67.2</td><td>67.2</td></tr>
<tr><td>65.5</td><td>65.5</td></tr>
<tr><td>63.0</td><td>63.0</td></tr>
<tr><td>62.4</td><td>62.4</td></tr>
<tr><td>21.1</td><td>21.1</td></tr>
<tr><td>20.9</td><td>20.9</td></tr>
<tr><td>20.8</td><td rowspan="2">20.7×2</td></tr>
<tr><td>20.7</td></tr>
<tr><td>20.6</td><td>20.6</td></tr>
<tr><td>20.5</td><td>20.5</td></tr>
</table>

Determination of glycosylation site of α(1,4)-L-fucoside 44.

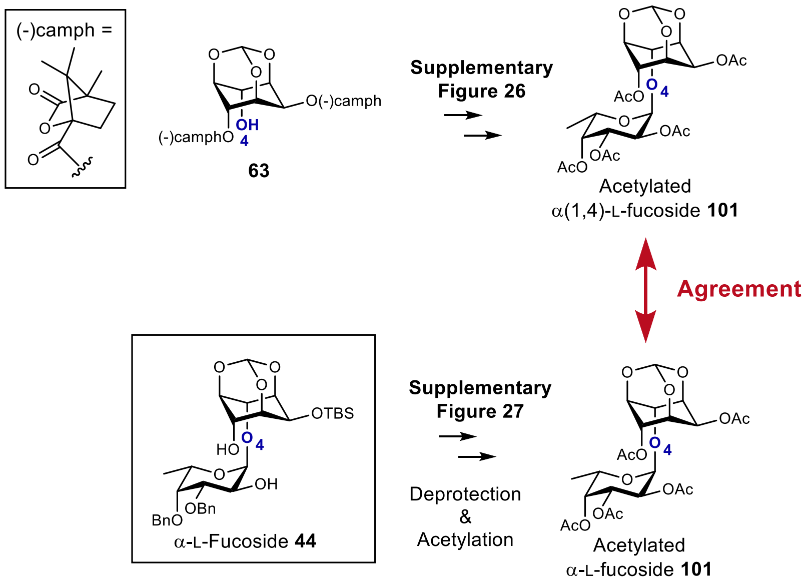

Supplementary Figure 25. Determination of glycosylation site of α-L-fucoside 44 synthesized by our desymmetric glycosylation of *meso*-diol 8.

Synthesis of acetylated α(1,4)-L-fucoside **101** from optically pure inositol **63**.

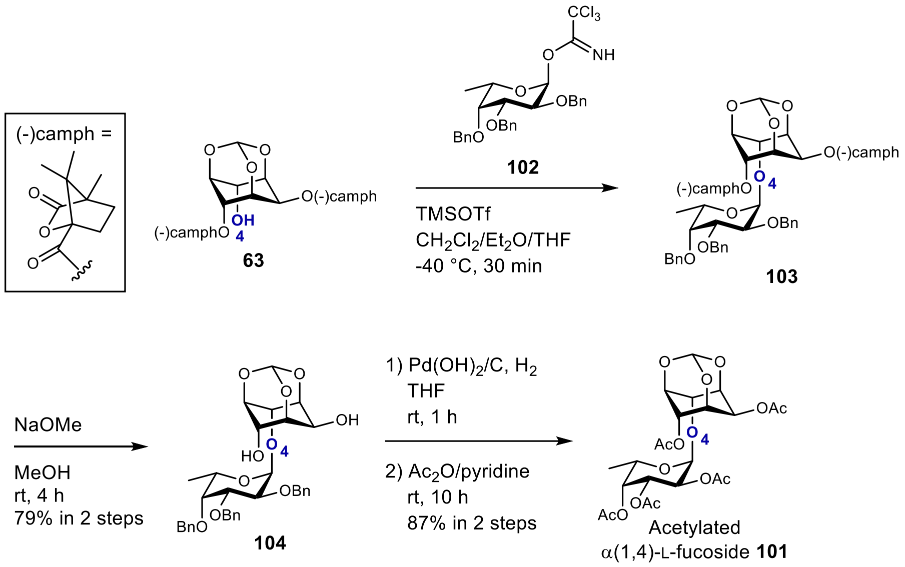

Supplementary Figure 26. Synthesis of acetylated α(1,4)-L-fucoside 101 from optically pure inositol 63.

4-*O*-(2,3,4-Tri-*O*-benzyl-α-L-fucopyranosyl)-D-*myo*-inositol-1,3,5-orthoformate (104)

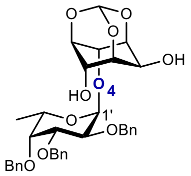

104

To a solution of **102**[18] (64.0 mg, 0.111 mmol) and **63**[13] (40.6 mg, 73.7 μmol) in dry THF-CH_2Cl_2-Et_2O (2/1/1, v/v/v, 1.11 mL) was added TMSOTf (2.00 μL, 11.1 μmol) at −40 °C. After the reaction mixture was stirred for 30 min at −40 °C, the reaction was quenched by addition of sat. $NaHCO_3$ aq. (1 mL). The resultant mixture was extracted with EtOAc (5 mL×3), and then the extracts were washed with brine (5 mL), dried over anhydrous Na_2SO_4, and concentrated in *vacuo*. The residue was subjected to silica gel column chromatography (5/1 PhMe/EtOAc) to give crude **103** (73.2 mg).

To a solution of crude **103** (73.2 mg) in MeOH (737 μL) was added NaOMe (142 μL, 0.737 mmol, 28% in MeOH) at room temperature. After the reaction mixture was stirred for 4 h at room temperature, the reaction was quenched by addition of sat. NH_4Cl aq. (1 mL). The resultant mixture was extracted with EtOAc (5 mL×3), and then the extracts were washed with brine (5 mL), dried over anhydrous Na_2SO_4, and concentrated in *vacuo*. The residue was subjected to silica gel column chromatography (3/1 PhMe/EtOAc) to give **104** (35.5 mg, 58.5 μmol, 79% yield in 2 steps).

Data for **104**: Colorless syrup; R_f 0.42 (2/1 PhMe/EtOAc); $[\alpha]^{26}_D$ −50.3° (*c* 1.0, $CHCl_3$); ^{1}H-NMR (500 MHz, $CDCl_3$) δ 7.36-7.29 (15H, m), 5.46 (1H, s), 4.97 and 4.64 (2H, ABq, *J*=11.5 Hz), 4.85 and 4.63 (2H, ABq, *J*=11.5 Hz), 4.84 (1H, d, *J*=4.0 Hz, H-1'), 4.76 and 4.71 (2H, ABq, *J*=11.5 Hz), 4.62 (1H, m), 4.44 (1H, m), 4.26 (1H, m), 4.21 (1H, m), 4.17 (1H, m), 4.09 (1H, br-d, *J*=11.5 Hz), 4.05 (1H, dd, *J*=4.0 Hz, *J*=10.0 Hz), 3.83 (1H, m), 3.82 (1H, d, *J*=11.5 Hz), 3.73 (1H, d, *J*=10.0 Hz), 3.65 (1H, br-s), 2.97 (1H, d, *J*=11.5 Hz), 1.15 (3H, d, *J*=7.0 Hz); ^{13}C-NMR (125 MHz, $CDCl_3$) δ 138.2, 137.8, 128.6, 128.5, 128.3×2, 128.1, 128.0, 127.8, 127.7, 127.5, 102.8, 94.9, 79.2, 76.9, 74.9×2, 74.8, 74.3, 73.0, 71.2, 69.6, 68.2, 67.9, 67.7, 60.2, 16.5; HRMS (ESI-TOF) 645.2073 (645.2102 calcd for $C_{34}H_{33}O_{10}K$ $[M+K]^+$).

2,6-Di-*O*-acetyl-4-*O*-(2,3,4-tri-*O*-acetyl-α-L-fucopyranosyl)-D-*myo*-inositol-1,3,5-orthoformate (101)

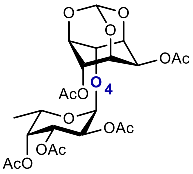

Acetylated
α(1,4)-L-fucoside **101**

To a solution of **104** (35.5 mg, 58.5 μmol) in THF (5.85 mL) was added 20% $Pd(OH)_2/C$ (35.5 mg, wetted with 50% water) at room temperature under Ar atmosphere. After changing the atmosphere to H_2 (balloon), the reaction mixture was stirred for 1 h. After changing the atmosphere to Ar, the reaction was filtered through celite pad, and the filtrate was concentrated in *vacuo*. To a solution of the residue in pyridine (1.48 mL) was added Ac_2O (1.48 mL) at room temperature. After the reaction mixture was stirred for 10 h at room temperature, the reaction was quenched by addition of H_2O (5 mL). The resultant mixture was extracted with EtOAc (10 mL×3), and then the extracts were washed with brine (10 mL), dried over anhydrous Na_2SO_4, and concentrated in *vacuo*. The residue was subjected to silica gel column chromatography (2/1 *n*-hexane/EtOAc) to give **101** (27.9 mg, 51.1 μmol, 87% yield in 2 steps).

Data for **101**: Colorless syrup; R_f 0.46 (1/1 *n*-hexane/EtOAc); $[\alpha]^{25}_D$ −108.4° (*c* 2.69, $CHCl_3$); ^{1}H-NMR (500 MHz, $CDCl_3$) δ 5.58 (1H, d, *J*=1.0 Hz), 5.47 (1H, m), 5.26 (1H, m), 5.24-5.22 (2H, m), 5.17 (1H, m), 5,14 (1H, dd, *J*=4.0 Hz, *J*=9.5 Hz), 4.60 (1H, m), 4.49 (1H, m), 4.32 (1H, m), 4.29 (1H, m), 4.09 (1H, q, *J*=6.5 Hz), 2.20 (6H, s), 2.18 (3H, s), 2.11 (3H, s), 1.98 (3H, s), 1.17 (3H, d, *J*=6.5 Hz); ^{13}C-NMR (125 MHz, $CDCl_3$) δ 170.6, 170.5, 170.2, 170.0, 169.9, 103.0, 94.8, 70.6, 70.1, 69.4, 68.7, 68.0, 67.9, 67.5, 67.4, 65.1, 63.0, 21.0, 20.7, 20.6, 15.9; HRMS (ESI-TOF) 547.1649 (547.1663 calcd for $C_{23}H_{31}O_{15}$ $[M+H]^+$).

Synthesis of acetylated α-L-fucoside **101** from **44**.

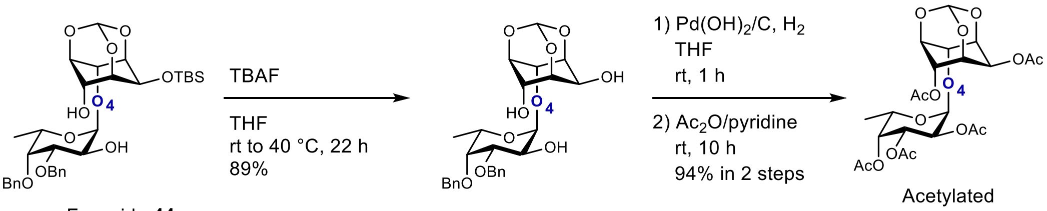

α-L-Fucoside **44** **105** Acetylated α-L-fucoside **101**

Supplementary Figure 27. Synthesis of acetylated α-L-fucoside 101 from 44.

4-*O*-(3,4-Di-*O*-benzyl-α-L-fucopyranosyl)-D-*myo*-inositol-1,3,5-orthoformate (105)

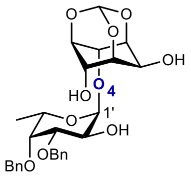

105

To a solution of **44** (15.7 mg, 24.9 μmol) in THF (498 μL) was added TBAF (49.8 μL, 49.8 μmol, 1.0 M in THF) at room temperature. The reaction mixture was stirred for 7 h and then was warmed to 40 °C. After being stirred for 15 h at 40 °C, the reaction mixture was concentrated in *vacuo*. The residue was subjected to silica gel column chromatography (1/1 PhMe/EtOAc) to give **105** (11.5 mg, 22.3 μmol, 89% yield).

Data for **105** Colorless syrup; R_f 0.22 (1/1 PhMe/EtOAc); $[\alpha]^{25}_D$ −118.1° (*c* 1.0, $CHCl_3$); ^{1}H-NMR (500 MHz, $CDCl_3$) δ 7.39-7.26 (10H, m), 5.48 (1H, s), 5.06 (1H, d, *J*=3.5 Hz, H-1'), 4.89 and 4.63 (2H, ABq, *J*=11.5 Hz), 4.74 and 4.55 (2H, ABq, *J*=11.0 Hz), 4.69 (1H, m), 4.46 (1H, m), 4.39 (1H, m), 4.25 (1H, m), 4.22-4.15 (3H, m), 3.91 (1H, q, *J*=6.5 Hz), 3.84 (1H, d, *J*=11.0 Hz), 3.71 (1H, s), 3.61 (1H, br-d, *J*=10.0 Hz), 3.15 (1H, d, *J*=12.0 Hz), 2.43 (1H, br-s), 1.22 (3H, d, *J*=6.5 Hz); ^{13}C-NMR (125 MHz, $CDCl_3$) δ 138.0, 137.4, 128.7, 128.4, 128.2×2, 127.9, 127.7, 102.8, 95.1, 79.5, 75.7, 74.9×2, 72.3, 71.2, 69.7, 68.3, 68.0, 67.1, 60.2, 16.8; HRMS (ESI-TOF) 539.1896 (539.1893 calcd for $C_{27}H_{32}O_{10}Na$ $[M+Na]^+$).

2,6-Di-*O*-acetyl-4-*O*-(2,3,4-tri-*O*-acetyl-α-L-fucopyranosyl)-D-*myo*-inositol-1,3,5-orthoformate (101)

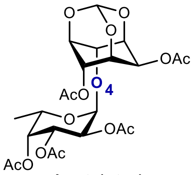

Acetylated
α-L-fucoside **101**

To a solution of **105** (10.3 mg, 19.9 μmol) in THF (1.99 mL) was added 20% $Pd(OH)_2/C$ (10.3 mg, wetted with 50% water) at room temperature under Ar atmosphere. After changing the atmosphere to H_2 (balloon), the reaction mixture was stirred for 1 h. After changing the atmosphere to Ar, the reaction was filtered through celite pad, and the filtrate was concentrated in *vacuo*. To a solution of the residue in pyridine (500 μL) was added Ac_2O (500 μL) at room temperature. After the reaction mixture was stirred for 10 h at room temperature, the reaction was quenched by addition of H_2O (2 mL). The resultant mixture was extracted with EtOAc (5 mL×3), and then the extracts were

washed with brine (5 mL), dried over anhydrous Na_2SO_4, and concentrated in *vacuo*. The residue was subjected to silica gel column chromatography (2/1 *n*-hexane/EtOAc) to give **101** (10.3 mg, 18.8 μmol, 94% yield in 2 steps).

Data for **101**: Colorless syrup; R_f 0.46 (1/1 *n*-hexane/EtOAc); $[\alpha]^{27}_D$ −111.7° (*c* 1.0, $CHCl_3$); ^{1}H-NMR (500 MHz, $CDCl_3$) δ 5.58 (1H, d, *J*=1.0 Hz), 5.47 (1H, m), 5.26 (1H, m), 5.24-5.22 (2H, m), 5.17 (1H, m), 5,14 (1H, dd, *J*=4.0 Hz, *J*=9.5 Hz), 4.60 (1H, m), 4.49 (1H, m), 4.32 (1H, m), 4.29 (1H, m), 4.09 (1H, q, *J*=6.5 Hz), 2.20 (6H, s), 2.18 (3H, s), 2.11 (3H, s), 1.98 (3H, s), 1.17 (3H, d, *J*=6.5 Hz); ^{13}C-NMR (125 MHz, $CDCl_3$) δ 170.6, 170.5, 170.3, 170.0×2, 103.0, 94.8, 70.7, 70.1, 69.4, 68.7, 68.0, 67.9, 67.5, 67.4, 65.1, 63.0, 21.0, 20.7, 20.6, 15.9; HRMS (ESI-TOF) 547.1669 (547.1663 calcd for $C_{23}H_{31}O_{15}$ $[M+H]^+$).

Determination of glycosylation site of 44

Comparison of NMR data of acetylated α-L-fucosides **101**

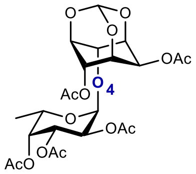

Acetylated
α(1,4)-L-fucoside **101**

Supplementary Table 24. Comparison of ^{1}H-NMR data of acetylated α-L-fucosides 101.

δ(Structurally defined acetylated α(1,4)-L-fucoside **101**) (500 MHz, $CDCl_3$)	δ(Acetylated α-L-fucoside **101** derived from **44**) (500 MHz, $CDCl_3$)
5.58 (d, *J*=1.0 Hz)	5.58 (d, *J*=1.0 Hz)
5.47 (m)	5.47 (m)
5.26 (m)	5.26 (m)
5.24-5.22 (m)	5.24-5.22 (m)
5.17 (m)	5.17 (m)
5,14 (dd, *J*=4.0, 9.5 Hz)	5,14 (dd, *J*=4.0, 9.5 Hz)
4.60 (m),	4.60 (m),
4.49 (m)	4.49 (m)
4.32 (m)	4.32 (m)
4.29 (m)	4.29 (m)
4.09 (q, *J*=6.5 Hz)	4.09 (q, *J*=6.5 Hz)
2.20 (s)	2.20 (s)
2.18 (s),	2.18 (s),
2.11 (s)	2.11 (s)
1.98 (s),	1.98 (s),
1.17 (d, *J*=6.5 Hz)	1.17 (d, *J*=6.5 Hz)

Supplementary Table 25. Comparison of ^{13}C-NMR data of acetylated α-L-fucosides 101.

δ (Structurally defined acetylated α(1,4)-L-fucoside **101**) (125 MHz, $CDCl_3$)	δ (Acetylated α-L-fucoside **101** derived from **44**) (125 MHz, $CDCl_3$)
170.6	170.6
170.5	170.5
170.2	170.3
170.0	170.0×2
169.9	
103.0	103.0
94.8	94.8
70.6	70.7
70.1	70.1
69.4	69.4
68.7	68.7
68.0	68.0
67.9	67.9
67.5	67.5
67.4	67.4
65.1	65.1
63.0	63.0
21.0	21.0
20.7	20.7
20.6	20.6
15.9	15.9

Determination of glycosylation site of β(1,4)-D-mannoside 57.

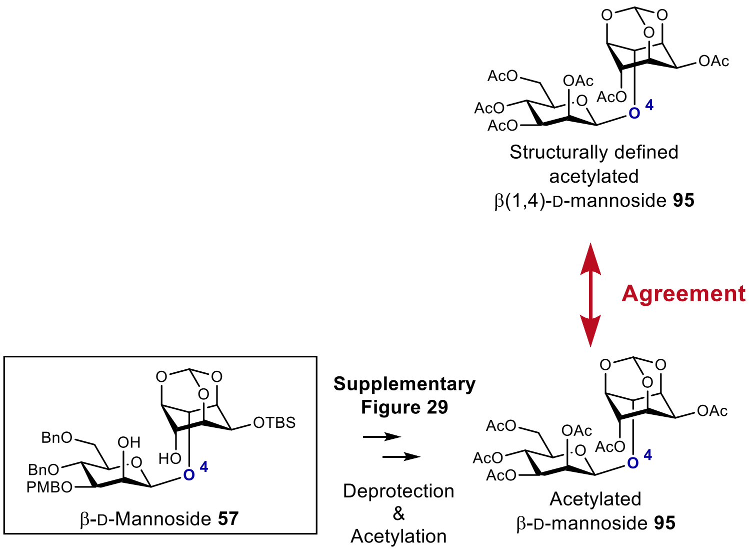

Supplementary Figure 28. Determination of glycosylation site of β-D-mannoside 57.

Synthesis of acetylated β-D-mannoside **95** from **57**.

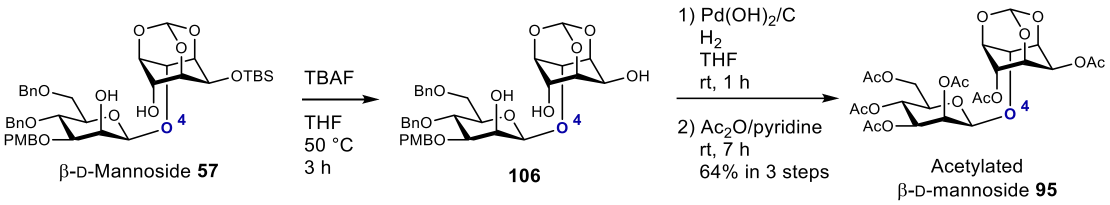

Supplementary Figure 29. Synthesis of acetylated β-D-mannoside 95 from 57.

2,6-Di-*O*-acetyl-4-*O*-(2,3,4,6-tetra-*O*-acetyl-β-D-mannopyranosyl)-D-*myo*-inositol-1,3,5-orthoformate (95)

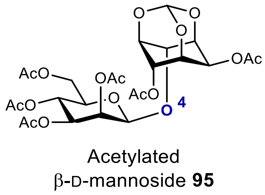

To a solution of **57** (17.8 mg, 23.2 μmol) in THF (464 μL) was added TBAF (46.4 μL, 46.4 μmol, 1.0 M in THF) at room temperature. After the reaction mixture was stirred for 3 h at 50 °C, the reaction mixture was concentrated in *vacuo*. The residue was subjected to silica gel column chromatography (3/2 PhMe/acetone) to give crude **106** (15.0 mg).

To a solution of crude **106** (15.0 mg) in THF (2.30 mL) was added 20% $Pd(OH)_2/C$ (15.0 mg, wetted with 50% water) at room temperature under Ar atmosphere. After changing the atmosphere to H_2 (balloon), the reaction mixture was stirred for 1 h. After changing the atmosphere to Ar, the reaction was filtered through celite pad, and the filtrate was concentrated in *vacuo*. To a solution of the residue in pyridine (575 μL) was added Ac_2O (575 μL) at room temperature. After the reaction mixture was stirred for 7 h at room temperature, the reaction was quenched by addition of H_2O (1 mL). The resultant mixture was extracted with EtOAc (5 mL×3), and then the extracts were washed with brine (5 mL), dried over anhydrous Na_2SO_4, and concentrated in *vacuo*. The residue was subjected to silica gel column chromatography (2/1 *n*-hexane/EtOAc) to give **95** (9.0 mg, 14.9 μmol, 64% yield in 3 steps).

Data for **95**: Colorless syrup; R_f 0.26 (1/1 *n*-hexane/EtOAc); $[\alpha]^{26}_D$ −32.4° (*c* 0.85, $CHCl_3$); ^{1}H-NMR (500 MHz, $CDCl_3$) δ 5.56 (1H, d, *J*=1.0 Hz), 5.53 (1H, dd, *J*=1.0 Hz, *J*=3.5 Hz), 5.48 (1H, m), 5.16 (1H, dd, *J*=10.0 Hz, *J*=10.0 Hz), 5.14 (1H, m), 5.06 (1H, dd, *J*=3.5 Hz, *J*=10.0 Hz), 4.81 (1H, d, *J*=1.0 Hz), 4.69 (1H, m), 4.53 (1H, m), 4.27-4.22 (3H, m), 4.05 (1H, dd, *J*=2.0 Hz, *J*=12.0 Hz), 3.68 (1H, m), 2.21 (3H, s), 2.19 (3H, s), 2.17 (3H, s), 2.12 (3H, s), 2.05 (3H, s), 2.00 (3H, s); ^{13}C-NMR (125

MHz, $CDCl_3$) δ 170.6, 170.3, 169.9×2, 169.5, 169.4, 102.9, 99.5, 75.5, 72.7, 70.6, 69.7, 69.4, 68.7, 67.8, 67.2, 65.5, 63.0, 62.4, 21.1, 20.9, 20.8, 20.7×2, 20.5; HRMS (ESI-TOF) 627.1533 (627.1537 calcd for $C_{25}H_{32}O_{17}Na$ $[M+Na]^+$).

Determination of glycosylation site of 57

Comparison of NMR data of acetylated β-D-mannosides **95**

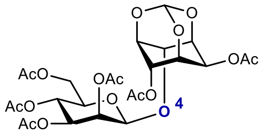

Acetylated
β(1,4)-D-mannoside **95**

Supplementary Table 26. Comparison of ^{1}H-NMR data of acetylated β-D-mannosides 95.

δ (Structurally defined acetylated β(1,4)-D-mannoside **95**) (500 MHz, $CDCl_3$)	δ (Acetylated β-D-mannoside **95** derived from **57**) (500 MHz, $CDCl_3$)
5.55 (d, *J*=1.5 Hz)	5.56 (d, *J*=1.0 Hz)
5.53 (dd, *J*=1.0, 3.0 Hz)	5.53 (dd, *J*=1.0, 3.5 Hz)
5.48 (m)	5.48 (m)
5.16 (dd, *J*=10.0, 10.0 Hz)	5.16 (dd, *J*=10.0, 10.0 Hz)
5.14 (m)	5.14 (m)
5.06 (dd, *J*=3.0, 10.0 Hz)	5.06 (dd, *J*=3.5, 10.0 Hz)
4.81 (d, *J*=1.0 Hz)	4.81 (d, *J*=1.0 Hz)
4.69 (m)	4.69 (m)
4.53 (m)	4.53 (m)
4.27-4.22 (m)	4.27-4.22 (m)
4.05 (dd, *J*=2.0, 12.0 Hz)	4.05 (dd, *J*=2.0, 12.0 Hz)
3.68 (m)	3.68 (m)
2.21 (s)	2.21 (s)
2.18 (s)	2.19 (s)
2.16 (s)	2.17 (s)
2.12 (s)	2.12 (s)
2.05 (s)	2.05 (s)
2.00 (s)	2.00 (s)

Supplementary Table 27. Comparison of ^{13}C-NMR data of acetylated β-D-mannosides 95.

δ (Structurally defined acetylated β(1,4)-D-mannoside **95**) (125 MHz, $CDCl_3$)	δ (Acetylated β-D-mannoside **95** derived from **57**) (125 MHz, $CDCl_3$)
170.6	170.6
170.3	170.3
169.9×2	169.9×2
169.5	169.5
169.4	169.4
102.9	102.9
99.5	99.5
75.5	75.5

72.7	72.7
70.6	70.6
69.7	69.7
69.4	69.4
68.7	68.7
67.8	67.8
67.2	67.2
65.5	65.5
63.0	63.0
62.4	62.4
21.1	21.1
20.9	20.9
20.8	20.8
20.7	20.7×2
20.6	
20.5	20.5

Determination of glycosylation site of 46 and 47.

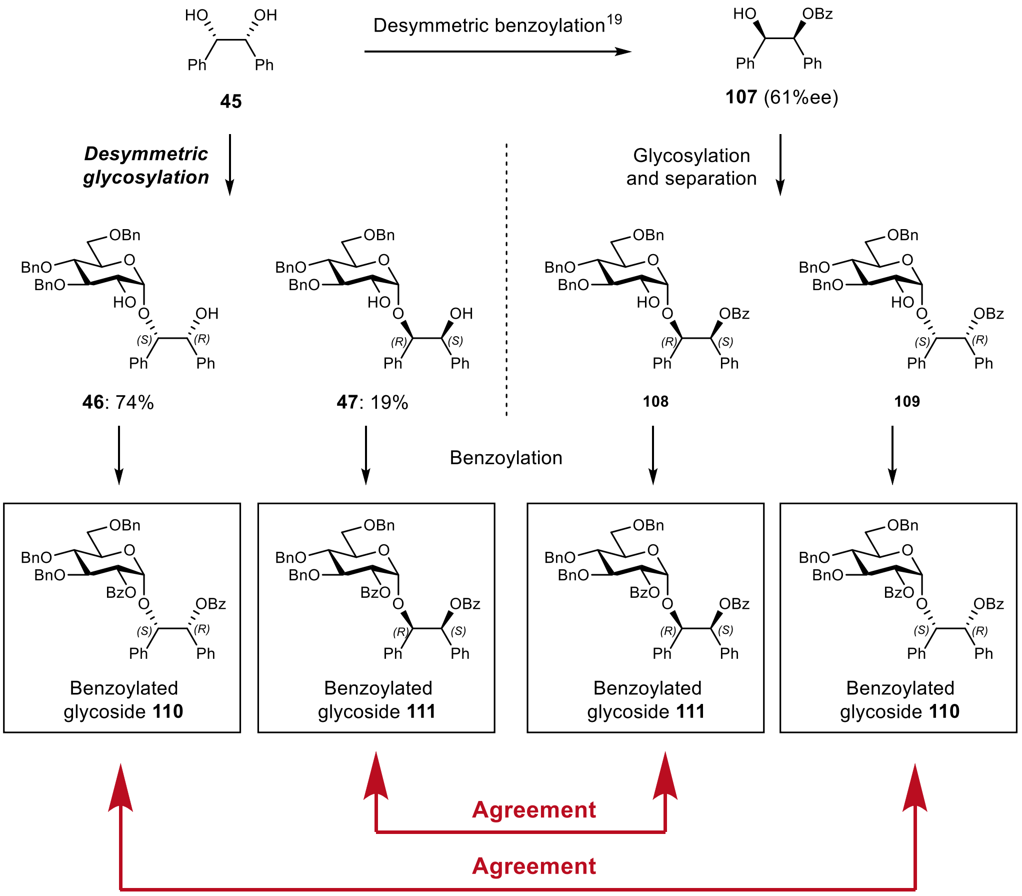

Supplementary Figure 30. Determination of glycosylation site of 46 and 47 synthesized by our desymmetric glycosylation of *meso*-diol 45.

Synthesis of benzoylated glycoside **110** from **46**.

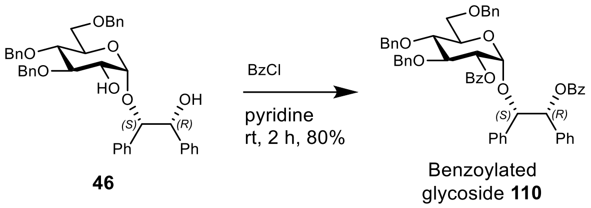

Supplementary Figure 31. Synthesis of benzoylated glycoside 110 from 46.

(1*R*, 2*S*)-2-*O*-(2-*O*-Benzoyl-3,4,6-tri-*O*-benzyl-α-D-glucopyranosyl)-1,2-diphenylethyl benzoate (110)

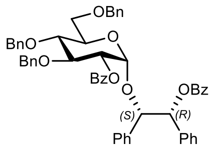

To a solution of **46** (16.2 mg, 25.0 μmol) in pyridine (500 μL) was added benzoyl chloride (23.2 μL, 0.200 mmol) at 0 °C under Ar atmosphere. After the reaction mixture was stirred for 2 h at rt, the reaction was quenched by addition of H_2O (1 mL). The resultant mixture was extracted with EtOAc (1 mL×3), and then the extracts were washed with brine (1 mL), dried over anhydrous Na_2SO_4, and concentrated in *vacuo*. The residue was subjected to silica gel column chromatography (4/1 *n*-hexane/EtOAc) to give **110** (17.1 mg, 20.0 μmol, 80% yield).

Data for **110**: Colorless foam; R_f 0.63 (2/1 *n*-hexane/EtOAc); $[\alpha]^{23}_D$ = +87.1° (*c* 1.0, $CHCl_3$); ^{1}H-NMR (400 MHz, $CDCl_3$) δ 7.96 (2H, m), 7.91 (2H, m), 7.60 (1H, m), 7.50 (1H, m), 7.45-7.39 (2H, m), 7.37-7.18 (22H, m), 7.07-7.01 (3H, m), 6.85-6.79 (2H, m), 6.08 (1H, d, *J*=6.0 Hz), 5.07 (1H, d, *J*=6.0 Hz), 5.04 (1H, dd, *J*=3.6 Hz, *J*=9.6 Hz), 4.91 (1H, d, $J_{1,2}$=3.6 Hz, H-1), 4.85-4.77 (3H, m), 4.55 and 4.37 (2H, ABq, *J* = 12.0 Hz), 4.47 (1H, ABq, *J* = 10.8 Hz), 4.17 (1H, dd, *J*= 9.6 Hz, *J*= 9.6 Hz), 3.71 (1H, dd, *J*=9.6 Hz, *J*=9.6 Hz), 3.41 (1H, dd, *J*=2.8 Hz, *J*=10.4 Hz), 3.34 (1H, dd, *J*=1.6 Hz, *J*=10.4 Hz), 3.27 (1H, m); ^{13}C-NMR (125 MHz, $CDCl_3$) δ 165.2, 165.0, 138.6, 138.1, 137.9, 137.0, 136.1, 133.1, 133.0, 129.9, 129.7, 129.6, 128.4×2, 128.3×2, 128.2×2, 128.1, 128.0×2, 127.9×2, 127.7, 127.6×2, 127.5×2, 93.0, 79.4, 79.2, 78.4, 77.5, 75.1, 74.5, 73.4, 73.2, 70.7, 68.0; HRMS (ESI-TOF) *m/z* 893.3111 (893.3092 calcd for $C_{55}H_{50}O_9K$, $[M+K]^+$).

Synthesis of benzoylated glycoside **111** from **47**.

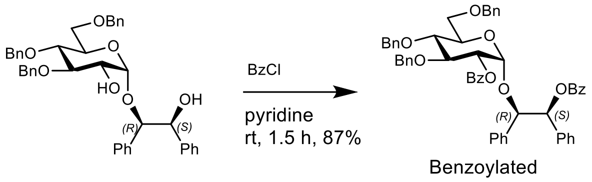

Supplementary Figure 32. Synthesis of benzoylated glycoside 111 from 47.

(1*S*, 2*R*)-2-*O*-(2-*O*-Benzoyl-3,4,6-tri-*O*-benzyl-α-D-glucopyranosyl)-1,2-diphenylethyl benzoate (111)

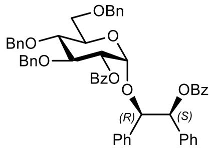

To a solution of **47** (10.1 mg, 15.6 μmol) in pyridine (156 μL) was added benzoyl chloride (14.5 μL, 0.125 mmol) at 0 ºC under Ar atmosphere. After the reaction mixture was stirred for 1.5 h at rt, the reaction was quenched by addition of sat. $NaHCO_3$ aq. (1 mL). The resultant mixture was extracted with EtOAc (1 mL×3), and then the extracts were washed with brine (1 mL), dried over anhydrous Na_2SO_4, and concentrated in *vacuo*. Purification of the residue by preparative TLC (3/1 *n*-hexane/EtOAc) gave **111** (11.6 mg, 13.6 μmol, 87% yield).

Data for **111**: Colorless foam; R_f 0.36 (3/1 *n*-hexane/EtOAc); $[\alpha]^{25}_{D}$ = +102.6° (*c* 1.0, $CHCl_3$); ^{1}H-NMR (400 MHz, $CDCl_3$) δ 8.05 (2H, m), 7.78 (2H, m), 7.59 (1H, m), 7.51-7.41 (3H, m), 7.33-7.15 (22H, m), 7.10-7.04 (3H, m), 6.97-6.91 (2H, m), 6.11 (1H, d, *J*=5.2 Hz), 5.34 (1H, d, $J_{1,2}$=3.6 Hz, H-1), 5.14 (1H, dd, *J*=3.6 Hz, *J*=10.0 Hz), 5.11 (1H, d, *J*=5.2 Hz), 4.79 (2H, s), 4.71 and 4.41 (2H, ABq, *J*=10.8 Hz), 4.49 and 4.29 (2H, ABq, *J*=12.4 Hz), 4.11 (1H, dd, *J*=10.0 Hz, *J*=9.6 Hz), 3.71 (1H, dd, *J*=9.6 Hz, *J*=9.6 Hz), 3.45 (1H, dd, *J*=4.0 Hz, *J*=10.8 Hz), 3.38 (1H, m), 3.13 (1H, dd, *J*=1.2 Hz, *J*=10.8 Hz); ^{13}C-NMR (125 MHz, $CDCl_3$) δ 165.6, 165.0, 138.1×2, 137.9×2, 136,2, 133.2, 132.9, 129.9, 129.7×2, 129.5, 128.4, 128.3×2, 128.2, 128.1, 128.0×2, 127.9, 127.8×2, 127.7, 127.6, 127.5, 97.3, 81.3, 80.0, 77.9, 77.7, 75.5, 75.1, 73.8, 73.1, 71.0, 67.6; HRMS (ESI-TOF) *m/z* 893.3059 (893.3092 calcd for $C_{55}H_{50}O_9K$ $[M+K]^+$).

Synthesis of **108** and **109** from **S47**.

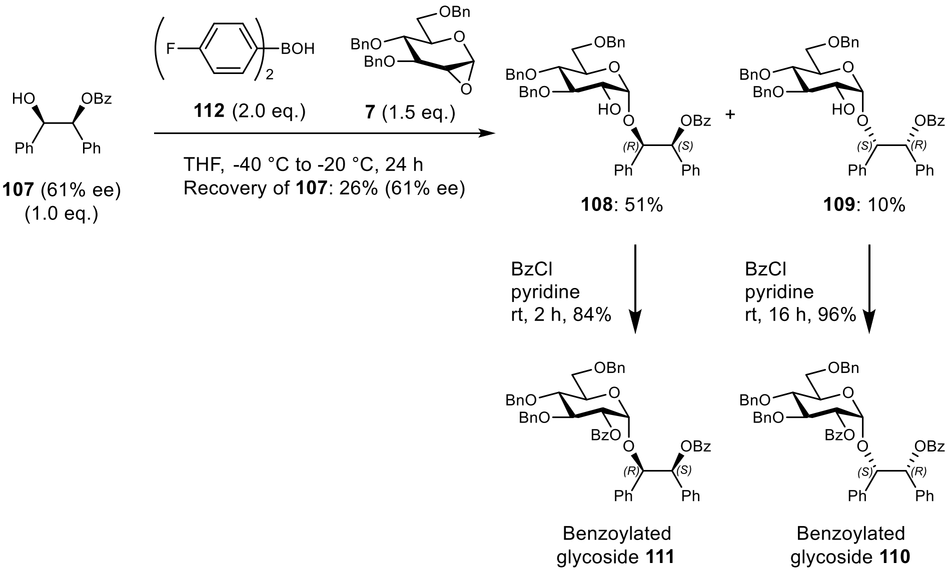

Supplementary Figure 33. Synthesis of benzoylated glycosides 111 and 110 from 107.

(1*S*, 2*R*)-2-*O*-(3,4,6-tri-*O*-Benzyl-α-D-glucopyranosyl)-1,2-diphenylethyl benzoate (108) and (1*R*, 2*S*)-2-*O*-(3,4,6-tri-*O*-Benzyl-α-D-glucopyranosyl)-1,2-diphenylethyl benzoate (109)

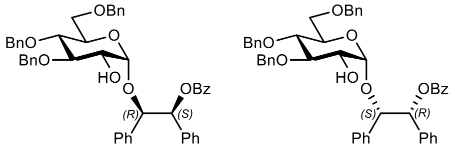

108 **109**

To a solution of **107**[19] (20.0 mg, 62.8 μmol, 61% ee) and bis(4-fluoro)phenylborinic acid (**112**[20]) (27.4 mg, 126 μmol) in dry THF (630 μL) was added a solution of **7** (54.3 mg, 126 μmol) in dry THF (630 μL) at -40 °C under Ar atmosphere. After the reaction mixture was stirred for 12 h, the reaction mixture was warmed to −20 °C. After the reaction mixture was stirred for 12 h, the reaction was quenched by addition of 0.05 M $NaBO_3$ aq. (2.76 mL, 138 μmol). To the resultant mixture was added sat. NH_4Cl aq. (2 mL) and extracted with EtOAc (3 mL×3), and then the combined extracts were washed with brine (5 mL), dried over anhydrous Na_2SO_4, and concentrated in *vacuo*. Purification of the residue by silica gel column chromatography (3/2 *n*-hexane/EtOAc) gave crude **108**, crude **109** and crude **107**. Purification of crude **108** by preparative TLC (25/1 $CHCl_3$/acetone) gave **108** (23.9 mg, 31.8 μmol, 51% yield). Purification of crude **109** by preparative TLC (40/1 PhMe/acetone) gave **109** (4.6 mg, 6.13 μmol, 10% yield). Purification of crude **107** by preparative TLC (3/1 *n*-hexane/Et_2O) gave **107** (5.1 mg, 23.4 μmol, 26% recovery yield).

Data for **108**: Colorless foam; R_f 0.58 (3/2 *n*-hexane/EtOAc); $[\alpha]^{23}_{D}$ = +86.6° (*c* 0.7, $CHCl_3$); ^{1}H-NMR (400 MHz, $CDCl_3$) δ 7.96 (2H, m), 7.55 (1H, m), 7.43-7.18 (25H, m), 7.05 (2H, m), 6.32 (1H, d, *J*=6.0 Hz), 5.06 (1H, d, $J_{1,2}$=3.2 Hz, H-1), 4.95 (1H, d, *J*=6.0 Hz), 4.95 and 4.76 (2H, ABq, *J*=11.2 Hz), 4.73 and 4.36 (2H, ABq, *J*=10.8 Hz), 4.41 and 4.21 (2H, ABq, *J*=12.4 Hz), 3.66-3.58 (2H, m), 3.52 (1H, dd, *J*=8.4 Hz, *J*=9.6 Hz), 3.42 (1H, dd, *J*=3.2 Hz, *J*=10.8 Hz), 3.37 (1H, m), 2.96 (1H, dd, *J*=2.0 Hz, *J*=10.8 Hz), 2.02 (1H, d, *J*=10.4 Hz); ^{13}C-NMR (125 MHz, $CDCl_3$) δ 165.4, 138.7, 138.0, 137.8, 137.6, 136.8, 133.2, 129.6, 128.6, 128.4, 128.3, 128.2×2, 128.0×2, 127.7×2, 127.6, 127.5×2, 127.3, 101.0, 84.4, 83.1, 77.8, 76.9, 75.1×2, 73.4, 73.1, 70.9, 67.6; HRMS (ESI-TOF) *m/z* 773.3099 (773.3090 calcd for $C_{48}H_{46}O_8Na$ $[M+Na]^+$).

Data for **109**: Colorless foam; R_f 0.54 (2/1 *n*-hexane/EtOAc); $[\alpha]^{25}_{D}$ = +86.3° (*c* 0.2, $CHCl_3$); ^{1}H-NMR (400 MHz, $CDCl_3$) δ 7.94 (2H, m), 7.50 (1H, m), 7.39-7.23 (25H, m), 7.16 (2H, m), 6.12 (1H, d, *J*=6.0 Hz), 5.10 (1H, d, *J*=6.0 Hz), 4.91 and 4.86 (2H, ABq, *J*=11.2 Hz), 4.78 and 4.42 (2H, ABq, *J*=11.2 Hz), 4.74 (1H, d, $J_{1,2}$=4.0 Hz, H-1), 4.52 and 4.35 (2H, ABq, *J*=12.0 Hz), 3.69 (1H, dd, *J*=9.2 Hz, *J*=8.8 Hz), 3.58 (1H, ddd, *J*=4.0 Hz, *J*=9.2 Hz, *J*=9.6 Hz), 3.42 (1H, dd, *J*=9.2 Hz, *J*=9.2 Hz), 3.36 (1H, dd, *J*=3.2 Hz, *J*=10.4 Hz), 3.31 (1H, dd, *J*=2.4 Hz, *J*=10.4 Hz), 3.14 (1H, m), 1.82 (1H, d, *J*=9.6 Hz); ^{13}C-NMR (125 MHz, $CDCl_3$) δ 165.1, 138.7, 138.6, 137.9, 137.0, 136.2, 133.1×2, 129.8, 129.6×2, 128.6, 128.4, 128.3×2, 128.2, 128.1, 127.8, 127.7, 127.6×2, 127.4, 127.0, 95.0, 82.7, 79.0, 78.3, 77.2, 75.1, 74.3, 73.4, 72.6, 70.7, 68.1; HRMS (ESI-TOF) *m/z* 773.3113 (773.3090 calcd for $C_{48}H_{46}O_8Na$ $[M+Na]^+$)

(1*S*, 2*R*)-2-*O*-(2-*O*-Benzoyl-3,4,6-tri-*O*-benzyl-α-D-glucopyranosyl)-1,2-diphenylethyl benzoate (111)

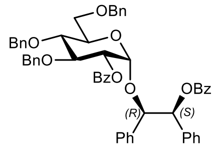

Benzoylated glycoside **111**

To a solution of **108** (4.10 mg, 5.46 μmol) in pyridine (364 μL) was added benzoyl chloride (9.43 μL, 81.9 μmol) and 4-dimethylaminopyridine (0.667 mg, 5.46 μmol) at 0 °C under Ar atmosphere. After the reaction mixture was stirred for 2 h at rt, the reaction was quenched by addition of H_2O (1 mL). The resultant mixture was extracted with EtOAc (1 mL×3), and then the extracts were washed

with brine (1 mL), dried over anhydrous Na_2SO_4, and concentrated in *vacuo*. Purification of the residue by preparative TLC (6/1 *n*-hexane/EtOAc) gave **111** (3.90 mg, 4.56 μmol, 84% yield).

Data for **111**: Colorless foam; R_f 0.53 (2/1 *n*-hexane/EtOAc); $[\alpha]^{23}_D$ = +85.8° (*c* 0.8, $CHCl_3$); ^{1}H-NMR (400 MHz, $CDCl_3$) δ 8.05 (2H, m), 7.78 (2H, m), 7.59 (1H, m), 7.51-7.41 (3H, m), 7.33-7.15 (22H, m), 7.10-7.05 (3H, m), 6.98-6.91 (2H, m), 6.11 (1H, d, *J*=5.6 Hz), 5.34 (1H, d, $J_{1,2}$=4.0 Hz, H-1), 5.14 (1H, dd, *J*=4.0 Hz, *J*=10.0 Hz), 5.11 (1H, d, *J*=5.6 Hz), 4.79 (2H, s), 4.71 and 4.41 (2H, ABq, *J*=10.8 Hz), 4.49 and 4.29 (2H, ABq, *J*=12.4 Hz), 4.11 (1H, dd, *J*=10.0 Hz, *J*=9.6 Hz), 3.70 (1H, dd, *J*=9.6 Hz, *J*=10.0 Hz), 3.45 (1H, dd, *J*=3.2 Hz, *J*=10.8 Hz), 3.38 (1H, m), 3.13 (1H, dd, *J*=1.2 Hz, *J*=10.8 Hz); HRMS (ESI-TOF) *m/z* 877.3374 (877.3353 calcd for $C_{55}H_{50}O_9Na$ $[M+Na]^+$)

(1*R*, 2*S*)-1-*O*-(2-*O*-Benzoyl-3,4,6-tri-*O*-benzyl-α-D-glucopyranosyl)-1,2-diphenylethyl benzoate (110)

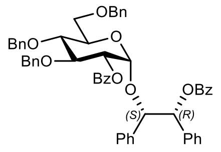

Benzoylated glycoside **110**

To a solution of **109** (9.50 mg, 12.7 μmol) in pyridine (438 μL) was added benzoyl chloride (21.8 μL, 0.191 mmol) and 4-dimethylaminopyridine (1.54 mg, 12.7 μmol) at 0 ºC under Ar atmosphere. After the reaction mixture was stirred for 16 h at rt, the reaction was quenched by addition of H_2O (1 mL). The resultant mixture was extracted with EtOAc (1 mL×3), and then the extracts were washed with brine (1 mL), dried over anhydrous Na_2SO_4, and concentrated in *vacuo*. Purification of the residue by preparative TLC (3/1 *n*-hexane/EtOAc) gave **110** (10.2 mg, 11.9 μmol, 96% yield).

Data for **110**: Colorless foam; R_f 0.71 (1/1 *n*-hexane/EtOAc); $[\alpha]^{25}_D$ = +93.8° (*c* 0.5, $CHCl_3$); ^{1}H-NMR (400 MHz, $CDCl_3$) δ 7.96 (2H, m), 7.91 (2H, m), 7.60 (1H, m), 7.50 (1H, m), 7.46-7.39 (2H, m), 7.37-7.17 (22H, m), 7.08-7.01 (3H, m), 6.85-6.78 (2H, m), 6.08 (1H, d, *J*=6.0 Hz), 5.06 (1H, d, *J*=6.0 Hz), 5.04 (1H, dd, *J*=3.6 Hz, *J*=9.6 Hz), 4.90 (1H, d, $J_{1,2}$=3.6 Hz, H-1), 4.85-4.77 (3H, m), 4.55 and 4.37 (2H, ABq, *J*=12.0 Hz), 4.47 (1H, ABq, *J*=11.6 Hz), 4.17 (1H, dd, *J*=9.6 Hz, *J*=9.6 Hz), 3.71 (1H, dd, *J*=9.6 Hz, *J*=9.6 Hz), 3.41 (1H, dd, *J*=3.2 Hz, *J*=10.4 Hz), 3.34 (1H, dd, *J*=1.6 Hz, *J*=10.8 Hz), 3.27 (1H, m); HRMS (ESI-TOF) *m/z* 877.3349 (877.3353 calcd for $C_{55}H_{50}O_9Na$ $[M+Na]^+$)

Determination of glycosylation site of 46 and **47**

Comparison of NMR data of benzoylated glycoside **110**

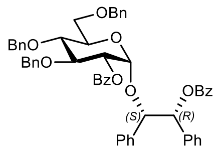

Benzoylated
glycoside **110**

Supplementary Table 28. Comparison of ^{1}H-NMR data of benzoylated glycoside 110.

δ (Structurally defined benzoylated glycoside **110**) (400 MHz, $CDCl_3$)	δ (Benzoylated glycoside **110** derived from **45**) (400 MHz, $CDCl_3$)
7.96 (m)	7.96 (m)
7.91 (m)	7.91 (m)
7.60 (m)	7.60 (m)
7.50 (m)	7.50 (m)
7.46-7.39 (m)	7.45-7.39 (m)
7.37-7.17 (m)	7.37-7.18 (m)
7.08-7.01 (m)	7.07-7.01 (m)
6.85-6.78 (m)	6.85-6.79 (m)
6.08 (d, *J*=6.0 Hz)	6.08 (d, *J*=6.0 Hz)
5.06 (d, *J*=6.0 Hz)	5.07 (d, *J*=6.0 Hz)
5.04 (dd, *J*=3.6, 9.6 Hz)	5.04 (dd, *J*=3.6, 9.6 Hz)
4.90 (d, *J*=3.6 Hz)	4.91 (d, *J*=3.6 Hz)
4.85-4.77 (m)	4.85-4.77 (m)
4.55 and 4.37 (ABq, *J*=12.0 Hz)	4.55 and 4.37 (ABq, *J*=12.0 Hz)
4.47 (d, *J*=11.6 Hz)	4.47 (d, *J*=10.8 Hz)
4.17 (dd, *J*=9.6, 9.6 Hz)	4.17 (dd, *J*=9.6, 9.6 Hz)
3.71 (dd, *J*=9.6, 9.6 Hz)	3.71 (dd, *J*=9.6, 9.6 Hz)
3.41 (dd, *J*=3.2, 10.4 Hz)	3.41 (dd, *J*=2.8, 10.4 Hz)
3.34 (dd, *J*=1.6, 10.4 Hz)	3.34 (dd, *J*=1.6, 10.4 Hz)
3.27 (m)	3.27 (m)

Comparison of NMR data of benzoylated glycoside **111**

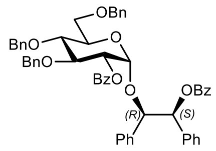

Benzoylated glycoside **111**

Supplementary Table 29. Comparison of ^{1}H-NMR data of benzoylated glycoside 111.

δ(Structurally defined benzoylated glycoside **111**) (400 MHz, $CDCl_3$)	δ(Benzoylated glycoside **111** derived from **45**) (400 MHz, $CDCl_3$)
8.05 (m)	8.05 (m)
7.78 (m)	7.78 (m)
7.59 (m)	7.59 (m)
7.51-7.41 (m)	7.51-7.41 (m)
7.33-7.15 (m)	7.33-7.15 (m)
7.10-7.05 (m)	7.10-7.04 (m)
6.98-6.91 (m)	6.97-6.91 (m)
6.11 (d, *J*=5.6 Hz)	6.11 (d, *J*=5.2 Hz)
5.34 (d, *J*=4.0 Hz)	5.34 (d, *J*=3.6 Hz)
5.14 (dd, *J*=4.0, 10.0 Hz)	5.14 (dd, *J*=3.6, 10.0 Hz)
5.11 (d, *J*=5.6 Hz)	5.11 (d, *J*=5.2 Hz)
4.79 (s)	4.79 (s)
4.71 and 4.41 (ABq, *J*=10.8 Hz)	4.71 and 4.41 (ABq, *J*=10.8 Hz)
4.49 and 4.29 (ABq, *J*=12.4 Hz)	4.49 and 4.29 (ABq, *J*=12.4 Hz)
4.11 (dd, *J*=10.0, 9.6 Hz)	4.11 (dd, *J*=10.0, 9.6 Hz)
3.70 (dd, *J*=9.6, 10.0 Hz)	3.71 (dd, *J*=9.6, 9.6 Hz)
3.45 (dd, *J*=3.2, 10.8 Hz)	3.45 (dd, *J*=4.0, 10.8 Hz)
3.38 (m)	3.38 (m)
3.13 (dd, *J*=1.2, 10.8 Hz)	3.13 (dd, *J*=1.2, 10.8 Hz)

Determination of glycosylation site of 49.

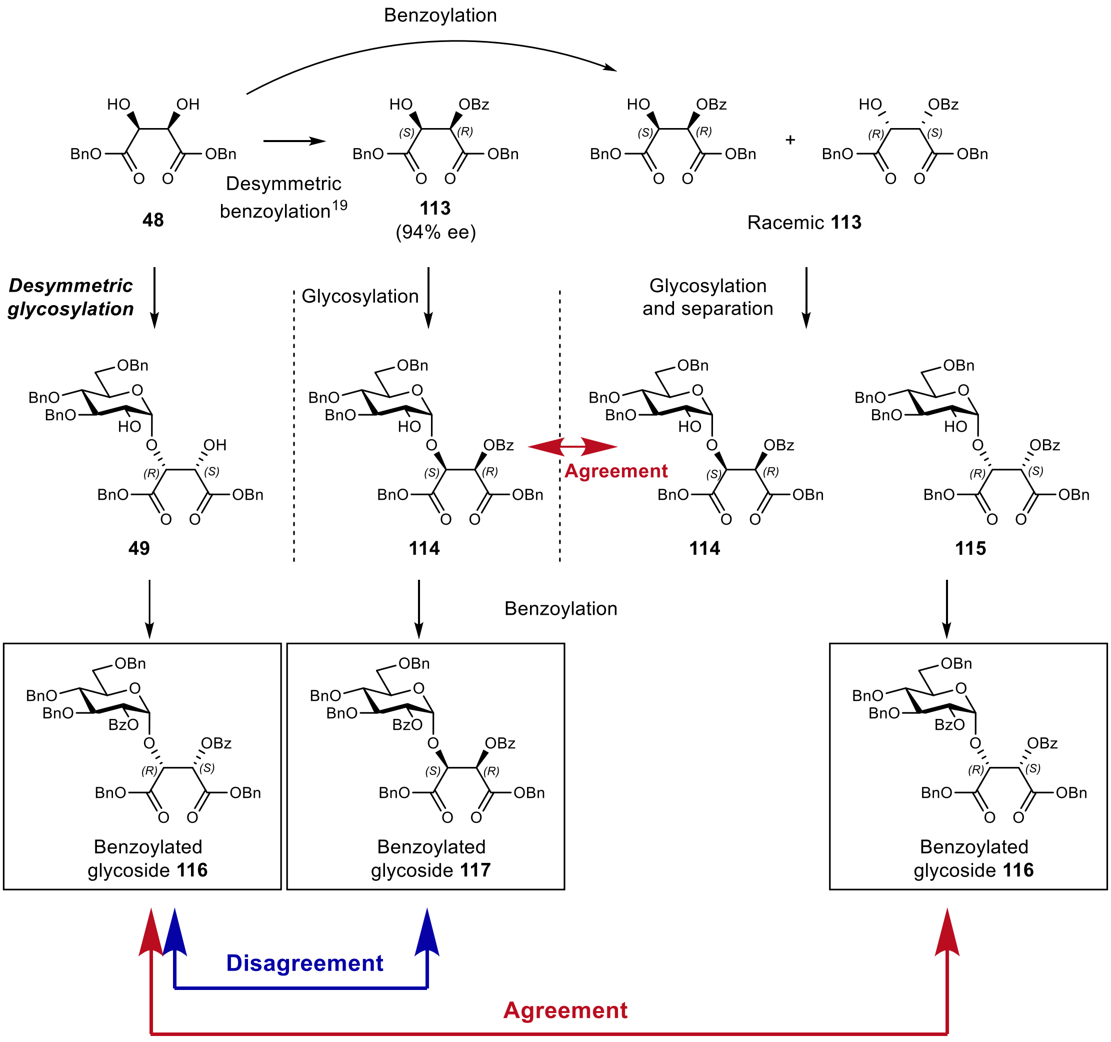

Supplementary Figure 34. Determination of glycosylation site of 49 synthesized by our desymmetric glycosylation of *meso*-diol 48.

Synthesis of benzoylated glycoside **116** from **49**.

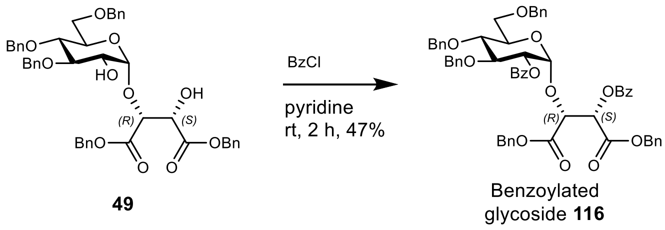

Supplementary Figure 35. Synthesis of benzoylated glycoside 116 from 49.

2-*O*-Benzoyl-3-*O*-(2-*O*-benzoyl-3,4,6-tri-*O*-benzyl-α-D-glucopyranosyl)-dibenzyl-(2*S*,3*R*)-tartrate (116)

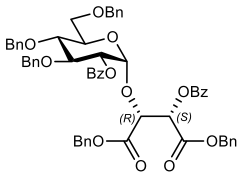

Benzoylated glycoside **116**

To a solution of **49** (9.40 mg, 12.3 μmol) in pyridine (153 μL) was added benzoyl chloride (11.5 μL, 98.6 μmol) at 0 ºC under Ar atmosphere. After the reaction mixture was stirred for 2 h at rt, the reaction was quenched by addition of H_2O (1 mL). The resultant mixture was extracted with EtOAc (1 mL×3), and then the extracts were washed with brine (1 mL), dried over anhydrous Na_2SO_4, and concentrated in *vacuo*. Purification of the residue by preparative TLC (4/1 *n*-hexane/EtOAc) gave **116** (5.60 mg, 5.77 μmol, 47% yield).

Data for **116**: Colorless syrup; R_f 0.64 (2/1 *n*-hexane/EtOAc); $[\alpha]^{25}_D$ = +115.1° (*c* 1.0, $CHCl_3$); ^{1}H-NMR (400 MHz, $CDCl_3$) δ 8.01 (2H, m), 7.97 (2H, m), 7.54-7.46 (2H, m), 7.36-7.13 (25H, m), 7.09-7.03 (4H, m), 5.91 (1H, d, *J*=2.4 Hz), 5.55 (1H, d, $J_{1,2}$=3.6 Hz, H-1), 5.16 (1H, dd, *J*=3.6 Hz, *J*=9.6 Hz), 5.13 and 4.84 (2H, ABq, *J*=12.0 Hz), 4.86 and 4.50 (2H, ABq, *J*=11.2 Hz), 4.84 (1H, d, *J*=2.4 Hz), 4.78 (2H, s), 4.77 (2H, s), 4.53 and 4.37 (2H, ABq, *J*=12.0 Hz), 4.23 (1H, dd, *J*=9.6 Hz, *J*=9.6 Hz), 4.11 (1H, m), 3.80 (1H, dd, *J*=9.6 Hz, *J*=9.6 Hz), 3.62 (1H, dd, *J*=3.6 Hz, *J*=10.4 Hz), 3.51 (1H, dd, *J*=1.6 Hz, *J*=10.4 Hz); ^{13}C-NMR (125 MHz, $CDCl_3$) δ 166.9, 165.8, 165.7, 165.4, 138.2, 138.1, 137.7, 134.6, 134.5, 133.4, 133.0, 130.0×2, 129.8, 128.8, 128.7, 128.5, 128.4×2, 128.3×3, 128.0, 127.9, 127.8, 127.7×2, 127.6, 94.9, 79.6, 77.4, 75.5, 75.1, 73.4×2, 73.1, 72.9, 71.3, 67.9, 67.7, 67.4; HRMS (ESI-TOF) *m/z* 993.3426 (993.3462 calcd for $C_{59}H_{54}O_{13}Na$ [M+Na]$^+$)

Synthesis of benzoylated glycoside **117** from **113**.

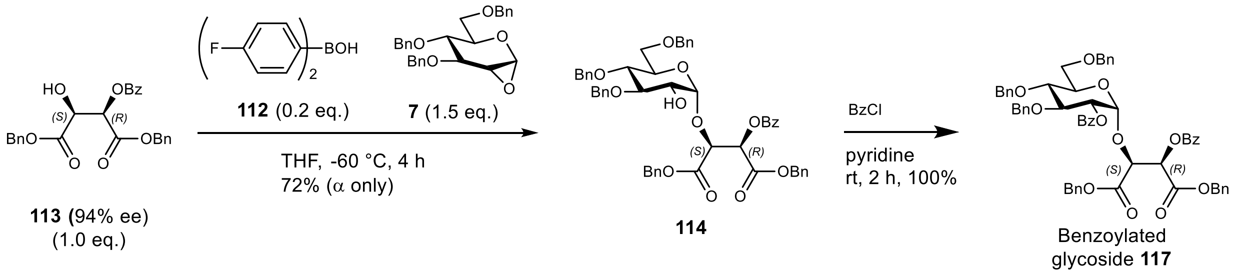

Supplementary Figure 36. Synthesis of benzoylated glycoside 117 from 113.

2-*O*-Benzoyl-3-*O*-(3,4,6-tri-*O*-benzyl-α-D-glucopyranosyl)-dibenzyl-(2*R*,3*S*)-tartrate (114)

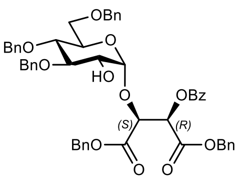

114

To a solution of **113**[19] (6.7 mg, 15.4 μmol, 94% ee) and bis(4-fluoro)phenylborinic acid (**S52**) (0.67 mg, 3.07 μmol) in dry THF (154 μL) was added a solution of **7** (13.3 mg, 30.8 μmol) in dry THF (154 μL) at -60 °C under Ar atmosphere. After the reaction mixture was stirred for 4 h at rt, the reaction was quenched by addition of 0.05 M $NaBO_3$ aq. (0.135 mL, 6.75 μmol). To the resultant mixture was added sat. NH_4Cl aq. (2 mL) and extracted with EtOAc (3 mL×3), and then the combined extracts were washed with brine (5 mL), dried over anhydrous Na_2SO_4, and concentrated in *vacuo*. Purification of the residue by preparative TLC (3/2 *n*-hexane/EtOAc and 15/1 $CHCl_3$/acetone) gave **114** (9.7 mg, 11.2 μmol, 72% yield).

Data for **114**: Colorless syrup; R_f 0.62 (3/2 *n*-hexane/EtOAc); ^{1}H-NMR (400 MHz, $CDCl_3$) δ 8.03 (2H, m), 7.60 (1H, m), 7.44 (1H, m), 7.36-7.21 (24H, m), 7.16-7.15 (2H, m), 5.87 (1H, d, *J*=2.8 Hz), 5.18 and 5.09 (2H, ABq, *J*=12.0 Hz), 5.14 and 4.96 (2H, ABq, *J*=12.0 Hz), 5.13 (1H, d, $J_{1,2}$=4.0 Hz, H-1), 4.89 and 4.70 (2H, ABq, *J*=11.2 Hz), 4.83 and 4.46 (2H, ABq, *J*=11.2 Hz), 4.76 (1H, d, *J*=2.8 Hz), 4.57 and 4.39 (2H, ABq, *J*=12.0 Hz), 4.18 (1H, br-d, *J*=9.2 Hz), 3.74 (1H, m), 3.66-3.58 (3H, m), 3.42 (1H, dd, *J*=2.0 Hz, *J*=11.2 Hz), 2.46 (1H, d, *J*=10.8 Hz); ^{13}C-NMR (100 MHz, $CDCl_3$) δ 167.1, 166.5, 165.5, 138.8, 138.5, 137.9, 134.6, 133.8, 130.1, 128.8, 128.6, 128.4, 128.3×2, 128.2, 127.9×2, 127.7×2, 127.5×2, 102.4, 83.0, 78.2, 77.2, 75.2, 74.8, 73.5, 73.4×2, 71.5, 68.0×2, 67.7; HRMS (ESI-TOF) *m/z* 905.2959 (905.2939 calcd for $C_{52}H_{50}O_{12}K$ $[M+K]^+$).

2-*O*-Benzoyl-3-*O*-(2-*O*-benzoyl-3,4,6-tri-*O*-benzyl-α-D-glucopyranosyl)-dibenzyl-(2*R*,3*S*)-tartrate (117)

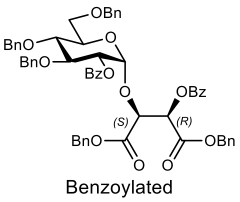

Benzoylated
glycoside **117**

To a solution of **114** (5.5 mg, 6.34 μmol) in pyridine (254 μL) was added benzoyl chloride (5.8 μL, 50.7 μmol) and at 0 °C under Ar atmosphere. After the reaction mixture was stirred for 2 h at rt,

the reaction was quenched by addition of sat. $NaHCO_3$ aq. (1 mL). The resultant mixture was extracted with EtOAc (1 mL×3), and then the extracts were washed with brine (1 mL), dried over anhydrous Na_2SO_4, and concentrated in *vacuo*. Purification of the residue by preparative TLC (2/1 *n*-hexane/EtOAc and 6/1 PhMe/ EtOAc) to give **117** (6.2 mg, 6.34 μmol, quantitative yield).

Data for **117**: Colorless syrup; R_f 0.53 (2/1 *n*-hexane/EtOAc); $[\alpha]^{23}_{D}$ = +49.7° (*c* 0.6, $CHCl_3$); ^1H-NMR (400 MHz, $CDCl_3$) δ 7.88 (2H, m), 7.73 (1H, m), 7.49 (1H, m), 7.40 (1H, m), 7.32-7.09 (29H, m), 5.84 (1H, d, *J*=2.8 Hz), 5.45 (1H, d, $J_{1,2}$=4.0 Hz, H-1), 5.19 (1H, dd, *J*=4.0 Hz, *J*=9.6 Hz), 5.15 and 4.97 (2H, ABq, *J*=12.0 Hz), 5.09 and 4.86 (2H, ABq, *J*=12.4 Hz), 4.83 and 4.52 (2H, ABq, *J*=10.8 Hz), 4.81 (1H, d, *J*=2.8 Hz), 4.73 and 4.67 (2H, ABq, *J*=11.2 Hz), 4.60 and 4.42 (2H, ABq, *J*=12.4 Hz), 4.34 (1H, m), 4.17 (1H, dd, *J*=9.6 Hz, *J*=9.6 Hz), 3.82 (1H, dd, *J*=9.6 Hz, *J*=9.6 Hz), 3.69 (1H, dd, *J*=3.2 Hz, *J*=11.2 Hz), 3.49 (1H, dd, *J*=1.6 Hz, *J*=11.2 Hz); ^{13}C-NMR (100 MHz, $CDCl_3$) δ 167.4, 165.9, 165.6, 165.1, 138.3, 138.1, 137.9, 135.0, 134.8, 133.3, 132.9, 129.9, 129.3, 128.7, 128.5×3, 128.4, 128.3, 128.2, 128.1, 127.8×2, 127.7, 127.6, 127.5, 98.2, 79.8, 77.4, 76.6, 75.5, 74.9, 73.4, 73.3, 73.1, 71.5, 67.9, 67.6, 67.3; HRMS (ESI-TOF) *m/z* 993.3421 (993.3462 calcd for $C_{59}H_{54}O_{13}Na$ $[M+Na]^+$).

Synthesis of benzoylated glycoside **116** from racemic **113**.

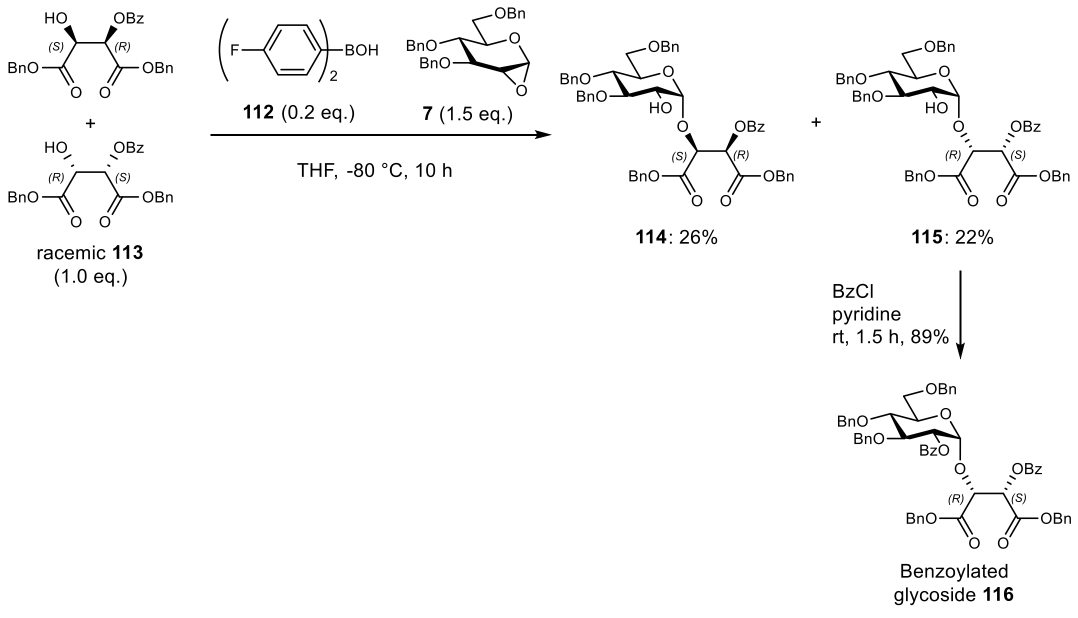

Supplementary Figure 37. Synthesis of benzoylated glycoside 116 from racemic 113.

2-*O*-Benzoyl-3-*O*-(3,4,6-tri-*O*-benzyl-α-D-glucopyranosyl)-dibenzyl-(2*R*,3*S*)-tartrate (114) and 2-*O*-Benzoyl-3-*O*-(3,4,6-tri-*O*-benzyl-α-D-glucopyranosyl)-dibenzyl-(2*S*,3*R*)-tartrate (115)

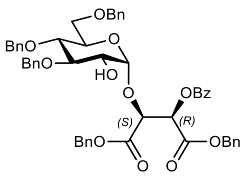

114

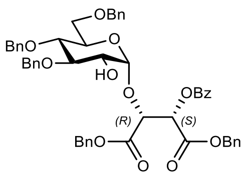

115

To a solution of racemic **113** (22.4 mg, 51.6 μmol) and bis(4-fluoro)phenylborinic acid (**112**) (2.2 mg, 10.1 μmol) in dry THF (501 μL) was added a solution of **7** (44.6 mg, 103 μmol) in dry THF (501 μL) at -80 °C under Ar atmosphere. After the reaction mixture was stirred for 10 h at rt, the reaction was quenched by addition of 0.05 M $NaBO_3$ aq. (0.444 mL, 22.2 μmol). To the resultant mixture was added sat. NH_4Cl aq. (2 mL) and extracted with EtOAc (3 mL×3), and then the combined extracts were washed with brine (5 mL), dried over anhydrous Na_2SO_4, and concentrated in *vacuo*. Purification of the residue by preparative TLC (8/1 PhMe/acetone) gave **114** (11.6 mg, 13.4 μmol, 26% yield) and crude **115**. Purification of crude **115** by preparative TLC (8/1 PhMe/acetone) gave **115** (11.6 mg, 13.4 μmol, 22% yield).

Data for **114**: Colorless syrup; R_f 0.62 (3/2 *n*-hexane/EtOAc); $[\alpha]^{23}_D$ = +34.5° (*c* 0.6, $CHCl_3$); ^1H-NMR (400 MHz, $CDCl_3$) δ 8.03 (2H, m), 7.59 (1H, m), 7.44 (1H, m), 7.36-7.22 (24H, m), 7.17-7.14 (2H, m), 5.88 (1H, d, *J*=2.8 Hz), 5.18 and 5.09 (2H, ABq, *J*=12.0 Hz), 5.13 and 4.96 (2H, ABq, *J*=12.0 Hz), 5.13 (1H, d, $J_{1,2}$=4.0 Hz, H-1), 4.89 and 4.70 (2H, ABq, *J*=11.6 Hz), 4.83 and 4.46 (2H, ABq, *J*=10.8 Hz), 4.76 (1H, d, *J*=2.8 Hz), 4.57 and 4.39 (2H, ABq, *J*=12.0 Hz), 4.18 (1H, br-d, *J*=8.8 Hz), 3.74 (1H, m), 3.66-3.58 (3H, m), 3.42 (1H, dd, *J*=2.0 Hz, *J*=10.8 Hz), 2.46 (1H, d, *J*=10.8 Hz); HRMS (ESI-TOF) *m/z* 889.3207 (889.3200 calcd for $C_{52}H_{50}O_{12}Na$ $[M+Na]^+$).

Data for **115**: Colorless syrup; R_f 0.48 (3/2 *n*-hexane/EtOAc); $[\alpha]^{23}_D$ = +69.1° (*c* 0.7, $CHCl_3$); ^1H-NMR (400 MHz, $CDCl_3$) δ 7.99 (2H, m), 7.54 (1H, m), 7.39-7.20 (25H, m), 7.11-7.09 (2H, m), 5.94 (1H, d, *J*=2.0 Hz), 5.18 and 5.10 (2H, ABq, *J*=12.0 Hz), 5.17 and 4.96 (2H, ABq, *J*=12.0 Hz), 5.04 (1H, d, $J_{1,2}$=3.6 Hz, H-1), 4.93 and 4.69 (2H, ABq, *J*=11.6 Hz), 4.81 (1H, d, *J*=2.0 Hz), 4.79 and 4.69 (2H, ABq, *J*=11.6 Hz), 4.50 and 4.21 (2H, ABq, *J*=12.0 Hz), 3.92 (1H, m), 3.74 (1H, m), 3.66-3.58 (3H, m), 3.38 (1H, dd, *J*=2.0 Hz, *J*=10.4 Hz), 2.88 (1H, d, *J*=10.4 Hz); ^{13}C-NMR (100 MHz, $CDCl_3$) δ 168.2, 165.5, 165.3, 138.8, 138.3, 137.7, 134.6, 134.4, 133.6, 130.0, 128.9, 128.8, 128.6×2, 128.5,

128.3×2, 128.2, 127.9×3, 127.7, 127.6, 127.5, 100.5, 83.1, 76.5, 76.0, 75.3, 75.0, 73.4, 73.1, 72.8, 71.5, 68.1, 67.8×2; HRMS (ESI-TOF) *m/z* 889.3189 (889.3200 calcd for $C_{52}H_{50}O_{12}Na$ $[M+Na]^+$).

2-*O*-Benzoyl-3-*O*-(2-*O*-benzoyl-3,4,6-tri-*O*-benzyl-α-D-glucopyranosyl)-dibenzyl-(2*S*,3*R*)-tartrate (116)

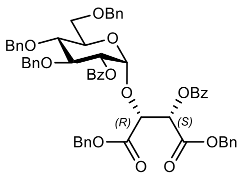

Benzoylated glycoside **116**

To a solution of **115** (6.60 mg, 7.61 μmol) in pyridine (76.1 μL) was added benzoyl chloride (17.7 μL, 0.152 mmol) and at 0 ºC under Ar atmosphere. After the reaction mixture was stirred for 1.5 h at rt, the reaction was quenched by addition of sat. $NaHCO_3$ aq. (1 mL). The resultant mixture was extracted with EtOAc (1 mL×3), and then the extracts were washed with brine (1 mL), dried over anhydrous Na_2SO_4, and concentrated in *vacuo*. Purification of the residue by preparative TLC (3/1 *n*-hexane/EtOAc and 40/1 $CHCl_3$/acetone) gave **116** (6.60 mg, 6.80 μmol, 89% yield).

Data for **116**: Colorless syrup; R_f 0.33 (4/1 *n*-hexane/EtOAc); $[\alpha]^{24}_D$ = +107.4° (*c* 0.4, $CHCl_3$); ^1H-NMR (400 MHz, $CDCl_3$) δ 8.01 (2H, m), 7.96 (2H, m), 7.54-7.46 (2H, m), 7.36-7.14 (25H, m), 7.09-7.04 (4H, m), 5.91 (1H, d, *J*=2.4 Hz), 5.55 (1H, d, $J_{1,2}$=3.6 Hz, H-1), 5.16 (1H, dd, *J*=3.6 Hz, *J*=10.0 Hz), 5.13 and 4.84 (2H, ABq, *J*=12.0 Hz), 4.86 and 4.50 (2H, ABq, *J*=11.2 Hz), 4.84 (1H, d, *J*=2.4 Hz), 4.78 (2H, s), 4.77 (2H, s), 4.53 and 4.37 (2H, ABq, *J*=12.0 Hz), 4.23 (1H, dd, *J*=10.0 Hz, *J*=9.6 Hz), 4.11 (1H, m), 3.80 (1H, dd, *J*=9.6 Hz, *J*=9.6 Hz), 3.62 (1H, dd, *J*=3.6 Hz, *J*=10.4 Hz), 3.51 (1H, dd, *J*=2.0 Hz, *J*=10.4 Hz); HRMS (ESI-TOF) *m/z* 1009.3234 (1009.3202 calcd for $C_{59}H_{54}O_{13}K$ $[M+K]^+$).

Determination of glycosylation site of 49

Comparison of NMR data of glycoside **114**

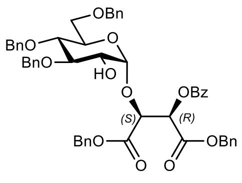

114

Supplementary Table 30. Comparison of ^{1}H-NMR data of 114.

δ(Structurally defined glycoside **114** from racemic **113**) (400 MHz, $CDCl_3$)	δ(glycoside **114** derived from **113** 96%ee) (400 MHz, $CDCl_3$)
8.03 (m)	8.03 (m)
7.59 (m)	7.60 (m)
7.44 (m)	7.44 (m)
7.36-7.22 (m)	7.36-7.21 (m)
7.17-7.14 (m)	7.16-7.15 (m)
5.88 (d, *J*=2.8 Hz)	5.87 (d, *J*=2.8 Hz)
5.18 and 5.09 (ABq, *J*=12.0 Hz)	5.18 and 5.09 (ABq, *J*=12.0 Hz)
5.13 and 4.96 (ABq, *J*=12.0 Hz)	5.14 and 4.96 (ABq, *J*=12.0 Hz)
5.13 (d, *J*=4.0 Hz)	5.13 (d, *J*=4.0 Hz)
4.89 and 4.70 (ABq, *J*=11.6 Hz)	4.89 and 4.70 (ABq, *J*=11.2 Hz)
4.83 and 4.46 (ABq, *J*=10.8 Hz)	4.83 and 4.46 (ABq, *J*=11.2 Hz)
4.76 (d, *J*=2.8 Hz)	4.76 (d, *J*=2.8 Hz)
4.57 and 4.39 (ABq, *J*=12.0 Hz)	4.57 and 4.39 (ABq, *J*=12.0 Hz)
4.18 (br-d, *J*=8.8 Hz)	4.18 (br-d, *J*=9.2 Hz)
3.74 (m)	3.74 (m)
3.66-3.58 (m)	3.66-3.58 (m)
3.42 (dd, *J*=2.0, 10.8 Hz)	3.42 (dd, *J*=2.0, 11.2 Hz)
2.46 (d, *J*=10.8 Hz)	2.46 (d, *J*=10.8 Hz)

Comparison of NMR data of benzoylated glycoside **116** and **117**

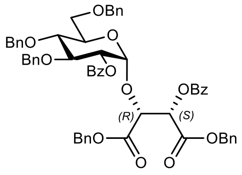

Benzoylated glycoside **116**

Supplementary Table 31. Comparison of ^{1}H-NMR data of benzoylated glycoside 116 and 117.

δ(Structurally defined benzoylated glycoside **117**) (400 MHz, $CDCl_3$)	δ(Structurally defined benzoylated glycoside **116**) (400 MHz, $CDCl_3$)	δ(Benzoylated glycoside **116** derived from **49**) (400 MHz, $CDCl_3$)
7.88 (m)	8.01 (m)	8.01 (m)
7.73 (m)	7.96 (m)	7.97 (m)
7.49 (m)	7.54-7.46 (m)	7.54-7.46 (m)
7.40 (m)		
7.32-7.09 (m)	7.36-7.14 (m)	7.36-7.13 (m)
	7.09-7.04 (m)	7.09-7.03 (m)
5.84 (d, *J*=2.8 Hz)	5.91 (d, *J*=2.4 Hz)	5.91 (d, *J*=2.4 Hz)
5.45 (d, *J*=4.0 Hz)	5.55 (d, *J*=3.6 Hz)	5.55 (d, *J*=3.6 Hz)
5.19 (dd, *J*=4.0, 9.6 Hz)	5.16 (dd, *J*=3.6, 10.0 Hz)	5.16 (dd, *J*=3.6, 9.6 Hz)
5.15 and 4.97 (ABq, *J*=12.0 Hz)	5.13 and 4.84 (ABq, *J*=12.0 Hz)	5.13 and 4.84 (ABq, *J*=12.0 Hz)
5.09 and 4.86 (ABq, *J*=12.4 Hz)	4.86 and 4.50 (ABq, *J*=11.2 Hz)	4.86 and 4.50 (ABq, *J*=11.2 Hz)

4.81 (d, *J*=2.8 Hz)	4.84 (d, *J*=2.4 Hz)	4.84 (d, *J*=2.4 Hz)
4.83 and 4.52 (ABq, *J*=10.8 Hz)	4.78 (s)	4.78 (s)
4.73 and 4.67 (ABq, *J*=10.8 Hz)	4.77 (s)	4.77 (s)
4.60 and 4.42 (ABq, *J*=12.4 Hz)	4.53 and 4.37 (ABq, *J*=12.0 Hz)	4.53 and 4.37 (ABq, *J*=12.0 Hz)
4.34 (m)	4.23 (dd, *J*=10.0, 9.6 Hz)	4.23 (dd, *J*=9.6, 9.6 Hz)
4.17 (dd, *J*=9.6, 9.6 Hz)	4.11 (m)	4.11 (m)
3.82 (dd, *J*=9.6, 9.6 Hz)	3.80 (dd, *J*=9.6, 9.6 Hz)	3.80 (dd, *J*=9.6, 9.6 Hz)
3.69 (dd, *J*=3.2, 11.2 Hz)	3.62 (dd, *J*=3.6, 10.4 Hz)	3.62 (dd, *J*=3.6, 10.4 Hz)
3.49 (dd, *J*=1.6, 11.2 Hz)	3.51 (dd, *J*=2.0, 10.4 Hz)	3.51 (dd, *J*=1.6, 10.4 Hz)

Synthesis of Core Structures of PIMs and GPI Anchors

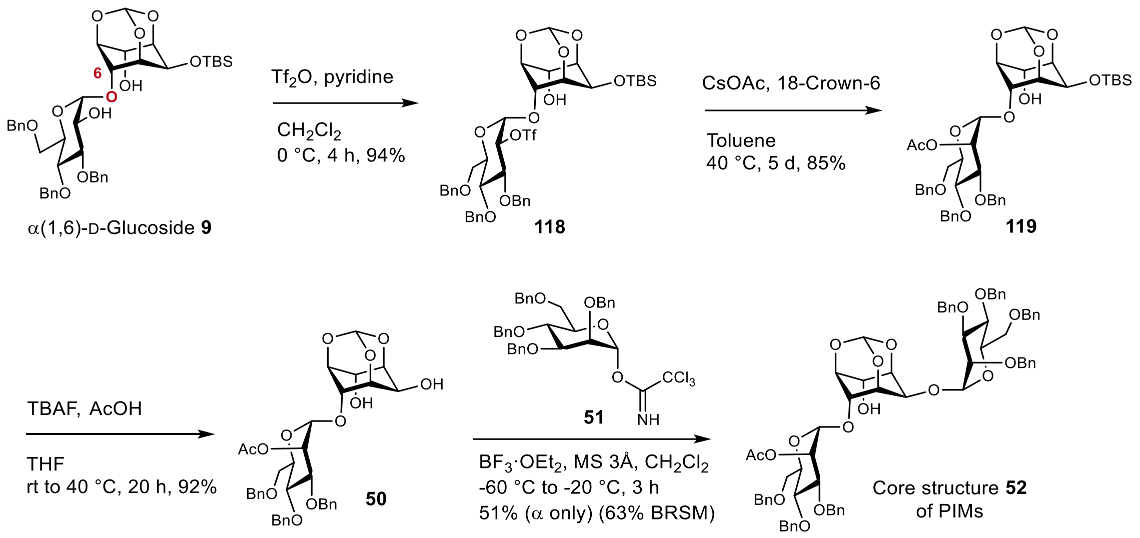

Supplementary Figure 38. Synthetic scheme of core structure 52 of PIMs.

6-*O*-(3,4,6-Tri-*O*-benzyl-2-*O*-trifluoromethanesulfonyl-α-D-glucopyranosyl)-2-*O*-*tert*-butyldimethylsilyl-D-*myo*-inositol-1,3,5-orthoformate (118)

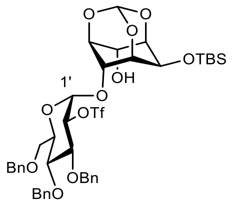

118

To a solution of **9** (52.2 mg, 70.8 μmol) and pyridine (28.5 μL, 0.106 mmol) in CH_2Cl_2 (496 μL) was added Tf_2O (106 μL, 106 μmol, 1.0 M in CH_2Cl_2) at 0 ºC under Ar atmosphere. After the reaction mixture was stirred for 4 h at 0 ºC, the reaction mixture was poured into sat. $NaHCO_3$ aq. (1 mL). The aqueous layer was extracted with $CHCl_3$ (1 mL×3), and then the combined extracts were washed with brine (1 mL), dried over anhydrous Na_2SO_4, and concentrated in *vacuo*. Purification of the residue by silica gel column chromatography (3/1 *n*-hexane/EtOAc) gave **118** (58.0 mg, 66.7 μmol, 94% yield).

Data for **118**: Colorless foam; R_f 0.46 (2/1 *n*-hexane/EtOAc); $[\alpha]^{24}_D$ +53.6° (c 1.0, $CHCl_3$); ^{1}H-NMR (500 MHz, $CDCl_3$) δ 7.38-7.27 (13H, m), 7.16-7.13 (2H, m), 5.50 (1H, d, *J*=1.0 Hz), 5.23 (1H, d, *J*=4.0 Hz, H-1'), 4.87 and 4.75 (2H, ABq, *J*=10.0 Hz), 4.77 and 4.48 (2H, ABq, *J*=10.5 Hz), 4.74 (1H, dd, *J*=4.0 Hz, *J*=10.0 Hz), 4.54 and 4.48 (2H, ABq, *J*=11.5 Hz), 4.32 (1H, m), 4.25 (1H, m), 4.22 (1H, m), 4.17 (1H, m), 4.13 (1H, m), 4.07 (1H, m), 3.86 (1H, t, *J*=8.5 Hz), 3.70 (1H, dd, *J*=2.0 Hz, *J*=10.0 Hz), 3.54 (1H, t, *J*=10.0 Hz), 3.70 (1H, dd, *J*=3.0 Hz, *J*=10.0 Hz), 3.16 (1H, d, *J*=6.5 Hz),

0.95 (9H, s), 0.16 (6H, s); ^{13}C-NMR (125 MHz, $CDCl_3$) δ 137.1, 136.9×2, 128.6, 128.5×2, 128.2×2, 128.1, 128.0×2, 118.4 (q, $J_{C\text{-}F}$ = 317.8 Hz, CF_3), 102.7, 97.7, 83.4, 78.8, 77.8, 76.7, 76.0, 75.3, 74.3, 73.8, 72.6, 71.3, 70.0, 68.4, 67.6, 60.8, 25.9, 18.4, -4.6, -4.7; HRMS (ESI-TOF) *m/z* 869.2846 (869.2850 calcd for $C_{41}H_{52}O_{13}SiSF_3$ $[M+H]^+$).

6-*O*-(2-*O*-Acetyl-3,4,6-tri-*O*-benzyl-α-D-mannopyranosyl)-2-*O*-*tert*-butyldimethylsilyl-D-*myo*-inositol-1,3,5-orthoformate (119)

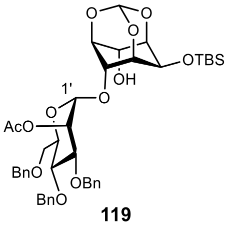

119

To a solution of **118** (12.9 mg, 14.8 μmol) and 18-Crown-6 (5.7 mg, 29.6 μmol) in PhMe (742 μL) was added CsOAc (7.8 mg, 29.6 μmol) at room temperature under Ar atmosphere. After the reaction mixture was stirred for 5 days at 40 °C, the reaction mixture was concentrated in *vacuo*. Purification of the residue by silica gel column chromatography (20/1 $CHCl_3$/EtOAc) gave **119** (9.8 mg, 12.6 μmol, 85% yield).

Data for **119**: Colorless foam; R_f 0.49 (2/1 *n*-hexane/EtOAc); $[\alpha]^{25}_D$ +32.4° (c 1.0, $CHCl_3$); ^{1}H-NMR (400 MHz, $CDCl_3$) δ 7.38-7.29 (13H, m), 7.19-7.17 (2H, m), 5.49 (1H, d, *J*=0.8 Hz), 5.17 (1H, t, *J*=2.4 Hz), 4.95 (1H, d, *J*=2.4 Hz, H-1'), 4.81 and 4.45 (2H, ABq, *J*=10.8 Hz), 4.63 and 4.50 (2H, ABq, *J*=11.6 Hz), 4.61 and 4.53 (2H, ABq, *J*=12.0 Hz), 4.54 (1H, m), 4.30-4.24 (2H, m), 4.14-4.11 (2H, m), 4.05 (1H, m), 3.98 (1H, m), 3.77-3.71 (3H, m), 3.65 (1H, dd, *J*=6.0 Hz, *J*=10.4 Hz), 3.16 (1H, d, *J*=6.4 Hz), 2.15 (3H, s), 0.94 (9H, s), 0.14 (6H, s); ^{13}C-NMR (125 MHz, $CDCl_3$) δ 170.1, 137.8, 137.4, 137.3, 128.5, 128.4, 128.1, 128.0, 127.9, 102.7, 98.5, 74.9, 74.8, 74.5, 74.2, 73.7, 72.5, 72.3, 72.2, 69.6, 69.2, 69.1, 67.7, 60.8, 25,9, 21.0, 18.5, -4.7, -4.8; HRMS (ESI-TOF) *m/z* 779.3457 (779.3463 calcd for $C_{42}H_{55}O_{12}Si$ $[M+H]^+$).

6-*O*-(2-*O*-Acetyl-3,4,6-tri-*O*-benzyl-α-D-mannopyranosyl)-D-*myo*-inositol-1,3,5-orthoformate (50)

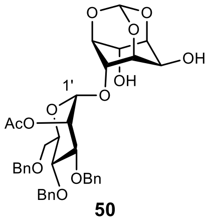

50

To a solution of **119** (57.8 mg, 74.2 μmol) and AcOH (8.5 μL, 14.8 μmol) in THF (297 μL) was added TBAF (445 μL, 445 μmol, 1.0 M in THF) at room temperature under Ar atmosphere. After the reaction mixture was stirred for 20 h at 40 ºC, the reaction mixture was concentrated in *vacuo*. Purification of the residue by silica gel column chromatography (1/1 *n*-hexane /EtOAc) gave **50** (45.4 mg, 68.3 μmol, 92% yield).

Data for **50**: Colorless foam; R_f 0.30 (1/1 *n*-hexane/EtOAc); $[\alpha]^{24}_{D}$ +42.0° (c 1.0, $CHCl_3$); ^{1}H-NMR (400 MHz, $CDCl_3$) δ 7.38-7.28 (13H, m), 7.19-7.15 (2H, m), 5.44 (1H, d, *J*=0.8 Hz), 5.19 (1H, t, *J*=2.4 Hz), 4.97 (1H, d, *J*=2.4 Hz, H-1'), 4.81 and 4.44 (2H, ABq, *J*=10.8 Hz), 4.65 and 4.54 (2H, ABq, *J*=11.2 Hz), 4.61 and 4.51 (2H, ABq, *J*=12.0 Hz), 4.59 (1H, m), 4.35-4.27 (2H, m), 4.21 (1H, m), 4.14 (1H, m), 4.00-3.93 (2H, m), 3.80-3.70 (3H, m), 3.66 (1H, dd, *J*=5.6 Hz, *J*=10.4 Hz), 3.22 (1H, d, *J*=6.8 Hz), 3.06 (1H, d, *J*=12.0 Hz), 2.15 (3H, s); ^{13}C-NMR (125 MHz, $CDCl_3$) δ 170.2, 137.8, 137.4, 137.3, 128.5×2, 128.4, 128.2, 128.0, 127.9×3, 127.3, 102.8, 98.5, 77.2, 74.9, 74.1×2, 73.9, 73.7, 72.4, 72.2, 72.0, 69.2, 69.1, 69.0, 67.3, 60.5, 21.0; HRMS (ESI-TOF) *m/z* 703.2173 (703.2157 calcd for $C_{36}H_{40}O_{12}K$ $[M+K]^+$).

6-*O*-(2-*O*-Acetyl-3,4,6-tri-*O*-benzyl-α-D-mannopyranosyl)-2-*O*-(2,3,4,6-tetra-*O*-benzyl-α-D-mannopyranosyl)-D-*myo*-inositol-1,3,5-orthoformate (52)

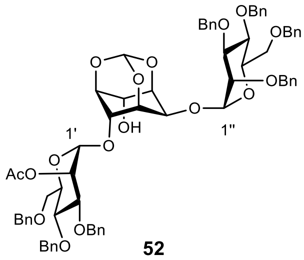

52

A solution of **50** (21.8 mg, 32.8 μmol), **51**[21] (22.5 mg, 32.8 μmol) and 3 Å molecular sieves (65 mg) in dry CH_2Cl_2 (865 μL) was stirred for 1 h at room temperature under Ar atmosphere. The reaction mixture was cooled to –60 °C, and then $BF_3{\cdot}OEt_2$ (1.30 μL, 9.80 μmol) was added to the reaction mixture. After stirring for 1 h at –60 °C, an additional solution of **51** (22.5 mg, 32.8 μmol) in dry CH_2Cl_2 (865 μL) and $BF_3{\cdot}OEt_2$ (1.30 μL, 9.80 μmol) were consecutively added, then the reaction mixture was warmed to −20 °C. After being stirred for 2 h at −20 °C, the reaction was quenched by addition of NEt_3 (5 μL). The reaction mixture was filtered through celite pad, and the filtrate was concentrated in *vacuo*. To the residue was added sat. $NaHCO_3$ aq. (1 mL). The aqueous layer was extracted with $CHCl_3$ (1 mL×5), and then the extracts were washed with brine (1 mL), dried over anhydrous Na_2SO_4, and concentrated in *vacuo*. Purification of the residue by silica gel column

chromatography (2/1 *n*-hexane/EtOAc) to give **52** (20.0 mg, 16.8 μmol, 51% yield) and unreacted substrate **50** (4.1 mg, 6.17 μmol, 19% yield).

Data for **52**: Colorless foam; R_f 0.33 (2/1 *n*-hexane/EtOAc); $[\alpha]^{25}_{D}$ +37.2° (c 1.0, $CHCl_3$); ^{1}H-NMR (400 MHz, $CDCl_3$) δ 7.42-7.14 (35H, m), 5.43 (1H, d, *J*=1.2 Hz), 5.22 (1H, t, *J*=1.6 Hz), 5.15 (1H, d, *J*=1.6 Hz, H-1'), 4.95 (1H, d, *J*=2.0 Hz, H-1''), 4.88-4.75 (4H, m), 4.71-4.61 (5H, m), 4.58-4.41 (6H, m), 4.40 (1H, m), 4.27 (1H, m), 4.23 (1H, m), 4.16 (1H, m), 4.09-3.96 (6H, m), 3.85-3.79 (2H, m), 3.87-3.64 (4H, m), 2.98 (1H, d, *J*=6.8 Hz), 2.14 (3H, s); ^{13}C-NMR (125 MHz, $CDCl_3$) δ 170.3, 138.6, 138.4×2, 137.9, 137.5, 128.5, 128.4, 128.3, 128.2, 128.1, 128.0, 127.9×2, 127.8×2, 127.7, 127.5, 127.4×2, 102.7, 98.9 ($^1J_{CH}$=170 Hz), 98.3 ($^1J_{CH}$=168 Hz), 80.2, 77.5, 75.4, 75.0×2, 74.7, 74.1, 73.2, 72.8, 72.5, 72.4, 72.3, 72.2, 69.9, 69.7, 69.3, 69.1, 67.4, 66.6, 21.0; HRMS (ESI-TOF) *m/z* 1225.4561 (1225.4563 calcd for $C_{70}H_{74}O_{17}K$ $[M+K]^+$).

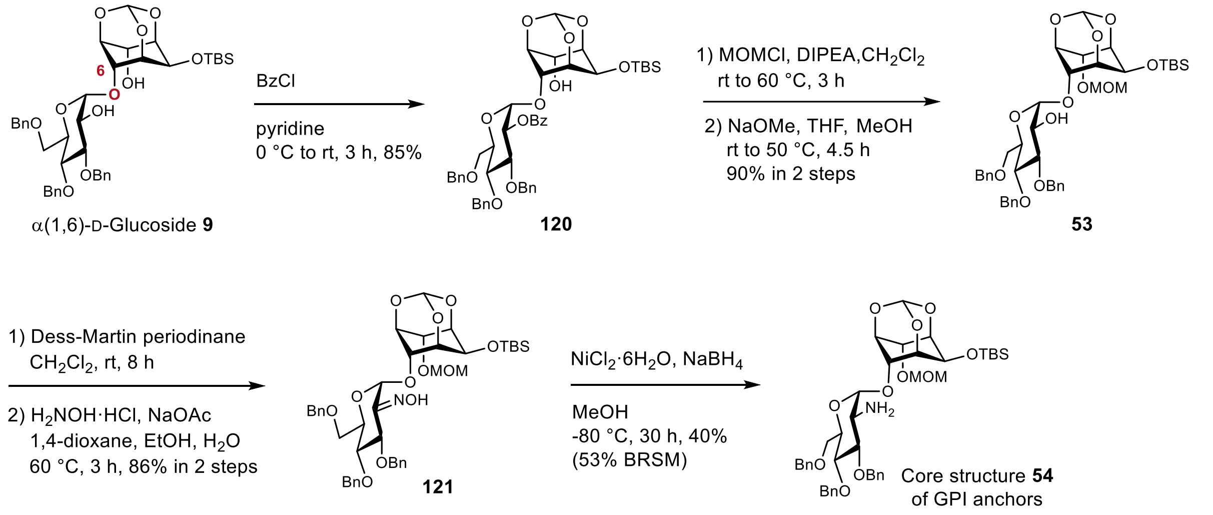

Supplementary Figure 39. Synthetic scheme of core structure 54 of GPI anchors.

6-*O*-(2-*O*-Benzoyl-3,4,6-tri-*O*-benzyl-α-D-glucopyranosyl)-2-*O*-*tert*-butyldimethylsilyl-D-*myo*-inositol-1,3,5-orthoformate (120)

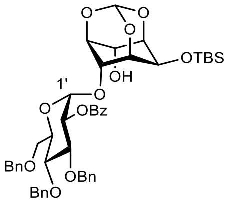

120

To a solution of **9** (74.4 mg, 101 μmol) in pyridine (2.02 mL) was added BzCl (23.5 μL, 202 μmol) at 0 °C under Ar atmosphere. The reaction mixture was stirred for 1 h, then the reaction mixture

was warmed to rt. After stirring for 2 h at room temperature, the reaction was quenched by addition of sat. $NaHCO_3$ aq. (2 mL). The aqueous layer was extracted with EtOAc (3 mL×3), and then the combined extracts were washed with brine (2 mL), dried over anhydrous Na_2SO_4, and concentrated in *vacuo*. Purification of the residue by silica gel column chromatography (12/1 PhMe/acetone) gave **120** (71.9 mg, 85.5 μmol, 85% yield).

Data for **120**: White solid; R_f 0.50 (2/1 *n*-hexane/EtOAc); $[\alpha]^{25}_D$ +95.5 (c 1.0, $CHCl_3$); mp 162-163 °C; ^{1}H-NMR (400 MHz, $CDCl_3$) δ 7.98-7.61 (2H, m), 7.59 (1H, t, *J*=7.2 Hz), 7.44 (2H, t, *J*=7.2 Hz), 7.38-7.28 (8H, m), 7.19-7.12 (7H, m), 5.50 (1H, d, *J*=0.8 Hz), 5.29 (1H, d, *J*=4.0 Hz, H-1'), 5.24 (1H, dd, *J*=4.0 Hz, *J*=9.6 Hz), 4.82 and 4.53 (2H, ABq, *J*=10.4 Hz), 4.78 and 4.72 (2H, ABq, *J*=11.2 Hz), 4.62 and 4.53 (2H, ABq, *J*=12.0 Hz), 4.60 (1H, m), 4.35 (1H, m), 4.28 (1H, m), 4.14 (1H, m), 4.04-3.94 (4H, m), 3.78-3.66 (4H, m), 0.79 (9H, s), -0.12 (6H, s); ^{13}C-NMR (100 MHz, $CDCl_3$) δ 165.2, 137.6, 137.5, 137.3, 133.7, 129.7, 128.8, 128.8, 128.7, 128.5×2, 128.3, 128.0, 127.9, 127.8×2, 102.7, 94.6, 79.8, 77.4, 75.6, 75.2, 74.6, 73.7, 72.8, 72.0, 71.6, 71.5, 68.9, 68.3, 68.0, 60.5, 25.7, 18.1, -5.1×2; HRMS (ESI-TOF) *m/z* 879.3177 (879.3178 calcd for $C_{47}H_{56}O_{12}SiK$ $[M+K]^+$).

6-*O*-(3,4,6-Tri-*O*-benzyl-α-D-glucopyranosyl)-2-*O*-*tert*-butyldimethylsilyl-4-*O*-methoxymethyl-D-*myo*-inositol-1,3,5-orthoformate (53)

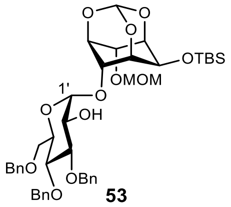

53

To a solution of **120** (48.2 mg, 57.3 μmol) and DIPEA (600 μL, 3.44 mmol) in 1,2-dichloroethane (573 μL) was added MOMCl (131 μL, 1.72 mmol) at room temperature under Ar atmosphere. After the reaction mixture was stirred for 3 h at 60 ºC, the reaction was quenched by addition of sat. $NaHCO_3$ aq. (2 mL). The aqueous layer was extracted with $CHCl_3$ (2 mL×5), and then the combined extracts were washed with brine (2 mL), dried over anhydrous Na_2SO_4, and concentrated in *vacuo*.

To a solution of the residue in THF-MeOH (1/1, v/v, 1.15 mL) was added NaOMe (69.0 μL, 344 μmol) at room temperature under Ar atmosphere. After the reaction mixture was stirred for 4.5 h at 50 ºC, the reaction was quenched by addition of sat. NH_4Cl aq. (2 mL). The aqueous layer was extracted with EtOAc (2 mL×5), and then the combined extracts were washed with brine (2 mL), dried over anhydrous Na_2SO_4, and concentrated in *vacuo*. Purification of the residue by silica gel column chromatography (3/1 *n*-hexane/EtOAc) gave **53** (40.4 mg, 51.7 μmol, 90% yield in 2 steps).

Data for **53**: Colorless syrup; R_f 0.30 (3/1 *n*-hexane/EtOAc); $[\alpha]^{25}_D$ +73.9° (c 1.0, $CHCl_3$); ^{1}H-NMR (400 MHz, $CDCl_3$) δ 7.37-7.26 (13H, m), 7.16-7.12 (2H, m), 5.50 (1H, d, *J*=1.6 Hz), 5.00 (1H, d, *J*=4.0 Hz, H-1'), 4.89 and 4.81 (2H, ABq, *J*=11.6 Hz), 4.82 and 4.49 (2H, ABq, *J*=11.2 Hz), 4.63 and 4.51 (2H, ABq, *J*=12.0 Hz), 4.62 and 4.56 (2H, ABq, *J*=6.4 Hz) 4.58 (1H, m), 4.44 (1H, m), 4.25 (2H, br-s), 4.19 (1H, m), 4.13 (1H, m), 3.86 (1H, m), 3.75-3.69 (2H, m), 3.67-3.58 (3H, m), 3.31 (3H, s), 2.25 (1H, d, *J*=9.6 Hz), 0.94 (9H, s), 0.14 (6H, s); ^{13}C-NMR (125 MHz, $CDCl_3$) δ 138.4, 138.0, 137.6, 128.3×2, 128.2, 127.9, 127.8, 127.7, 127.6×2, 102.9, 96.3, 96.2, 82.7, 76.9, 74.9, 74.8, 73.5×2, 72.8, 72.5, 71.8, 70.9, 68.8, 68.1, 60.8, 55.8, 25.8, 18.2, -4.7, -4.8; HRMS (ESI-TOF) *m/z* 803.3427 (803.3439 calcd for $C_{42}H_{56}O_{12}SiNa$ $[M+Na]^+$).

6-*O*-(3,4,6-Tri-*O*-benzyl-2-oximino-α-D-glucopyranosyl)-2-*O*-*tert*-butyldimethylsilyl-4-*O*-methoxymethyl-D-*myo*-inositol-1,3,5-orthoformate (121)

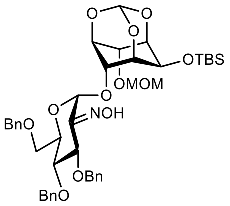

121

To a solution of **53** (9.8 mg, 12.5 μmol) in CH_2Cl_2 (313 μL) was added Dess-Martin periodinane (8.0 mg, 18.9 μmol) at 0 ºC under Ar atmosphere. The reaction mixture was warmed to room temperature, and then Dess-Martin periodinane (8.0 mg, 18.9 μmol) was added to the reaction mixture. After the reaction mixture was stirred for 8 h at room temperature, the reaction was filtered through celite pad, and the filtrate was concentrated in *vacuo*. The residue was diluted with 1,4-dioxane-H_2O-EtOH (2/2/1, v/v/v, 1.25 mL), and then to the resulting mixture were added NaOAc (2.6 mg, 31.7 μmol) and hydroxylamine hydrochloride (1.2 mg, 18.6 μmol) at room temperature. After the reaction mixture was stirred for 3 h at 60 ºC, the reaction mixture was concentrated in *vacuo*. Purification of the residue by preparative TLC (2/1 *n*-hexane/EtOAc) gave **121** (8.6 mg, 10.8 μmol, 86% yield in 2 steps).

Data for **121**: Colorless syrup; R_f 0.41 (2/1 *n*-hexane/EtOAc); $[\alpha]^{25}_D$ +26.0° (*c* 1.0, $CHCl_3$); ^{1}H-NMR (400 MHz, $CDCl_3$) δ 7.84 (1H, br-s), 7.38-7.24 (13H, m), 7.16-7.11 (2H, m), 6.12 (1H, s), 5.52 (1H, s), 4.91 (1H, d, *J*=12.4 Hz), 4.91 (1H, d, *J*=10.8 Hz), 4.62-4.45 (7H, m), 4.40 (1H, m), 4.36 (1H, d, *J*=9.2 Hz), 4.31 (1H, m), 4.19 (1H, m), 4.15 (1H, m), 4.13-4.06 (2H, m), 3.76 (1H, dd, *J*=9.2 Hz, *J*=9.2 Hz), 3.71-3.62 (2H, m), 3.20 (3H, s), 0.93 (9H, s), 0.13 (6H, s); ^{13}C-NMR (125 MHz, $CDCl_3$)

δ 151.8, 138.0, 137.7, 137.6, 128.4, 128.3, 127.8×3, 127.7×2, 102,9, 96.0, 90.8, 78.6, 78.3, 74.9, 73.7, 73.5×2, 73.0, 72.9, 72.7, 71.7, 69.3, 68.4, 61.2, 55.8, 25.9, 18.4, -4.70, -4.73; HRMS (ESI-TOF) *m/z* 832.3102 (832.3131 calcd for $C_{42}H_{55}NO_{12}SiK$ $[M+K]^+$).

6-*O*-(2-Amino-3,4,6-tri-*O*-benzyl-2-deoxy-α-D-glucopyranosyl)-2-*O*-*tert*-butyldimethylsilyl-4-*O*-methoxymethyl-D-*myo*-inositol-1,3,5-orthoformate (54)

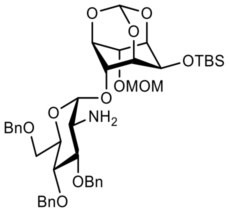

54

To a solution of **121** (8.7 mg, 11.0 µmol) in MeOH (5.5 mL) was added $NiCl_2 \cdot 6H_2O$ (5.2 mg, 21.9 µmol) at room temperature, followed by $NaBH_4$ (4.2 mg, 0.111 mmol) at −80 ºC. Then $NiCl_2 \cdot 6H_2O$ (52.0 mg, 0.219 mmol) and $NaBH_4$ (42.0 mg, 1.11 mmol) were added to the reaction mixture over 30 h. The reaction mixture was filtered through celite pad, and the filtrate was concentrated in *vacuo*. To the residue was added H_2O (2 mL). The aqueous layer was extracted with EtOAc (2 mL×6), and then the combined extracts were washed with brine (2 mL), dried over anhydrous Na_2SO_4, and concentrated in *vacuo*. Purification of the residue by preparative TLC (35/1 $CHCl_3$/MeOH) gave **54** (3.4 mg, 4.36 µmol, 40% yield) and unreacted substrate **121** (2.1 mg, 2.64 µmol, 24% yield).

Data for **54**: Colorless syrup; R_f 0.51 (20/1 $CHCl_3$/MeOH); $[\alpha]^{25}_D$ +79.8° (*c* 0.63, $CHCl_3$); ^{1}H-NMR (400 MHz, $CDCl_3$) δ 7.37-7.26 (13H, m), 7.18-7.14 (2H, m), 5.52 (1H, d, *J*=0.8 Hz), 4.96 (1H, d, *J*=3.6 Hz, H-1), 4.92 (1H, d, *J*=11.6 Hz), 4.78 (1H, d, *J*=11.2 Hz), 4.68-4.60 (3H, m), 4.56-4.49 (4H, m), 4.43 (1H, m), 4.27 (1H, m), 4.18-4.14 (2H, m), 4.12 (1H, m), 3.95 (1H, m), 3.72 (1H, dd, *J*=3.6 Hz, *J*=10.8 Hz), 3.66 (1H, dd, *J*=2.4 Hz, *J*=10.8 Hz), 3.63 (1H, dd, *J*=9.6 Hz, *J*=9.6 Hz), 3.39 (1H, dd, *J*=9.6 Hz, *J*=9.6 Hz), 3.31 (3H, s), 2.79 (1H, dd, *J*=3.6 Hz, *J*=9.6 Hz), 1.44 (2H, br-s), 0.93 (9H, s), 0.12 (6H, s); ^{13}C-NMR (100 MHz, $CDCl_3$) δ 138.4, 138.1, 137.7, 128.5, 128.4×2, 127.9, 127.8, 127.7, 103.0, 99.2, 96.1, 83.5, 78.6, 75.2, 74.6, 73.7, 73.6, 72.8, 72.4, 72.0, 71.7, 69.2, 68.4, 61.2, 55.9, 55.5, 25.9, 18.3, -4.57, -4.62; HRMS (ESI-TOF) *m/z* 780.3755 (780.3779 calcd for $C_{42}H_{58}NO_{11}Si$ $[M+H]^+$).

Synthesis of Common Mannosyl Structure 55β

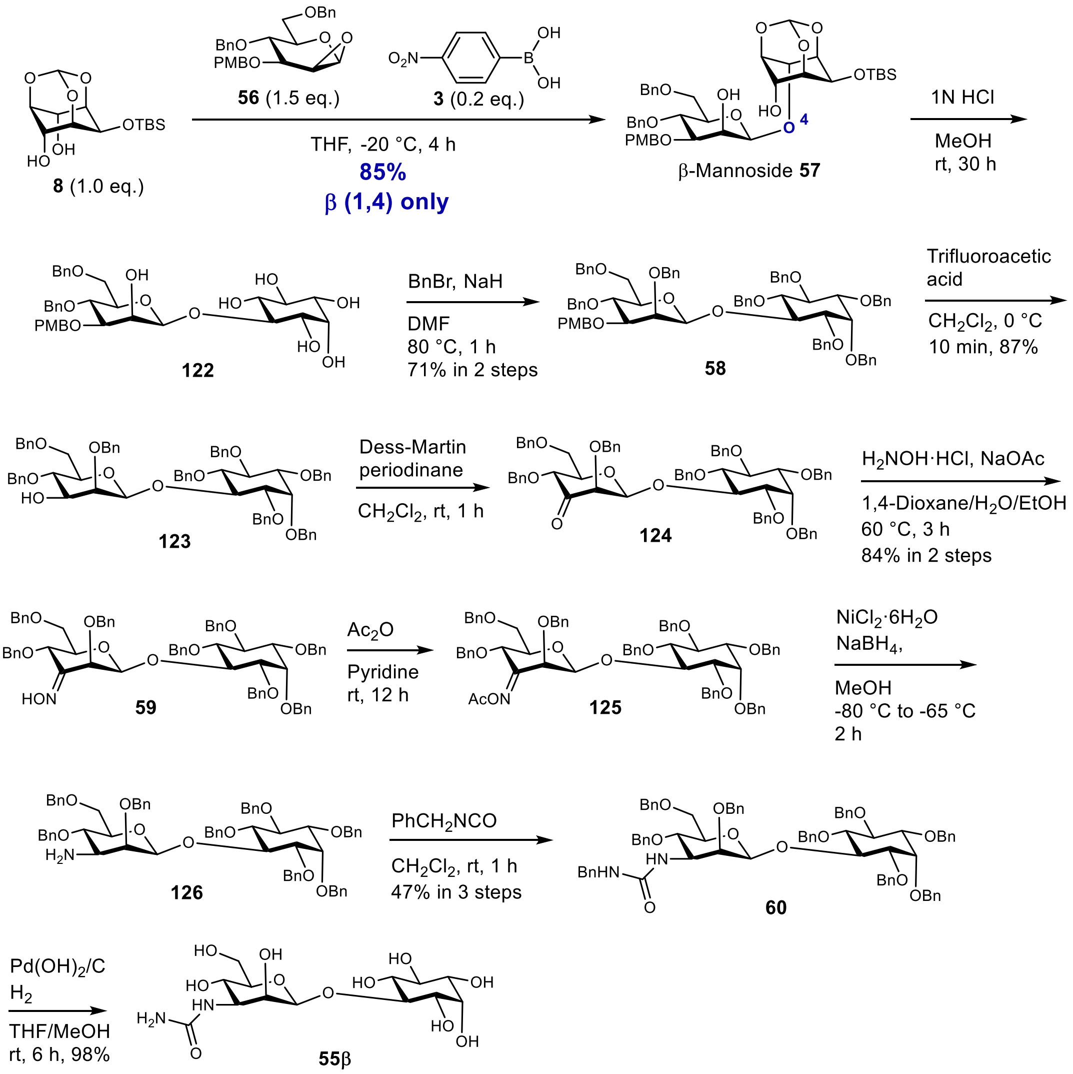

Supplementary Figure 40. Synthetic scheme of mannoside 55β.

4-*O*-(4,6-Di-*O*-benzyl-3-*O*-*p*-methoxybenzyl-β-D-mannopyranosyl)-2-*O*-*tert*-butyldimethylsilyl-D-*myo*-inositol-1,3,5-orthoformate (57)

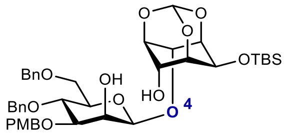

57

To a solution of **8** (10.8 mg, 35.5 μmol) and *p*-nitrophenylboronic acid (**3**) (1.19 mg, 7.10 μmol) in dry THF (267 μL) was added a solution of **56** (24.6 mg, 53.5 μmol) in dry THF (267 μL) at −20 ºC under Ar atmosphere. After the reaction mixture was stirred for 4 h, the reaction was quenched by addition of 0.05 M $NaBO_3$ aq. (0.254 mL, 12.7 μmol). To the resultant mixture was added sat. NH_4Cl

aq. (2 mL). The aqueous layer was extracted with EtOAc (3 mL×3), and then the combined extracts were washed with brine (5 mL), dried over anhydrous Na_2SO_4, and concentrated in *vacuo*. Purification of the residue by silica gel column chromatography (6/1 $CHCl_3$/EtOAc) gave **57** (23.0 mg, 30.0 μmol, 85% yield).

Data for **57**: Colorless syrup; R_f 0.57 (1/1 *n*-hexane/EtOAc); $[\alpha]^{26}_D$ −7.5° (*c* 1.0, $CHCl_3$); ^{1}H-NMR (500 MHz, $CDCl_3$) δ 7.36-7.20 (12H, m), 6.87-6.85 (2H, m), 5.51 (1H, s), 4.81 and 4.53 (2H, ABq, *J*=11.5 Hz), 4.64-4.52 (7H, m), 4.48 (1H, m), 4.26 (1H, m), 4.13 (1H, m), 4.10 (1H, m), 3.98 (1H, br-s), 3.86 (1H, d, *J*=7.5 Hz), 3.80 (3H, s), 3.77 (1H, dd, *J*=9.0 Hz, *J*=9.0 Hz), 3.71 (1H, br-d, *J*=10.5 Hz), 3.66 (1H, dd, *J*=3.5 Hz, *J*=10.5 Hz), 3.57 (1H, m), 3.46 (1H, m), 2.39 (1H, br-s), 0.94 (9H, s), 0.15 (6H, s); ^{13}C-NMR (125 MHz, $CDCl_3$) δ 159.5, 137.9, 129.6, 129.4, 128.4×2, 128.0, 127.9, 127.7, 114.0, 102.5, 99.6 ($^1J_{CH}$=159 Hz), 80.6, 75.2, 75.0, 74.8, 74.3, 73.8, 73.4, 72.8, 71.6, 69.3, 68.7, 68.2, 67.8, 61.0, 55.2, 25.9, 18.4, −4.6, −4.7; HRMS (ESI-TOF) *m/z* 789.3305 (789.3282 calcd for $C_{41}H_{54}O_{12}SiNa$ $[M+Na]^+$).

1,2,3,5,6-*O*-Penta-benzyl-4-*O*-(2,4,6-tri-*O*-benzyl-3-*O*-*p*-methoxybenzyl-β-D-mannopyranosyl)-D-*myo*-inositol (58)

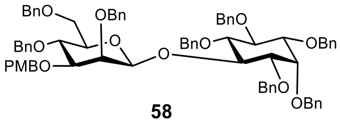

58

To a solution of **57** (1.19 g, 1.55 mmol) in MeOH (31.1 mL) was added 1N HCl aq. (7.73 mL) at room temperature. After the reaction mixture was stirred for 30 h, the reaction mixture was neutralized by addition of sat. $NaHCO_3$ aq. The resultant mixture was dried over anhydrous Na_2SO_4 and concentrated in *vacuo*. Purification of the residue by silica gel column chromatography (4/1 $CHCl_3$/MeOH) gave crude **122** (771 mg).

To a solution of crude **122** (771 mg) in DMF (12.0 mL) was added NaH (960 mg, 60% dispersion in mineral oil, 24.0 mmol) at 0 ºC, followed by BnBr (2.85 mL, 24.0 mmol). After the reaction mixture was stirred for 1 h at 80 ºC, the reaction was quenched by addition of H_2O (3 mL). To the resultant mixture was added H_2O (30 mL). The aqueous layer was extracted with EtOAc (50 mL×3), and then the combined extracts were washed with brine (30 mL), dried over anhydrous Na_2SO_4, and concentrated in *vacuo*. Purification of the residue by silica gel column chromatography (4/1 *n*-hexane/EtOAc) gave **58** (1.30 g, 1.10 mmol, 71% yield in 2 steps).

Data for **58**: Colorless syrup; R_f 0.46 (3/1 *n*-hexane/EtOAc); $[\alpha]^{28}_{D}$ −5.0° (*c* 1.0, $CHCl_3$); ^{1}H-NMR (500 MHz, $CDCl_3$) δ 7.42-7.14 (42H, m), 6.82-6.80 (2H, m), 5.34 and 4.66 (2H, ABq, *J*=11.0 Hz), 4.89-4.79 (6H, m), 4.81 and 4.51 (2H, ABq, *J*=10.5 Hz), 4.76 (1H, br-s), 4.69 and 4.62 (2H, ABq, *J*=11.5 Hz), 4.48 and 4.41 (2H, ABq, *J*=12.0 Hz), 4.43 and 4.05 (2H, ABq, *J*=11.0 Hz), 4.26 (1H, dd, *J*=9.0 Hz, *J*=9.0 Hz), 4.12 and 4.09 (2H, ABq, *J*=11.0 Hz), 4.05-4.01 (2H, m), 3.91 (1H, dd, *J*=9.5 Hz, *J*=9.5 Hz), 3.78 (3H, s), 3.77 (1H, dd, *J*=1.5 Hz, *J*=11.0 Hz), 3.72 (1H, br-s), 3.70 (1H, dd, *J*=4.5 Hz, *J*=11.0 Hz), 3.46 (1H, dd, *J*=9.0 Hz, *J*=9.0 Hz), 3.39 (1H, m), 3.37 (1H, dd, *J*=2.5 Hz, *J*=10.0 Hz), 3.26 (1H, dd, *J*=3.0 Hz, *J*=9.5 Hz), 3.14 (1H, dd, *J*=2.0 Hz, *J*=9.0 Hz); ^{13}C-NMR (125 MHz, $CDCl_3$) δ 159.0, 139.5, 139.2, 138.9, 138.8, 138.7, 138.6, 138.4, 138.0, 130.6, 129.0, 128.5, 128.4, 128.2×2, 128.1×2, 128.0×2, 127.9, 127.7×2, 127.6×2, 127.5, 127.4, 127.2, 127.1, 126.9, 113.7, 102.8, 83.0, 82.5, 81.6, 81.4, 81.3, 80.8, 76.1, 75.9, 75.5, 75.0, 74.8, 74.2, 73.9, 73.5, 73.0, 72.9, 71.7, 71.2, 69.7, 55.2; HRMS (ESI-TOF) *m/z* 1221.5090 (1221.5130 calcd for $C_{76}H_{78}O_{12}K$ $[M+K]^+$).

1,2,3,5,6-*O*-Penta-benzyl-4-*O*-(2,4,6-tri-*O*-benzyl-β-D-mannopyranosyl)-D-*myo*-inositol (123)

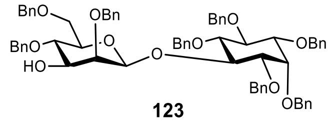

123

To a solution of **58** (73.2 mg, 61.8 μmol) in CH_2Cl_2 (2.78 mL) was added trifluoroacetic acid (309 μL) at 0 ºC. After the reaction mixture was stirred for 10 min, the reaction was quenched by addition of sat. $NaHCO_3$ aq. (2 mL). The aqueous layer was extracted with $CHCl_3$ (4 mL×3), and then the combined extracts were washed with brine (4 mL), dried over anhydrous Na_2SO_4, and concentrated in *vacuo*. Purification of the residue by silica gel column chromatography (3/1 *n*-hexane/EtOAc) gave **123** (57.1 mg, 53.7 μmol, 87% yield).

Data for **123**: Colorless syrup; R_f 0.30 (3/1 *n*-hexane/EtOAc); $[\alpha]^{19}_{D}$ −7.4° (*c* 1.0, $CHCl_3$); ^{1}H-NMR (500 MHz, $CDCl_3$) δ 7.38-7.20 (38H, m), 7.15-7.14 (2H, m), 5.28 and 4.63 (2H, ABq, *J*=11.0 Hz), 5.03 and 4.59 (2H, ABq, *J*=11.5 Hz), 4.90 (1H, br-s), 4.87 and 4.82 (2H, ABq, *J*=12.5 Hz), 4.84 and 4.82 (2H, ABq, *J*=13.0 Hz), 4.82 and 4.53 (2H, ABq, *J*=11.5 Hz), 4.69 and 4.61 (2H, ABq, *J*=12.0 Hz), 4.52 and 4.30 (2H, ABq, *J*=11.5 Hz), 4.47 and 4.41 (2H, ABq, *J*=12.0 Hz), 4.31 (1H, dd, *J*=9.5 Hz, *J*=10.0 Hz), 4.05 (1H, dd, *J*=9.5 Hz, *J*=10.0 Hz), 4.03 (1H, br-s), 3.78 (1H, dd, *J*=1.0 Hz, *J*=11.5 Hz), 3.68 (1H, dd, *J*=5.0 Hz, *J*=11.5 Hz), 3.65 (1H, br-d, *J*=3.5 Hz), 3.62 (1H, dd, *J*=9.0 Hz, *J*=9.0 Hz), 3.47 (1H, ddd, *J*=3.5 Hz, *J*=9.0 Hz, *J*=10.0 Hz), 3.43 (1H, dd, *J*=9.5 Hz, *J*=9.5 Hz), 3.36 (1H, dd, *J*=2.0 Hz, *J*=10.0 Hz), 3.35 (1H, m), 3.27 (1H, dd, *J*=2.0 Hz, *J*=10.0 Hz), 2.33 (1H, d, *J*=10.0 Hz); ^{13}C-NMR (125 MHz, $CDCl_3$) δ 139.4, 138.9, 138.7, 138.6, 138.5, 138.4, 137.6, 128.6, 128.4,

128.3, 128.2×2, 128.1, 128.0, 127.9, 127.8×2, 127.6×2, 127.4×2, 127.2, 127.0, 102.5, 82.8, 81.4, 81.2, 80.7, 80.5, 78.3, 76.9, 75.9, 75.6, 75.4, 74.7×2, 74.3, 73.9, 73.5, 73.0, 72.9, 72.0, 69.6; HRMS (ESI-TOF) *m/z* 1063.4949 (1063.4996 calcd for $C_{68}H_{71}O_{11}$ $[M+H]^+$).

1,2,3,5,6-*O*-Penta-benzyl-4-*O*-(2,4,6-tri-*O*-benzyl-3-oximino-β-D-mannopyranosyl)-D-*myo*-inositol (59)

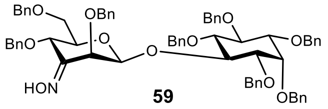

59

To a solution of **123** (57.1 mg, 53.7 μmol) in CH_2Cl_2 (2.68 mL) was added Dess-Martin periodinane (68.3 mg, 0.161 mmol) at 0 ºC under Ar atmosphere. After the reaction mixture was stirred for 1 h at room temperature, the reaction was filtered through celite pad, and the filtrate was concentrated in *vacuo*. Purification of the residue by silica gel column chromatography (4/1 *n*-hexane/EtOAc) gave crude **124** (50.6 mg).

To a solution of crude **124** (50.6 mg) in 1,4-dioxane-H_2O-EtOH (2/2/1, v/v/v, 1.90 mL) were added NaOAc (9.8 mg, 0.119 mmol) and hydroxylamine hydrochloride (5.0 mg, 71.6 μmol) at room temperature. After the reaction mixture was stirred for 3 h at 60 ºC, the reaction mixture was concentrated in *vacuo*. Purification of the residue by silica gel column chromatography (2/1 *n*-hexane/EtOAc) gave **59** (48.4 mg, 45.0 μmol, 84% in 2 steps).

Data for **59**: Colorless syrup; R_f 0.53 (2/1 *n*-hexane/EtOAc); $[\alpha]^{21}_D$ +19.9° (*c* 1.0, $CHCl_3$); ^{1}H-NMR (500 MHz, $CDCl_3$) δ7.63 (1H, br-s), 7.34-7.18 (37H, m), 7.14-7.11 (3H, m), 5.30 and 4.71 (2H, ABq, *J*=11.5 Hz), 5.19 (1H, d, *J*=1.5 Hz), 4.92 (1H, d, *J*=1.5 Hz), 4.81 (2H, s), 4.78 and 4.75 (2H, ABq, *J*=12.0 Hz), 4.68 and 4.38 (2H, ABq, *J*=11.0 Hz), 4.65 and 4.57 (2H, ABq, *J*=11.5 Hz), 4.58 and 4.53 (2H, ABq, *J*=12.5 Hz), 4.46 and 4.40 (2H, ABq, *J*=12.0 Hz), 4.45 (1H, d, *J*=9.5 Hz), 4.38 and 4.33 (2H, ABq, *J*=11.5 Hz), 4.32 (1H, dd, *J*=9.5 Hz, *J*=10.0 Hz), 4.01 (1H, dd, *J*=9.5 Hz, *J*=10.0 Hz), 3.94 (1H, m), 3.71 (1H, dd, *J*=1.5 Hz, *J*=11.5 Hz), 3.64 (1H, dd, *J*=4.5 Hz, *J*=11.5 Hz), 3.55 (1H, m), 3.48 (1H, dd, *J*=9.5 Hz, 9.5 Hz), 3.31 (1H, dd, *J*=2.0 Hz, *J*=10.0 Hz), 3.23 (1H, dd, *J*=2.0 Hz, *J*=10.0 Hz); ^{13}C-NMR (125 MHz, $CDCl_3$) δ154.8, 139.5, 138.9, 138.8, 138.7, 138.4, 138.3, 137.6×2, 128.4, 128.3, 128.2×2, 128.1×2, 128.0×2, 127.9, 127.7×2, 127.6, 127.4×2, 127.3, 127.1, 126.9, 101.8, 82.3, 81.2, 80.8, 80.6×2, 77.7, 75.9, 75.4, 73.9, 73.5×2, 73.2, 72.9, 72.4, 72.3, 71.6, 70.2, 69.1; HRMS (ESI-TOF) *m/z* 1076.4971 (1076.4949 calcd for $C_{68}H_{70}NO_{11}$ $[M+H]^+$).

1,2,3,5,6-*O*-Penta-benzyl-4-*O*-[2,4,6-tri-*O*-benzyl-3-deoxy-3-(*N*'-benzyl-ureido)-β-D-mannopyranosyl]-D-*myo*-inositol (60)

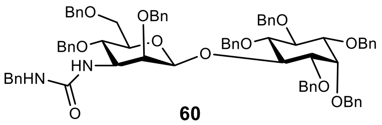

60

To a solution of **59** (8.5 mg, 7.60 μmol) in pyridine (570 μL) was added Ac_2O (190 μL) at room temperature under Ar atmosphere. After the reaction mixture was stirred for 12 h, the reaction was quenched by addition of H_2O (1 mL). The aqueous layer was extracted with EtOAc (3 mL×3), and then the combined extracts were washed with brine (5 mL), dried over anhydrous Na_2SO_4, and concentrated in *vacuo*. Purification of the residue by silica gel column chromatography (3/1 *n*-hexane/EtOAc) gave crude **125** (11.4 mg).

To a solution of crude **125** (11.4 mg) in MeOH (3.0 mL) was added $NiCl_2{\cdot}6H_2O$ (3.61 mg, 15.2 μmol) at room temperature, followed by $NaBH_4$ (2.90 mg, 76.0 μmol) at −80 °C. The reaction mixture was warmed to −65 °C, and then $NiCl_2{\cdot}6H_2O$ (3.61 mg, 15.2 μmol) and $NaBH_4$ (2.90 mg, 76.0 μmol) were added to the reaction mixture in 3 portions over 2 h. The reaction mixture was filtered through celite pad, and the filtrate was concentrated in *vacuo*. To the residue was added H_2O (1 mL). The aqueous layer was extracted with EtOAc (5 mL×6), and then the combined extracts were washed with brine (2 mL), dried over anhydrous Na_2SO_4, and concentrated in *vacuo*. Purification of the residue by preparative TLC (1/1 *n*-hexane/EtOAc) gave crude **126** (4.0 mg).

To a solution of crude **126** (4.0 mg) in CH_2Cl_2 (753 mL) was added benzyl isocyanate (0.69 μL, 5.65 μmol) at room temperature. After the reaction mixture was stirred for 1 h at room temperature, the reaction mixture was concentrated in *vacuo*. Purification of the residue by preparative TLC (1/1 *n*-hexane/EtOAc) gave **60** (4.3 mg, 3.60 μmol, 47% in 3 steps).

Data for **60**: Colorless syrup; R_f 0.24 (2/1 *n*-hexane/EtOAc); $[\alpha]^{22}_D$ −12.0° (*c* 1.13, $CHCl_3$); ^{1}H-NMR (500 MHz, $CDCl_3$) δ 7.39-7.38 (2H, m), 7.31-7.20 (37H, m), 7.13-7.10 (6H, m), 5.23 and 4.59 (2H, ABq, *J*=11.5 Hz), 5.00 and 4.44 (2H, ABq, *J*=11.5 Hz), 4.93 (1H, br-s), 4.87 and 4.81 (2H, ABq, *J*=12.0 Hz), 4.83 and 4.81 (2H, ABq, *J*=11.5 Hz), 4.68 and 4.60 (2H, ABq, *J*=12.0 Hz), 4.52 and 4.31 (2H, ABq, *J*=11.5 Hz), 4.50 and 4.46 (2H, ABq, *J*=11.5 Hz), 4.50 and 4.36 (2H, ABq, *J*=11.5 Hz), 4.31 (1H, m), 4.28 (1H, dd, *J*=9.5 Hz, *J*=9.5 Hz), 4.05 (1H, dd, *J*=9.5 Hz, *J*=9.5 Hz), 4.01 (1H, br-s), 3.96 (1H, m), 3.73 (1H, br-d, *J*=10.0 Hz), 3.69 (1H, dd, *J*=4.0 Hz, *J*=10.0 Hz), 3.66 (1H, dd, *J*=9.5 Hz, *J*=9.5 Hz), 3.57 (1H, br-d, *J*=2.5 Hz), 3.54 (1H, br-s), 3.42-3.38 (2H, m), 3.34 (1H, dd, *J*=2.5 Hz, *J*=9.5 Hz), 3.27 (1H, dd, *J*=2.0 Hz, *J*=9.5 Hz); ^{13}C-NMR (125 MHz, $CDCl_3$) δ 157.8, 139.4, 139.2, 138.9, 138.8, 138.6, 138.5, 138.3, 138.0, 137.5, 128.7, 128.5, 128.4×2, 128.3, 128.2×3, 128.1, 128.0,

127.9, 127.8, 127.7×2, 127.6, 127.4, 127.3, 127.2×2, 126.9, 102.9, 82.8, 81.2×2, 80.7, 80.5, 78.3, 76.5, 75.8, 75.3, 74.7, 74.4, 73.9, 73.5, 73.1, 72.9, 72.1, 69.4, 55.2, 44.5; HRMS (ESI-TOF) *m/z* 1195.5717 (1195.5684 calcd for $C_{76}H_{79}N_2O_{11}$ $[M+H]^+$).

4-*O*-(3-Deoxy-3-ureido-β-D-mannopyranosyl)-D-*myo*-inositol (55β)

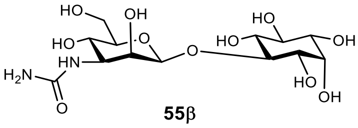

55β

To a solution of **60** (11.8 mg, 9.87 μmol) in THF (2.0 mL) was added 20% $Pd(OH)_2/C$ (11.8 mg, wetted with 50% water) at room temperature under Ar atmosphere. After changing the atmosphere to H_2 (balloon), the reaction mixture was stirred for 6 h. After changing the atmosphere to Ar, the reaction was filtered through celite pad, and the filtrate was concentrated in *vacuo* to give **55β** (3.7 mg, 9.63 μmol, 98% yield).

Data for **55β**: White solid; R_f 0.24 (3/3/1 *n*-butanol/MeOH/H_2O); $[\alpha]^{24}_D$ −31.7° (*c* 0.37, H_2O); mp 276-268 °C; ^{1}H-NMR (500 MHz, D_2O) δ 4.92 (1H, br-s), 4.02-4.00 (2H, m), 3.91 (1H, br-d, *J*=12.5 Hz), 3.81 (1H, t, *J*=9.5 Hz), 3.73-3.69 (2H, m), 3.66-3.62 (2H, m), 3.54-3.46 (3H, m), 3.38 (1H, t, *J*=9.5 Hz); ^{13}C-NMR (125 MHz, D_2O) δ 161.9, 101.6 ($^1J_{CH}$=160 Hz), 82.5, 78.2, 73.5, 73.1, 72.9, 71.8, 71.6, 70.5, 66.1, 61.9, 56.2; HRMS (ESI-TOF) *m/z* 385.1459 (384.1458 calcd for $C_{13}H_{25}N_2O_{11}$ $[M+H]^+$).

Supplementary Table 32. Comparison of ^{13}C-NMR data of 55β

δ (Literature data[22]) (25 MHz, $CDCl_3$)	δ (Mannoside **55**β derived from **57**) (125 MHz, $CDCl_3$)
Not mentioned	161.9
101.6	101.6
82.6	82.5
78.2	78.2
73.5	73.5
73.0×2	73.1
	72.9
72.1	71.8
71.9	71.6
70.5	70.5
66.2	66.1
61.7	61.9
56.1	56.2

DFT Calculations

All geometries were fully optimized using the B3LYP functional and the 6-31G* level of theory. All optimized geometries were verified by frequency calculations as minima (zero imaginary frequencies) or transition structures (a single imaginary frequency). Single-point energy calculations on the optimized geometries were then evaluated using B3LYP and the 6-31+G** basis set. The transition state structures were located using a standard eigenvector following method by using vibrational analysis. Intrinsic reaction coordinate (IRC) calculations were carried out in order to ensure that the TSs indeed connected the appropriate reactants and products.

Conformational searches were performed with Macromodel version 11.1[23] and the OPLS-3 force field. All quantum chemical calculations were performed using Jaguar version 9.1[24,25].

Geometries of 1,2-anhydro-D-glucose **1** and boronic acid **3** was already described in the previous paper[26].

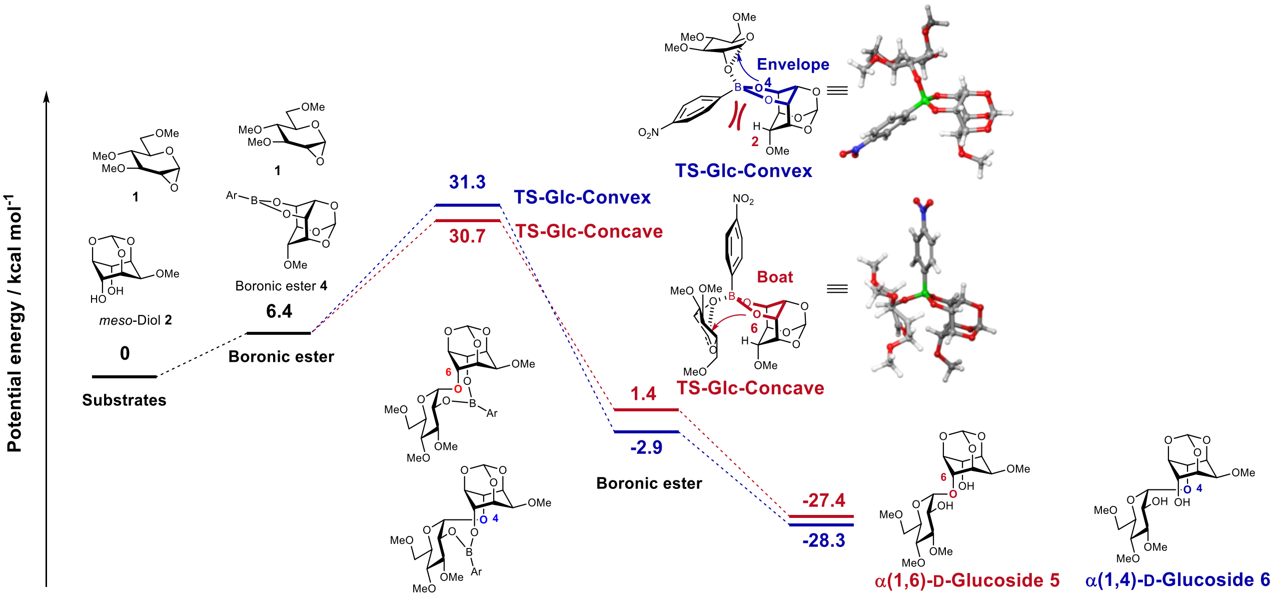

Supplementary Figure 41. Potential energy surfaces for the present desymmetric glycosylation.

NMR Spectral Charts

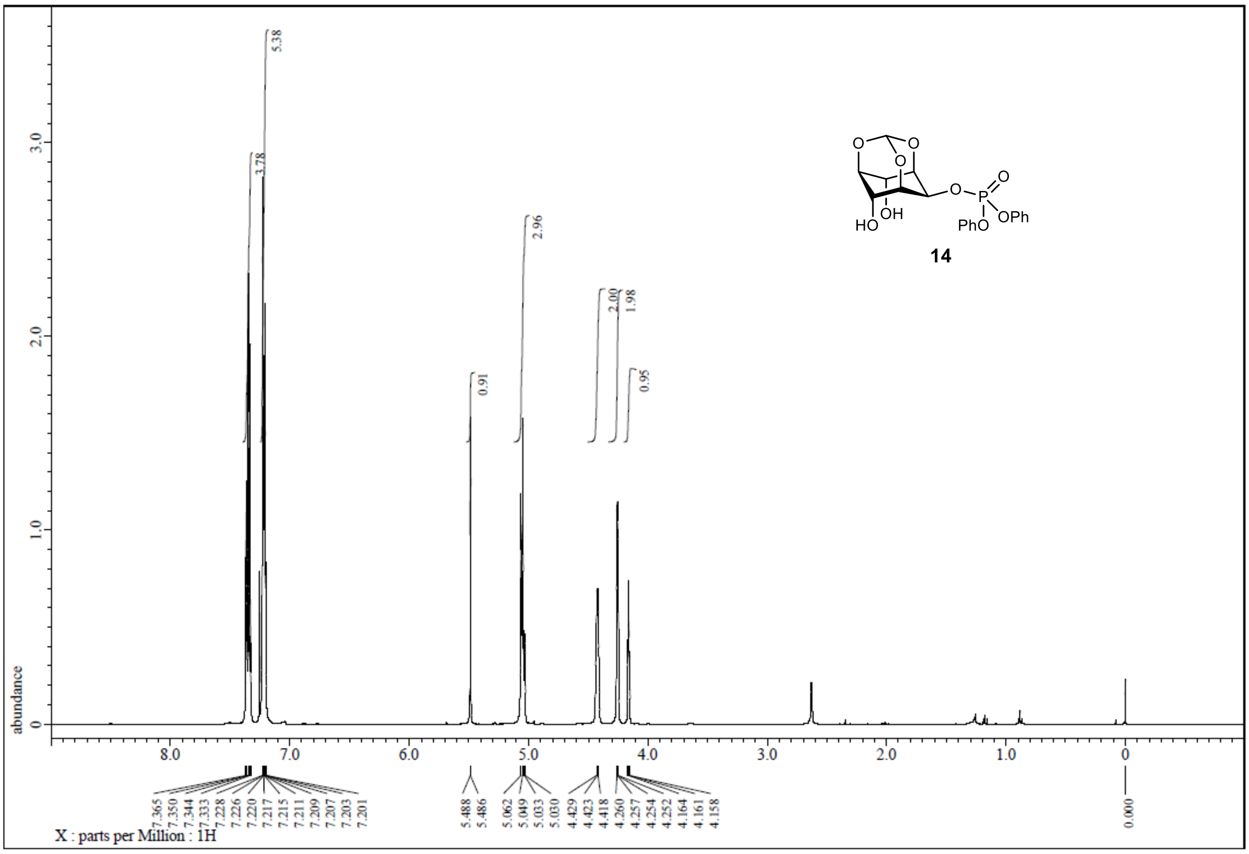

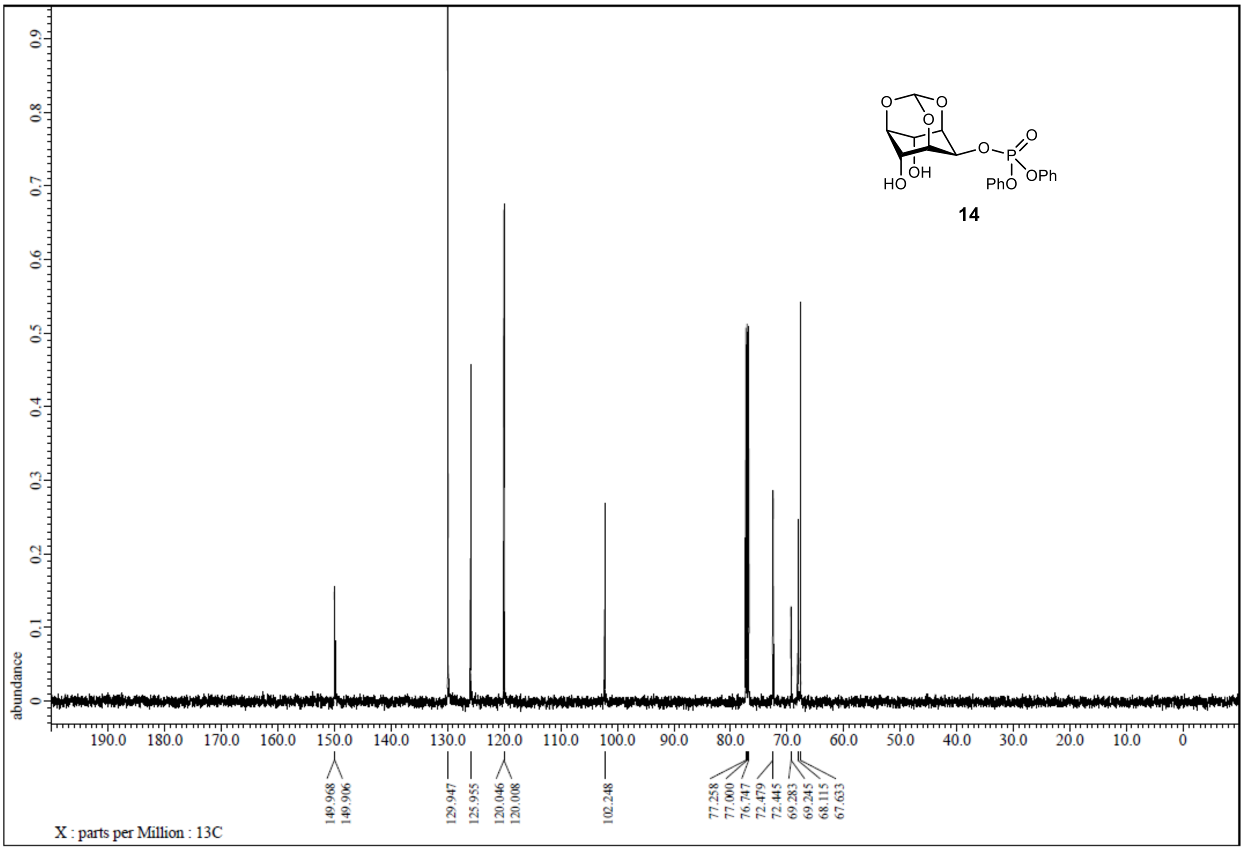

Supplementary Figure 42. ^{1}H and ^{13}C-NMR spectra of compound 14.

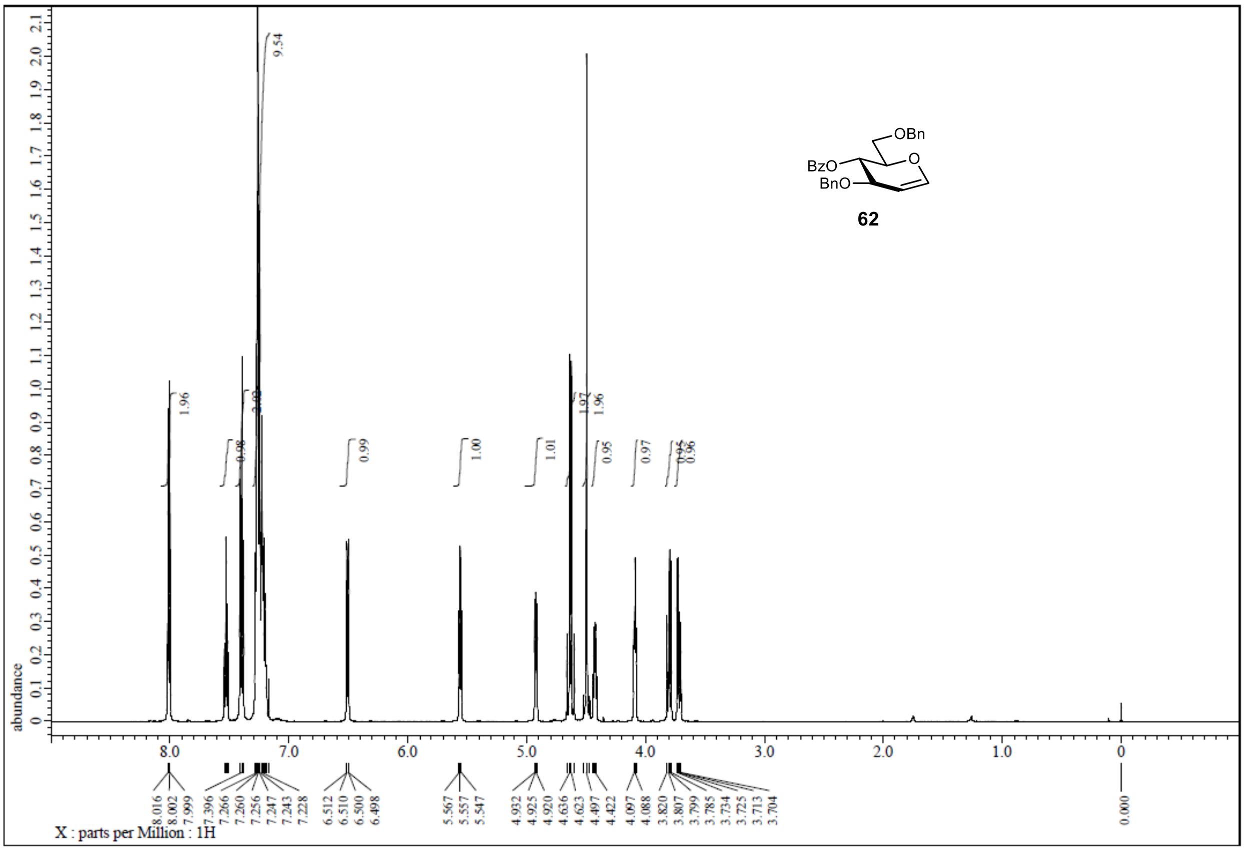

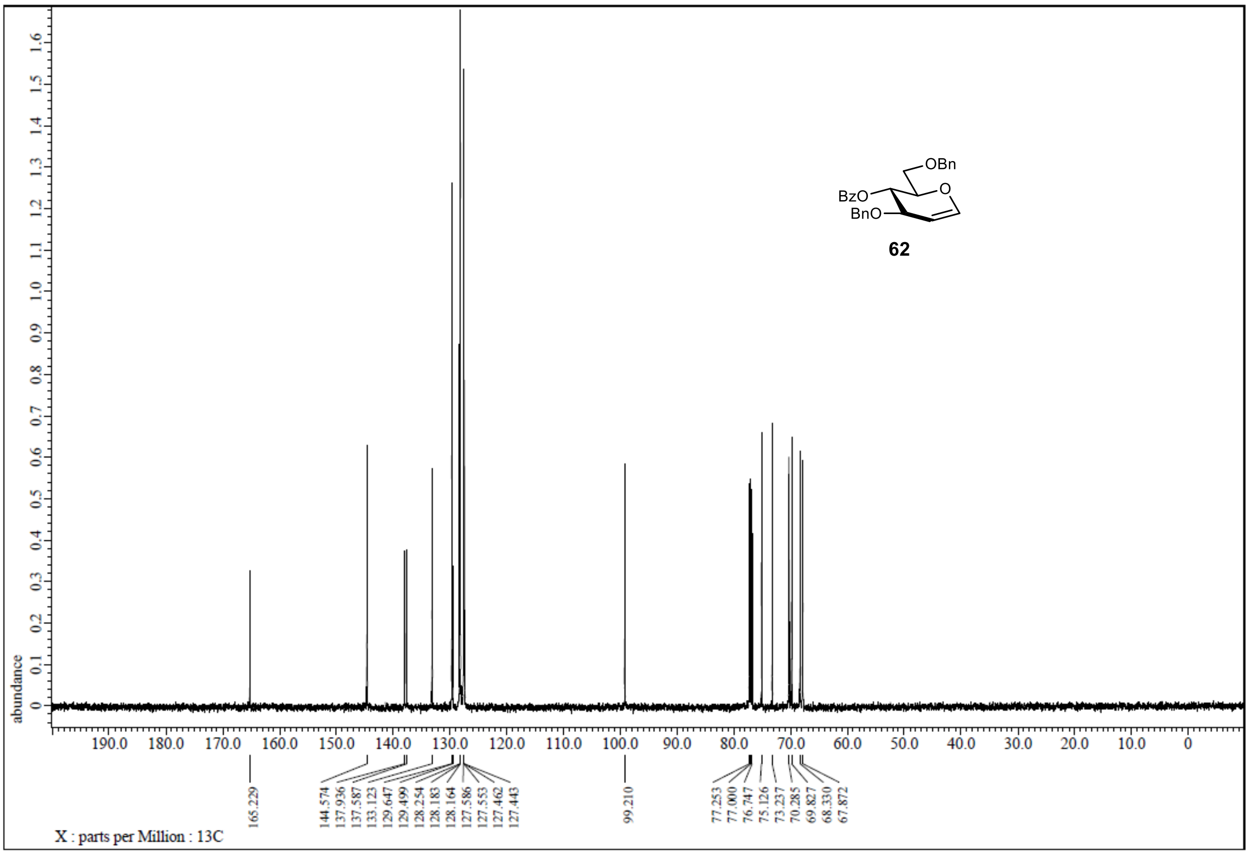

Supplementary Figure 43. ^{1}H and ^{13}C-NMR spectra of compound 62.

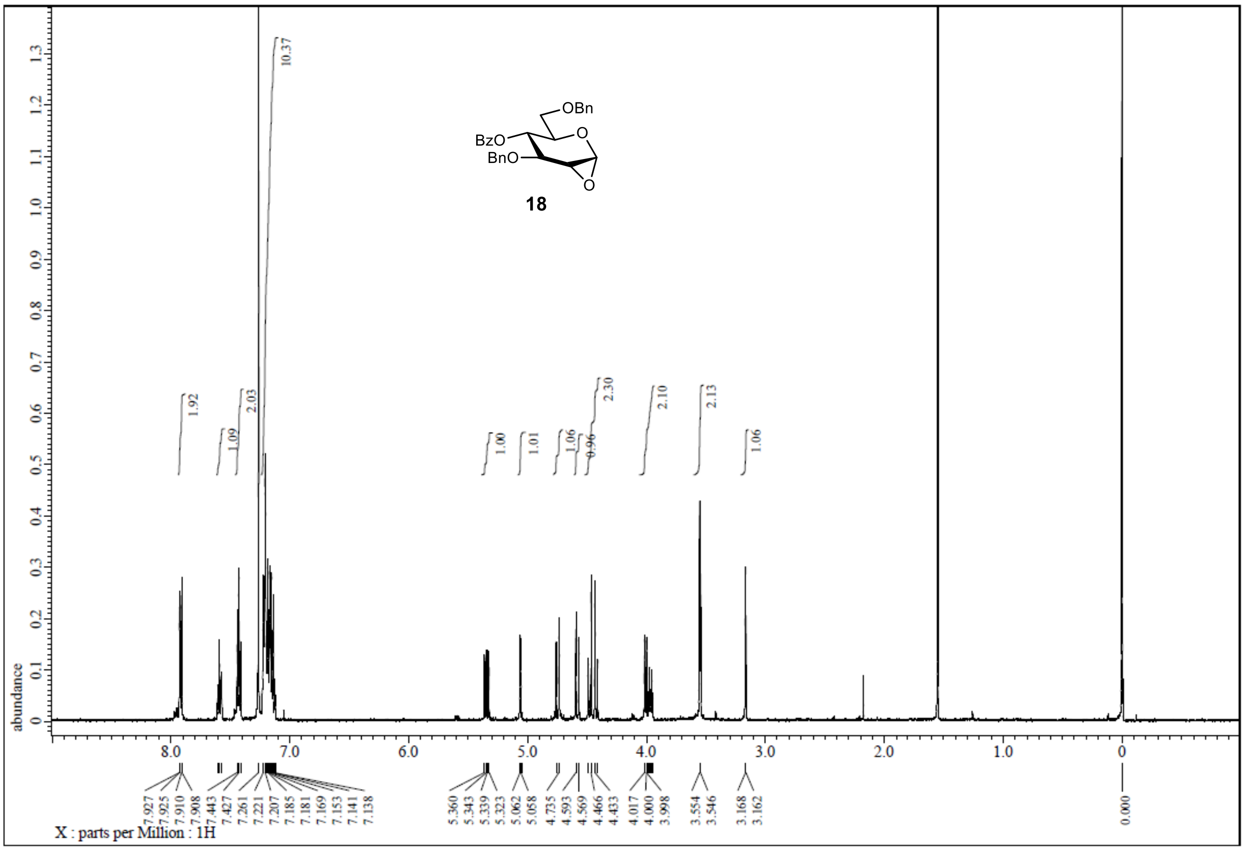

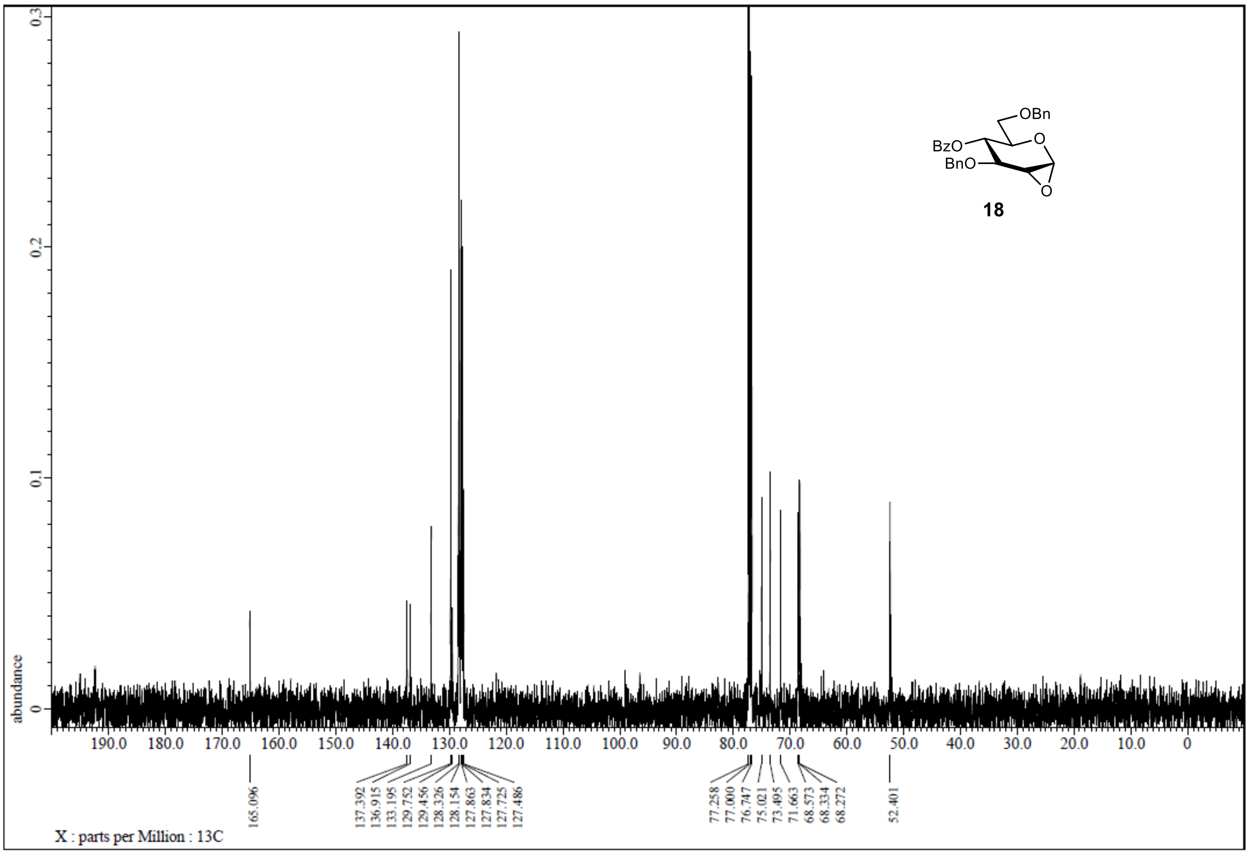

Supplementary Figure 44. ^{1}H and ^{13}C-NMR spectra of compound 18.

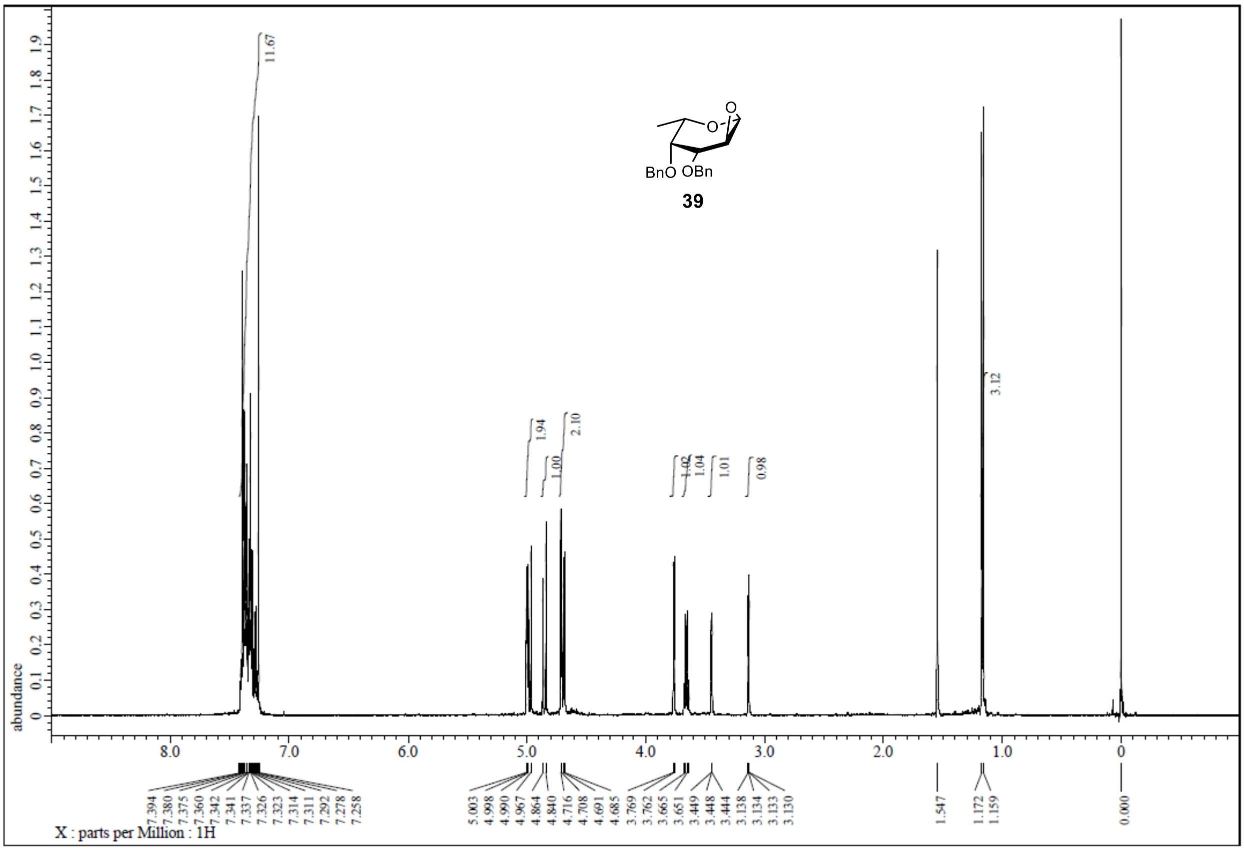

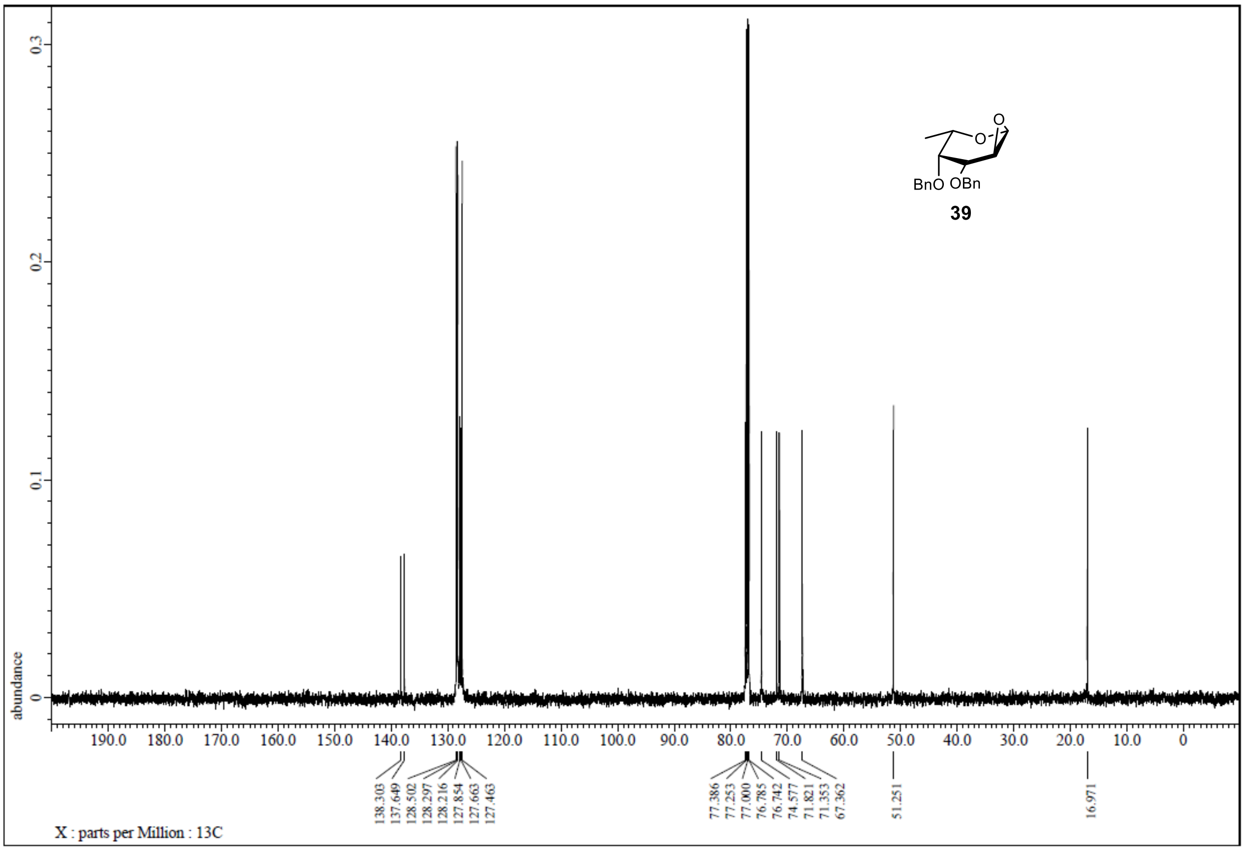

Supplementary Figure 45. ^{1}**H and** ^{13}**C-NMR spectra of compound 39.**

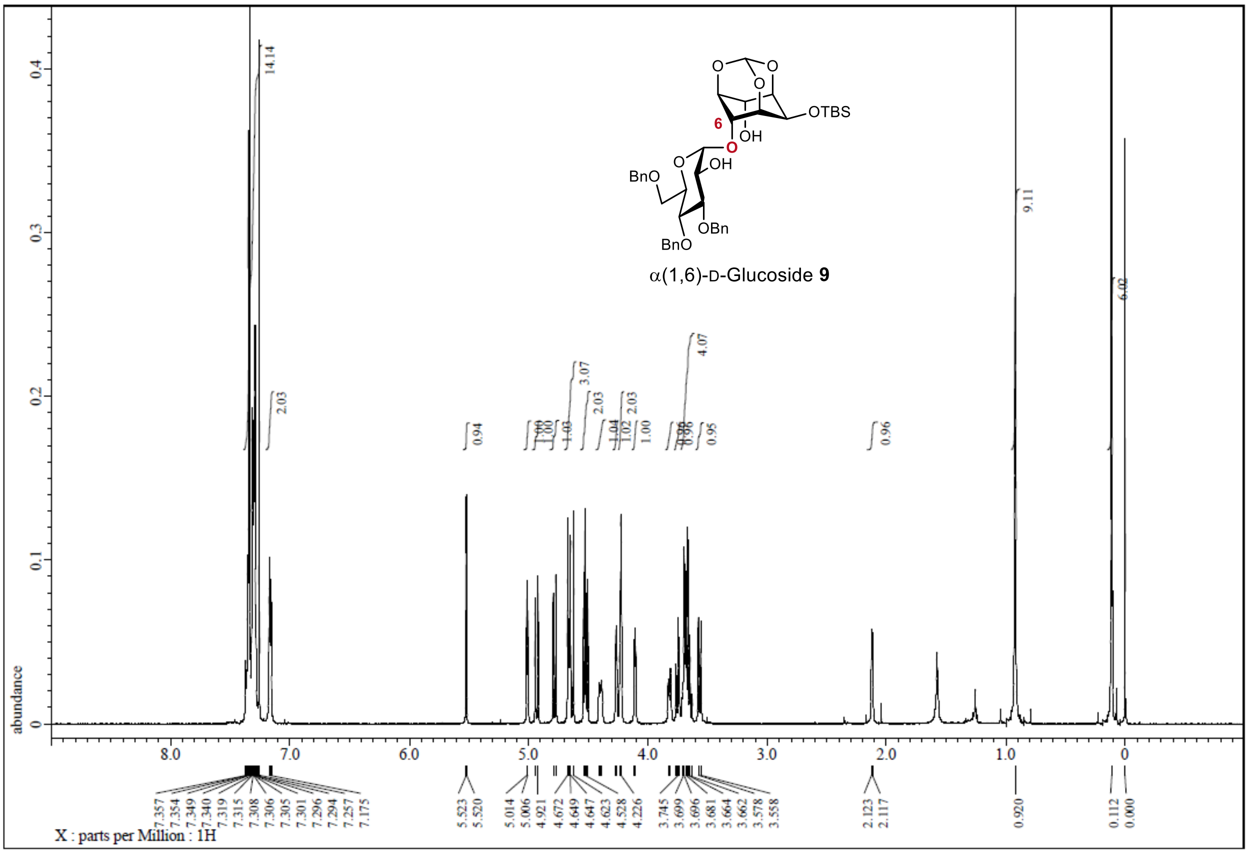

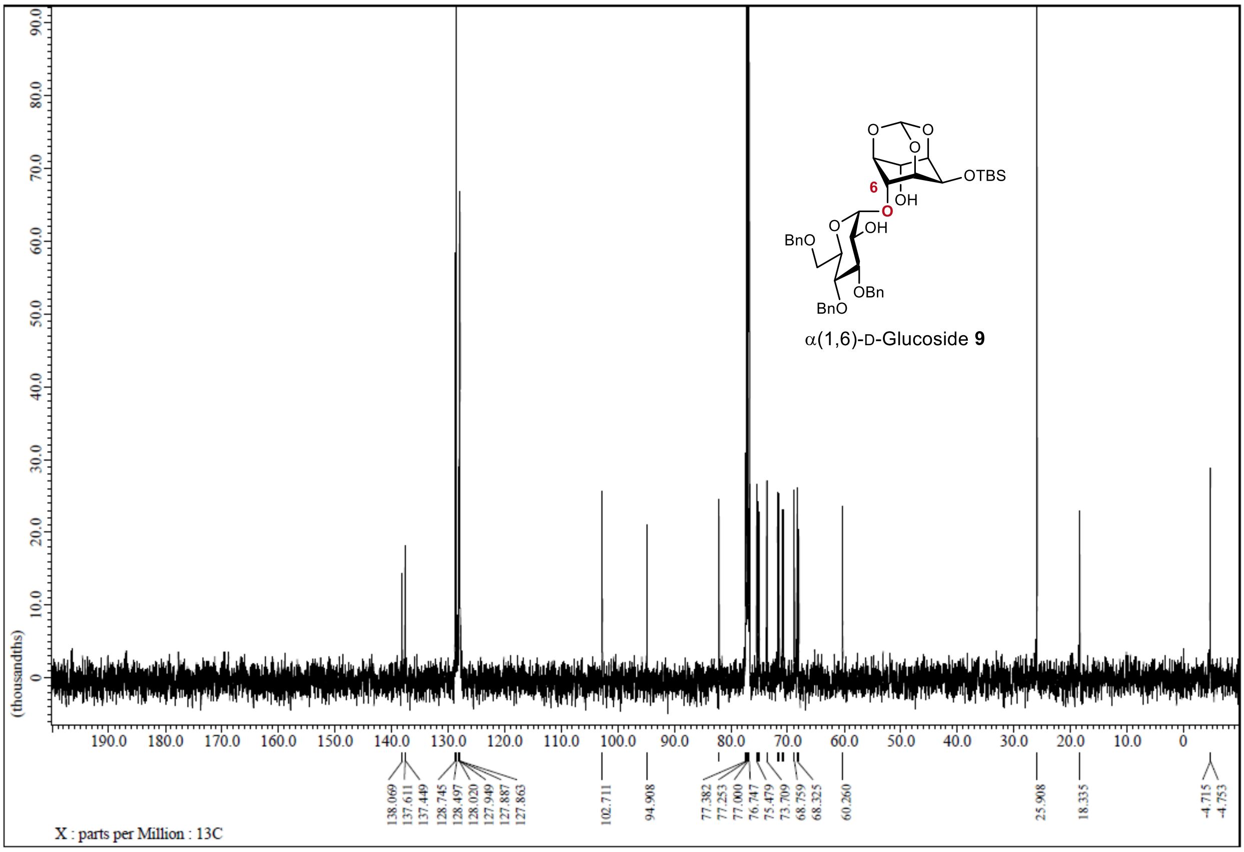

Supplementary Figure 46. ^{1}H and ^{13}C-NMR spectra of compound 9.

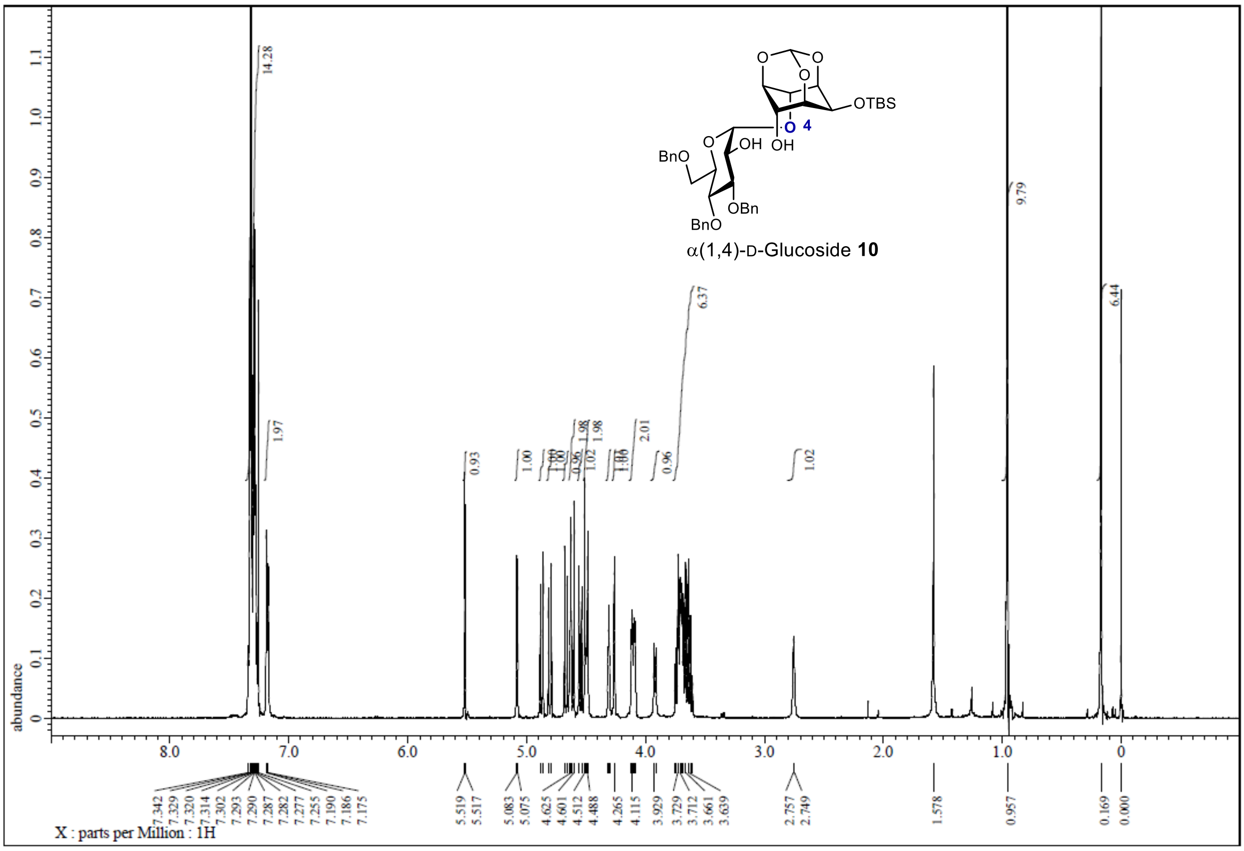

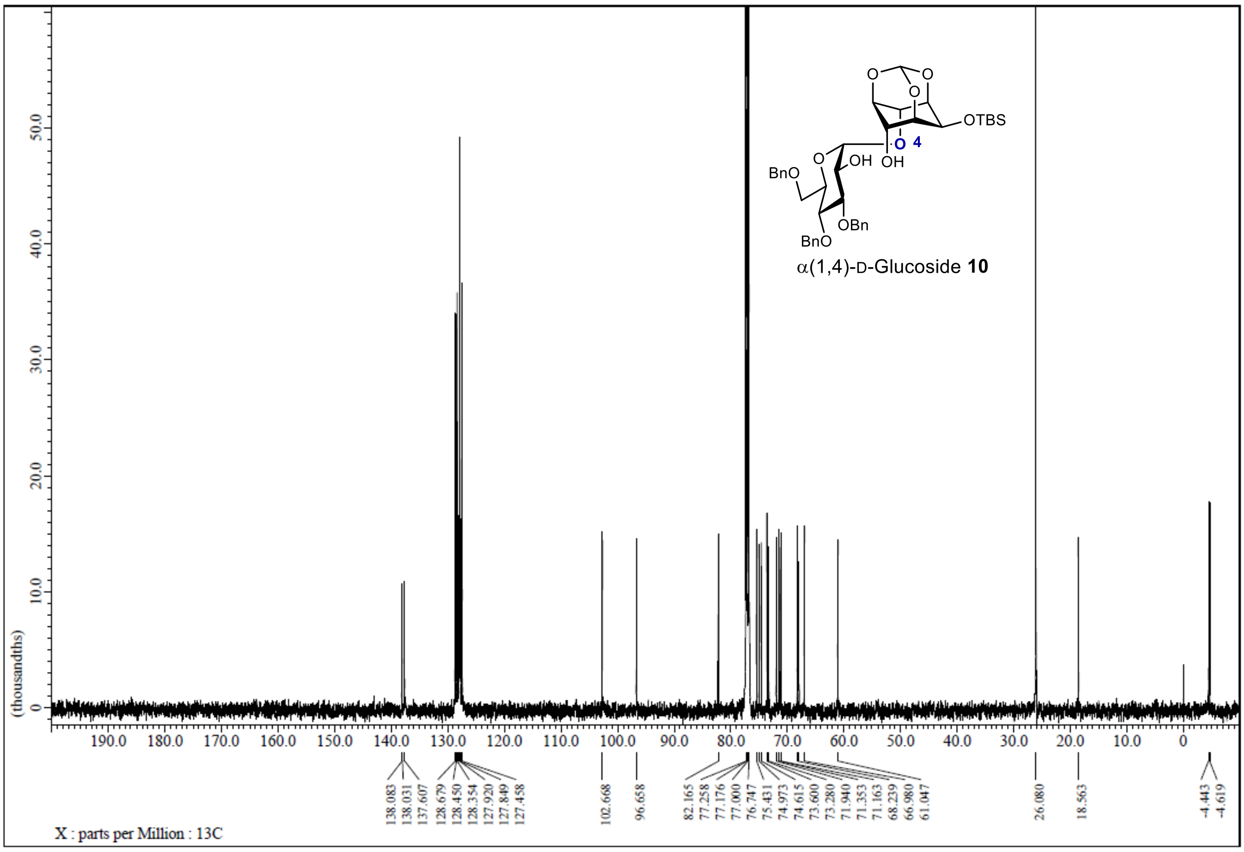

Supplementary Figure 47. ^{1}H and ^{13}C-NMR spectra of compound 10.

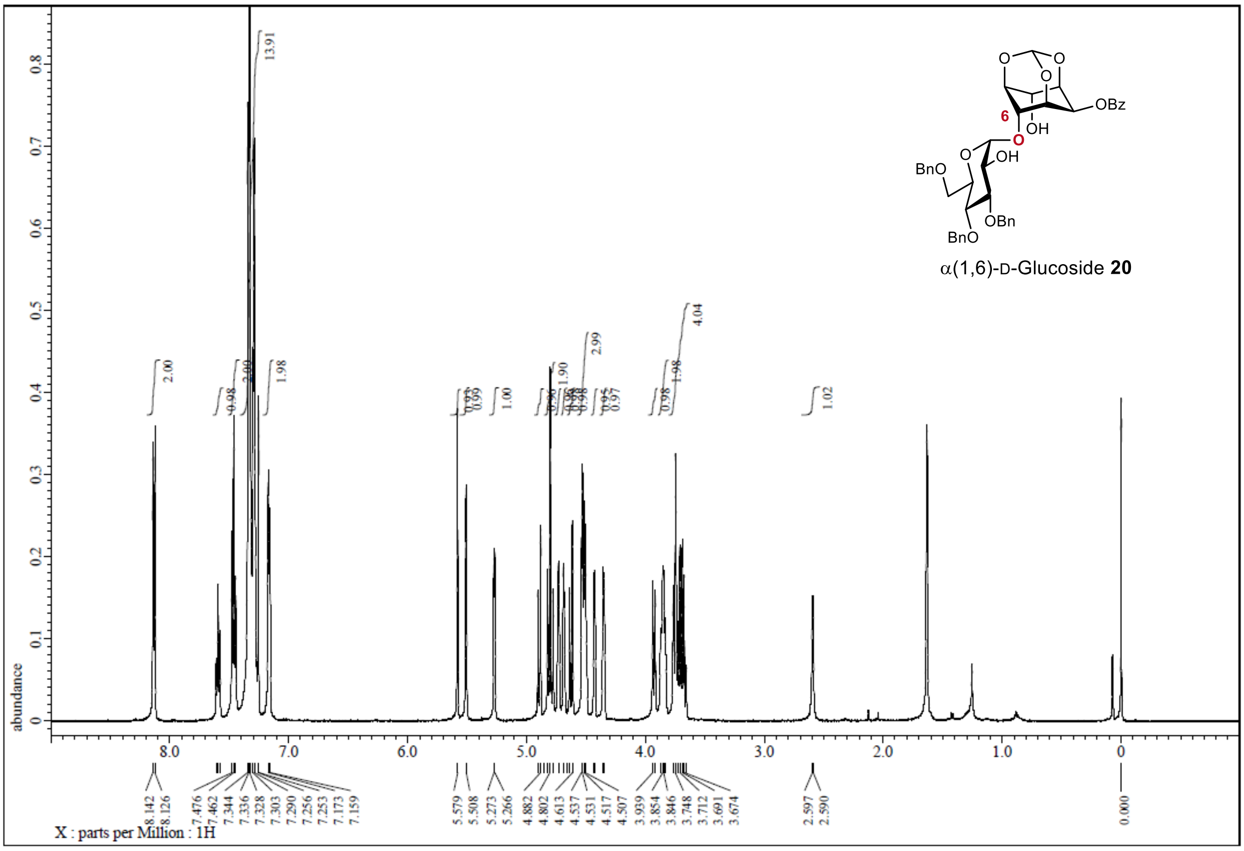

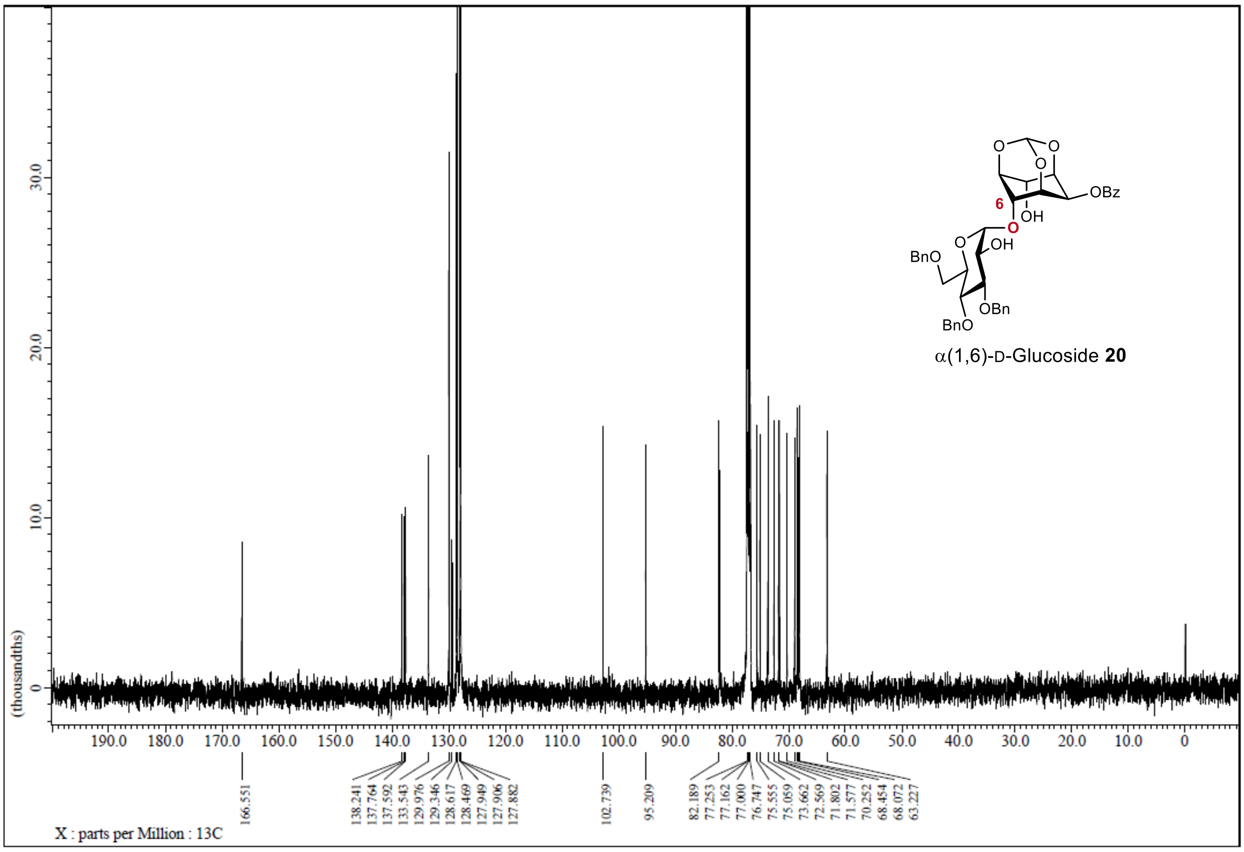

Supplementary Figure 48. ^{1}H and ^{13}C-NMR spectra of compound 20.

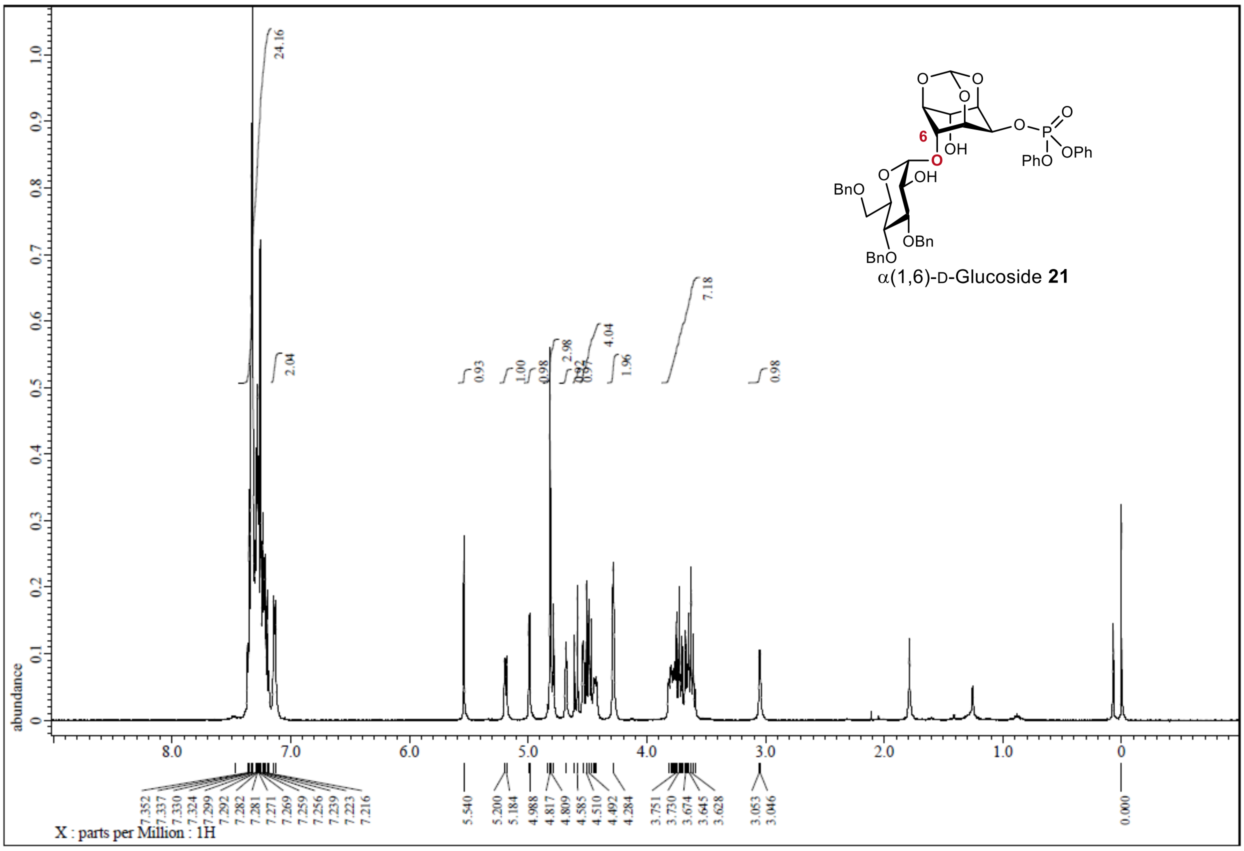

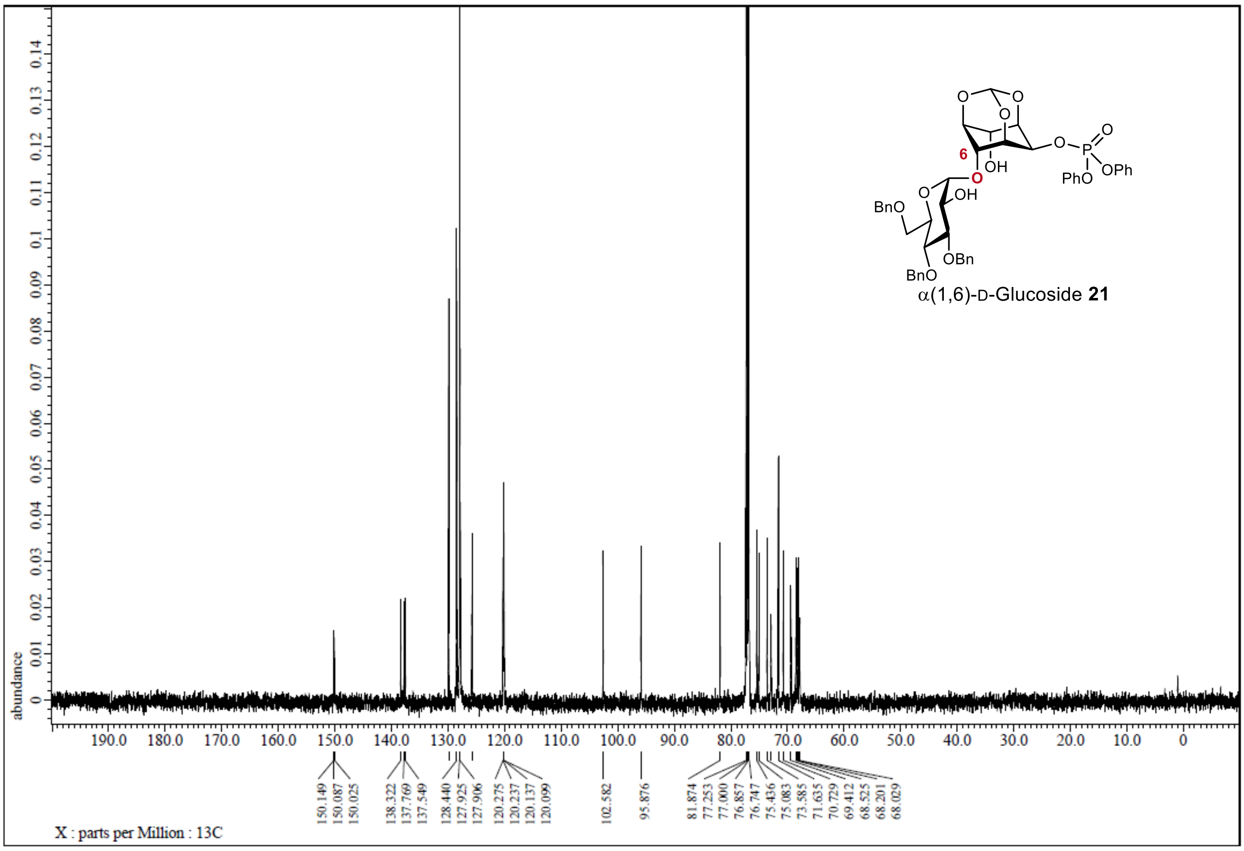

Supplementary Figure 49. ^{1}H and ^{13}C-NMR spectra of compound 21.

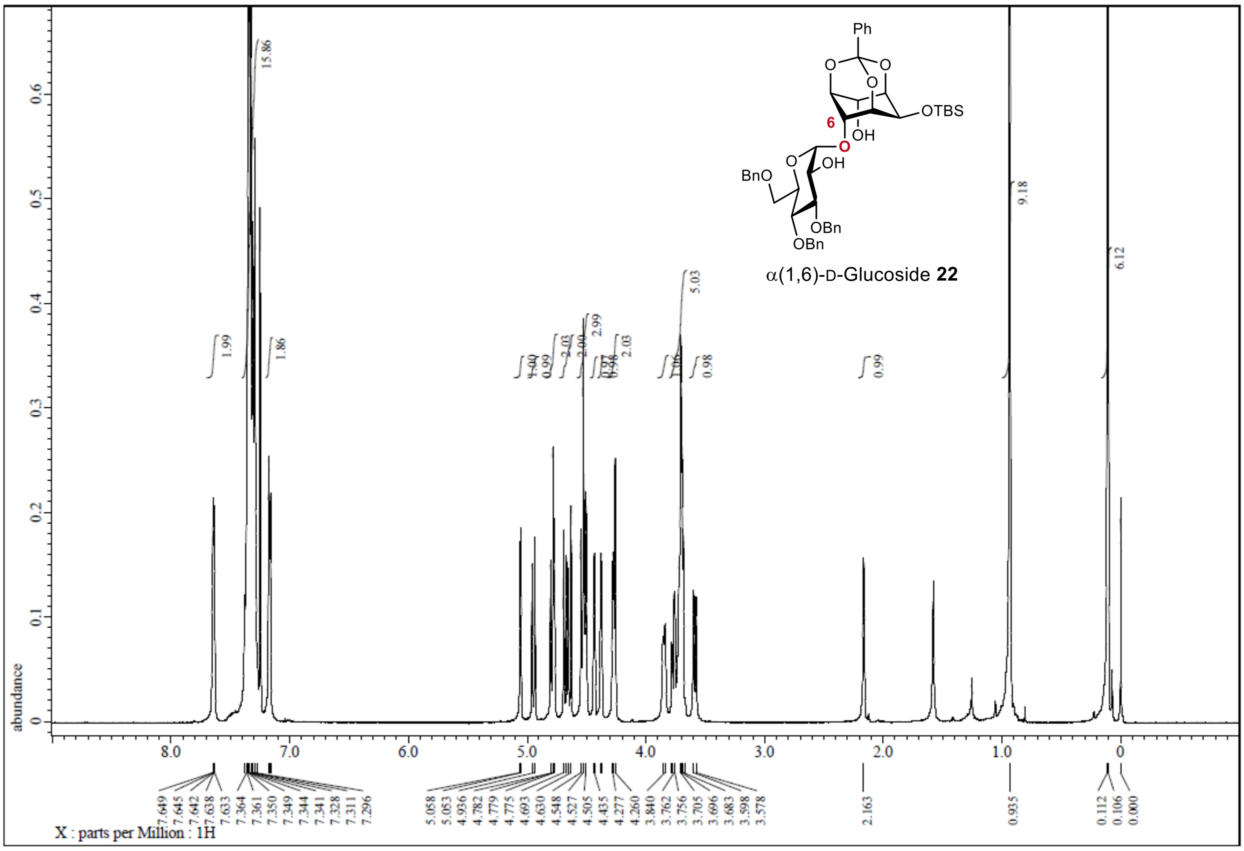

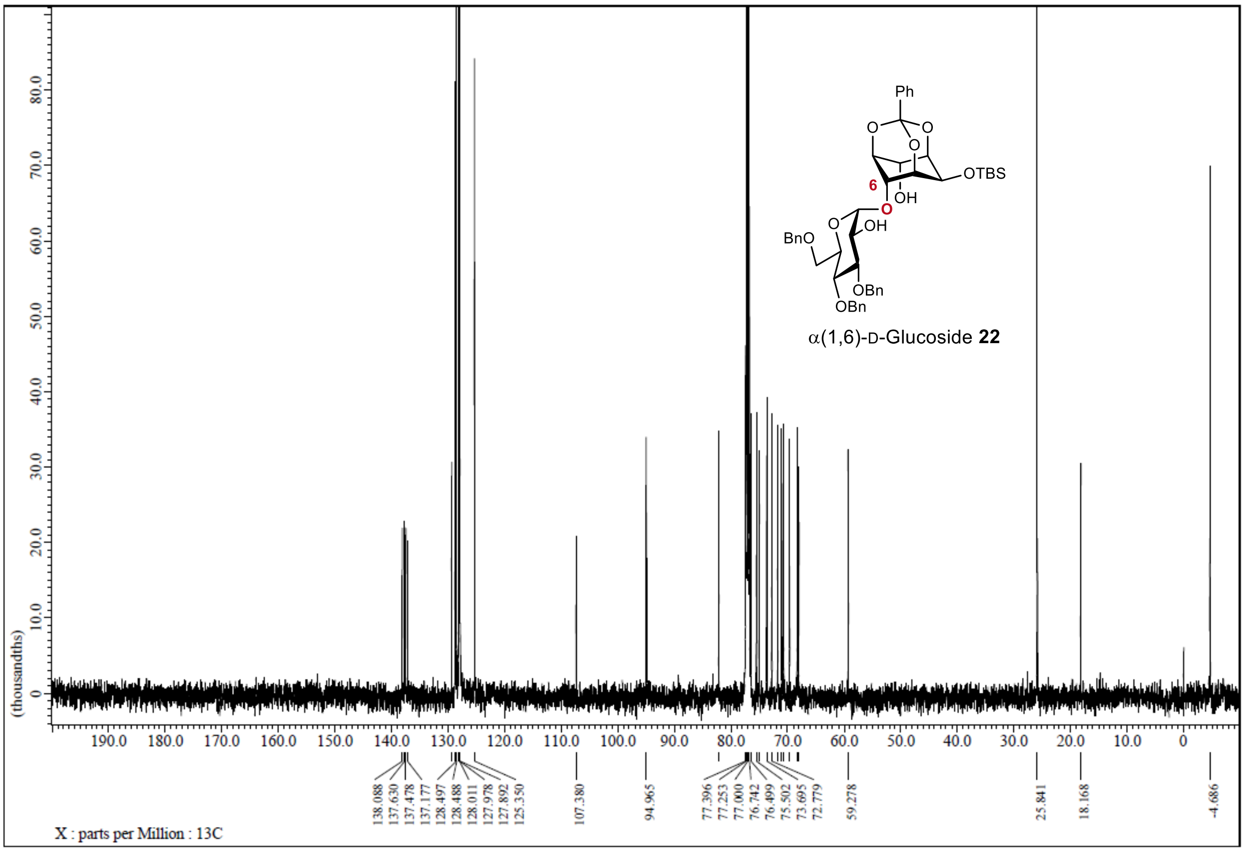

Supplementary Figure 50. ^{1}H and ^{13}C-NMR spectra of compound 22.

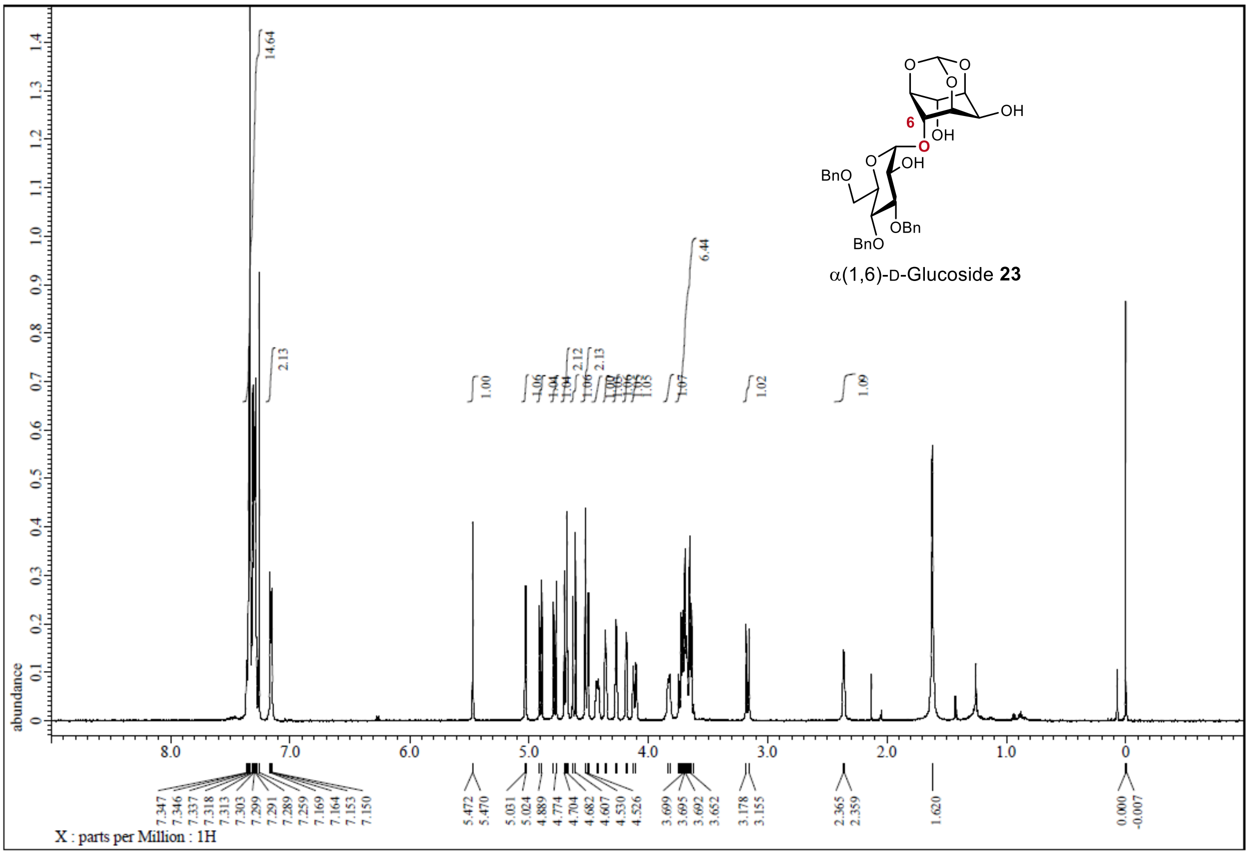

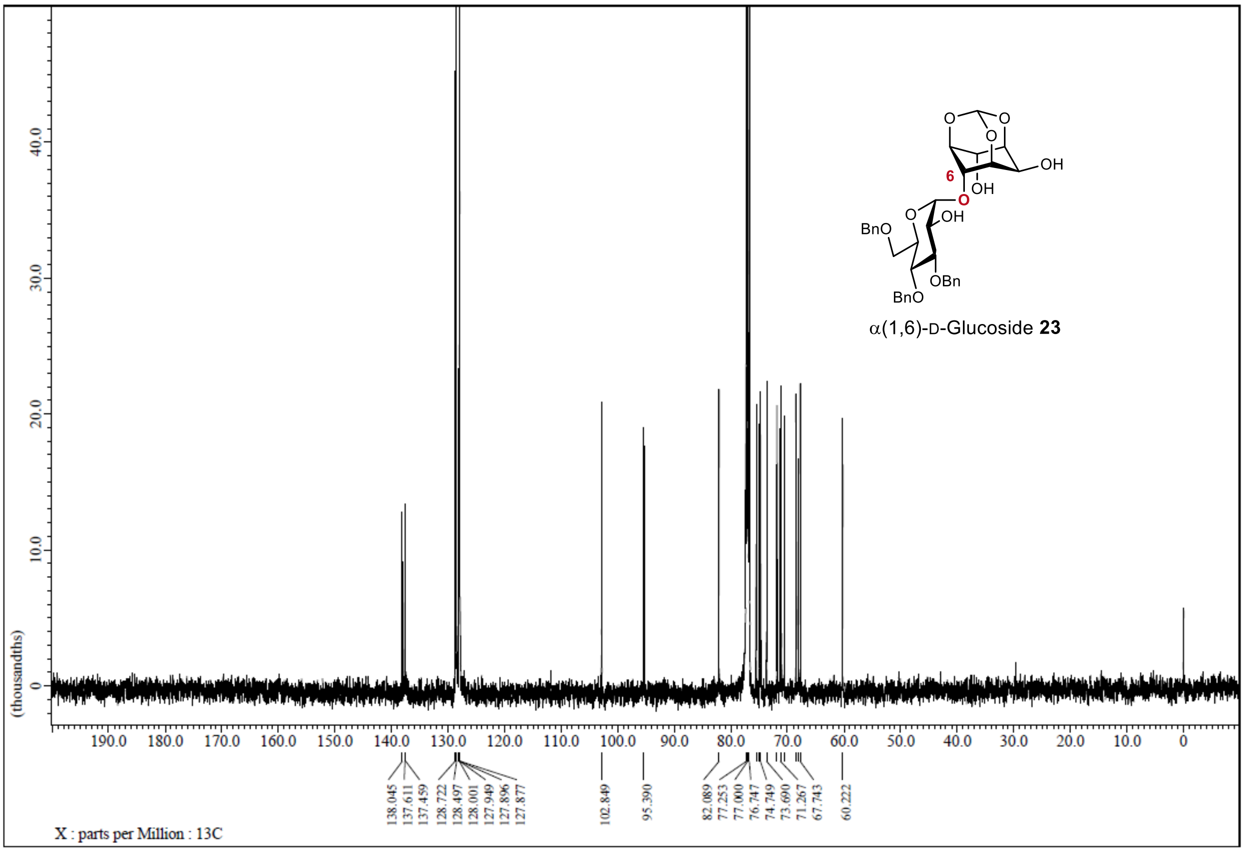

Supplementary Figure 51. ^{1}H and ^{13}C-NMR spectra of compound 23.

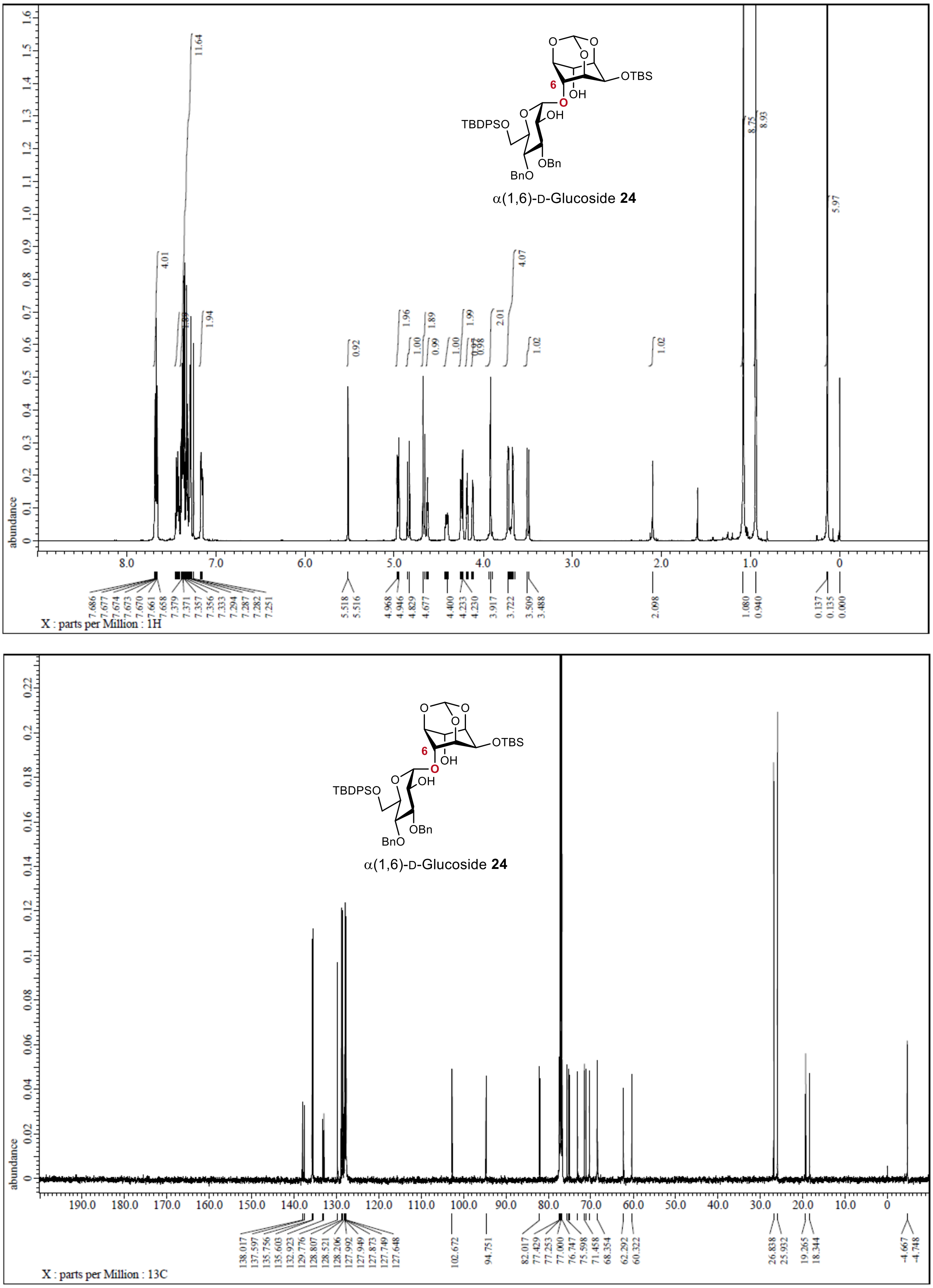

Supplementary Figure 52. ^{1}H and ^{13}C-NMR spectra of compound 24.

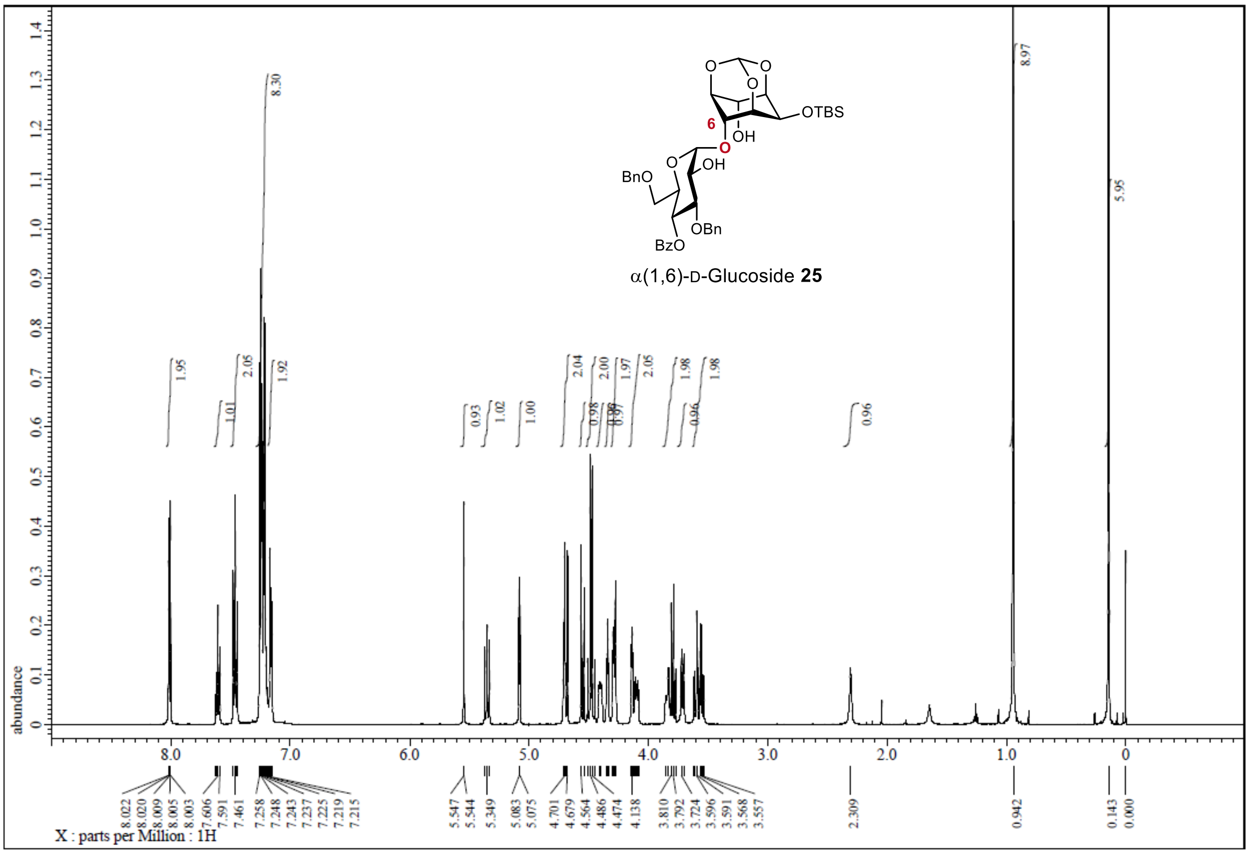

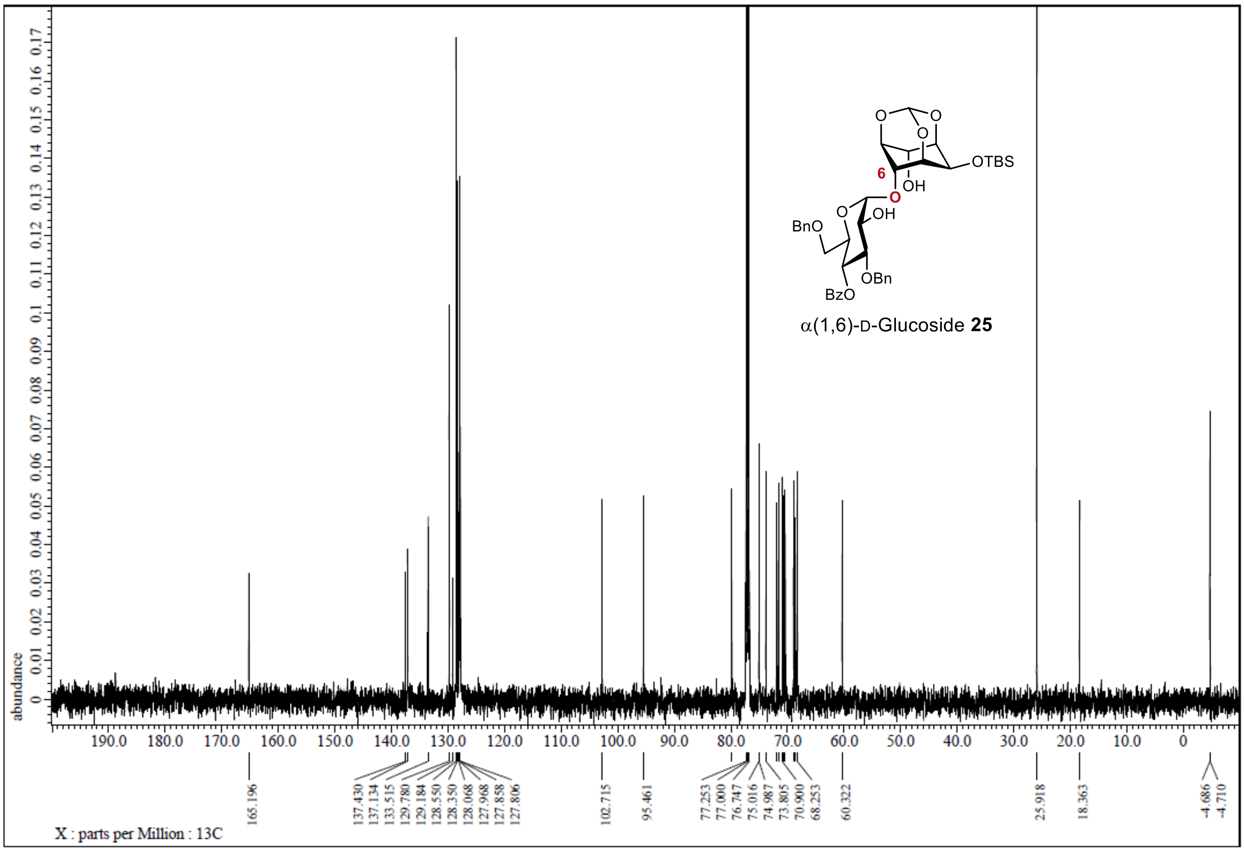

Supplementary Figure 53. ^{1}H and ^{13}C-NMR spectra of compound 25.

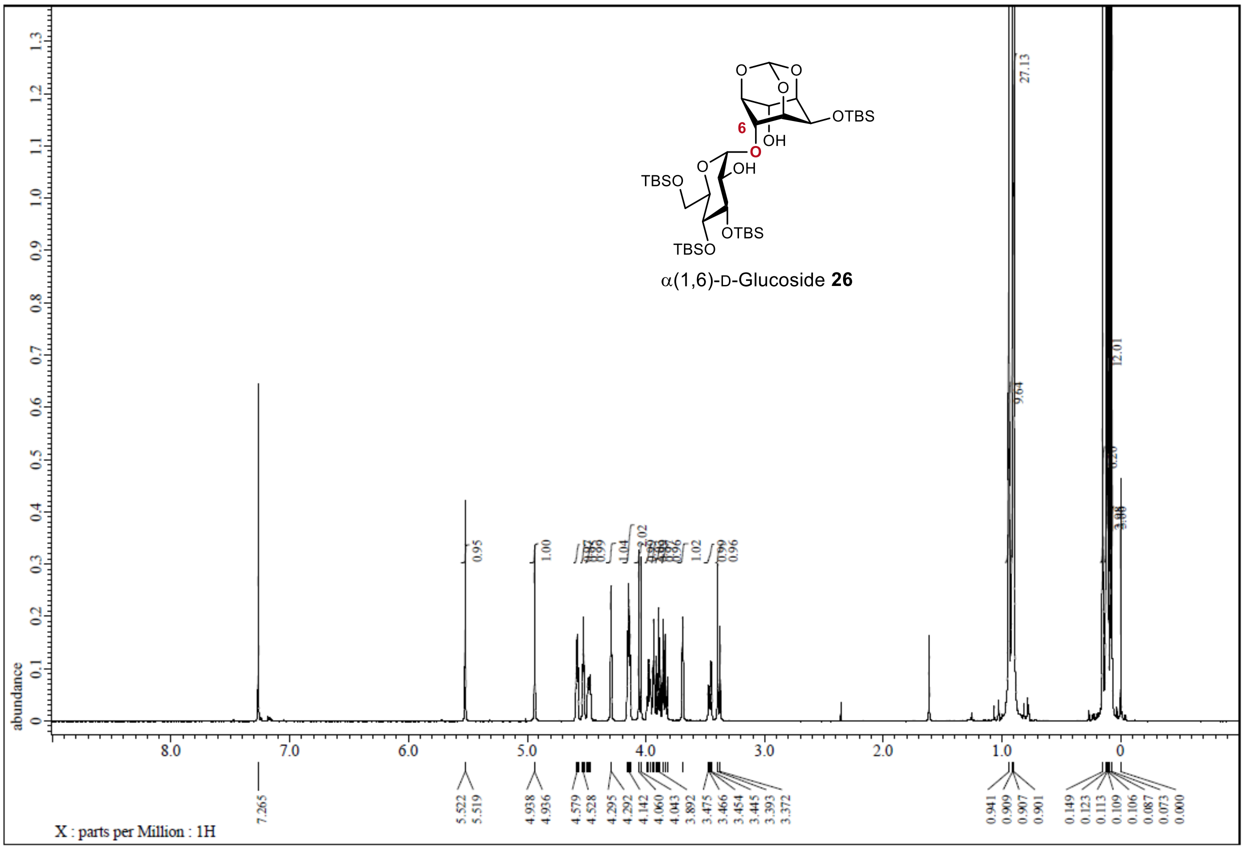

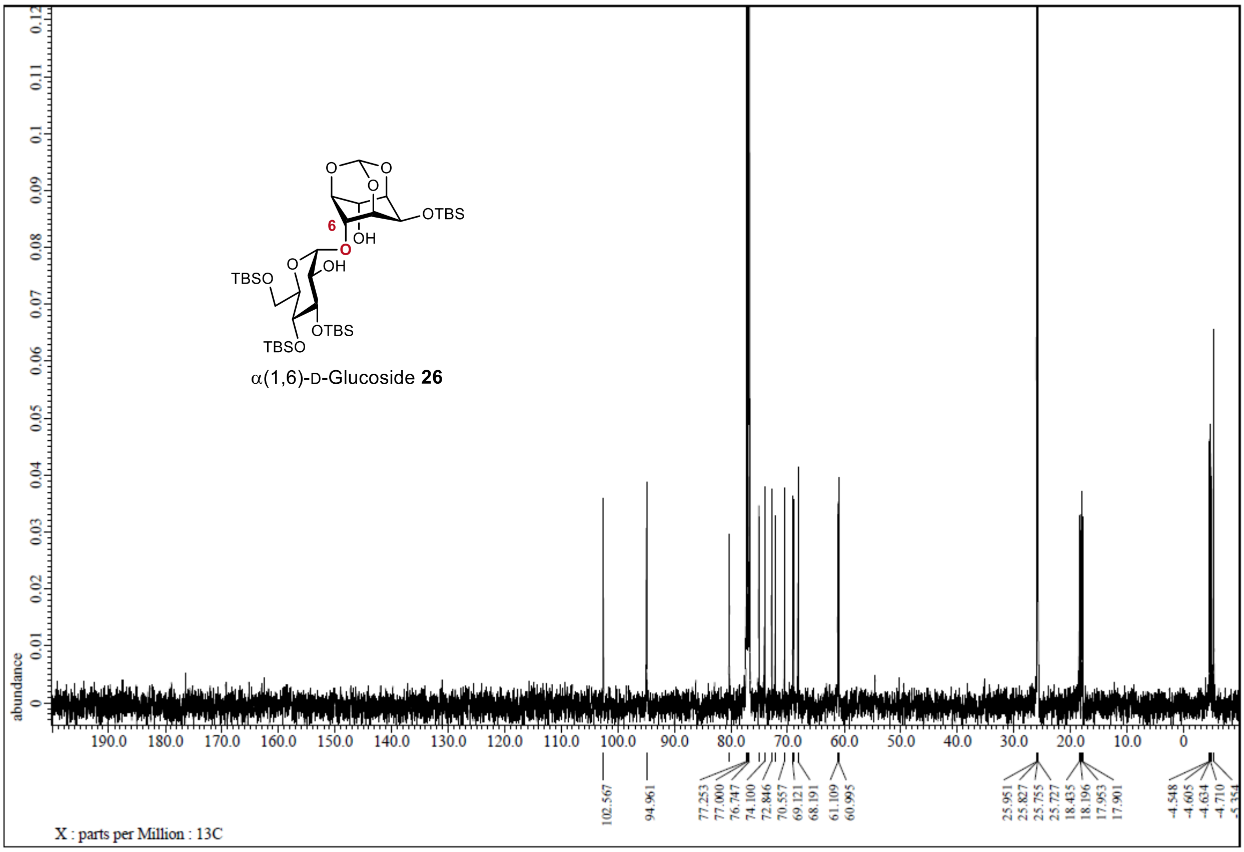

Supplementary Figure 54. ^{1}H and ^{13}C-NMR spectra of compound 26.

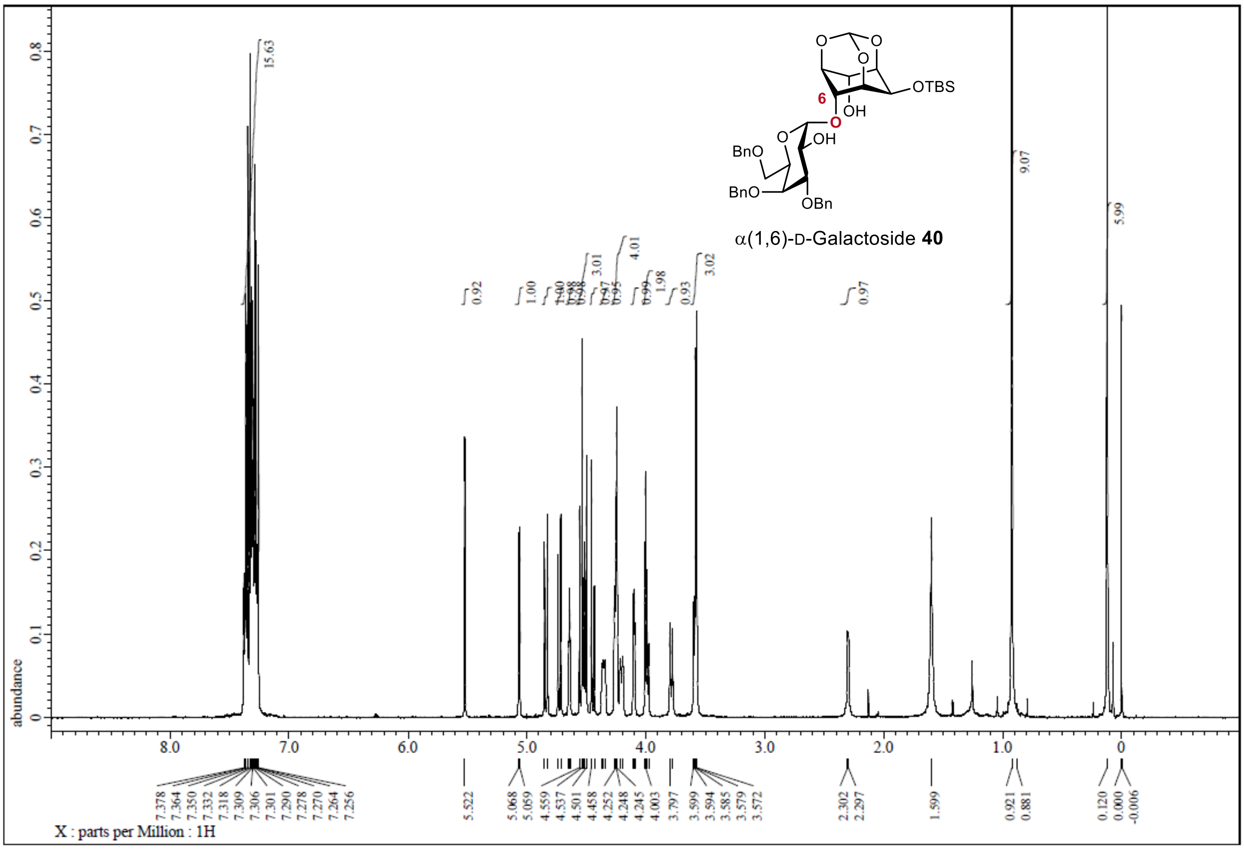

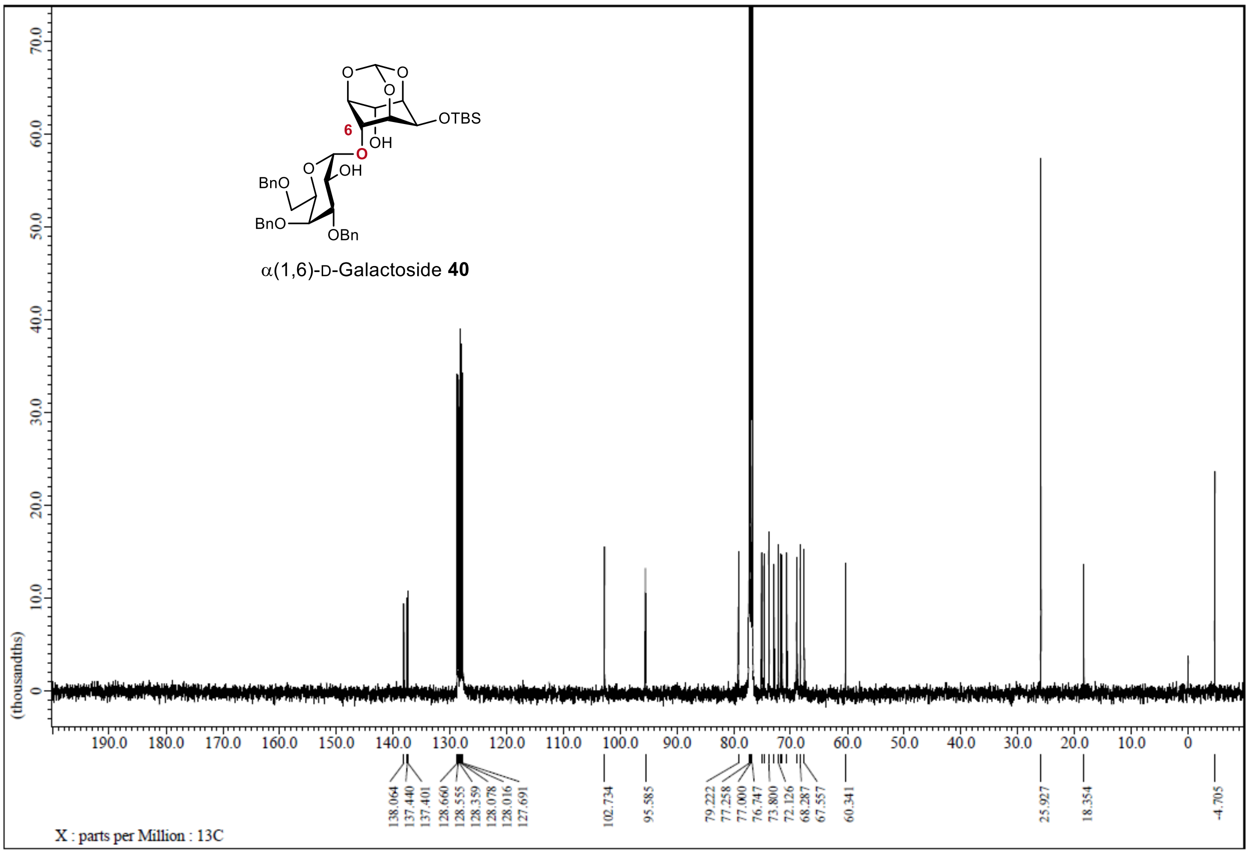

Supplementary Figure 55. ^{1}H and ^{13}C-NMR spectra of compound 40.

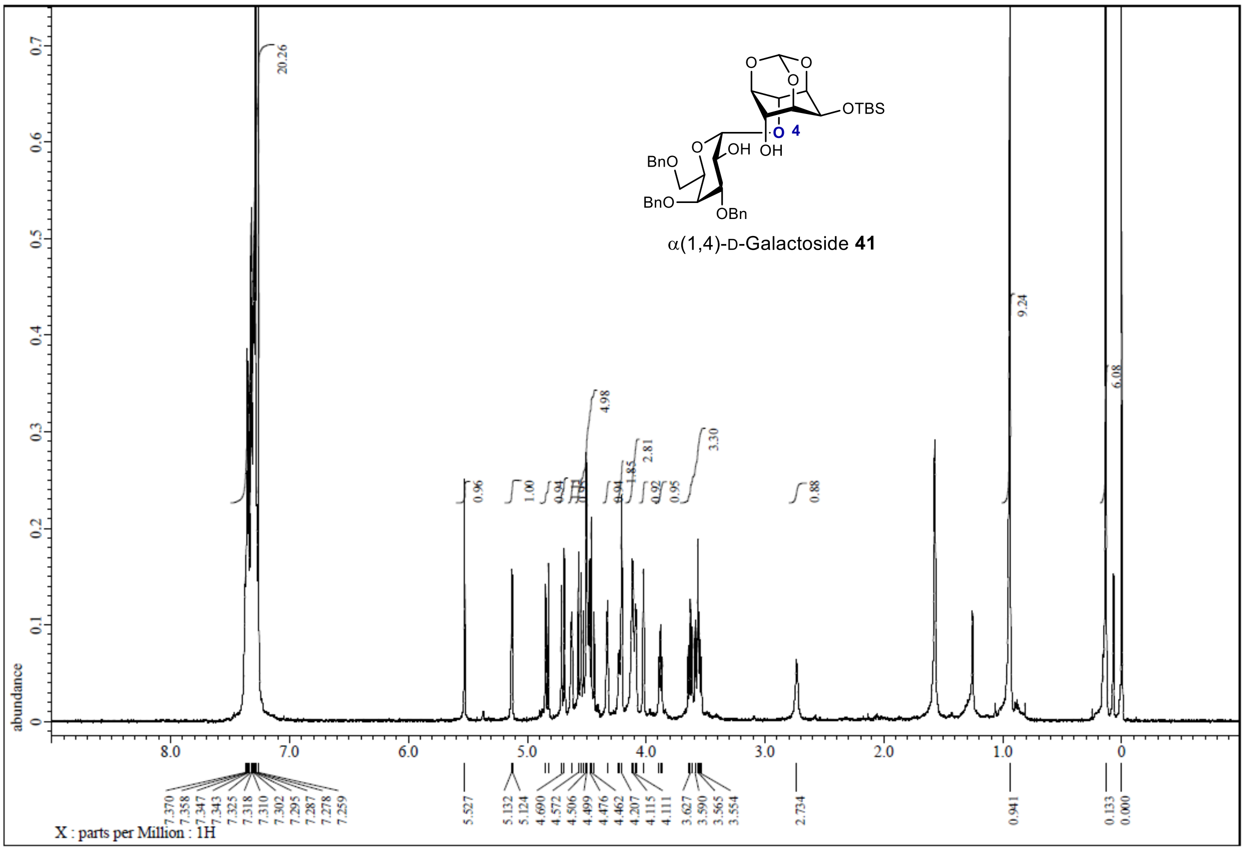

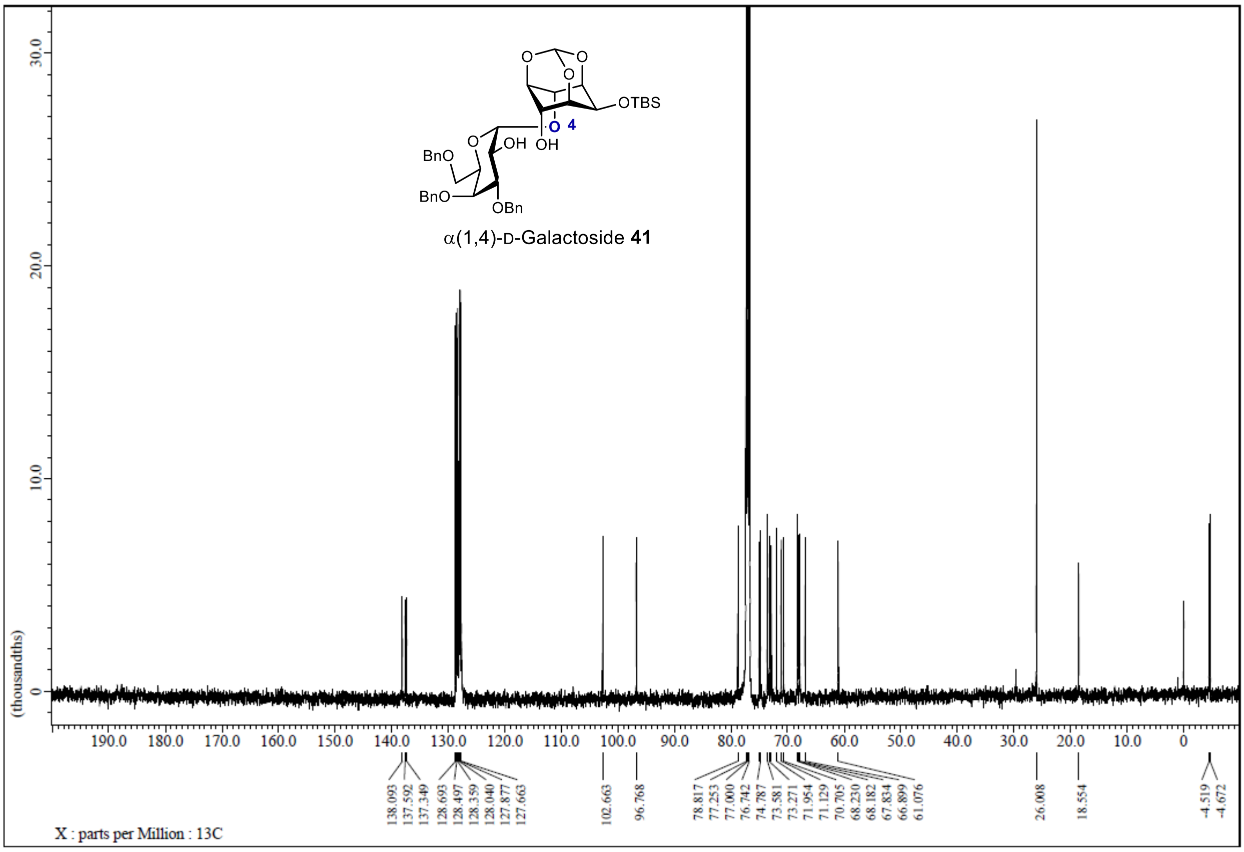

Supplementary Figure 56. ^{1}H and ^{13}C-NMR spectra of compound 41.

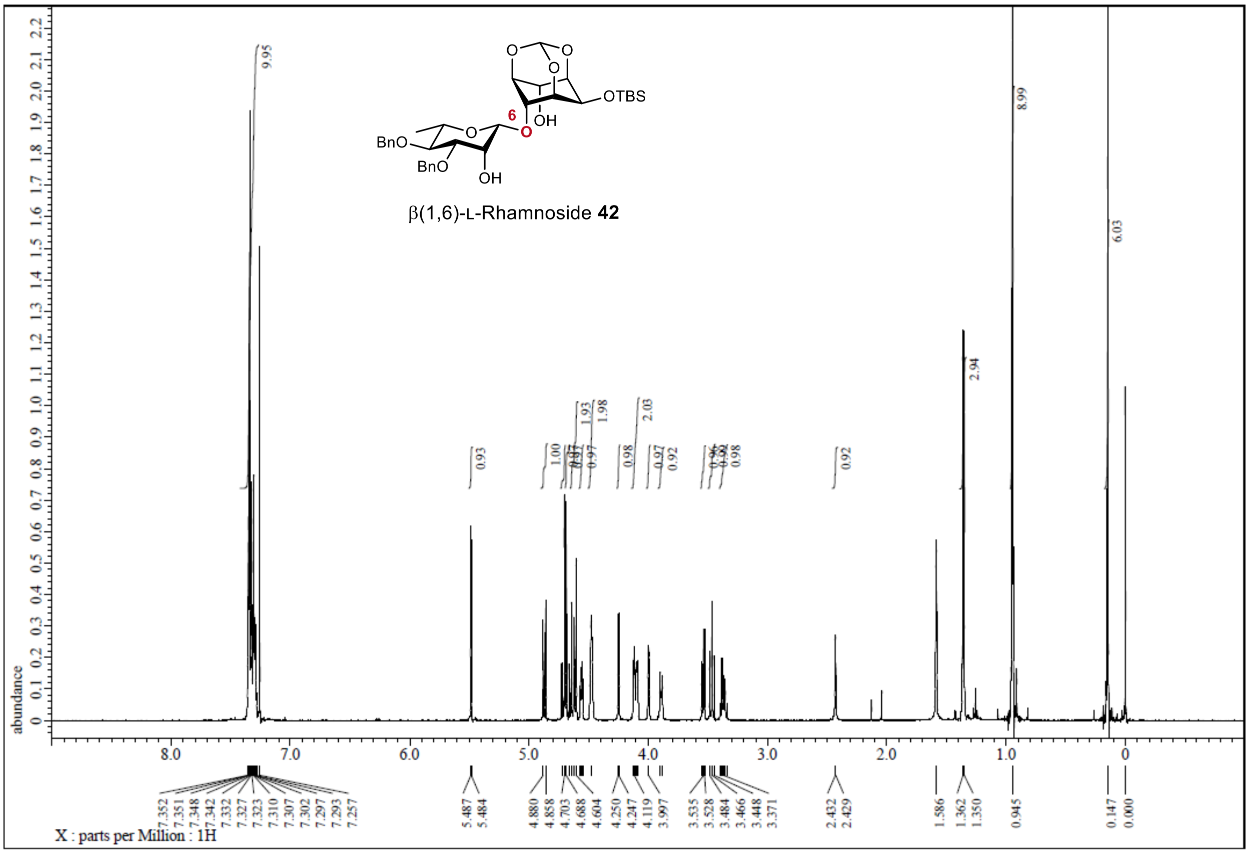

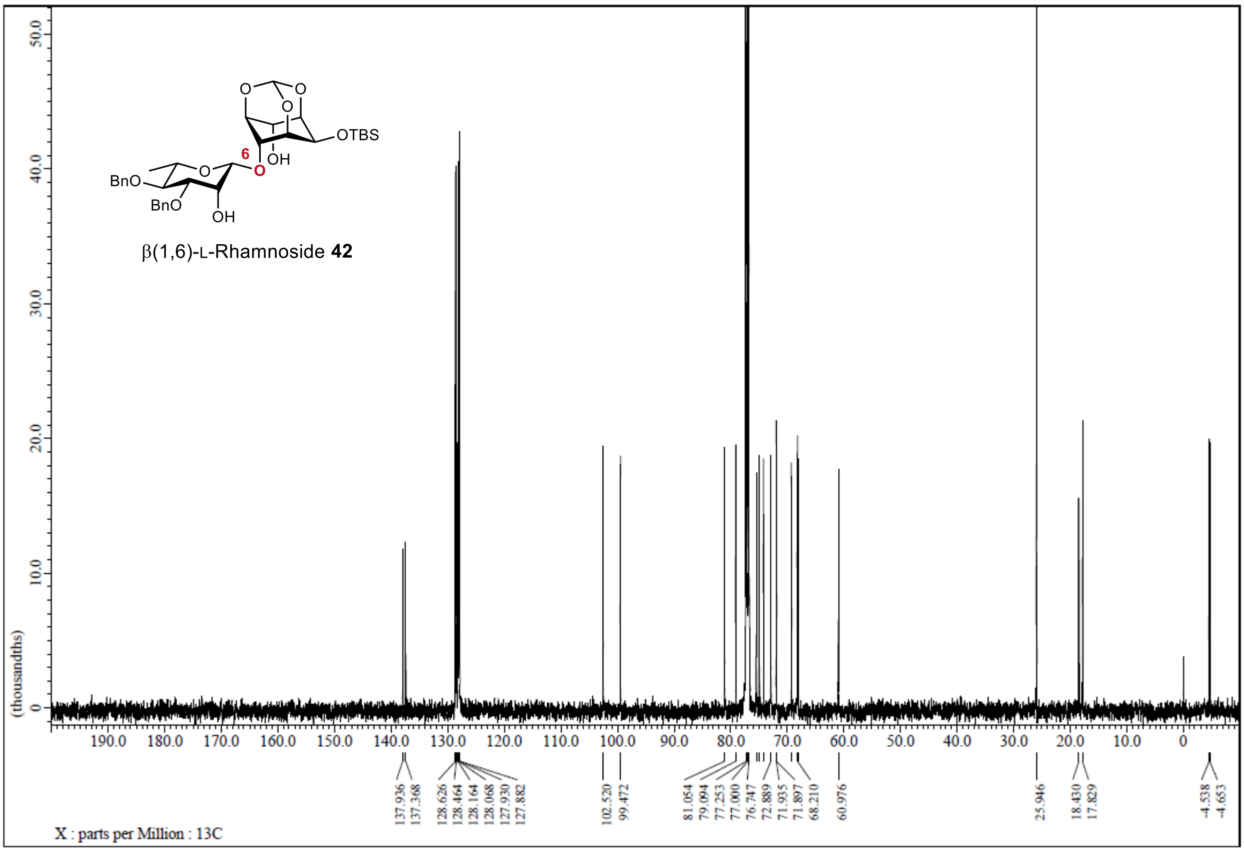

Supplementary Figure 57. ^{1}H and ^{13}C-NMR spectra of compound 42.

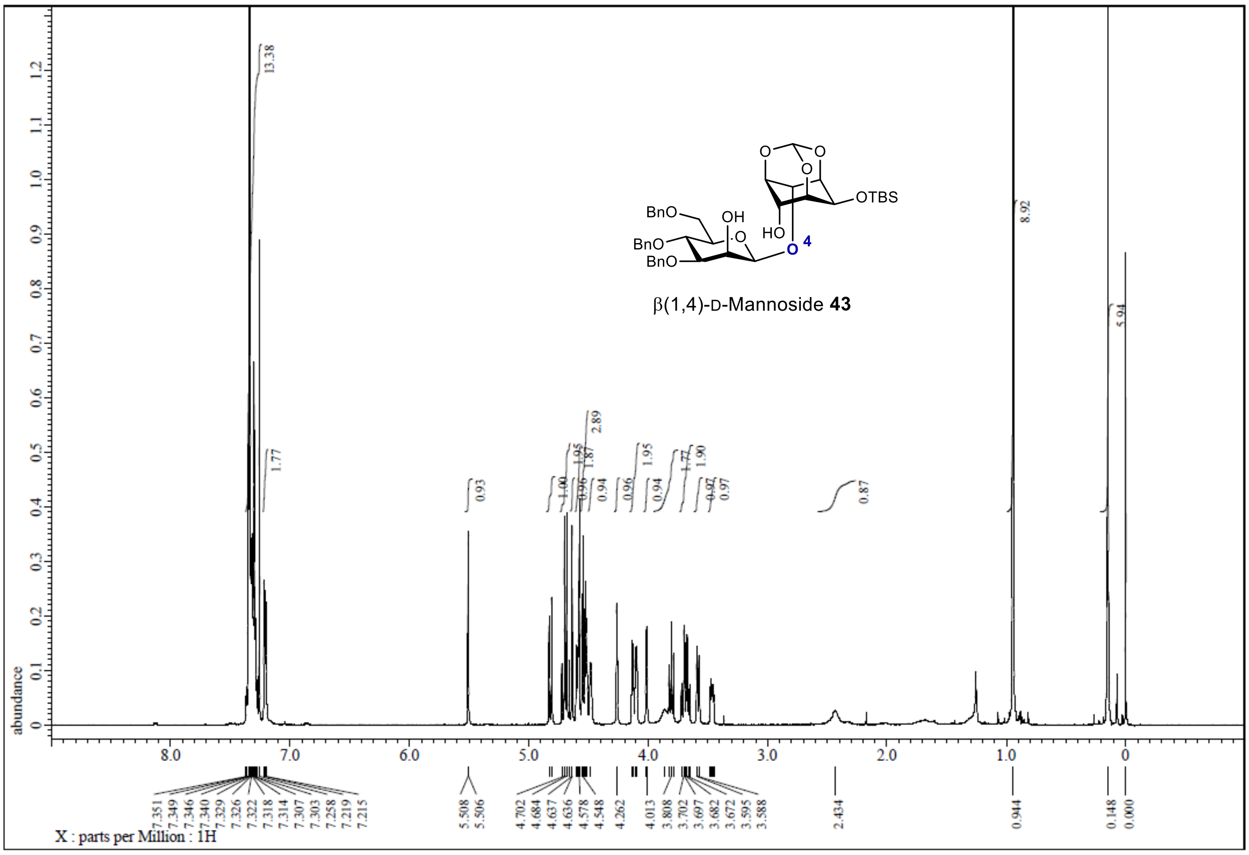

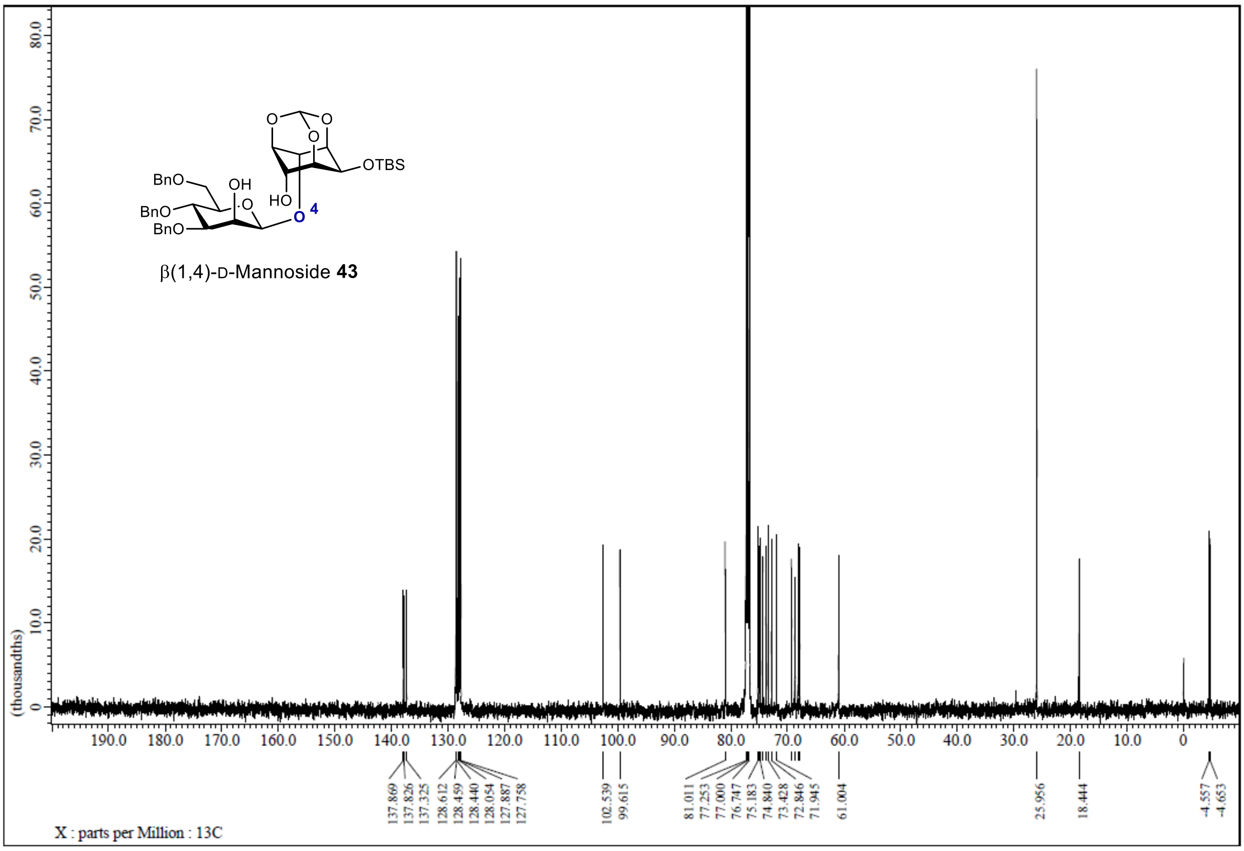

Supplementary Figure 58. ^{1}H and ^{13}C-NMR spectra of compound 43.

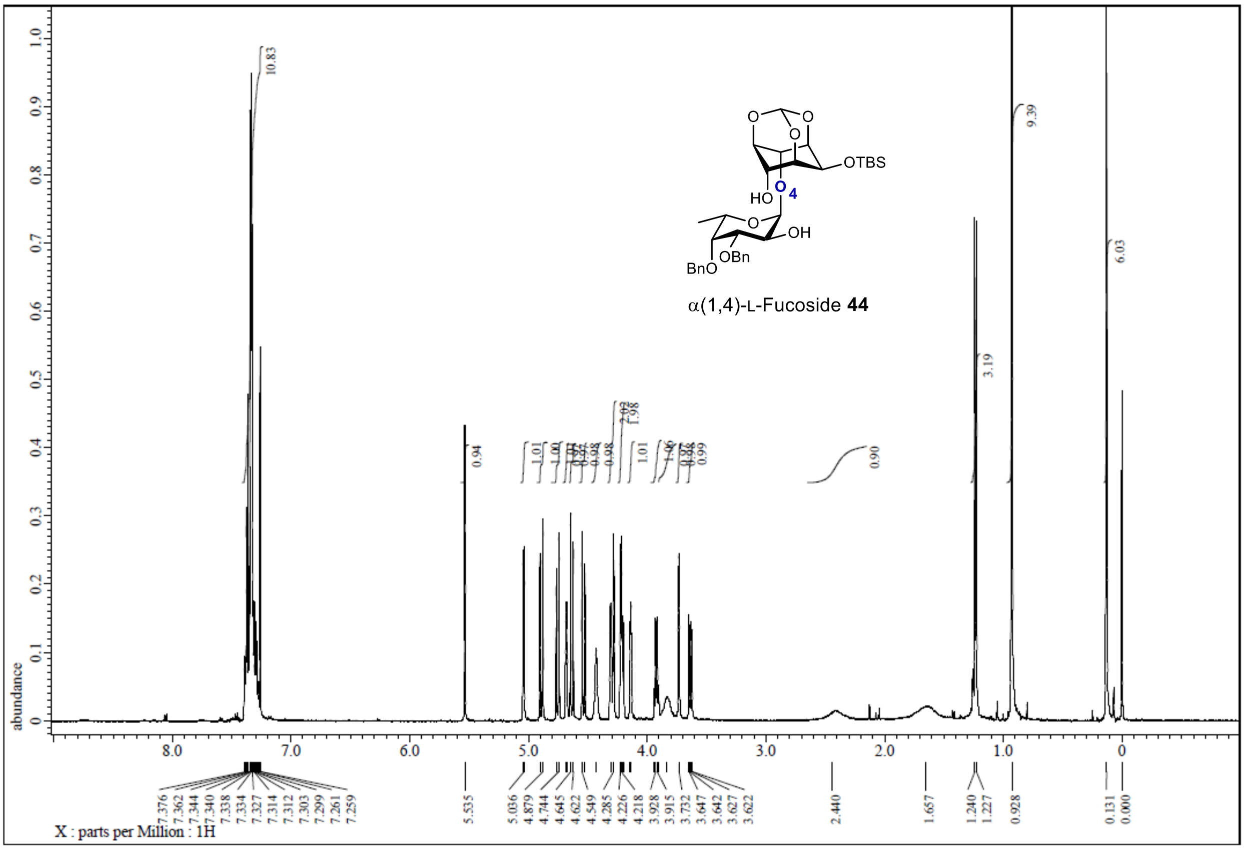

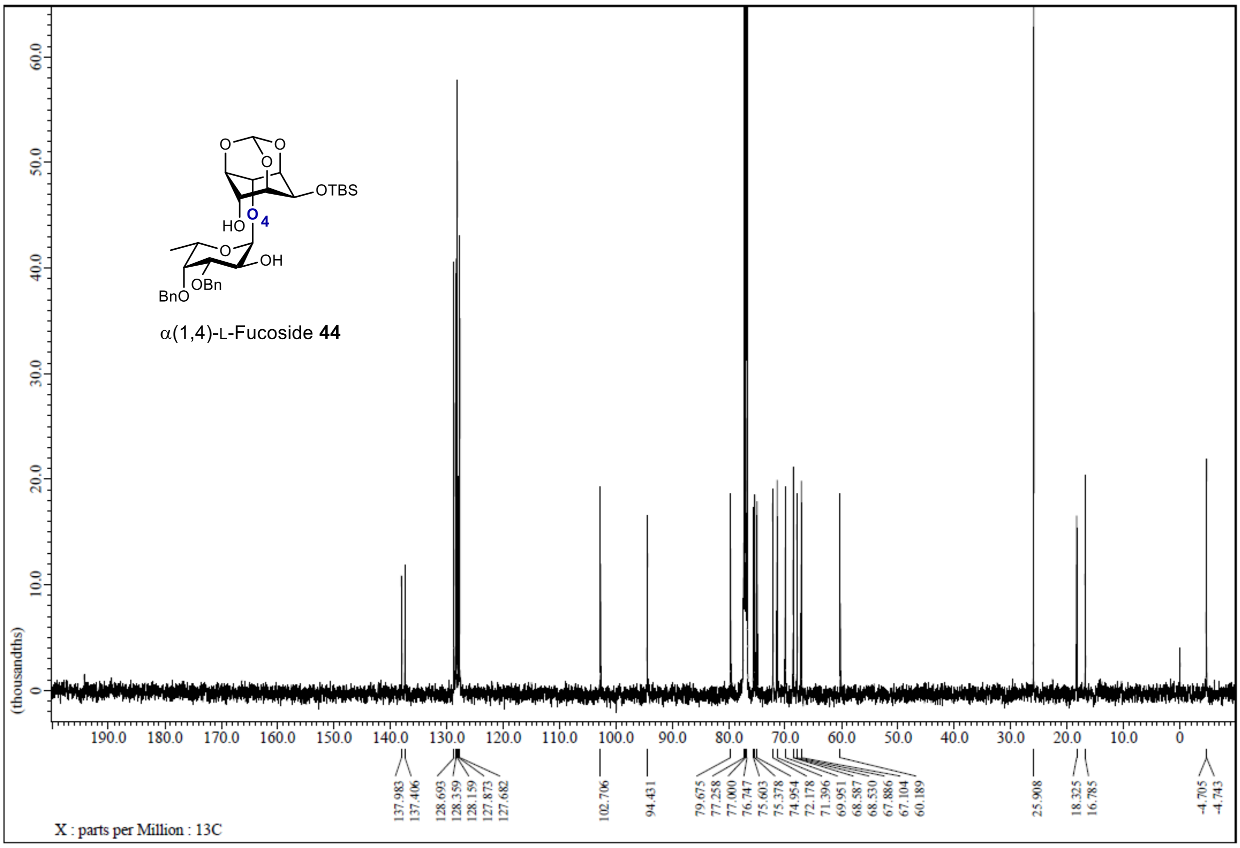

Supplementary Figure 59. ^{1}H and ^{13}C-NMR spectra of compound 44.

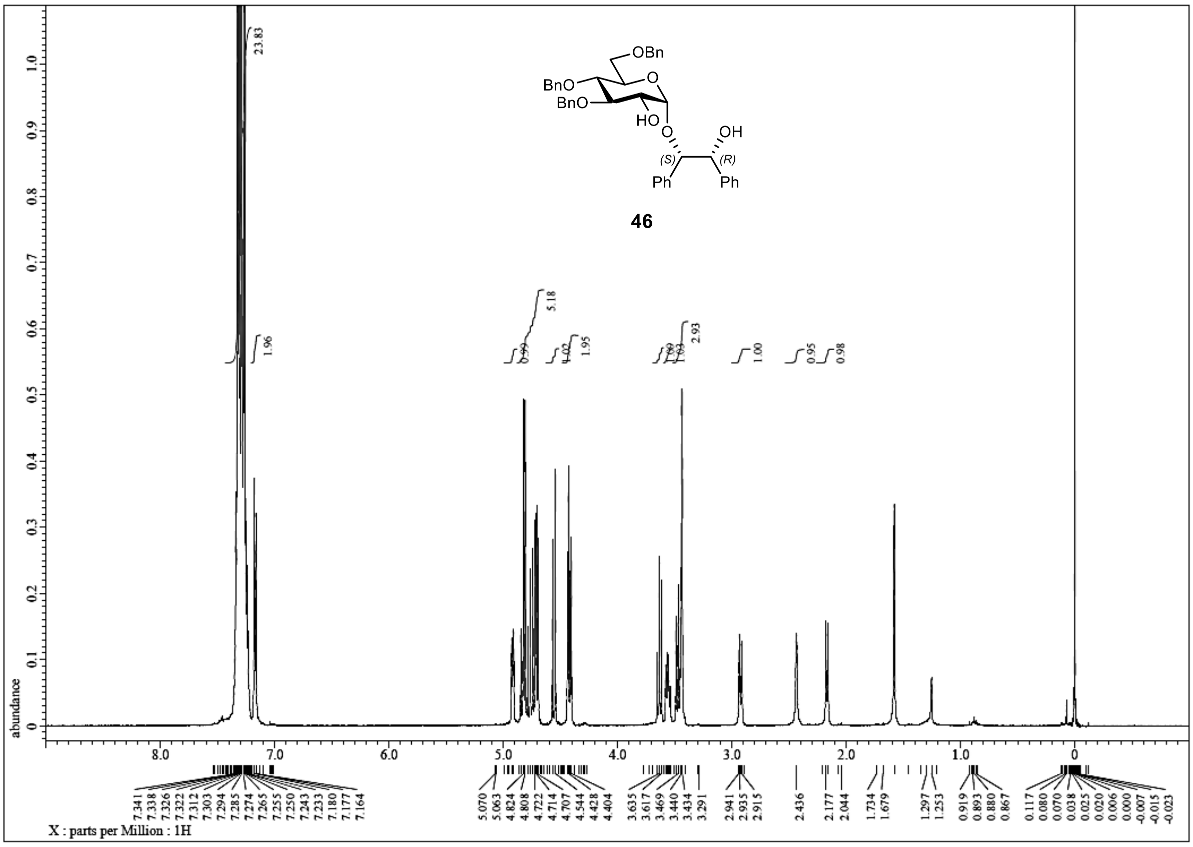

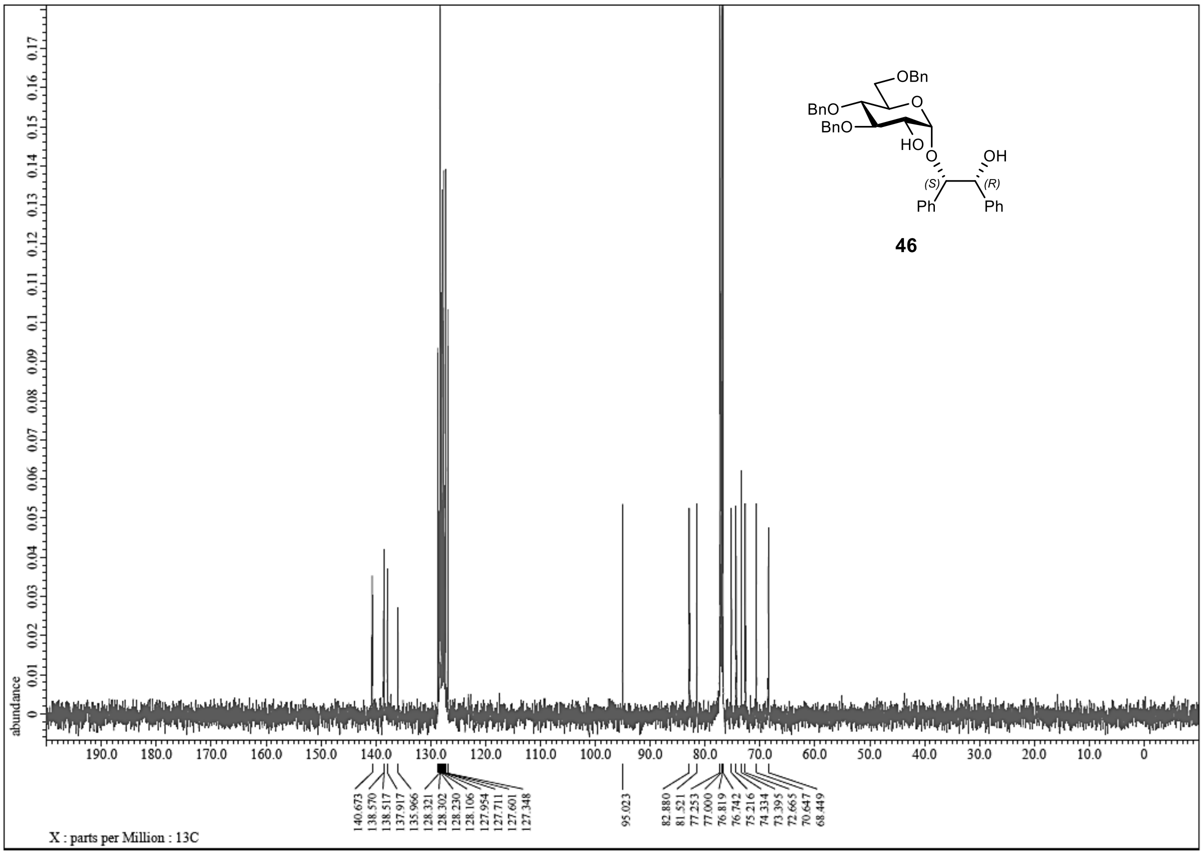

Supplementary Figure 60. ^{1}H and ^{13}C-NMR spectra of compound 46.

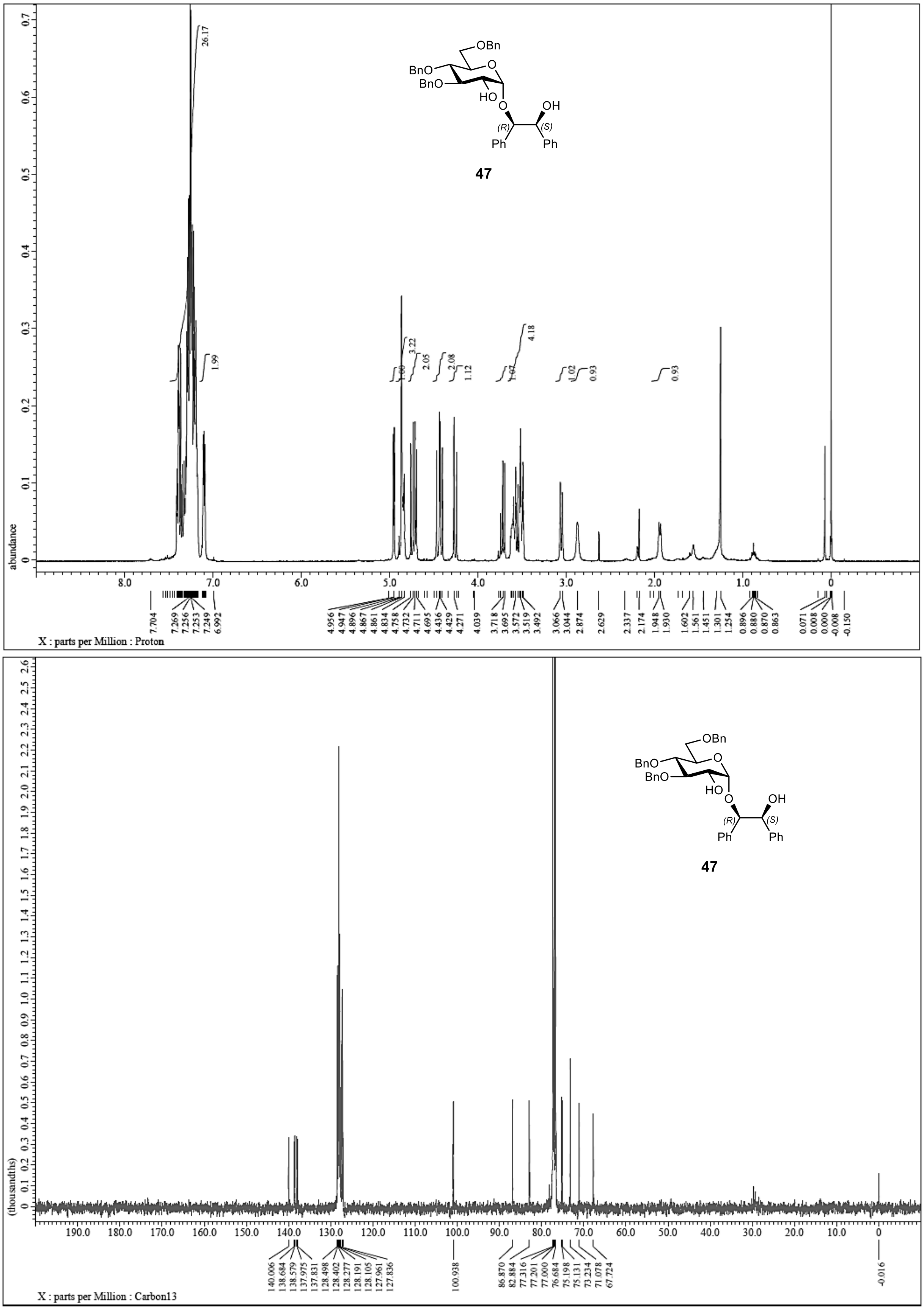

Supplementary Figure 61. ^{1}H and ^{13}C-NMR spectra of compound 47.

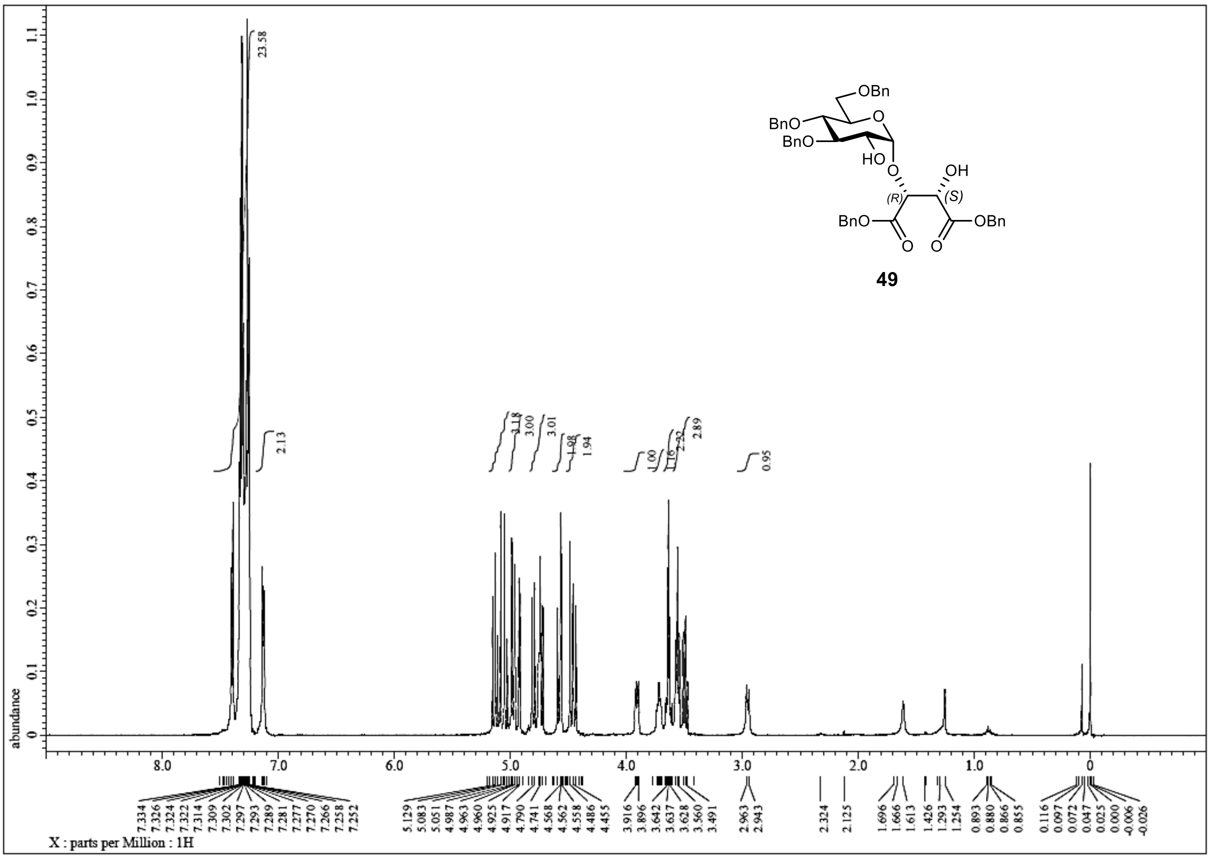

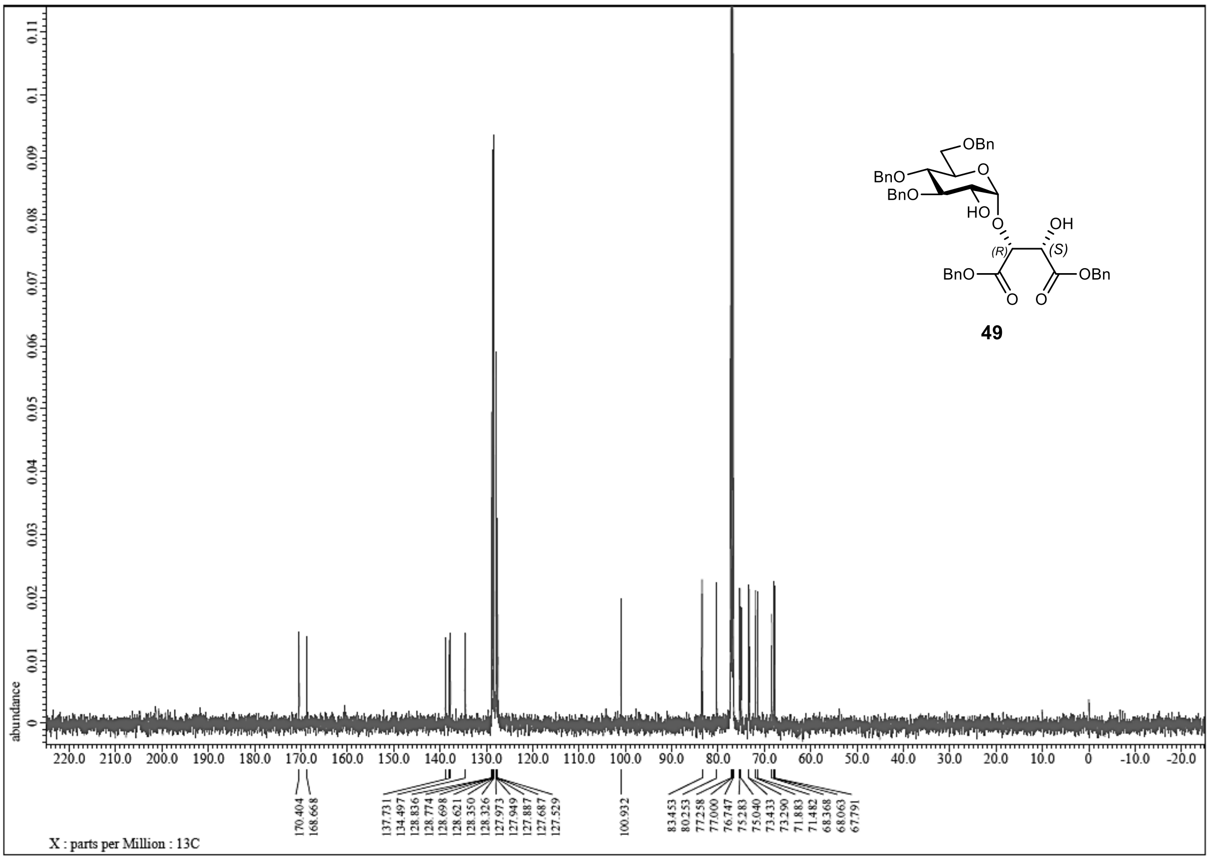

Supplementary Figure 62. ^{1}H and ^{13}C-NMR spectra of compound 49.

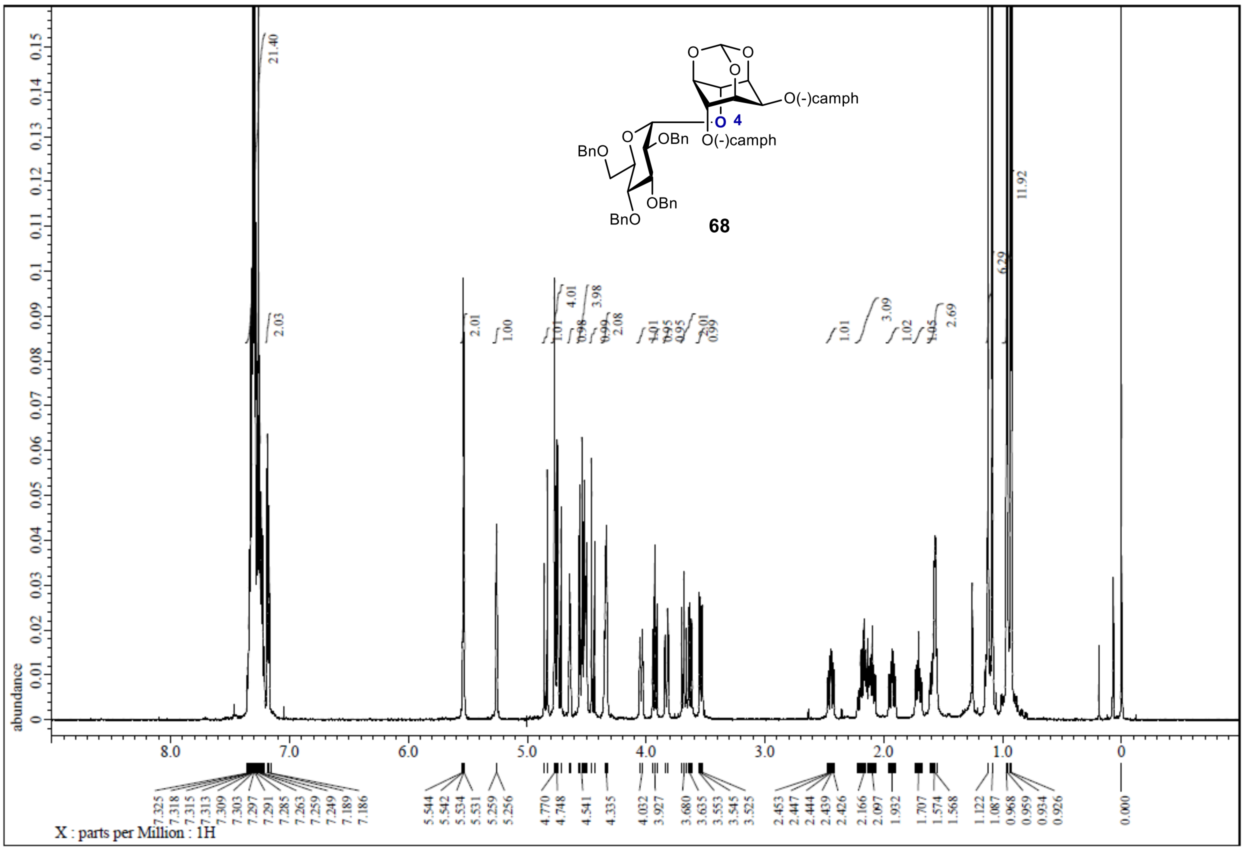

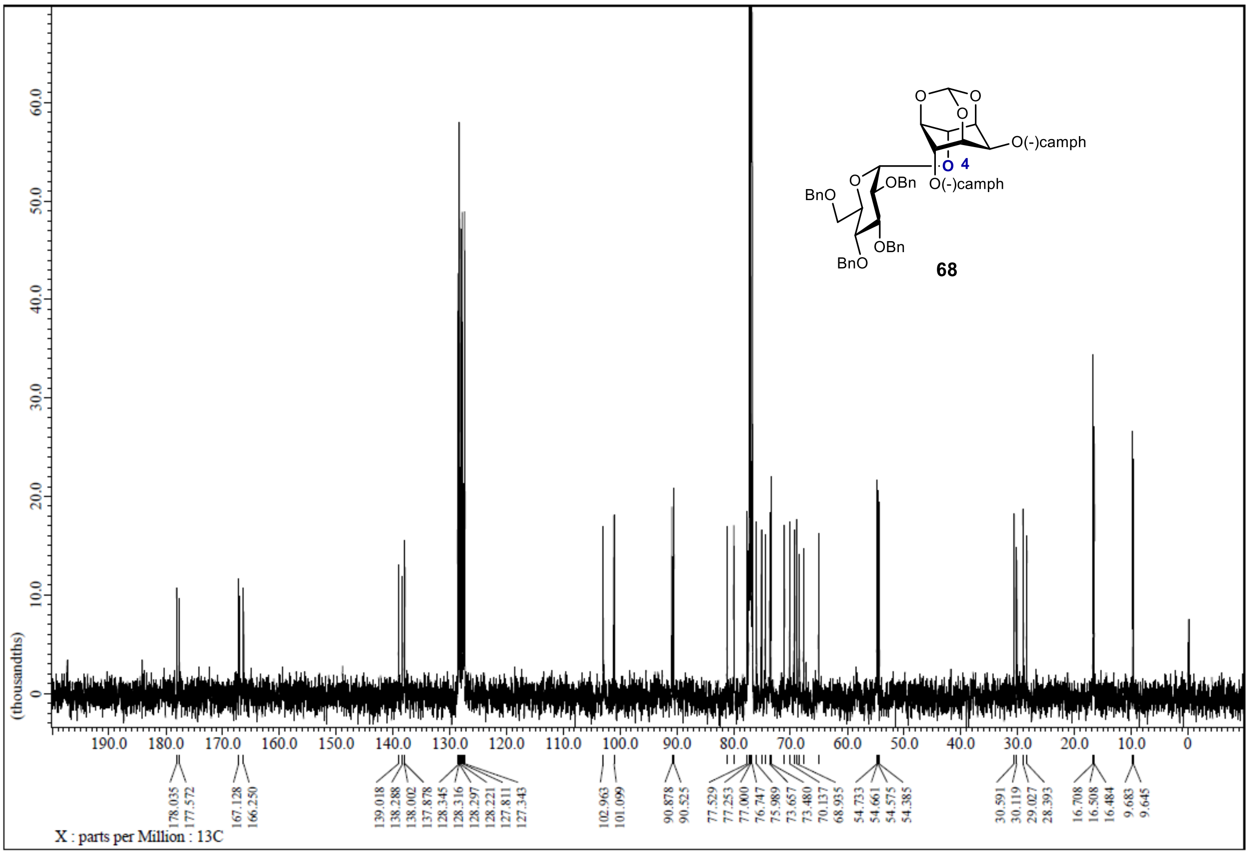

Supplementary Figure 63. ¹H and ¹³C-NMR spectra of compound 68.

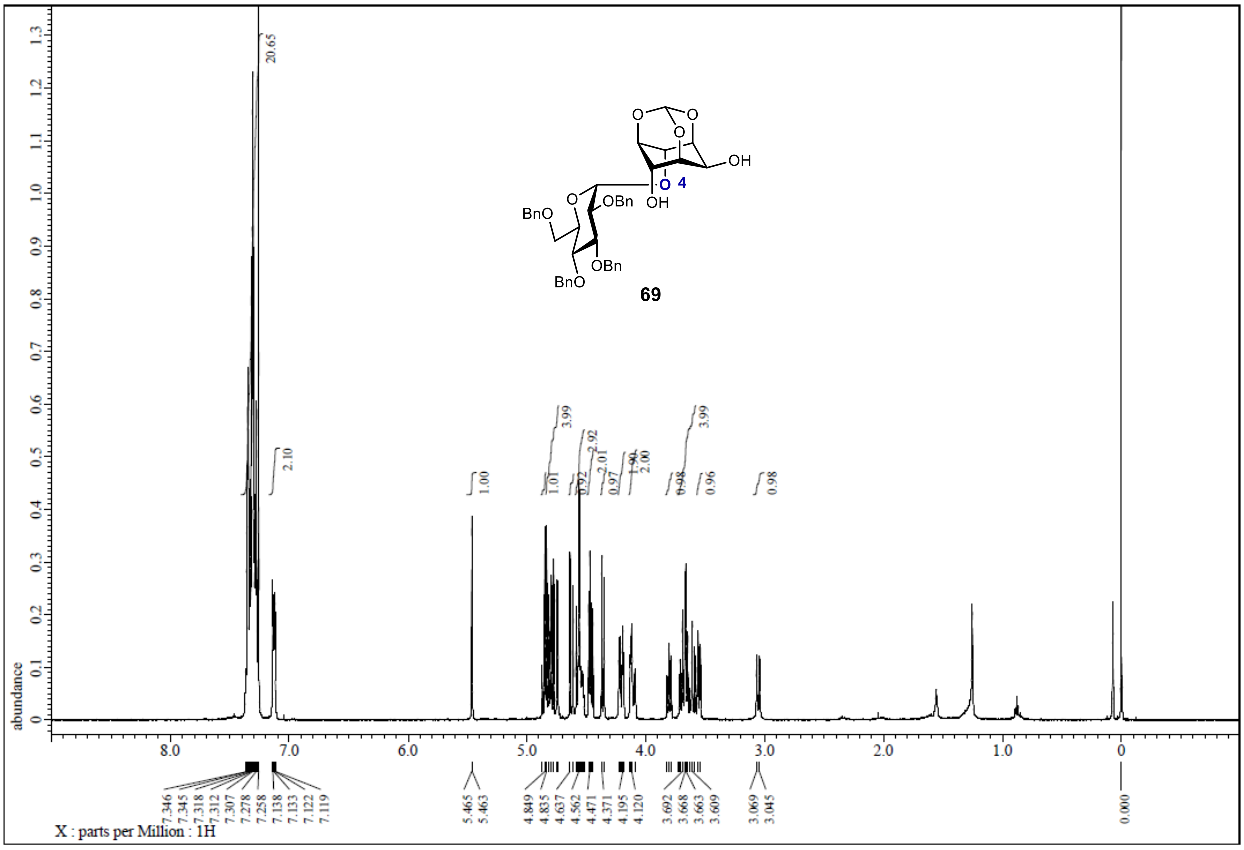

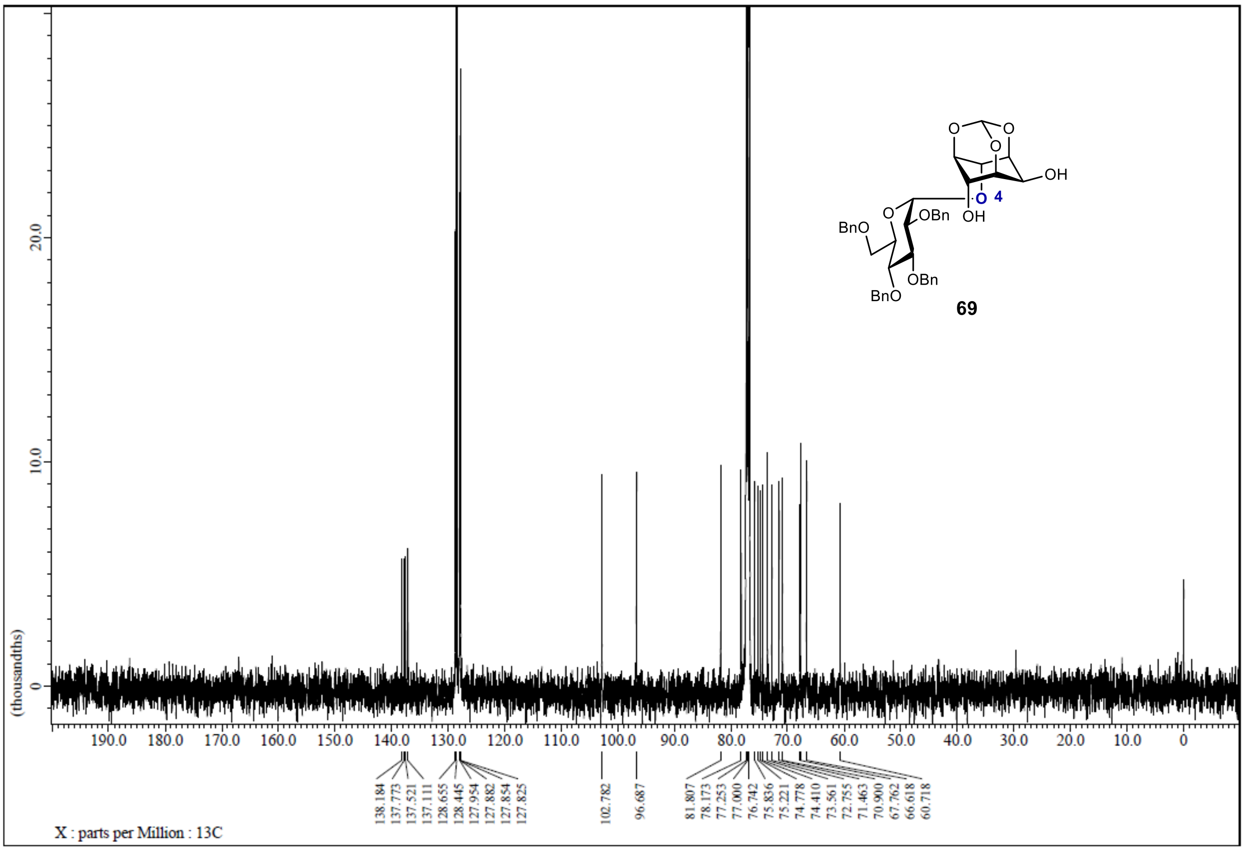

Supplementary Figure 64. ^{1}H and ^{13}C-NMR spectra of compound 69.

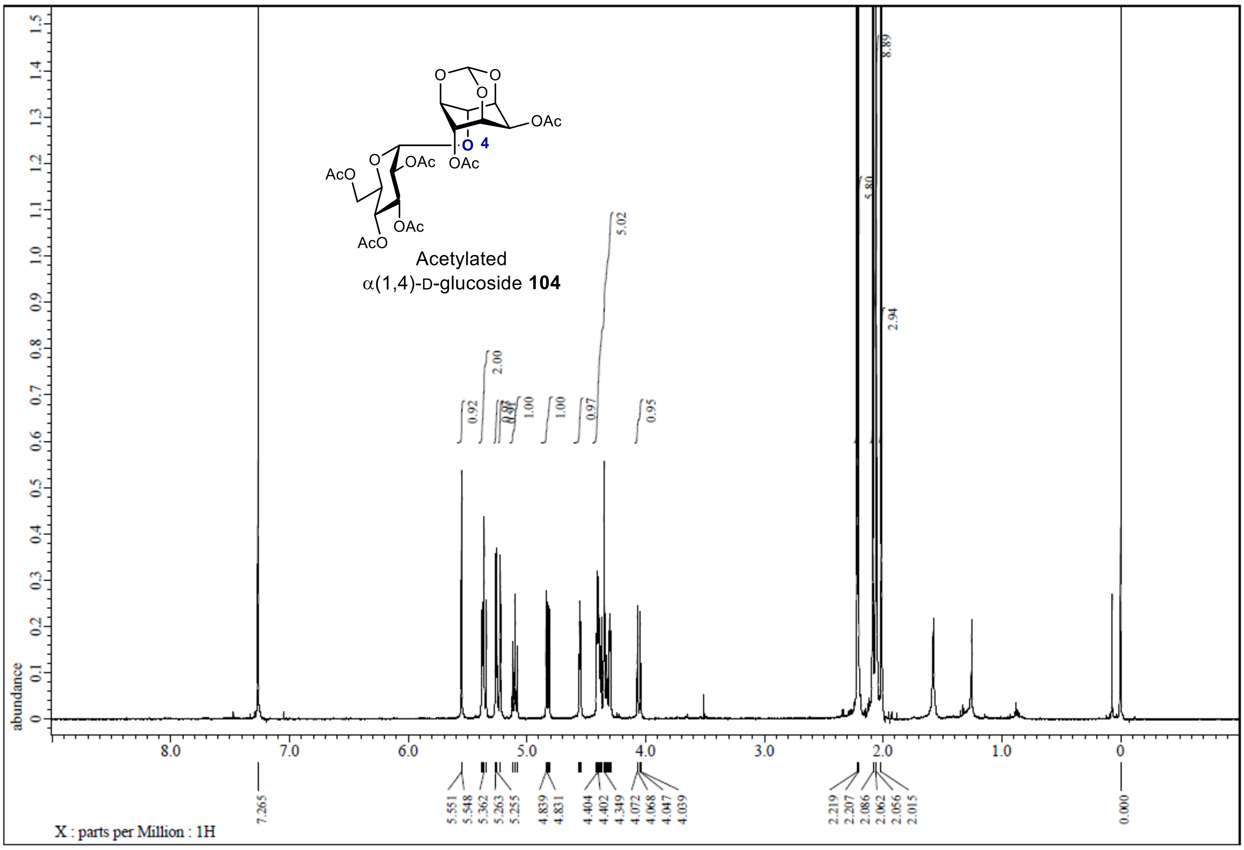

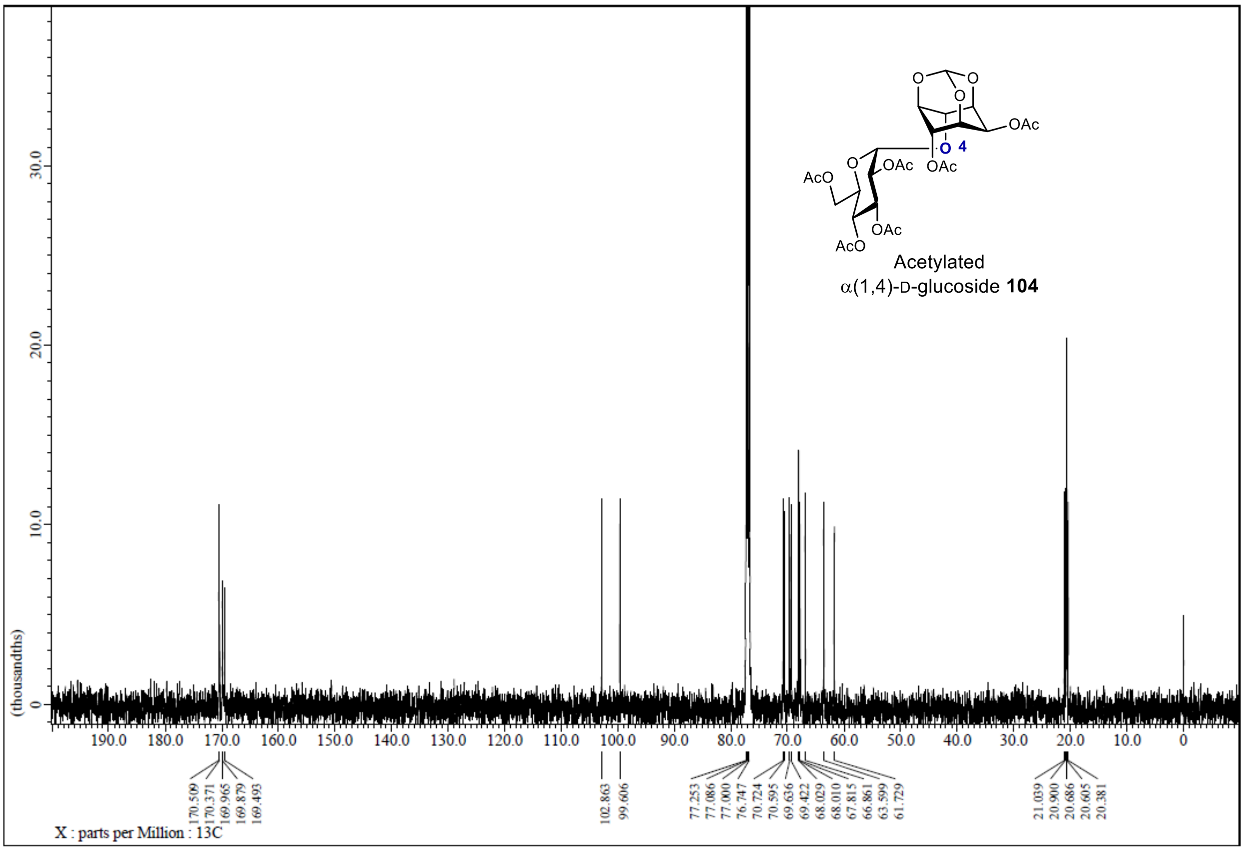

Supplementary Figure 65. ^{1}H and ^{13}C-NMR spectra of compound 104.

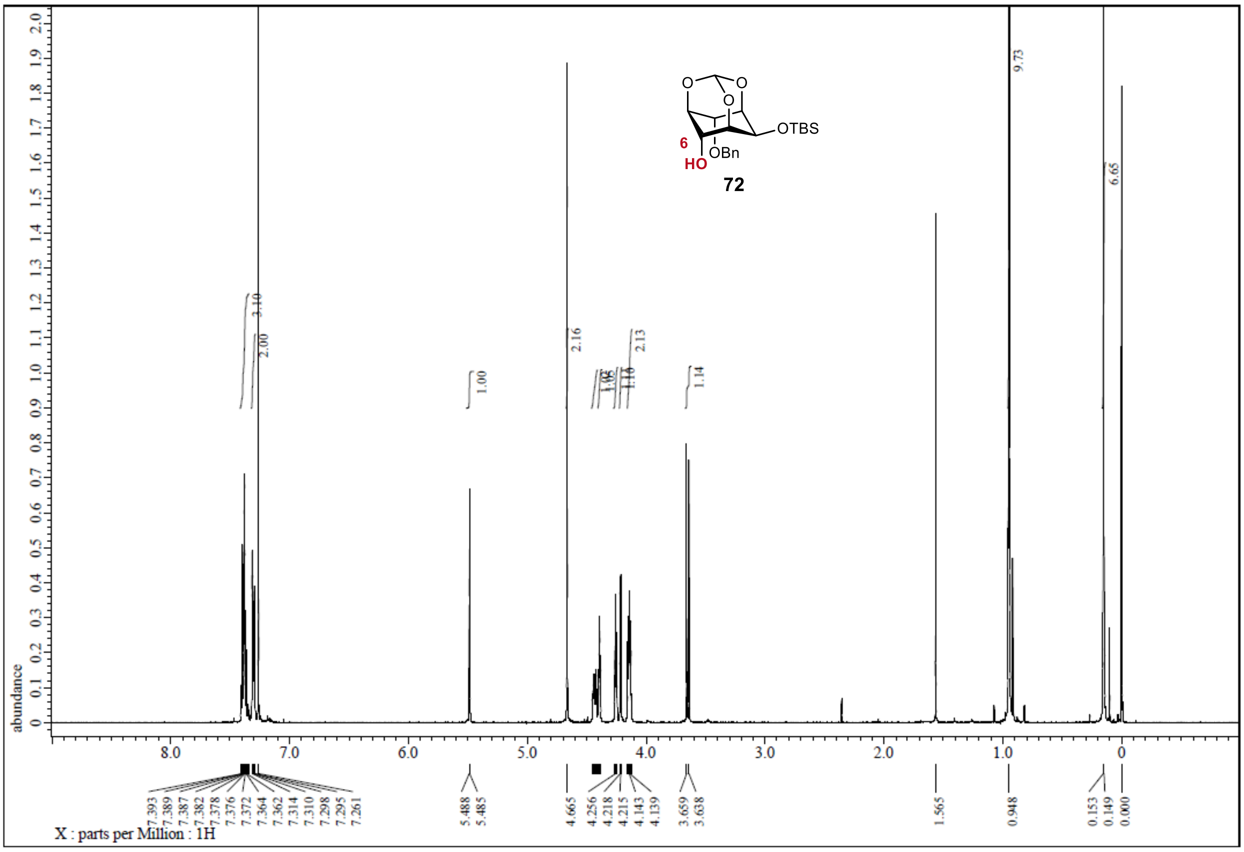

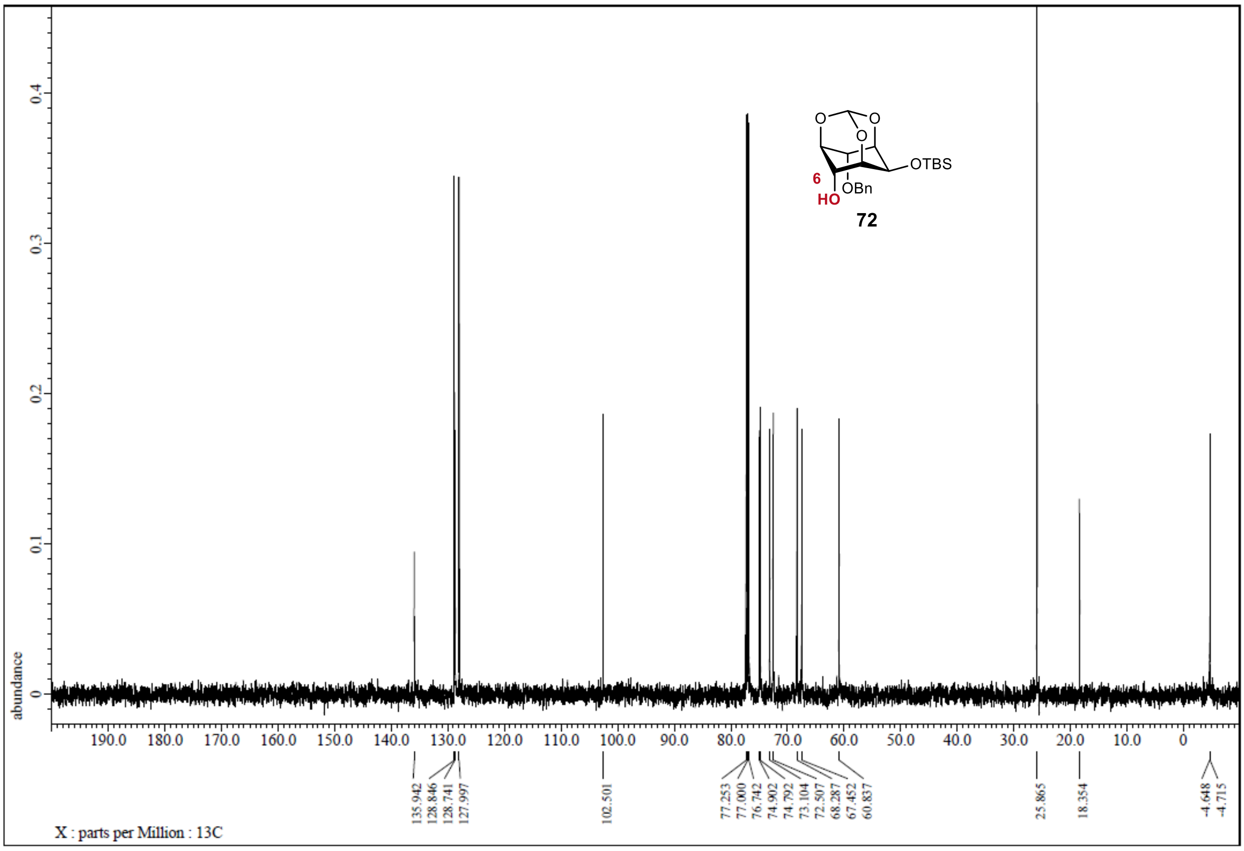

Supplementary Figure 66. ^{1}H and ^{13}C-NMR spectra of compound 72.

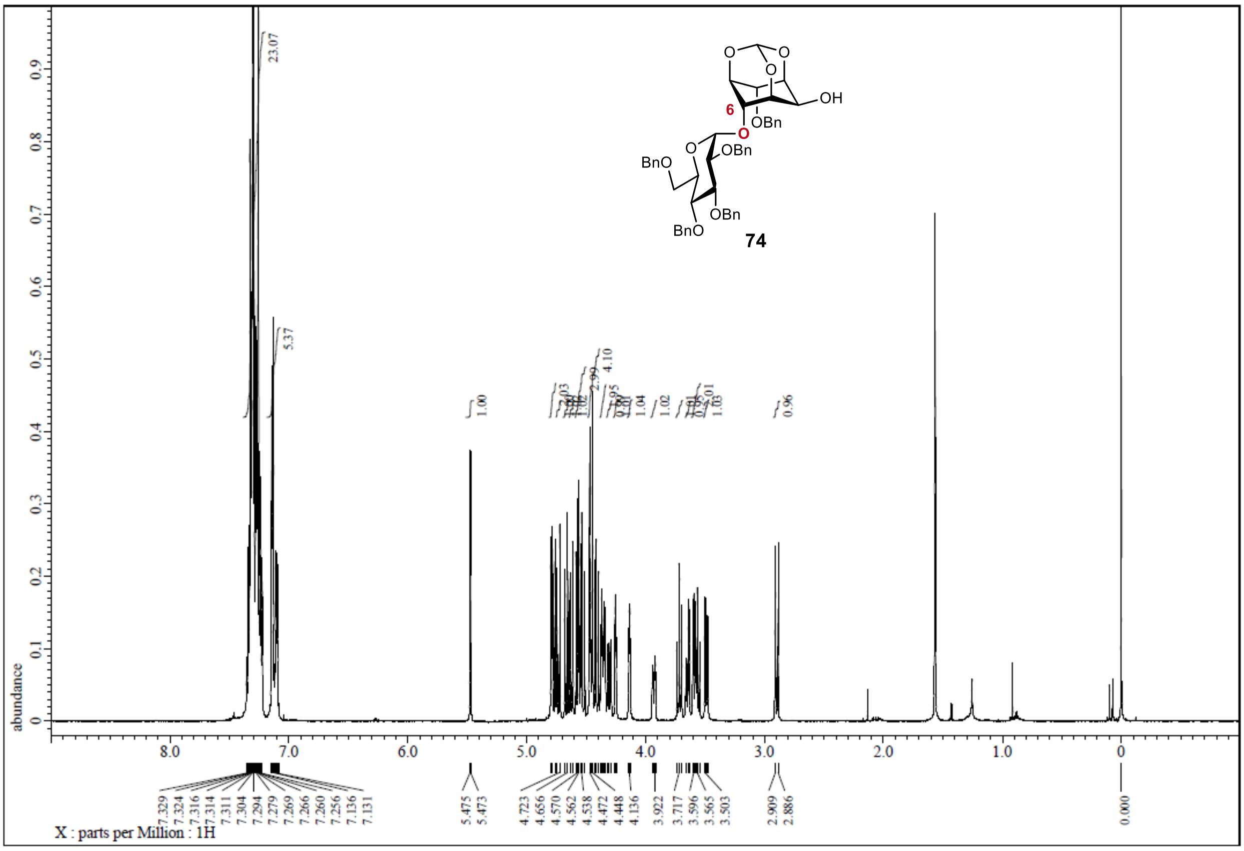

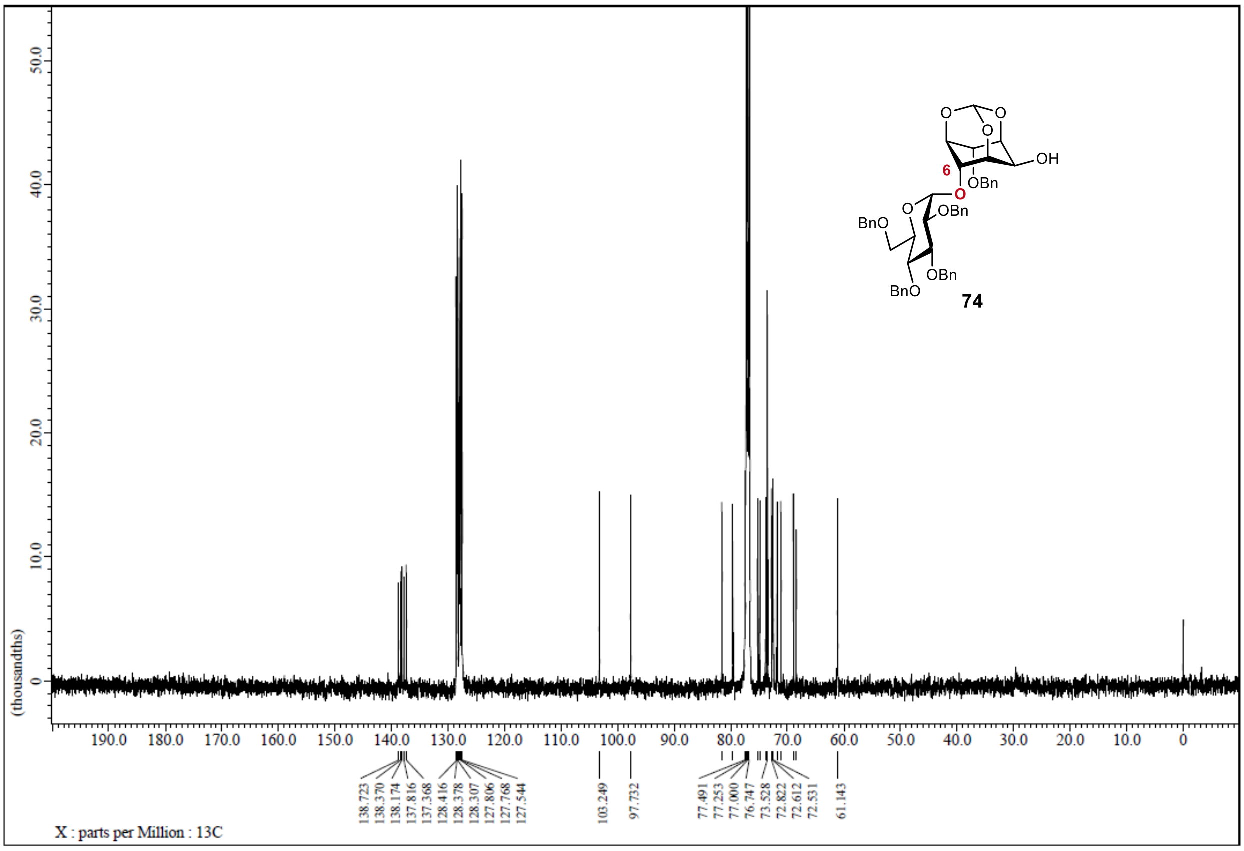

Supplementary Figure 67. ^{1}H and ^{13}C-NMR spectra of compound 74.

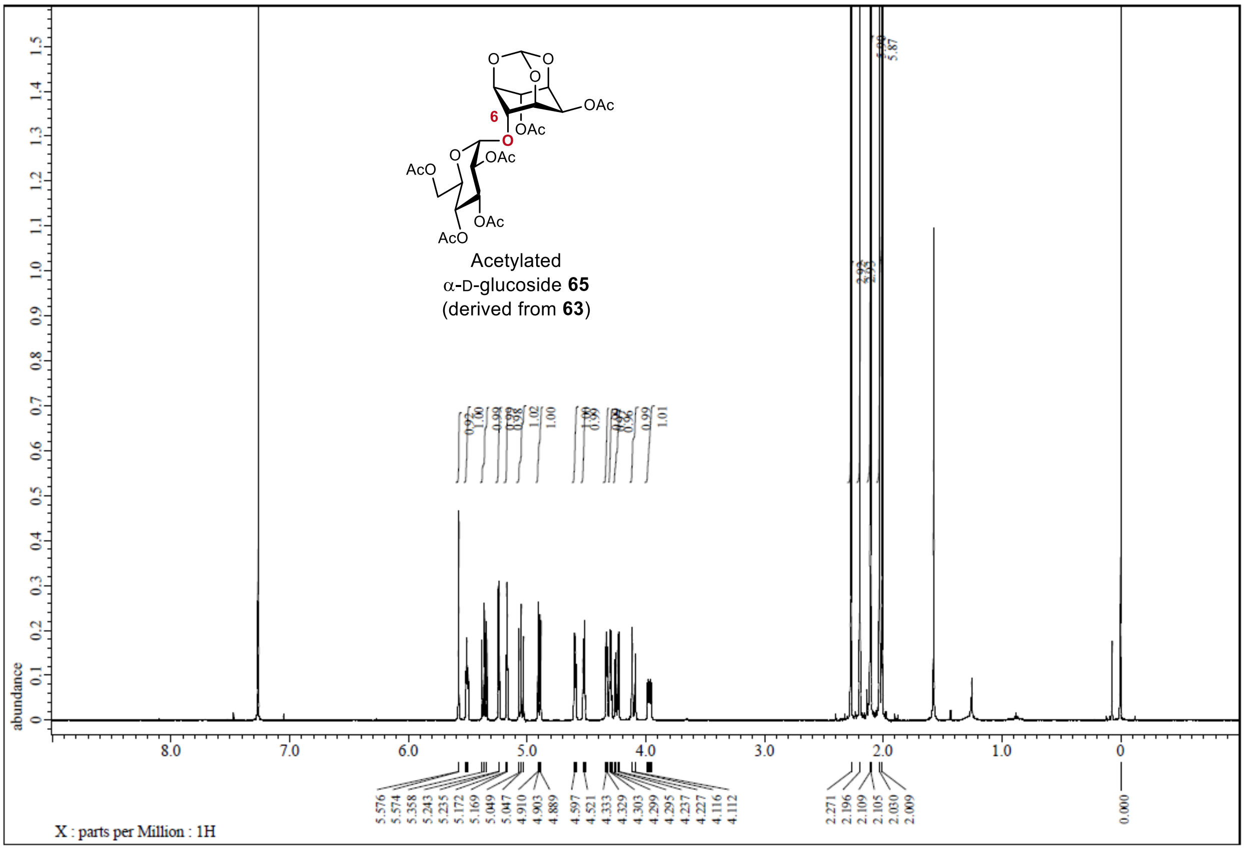

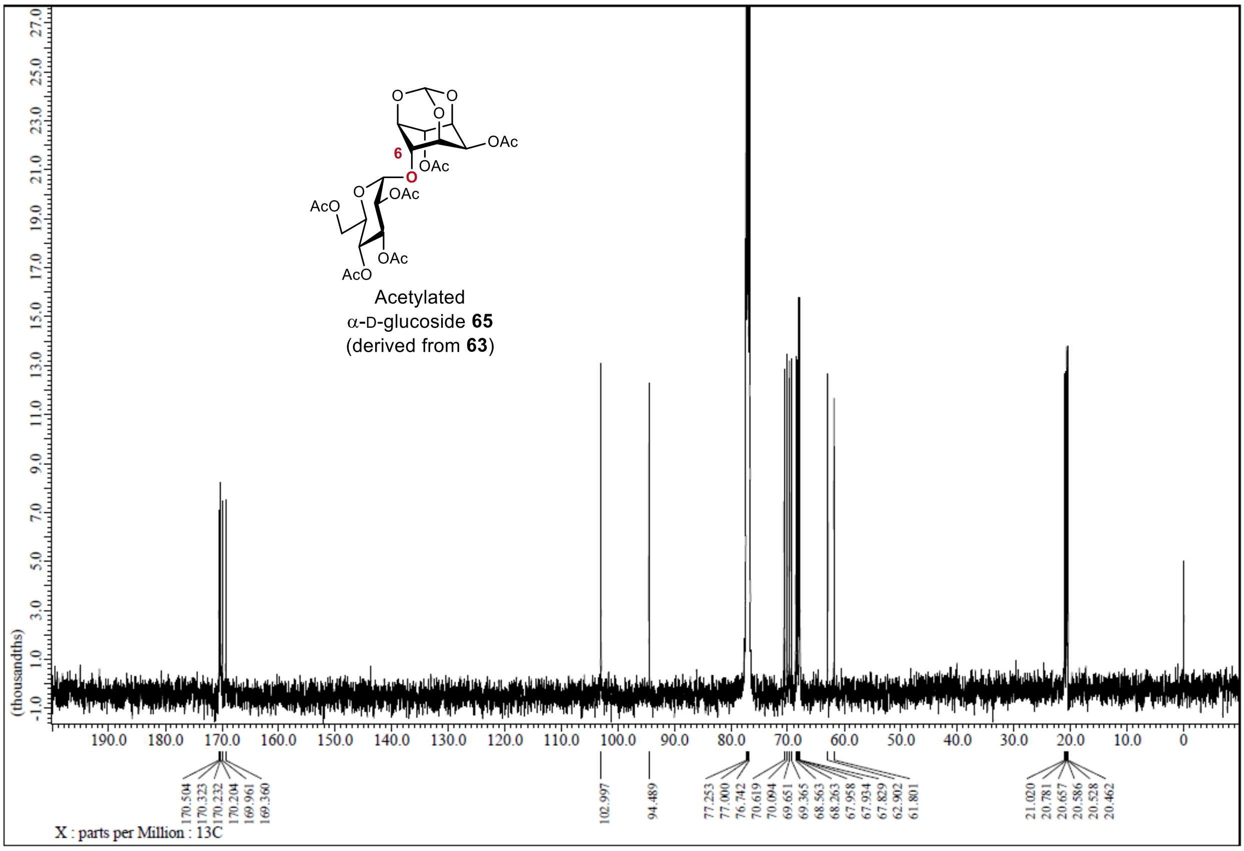

Supplementary Figure 68. ^{1}H and ^{13}C-NMR spectra of compound 65.

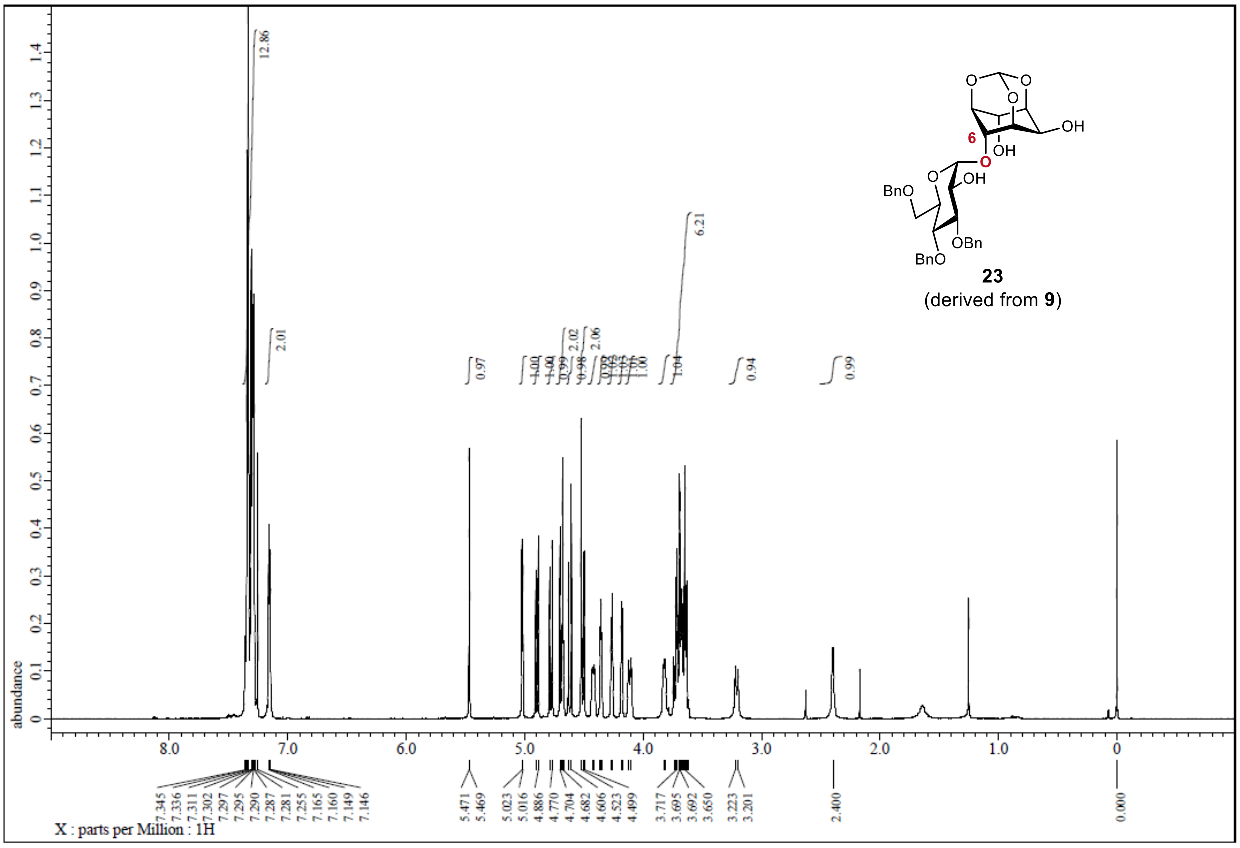

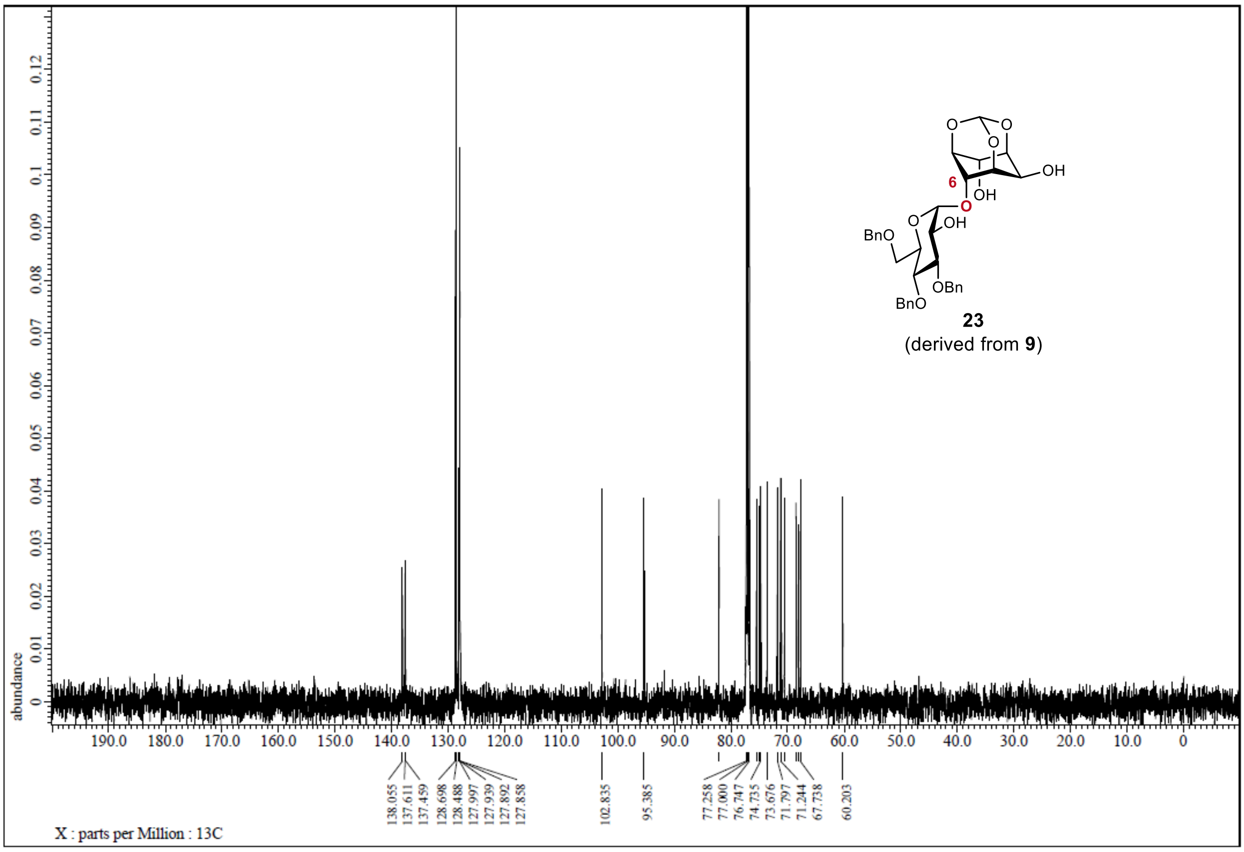

Supplementary Figure 69. ^{1}H and ^{13}C-NMR spectra of compound 23.

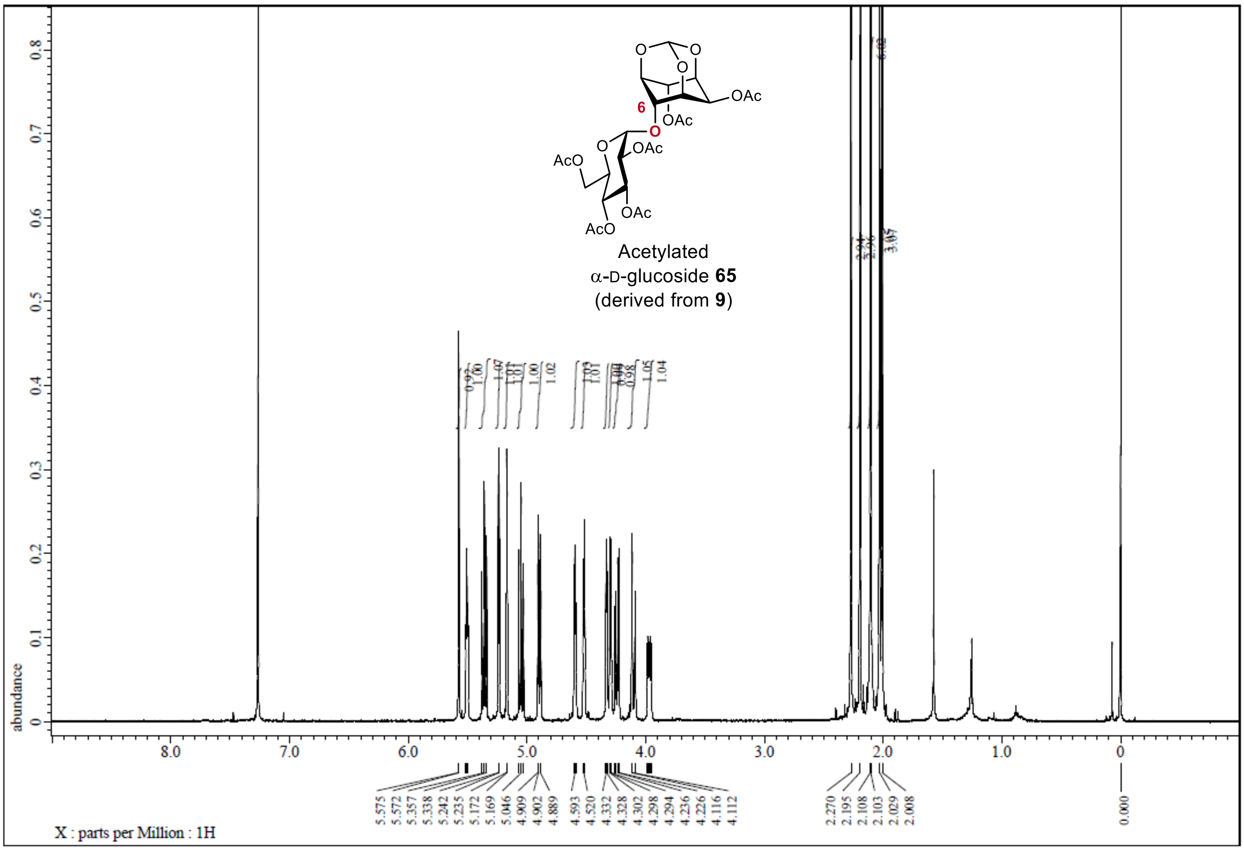

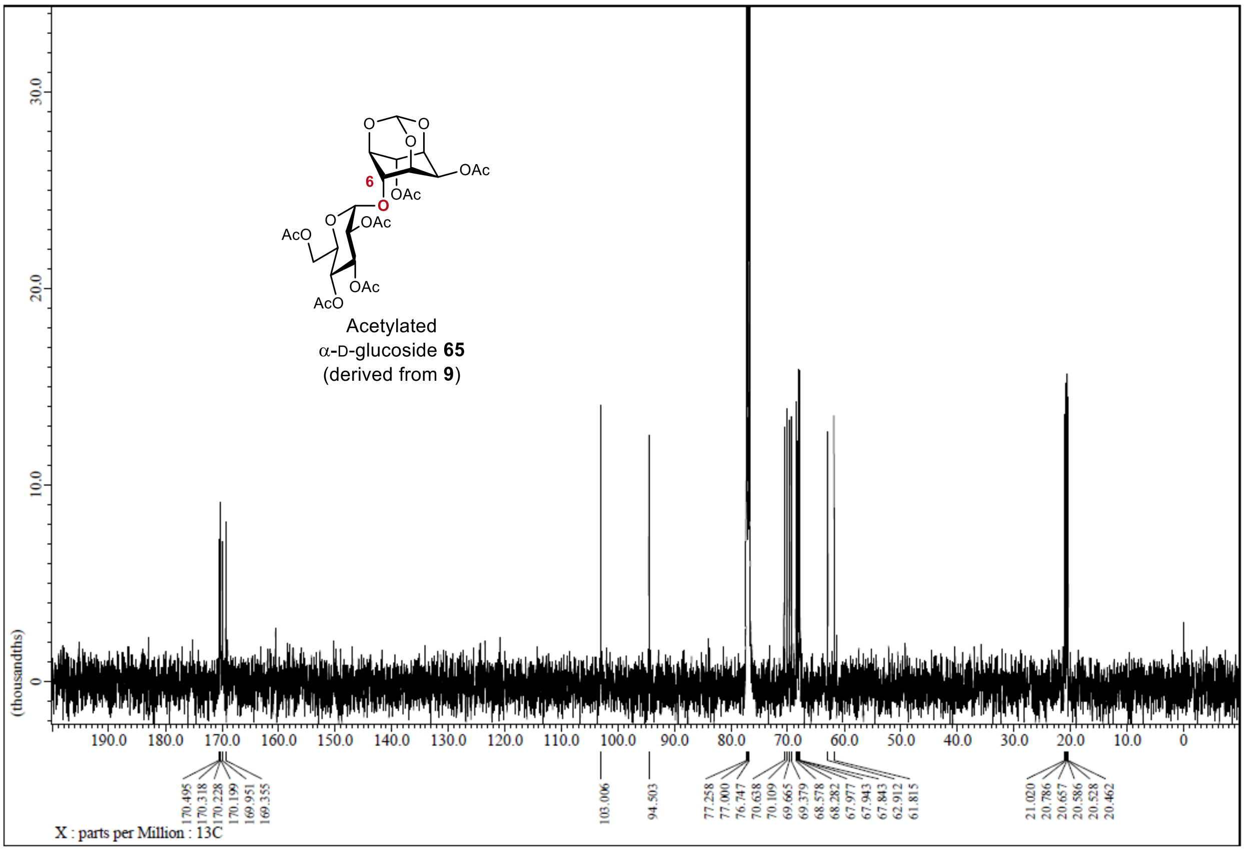

Supplementary Figure 70. ^{1}H and ^{13}C-NMR spectra of compound 65.

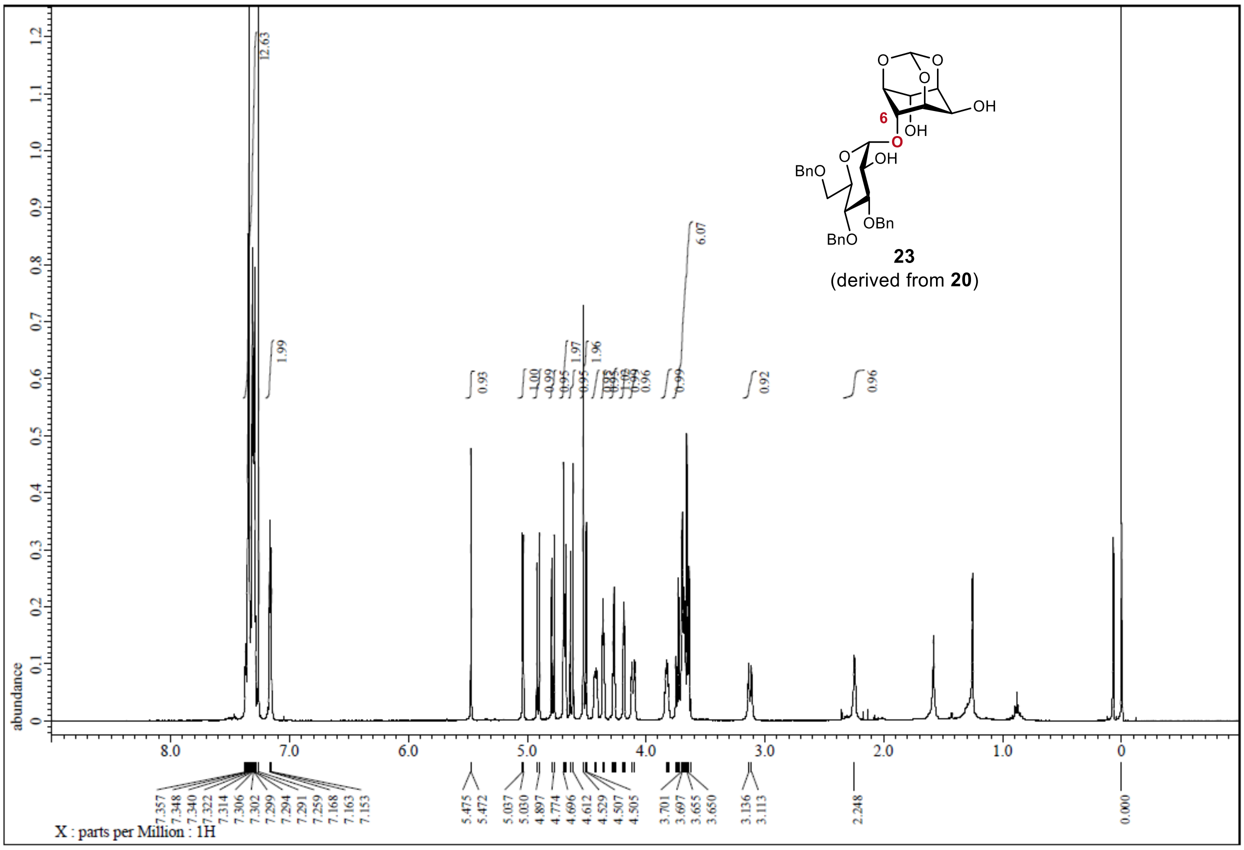

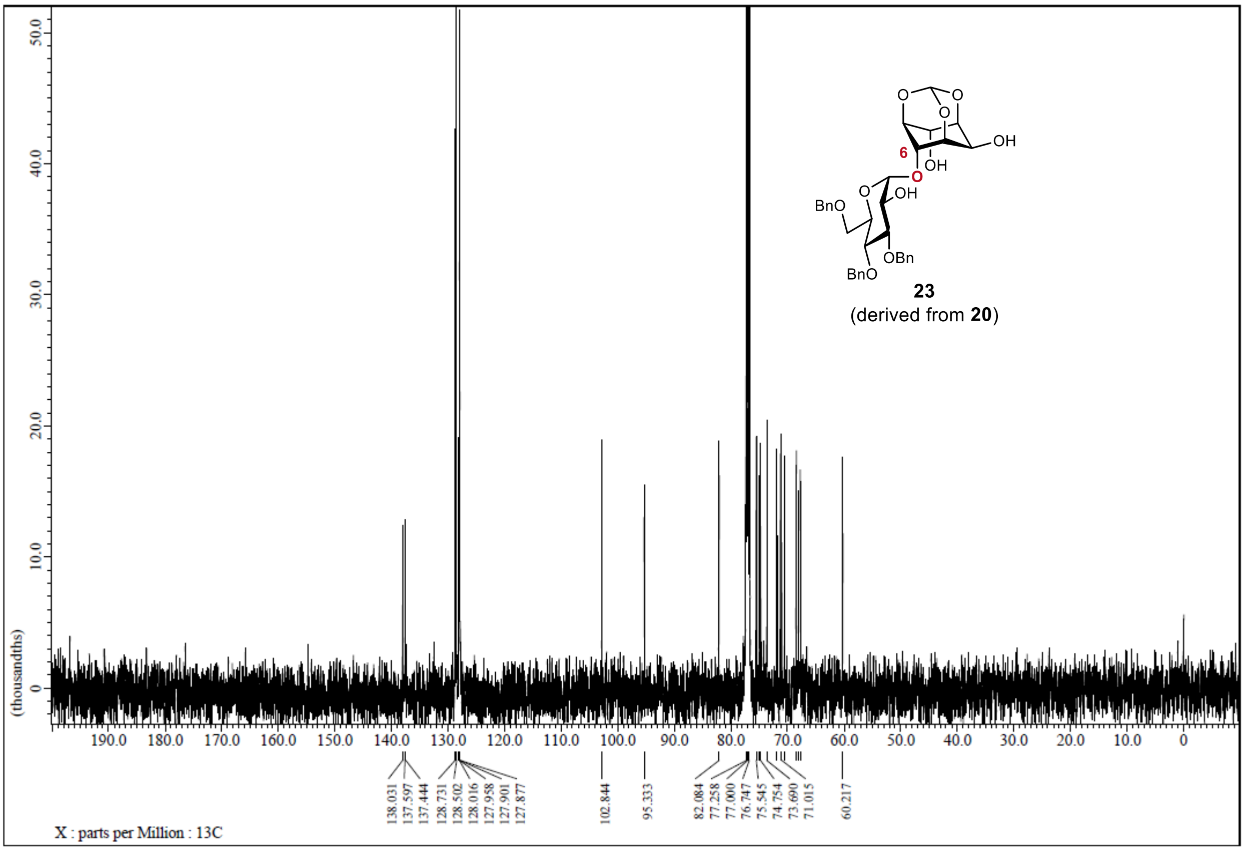

Supplementary Figure 71. ^{1}H and ^{13}C-NMR spectra of compound 23.

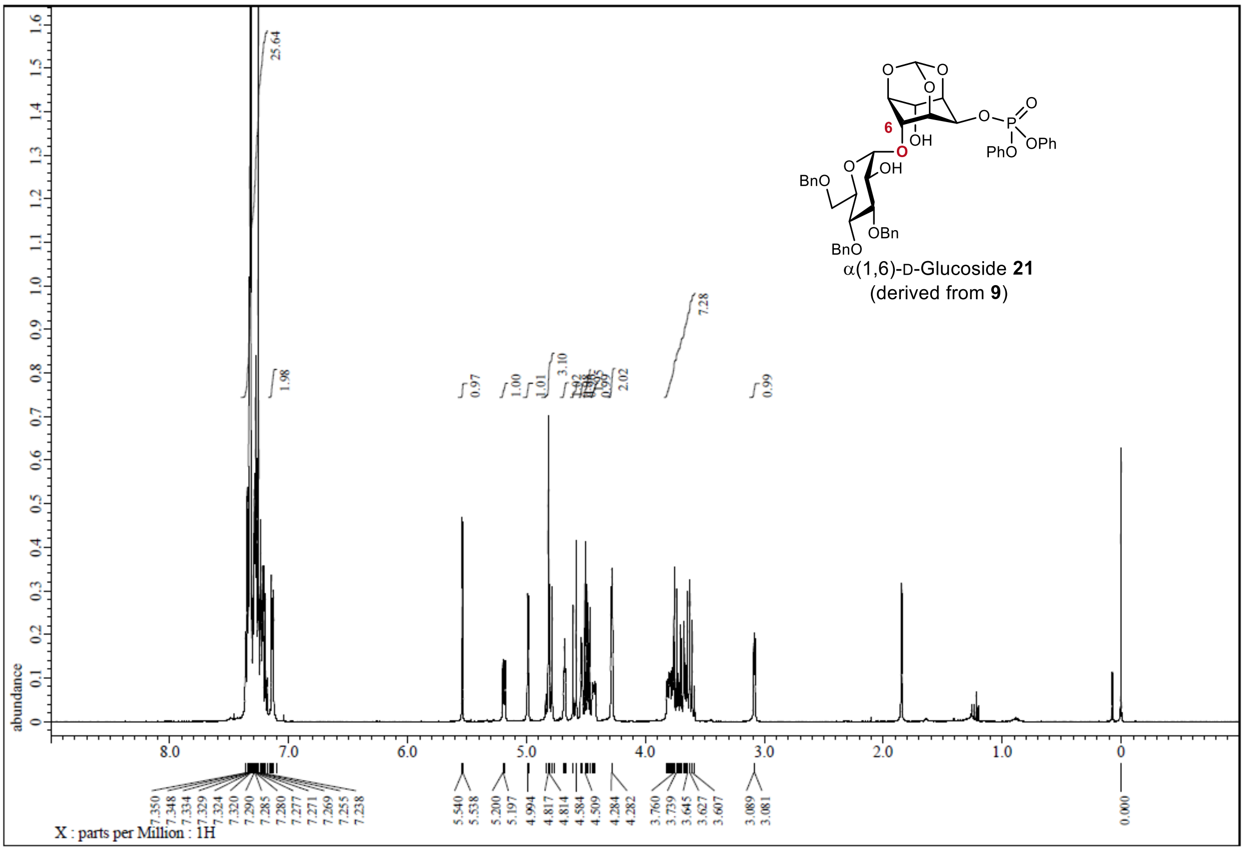

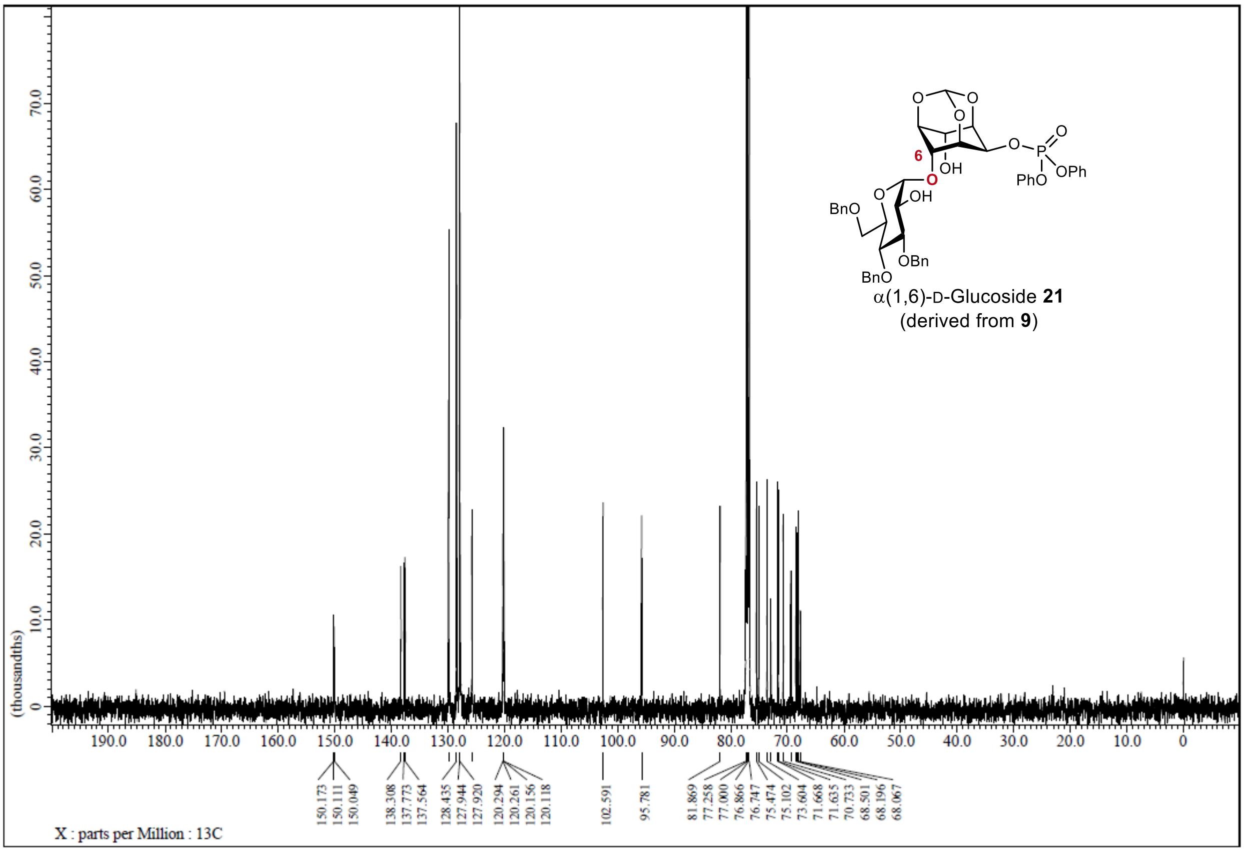

Supplementary Figure 72. ^{1}H and ^{13}C-NMR spectra of compound 21.

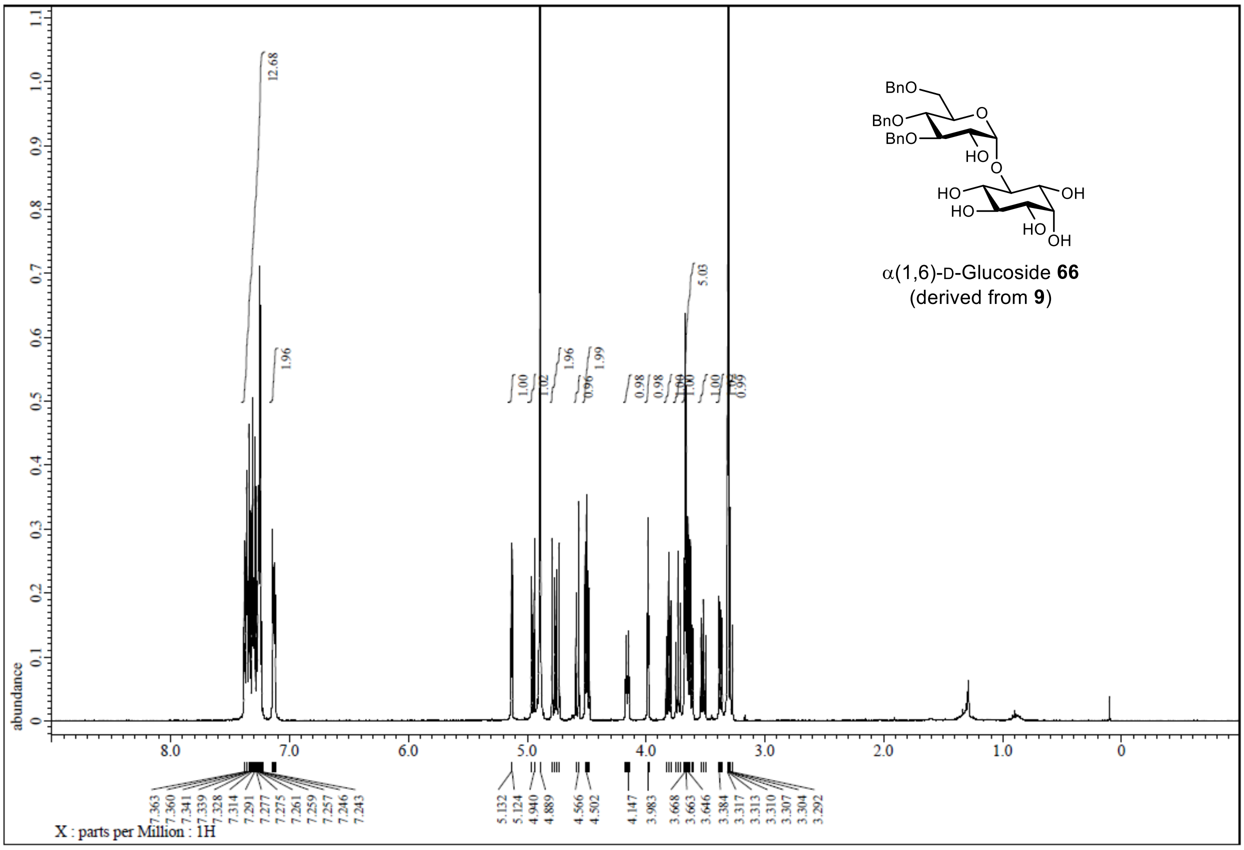

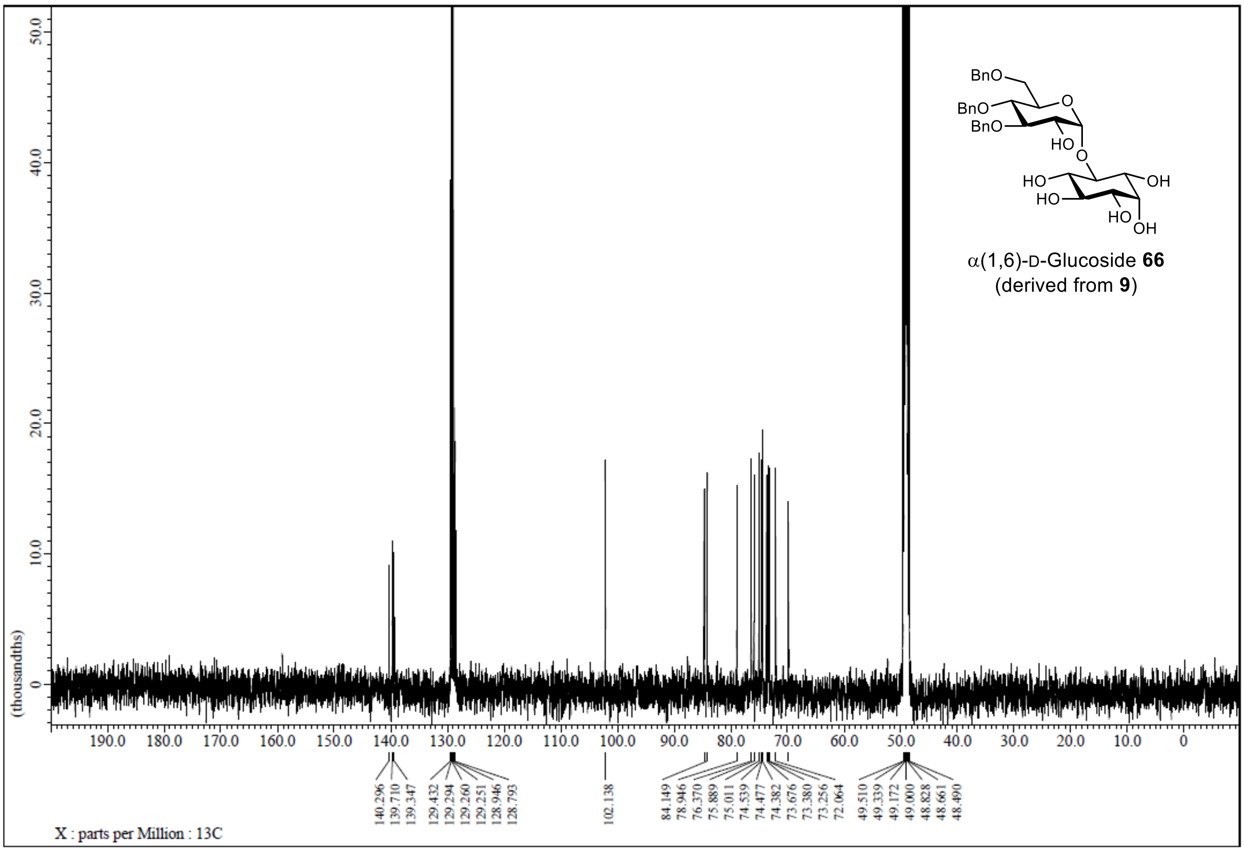

Supplementary Figure 73. ^{1}H and ^{13}C-NMR spectra of compound 66.

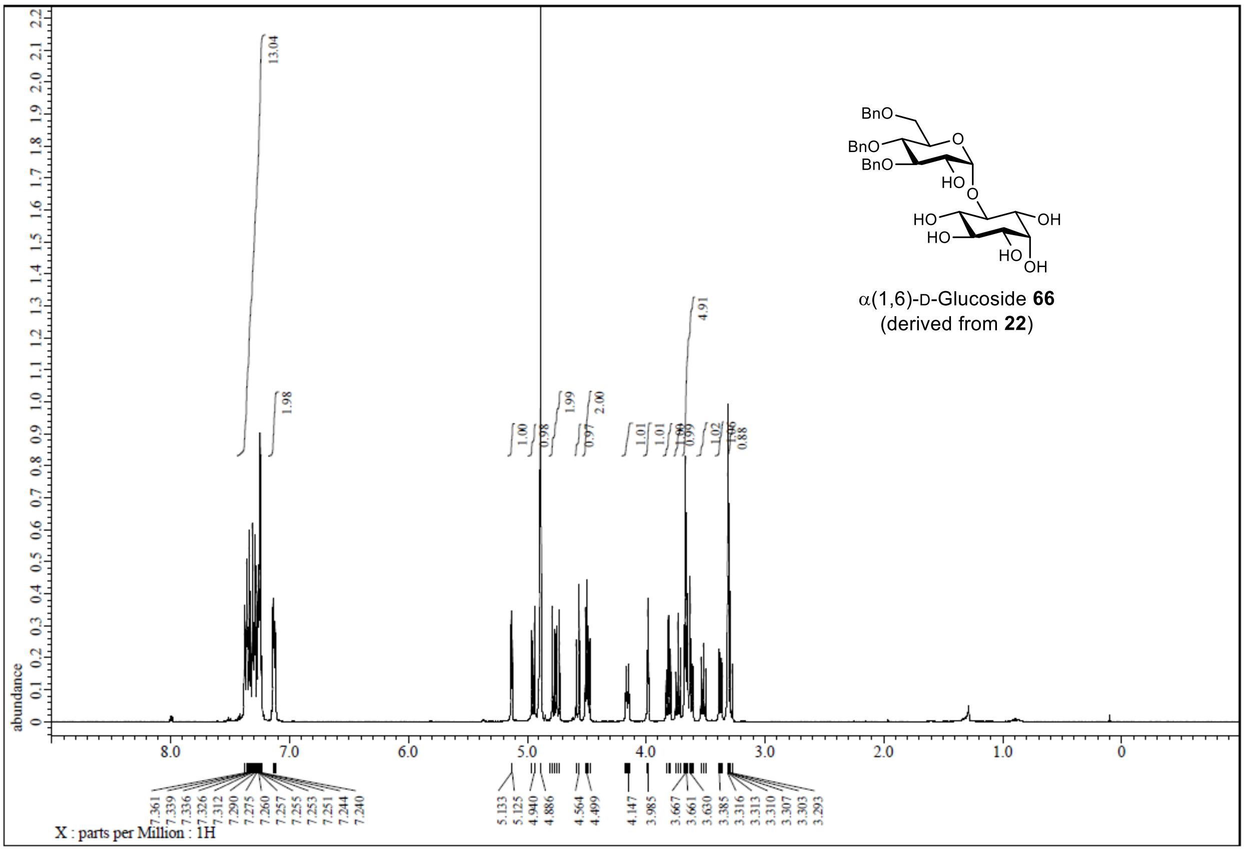

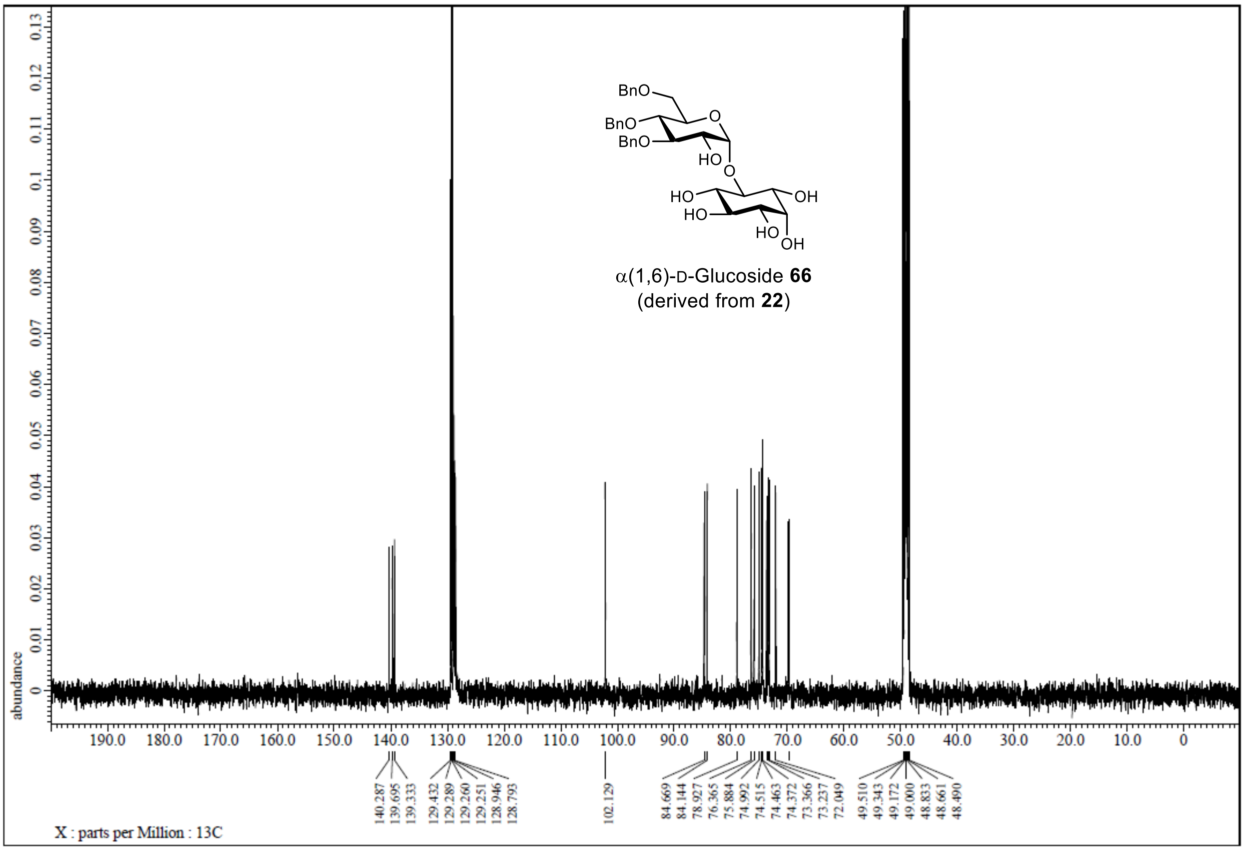

Supplementary Figure 74. ^{1}H and ^{13}C-NMR spectra of compound 66.

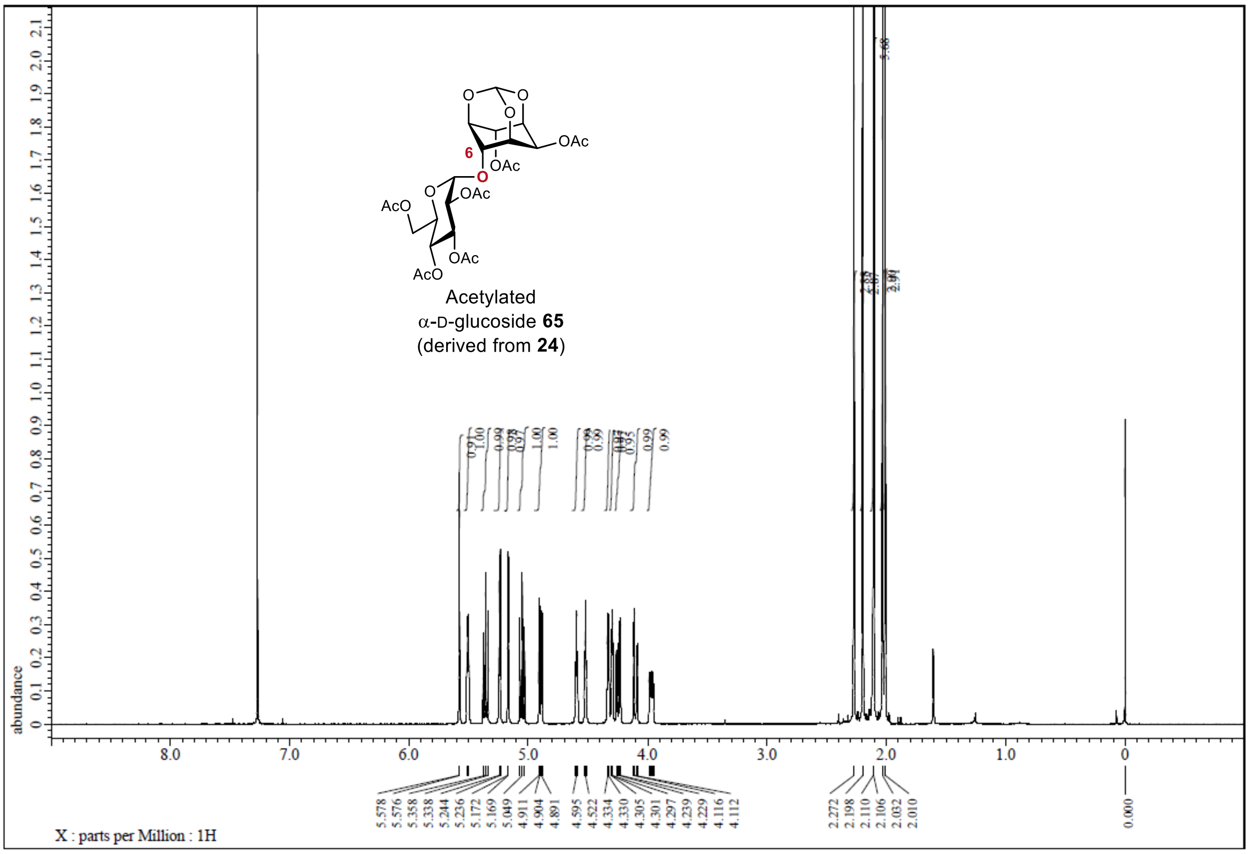

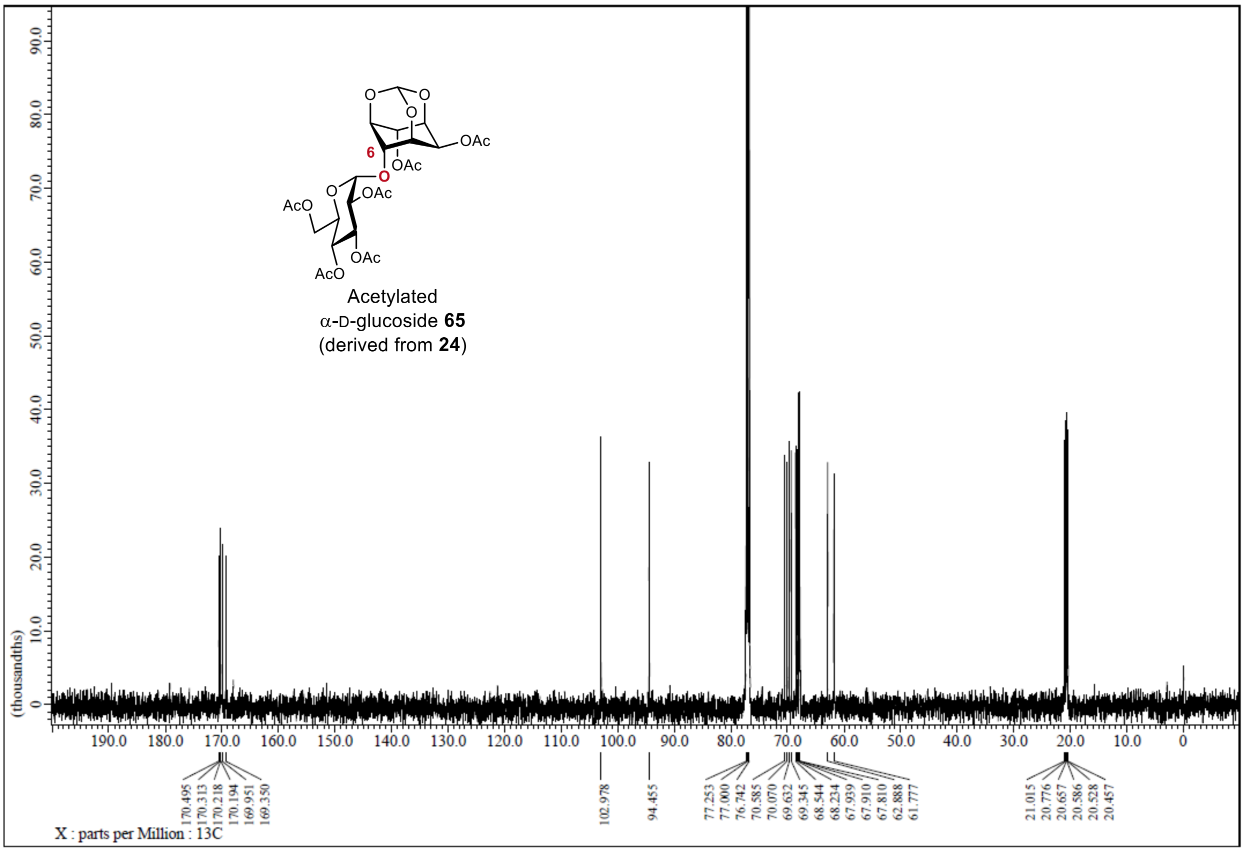

Supplementary Figure 75. ^{1}H and ^{13}C-NMR spectra of compound 65.

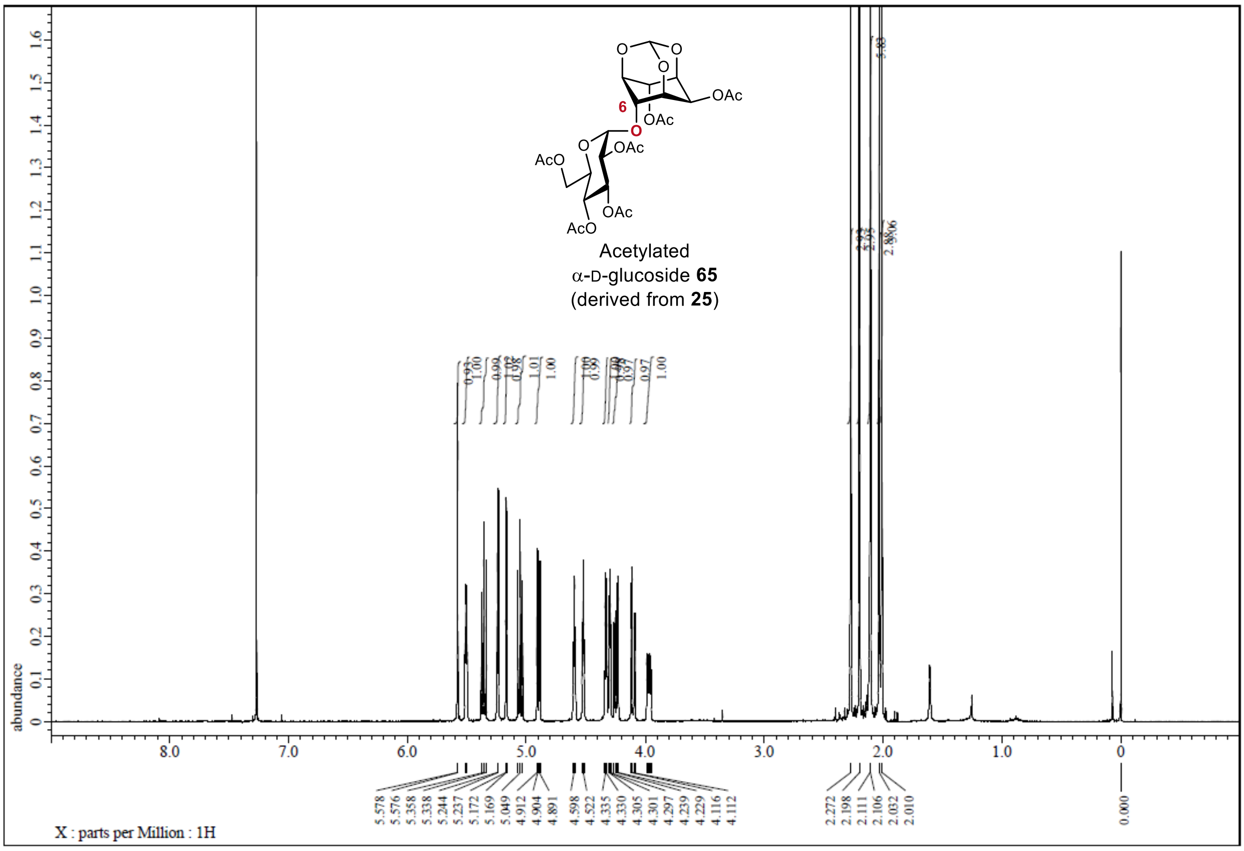

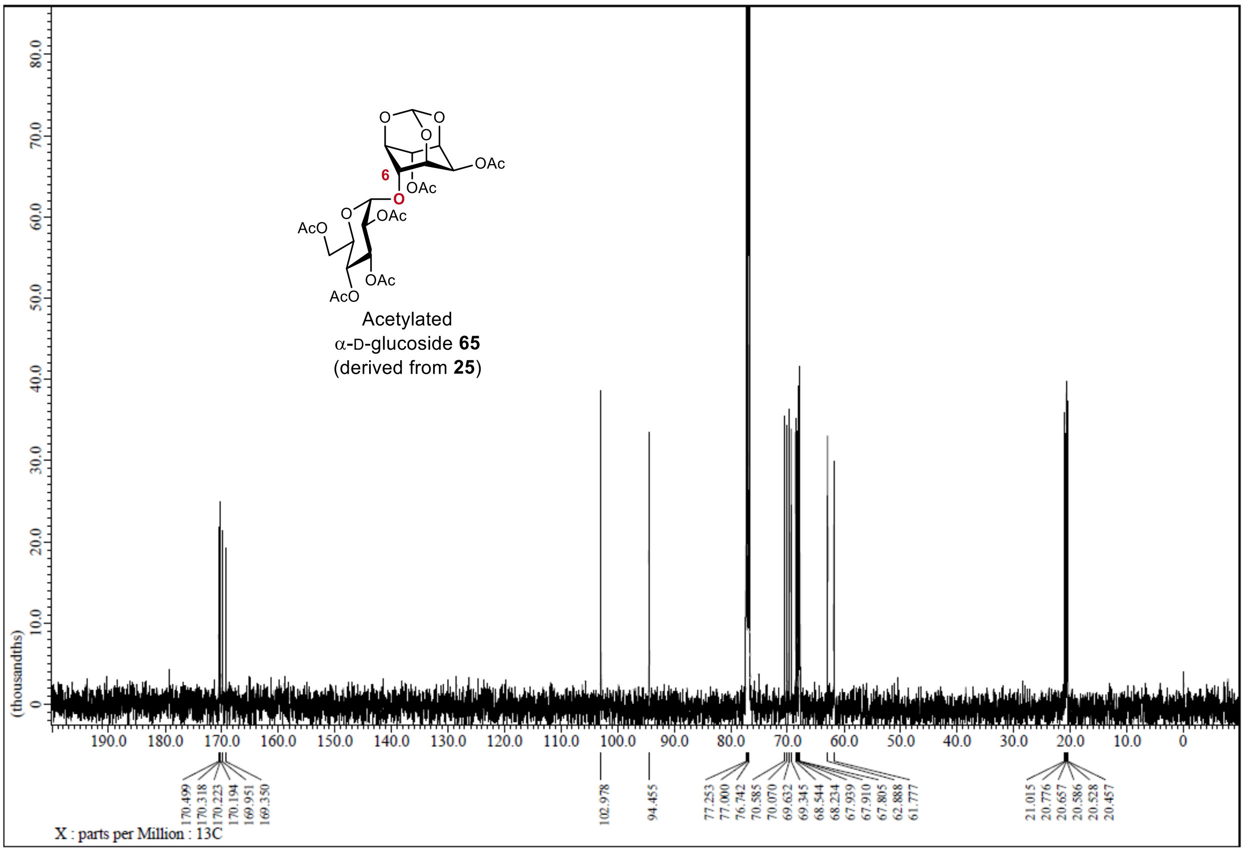

Supplementary Figure 76. ^{1}H and ^{13}C-NMR spectra of compound 65.

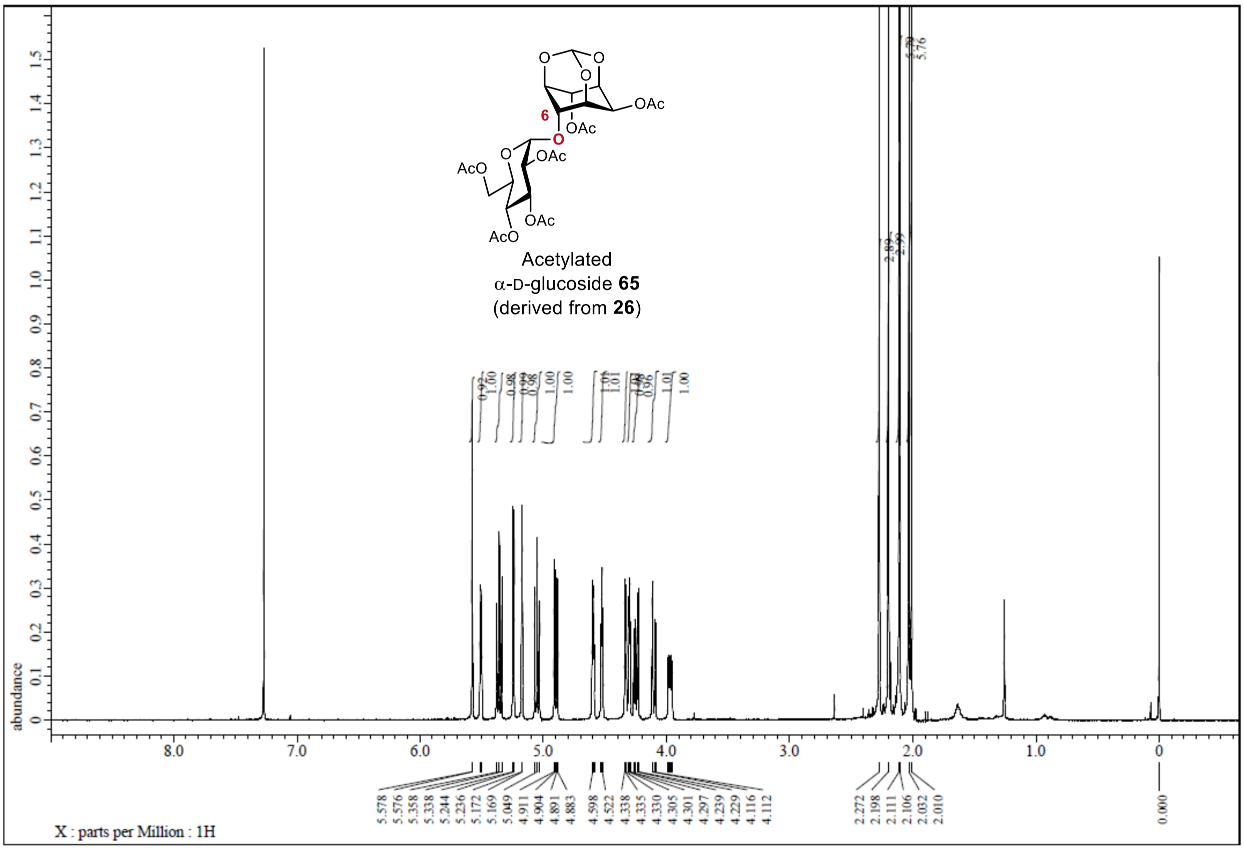

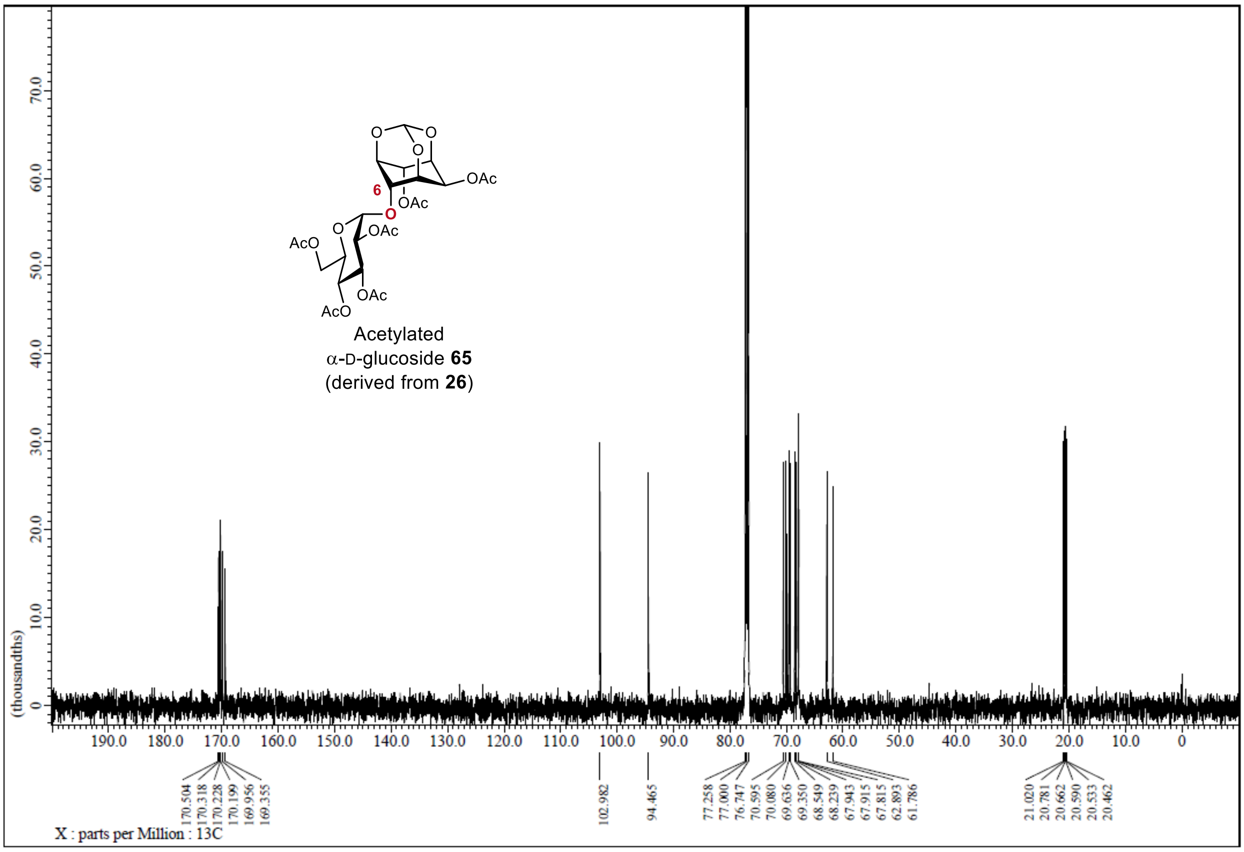

Supplementary Figure 77. ^{1}H and ^{13}C-NMR spectra of compound 65.

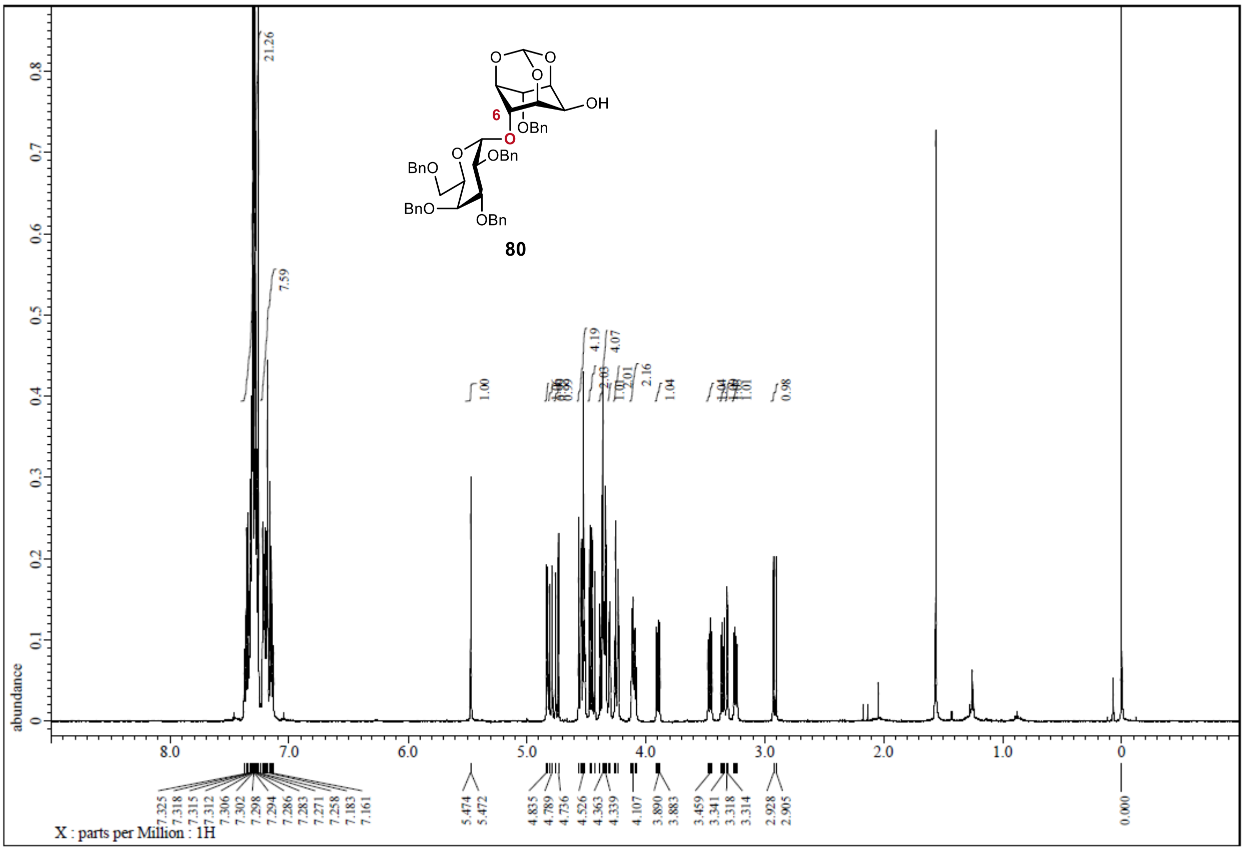

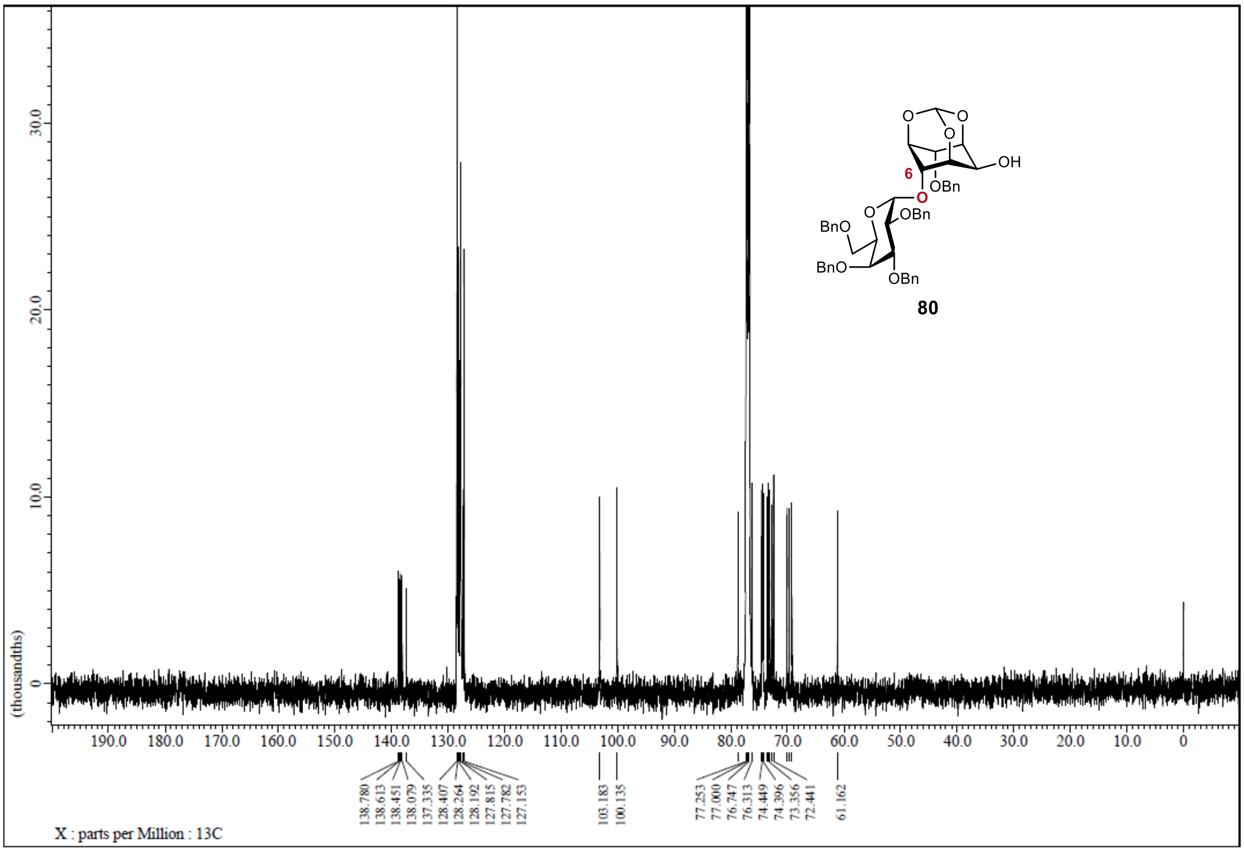

Supplementary Figure 78. ^{1}H and ^{13}C-NMR spectra of compound 80.

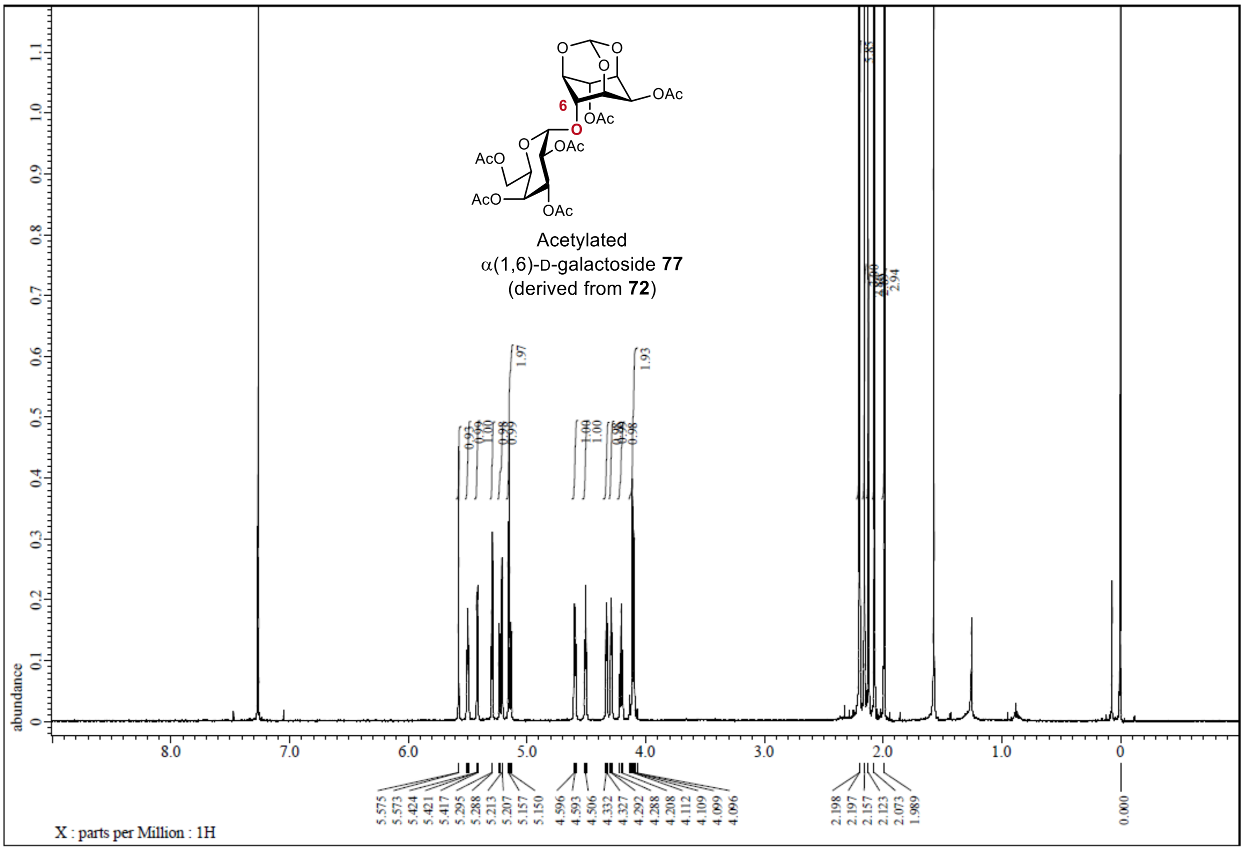

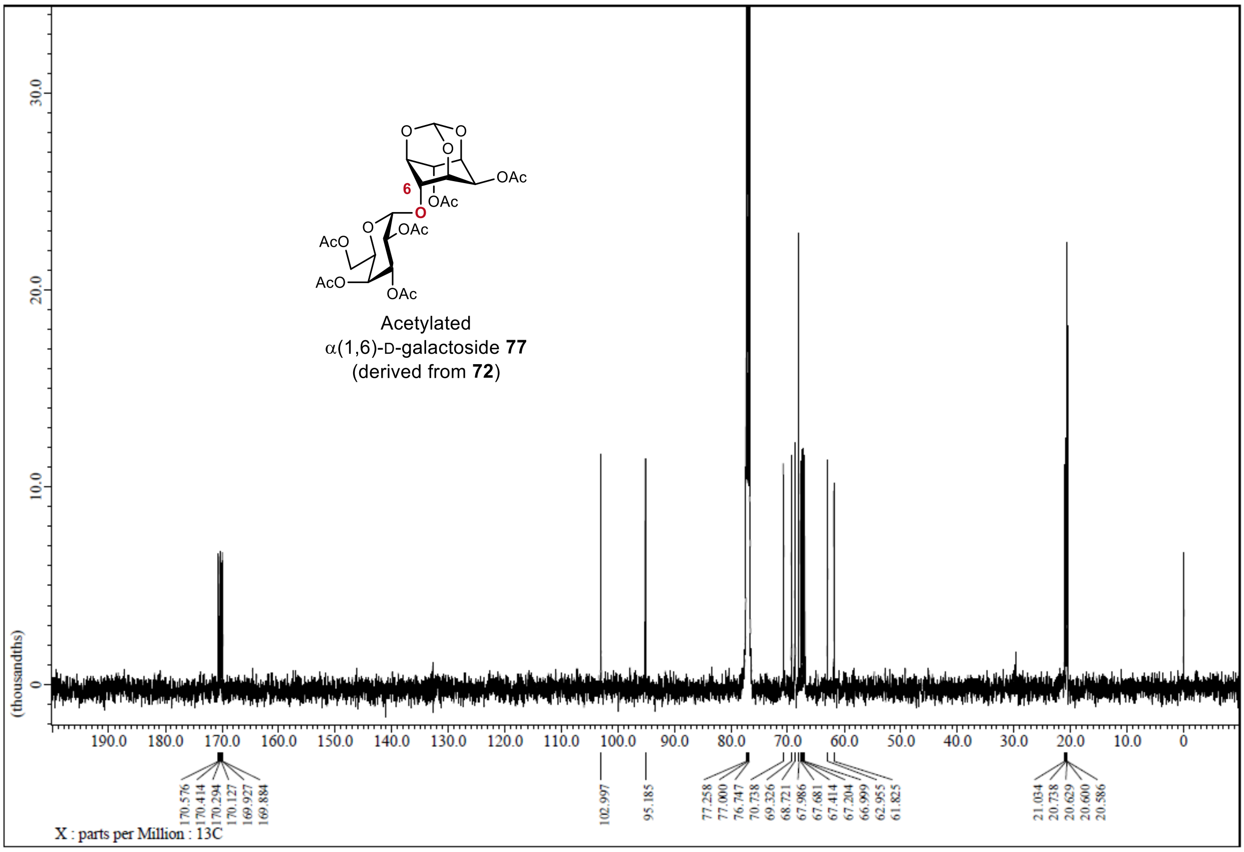

Supplementary Figure 79. ^{1}H and ^{13}C-NMR spectra of compound 77.

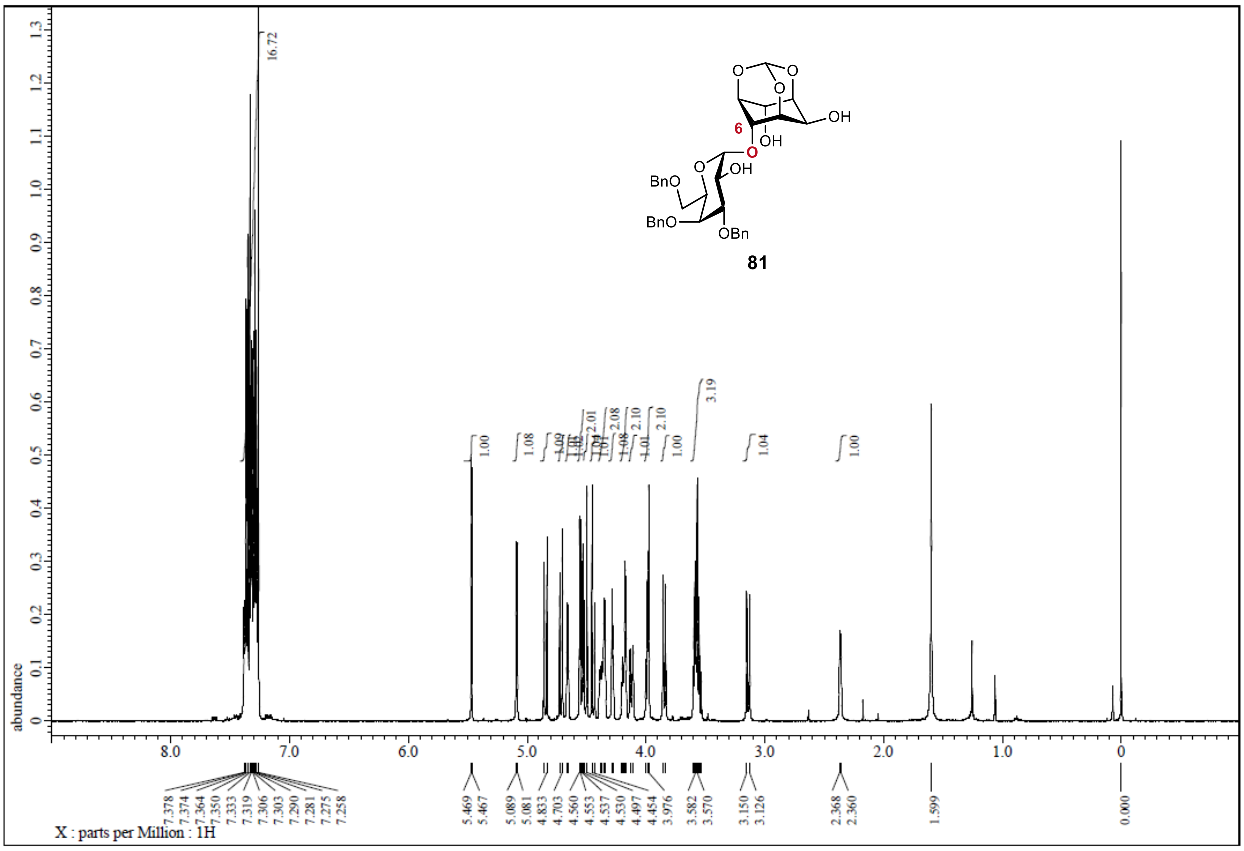

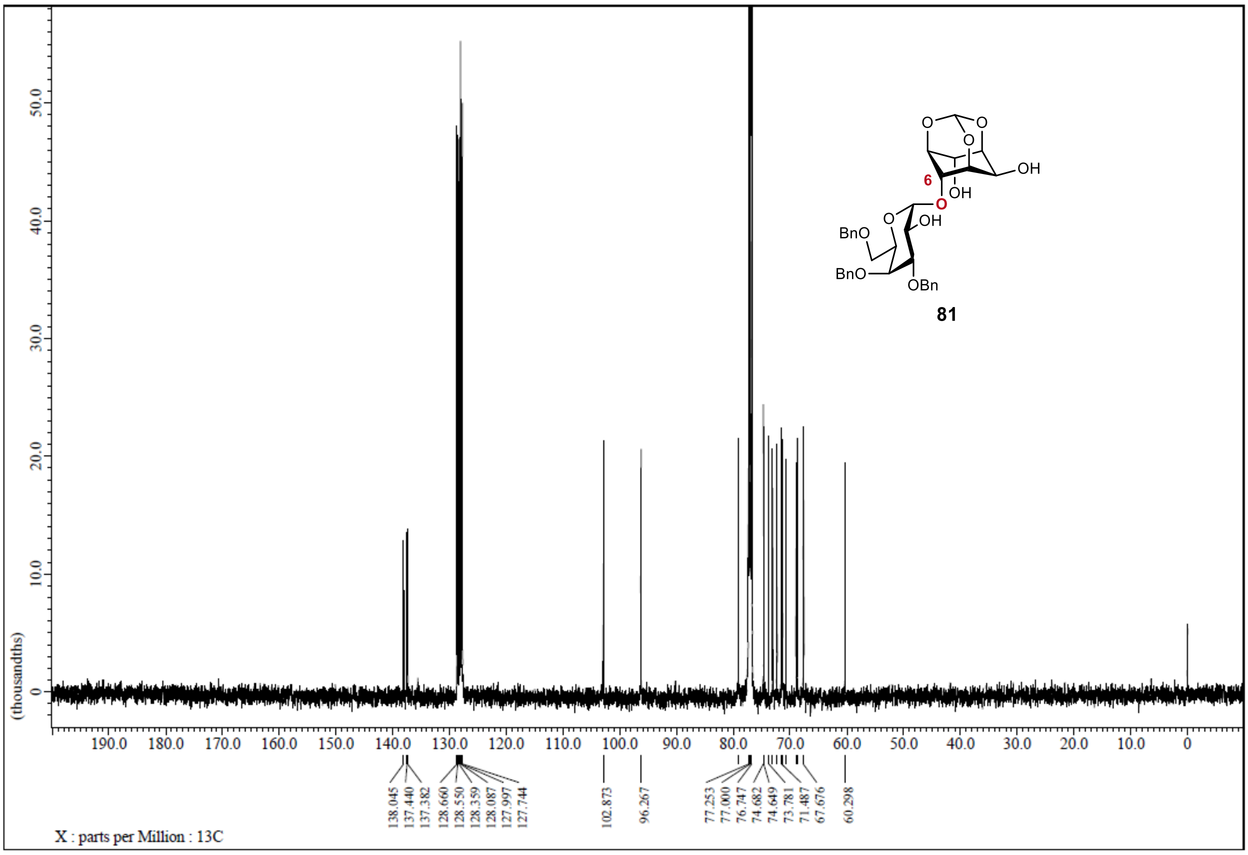

Supplementary Figure 80. ^{1}**H and** ^{13}**C-NMR spectra of compound 81.**

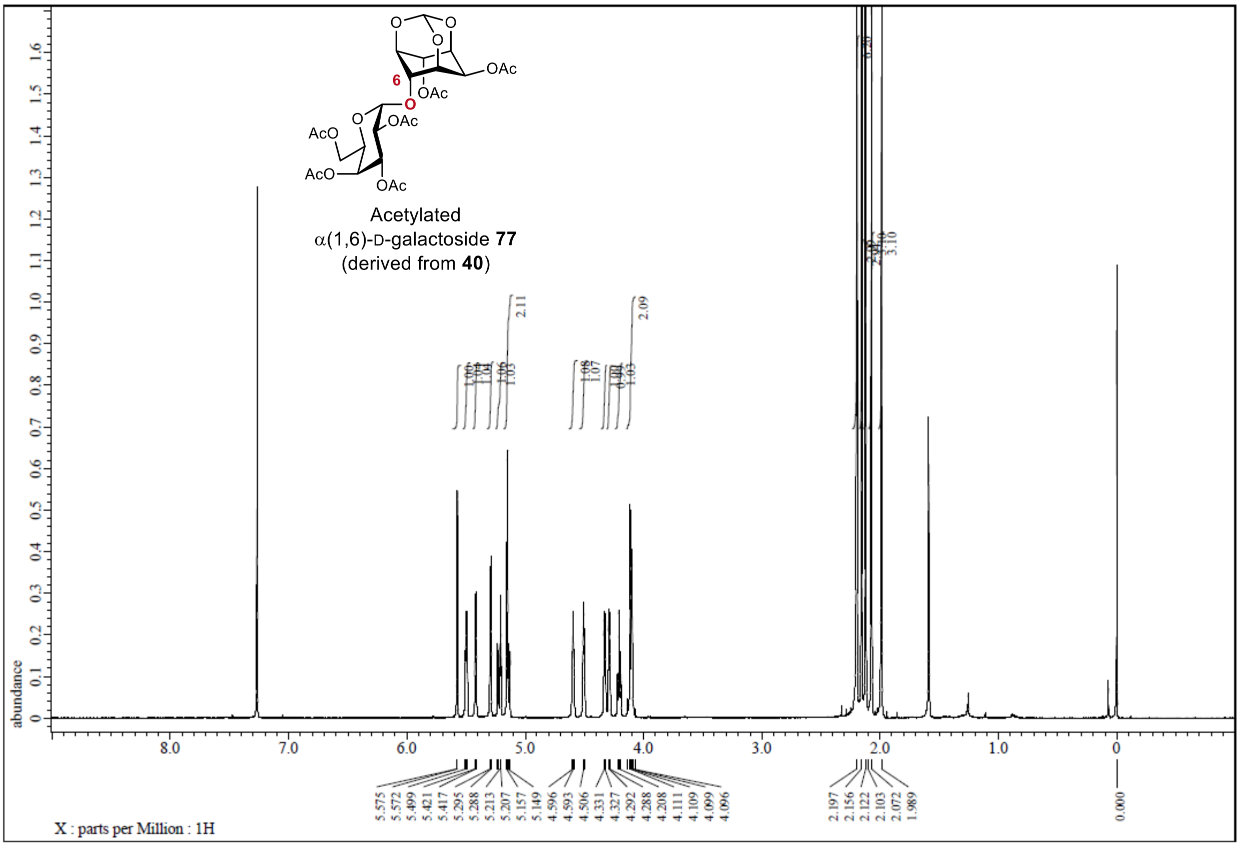

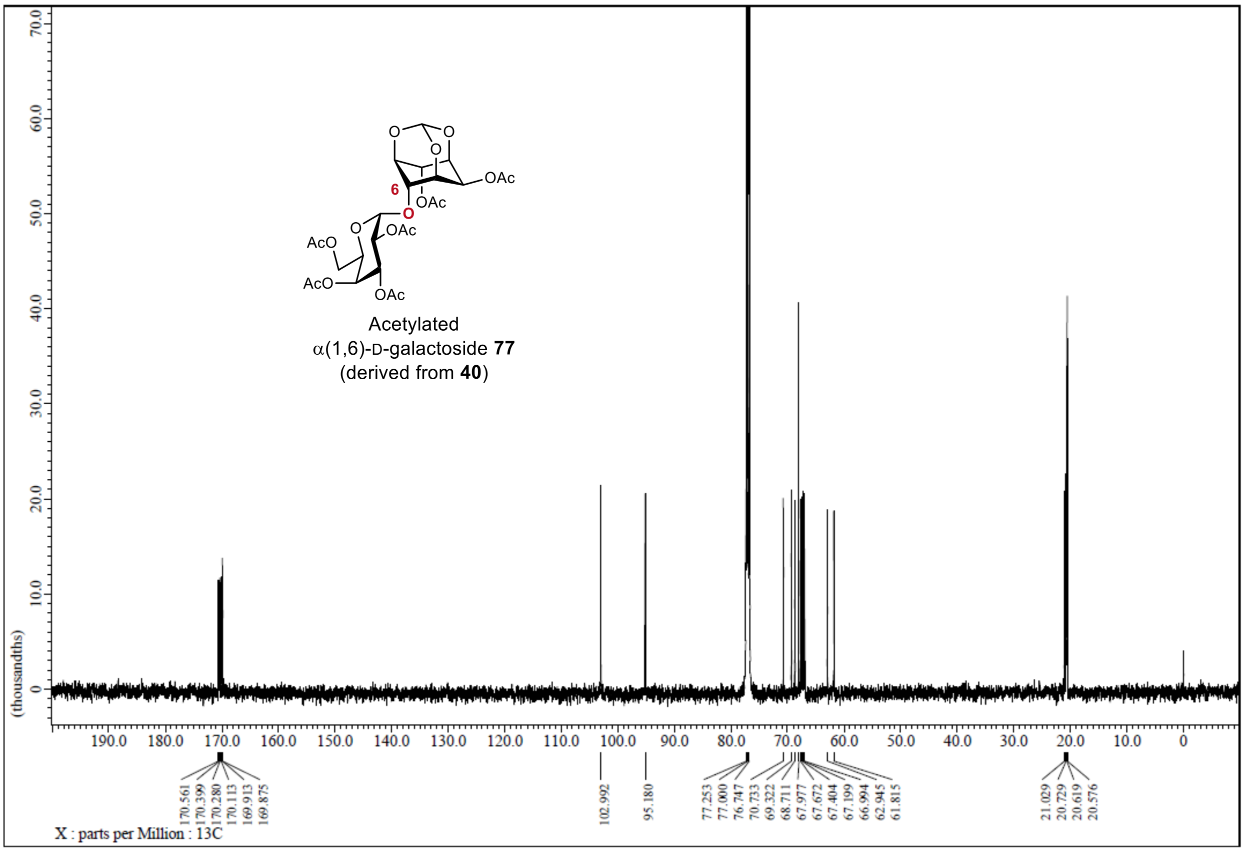

Supplementary Figure 81. ^{1}H and ^{13}C-NMR spectra of compound 77.

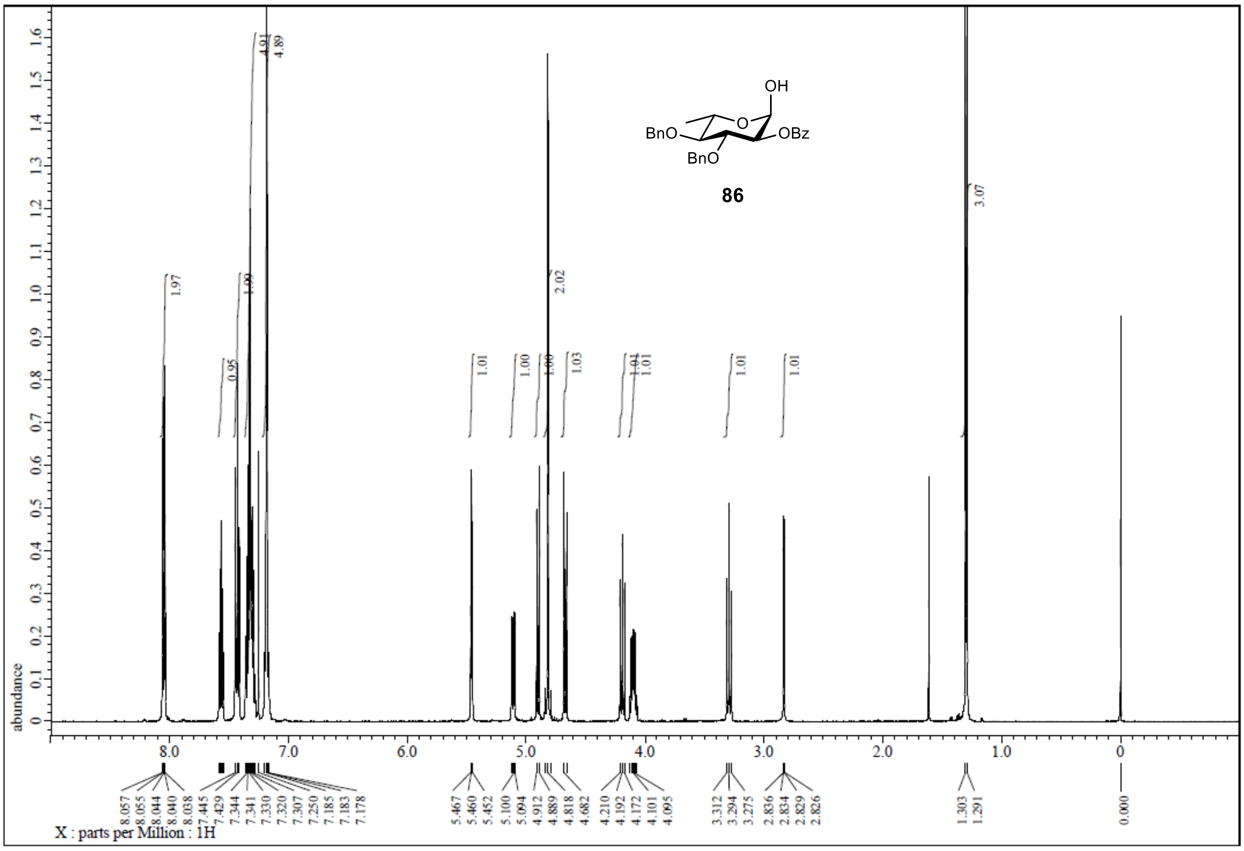

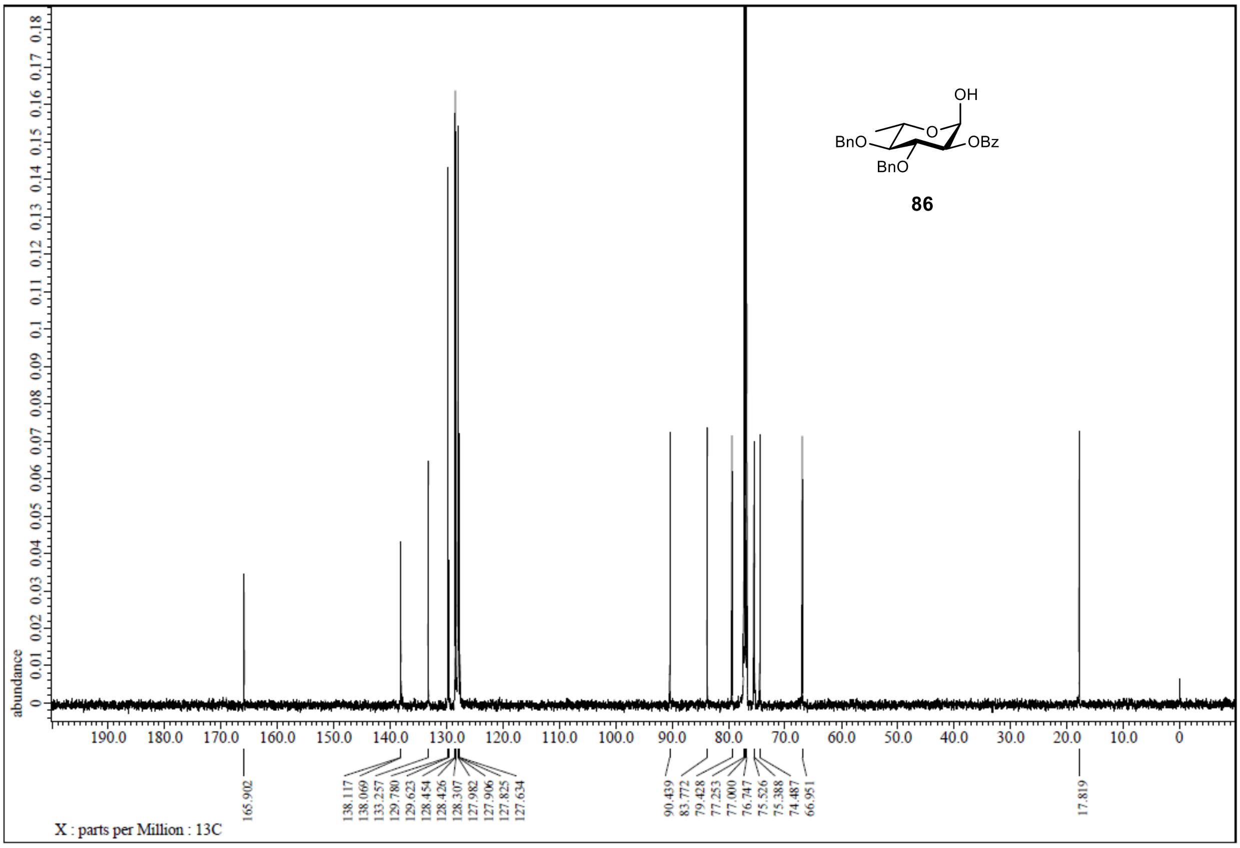

Supplementary Figure 82. ^{1}H and ^{13}C-NMR spectra of compound 86.

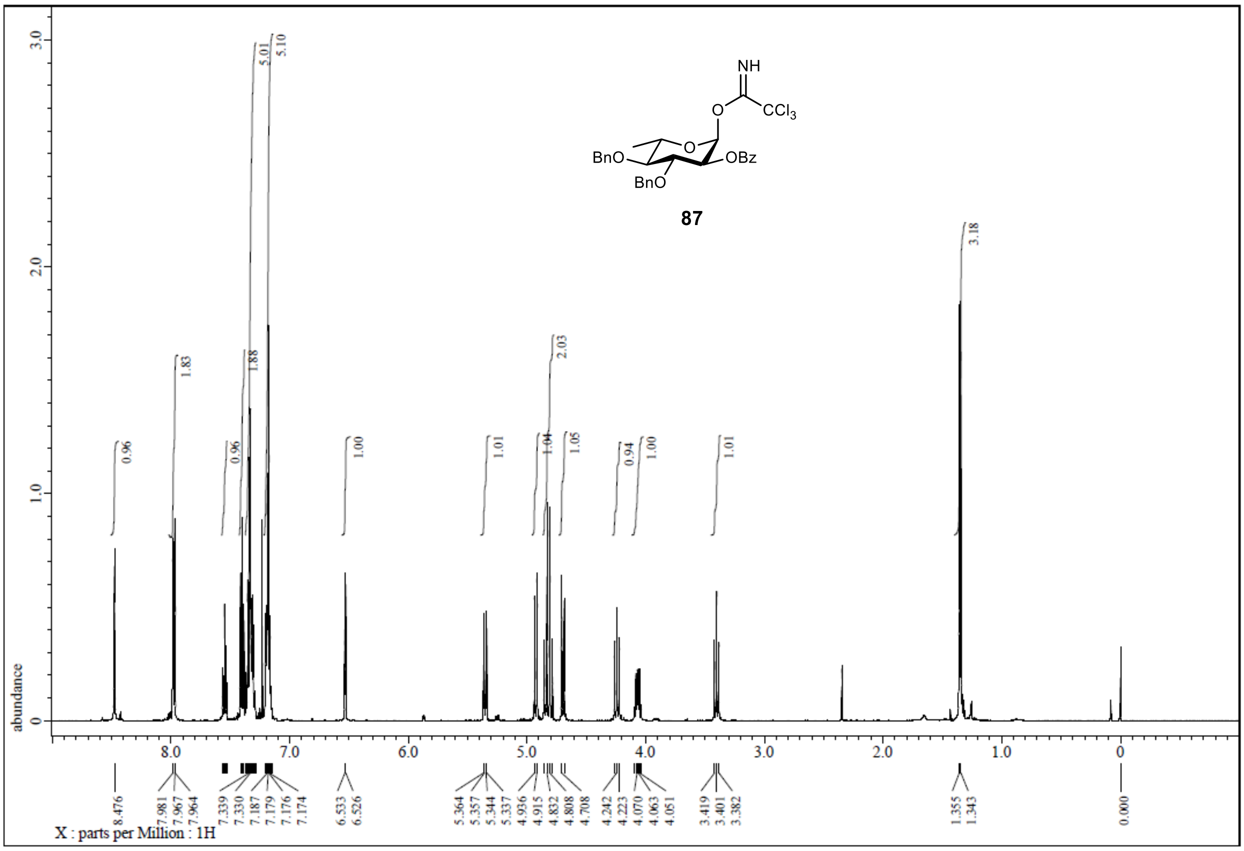

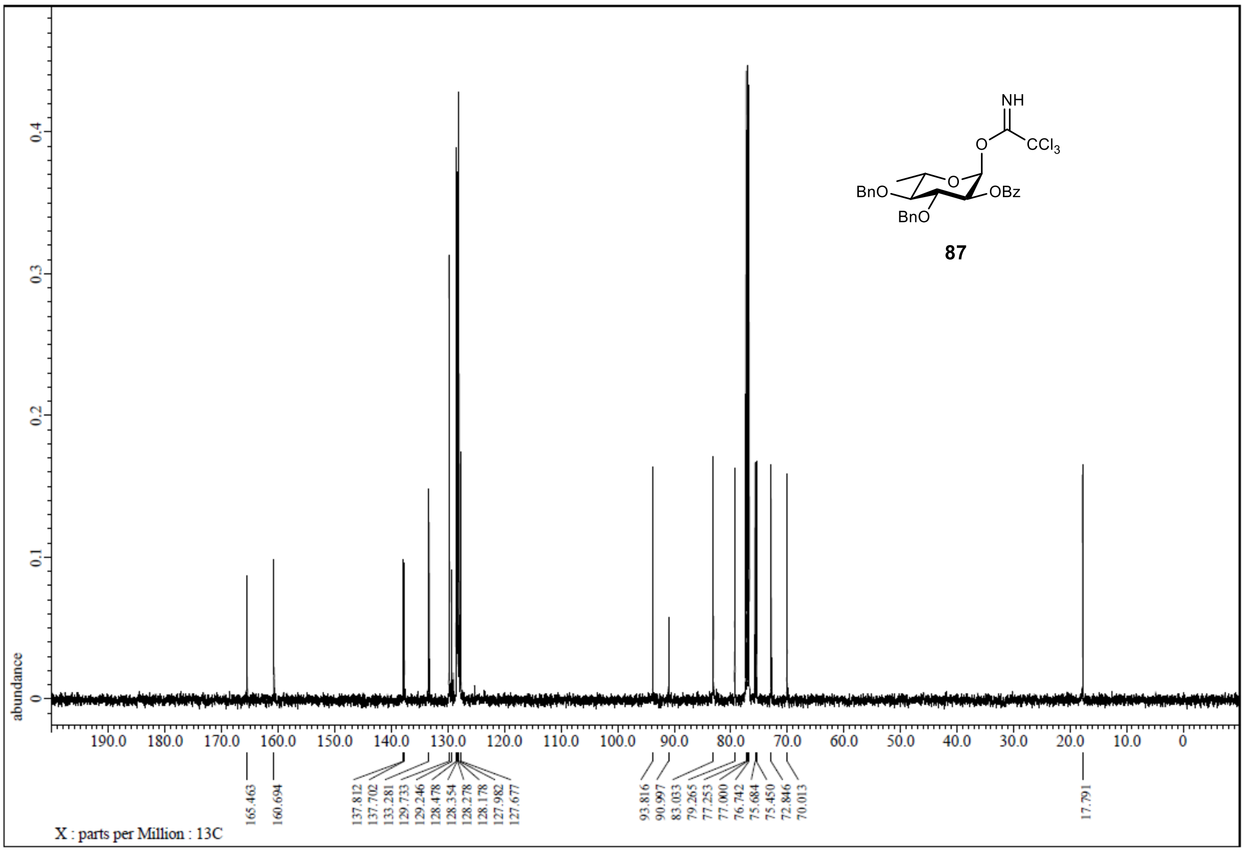

Supplementary Figure 83. ^{1}H and ^{13}C-NMR spectra of compound 87.

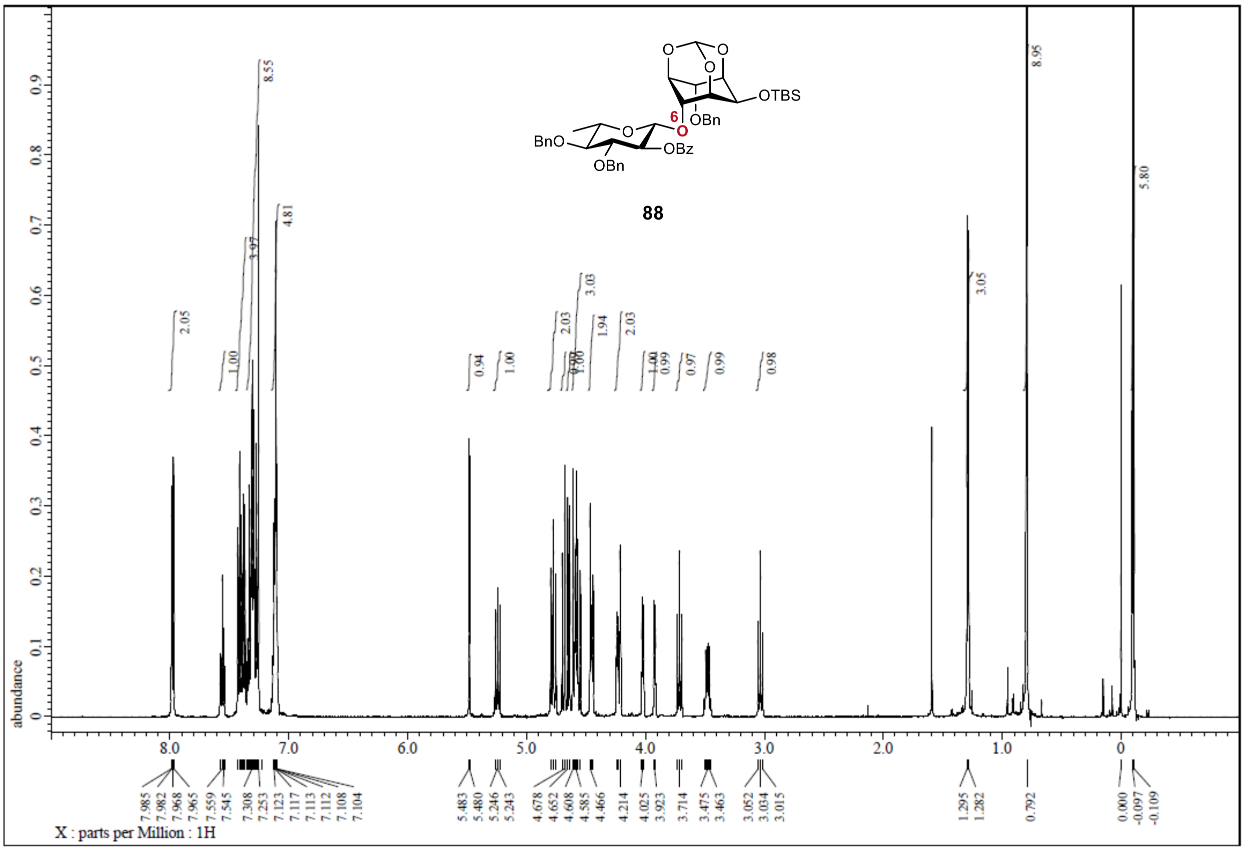

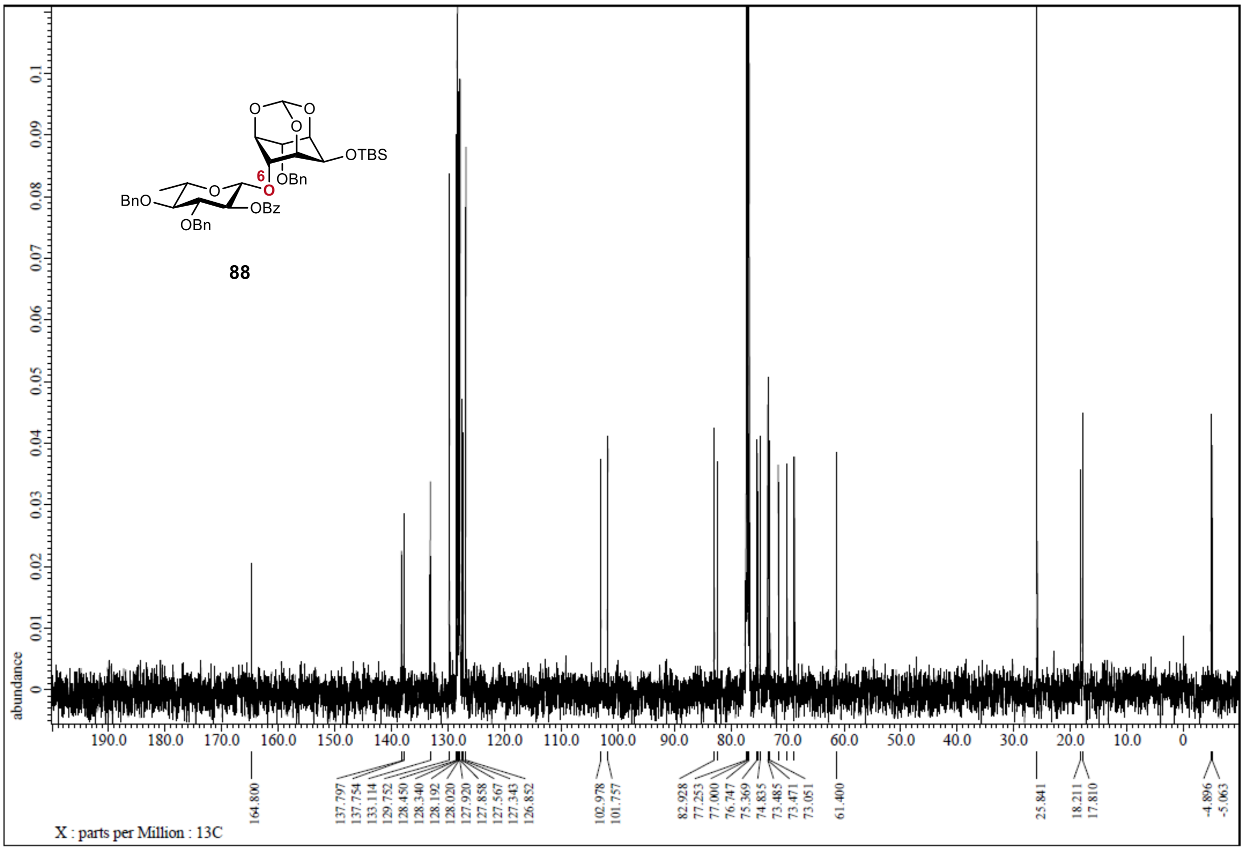

Supplementary Figure 84. ^{1}H and ^{13}C-NMR spectra of compound 88.

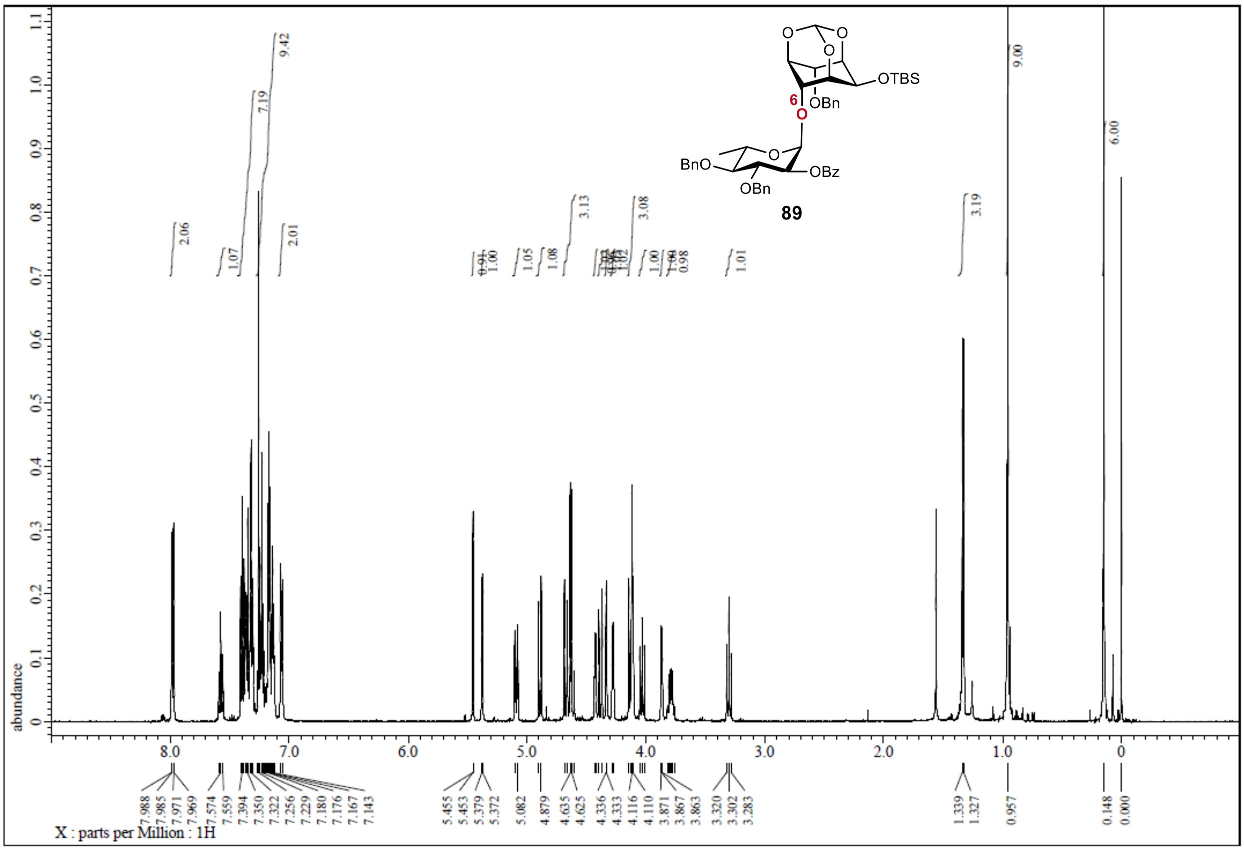

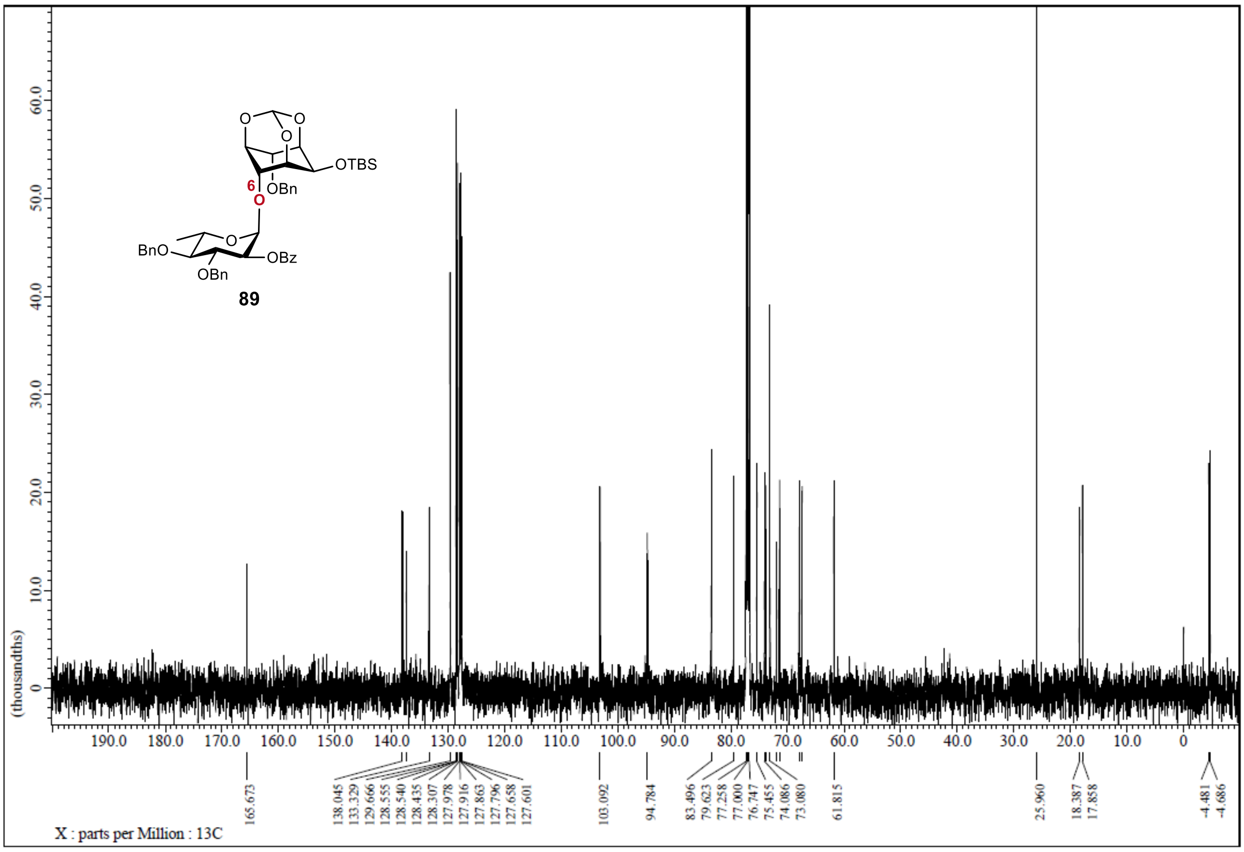

Supplementary Figure 85. ^{1}H and ^{13}C-NMR spectra of compound 89.

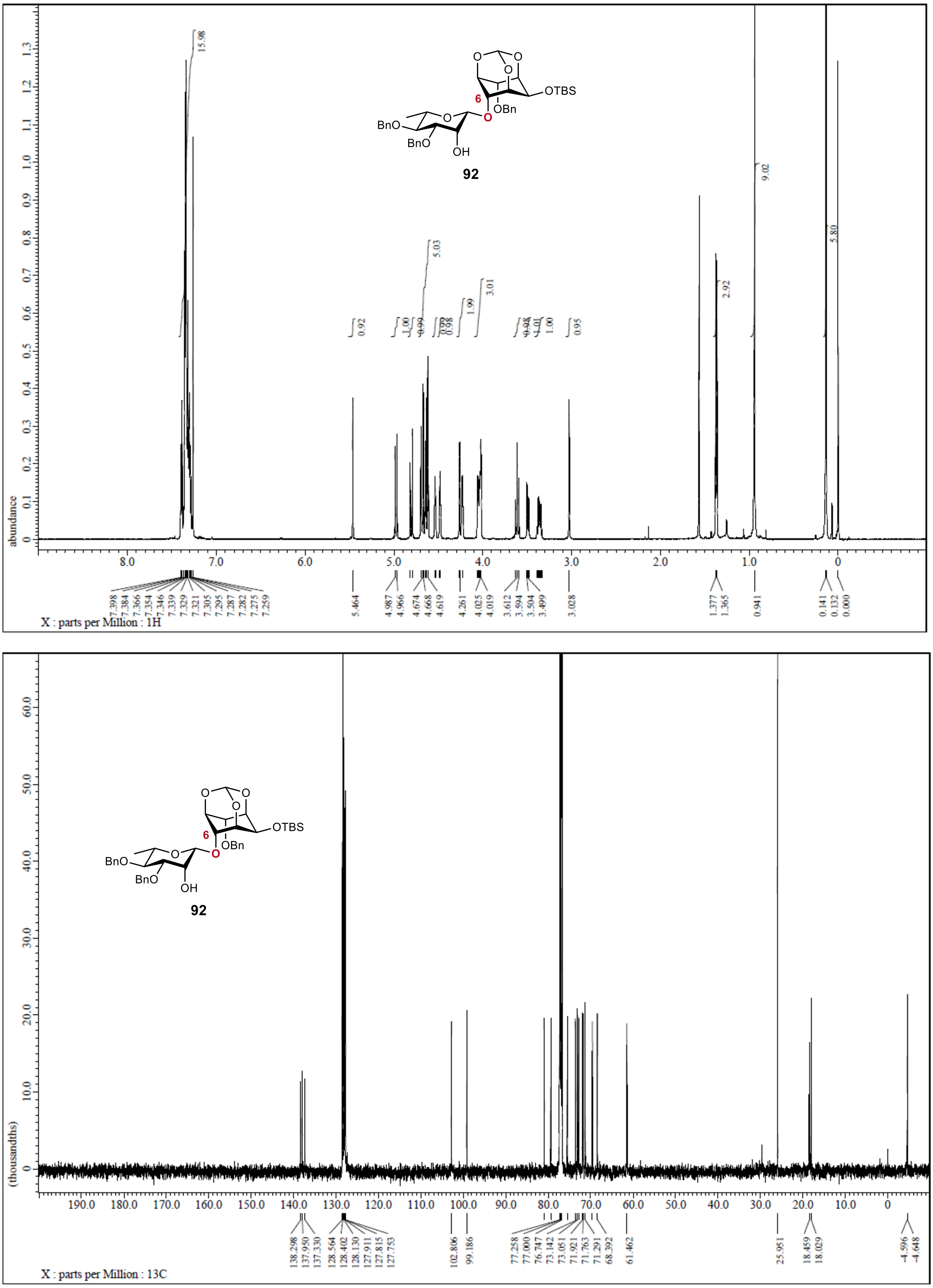

Supplementary Figure 86. ^{1}H and ^{13}C-NMR spectra of compound 92.

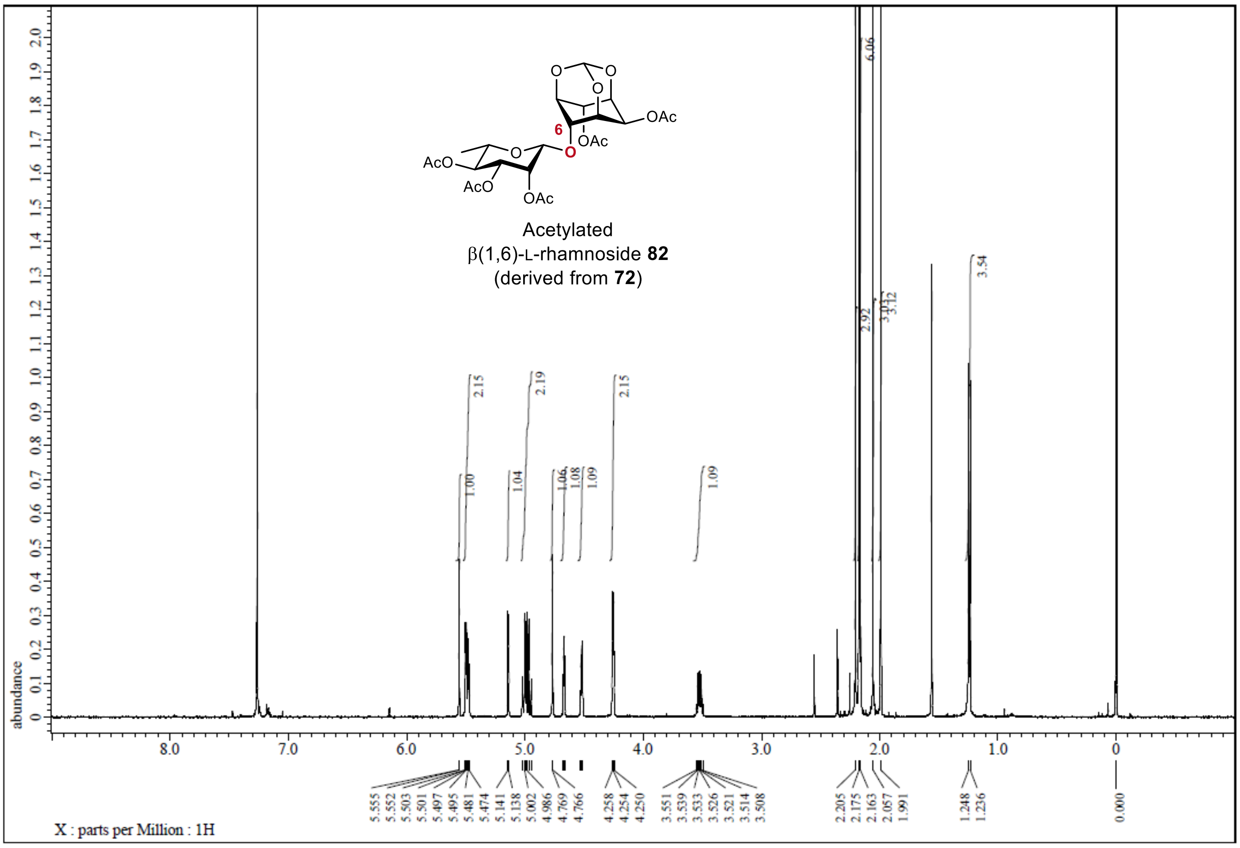

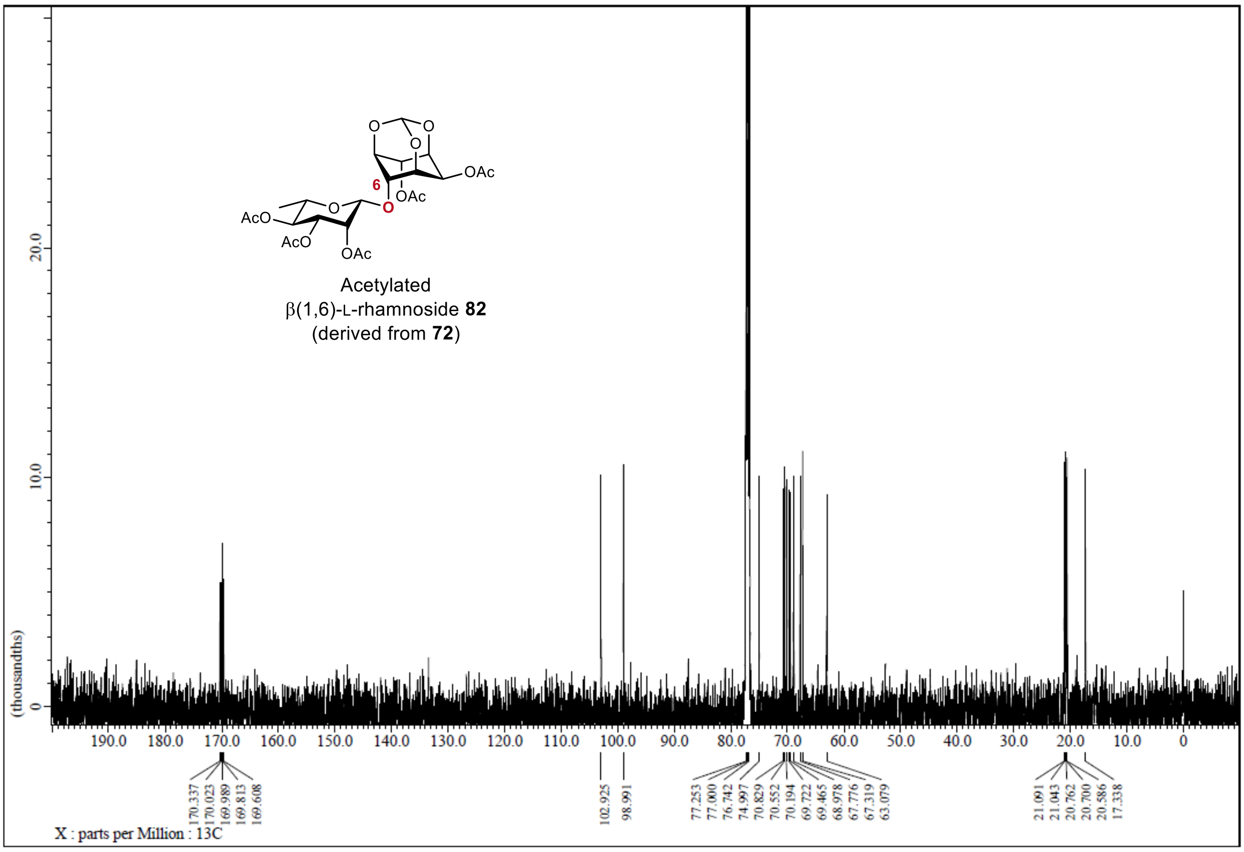

Supplementary Figure 87. ^{1}H and ^{13}C-NMR spectra of compound 82.

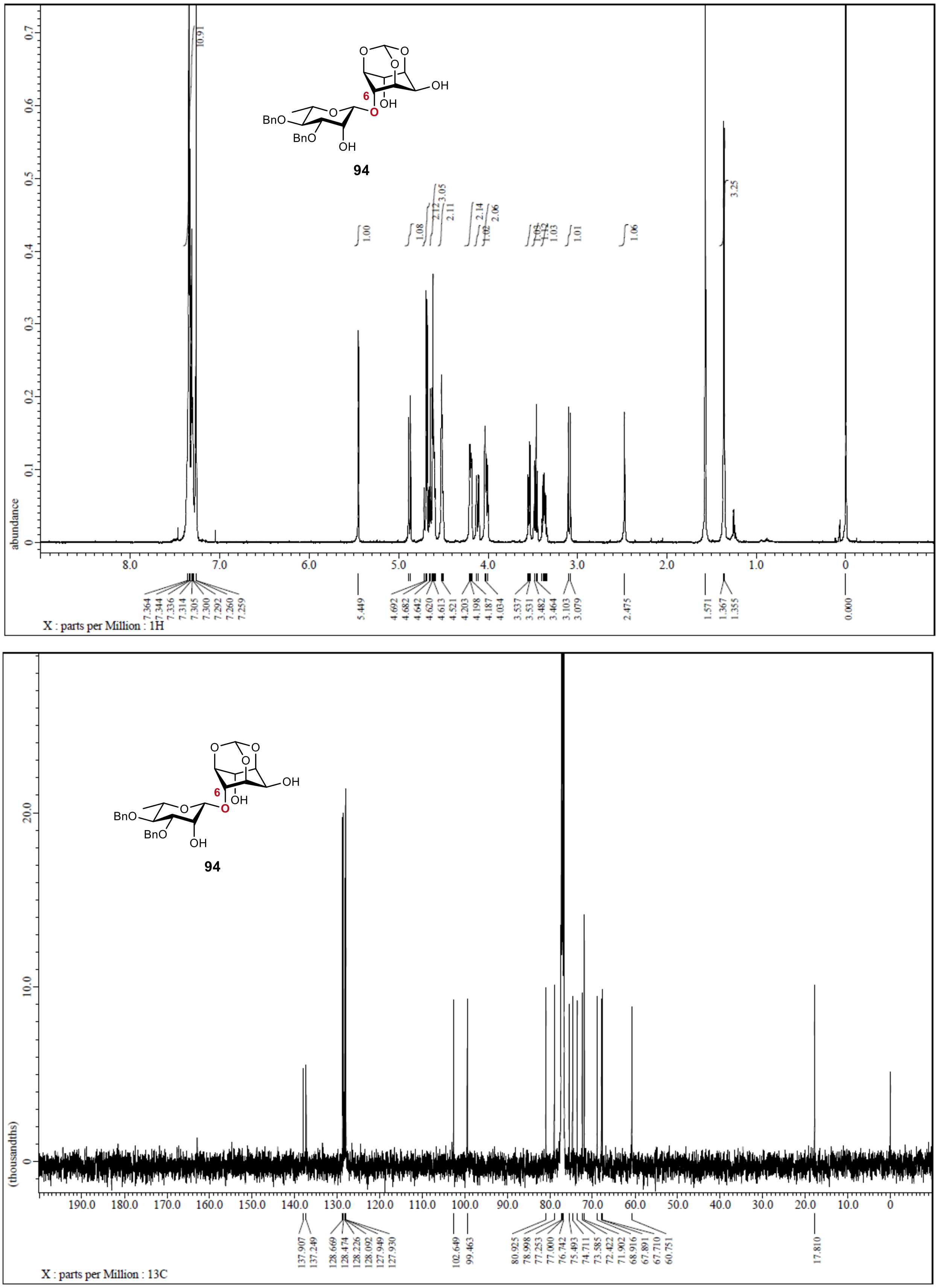

Supplementary Figure 88. ^{1}H and ^{13}C-NMR spectra of compound 94.

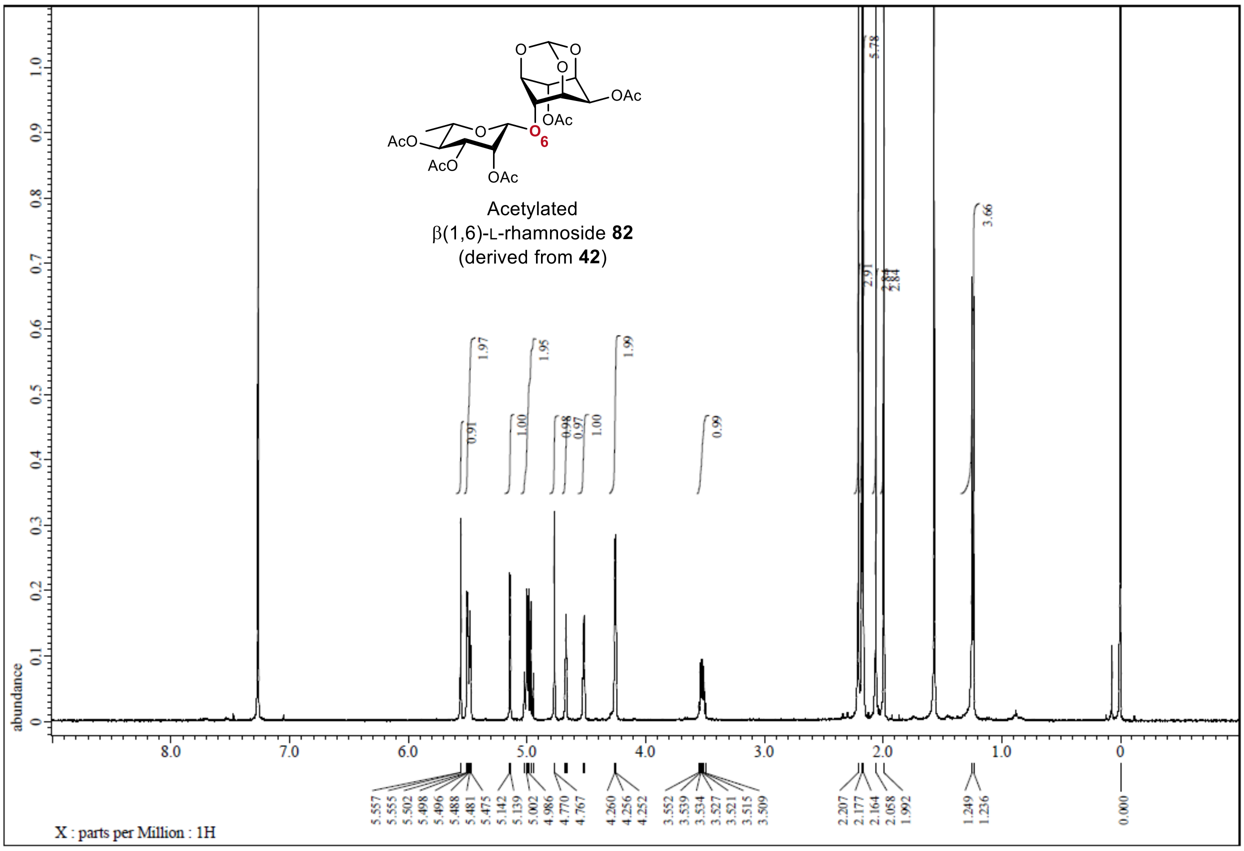

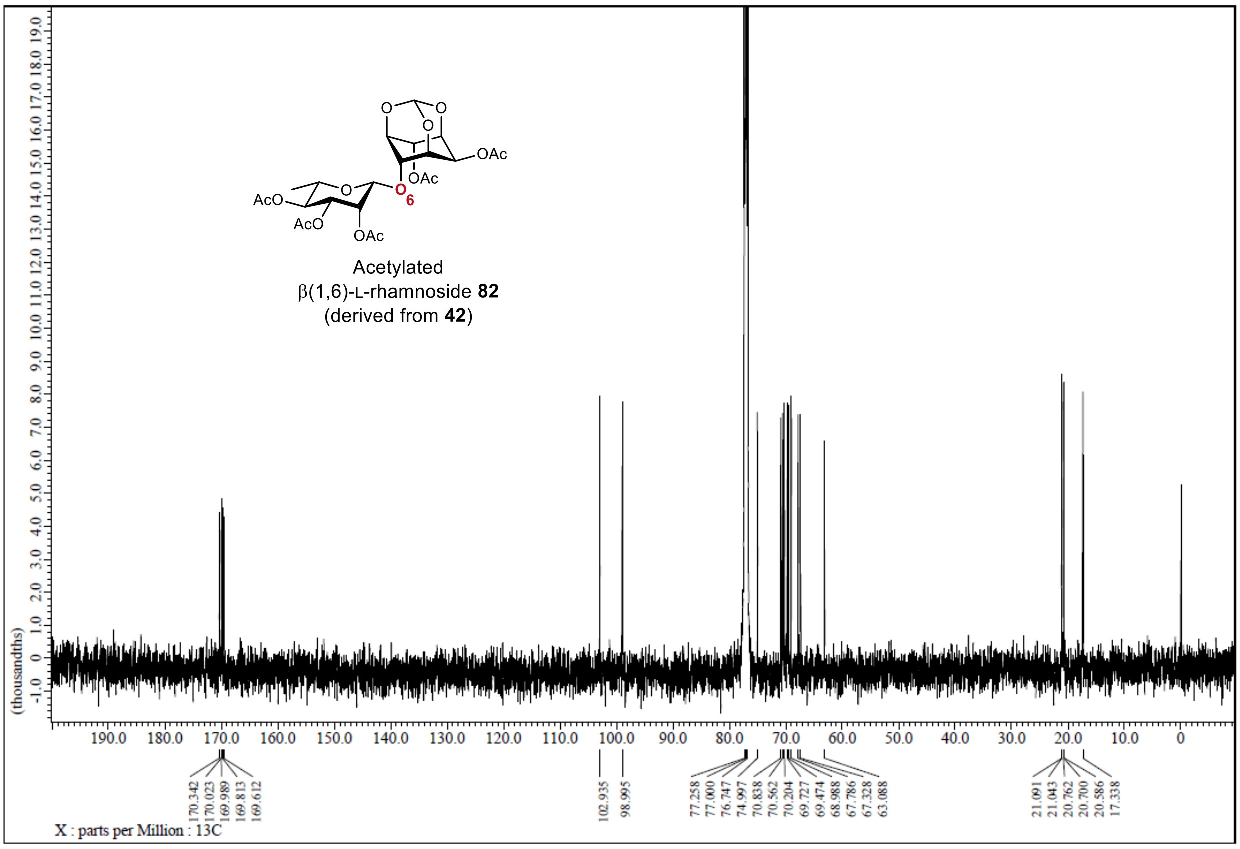

Supplementary Figure 89. ^{1}H and ^{13}C-NMR spectra of compound 82.

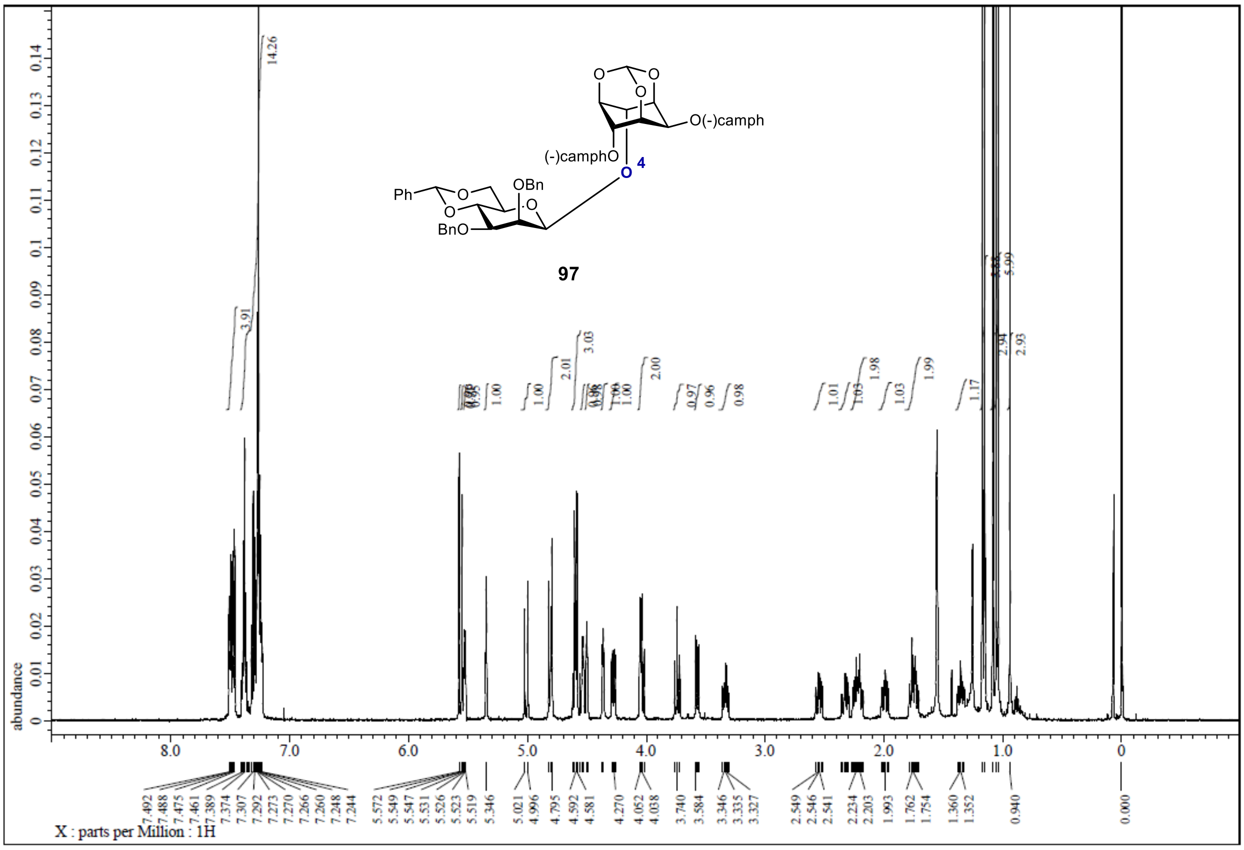

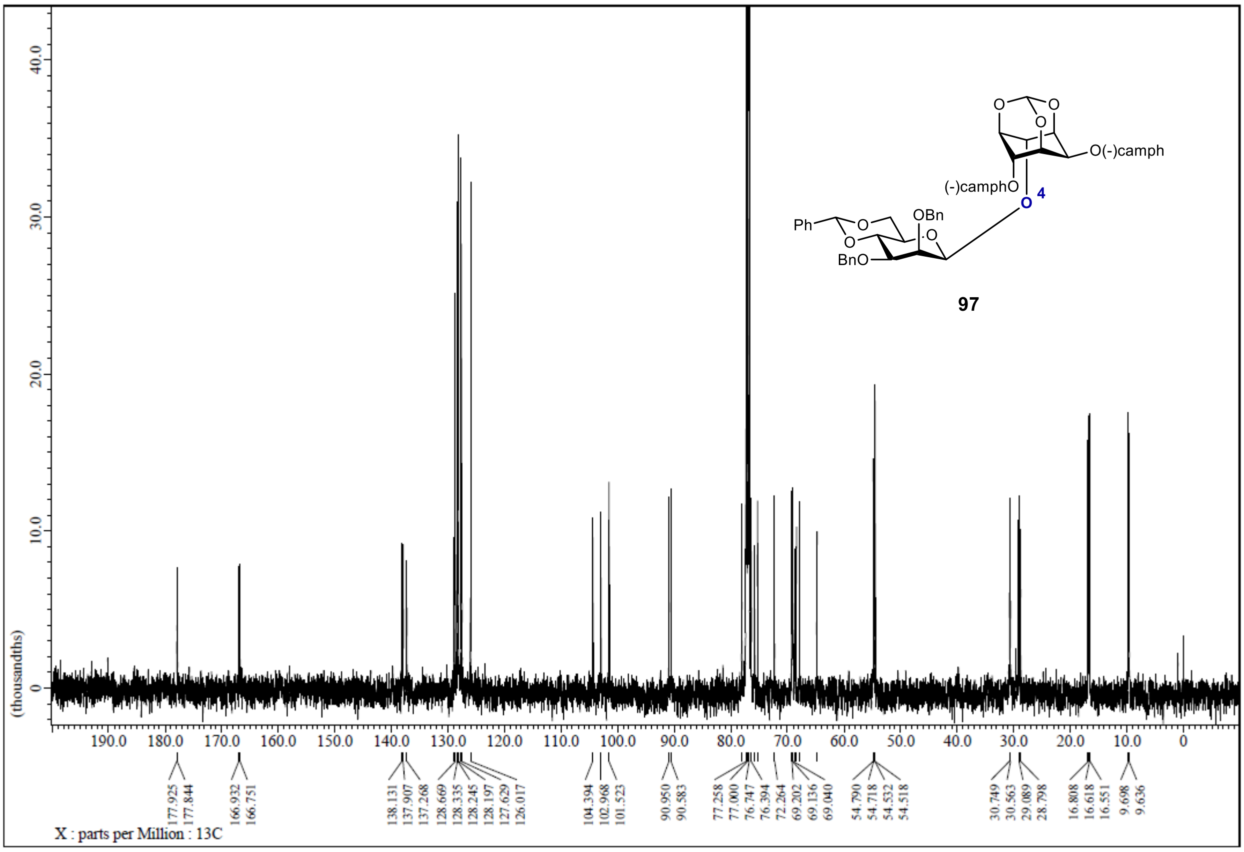

Supplementary Figure 90. ^{1}H and ^{13}C-NMR spectra of compound 97.

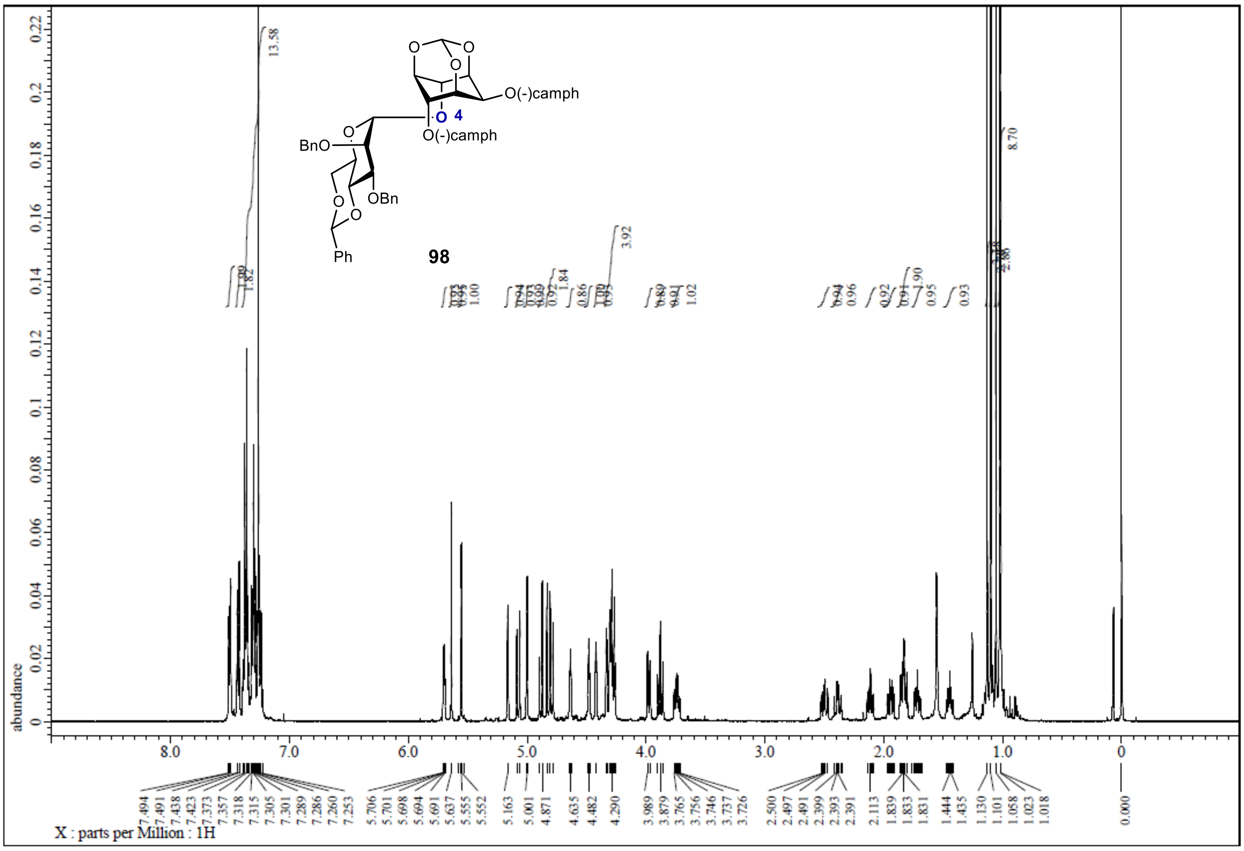

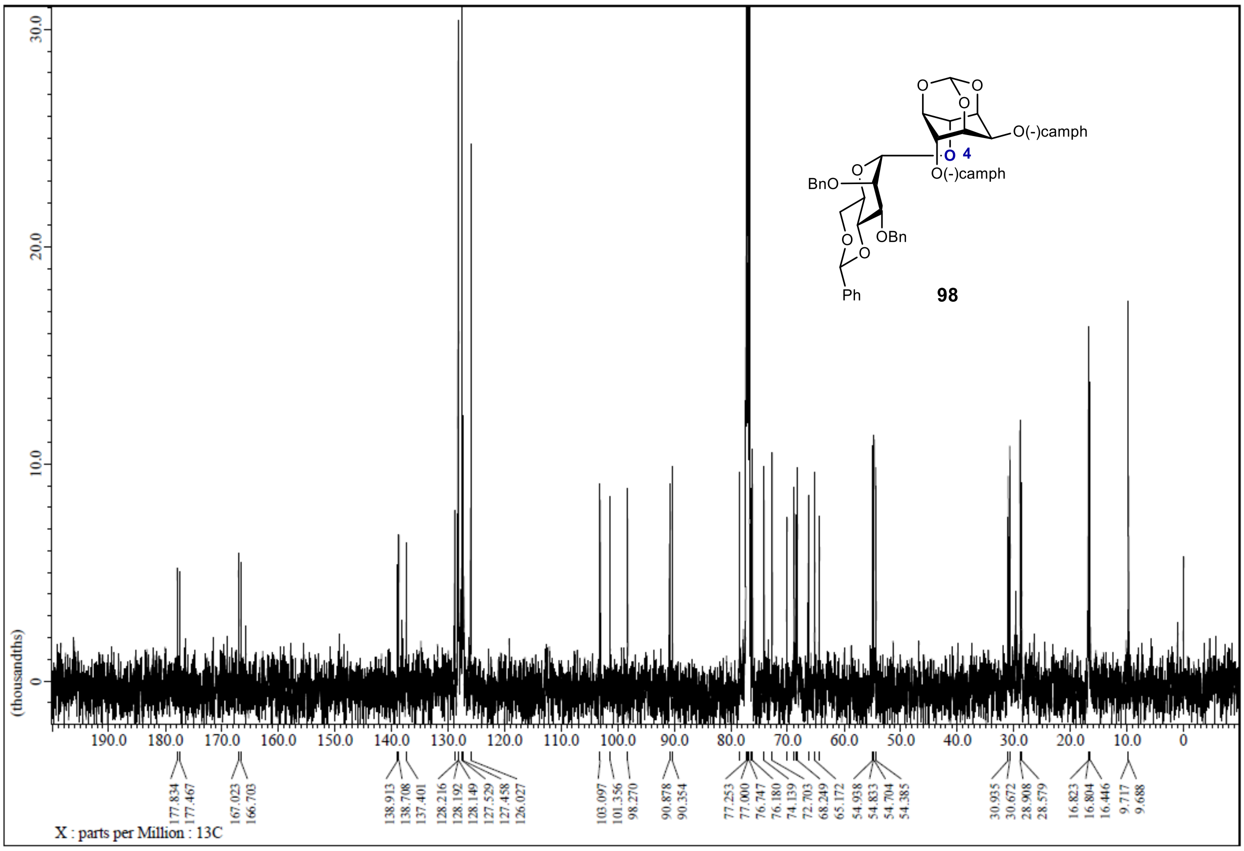

Supplementary Figure 91. ^{1}H and ^{13}C-NMR spectra of compound 98.

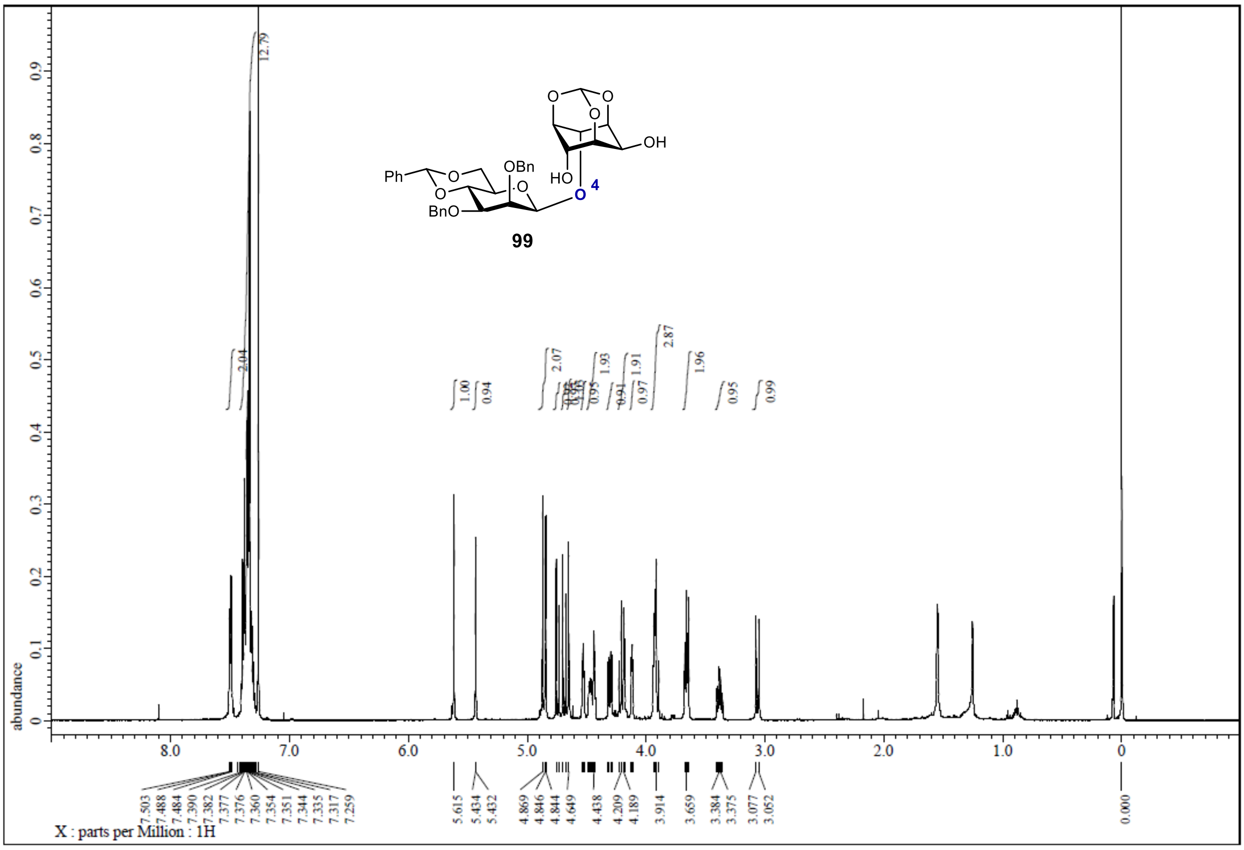

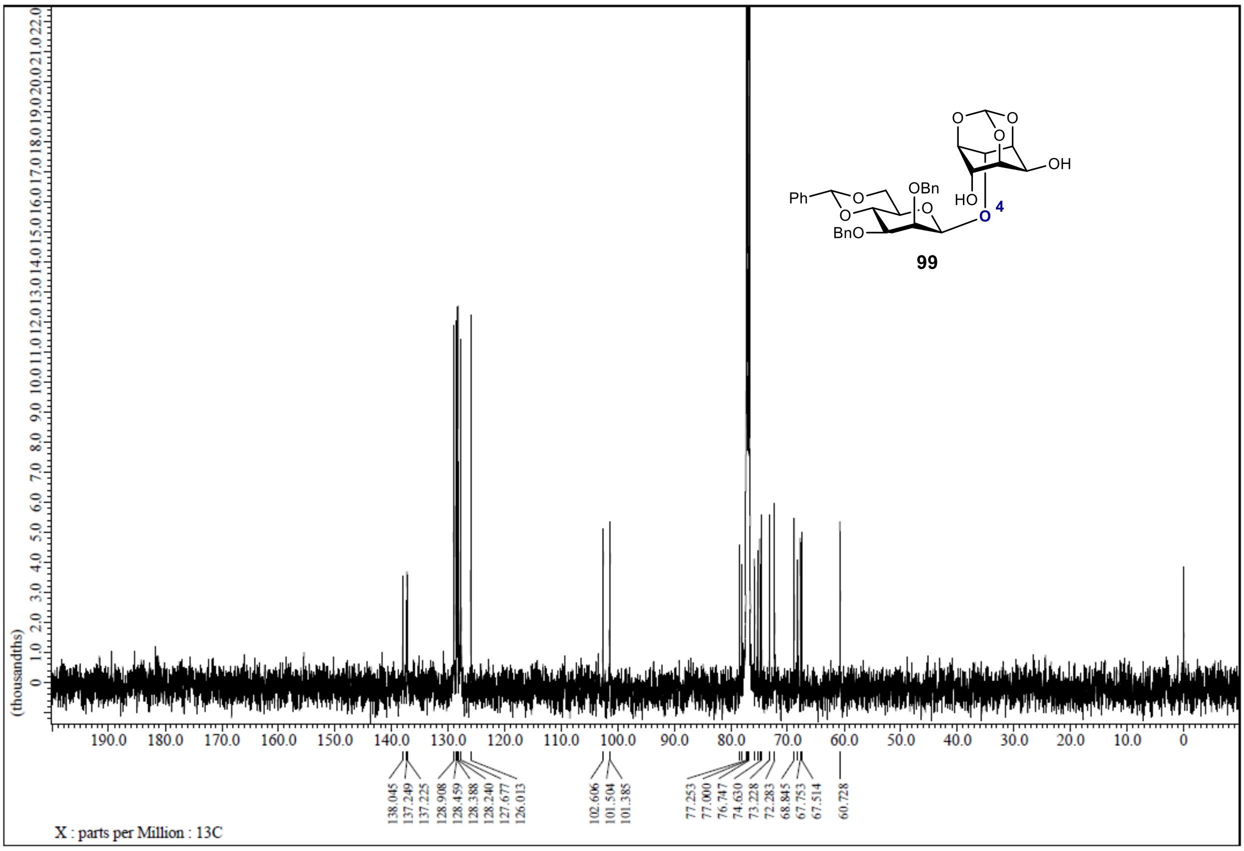

Supplementary Figure 92. ^{1}H and ^{13}C-NMR spectra of compound 99.

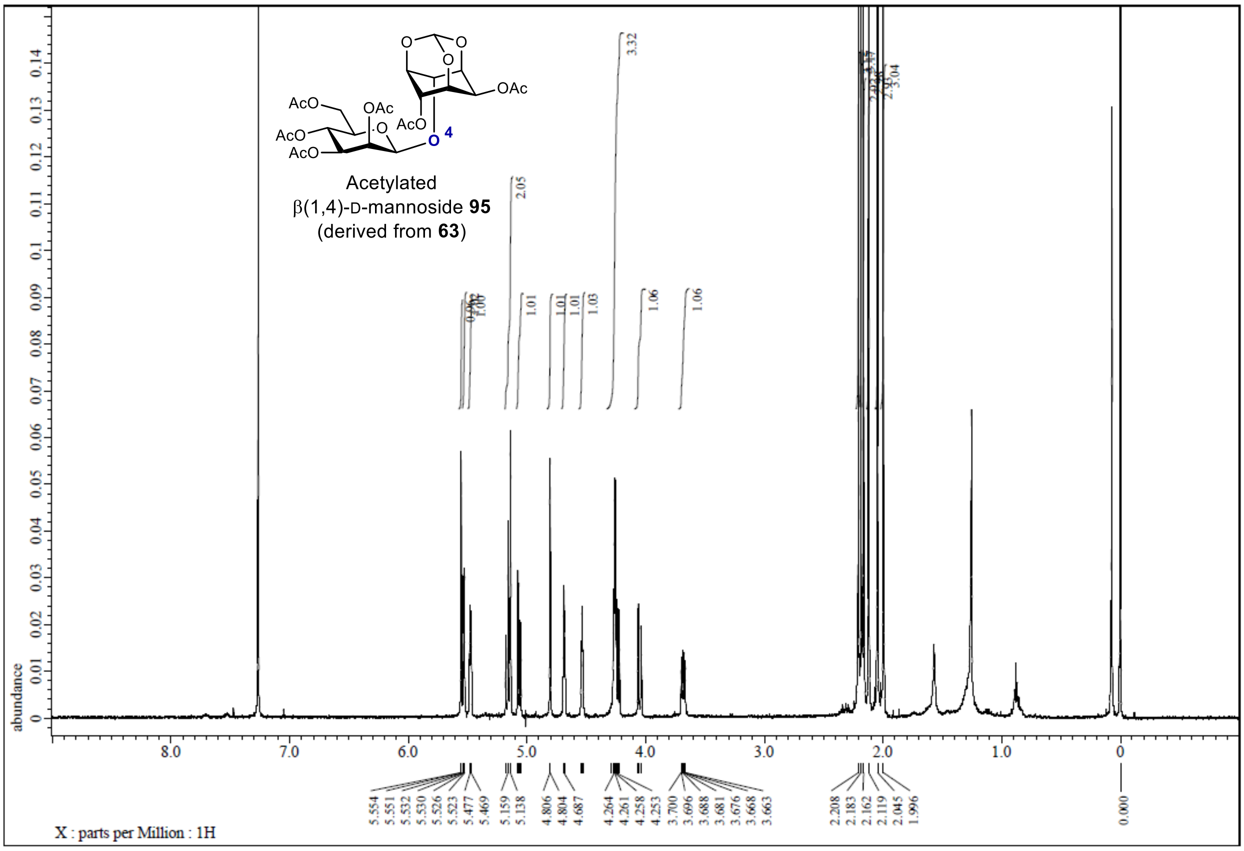

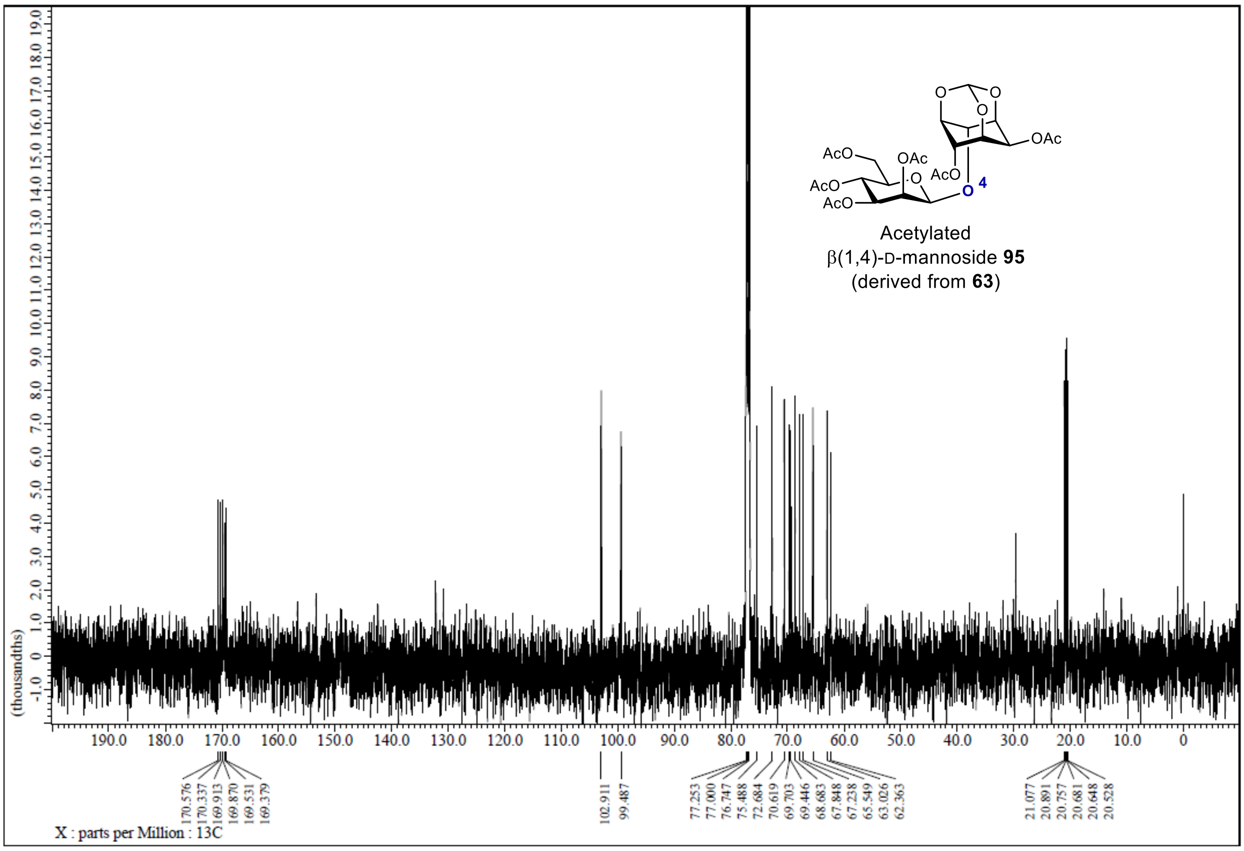

Supplementary Figure 93. ^{1}H and ^{13}C-NMR spectra of compound 95.

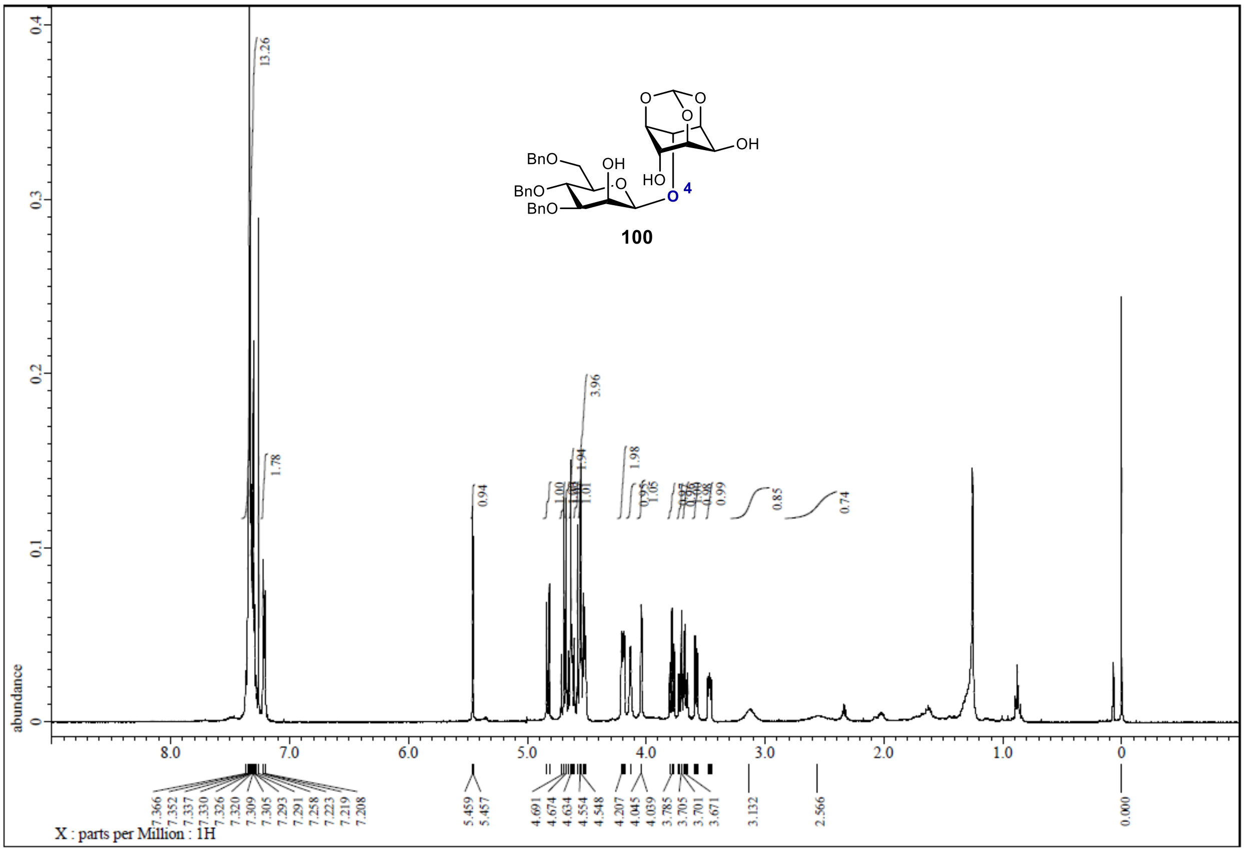

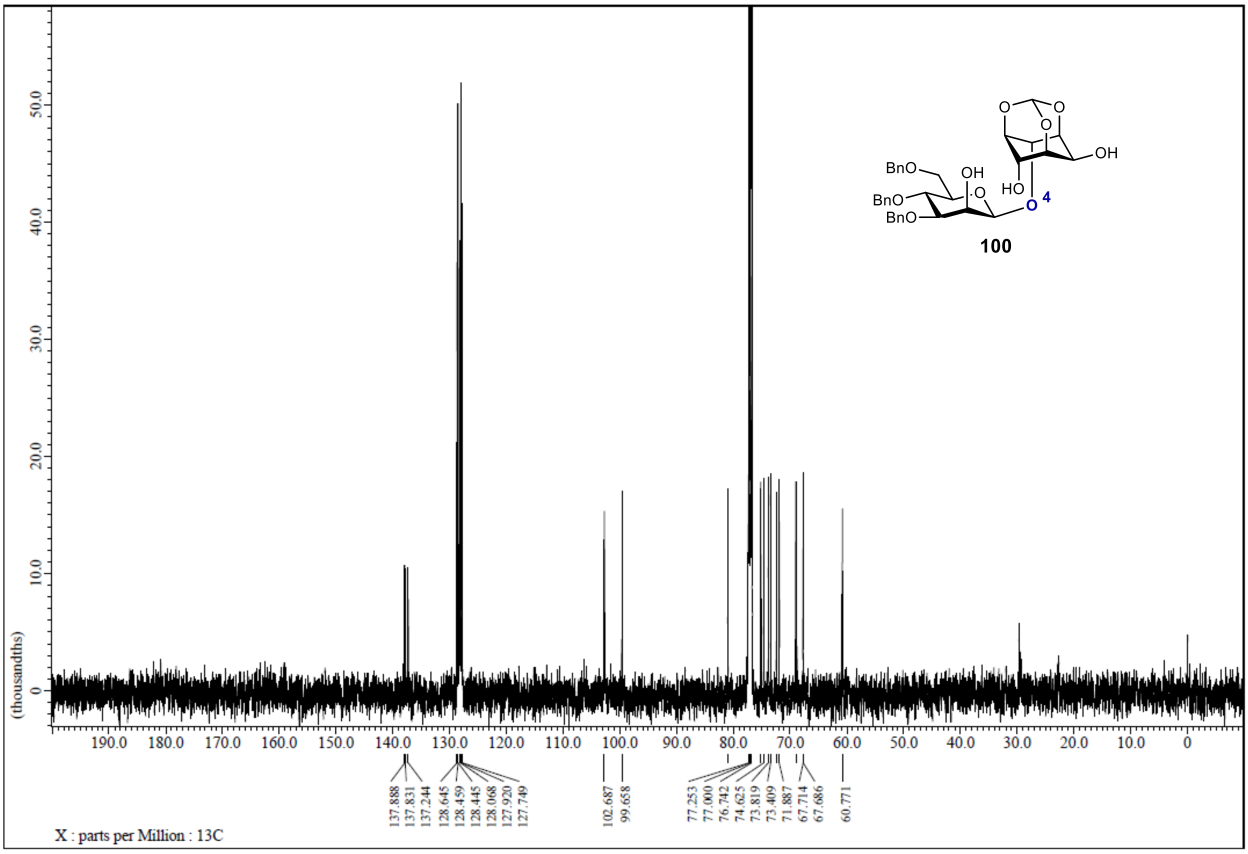

Supplementary Figure 94. ^{1}H and ^{13}C-NMR spectra of compound 100.

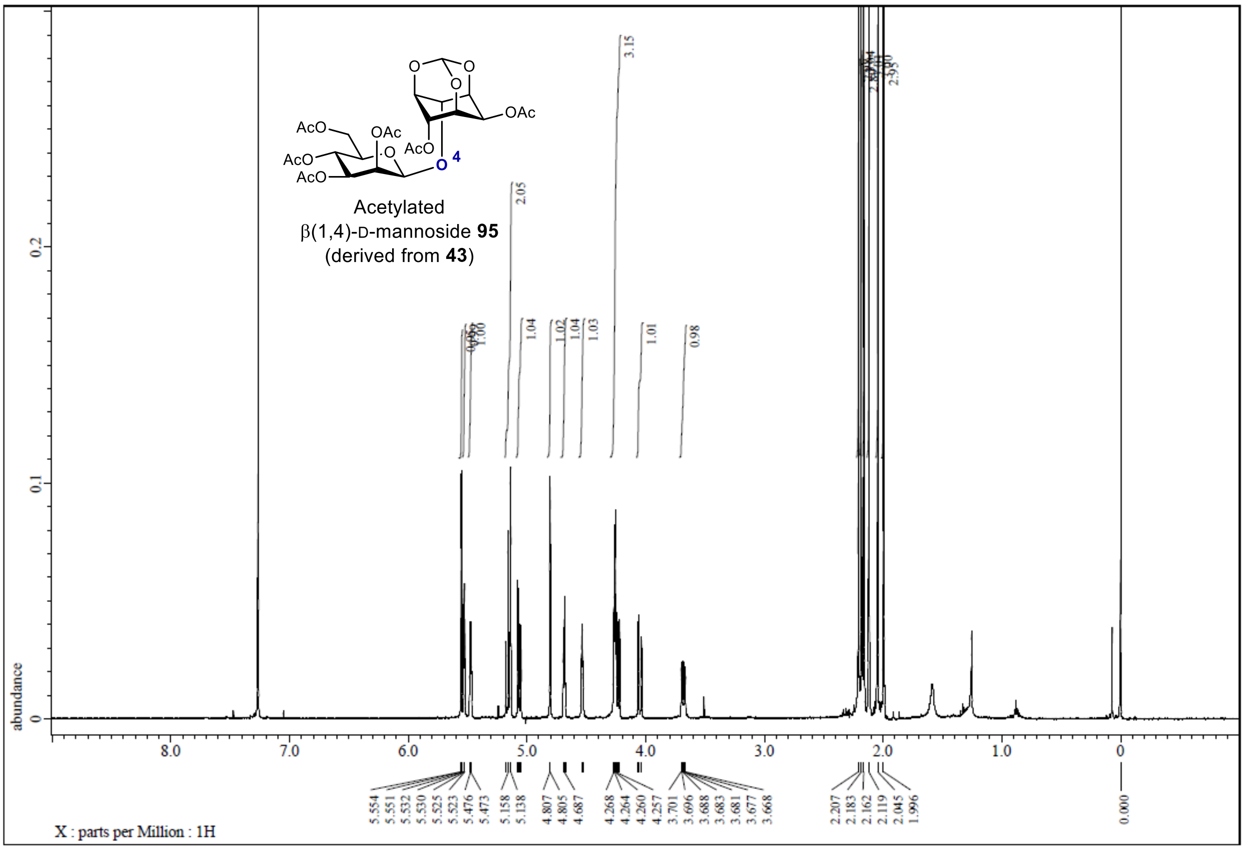

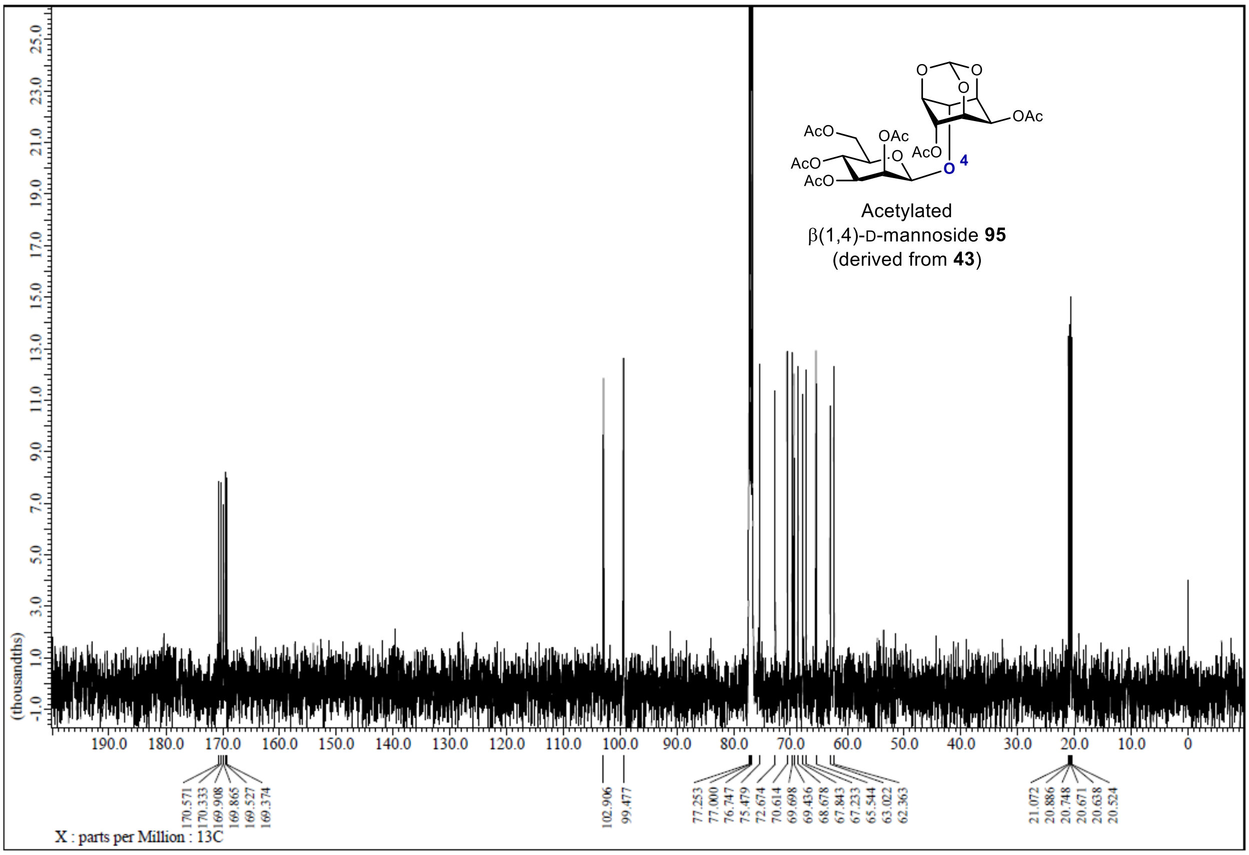

Supplementary Figure 95. ^{1}H and ^{13}C-NMR spectra of compound 95.

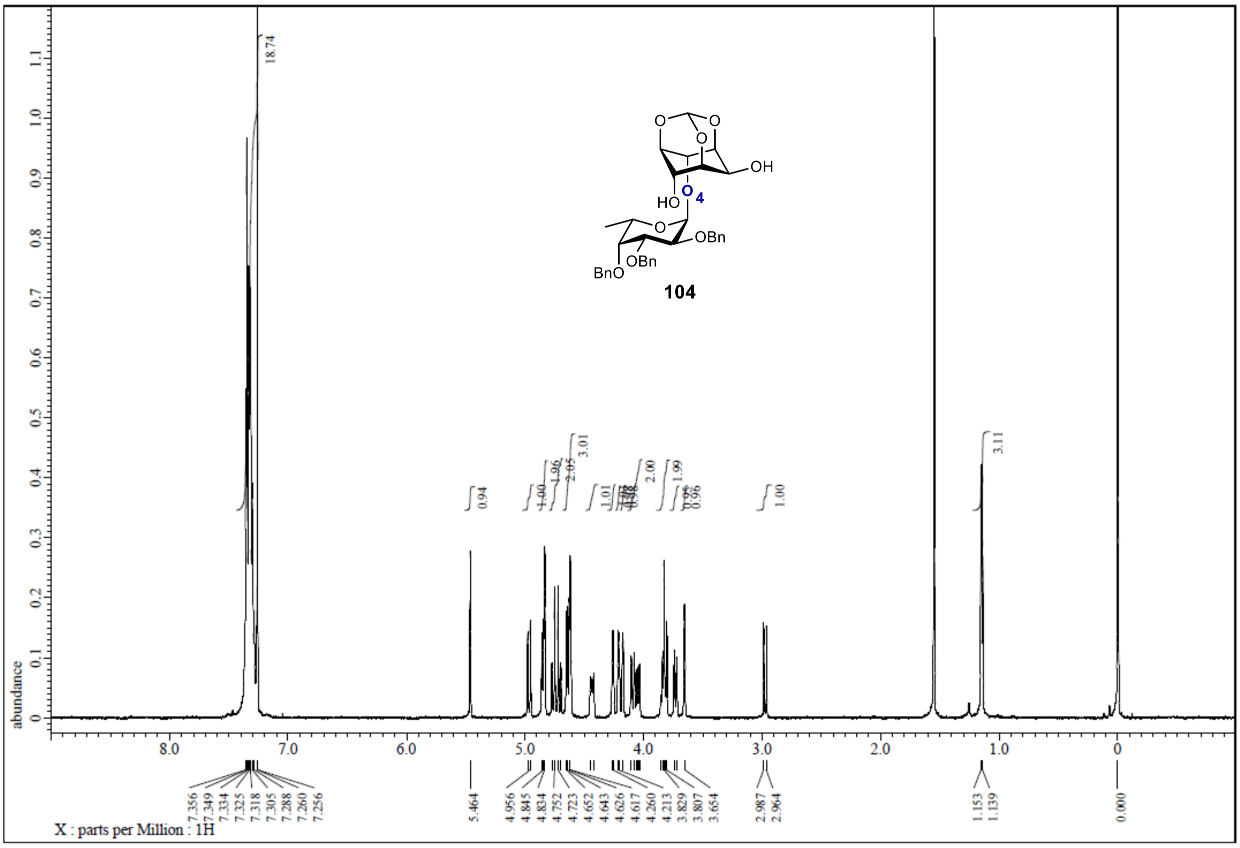

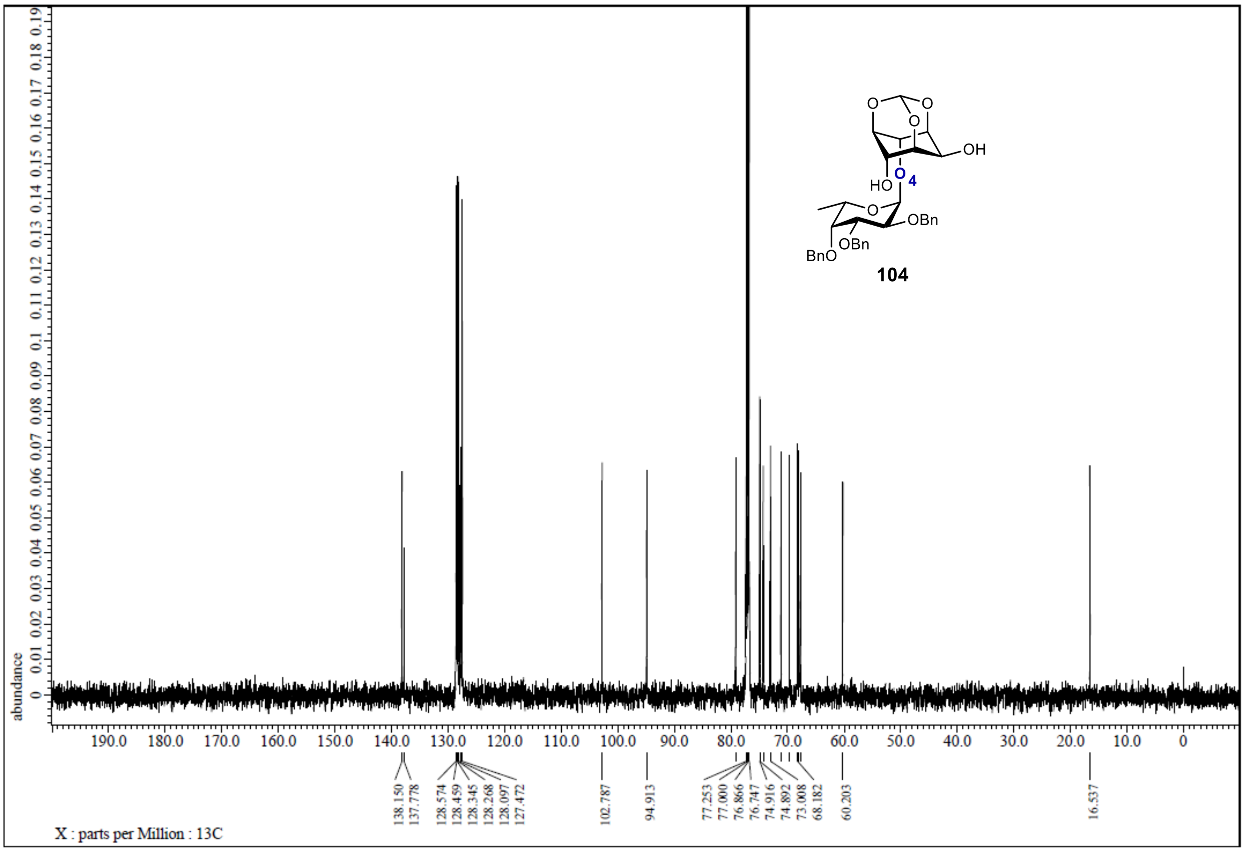

Supplementary Figure 96. ^{1}H and ^{13}C-NMR spectra of compound 104.

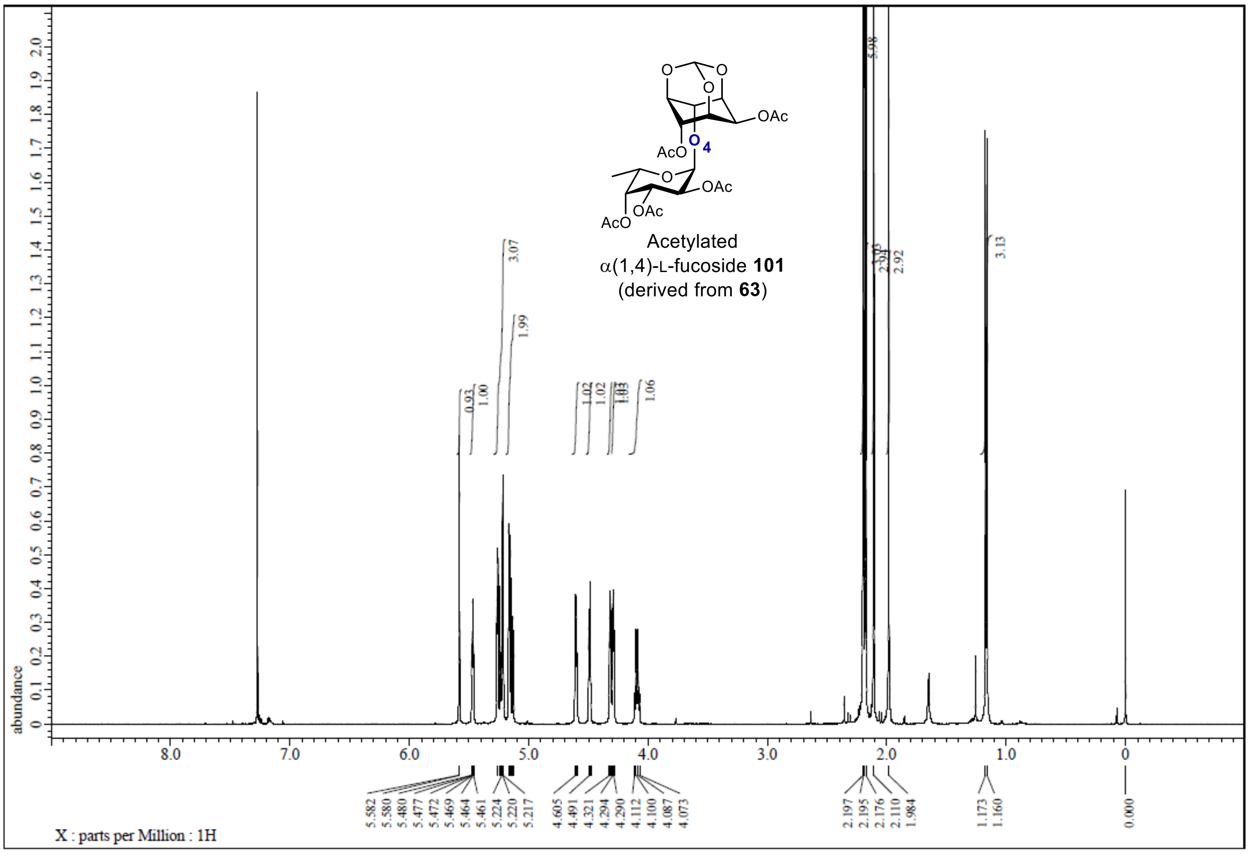

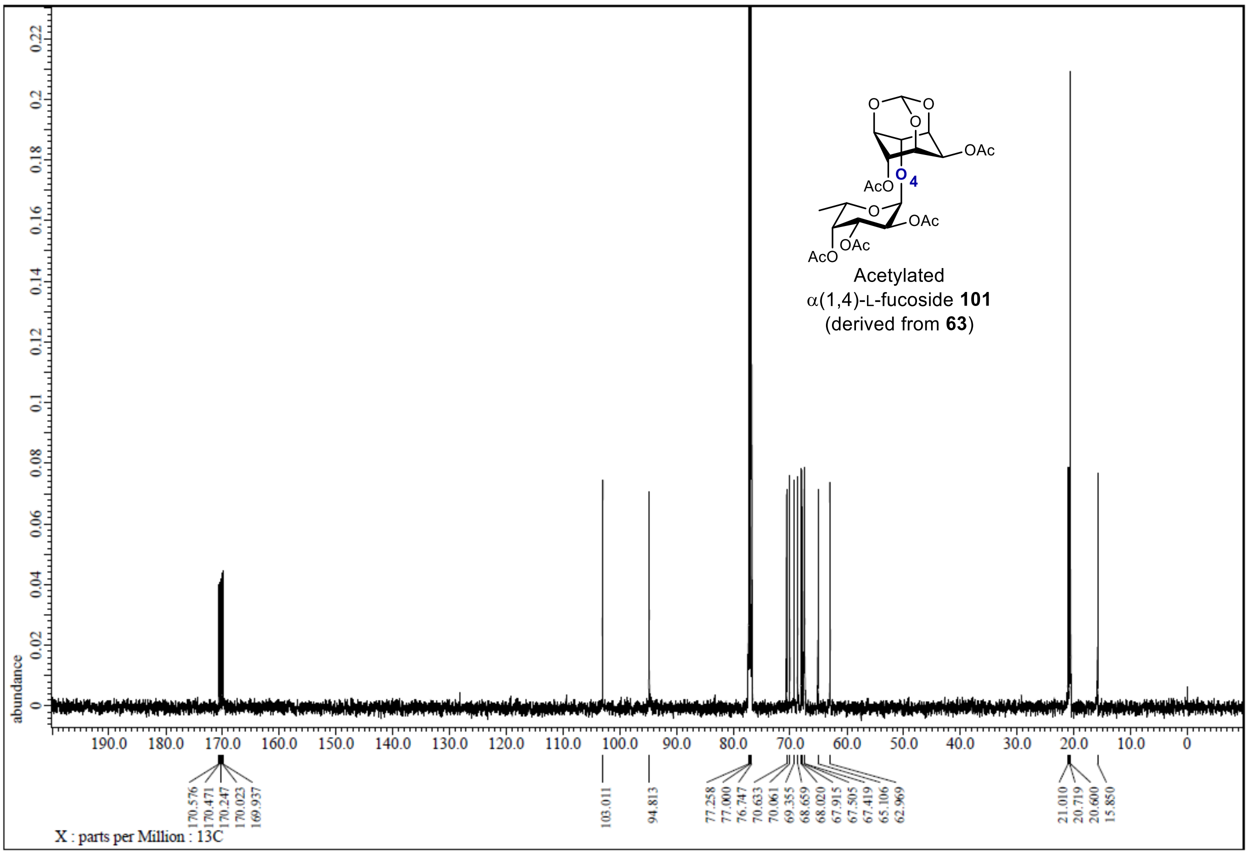

Supplementary Figure 97. ^{1}H and ^{13}C-NMR spectra of compound 101.

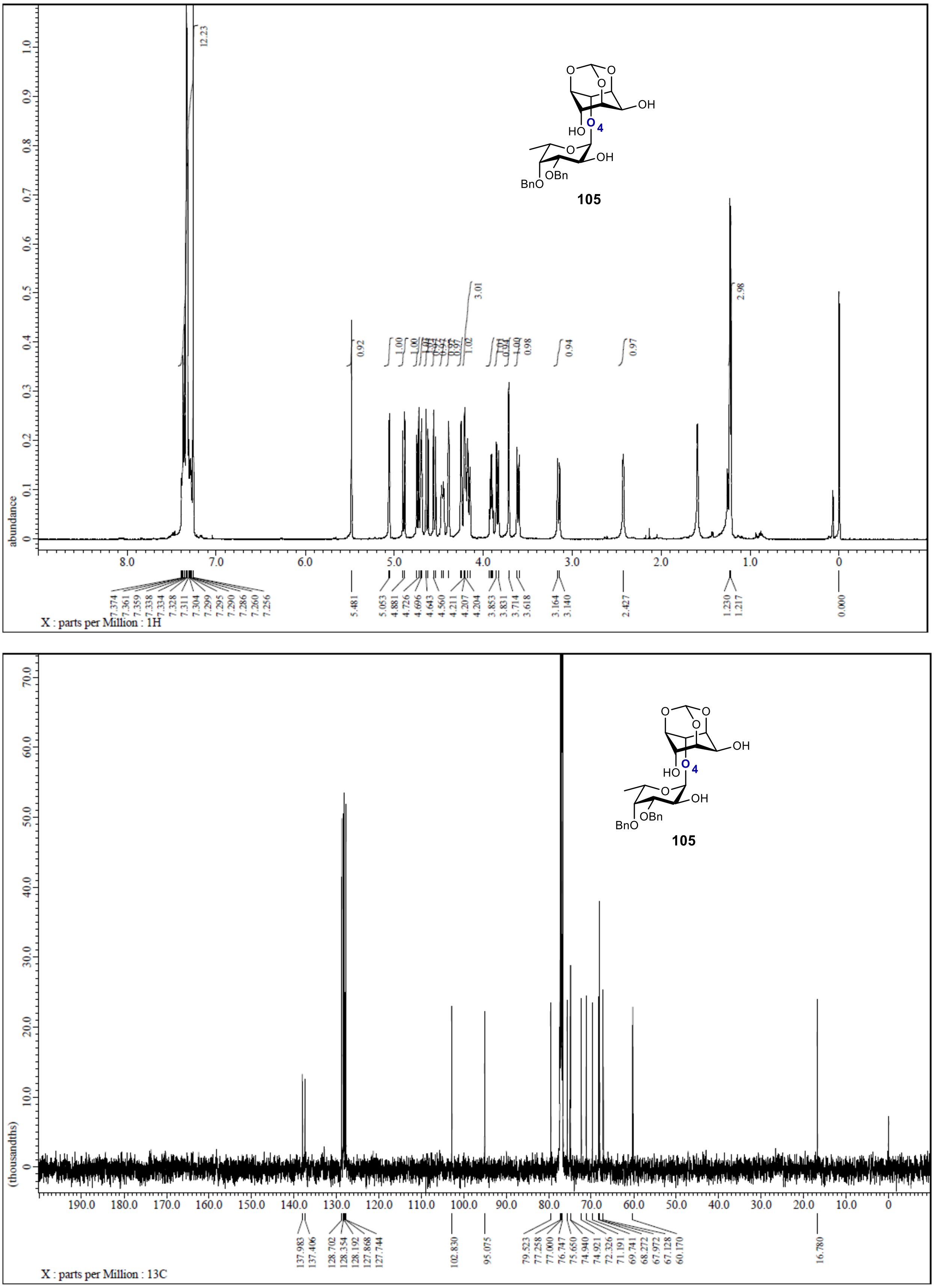

Supplementary Figure 98. ^{1}H and ^{13}C-NMR spectra of compound 105.

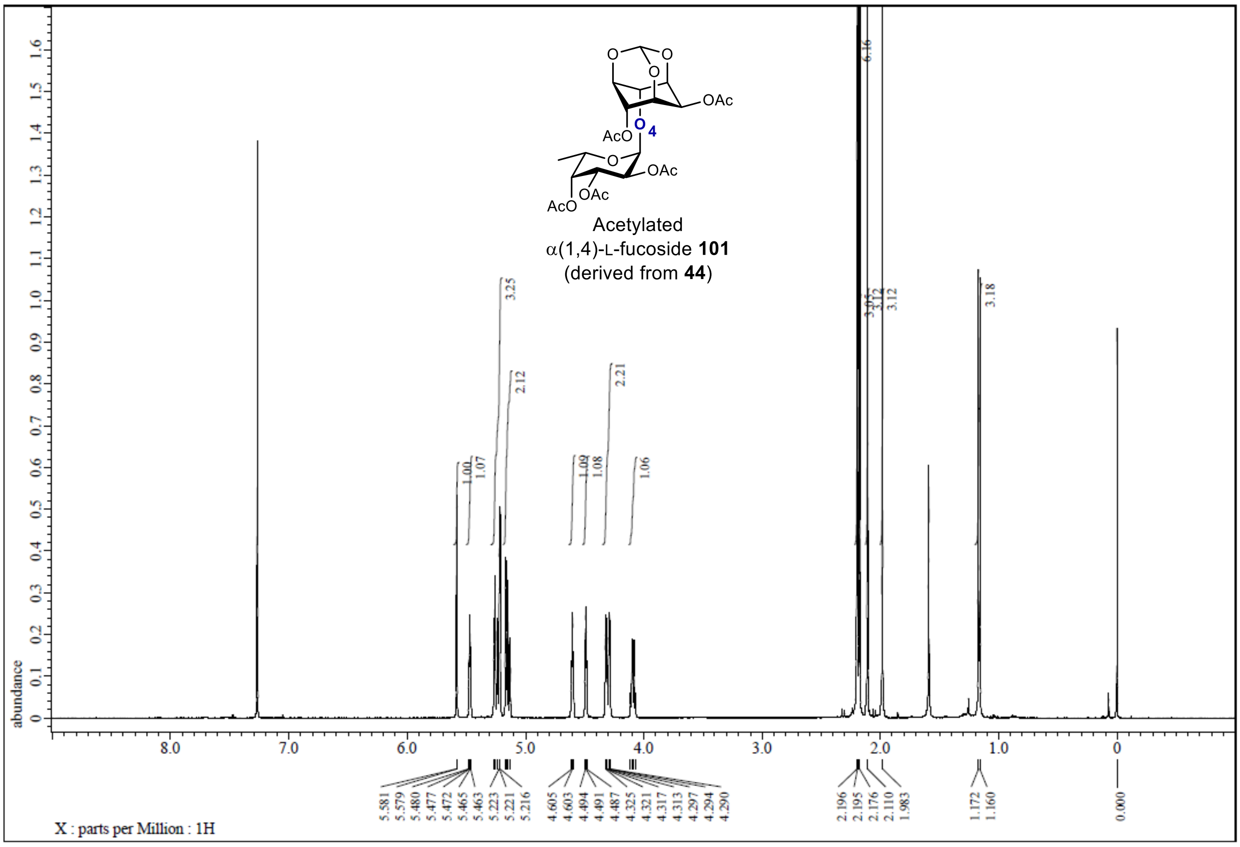

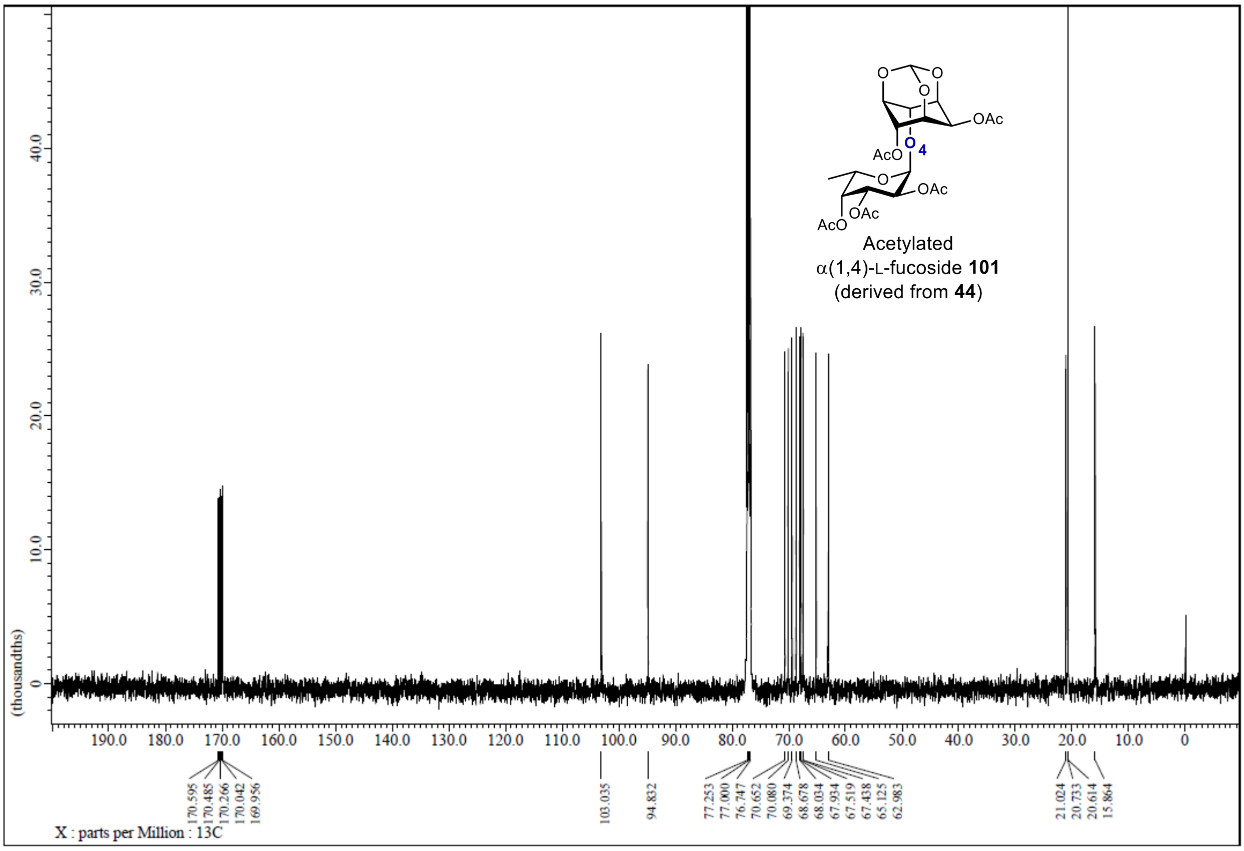

Supplementary Figure 99. ¹H and ¹³C-NMR spectra of compound 101.

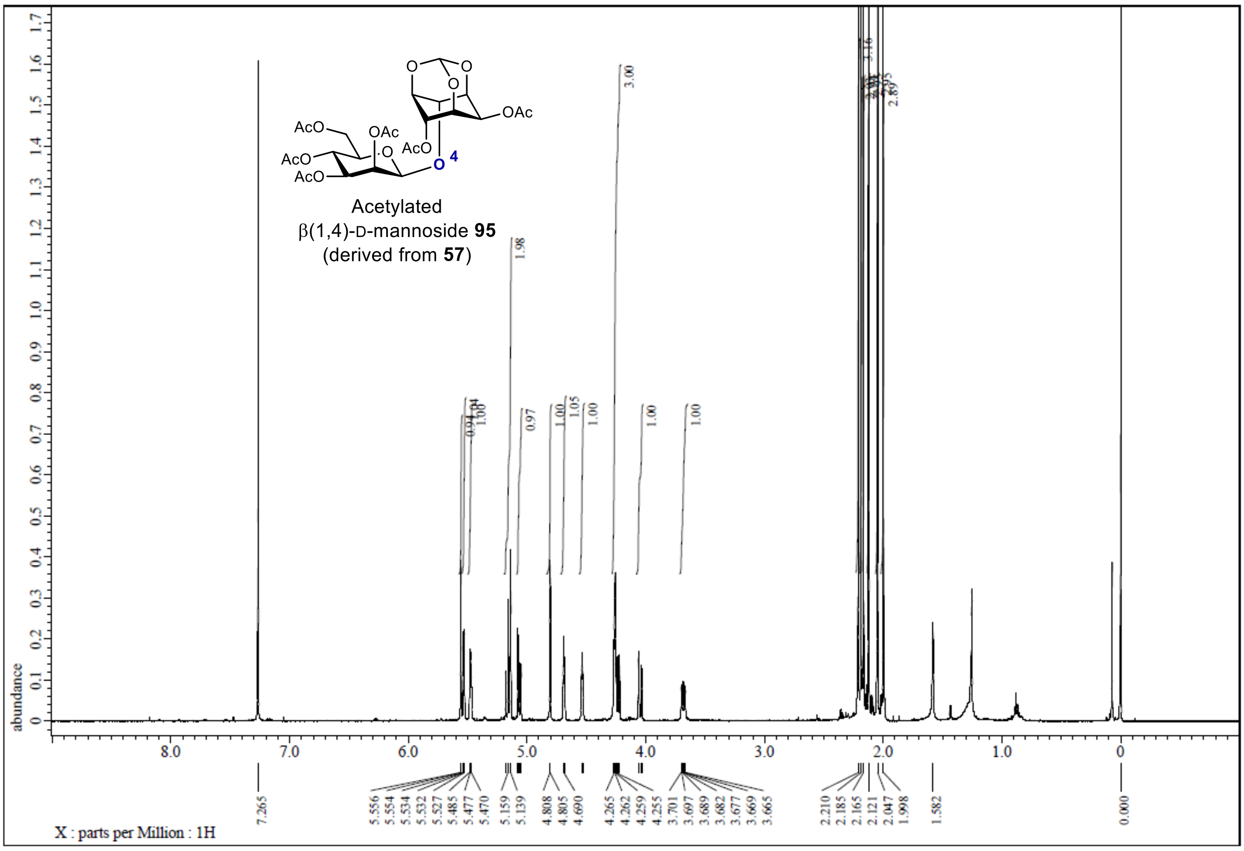

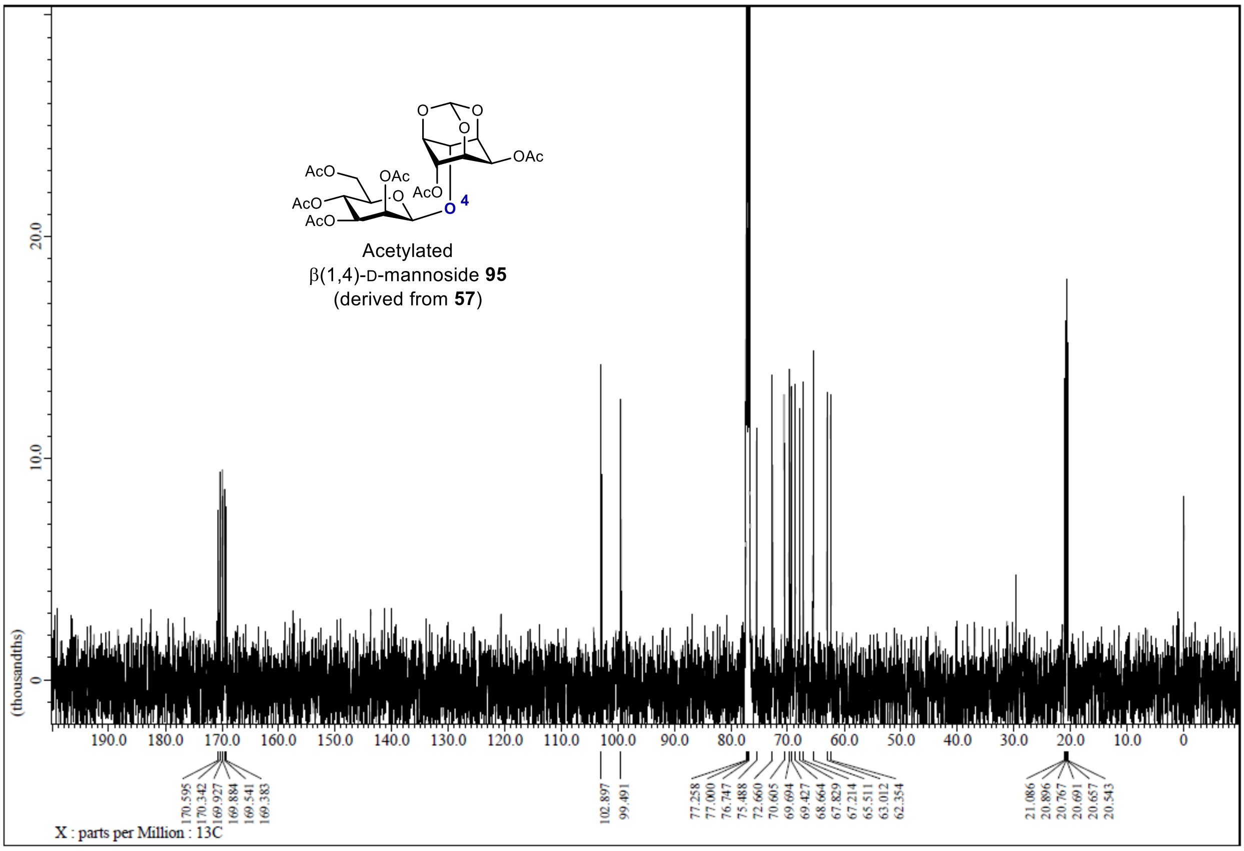

Supplementary Figure 100. ^{1}H and ^{13}C-NMR spectra of compound 95.

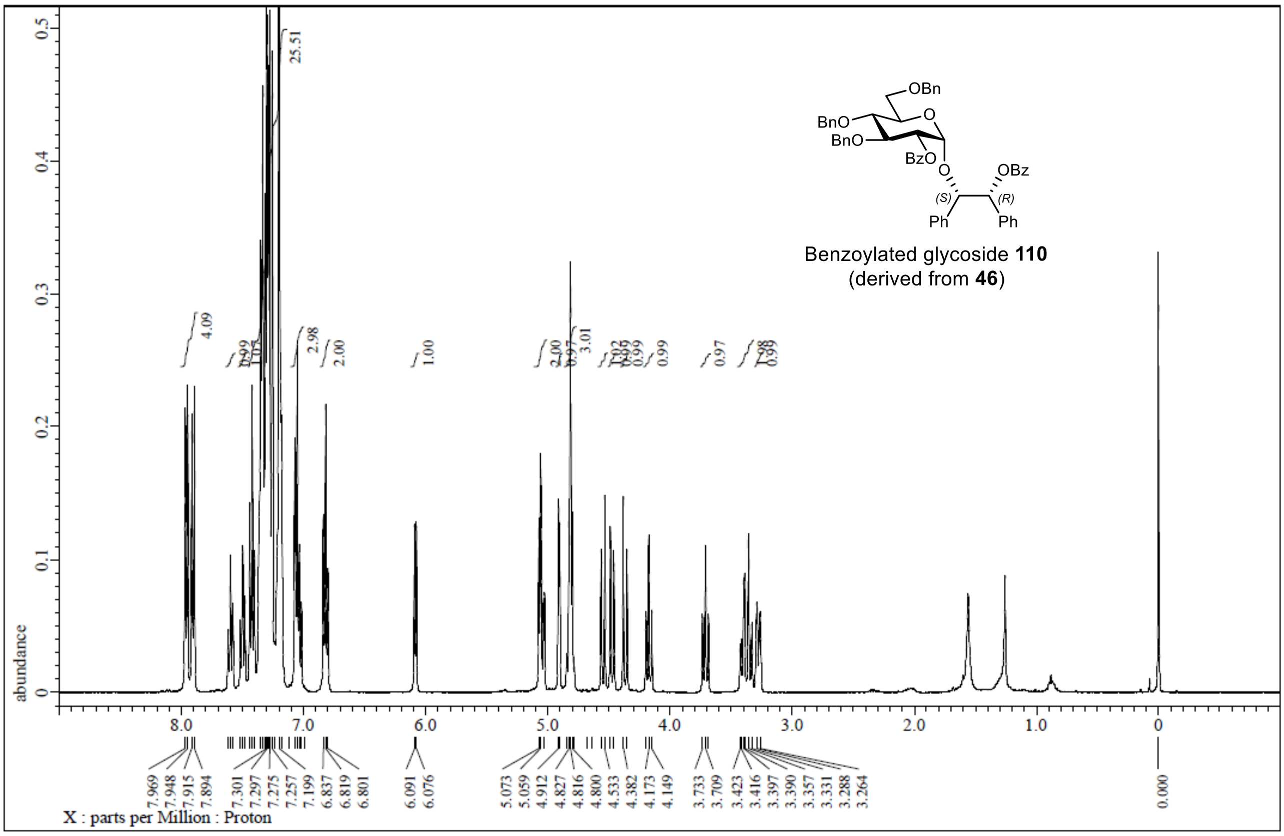

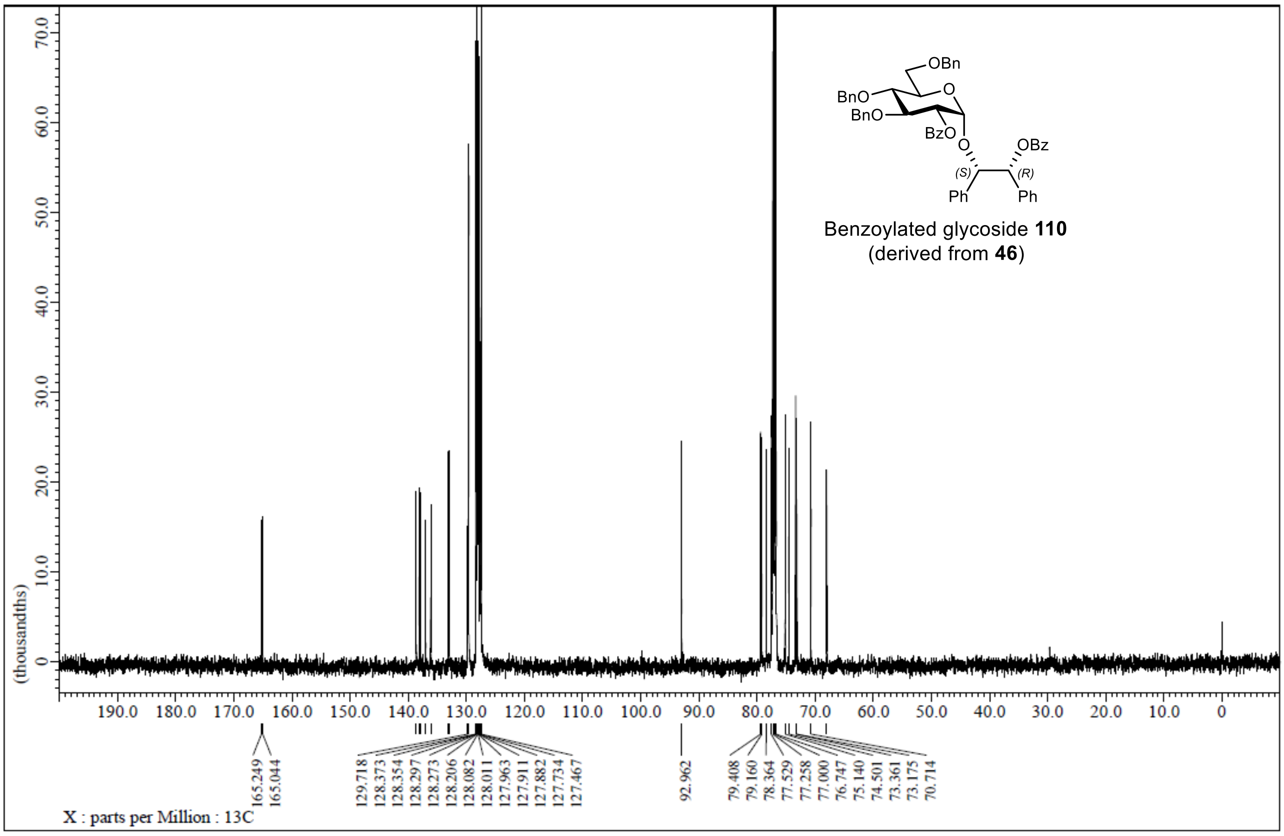

Supplementary Figure 101. ^{1}H and ^{13}C-NMR spectra of compound 110.

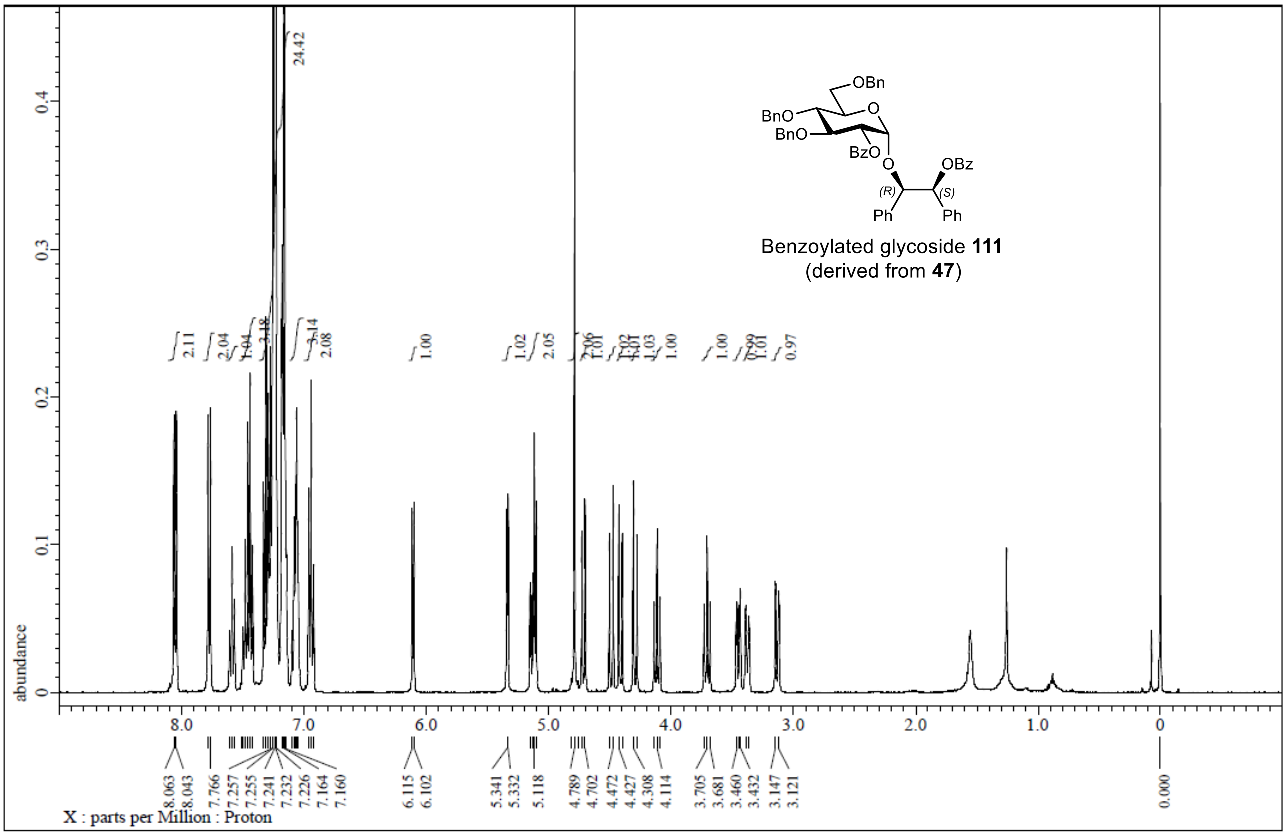

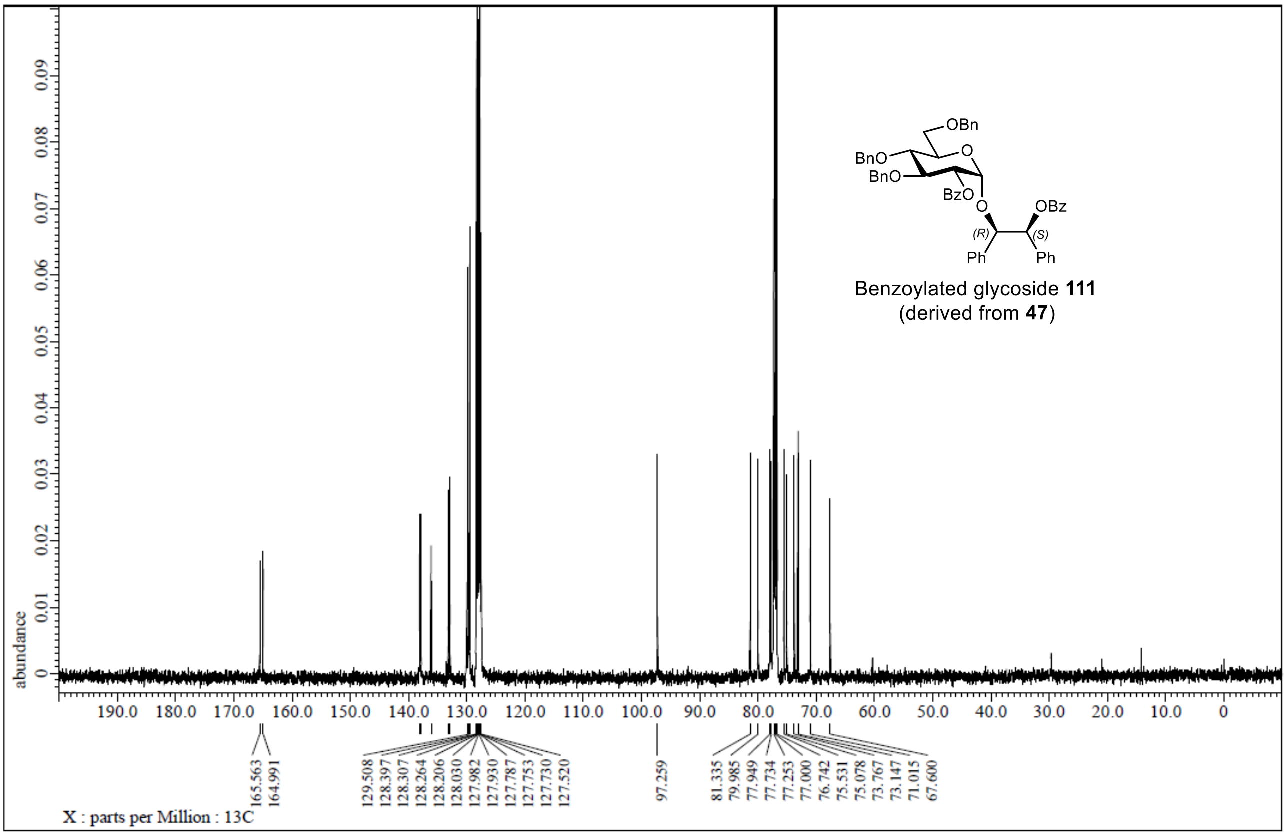

Supplementary Figure 102. ^{1}H and ^{13}C-NMR spectra of compound 111.

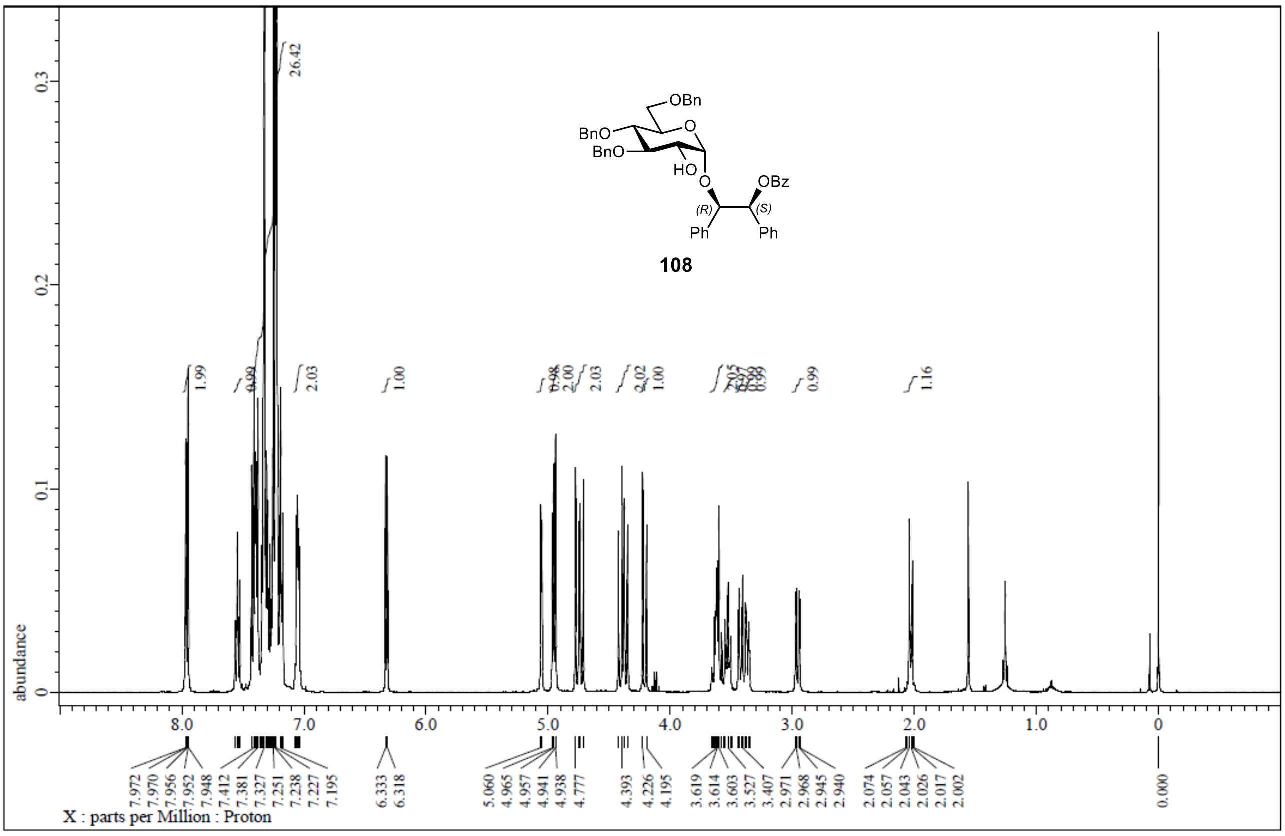

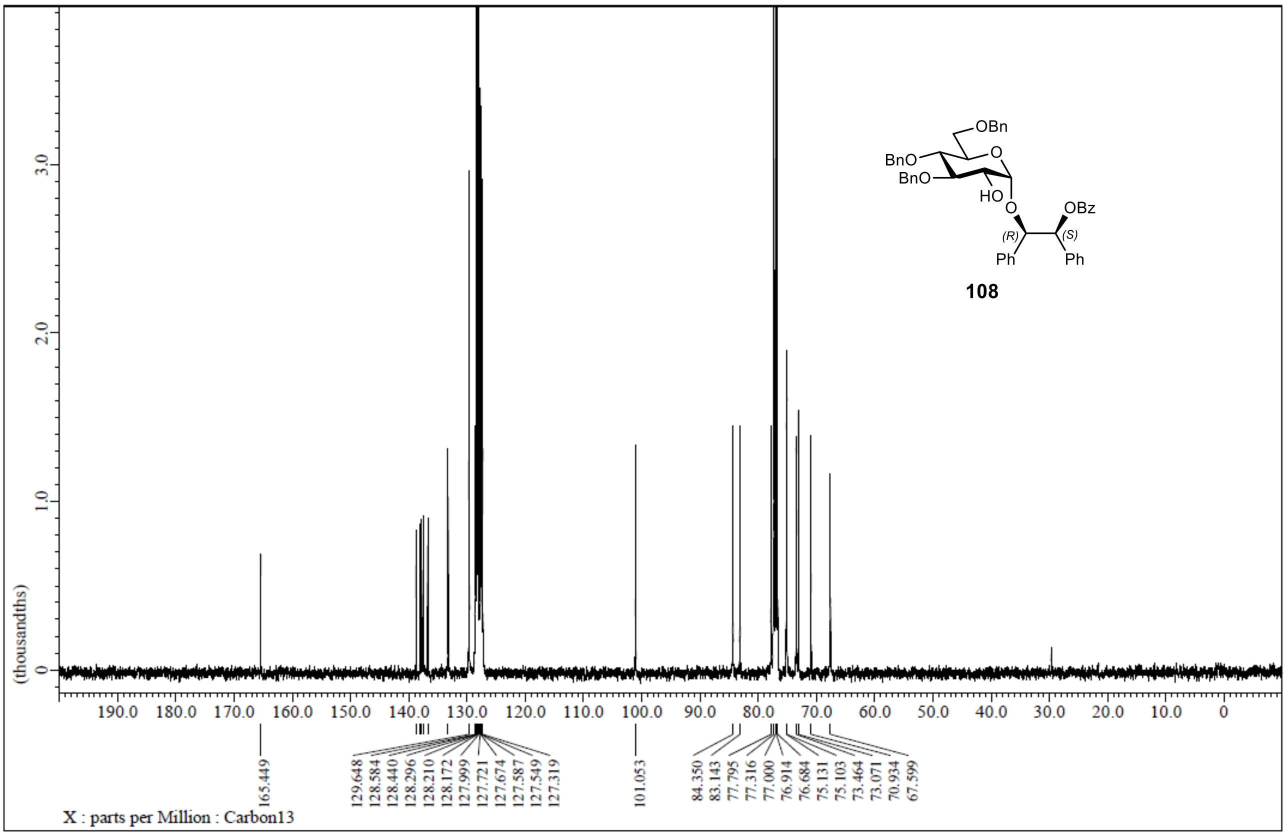

Supplementary Figure 103. ^{1}H and ^{13}C-NMR spectra of compound 108.

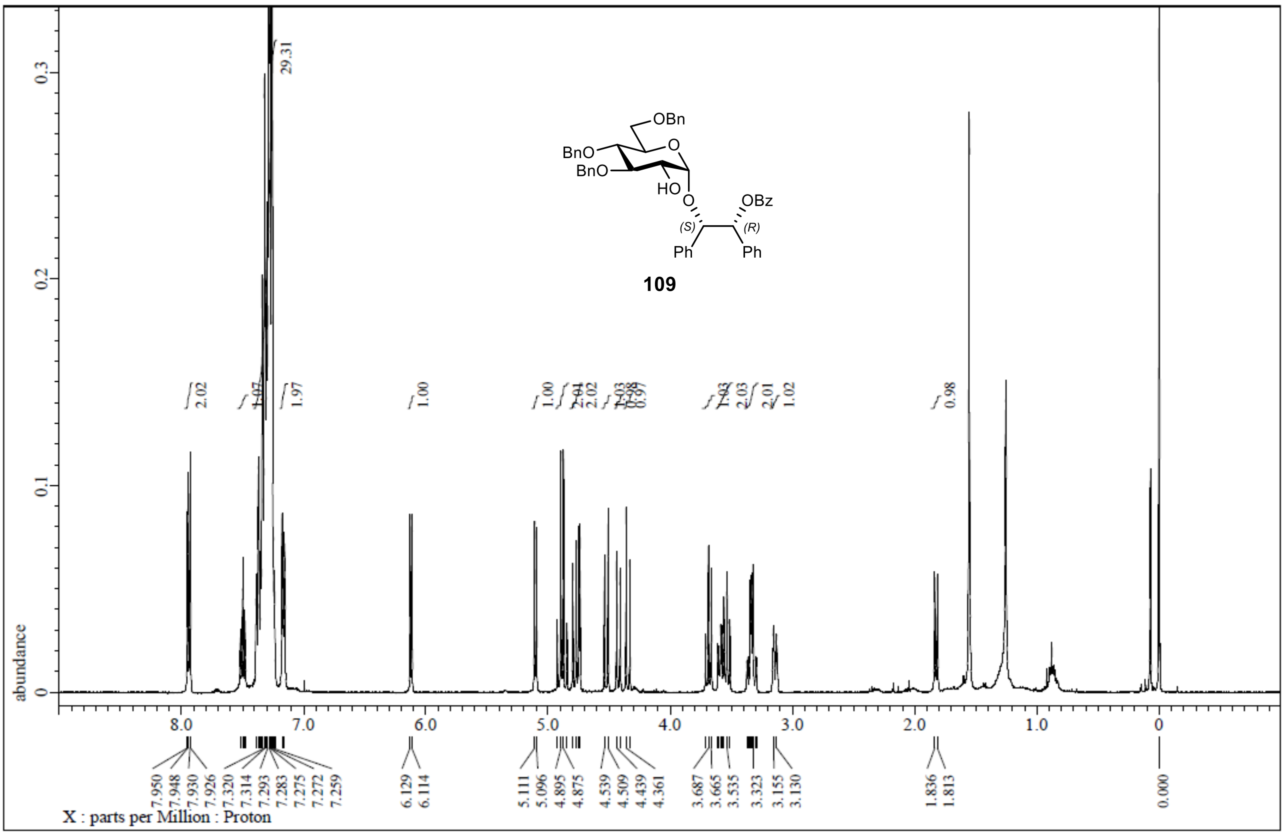

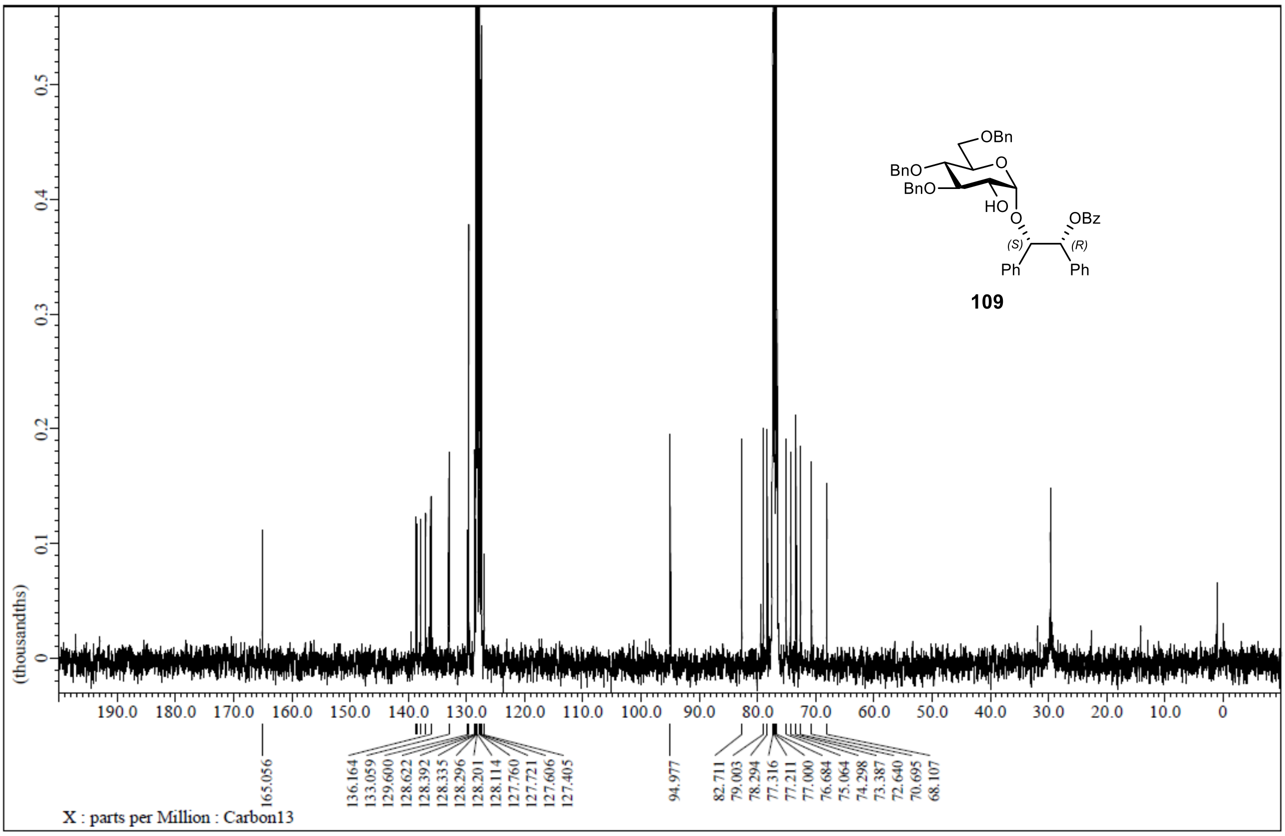

Supplementary Figure 104. ^{1}H and ^{13}C-NMR spectra of compound 109.

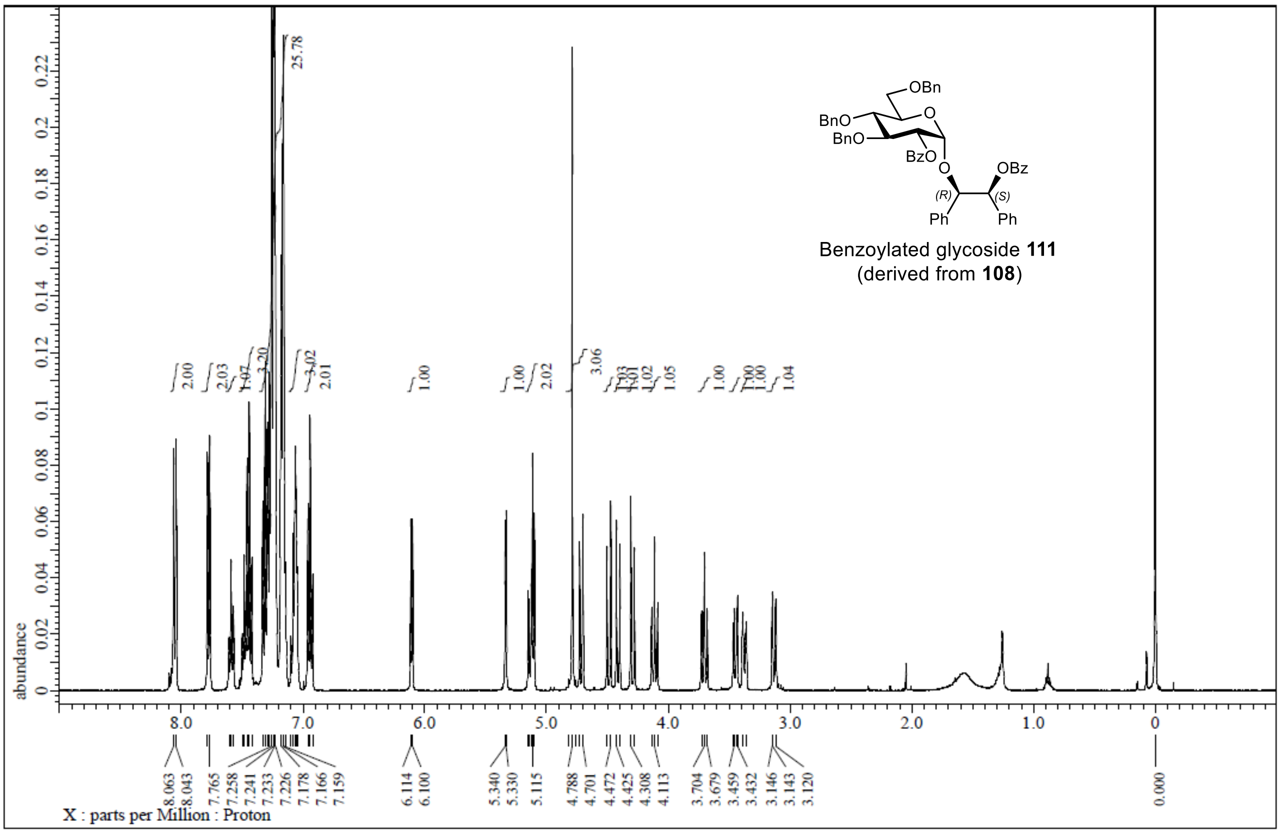

Supplementary Figure 105. ¹H-NMR spectrum of compound 111.

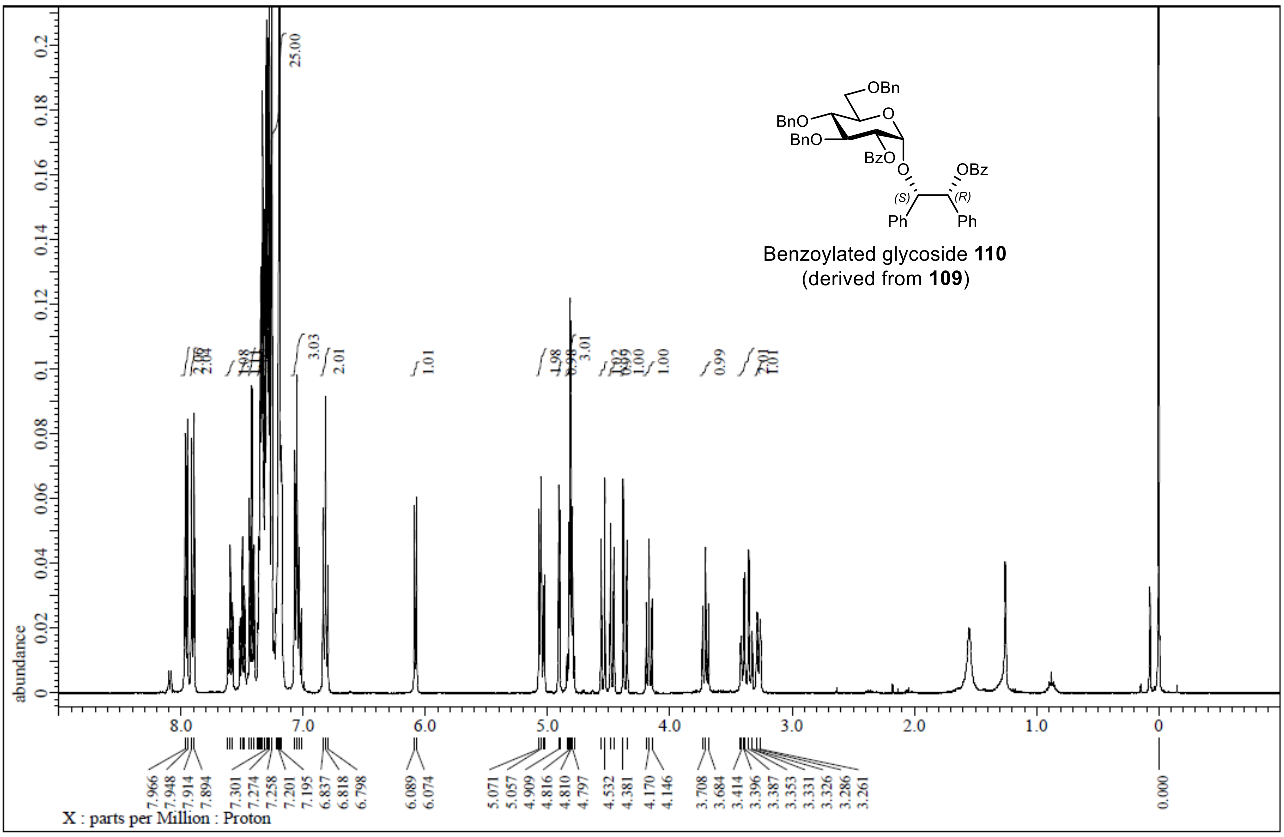

Supplementary Figure 106. ¹H-NMR spectrum of compound 110.

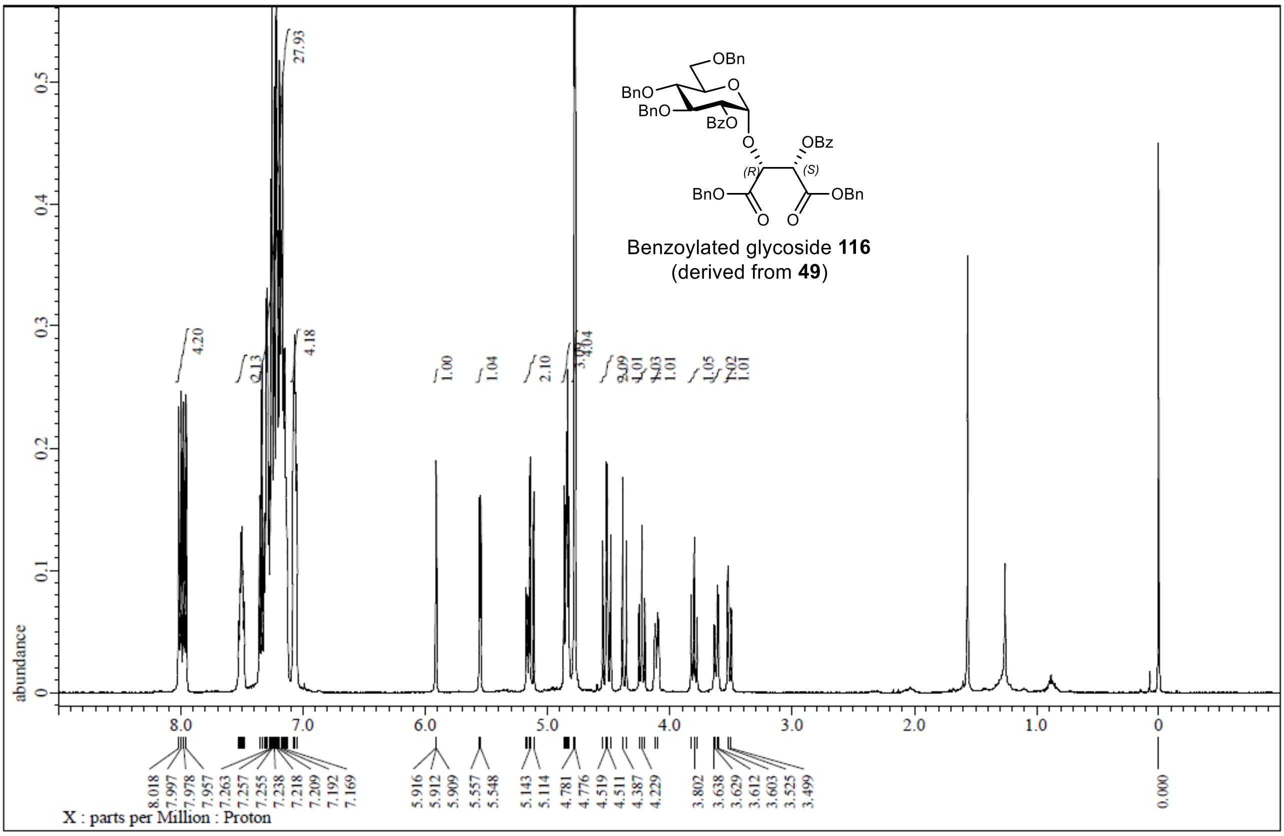

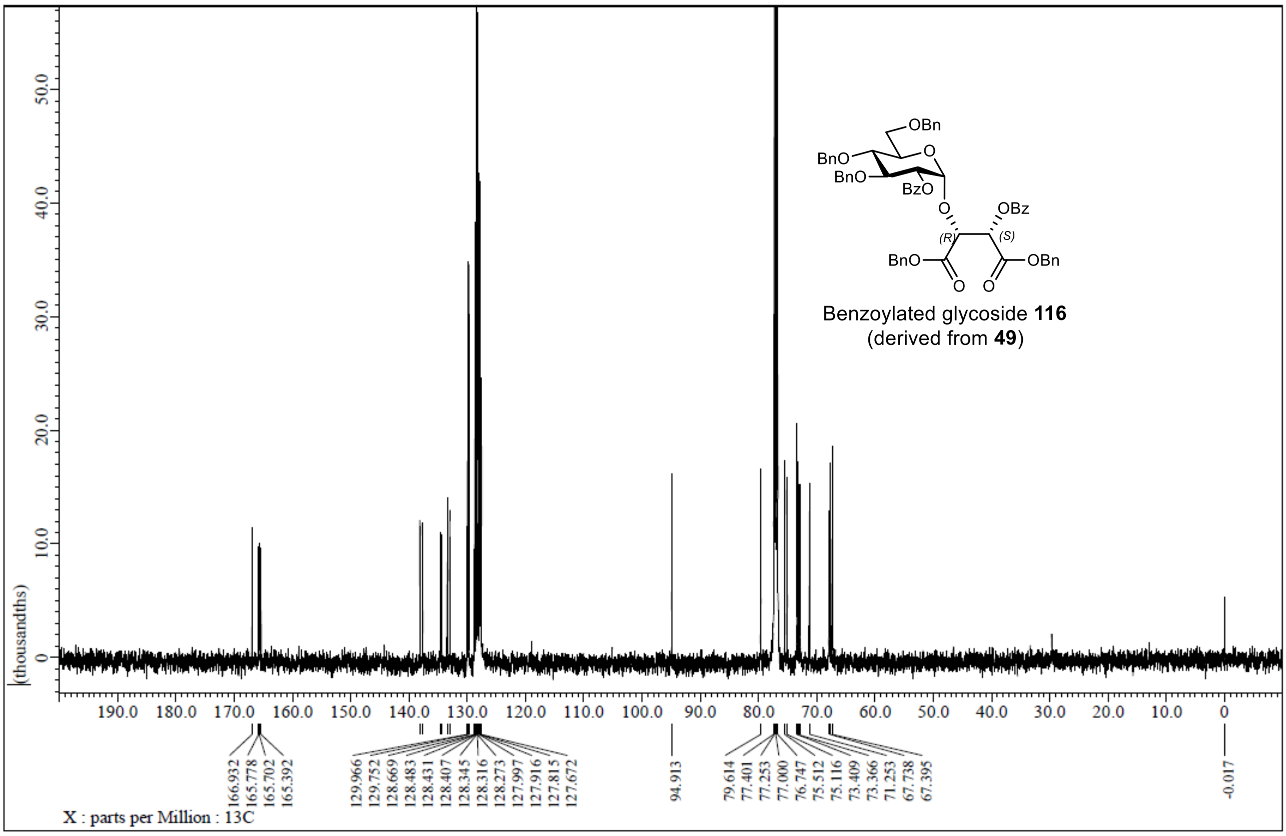

Supplementary Figure 107. ^{1}H and ^{13}C-NMR spectra of compound 116.

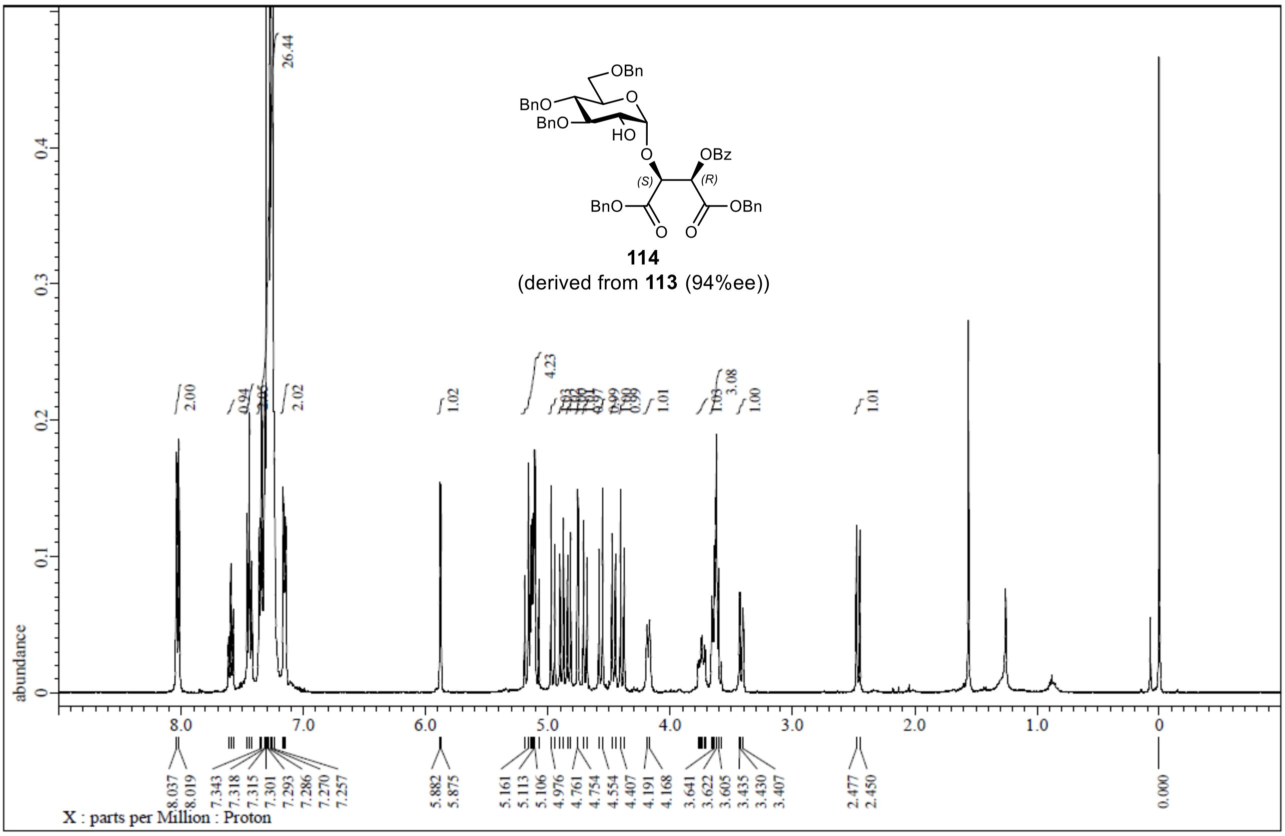

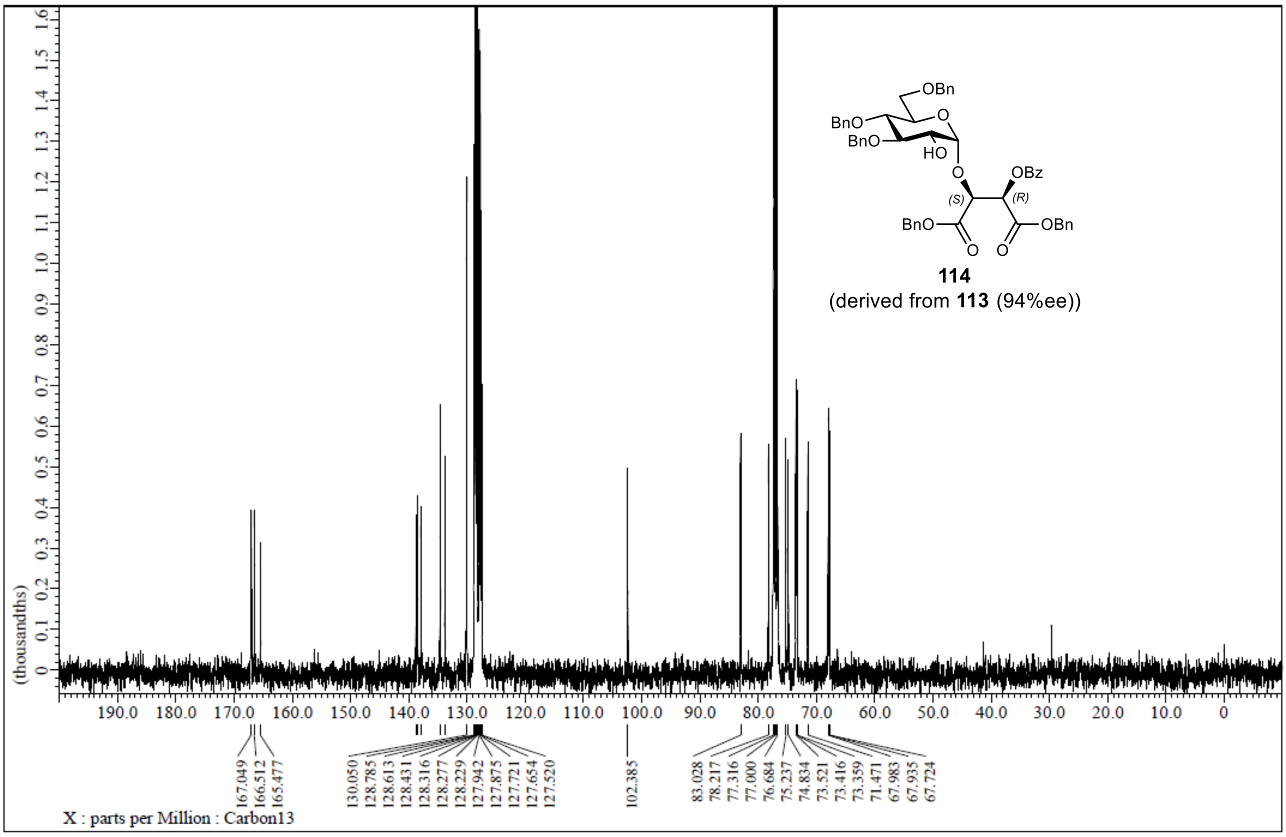

Supplementary Figure 108. ^{1}H and ^{13}C-NMR spectra of compound 114.

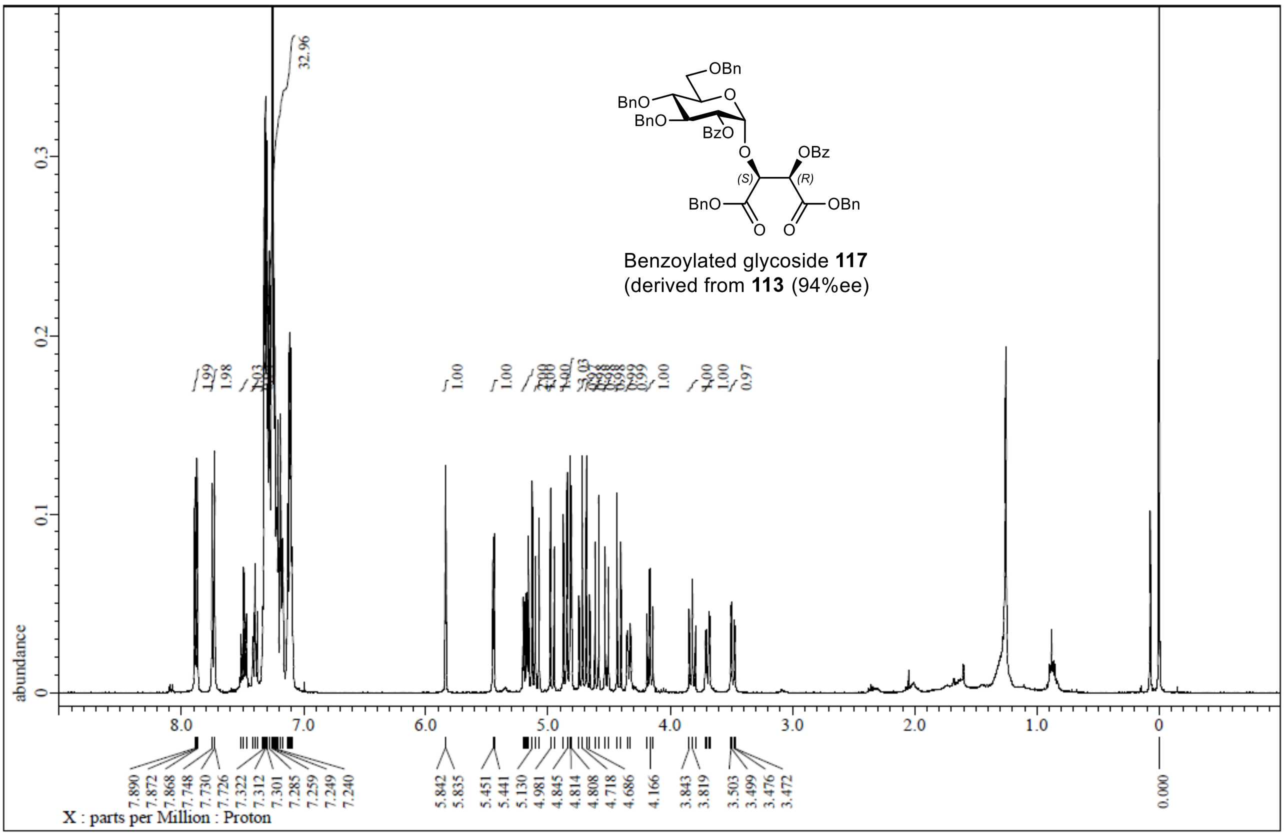

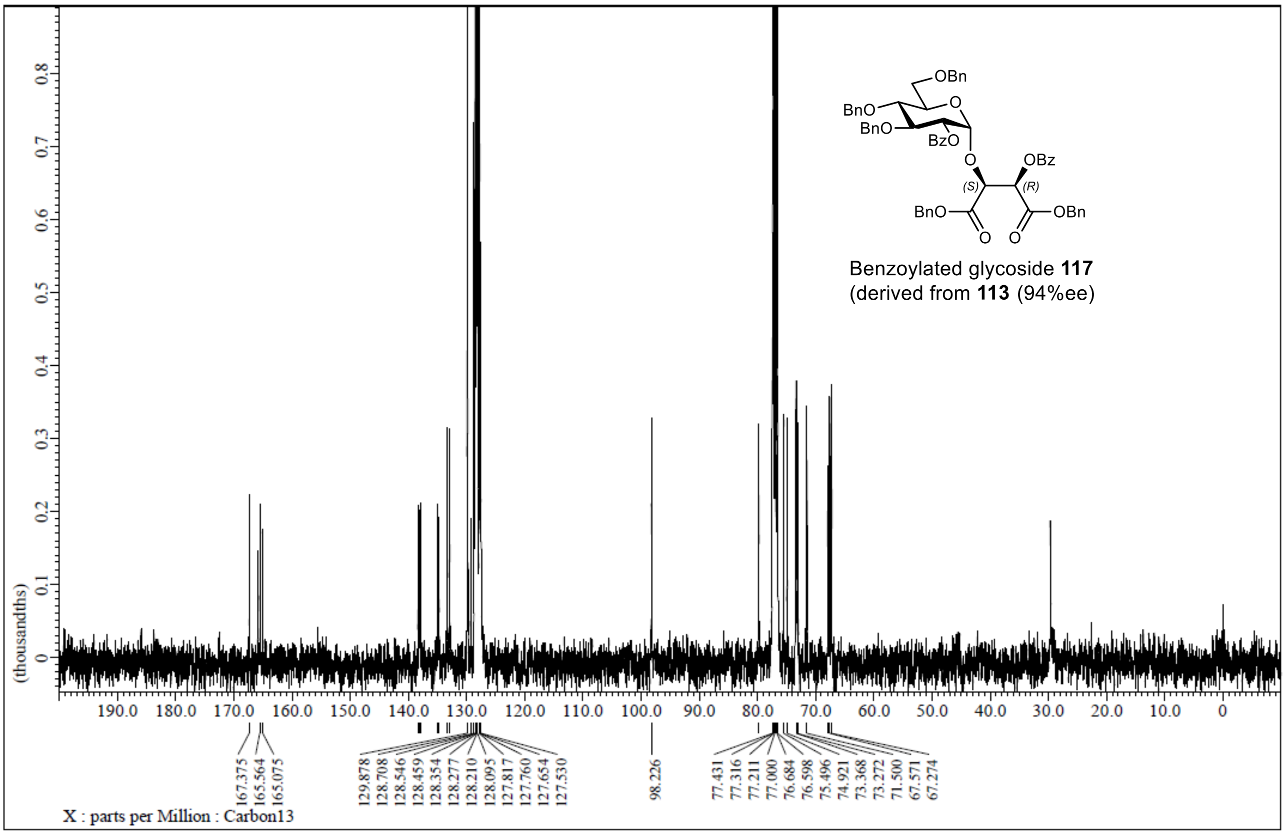

Supplementary Figure 109. ^{1}H and ^{13}C-NMR spectra of compound 117.

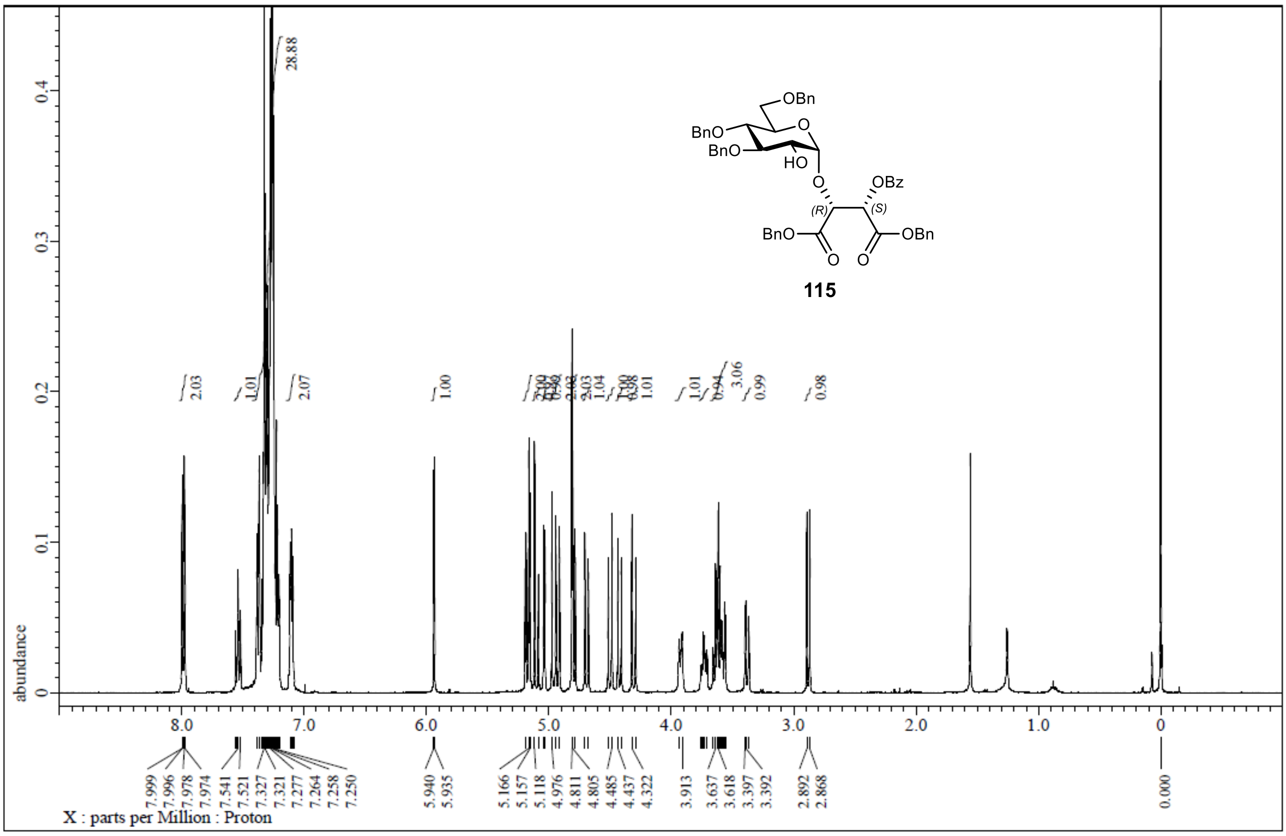

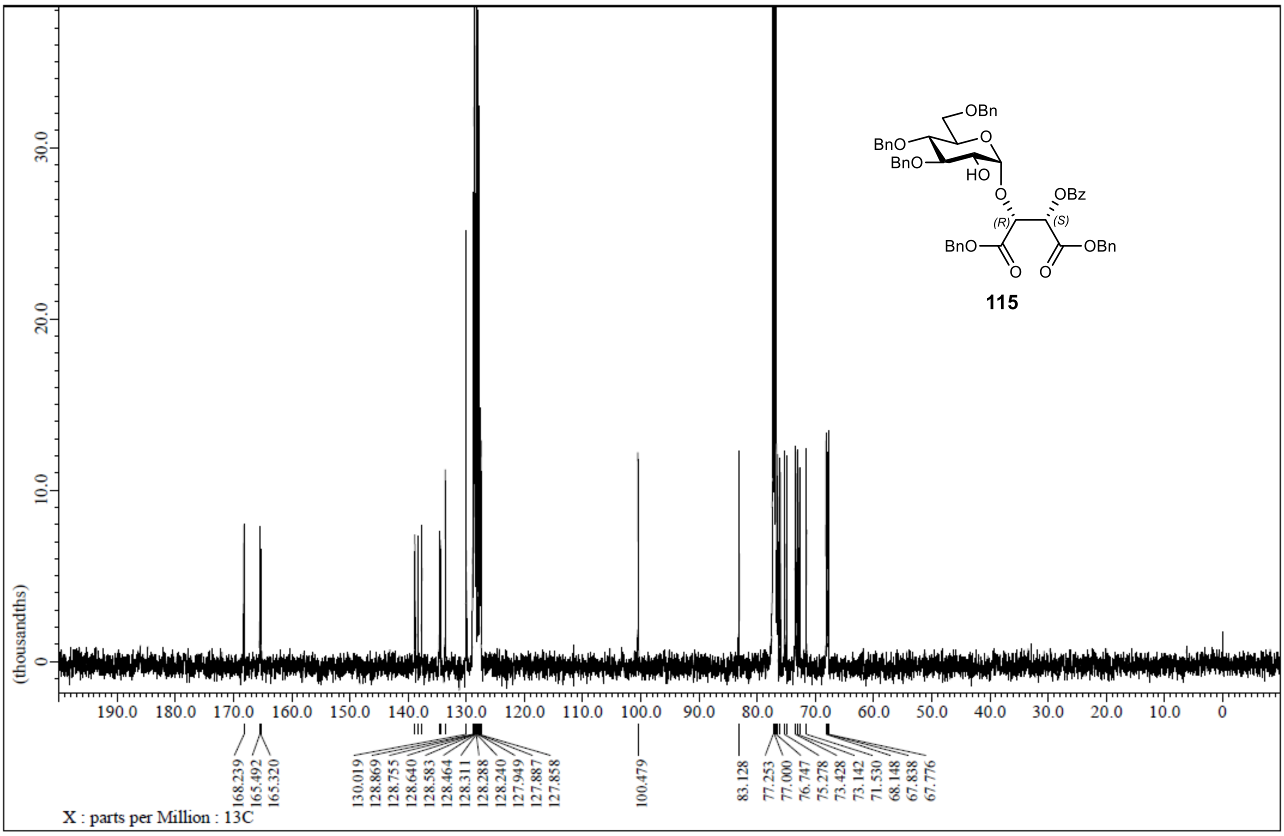

Supplementary Figure 110. ^{1}H and ^{13}C-NMR spectra of compound 115.

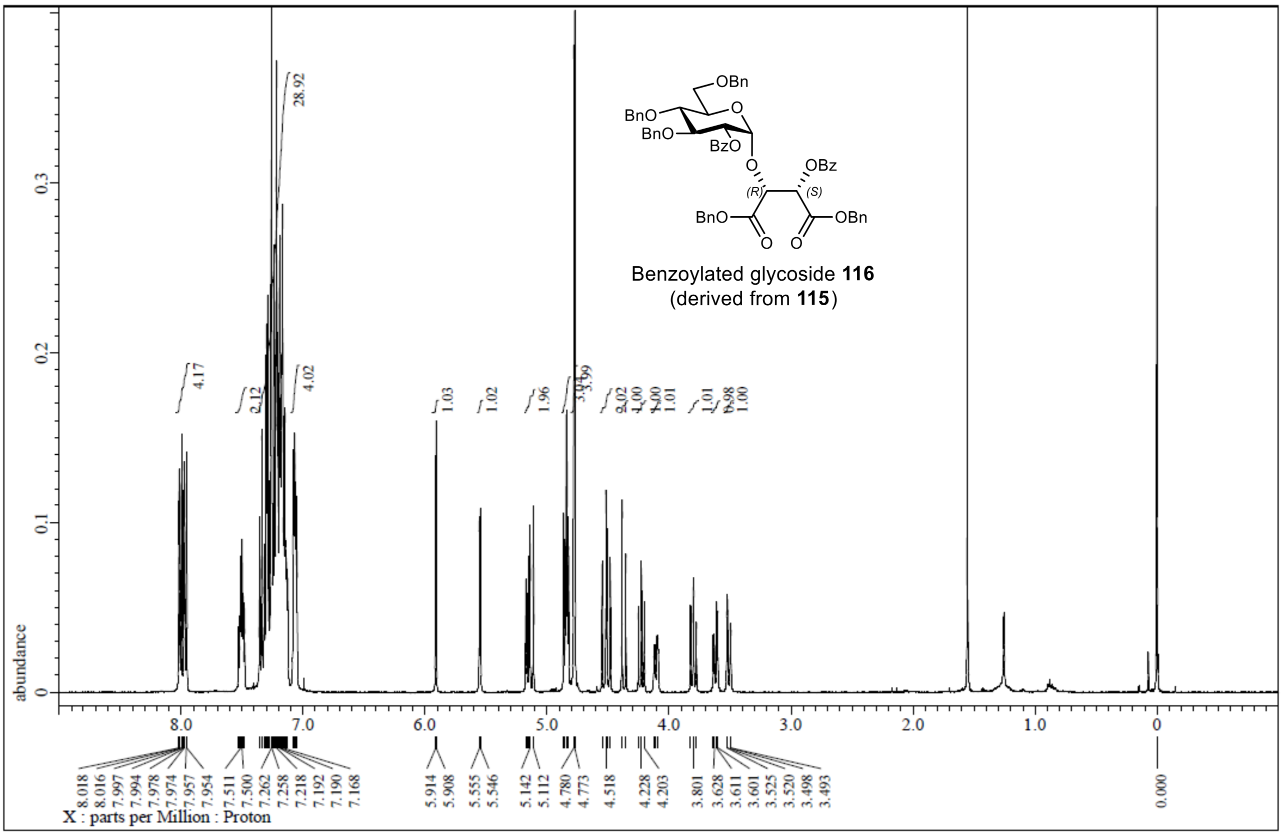

Supplementary Figure 111. ^{1}H-NMR spectrum of compound 116.

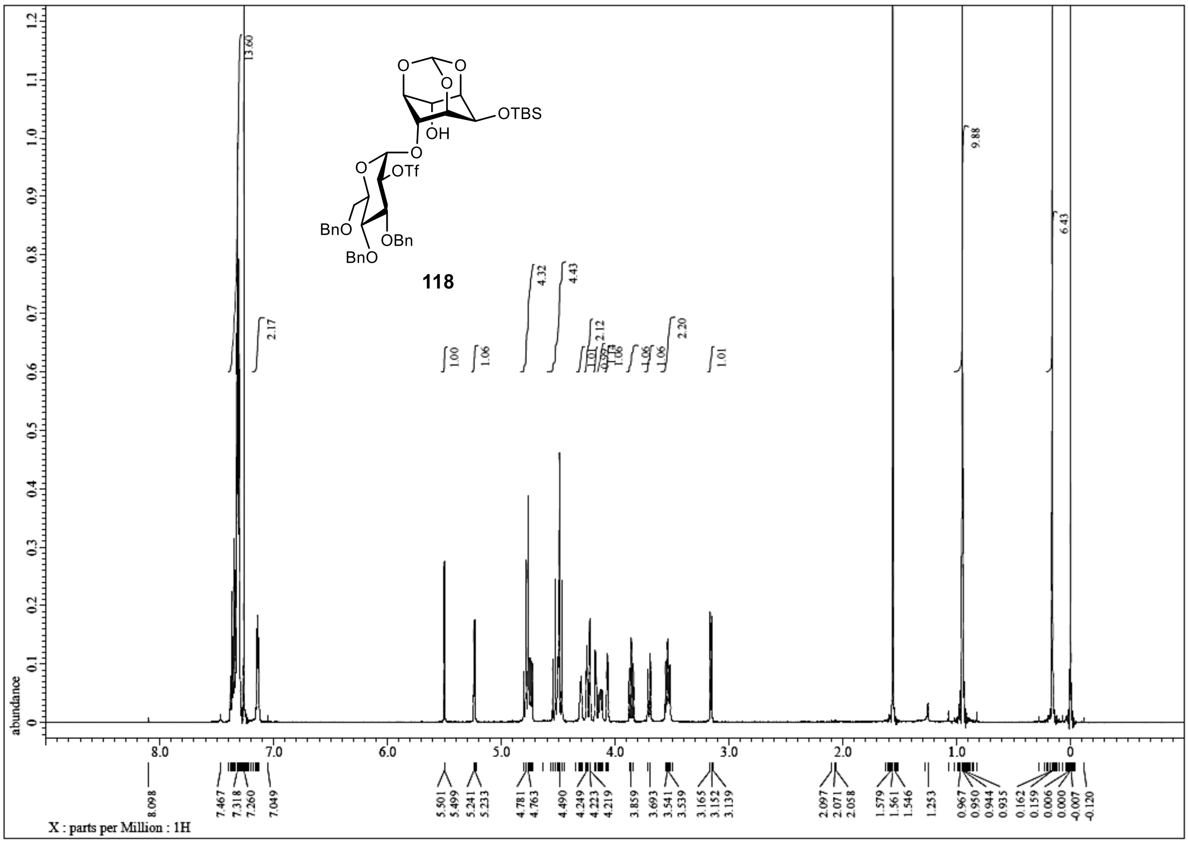

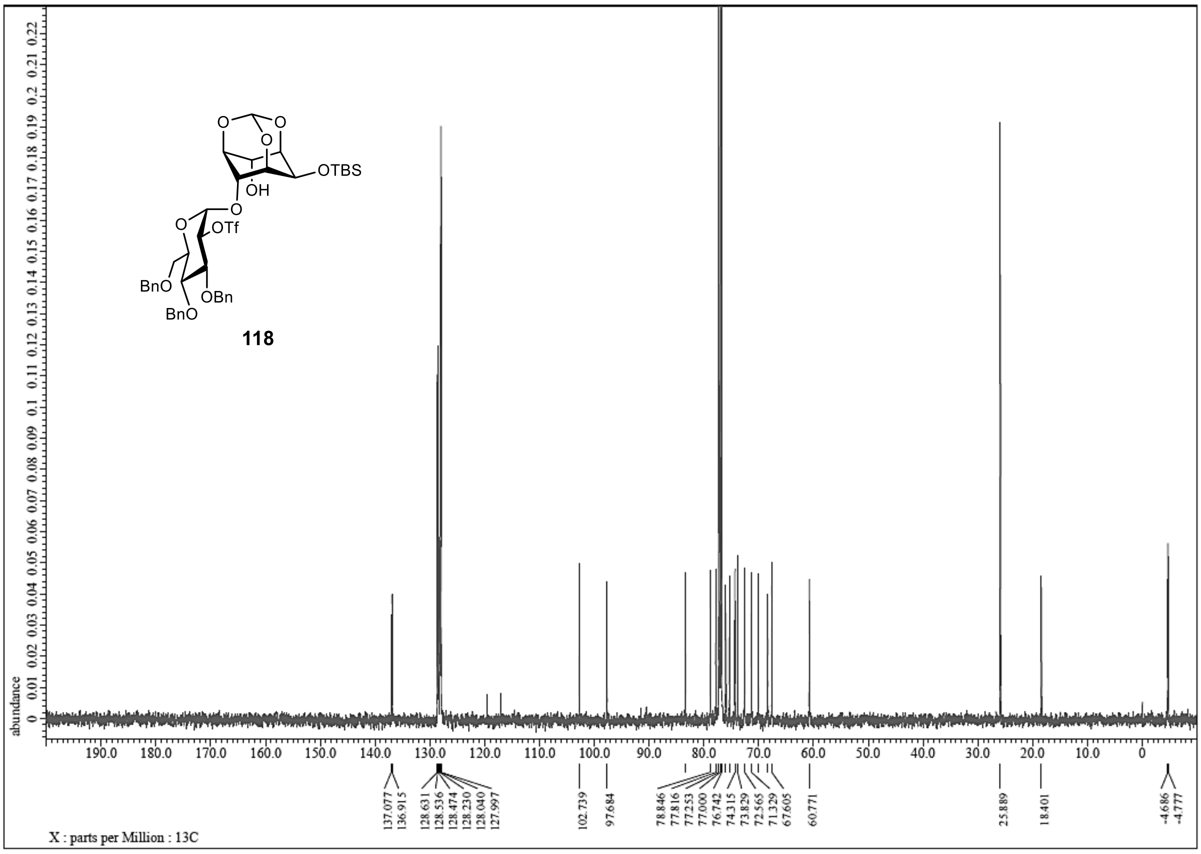

Supplementary Figure 112. ^{1}H and ^{13}C-NMR spectra of compound 118.

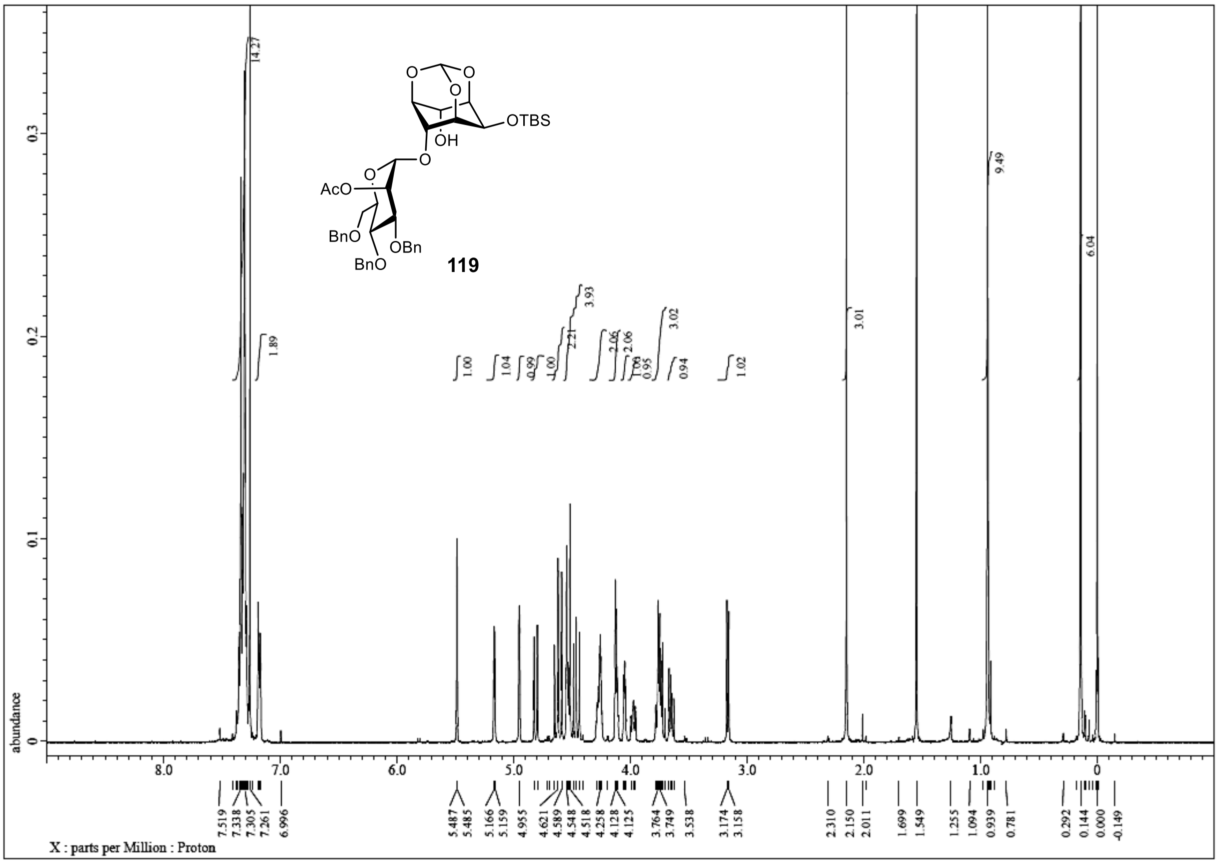

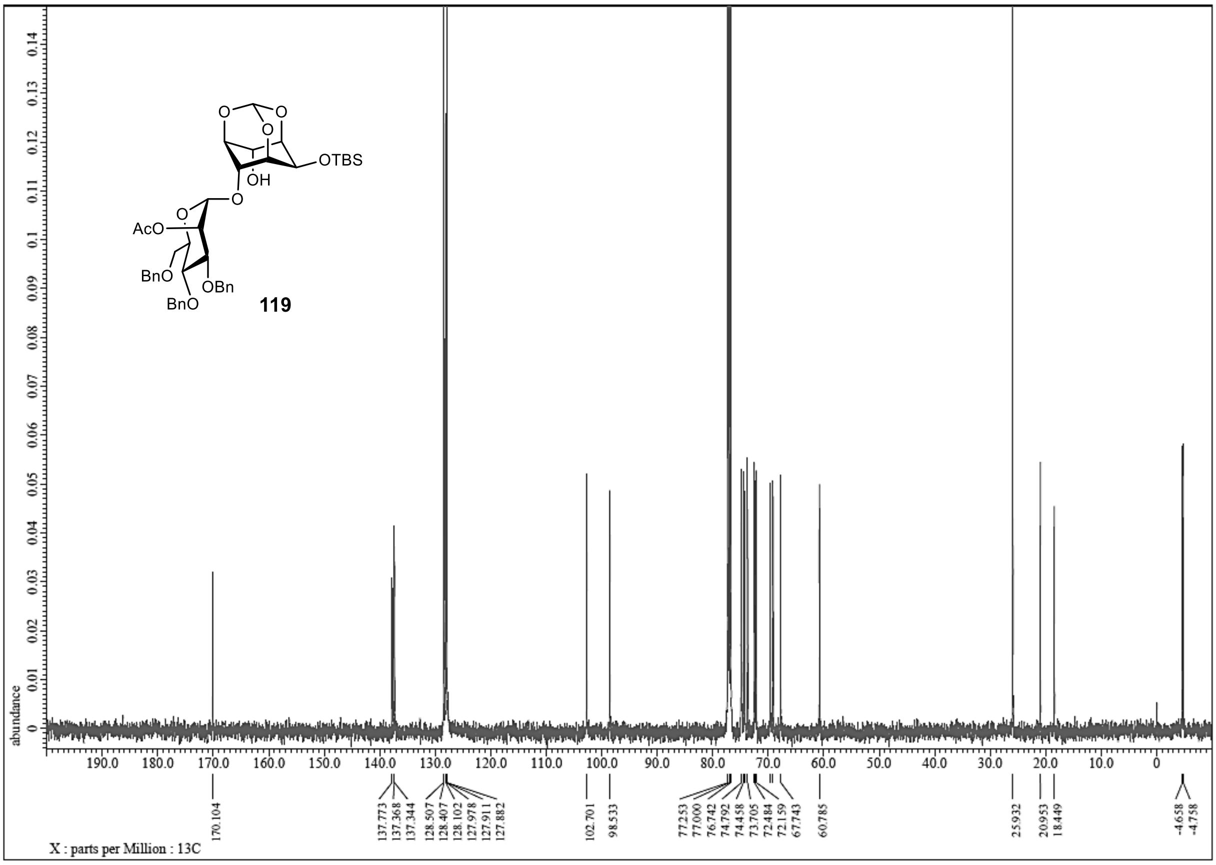

Supplementary Figure 113. ^{1}H and ^{13}C-NMR spectra of compound 119.

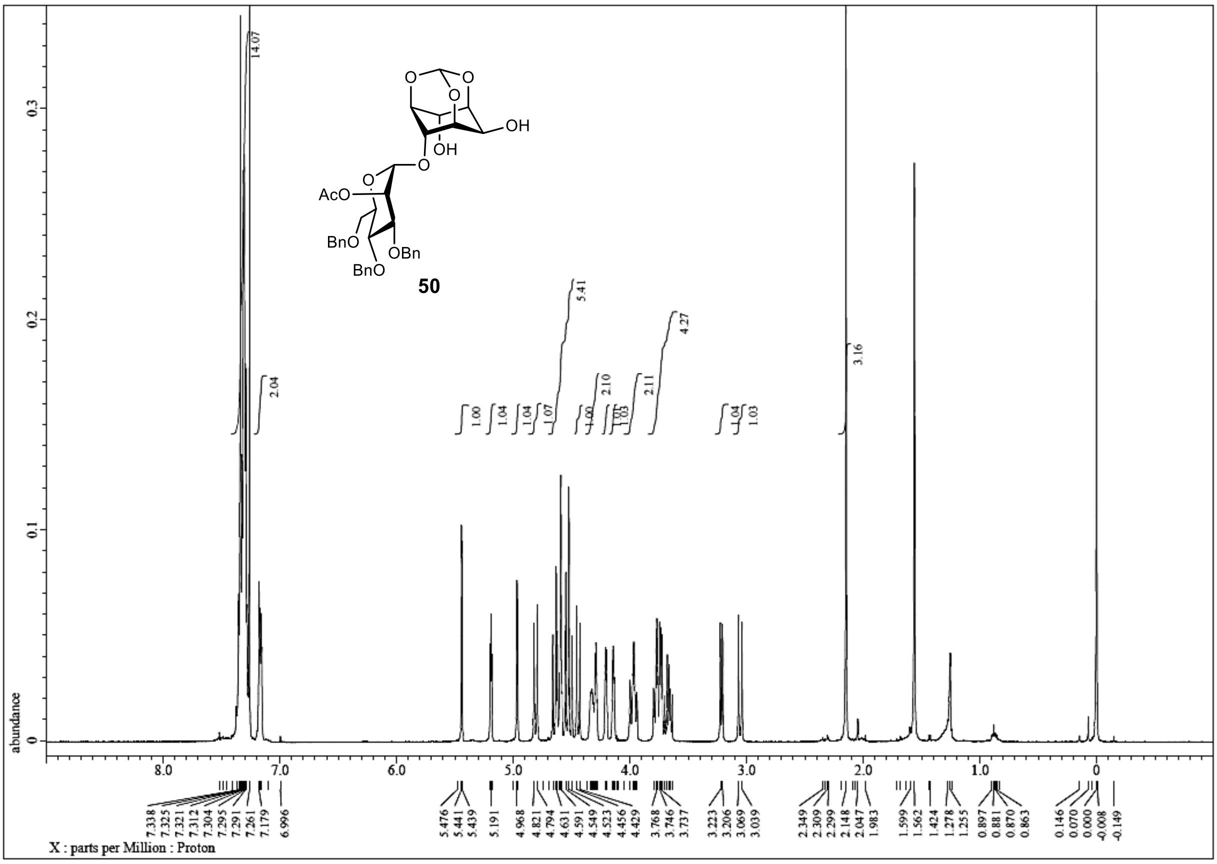

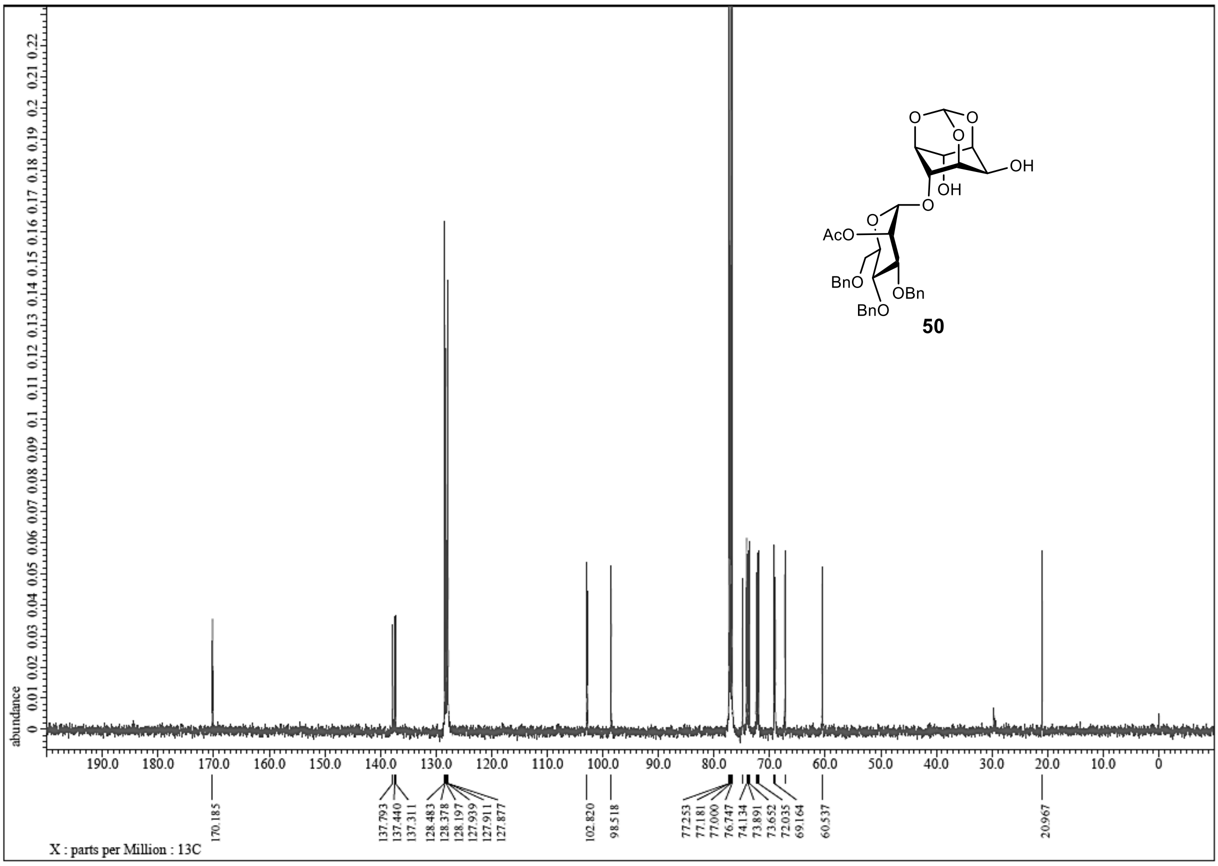

Supplementary Figure 114. ^{1}H and ^{13}C-NMR spectra of compound 50.

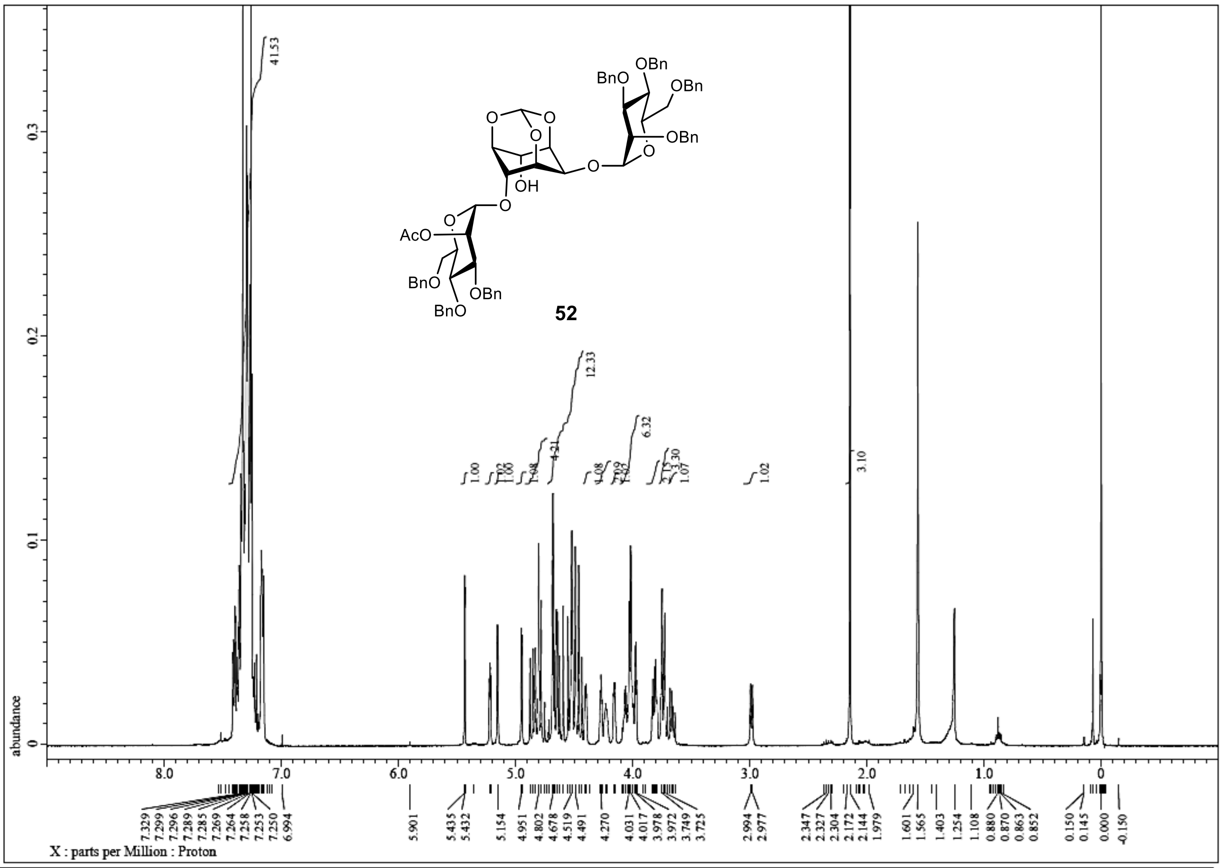

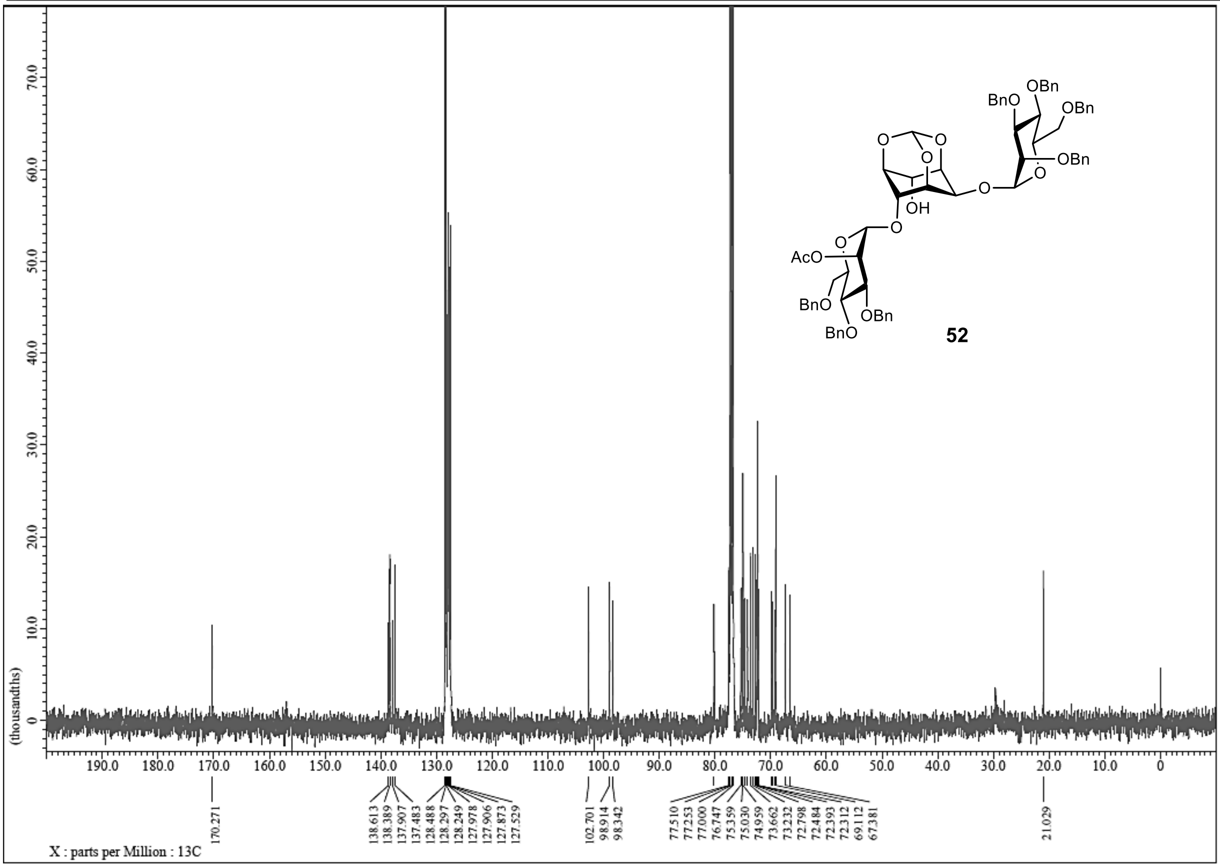

Supplementary Figure 115. ^{1}H and ^{13}C-NMR spectra of compound 52.

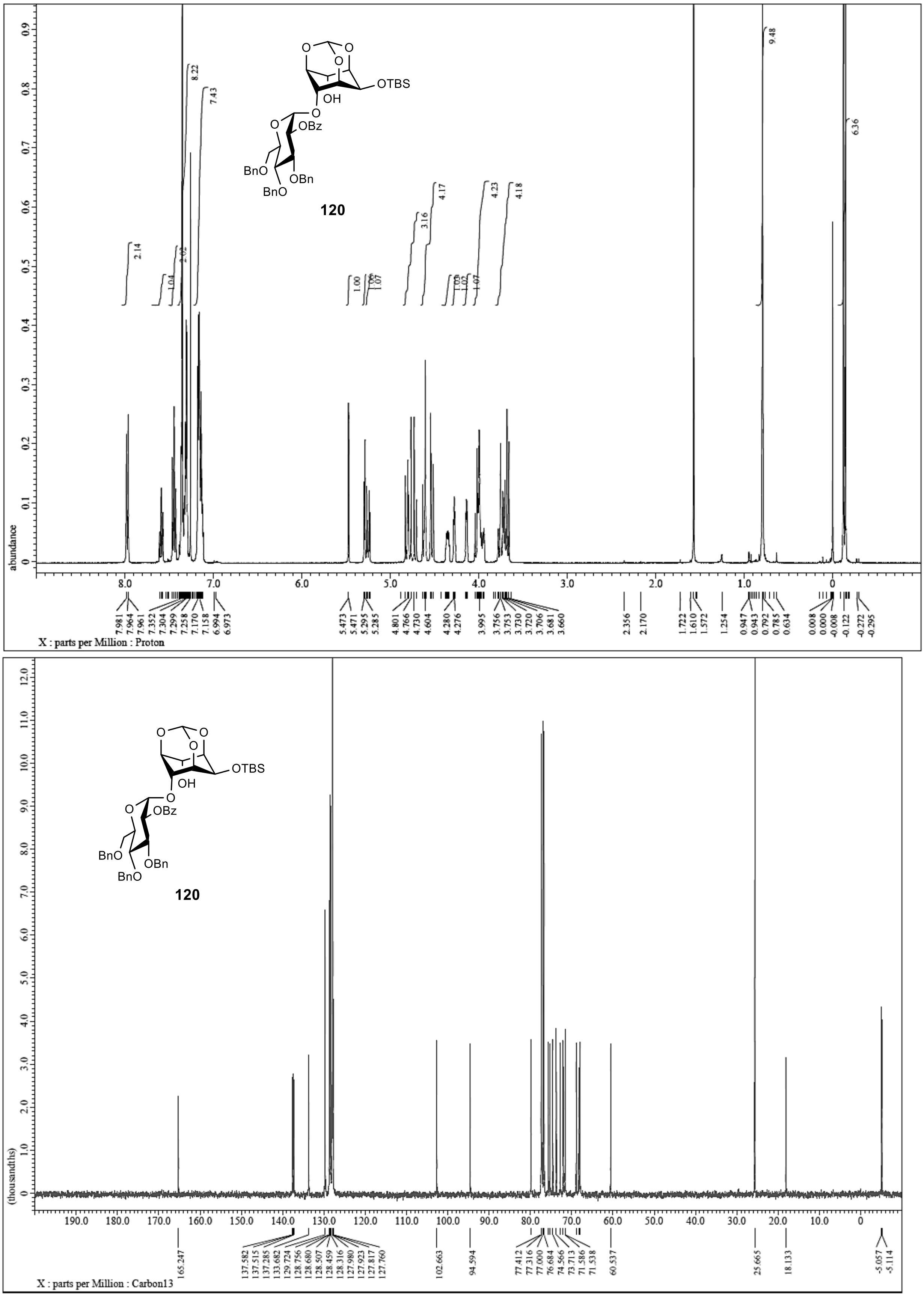

Supplementary Figure 116. ^{1}H and ^{13}C-NMR spectra of compound 120.

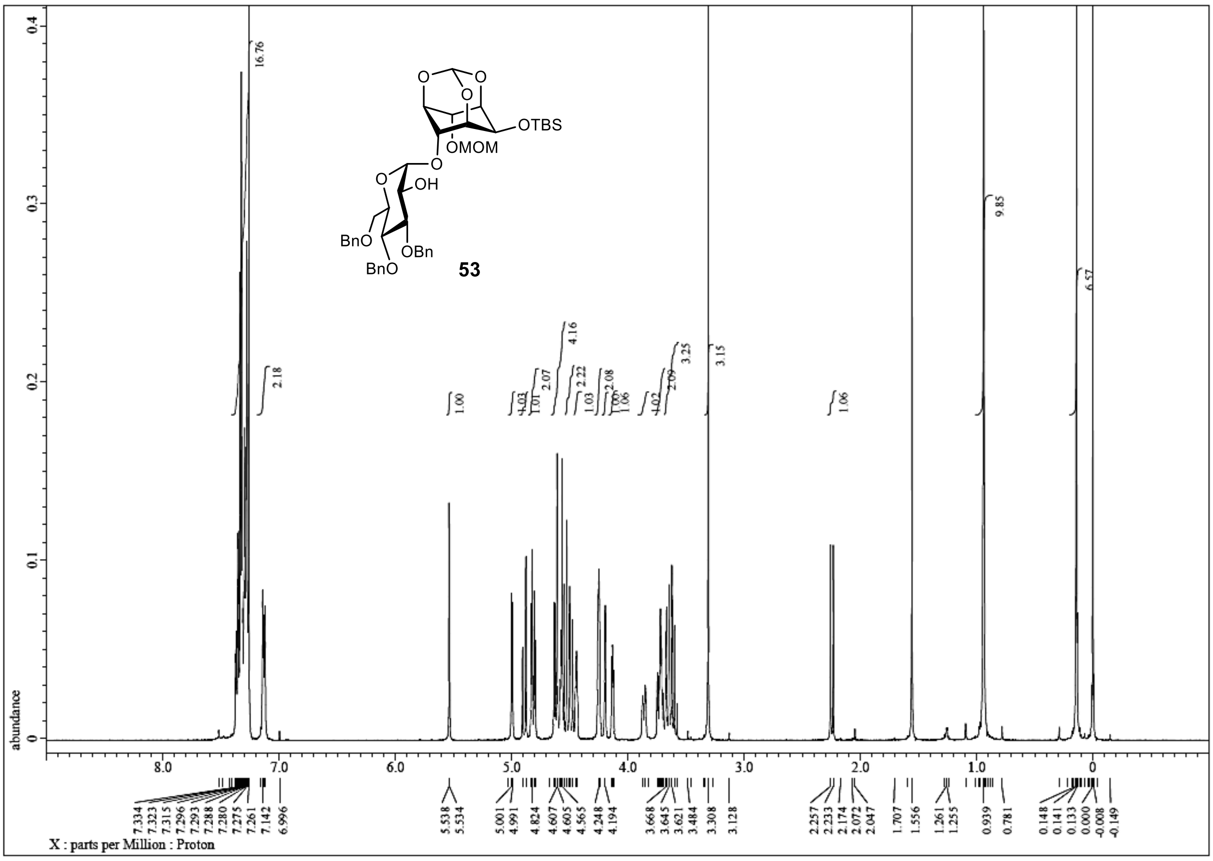

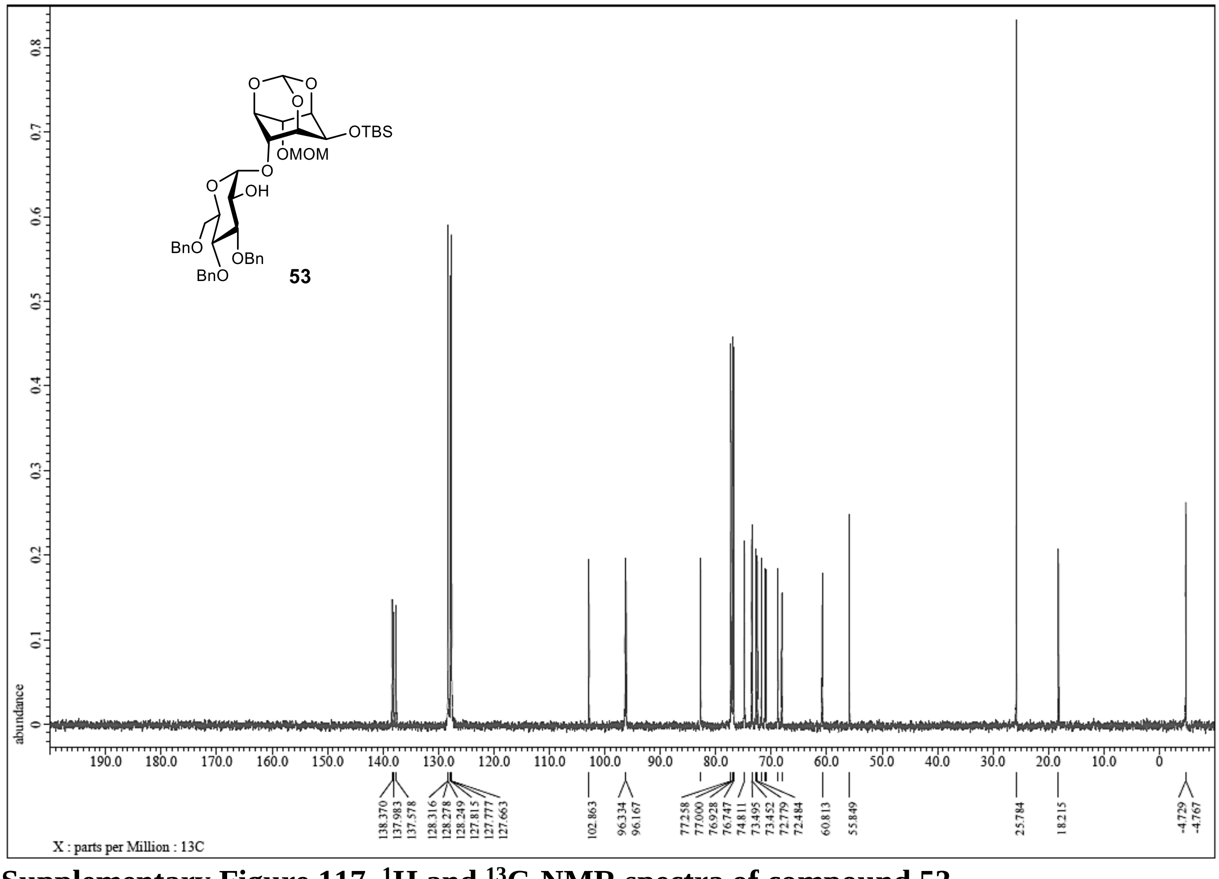

Supplementary Figure 117. ^{1}H and ^{13}C-NMR spectra of compound 53.

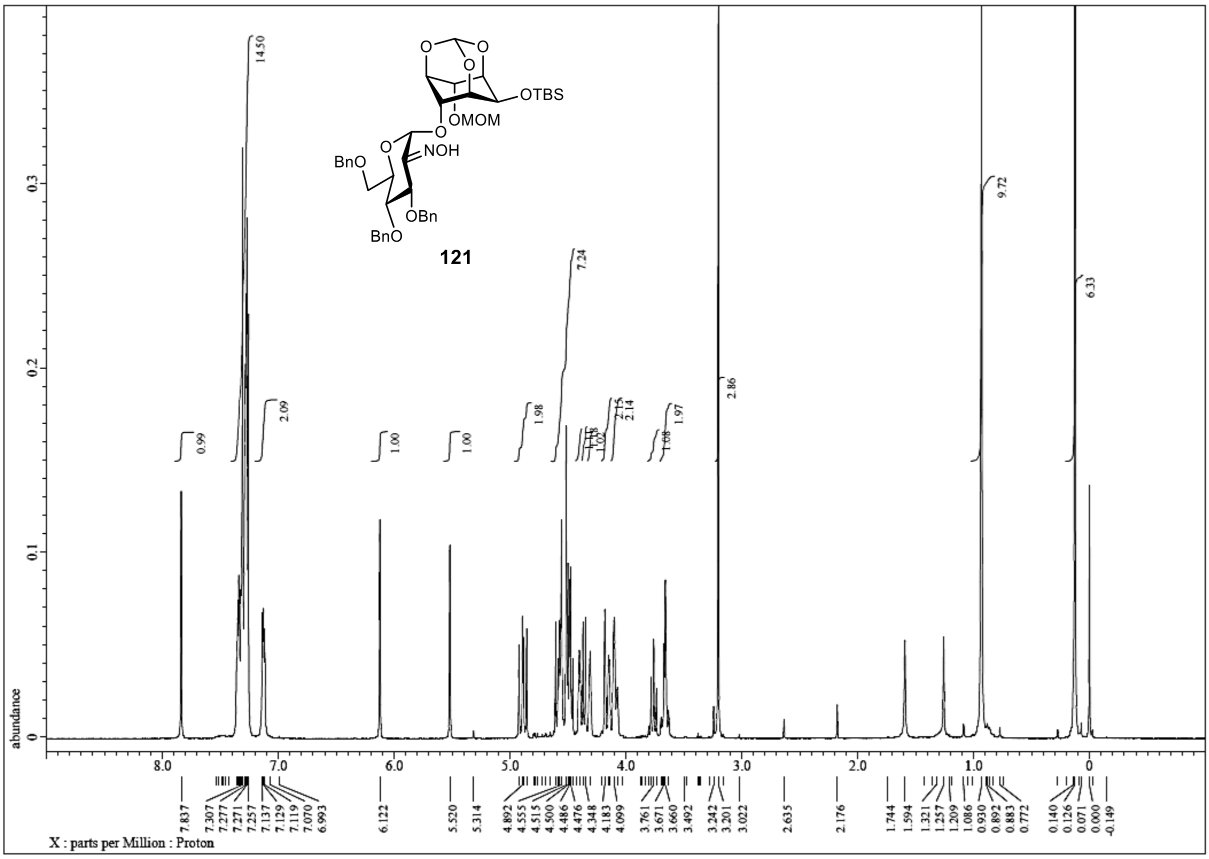

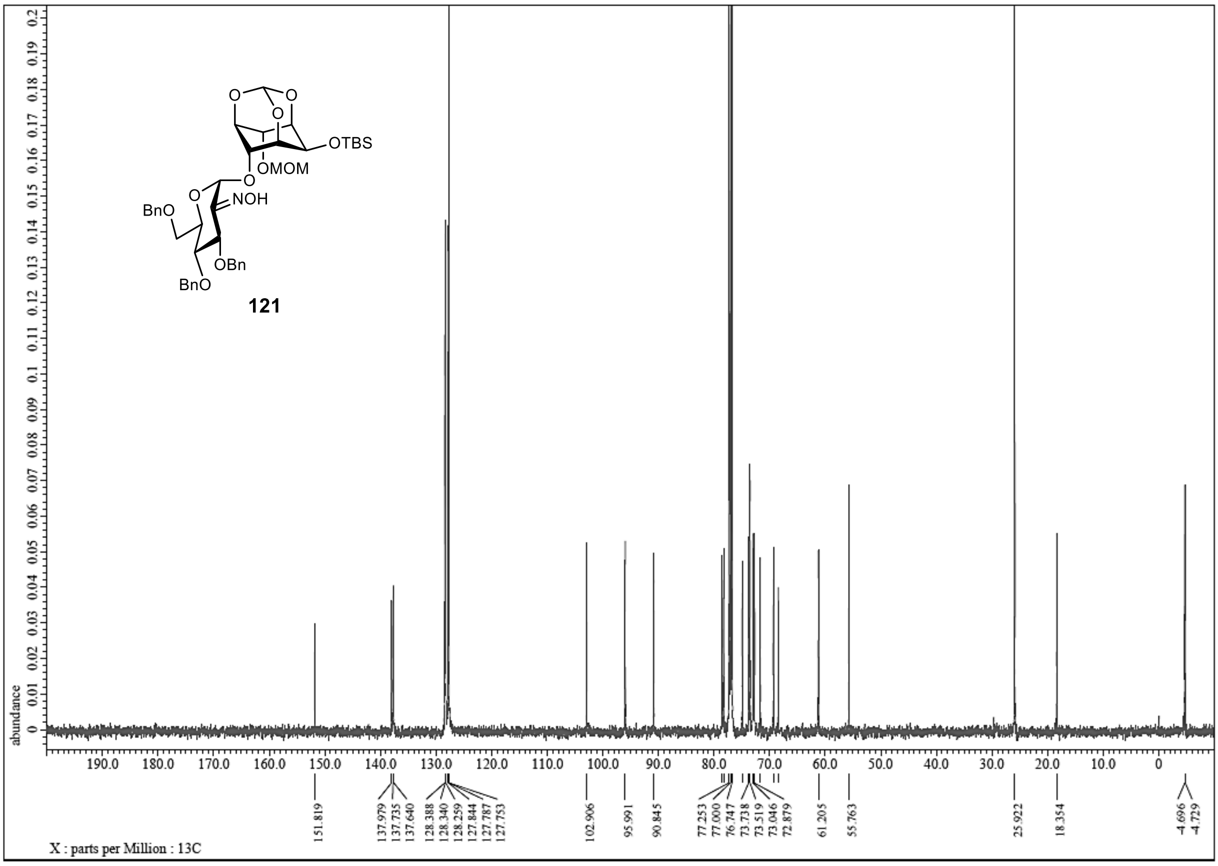

Supplementary Figure 118. ¹H and ¹³C-NMR spectra of compound 121.

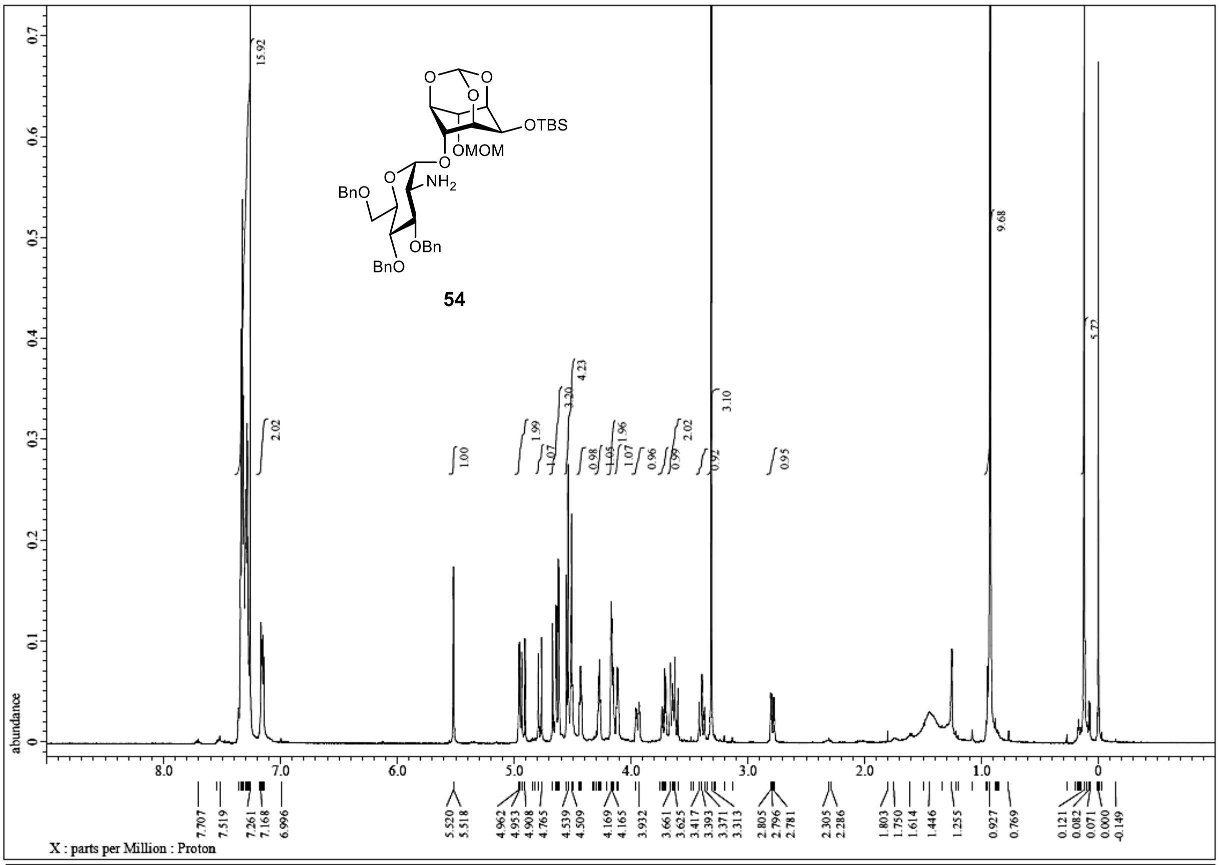

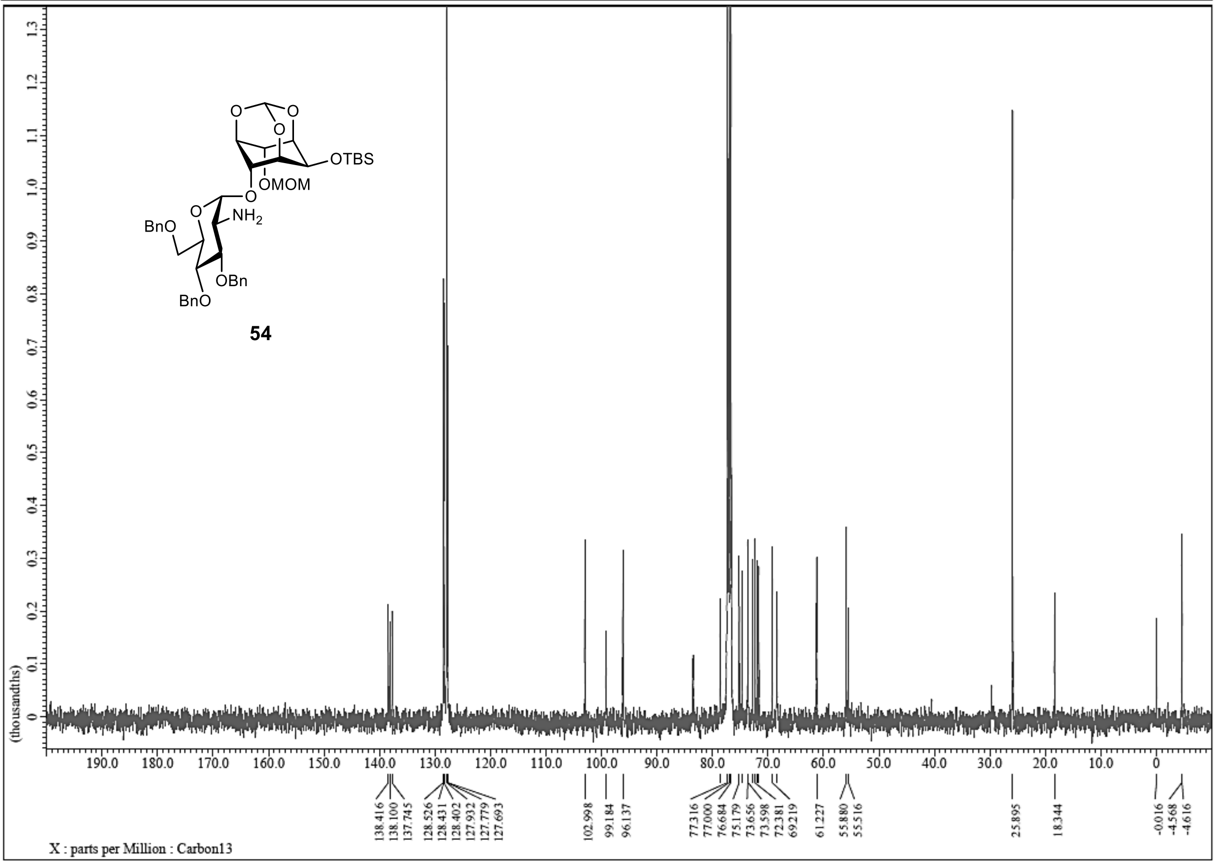

Supplementary Figure 119. ^{1}H and ^{13}C-NMR spectra of compound 54.

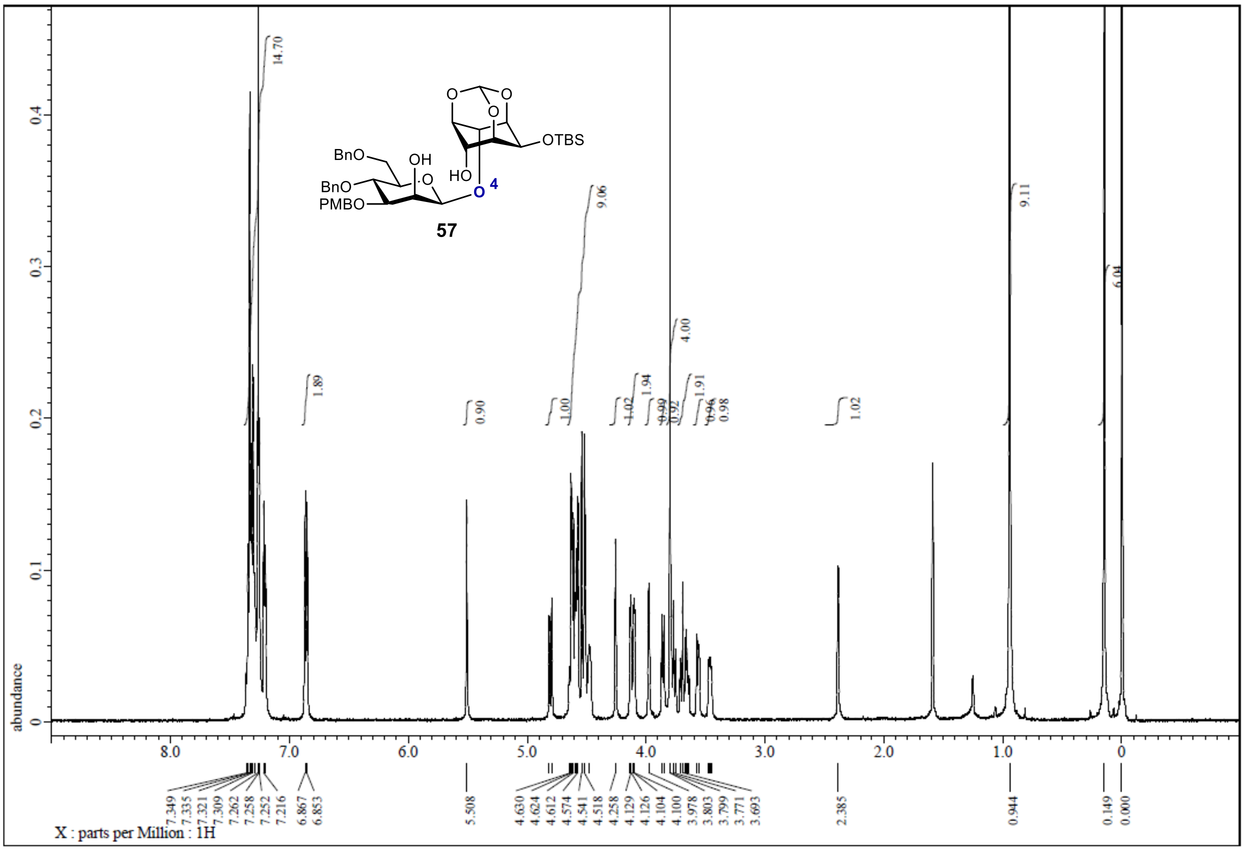

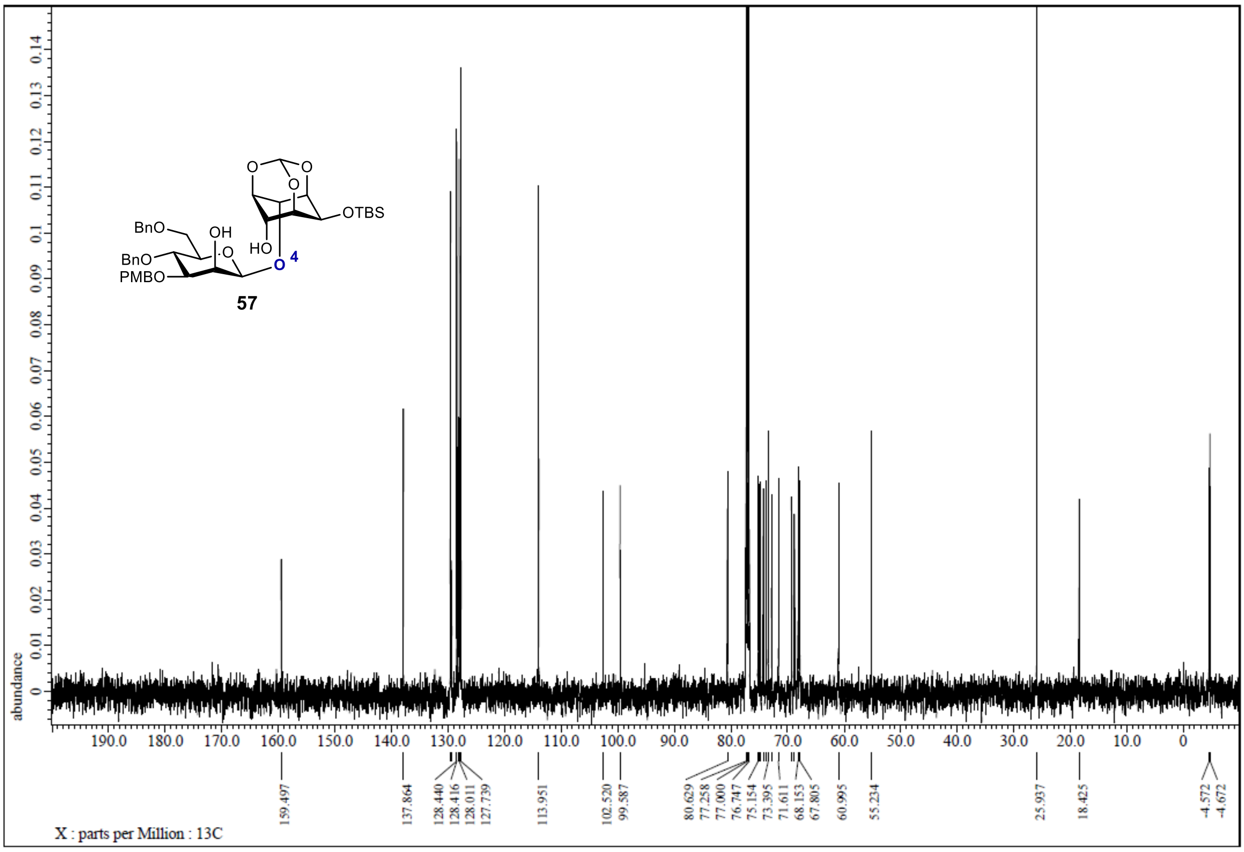

Supplementary Figure 120. ^{1}H and ^{13}C-NMR spectra of compound 57.

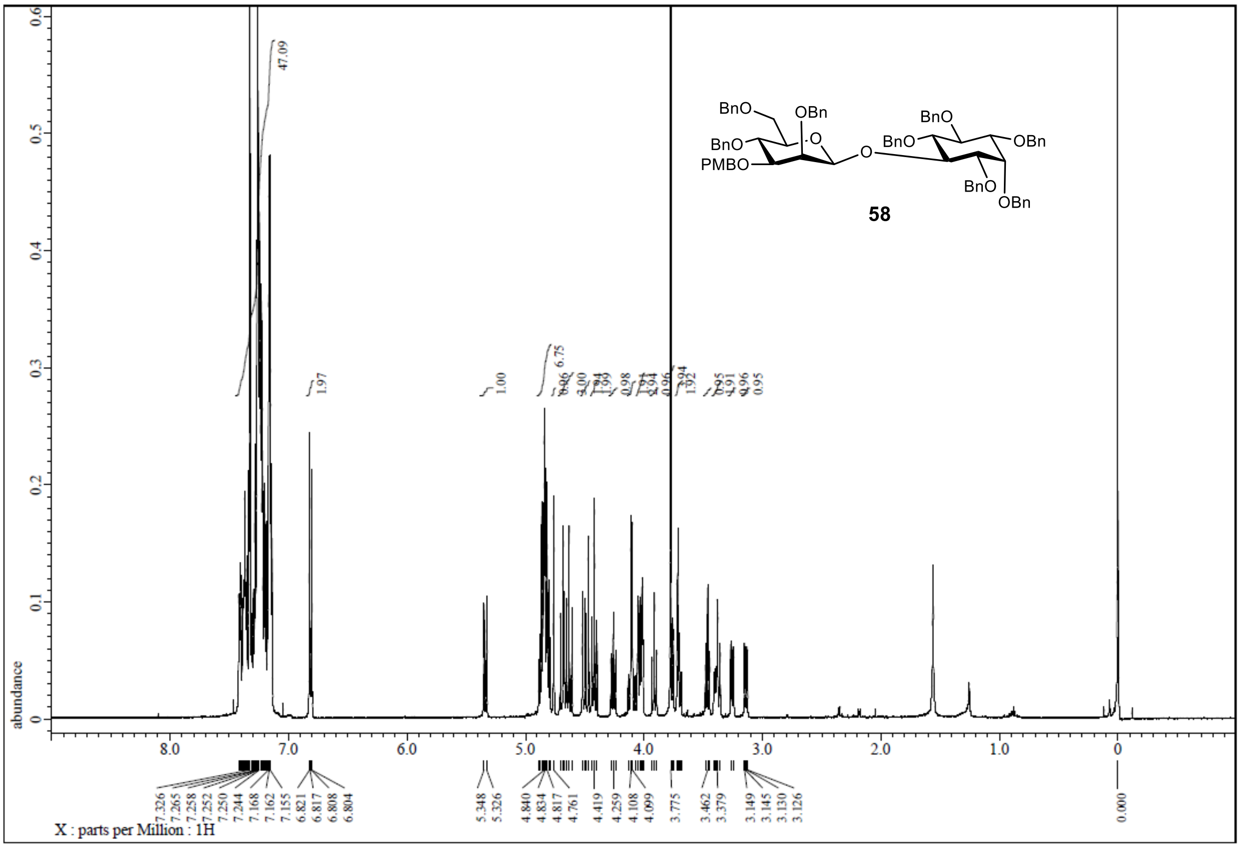

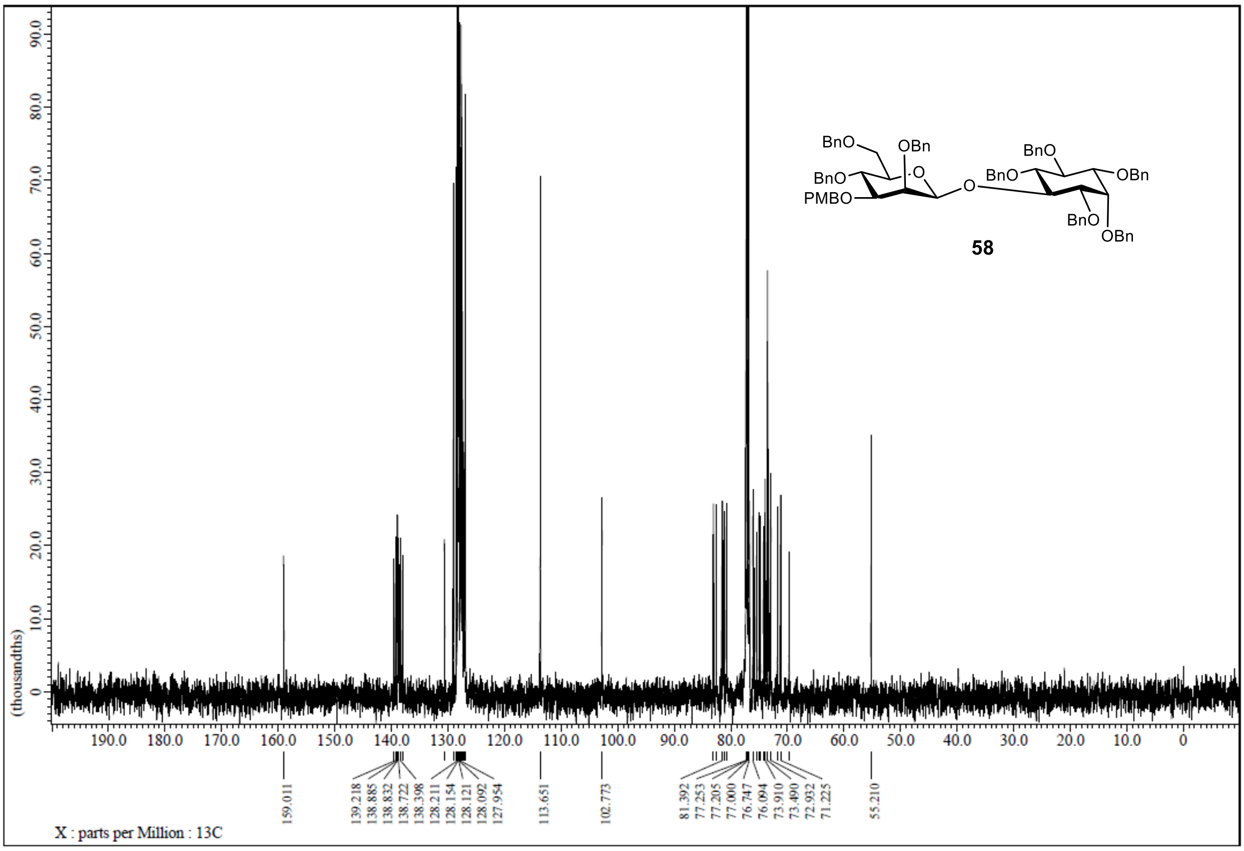

Supplementary Figure 121. ^{1}H and ^{13}C-NMR spectra of compound 58.

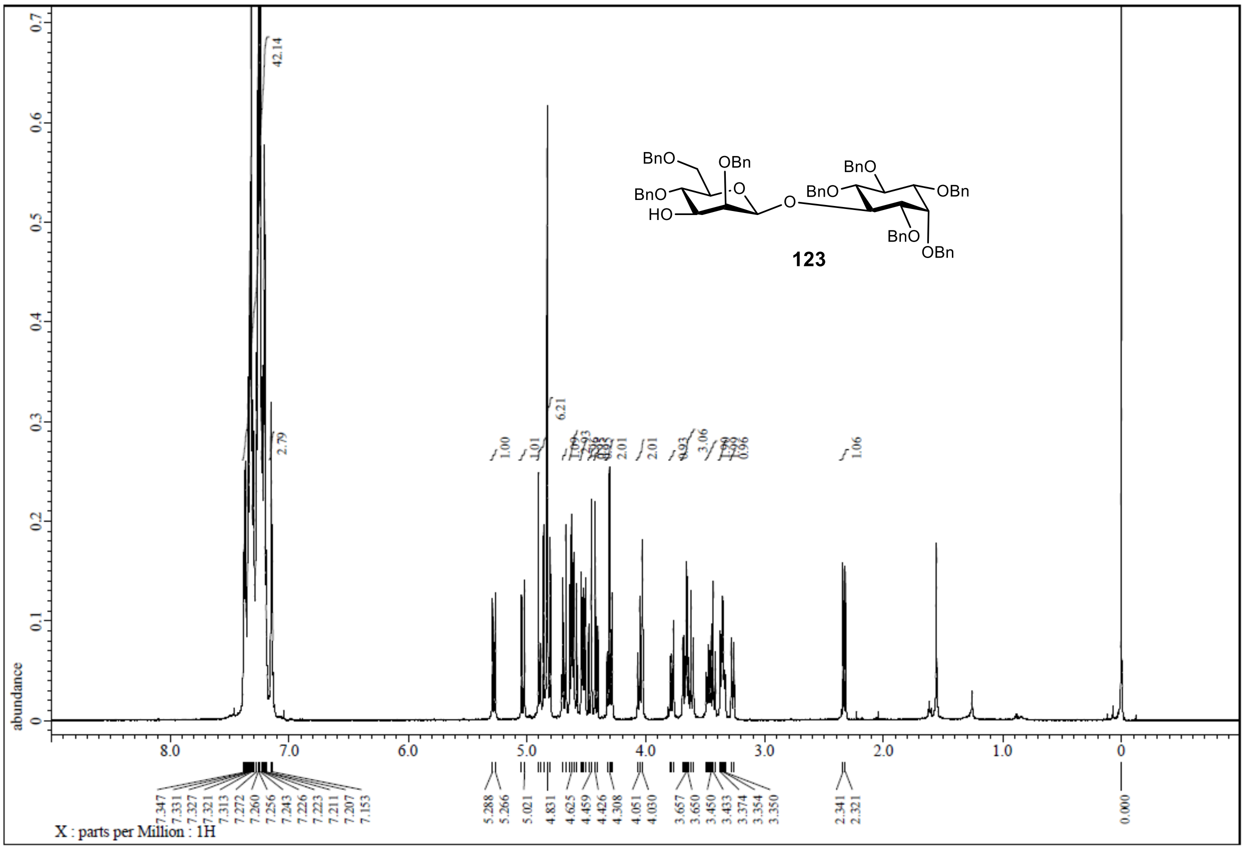

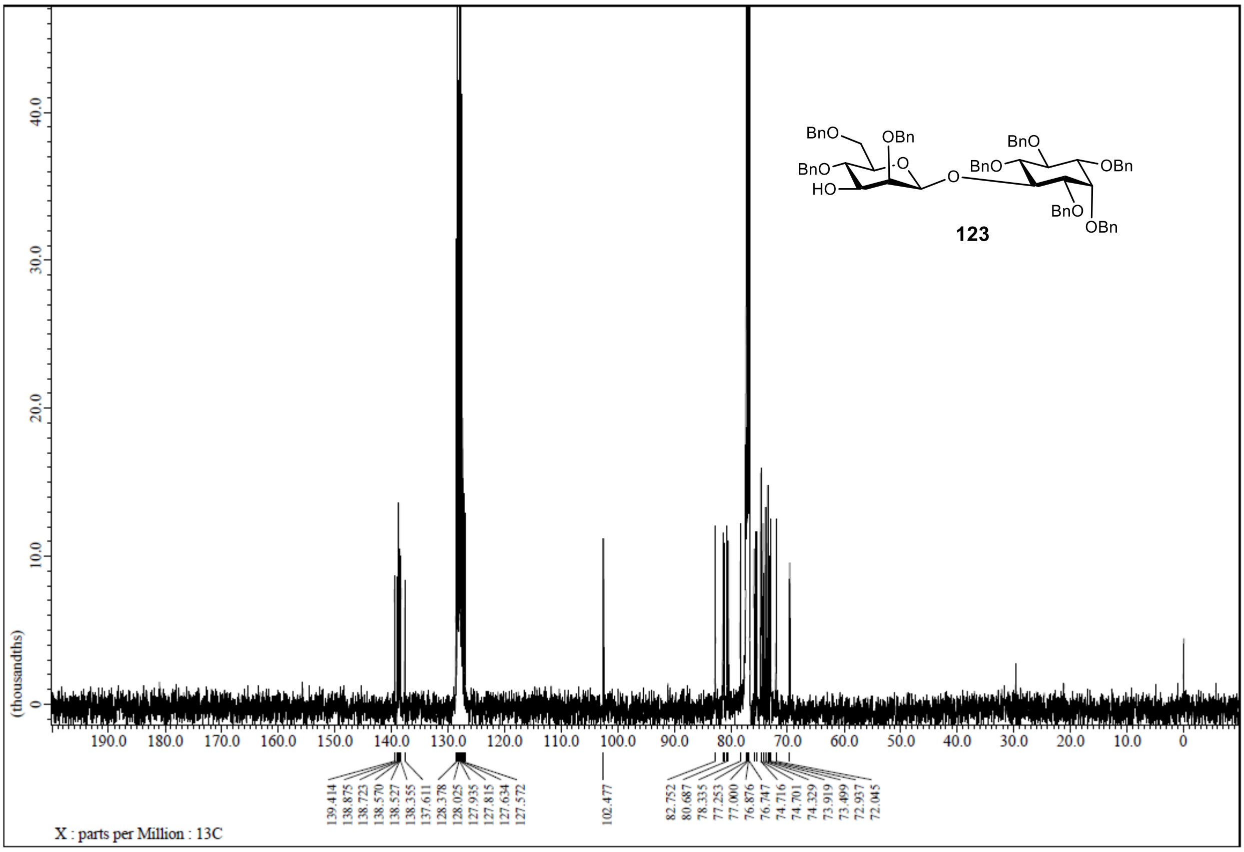

Supplementary Figure 122. ^{1}H and ^{13}C-NMR spectra of compound 123.

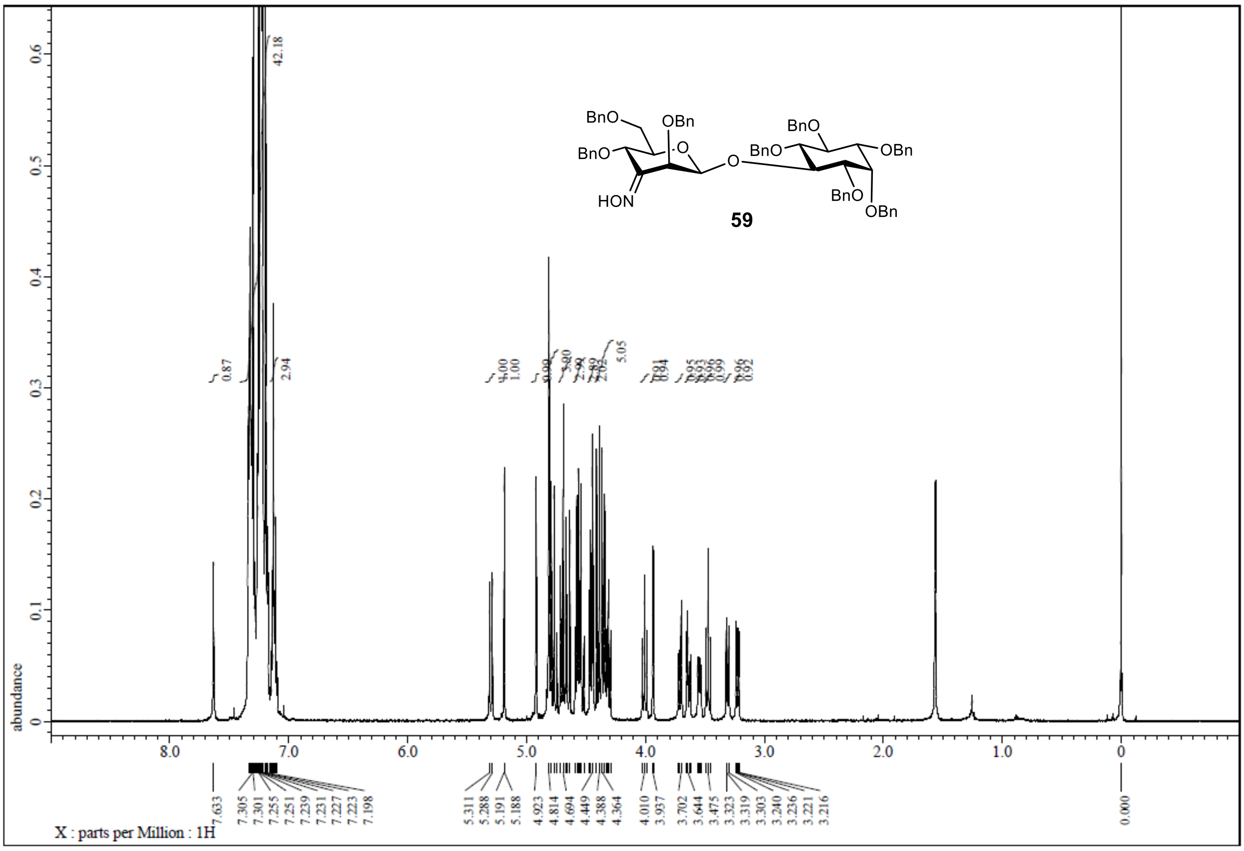

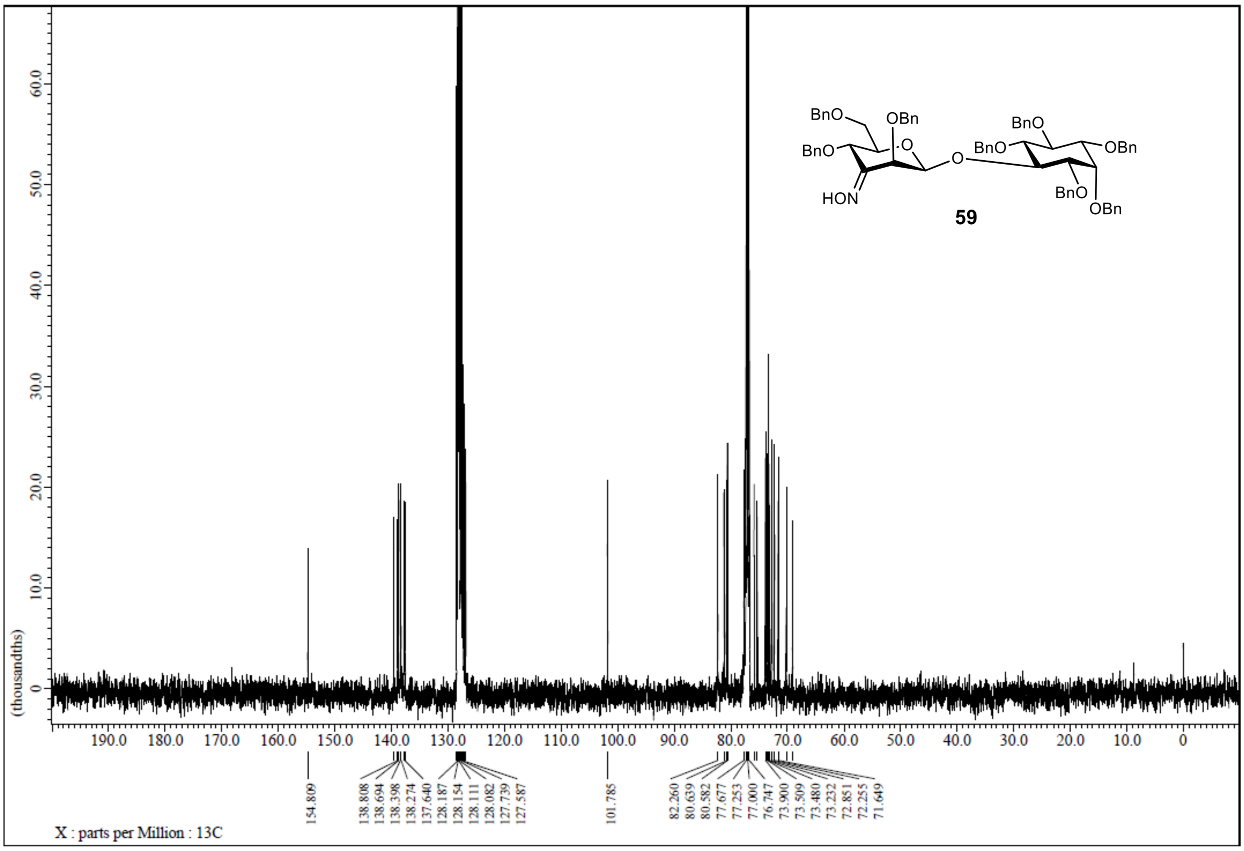

Supplementary Figure 123. ^{1}H and ^{13}C-NMR spectra of compound 59.

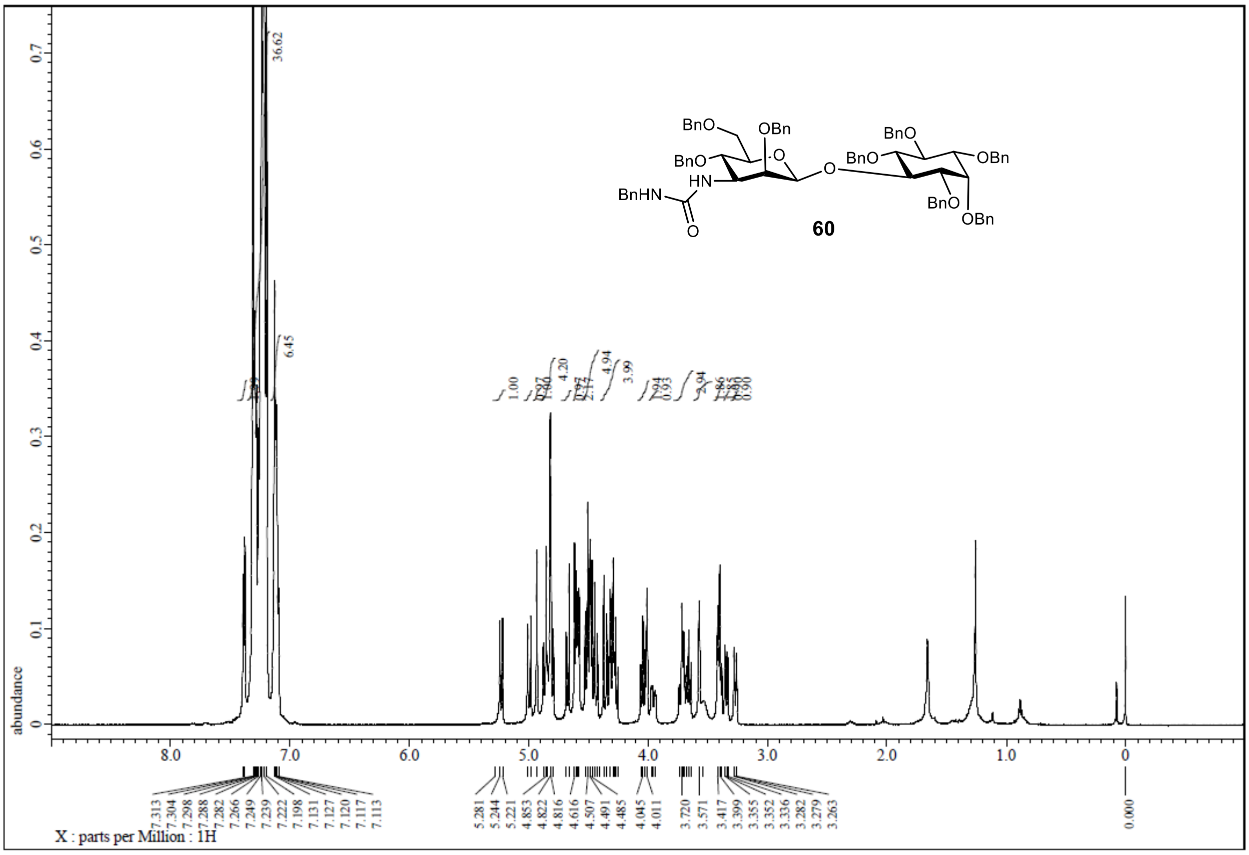

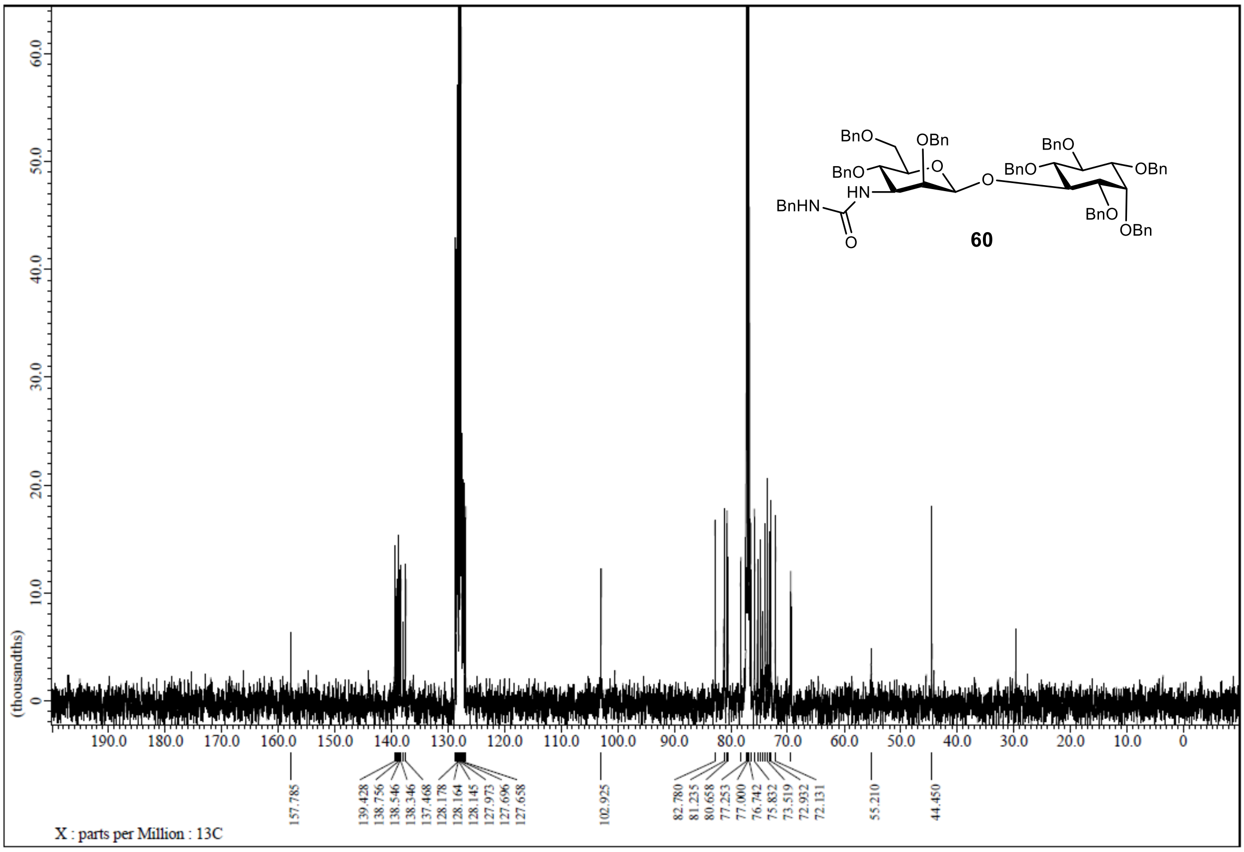

Supplementary Figure 124. ¹H and ¹³C-NMR spectra of compound 60.

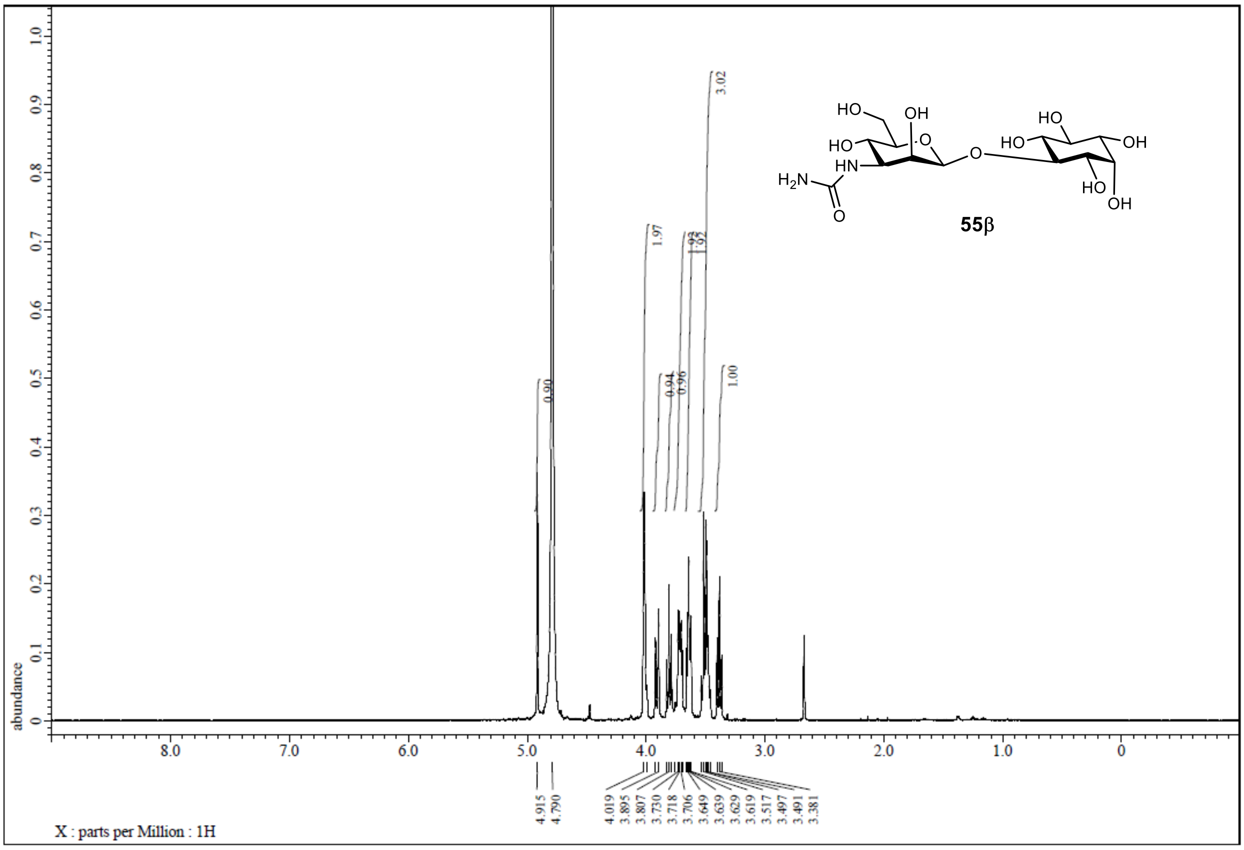

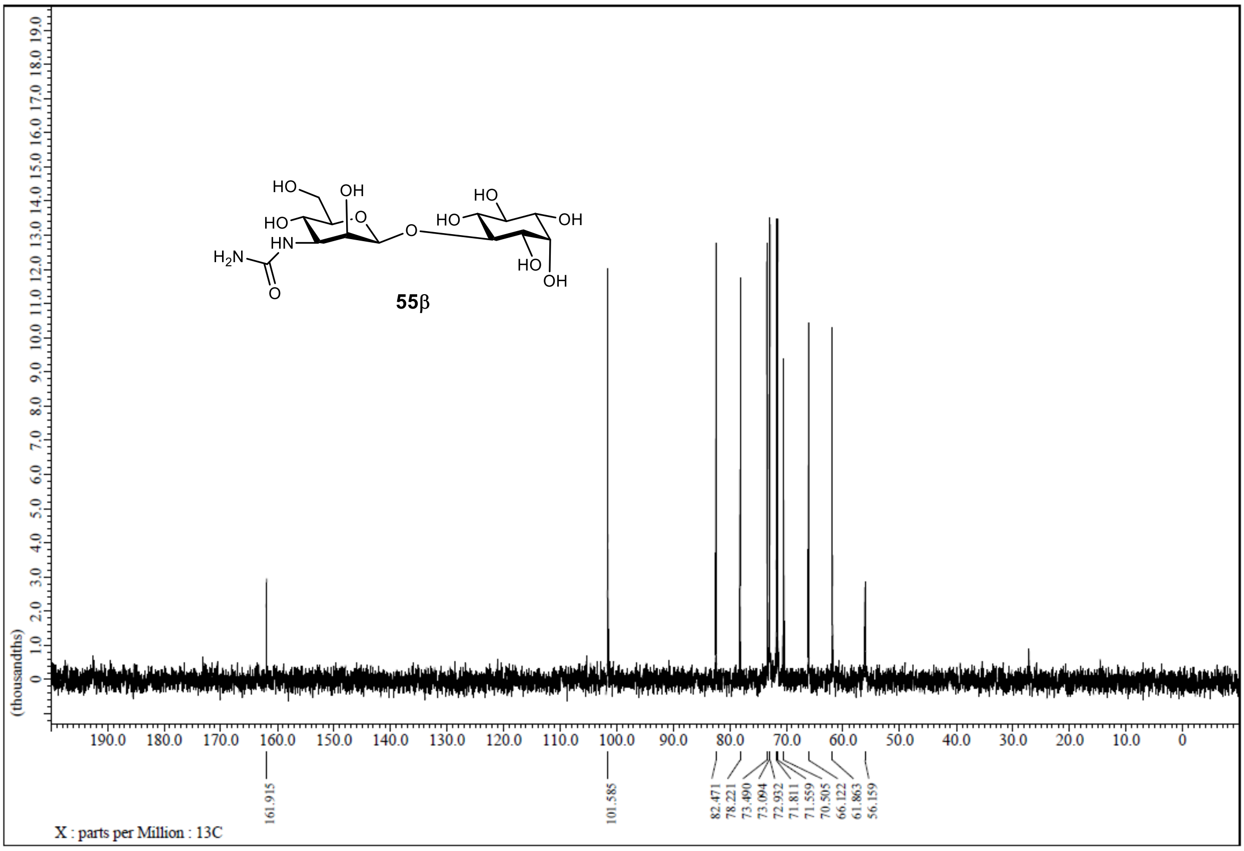

Supplementary Figure 125. ^{1}H and ^{13}C-NMR spectra of compound 55β.

Supplementary References

1. Halcomb, R. L. & Danishefsky, S. J. On the direct epoxidation of glycals: application of a reiterative strategy for the synthesis of β-linked oligosaccharides. *J. Am. Chem. Soc.* **111**, 6661-6666 (1989).
2. Lee, H. W. & Kishi, Y. Synthesis of mono- and unsymmetrical bis-orthoesters of *scyllo*-inositol. *J. Org. Chem.* **50**, 4402-4404 (1985).
3. Keddie, N. S., Slawin, A. M. Z., Lebl, T., Philp, D. & O'Hagan, D. All-*cis* 1,2,3,4,5,6-hexafluorocyclohexane is a facially polarized cyclohexane. *Nat. Chem.* **7**, 483-488 (2015).
4. Duss, M., Capolicchio, S., Linden, A., Ahmed, N. & Jessen, H. J. Desymmetrization of *myo*-inositol derivatives by lanthanide catalyzed phosphitylation with C_2-symmetric phosphites. *Bioorg. Med. Chem.* **23**, 2854-2861 (2015).
5. Timmers, C. M., van Straten, N. C. R., van der Marel, G. A. & van Boom, J. H. An expeditious route to *Streptococci* and *Enterococci* glycolipids *via* ring-opening of 1,2-anhydrosugars with protic acids. *J. Carbohydr. Chem.* **17**, 471-487 (1998).
6. Alberch, L., Cheng, G., Seo, S.-K., Li, X., Boulineau, F. P. & Wei, A. Stereoelectronic factors in the stereoselective epoxidation of glycals and 4-deoxypentenosides. *J. Org. Chem.* **76**, 2532-2547 (2011).
7. Chen, Q., Kong, F. & Cao, L. Synthesis, conformational analysis, and the glycosidic coupling reaction of substituted 2,7-dioxabicyclo[4.1.0]heptanes: 1,2-anhydro-3,4-di-*O*-benzyl-β-L- and β-D-rhamnopyranoses. *Carbohydr. Res.* **240**, 107–117 (1993).
8. Manabe, S., Marui, Y. & Ito, Y. Total synthesis of mannosyl tryptophan and its derivatives. *Chem. Eur. J.* **9**, 1435-1447 (2003).
9. Nashida, J., Nishi, N., Takahashi, Y., Hayashi, C., Igarashi, M., Takahashi, D. & Toshima, K. Systematic and stereoselective total synthesis of mannosylerythritol lipids and evaluation of their antibacterial activity. *J. Org. Chem.* **83**, 7281-7289 (2018).
10. Gallant, M., Link, J. T. & Danishefsky, S. J. A stereoselective synthesis of indole-β-*N*-glycosides: an application to the synthesis of rebeccamycin. *J. Org. Chem.* **58**, 343-349 (1993).
11. Hanessian, S. & Rogel, O. Synthesis of glycophostones: cyclic phosphonate analogues of biologically relevant sugars. *J. Org. Chem.* **65**, 2667-2674 (2000).
12. Hashimoto, Y., Michimuko, C., Yamaguchi, K., Nakajima, M. & Sugiura, M. Selective monoacylation of diols and asymmetric desymmetrization of dialkyl *meso*-tartrates using 2-pyridyl esters as acylating agents and metal carboxylates as catalysts. *J. Org. Chem.* **84**, 9313-9321 (2019).

13. Riley, A. M., Mahon, M. F. & Potter, B. V. L. Rapid synthesis of the enantiomers of *myo*-inositol-1,3,4,5-tetrakisphosphate by direct chiral desymmetrization of *myo*-inositol orthoformate. *Angew. Chem. Int. Ed. Engl.* **36**, 1472-1474 (1997).
14. Larsen, K., Worm-Leonhard, K., Olsen, P., Hoel, A. & Jensen, K. J. Reconsidering glycosylations at high temperature: precise microwave heating. *Org. Biomol. Chem.* **3**, 3966-3970 (2005).
15. Kim, S., Song, S., Lee, T., Jung, S. & Kim, D. Practical synthesis of KRN7000 from phytosphingosine. *Synthesis* **6**, 847-850 (2004).
16. Aiguabella, N., Holland, M. C. & Gilmour, R. Fluorine-directed 1,2-*trans* glycosylation of rare sugars. *Org. Biomol. Chem.* **14**, 5534-5538 (2016).
17. Yadav, R., Ben-Arye, S. L., Subramani, B., Padler-Karavani, V. & Kikkeri, R. Screening of Neu5Acα(2–6)gal isomer preferences of siglecs with a sialic acid microarray. *Org. Biomol. Chem.* **14**, 10812-10815 (2016).
18. Daly, R., Vaz, G., Davies, A. M., Senge, M. O. & Scanlan, E. M. Synthesis and biological evaluation of a library of glycoporphyrin compounds. *Chem. Eur. J.* **18**, 14671-14679 (2012).
19. Hashimoto, Y., Michimuko C., Yamaguchi, K., Nakajima, M. & Sugiura, M. Selective monoacylation of diols and asymmetric desymmetrization of dialkyl *meso*-tartrates using 2-pyridyl esters as acylating agents and metal carboxylates as catalysts. *J. Org. Chem.* **84**, 9313-9321 (2019).
20. Mori, Y., Kobayashi, J., Manabe, K. & Kobayashi, S. Use of boron nolates in water. The first boron enolate-mediated diastereoselective aldol reactions using catalytic boron sources. *Tetrahedron* **58**, 8263-8268 (2002).
21. Ali, A., Gowda, D. C., & Vishwakarma, R. A new approach to construct full-length glycosylphosphatidylinositols of parasitic protozoa and [4-deoxy-Man-III]-GPI analogues. *Chem. Commun.* 519-521 (2005).
22. McGahren, W. J., Hardy, B. A., Morton, G. O., Lovell, F. M., Perkinson, N. A., Hargreaves, R. T., Borders, D. B. & Ellestad, G. A. (β-Lysyloxy)myoinositol guanidino glycoside antibiotics. *J. Org. Chem.* **46**, 792-799 (1981).
23. Schrödinger Release 2017-4: MacroModel, Schrödinger, LLC, New York, NY, 2017.
24. Schrödinger Release 2017-4: Jaguar, Schrödinger, LLC, New York, NY, 2017.
25. Bochevarov, A. D., Harder, E., Hughes, T. F., Greenwood, J. R., Braden, D. A., Philipp, D. M., Rinaldo, D., Halls, M. D., Zhang, J. & Friesner, R. A. Jaguar: A high-performance quantum chemistry software program with strengths in life and materials sciences. *Int. J. Quantum. Chem.* **113**, 2110-2142 (2013).

26. Tanaka, M., Nakagawa, A., Nishi, N., Iijima, K., Sawa, R., Takahashi, D. & Toshima, K. Boronic-acid-catalyzed regioselective and 1,2-*cis*-stereoselective glycosylation of unprotected sugar acceptors via S_Ni-type mechanism. *J. Am. Chem. Soc.* **140**, 3644-3651 (2018).
