# Supplementary material for: Diastereoselective desymmetric 1,2-cis-glycosylation of meso-diols via chirality transfer from a glycosyl donor
Source: Nat Commun. 2020 May 15;11:2431. doi: 10.1038/s41467-020-16365-8 (PMC7229163; doi:10.1038/s41467-020-16365-8)
Supplement: Supplementary file 2 — Description of Additional Supplementary Files [file 41467_2020_16365_MOESM2_ESM.pdf]

Description of Additional Supplementary Files

File Name: Supplementary Data 1
Description: The data of DFT calculations.
