# Supplementary material for: Diastereoselective desymmetric 1,2-cis-glycosylation of meso-diols via chirality transfer from a glycosyl donor
Source: Nat Commun. 2020 May 15;11:2431. doi: 10.1038/s41467-020-16365-8 (PMC7229163; doi:10.1038/s41467-020-16365-8)
Supplement: Supplementary file 3 — Supplementary Data 1 [file 41467_2020_16365_MOESM3_ESM.zip › 219252_3_data_set_4570823_q990cr.docx]

**The data of DFT calculations**

**TS-Glc-Concave**

C1 -3.3010460000 3.1998720000 6.9058550000

C2 -4.2540830000 4.2835380000 7.4873970000

C3 -3.5710560000 5.6440490000 7.2645330000

C4 -2.2910610000 5.6693980000 8.1184260000

C5 -1.3638360000 4.5537590000 7.5603760000

C6 -2.0167730000 3.1737310000 7.7455320000

O7 -3.0026410000 3.5410230000 5.5316100000

H8 -3.7796220000 2.2180370000 6.8818560000

O9 -4.5003000000 4.0561250000 8.8534540000

H10 -5.1979030000 4.2490990000 6.9283720000

H11 -4.2376300000 6.4683410000 7.5287250000

H12 -1.7829510000 6.6327890000 7.9837120000

O13 -2.5885160000 5.4871110000 9.4928270000

O14 -1.1565360000 4.8237690000 6.1547420000

H15 -0.3724360000 4.5812550000 8.0222760000

H16 -2.3366240000 3.0780650000 8.7826340000

O17 -1.1530680000 2.0617640000 7.5728980000

O18 -3.2327930000 5.8208410000 5.8805080000

C20 -2.3777160000 4.7962210000 5.4463730000

H20 -2.1490590000 4.9805140000 4.3983550000

B21 -3.8918800000 4.8948160000 9.8762070000

C23 -0.6038620000 1.8010590000 6.2823290000

H24 -1.3810610000 1.6048890000 5.5358970000

H25 0.0220380000 2.6259050000 5.9247400000

H26 0.0123430000 0.9052660000 6.4048990000

C26 -6.8252610000 7.9042910000 11.3202340000

C27 -5.5579100000 8.3366500000 10.9375920000

C28 -4.6389740000 7.3893910000 10.4888850000

C29 -4.9518980000 6.0207480000 10.4109300000

C30 -6.2547230000 5.6375140000 10.7877950000

C31 -7.1910090000 6.5584710000 11.2453730000

N32 -7.7949480000 8.8863340000 11.8147190000

H33 -5.3129140000 9.3906180000 10.9985660000

H34 -3.6469150000 7.7208190000 10.1914330000

H35 -6.5364820000 4.5902860000 10.7131760000

H36 -8.1905430000 6.2612810000 11.5412770000

O37 -8.9058710000 8.4770220000 12.1583240000

O38 -7.4456040000 10.0679070000 11.8657520000

C22 -2.5116670000 5.4095670000 12.7785820000

C40 -2.7290540000 4.0344720000 12.0848260000

C24 -1.5370030000 3.5159620000 11.3534510000

C25 -0.2994640000 5.5529020000 11.5530190000

H43 -2.9971930000 3.3192310000 12.8754110000

H27 -0.7715320000 6.1685910000 10.7837460000

O29 -3.6345530000 3.8951390000 11.0255090000

C46 -1.0143120000 5.7273220000 12.8996810000

H31 -0.5546100000 5.0486960000 13.6314930000

H32 -2.9845970000 6.1985560000 12.1853040000

O33 -0.4871830000 4.1627050000 11.0528290000

H50 -1.5780560000 2.5176650000 10.9217040000

C35 1.1973600000 5.7774800000 11.5996080000

H52 1.6338450000 5.5291710000 10.6187250000

H37 1.3672880000 6.8472890000 11.7894690000

O54 1.7440970000 4.9725750000 12.6202380000

O39 -0.8030520000 7.0771170000 13.2566870000

O40 -3.0247940000 5.3911460000 14.0973230000

C57 3.1508370000 5.1047100000 12.7330790000

H47 3.4405760000 6.1392670000 12.9713140000

H48 3.4677370000 4.4470500000 13.5455990000

C49 -0.5802980000 7.3040700000 14.6481270000

H61 -1.4489660000 7.0040230000 15.2413630000

H51 0.3089300000 6.7590750000 14.9955130000

C52 -4.4402840000 5.5509230000 14.1751160000

H53 -4.7555910000 6.5128080000 13.7515640000

H54 -4.9706070000 4.7460520000 13.6489140000

H73 3.6587800000 4.8018030000 11.8052000000

H74 -0.4101850000 8.3777420000 14.7570120000

H75 -4.6956240000 5.5164490000 15.2366300000

**TS-Glc-Convex**

C1 -3.9689630000 2.6600820000 7.1540540000

C2 -4.6612420000 3.9384790000 7.7015630000

C3 -3.9243640000 5.1406530000 7.0843170000

C4 -2.4801660000 5.1157670000 7.6092610000

C5 -1.8178940000 3.8293840000 7.0389350000

C6 -2.5077540000 2.5893700000 7.6292230000

O7 -4.0206500000 2.7337030000 5.7081530000

H8 -4.5158810000 1.7555670000 7.4326750000

O9 -4.6541310000 4.0004810000 9.1174100000

H10 -5.7033260000 3.9297530000 7.3538160000

H11 -4.4093670000 6.0865480000 7.3405410000

H12 -1.9322950000 5.9854910000 7.2254460000

O13 -2.4465390000 5.1208480000 9.0152030000

O14 -1.9716910000 3.8492630000 5.5978090000

H15 -0.7428800000 3.8153640000 7.2319210000

H16 -2.4949260000 2.6875160000 8.7154020000

O17 -1.8617940000 1.3462920000 7.4198000000

O18 -3.9524860000 5.0497800000 5.6538490000

C20 -3.3253300000 3.8619290000 5.2299010000

H20 -3.3731110000 3.8378940000 4.1427020000

B21 -3.5986560000 4.7663820000 9.8140330000

C23 -1.5954780000 0.8942030000 6.0948000000

H24 -2.5108990000 0.7647370000 5.5071930000

H25 -0.9246720000 1.5697180000 5.5525600000

H26 -1.1047810000 -0.0760580000 6.2167270000

C26 -2.1058420000 2.6308660000 13.4017360000

C27 -3.0575870000 2.0079320000 12.5993320000

C28 -3.5287290000 2.6857870000 11.4764510000

C29 -3.0785600000 3.9740970000 11.1379980000

C30 -2.0999180000 4.5532580000 11.9696840000

C31 -1.6098000000 3.8996750000 13.0952740000

N32 -1.6146550000 1.9377110000 14.5967180000

H33 -3.4093780000 1.0167240000 12.8601660000

H34 -4.2637440000 2.2007030000 10.8400170000

H35 -1.7115100000 5.5372200000 11.7200810000

H36 -0.8572630000 4.3458880000 13.7351550000

O37 -0.7768990000 2.5142260000 15.2937790000

O38 -2.0699550000 0.8189700000 14.8440010000

C22 -5.9539680000 5.4472210000 12.0201170000

C40 -5.4675460000 6.3464720000 10.8449840000

C24 -6.3031670000 6.2542360000 9.6166980000

C25 -7.4899990000 4.3181630000 10.3480420000

H43 -5.5190850000 7.3845880000 11.2065110000

H27 -6.7249730000 3.5588120000 10.1642380000

O29 -4.2143860000 6.1488670000 10.2460040000

C46 -7.3673370000 4.9029630000 11.7616420000

H31 -8.0965800000 5.7183380000 11.8647690000

H32 -5.2762170000 4.5954670000 12.1331110000

O33 -7.1765110000 5.3746500000 9.3434620000

H50 -6.1112110000 6.9500060000 8.8019520000

C35 -8.8681360000 3.8003160000 9.9959460000

H52 -8.8874080000 3.5077720000 8.9336650000

H37 -9.0473230000 2.9004840000 10.6022270000

O54 -9.8160250000 4.8086560000 10.2657670000

O39 -7.6725080000 3.8357810000 12.6338600000

O40 -6.0317540000 6.1946900000 13.2210330000

C57 -11.1441040000 4.4083390000 9.9734740000

H47 -11.4427290000 3.5354420000 10.5732950000

H48 -11.7921800000 5.2522340000 10.2203590000

C49 -8.4243280000 4.1999990000 13.7910910000

H61 -7.8729440000 4.9113910000 14.4129310000

H51 -9.3934060000 4.6338650000 13.5064280000

C52 -4.7738870000 6.4031200000 13.8583310000

H53 -4.3033310000 5.4506920000 14.1333110000

H54 -4.0800260000 6.9625380000 13.2157100000

H73 -11.2644390000 4.1608230000 8.9080840000

H74 -8.5923660000 3.2748050000 14.3470680000

H75 -4.9776440000 6.9858440000 14.7595320000

**TS-Rha-Concave**

O1 -1.2333140000 4.5805400000 10.7496910000

C2 -0.6191620000 3.7894880000 11.8725910000

C3 -1.4969740000 2.6526800000 12.4373830000

C4 -2.6200510000 2.2148450000 11.4873400000

H5 0.2353230000 3.3340070000 11.3680560000

H6 -1.9834320000 2.9911060000 13.3593090000

O7 -0.5596270000 1.6248960000 12.7063050000

H8 -2.1877400000 1.7001490000 10.6103660000

O9 -3.4402450000 1.3359200000 12.2188210000

C10 -0.1808460000 4.8083190000 12.9062990000

H11 0.3555420000 4.2778100000 13.6995360000

H12 0.4906520000 5.5532780000 12.4703230000

H13 -1.0503410000 5.3102380000 13.3411110000

C14 -4.5382120000 0.7824800000 11.5034030000

H15 -4.9456870000 -0.0094590000 12.1351810000

H16 -5.3220680000 1.5285790000 11.3221370000

H17 -4.2160240000 0.3464990000 10.5458250000

C18 -0.8588530000 0.8043930000 13.8352470000

H19 0.0057750000 0.1500230000 13.9700060000

H20 -0.9949890000 1.4131510000 14.7405760000

H21 -1.7584910000 0.2054100000 13.6688680000

C22 -2.4262980000 4.4148020000 10.3487700000

C23 -3.3725240000 3.4740330000 11.0160560000

H24 -2.6907810000 5.0461780000 9.5073980000

H25 -4.2037800000 3.2304500000 10.3474390000

O26 -3.7824290000 4.2206190000 12.1364110000

C1 -6.9316110000 5.3334290000 9.1311650000

C28 -6.8903300000 5.6099990000 10.6625030000

C29 -6.4481090000 7.0778560000 10.8293850000

C30 -4.9991910000 7.1905180000 10.3106310000

C5 -5.0582060000 6.8835890000 8.7862240000

C6 -5.5107360000 5.4320490000 8.5582640000

O33 -7.7858480000 6.3221770000 8.5117740000

H34 -7.3794280000 4.3609610000 8.9105220000

O35 -6.0021510000 4.7293820000 11.3254470000

H10 -7.9071110000 5.4867790000 11.0576500000

H37 -6.5206830000 7.3982380000 11.8693300000

H38 -4.6385860000 8.2189610000 10.4390470000

O13 -4.1453790000 6.2900310000 11.0018750000

O14 -6.0017690000 7.7932580000 8.1801280000

H41 -4.0979170000 7.0799200000 8.2986210000

H42 -4.8888000000 4.7852780000 9.1733430000

O17 -5.3065170000 4.9193110000 7.2522420000

O18 -7.3076360000 7.9529490000 10.0851220000

C20 -7.2931830000 7.6232890000 8.7244100000

H46 -7.9566810000 8.3139260000 8.2074870000

B21 -4.7903040000 5.3017280000 11.9339170000

C48 -6.0245000000 5.4898730000 6.1580340000

H49 -7.1079500000 5.3864670000 6.2785110000

H50 -5.7882310000 6.5499150000 6.0174460000

H26 -5.7030570000 4.9265900000 5.2769420000

C26 -5.3226490000 6.8192160000 16.0647340000

C27 -4.3269110000 7.3750090000 15.2624280000

C54 -4.1854720000 6.9072790000 13.9587350000

C55 -5.0129220000 5.8969960000 13.4369830000

C56 -6.0058250000 5.3717990000 14.2818960000

C31 -6.1711910000 5.8200830000 15.5894860000

N32 -5.4796540000 7.2970150000 17.4421620000

H33 -3.6914600000 8.1548270000 15.6659270000

H60 -3.4191770000 7.3415730000 13.3207200000

H35 -6.6610610000 4.5931350000 13.9001550000

H36 -6.9350750000 5.4146770000 16.2426870000

O37 -6.3716190000 6.7951850000 18.1296450000

O38 -4.7087200000 8.1733920000 17.8407440000

**TS-Rha-Convex**

C1 -3.8061080000 2.7449500000 7.2211810000

C2 -4.5961590000 4.0488170000 7.5347710000

C3 -3.8368910000 5.2020340000 6.8563280000

C4 -2.4684370000 5.3123690000 7.5475780000

C5 -1.7004100000 3.9966330000 7.2258990000

C6 -2.4053350000 2.7838780000 7.8507330000

O7 -3.6916010000 2.6619290000 5.7782580000

H8 -4.3562130000 1.8550030000 7.5356250000

O9 -4.7710870000 4.3195320000 8.9134820000

H10 -5.5894010000 3.9404170000 7.0783360000

H11 -4.3890610000 6.1443940000 6.9382150000

H12 -1.9009060000 6.1402510000 7.1052560000

O13 -2.6320740000 5.5705760000 8.9238330000

O14 -1.6790460000 3.8562030000 5.7811680000

H15 -0.6560690000 4.0535670000 7.5411680000

H16 -2.5148860000 2.9653100000 8.9189850000

O17 -1.6886380000 1.5628950000 7.8238830000

O18 -3.6906940000 4.9539100000 5.4534670000

C20 -2.9769570000 3.7561790000 5.2544700000

H20 -2.8862950000 3.6032810000 4.1806480000

B21 -3.6943670000 4.9649270000 9.7329070000

C23 -1.2960170000 0.9779340000 6.5841590000

H24 -2.1543810000 0.7294880000 5.9508380000

H25 -0.6165450000 1.6228700000 6.0165980000

H26 -0.7722470000 0.0572750000 6.8579040000

C26 -2.2307890000 2.4655620000 13.0901390000

C27 -3.5000110000 2.2221280000 12.5660290000

C28 -3.9372720000 2.9940080000 11.4929000000

C29 -3.1378590000 4.0043770000 10.9287460000

C30 -1.8626590000 4.2069870000 11.4846950000

C31 -1.3994520000 3.4504770000 12.5577620000

N32 -1.7609730000 1.6678350000 14.2265830000

H33 -4.1134800000 1.4402710000 12.9984150000

H34 -4.9197680000 2.8009900000 11.0686870000

H35 -1.2208800000 4.9737390000 11.0593160000

H36 -0.4165480000 3.6069590000 12.9866610000

O37 -0.6330150000 1.8986640000 14.6678290000

O38 -2.5211920000 0.8110240000 14.6842990000

O1 -7.2603900000 6.2844490000 10.2547370000

C40 -7.2626810000 6.8861510000 11.6326540000

C41 -6.0450660000 7.7724220000 11.9696650000

C42 -5.2916270000 8.2835130000 10.7337100000

H5 -8.1434470000 7.5296530000 11.5844440000

H6 -5.3224640000 7.1912470000 12.5538620000

O45 -6.6093230000 8.8122000000 12.7490610000

H46 -5.9124580000 9.0244880000 10.1980710000

O47 -4.1115040000 8.8842590000 11.2107880000

C10 -7.4672190000 5.7260300000 12.5875820000

H49 -7.5612080000 6.1313250000 13.6000800000

H50 -8.3786620000 5.1707360000 12.3490350000

H13 -6.6082180000 5.0490230000 12.5552580000

C14 -3.2616930000 9.4464700000 10.2183130000

H53 -2.5163010000 10.0380550000 10.7542780000

H54 -2.7469060000 8.6692870000 9.6397860000

H17 -3.8195040000 10.1070530000 9.5370540000

C18 -5.7693020000 9.3272360000 13.7818020000

H19 -6.3870880000 10.0237030000 14.3537990000

H58 -5.4229030000 8.5229350000 14.4464160000

H21 -4.9010300000 9.8490540000 13.3699080000

C22 -6.2913860000 6.4050470000 9.4423210000

C61 -5.0186380000 7.0857960000 9.8056480000

H62 -6.4721520000 5.9244660000 8.4867690000

H63 -4.4622510000 7.3746330000 8.9070910000

O26 -4.3893890000 6.0556960000 10.5268420000

**TS-Man-Concave**

C1 2.3912810000 -2.3533190000 7.9795910000

C2 1.5400900000 -1.1576560000 8.4964850000

C3 2.0749190000 0.0974590000 7.7772160000

C4 3.5249960000 0.3201350000 8.2479990000

C5 4.3497970000 -0.8935550000 7.7288920000

C6 3.8567430000 -2.1786310000 8.4092980000

O7 2.3009510000 -2.3810170000 6.5383130000

H8 1.9903770000 -3.3117360000 8.3241240000

O9 1.6350960000 -1.0129760000 9.9058180000

H10 0.4951770000 -1.3318970000 8.2097320000

H11 1.4524970000 0.9684590000 7.9881900000

H12 3.9282310000 1.2280760000 7.7813690000

O13 3.5899450000 0.4332050000 9.6564310000

O14 4.1615510000 -0.9891790000 6.2976480000

H15 5.4213360000 -0.7481960000 7.8880490000

H16 3.8697270000 -1.9867580000 9.4801920000

O17 4.6782810000 -3.3273800000 8.2843370000

O18 2.0275860000 -0.0892160000 6.3553790000

C20 2.8063880000 -1.1896790000 5.9751030000

H20 2.7380280000 -1.2872350000 4.8933030000

B21 2.3718730000 0.1808410000 10.4291340000

C23 4.9606600000 -3.8488570000 6.9860340000

H24 4.0527680000 -4.1649620000 6.4615850000

H25 5.4930250000 -3.1259930000 6.3588680000

H26 5.6016830000 -4.7186450000 7.1580520000

C26 -0.2048300000 3.7309720000 11.1049860000

C27 1.1664760000 3.9051920000 10.9170670000

C28 1.9562440000 2.7832420000 10.6800630000

C29 1.4106410000 1.4882670000 10.6253350000

C30 0.0214140000 1.3635520000 10.8080560000

C31 -0.7931260000 2.4678030000 11.0488820000

N32 -1.0473130000 4.9002980000 11.3724600000

H33 1.5878140000 4.9026620000 10.9632150000

H34 3.0262750000 2.9072600000 10.5353470000

H35 -0.4292280000 0.3753080000 10.7517400000

H36 -1.8638540000 2.3723890000 11.1880720000

O37 -2.2510590000 4.7119590000 11.5664900000

O38 -0.5078940000 6.0084970000 11.3951030000

C39 1.9478670000 -1.8413520000 14.2530070000

C40 3.2326780000 -1.0232200000 14.5650810000

C41 4.3189220000 -1.1035590000 13.4742180000

C42 3.6962910000 -1.1846680000 12.0824990000

C43 2.7439740000 -2.3194280000 12.0114470000

H6 1.8419820000 -2.6278410000 15.0020510000

H7 2.9441340000 0.0300950000 14.6608130000

H46 4.8890770000 -2.0329270000 13.6429020000

H9 4.4712870000 -1.2250990000 11.3133620000

H48 2.5670970000 -2.9101060000 11.1184760000

O11 2.7558830000 -0.1535470000 11.8511790000

O12 1.9761370000 -2.6246240000 12.9824820000

O51 5.1714580000 0.0137420000 13.5010870000

O52 3.8321300000 -1.4918940000 15.7628280000

C15 0.6583090000 -1.0178900000 14.1680610000

H54 0.7772620000 -0.2252450000 13.4176730000

H17 -0.1754730000 -1.6696360000 13.8624460000

O56 0.4537070000 -0.4914080000 15.4600630000

C19 -0.6348550000 0.4209780000 15.5152500000

C58 3.2826340000 -0.9586220000 16.9662520000

H59 3.8711410000 -1.3840500000 17.7823940000

H60 3.3684530000 0.1365480000 16.9872380000

H61 2.2272490000 -1.2262560000 17.0927180000

C62 6.3038290000 -0.1011820000 14.3560460000

H28 6.0169310000 -0.1701000000 15.4107440000

H29 6.8982050000 0.8006040000 14.1935530000

H30 6.9099400000 -0.9819070000 14.0960590000

H66 -0.6813670000 0.7942900000 16.5405200000

H67 -1.5844990000 -0.0758080000 15.2682130000

H68 -0.4854710000 1.2634450000 14.8257290000

**TS-Man-Convex**

C1 -4.4137590000 2.5360820000 7.5155430000

C2 -5.1220460000 3.9040320000 7.7421450000

C3 -4.5535660000 4.8738020000 6.6936520000

C4 -3.0636650000 5.0716670000 7.0199220000

C5 -2.3713970000 3.6932230000 6.8139910000

C6 -2.9099060000 2.6502580000 7.8058330000

O7 -4.6162980000 2.1691640000 6.1262660000

H8 -4.8731150000 1.7430470000 8.1100340000

O9 -4.9538830000 4.4249450000 9.0398820000

H10 -6.1940220000 3.7571890000 7.5637290000

H11 -5.0932440000 5.8261780000 6.7109530000

H12 -2.6162990000 5.7681940000 6.2982440000

O13 -2.9118920000 5.6210300000 8.3168960000

O14 -2.6664950000 3.2759580000 5.4573410000

H15 -1.2837420000 3.7745920000 6.8786310000

H16 -2.7868970000 3.0406440000 8.8152400000

O17 -2.1879490000 1.4330810000 7.8639710000

O18 -4.7133100000 4.3546130000 5.3684310000

C20 -4.0518910000 3.1169590000 5.2575660000

H20 -4.1956960000 2.7583480000 4.2400310000

B21 -3.7868660000 5.1928720000 9.4504740000

C23 -2.1349550000 0.5666410000 6.7329040000

H24 -3.1256360000 0.2001300000 6.4431620000

H25 -1.6701640000 1.0446430000 5.8639330000

H26 -1.5181970000 -0.2790790000 7.0513990000

C26 -1.5824150000 3.4657200000 12.8842160000

C27 -2.9371850000 3.1753500000 12.7246600000

C28 -3.6059610000 3.6968410000 11.6210000000

C29 -2.9540620000 4.5030840000 10.6710180000

C30 -1.5834240000 4.7531700000 10.8640190000

C31 -0.8891610000 4.2458670000 11.9594530000

N32 -0.8698960000 2.9428300000 14.0540530000

H33 -3.4395260000 2.5573930000 13.4597650000

H34 -4.6615200000 3.4773960000 11.4861610000

H35 -1.0493310000 5.3512830000 10.1295310000

H36 0.1676240000 4.4352970000 12.1081950000

O37 0.3169520000 3.2502940000 14.1921700000

O38 -1.4958890000 2.2309210000 14.8420200000

C39 -3.4007030000 9.3116720000 10.7946460000

C40 -4.8671230000 9.0733870000 11.2527460000

C41 -5.7685750000 8.4015380000 10.1954740000

C42 -4.9713140000 7.4174100000 9.3422490000

C43 -3.8009670000 8.0971710000 8.7333780000

H6 -3.2037810000 10.3849950000 10.7977540000

H7 -4.8390490000 8.4122830000 12.1270030000

H46 -6.1503230000 9.2005410000 9.5358440000

H9 -5.6195140000 6.9414030000 8.6004780000

H48 -3.4095750000 7.8818240000 7.7448550000

O11 -4.2538780000 6.4904030000 10.1237810000

O12 -3.1056890000 8.9519780000 9.3756820000

O51 -6.8312470000 7.6851670000 10.7701530000

O52 -5.4835160000 10.3106960000 11.5753740000

C15 -2.3334830000 8.5835960000 11.6188110000

H54 -2.5440270000 7.5067250000 11.6287580000

H17 -1.3434190000 8.7441670000 11.1632540000

O56 -2.4028670000 9.1455950000 12.9108680000

C19 -1.5695010000 8.4740930000 13.8471180000

C58 -5.2112190000 10.7967690000 12.8881600000

H59 -5.7711230000 11.7293200000 12.9889950000

H60 -5.5516670000 10.0828910000 13.6511430000

H61 -4.1432470000 10.9877760000 13.0453570000

C62 -7.9647740000 8.4564360000 11.1492690000

H28 -7.7535180000 9.1134840000 12.0000180000

H29 -8.7392100000 7.7375630000 11.4260280000

H30 -8.3254700000 9.0721360000 10.3120780000

H66 -1.7274420000 8.9584920000 14.8131290000

H67 -0.5088050000 8.5587290000 13.5694200000

H68 -1.8310810000 7.4097410000 13.9241450000

**TS-Fuc-Concave**

O1 -3.9931320000 1.6678410000 9.5700520000

C2 -5.3998460000 1.1469400000 9.6612070000

C3 -5.6269870000 0.6429420000 11.0878160000

C4 -5.4967310000 1.8185730000 12.0716700000

H5 -6.0002170000 2.0376760000 9.4639300000

H6 -6.6452260000 0.2318270000 11.1528770000

O7 -4.6532400000 -0.3619720000 11.3195550000

H8 -6.2451250000 2.5779880000 11.8151960000

O9 -5.7220180000 1.3099620000 13.3684880000

C10 -5.5165730000 0.1019760000 8.5728330000

H11 -6.5590210000 -0.2269580000 8.5070530000

H12 -5.2290940000 0.5199000000 7.6039990000

H13 -4.8875360000 -0.7615020000 8.7993490000

O14 -3.9048740000 3.8213560000 11.8914650000

C15 -3.4348670000 2.1423700000 10.6036820000

C16 -4.0788590000 2.4325130000 11.9211080000

H17 -2.4104700000 2.4732490000 10.4417680000

H18 -3.4528060000 2.0103130000 12.7187550000

C19 -5.9395280000 2.3044030000 14.3695640000

H20 -6.0933350000 1.7642840000 15.3064070000

H21 -5.0768640000 2.9744360000 14.4733870000

H22 -6.8268130000 2.9078050000 14.1425310000

C23 -5.0192690000 -1.3926950000 12.2382140000

H24 -4.2133850000 -2.1296860000 12.2008930000

H25 -5.1275350000 -1.0046610000 13.2535310000

H26 -5.9585410000 -1.8735270000 11.9292960000

C1 -4.2632430000 4.7315160000 7.7829680000

C28 -5.4247340000 5.0244740000 8.7743800000

C29 -5.3564230000 6.5277560000 9.0963460000

C30 -4.0201840000 6.7909390000 9.8129690000

C5 -2.8911310000 6.5023970000 8.7814340000

C6 -2.9049330000 5.0040420000 8.4503850000

O33 -4.4400330000 5.6007820000 6.6404480000

H34 -4.3082530000 3.7099540000 7.3942330000

O35 -5.3429210000 4.2432400000 9.9555180000

H10 -6.3700610000 4.8053820000 8.2616120000

H37 -6.2032260000 6.8376680000 9.7129950000

H38 -3.9602460000 7.8470970000 10.1055240000

O13 -3.8809800000 5.9701490000 10.9474810000

O40 -3.1403780000 7.2880840000 7.5926470000

H15 -1.9149700000 6.8165190000 9.1584950000

H16 -2.8799410000 4.4858600000 9.4082690000

O17 -1.7749310000 4.4797760000 7.7725000000

O18 -5.4394910000 7.2869070000 7.8807820000

C20 -4.3803160000 6.9593780000 7.0204210000

H46 -4.4946940000 7.5542780000 6.1162120000

B21 -4.7975850000 4.8628850000 11.1882520000

C48 -1.4250570000 5.0053320000 6.4932200000

H49 -2.2171060000 4.8517430000 5.7522340000

H50 -1.1916450000 6.0745630000 6.5370940000

H51 -0.5310520000 4.4535070000 6.1876130000

C26 -7.9208400000 6.0969100000 14.1009980000

C27 -6.5752100000 6.3578190000 14.3679540000

C54 -5.6222170000 5.9637090000 13.4353830000

C55 -5.9724620000 5.3026450000 12.2409380000

C56 -7.3412830000 5.0789020000 12.0113270000

C31 -8.3209050000 5.4675090000 12.9245050000

N32 -8.9365810000 6.4946700000 15.0805790000

H33 -6.3033050000 6.8607610000 15.2887980000

H60 -4.5739180000 6.1755630000 13.6290710000

H35 -7.6480900000 4.5916950000 11.0882550000

H36 -9.3757840000 5.2950160000 12.7446680000

O37 -10.1166170000 6.2471900000 14.8211650000

O38 -8.5556960000 7.0499360000 16.1132480000

**TS-Fuc-Convex**

C1 -3.8591950000 2.9148490000 7.2125550000

C2 -4.6297860000 4.0918760000 7.8776860000

C3 -4.0030110000 5.3878430000 7.3433670000

C4 -2.5537110000 5.4454990000 7.8566080000

C5 -1.8024500000 4.2472630000 7.2136470000

C6 -2.3987890000 2.9126410000 7.6912030000

O7 -3.9126150000 3.1047290000 5.7761090000

H8 -4.3435210000 1.9565290000 7.4126480000

O9 -4.5542640000 4.0330240000 9.2792720000

H10 -5.6800600000 4.0332500000 7.5655960000

H11 -4.5808120000 6.2570360000 7.6698690000

H12 -2.0781290000 6.3683270000 7.4969320000

O13 -2.5066220000 5.4349750000 9.2729720000

O14 -1.9514540000 4.3739580000 5.7783310000

H15 -0.7286190000 4.2915120000 7.4133630000

H16 -2.4055770000 2.9093280000 8.7817640000

O17 -1.6336480000 1.7616150000 7.3810080000

O18 -4.0171060000 5.4153650000 5.9107350000

C20 -3.3069830000 4.3120750000 5.3981370000

H20 -3.3444590000 4.3757910000 4.3121810000

B21 -3.6043250000 4.8100090000 10.0494750000

C23 -1.4472910000 1.3701660000 6.0230040000

H24 -2.3938120000 1.1318120000 5.5257400000

H25 -0.9277090000 2.1358970000 5.4367070000

H26 -0.8284810000 0.4689890000 6.0675440000

C26 -2.1696110000 2.1675090000 13.3110020000

C27 -3.5391110000 2.3335410000 13.0952560000

C28 -3.9550280000 3.1745590000 12.0700190000

C29 -3.0388460000 3.8622150000 11.2494430000

C30 -1.6713420000 3.6467460000 11.4930970000

C31 -1.2239410000 2.8095460000 12.5152470000

N32 -1.7185570000 1.2948390000 14.3992110000

H33 -4.2446140000 1.8052530000 13.7259560000

H34 -5.0191270000 3.2998870000 11.8892020000

H35 -0.9385110000 4.1380350000 10.8573400000

H36 -0.1691510000 2.6426090000 12.7005140000

O37 -0.5043510000 1.1717180000 14.5746420000

O38 -2.5776910000 0.7359670000 15.0844590000

O29 -4.4395990000 5.9585980000 10.7033210000

O1 -2.7236380000 8.3197330000 9.3235990000

C41 -1.4290600000 8.2604800000 10.0893310000

C42 -1.7589300000 8.3928770000 11.5778760000

C43 -2.6156190000 7.1941040000 12.0232180000

H5 -1.0572820000 7.2591950000 9.8591600000

H6 -0.8156480000 8.3880560000 12.1439680000

O46 -2.4393550000 9.6285150000 11.7272700000

H47 -2.0506210000 6.2716580000 11.8442410000

O48 -2.8814700000 7.3558750000 13.3995870000

C10 -0.5651440000 9.3588370000 9.5089780000

H50 0.4384580000 9.2870900000 9.9411210000

H51 -0.4779980000 9.2483370000 8.4244860000

H13 -0.9818230000 10.3404100000 9.7447250000

C15 -3.7815740000 7.8826560000 9.8679940000

C16 -3.9151030000 7.1687200000 11.1723520000

H17 -4.6687240000 7.9689710000 9.2429240000

H18 -4.6896600000 7.6884060000 11.7555580000

C19 -3.3832530000 6.1887420000 14.0515250000

H58 -3.5385960000 6.4674270000 15.0962640000

H21 -4.3339760000 5.8567260000 13.6167250000

H22 -2.6638840000 5.3628350000 13.9943520000

C61 -2.2915540000 10.2794340000 12.9903140000

H62 -2.7745270000 11.2539500000 12.8849670000

H63 -2.7651460000 9.7092470000 13.7925240000

H64 -1.2288160000 10.4302200000 13.2282130000

***meso-*Diol 2**

C1 -2.5297090000 4.1615280000 7.3723420000

C2 -4.0252760000 4.2908680000 7.0133230000

C3 -4.1699940000 5.5331790000 6.1136350000

C4 -3.6827740000 6.8187250000 6.8150820000

C5 -2.1928090000 6.5944990000 7.1645030000

C6 -2.0322300000 5.4028140000 8.1276790000

O7 -1.8156680000 4.0186370000 6.1344820000

H8 -2.3179430000 3.2559310000 7.9502680000

O9 -4.8708100000 4.4681850000 8.1558960000

H10 -4.3272460000 3.4054480000 6.4401100000

H11 -5.1991950000 5.6420530000 5.7618210000

H12 -3.7350000000 7.6371230000 6.0900310000

O13 -4.4628070000 7.1979480000 7.9338500000

O14 -1.4889520000 6.3136630000 5.9374970000

H15 -1.7524920000 7.5091860000 7.5671360000

H16 -2.6628100000 5.5733760000 9.0137840000

O17 -0.6991390000 5.1511180000 8.5222660000

O18 -3.3460300000 5.3121440000 4.9557330000

C19 -1.9884910000 5.1581700000 5.3238140000

H20 -1.4237130000 4.9952910000 4.4085620000

C21 -0.1860540000 6.0678120000 9.4704400000

H22 -0.0578490000 7.0771670000 9.0543820000

H23 -0.8330540000 6.1359300000 10.3598930000

H24 0.7939910000 5.6892820000 9.7716130000

H25 -4.7890790000 3.6912670000 8.7314740000

H26 -4.7663540000 6.3823390000 8.3716780000

**Boronic ester 4**

C1 10.9598920000 1.7879690000 14.4909830000

C2 10.7577700000 0.9179980000 15.7555510000

C3 9.3886110000 1.2835020000 16.3501570000

C4 9.4661020000 2.7529090000 16.7951630000

C5 9.6757990000 3.6115050000 15.5254990000

C6 11.0170190000 3.2888100000 14.8512190000

O7 9.8311500000 1.5647520000 13.6282960000

H8 11.8407660000 1.4665340000 13.9307840000

O9 11.7894170000 1.1376410000 16.7243870000

H10 10.7854280000 -0.1381370000 15.4760950000

H11 9.1361220000 0.6302600000 17.1904100000

H12 8.5380140000 3.0565880000 17.2854310000

O13 10.5305590000 2.9217180000 17.7375480000

O14 8.6068670000 3.2949830000 14.6158430000

H15 9.5961100000 4.6801360000 15.7344520000

H16 11.8464020000 3.4804220000 15.5466550000

O17 11.1401300000 4.1271200000 13.7238900000

O18 8.3663570000 1.1164940000 15.3680450000

C20 8.6265080000 1.9403250000 14.2484340000

H20 7.8240320000 1.7804540000 13.5322940000

B21 11.6331230000 2.1047200000 17.6876470000

C23 12.4488270000 4.2000730000 13.1868830000

H24 13.1827180000 4.5060510000 13.9492440000

H25 12.7765290000 3.2475680000 12.7460600000

H26 12.4196060000 4.9554850000 12.3981160000

C26 14.7702870000 2.6444550000 20.6549650000

C27 14.9077650000 1.6342490000 19.7058650000

C28 13.8997580000 1.4704860000 18.7605850000

C29 12.7631850000 2.2987690000 18.7546600000

C30 12.6641330000 3.3053700000 19.7319440000

C31 13.6614970000 3.4871460000 20.6852300000

N32 15.8328640000 2.8279120000 21.6597310000

H33 15.7872480000 1.0019110000 19.7212440000

H34 13.9917900000 0.6876590000 18.0136370000

H35 11.7925790000 3.9530590000 19.7421890000

H36 13.5969850000 4.2596660000 21.4420800000

O37 15.6877440000 3.7274990000 22.4870600000

O38 16.8020170000 2.0710760000 21.6115000000

**α(1,6)-d-Glucoside boronic ester**

C1 -17.9733340000 3.6996080000 8.6352760000

C2 -18.3829010000 3.9249260000 7.1629410000

C3 -17.1671870000 3.5267110000 6.2871650000

C4 -15.8399210000 4.2461180000 6.6246760000

C5 -15.5754530000 4.0342010000 8.1268670000

C6 -16.7445160000 4.5691730000 8.9734860000

O7 -17.6744760000 2.2988540000 8.7890520000

H8 -18.8046530000 3.9171770000 9.3099060000

O9 -18.9042470000 5.2498730000 7.0717260000

H10 -19.1952320000 3.2319690000 6.9210000000

H11 -17.3865040000 3.5739330000 5.2224960000

H12 -15.0519060000 3.7313220000 6.0749830000

O13 -15.7953440000 5.6500270000 6.3480680000

O14 -15.4267880000 2.6062860000 8.3033760000

H15 -14.6293180000 4.4859840000 8.4331760000

H16 -16.9518150000 5.5891830000 8.6362320000

O17 -16.4772510000 4.7301840000 10.3529620000

O18 -16.9365230000 2.1274710000 6.5936750000

C20 -16.6082210000 1.9300240000 7.9478940000

H20 -16.4316590000 0.8652180000 8.0866000000

B21 -18.6857110000 6.2051770000 6.1320920000

C23 -16.1541840000 3.5804260000 11.1336200000

H24 -16.9707030000 2.8501450000 11.1518440000

H25 -15.2474690000 3.0796930000 10.7767390000

H26 -15.9841940000 3.9575070000 12.1459860000

C26 -20.4115080000 10.1229250000 7.0263650000

C27 -20.1979560000 9.7699010000 5.6966020000

C28 -19.6312540000 8.5282560000 5.4239120000

C29 -19.2816050000 7.6340640000 6.4521130000

C30 -19.5265000000 8.0307060000 7.7796060000

C31 -20.0832820000 9.2706150000 8.0781800000

N32 -21.0076810000 11.4359410000 7.3277880000

H33 -20.4752740000 10.4599670000 4.9088600000

H34 -19.4723120000 8.2437590000 4.3872630000

H35 -19.2807530000 7.3516630000 8.5903060000

H36 -20.2696930000 9.5836190000 9.0985360000

O37 -21.1839770000 11.7259230000 8.5107190000

O38 -21.2942750000 12.1649900000 6.3780110000

C22 -17.0012080000 6.8729280000 2.9778010000

C40 -16.9593540000 6.7053680000 4.5064960000

C24 -15.6363600000 6.0665200000 5.0004390000

C25 -15.1624650000 5.1140750000 2.8089320000

H43 -17.0163580000 7.7092400000 4.9429890000

H27 -15.0276180000 4.0954100000 2.4356240000

O29 -18.0473490000 5.9105320000 4.9481230000

C46 -16.4921810000 5.6160880000 2.2361860000

H31 -16.3324020000 5.8475270000 1.1750980000

H32 -18.0456790000 7.0665110000 2.6883220000

O33 -15.2195780000 4.9613460000 4.2312120000

H50 -14.8581410000 6.8432420000 5.0085460000

C35 -13.9313940000 5.9379190000 2.4036290000

H52 -13.9731250000 6.9543490000 2.8166510000

H37 -13.0373030000 5.4375840000 2.8083770000

O54 -13.8879510000 5.9822690000 0.9873410000

O39 -17.4170700000 4.5415680000 2.3640180000

O40 -16.2052360000 8.0224840000 2.7048290000

C57 -12.7212130000 6.6129950000 0.4951950000

H47 -11.8110400000 6.0791690000 0.8094180000

H48 -12.7835900000 6.5988740000 -0.5960750000

C49 -18.4831080000 4.5632330000 1.4336050000

H61 -19.1479990000 5.4286290000 1.5703750000

H51 -18.1120280000 4.5667840000 0.3966980000

C52 -16.3852960000 8.5938330000 1.4175510000

H53 -15.9478560000 7.9758300000 0.6233670000

H54 -17.4518880000 8.7590010000 1.2023420000

H73 -12.6475910000 7.6568440000 0.8381830000

H74 -19.0628450000 3.6519810000 1.6000940000

H75 -15.8750000000 9.5601470000 1.4336010000

**α(1,4)-d-Glucoside-boronic ester**

C1 -9.4988030000 13.3134520000 -6.0530860000

C2 -8.5107290000 12.3456720000 -5.3862460000

C3 -7.0887360000 12.8204880000 -5.7471910000

C4 -6.8168390000 12.9280620000 -7.2658470000

C5 -7.8915500000 13.8715490000 -7.8456410000

C6 -9.3096700000 13.3253480000 -7.5764370000

O7 -9.2293130000 14.6173490000 -5.5085350000

H8 -10.5333070000 13.0766540000 -5.7967350000

O9 -8.7718550000 11.0209660000 -5.8269180000

H10 -8.6386010000 12.4226990000 -4.2990400000

H11 -6.3210610000 12.2268900000 -5.2463150000

H12 -5.8493460000 13.4324870000 -7.3671860000

O13 -6.7566910000 11.7559780000 -8.0655170000

O14 -7.7324160000 15.1459790000 -7.2018900000

H15 -7.7123200000 14.0405400000 -8.9097110000

H16 -9.3809340000 12.2948330000 -7.9545030000

O17 -10.3310630000 14.1288470000 -8.1310260000

O18 -6.9601940000 14.1656850000 -5.2391340000

C20 -7.9242270000 15.0300110000 -5.8160920000

H20 -7.7750000000 16.0135730000 -5.3763420000

B21 -6.4621880000 10.4369370000 -7.8612010000

C23 -10.5381640000 13.9346330000 -9.5172920000

H24 -9.6714550000 14.2413160000 -10.1210560000

H25 -10.7702720000 12.8826780000 -9.7483200000

H26 -11.3919730000 14.5569270000 -9.7963340000

C26 -4.9283950000 8.3833830000 -11.4003010000

C27 -4.7001040000 7.8325880000 -10.1412800000

C28 -5.2219980000 8.4826740000 -9.0267500000

C29 -5.9531440000 9.6779590000 -9.1505860000

C30 -6.1421800000 10.2074440000 -10.4401590000

C31 -5.6460350000 9.5655340000 -11.5704980000

N32 -4.3955850000 7.6953050000 -12.5879500000

H33 -4.1306890000 6.9148700000 -10.0541990000

H34 -5.0717940000 8.0535720000 -8.0422310000

H35 -6.6850890000 11.1405010000 -10.5559640000

H36 -5.7960250000 9.9612130000 -12.5678760000

O37 -3.7721980000 6.6486180000 -12.4121190000

O38 -4.6066320000 8.2055070000 -13.6882560000

C22 -8.4008250000 8.2393280000 -6.8248770000

C40 -7.4341740000 9.0481520000 -5.9421360000

C24 -8.2074380000 10.0007160000 -5.0149110000

C25 -10.1708700000 8.6629040000 -5.0470440000

H43 -6.8826930000 8.3368070000 -5.3177160000

H27 -10.6835310000 9.3903020000 -5.6912610000

O29 -6.4600890000 9.8168900000 -6.6347210000

C46 -9.4925130000 7.6054550000 -5.9358790000

H31 -9.0155090000 6.8583880000 -5.2913010000

H32 -8.8869170000 8.9092820000 -7.5513480000

O49 -9.1807780000 9.3411420000 -4.2581380000

H50 -7.5187550000 10.4361690000 -4.2843220000

C35 -11.2059500000 8.0947330000 -4.0895440000

H52 -11.5251320000 8.8989480000 -3.4079340000

H37 -12.0852970000 7.7682960000 -4.6670190000

O54 -10.6550380000 7.0138780000 -3.3610380000

O39 -10.4802510000 6.9982990000 -6.7644670000

O40 -7.6494020000 7.2431810000 -7.4976950000

C57 -11.5269870000 6.5325550000 -2.3579260000

H47 -12.4760200000 6.1667590000 -2.7820390000

H48 -11.0174920000 5.7041960000 -1.8590270000

C49 -10.7246110000 5.6248290000 -6.4828780000

H61 -9.8456500000 5.0065160000 -6.7138060000

H51 -10.9982440000 5.4722250000 -5.4317540000

C52 -8.1464750000 6.8587450000 -8.7761360000

H53 -9.1158150000 6.3575620000 -8.7049590000

H54 -8.2428160000 7.7283400000 -9.4412790000

H73 -11.7569290000 7.3120960000 -1.6150680000

H74 -11.5544170000 5.3179450000 -7.1253660000

H75 -7.4060820000 6.1761240000 -9.2001500000

**α(1,6)-d-Glucoside 6**

C1 -2.9126000000 -2.9024100000 11.2765220000

C2 -1.7956520000 -1.8349320000 11.3428630000

C3 -2.4915630000 -0.4884320000 11.6541220000

C4 -3.5225690000 -0.1028220000 10.5744820000

C5 -4.5711040000 -1.2403520000 10.5239990000

C6 -3.9169190000 -2.5789650000 10.1530070000

O7 -3.6010000000 -2.8983900000 12.5448910000

H8 -2.4830190000 -3.9004980000 11.1656710000

O9 -0.9591680000 -1.8257810000 10.2031380000

H10 -1.1566110000 -2.0771210000 12.1976880000

H11 -1.7573220000 0.3094800000 11.7924830000

H12 -4.0222980000 0.8308580000 10.8551410000

O13 -2.7860510000 0.0677210000 9.3578000000

O14 -5.1511220000 -1.3155180000 11.8370070000

H15 -5.4029240000 -1.0264810000 9.8489430000

H16 -3.3798550000 -2.4745010000 9.1987330000

O17 -4.9501350000 -3.5372000000 10.0224160000

O18 -3.2011650000 -0.6324580000 12.8972130000

C19 -4.1802730000 -1.6493020000 12.8002520000

H20 -4.6825170000 -1.7056560000 13.7636490000

C21 -4.5687790000 -4.7058480000 9.3214140000

H22 -3.8036670000 -5.2874450000 9.8560680000

H23 -5.4674350000 -5.3205450000 9.2251650000

H24 -4.1862730000 -4.4681660000 8.3160170000

C25 -1.3438280000 1.1323950000 7.0229150000

C26 -2.5909140000 0.2429830000 7.0083270000

C27 -3.4825850000 0.5290660000 8.2194950000

C28 -2.6696340000 2.7918670000 8.3528380000

H29 -3.1767730000 0.4937230000 6.1103620000

H30 -2.0780180000 2.5621990000 9.2505440000

O31 -2.2436520000 -1.1309350000 7.0045030000

C32 -1.7610810000 2.6030740000 7.1190530000

H33 -2.3224670000 2.8746370000 6.2167600000

H34 -0.7269010000 0.8739470000 7.8955340000

O35 -3.8029590000 1.9000690000 8.2939550000

H36 -4.4453480000 0.0189120000 8.1180990000

C37 -3.1979800000 4.2175920000 8.5064430000

H38 -3.9293740000 4.2360800000 9.3288080000

H39 -2.3535280000 4.8564040000 8.7827070000

O40 -3.7475000000 4.7815490000 7.3289210000

O41 -0.6104670000 3.4311860000 7.2579770000

O42 -0.6382400000 0.8170060000 5.8244850000

C43 -5.1034310000 4.4424720000 7.0723880000

H44 -5.7411270000 4.6844080000 7.9360970000

H45 -5.4187220000 5.0483060000 6.2185810000

C46 -0.5260230000 4.4889390000 6.3076270000

H47 -0.3948550000 4.0987150000 5.2881300000

H48 -1.4175950000 5.1272140000 6.3324170000

C49 0.7799670000 0.9240870000 5.9137350000

H50 1.0958200000 1.9506460000 6.1221100000

H51 1.1785370000 0.2652550000 6.6993050000

H52 -5.2278340000 3.3799610000 6.8348280000

H53 0.3536140000 5.0791450000 6.5780330000

H54 1.1757030000 0.6048290000 4.9458010000

H55 -1.4304940000 -1.3809320000 9.4755430000

H56 -1.5638830000 -1.2166130000 6.3130490000

**α(1,4)-d-Glucoside 5**

C1 -6.7911760000 10.4730300000 4.8402170000

C2 -7.4554930000 9.6703220000 5.9709090000

C3 -7.5181500000 10.5952480000 7.2051930000

C4 -6.1316690000 11.1149760000 7.6515420000

C5 -5.5427760000 11.8752460000 6.4452380000

C6 -5.3808440000 10.9263330000 5.2409880000

O7 -7.6315560000 11.6180940000 4.6050790000

H8 -6.7637120000 9.9107450000 3.9042720000

O9 -6.7066130000 8.4827710000 6.2330290000

H10 -8.4761790000 9.4192040000 5.6585410000

H11 -8.0430560000 10.1120590000 8.0360030000

H12 -6.3141360000 11.8472050000 8.4523590000

O13 -5.2100690000 10.1320310000 8.0753870000

O14 -6.4493540000 12.9421800000 6.1055600000

H15 -4.5981950000 12.3521980000 6.7165290000

H16 -4.7939140000 10.0531930000 5.5567700000

O17 -4.7917150000 11.5405790000 4.1095040000

O18 -8.3076970000 11.7401070000 6.8283890000

C19 -7.7040800000 12.4338090000 5.7491170000

H20 -8.3501330000 13.2759600000 5.5100290000

C21 -3.3857000000 11.6681800000 4.1867690000

H22 -3.0667340000 12.3551510000 4.9844120000

H23 -2.8981810000 10.6939930000 4.3528070000

H24 -3.0534900000 12.0732540000 3.2272630000

C25 -5.3257720000 6.1173230000 7.2823790000

C26 -6.6440120000 6.6892690000 7.8059410000

C27 -7.4319960000 7.3608410000 6.6795500000

C28 -6.4620670000 5.8867790000 5.0549660000

H29 -7.2559170000 5.8591700000 8.1872130000

H30 -5.8634190000 6.7107010000 4.6422380000

O31 -6.4192910000 7.6298240000 8.8520390000

C32 -5.6124180000 5.1588930000 6.1208740000

H33 -6.1775190000 4.2992650000 6.5013940000

H34 -4.6928700000 6.9421620000 6.9258460000

O35 -7.6615400000 6.4310160000 5.6432240000

H36 -8.4264570000 7.6577400000 7.0297500000

C37 -6.8679680000 4.9884520000 3.8872750000

H38 -7.5701220000 5.5413020000 3.2451430000

H39 -5.9667550000 4.7728550000 3.3049990000

O40 -7.4050270000 3.7304900000 4.2577440000

O41 -4.3891640000 4.7297800000 5.5307590000

O42 -4.7108400000 5.4889470000 8.4057740000

C43 -8.7916400000 3.7348970000 4.5681040000

H44 -9.3801400000 4.1579660000 3.7399690000

H45 -9.0816050000 2.6903600000 4.7110000000

C46 -4.2501830000 3.3162110000 5.4193660000

H47 -4.1825210000 2.8435210000 6.4098630000

H48 -5.0862010000 2.8695410000 4.8680770000

C49 -3.2853580000 5.4996860000 8.3973840000

H50 -2.9674720000 5.0426360000 9.3382600000

H51 -2.8824900000 4.9302310000 7.5546100000

H52 -9.0111720000 4.3050850000 5.4778210000

H53 -3.3180280000 3.1380900000 4.8764820000

H54 -2.8973310000 6.5275640000 8.3480210000

H55 -5.6795060000 9.3037240000 8.3003540000

H56 -5.7779260000 7.1961650000 9.4440780000
